# Serum and tear autoantibodies from NOD and NOR mice as potential diagnostic indicators of local and systemic inflammation in Sjögren’s disease

**DOI:** 10.3389/fimmu.2024.1516330

**Published:** 2025-01-28

**Authors:** Shruti Singh Kakan, Sara Abdelhamid, Yaping Ju, J. Andrew MacKay, Maria C. Edman, Indu Raman, Chengsong Zhu, Prithvi Raj, Sarah F. Hamm-Alvarez

**Affiliations:** ^1^ Department of Ophthalmology, Roski Eye Institute, Keck School of Medicine, University of Southern California, Los Angeles, CA, United States; ^2^ Department of Pharmacology & Pharmaceutical Sciences, Alfred E. Mann School of Pharmacy and Pharmaceutical Sciences, University of Southern California, Los Angeles, CA, United States; ^3^ Alfred E. Mann Department of Biomedical Engineering, Viterbi School of Engineering, University of Southern California, Los Angeles, CA, United States; ^4^ Department of Immunology, Microarray and Immune Phenotyping Core Facility, University of Texas Southwestern Medical Center, Dallas, TX, United States

**Keywords:** Sjögren’s disease, autoimmune disease, IgA autoantibodies, IgG autoantibodies, tear film, serum

## Abstract

**Background:**

Sjögren’s Disease (SjD) is an autoimmune disease characterized by lymphocytic infiltration of salivary and lacrimal glands (LG). The LG produces the protein-rich aqueous component of tears, and SjD-associated autoimmune dacryoadenitis (AD) may thus alter tear autoantibody composition.

**Methods:**

The presence of tertiary lymphoid structures (TLS) in LG from two murine models of SjD-associated AD, male non-obese diabetic (NOD) and male non-obese insulitis resistant (NOR) mice, were evaluated using immunofluorescence. IgG and IgA reactivity in serum and tears from these models were probed in three studies against a panel of 80-120 autoantigens using autoantibody microarrays relative to serum and tears from healthy male BALB/c mice. Sources of Ig in tears were investigated using scRNA-Seq of the LG (GSE132420). Data were analyzed by R package Limma and Seurat.

**Results:**

Analysis of immunofluorescence in LG sections from both SjD models showed TLS. Only one autoantibody was significantly elevated in tears and serum in both SjD models across all studies. Three autoantibodies were significantly elevated in serum but not in tears in both SjD models across all studies. Conversely, six IgG and thirteen IgA autoantibodies (6 sharing the same autoantigen) were significantly elevated in tears but not serum in both SjD models. Igha and Ighg2b expressing cells were identified in the plasma cell cluster of NOD.H2b LG.

**Conclusion:**

NOD and NOR mice with SjD-associated AD have distinct autoantibody profiles in tears and serum. Tear IgA isotype autoantibodies showed a greater diversity than tear IgG autoantibodies. TLS observed in LG are a likely source of the tear autoantibodies.

## Introduction

Sjögren’s Disease (SjD) is characterized by autoimmune exocrinopathy of lacrimal glands (LG) and salivary glands (SG), leading to clinical symptoms of dry eye and dry mouth, respectively (1). Extraglandular manifestations including fatigue, musculoskeletal pain, arthralgia, interstitial lung disease, and tubulointerstitial nephritis frequently occur. SjD patients also have a significantly higher risk of developing B-cell lymphoma (2).

Pathogenesis of SjD is complex and includes genetic predisposition, environmental triggers, and activation of innate and adaptive immunity (3–5). Inflammation of LG and SG via infiltrating immune cells result in the activation of apoptotic pathways in glandular epithelial cells, releasing autoantigens such as ribonucleoprotein complexes Ro/SSA and La/SSB, which further recruit dendritic cells to the exocrine glands and lead to a cycle of inflammation (2). Detection of Ro/SSA autoantibodies in serum is part of the clinical diagnosis of SjD (6). Although the presence of serum SSA autoantibody is weighted equally to a positive SG biopsy in the American-European consensus group (AECG) classification criteria for SjD (7), SSA is not specific to SjD and is elevated in other systemic autoimmune diseases (8–10).

Tear autoantibodies may have potential as diagnostic tools denoting ocular involvement in SjD. It is established that tertiary lymphoid structures (TLS) form in the LG and SG in SjD (11). These localized TLS can generate autoantibodies distinct from those found systemically in disease. As the LG is a target organ in SjD, with autoimmune dacryoadenitis (AD) leading to changes in tear composition and volume, it is possible that the presence of specific tear autoantibodies could aid in distinguishing SjD from other autoimmune and dry eye diseases. Small scale studies of autoantibodies in tears (12, 13) and saliva (14) have shown that SSA and SSB antibodies are detected in these biofluids in some seronegative patients, suggesting that tear and salivary SSA and SSB autoantibodies may be more sensitive than serum measurements. Further, symptoms of dry eye and dry mouth correlate highly with markers of inflammation in the tear fluid and saliva, respectively (15).

To understand the similarities and differences between serum and tear autoantibody expression during disease development in SjD, we analyzed tears and serum from two related murine models relative to healthy control BALB/c mice. The male non-obese diabetic (NOD) mouse spontaneously develops SjD-like AD (16) and has served as a source for identification of tear biomarkers that have been validated in human subjects (17–19). The NOD strain is reported to express serum autoantibodies to SSA and SSB (20). To account for potential confounding factors from diabetes development that occur late in this strain, we also used the haplotype-matched NOD-derived male non-obese diabetes resistant NOR/Ltj mouse (21) to investigate autoantibody composition of tear fluid and serum. The NOR mouse is a recombinant congenic strain with limited regions of the NOD/LtJ genome replaced by the genome of the C57Bl/KsJ strain, resulting in maintenance of SjD-like AD but loss of the late-developing diabetes phenotype (21). We have previously characterized SjD-like AD development in both male NOD and NOR mice, which showed marked similarities (22). Both NOD and NOR strains exhibit sexually dimorphic disease development, where males develop earlier and more severe AD than females, and females develop earlier and more severe autoimmune sialadenitis than males (22–25). Since our primary focus is in SjD-associated AD, we used male mice in this study which recapitulate LG disease. Our analysis identified a few autoantibodies common to tears and serum, but, strikingly, found additional discrete autoantibodies elevated in tears or in serum in these models of SjD.

## Methods

### Animals

Three autoantibody microarrays were performed between 2020 and 2022. Both male NOD (#001976, Jackson Laboratory, Sacramento, CA) and male NOR (#002050, Jackson Laboratory, Sacramento, CA) mice were utilized as models of AD in SjD, with healthy male BALB/c (#000651, Jackson Laboratory, Sacramento, CA) as controls. Consistent with previous work (16, 22, 24, 26), NOD/NOR mice aged 8-12 weeks were considered to have early disease, mice aged 12-20 weeks to have intermediate disease, and mice aged >20 weeks to have advanced disease (26). In *Study 1*, serum was collected from male NOD and BALB/c mice with early (n=3/strain) and advanced (n=3/strain) disease. As these studies showed no major differences in serum autoantibody development across this broad age range in NOD mice, we used mice with intermediate disease for subsequent studies to minimize both the minor variability in time of disease onset and comorbidities occurring later in development (27). For *Study 2*, tears and serum were collected from male NOR mice with intermediate disease (n=6) and healthy control male BALB/c mice (n=3). While serum autoantibodies were subjected to a complete analysis, tears were pooled from one, two or three mice to assess the limits of detection. For *Study 3*, tears and serum were collected from male NOD (n=5) and male NOR (n=4) mice with intermediate disease and age-matched male BALB/c mice (n=6). All study procedures conducted on animals were pre-approved by the University of Southern California’s Institutional Animal Care and Use Committee (IACUC) and were conducted in accordance with the GUIDE for Care and Use of Laboratory Animals (8^th^ Edition (28)). ARRIVE checklist is included in **Supplementary Material**.

### Tissue & biofluid collection

Mice were anesthetized with Ketamine (60-70 mg/kg)/Xylazine (5-10 mg/kg). Tears were collected using capillaries as described previously (29) following topical carbachol administration to the LG and flash frozen. 0.5-1 mL of blood was collected by cardiac puncture using a 1 mL syringe and added to MiniCollect 0.8 mL gold cap Serum Separator tubes (Greiner Bio-One, Kremsmünster, Austria). After centrifuging the blood at 4°C, 1500 x g for 15 min, serum was obtained and stored at -80°C. For tissue collection, anesthetized mice were euthanized by cervical dislocation and LG were collected and fixed as described below.

### Immunofluorescence and confocal microscopy

For immunofluorescence labeling, LG were collected from male NOR and NOD mice with intermediate disease. LG were fixed, frozen in OCT, sectioned and prepared for immunolabelling as described (19). Sections were incubated overnight at 4˚C with the following primary antibodies: anti-CD3e (T-cell marker, Hamster Anti-Mouse CD3e 500A2, catalog #553238, BD Biosciences, Franklin Lakes, NJ), anti-IgD (Alexa Fluor^®^ 647 anti-mouse IgD antibody, 11-26c.2a, catalog #405708, Biolegend, San Diego, CA), anti-B220 (B cell marker, rabbit anti-mouse RA3-6B2, catalog #MA5-48137, Thermofisher Scientific, Waltham MA), anti-CD21/CD35 (catalog# 13-0211-82, Thermofisher Scientific) and anti-PNAd carbohydrate epitope (Glycam-1 marker, catalog# 553863, BD Biosciences Franklin Lakes, NJ). Sections without primary antibodies served as negative controls. Slides were washed 3x with 0.5% Tween 20 in PBS and incubated with compatible secondary antibodies and additional probes: Alexa Fluor^®^ 568 goat anti-hamster IgG (1:1000, catalog #A-21112), Alexa Fluor^®^ 488 goat anti-rabbit (1:200, catalog# A-11008), Alexa Fluor^®^ Plus 488 donkey anti-rat (1:200, catalog# A48269TR); Alexa Fluor™ Plus 647 Phalloidin (1:200, catalog# A30107), and DAPI (catalog #D1306), all from Thermofisher Scientific, at 37°C for 1 h then washed 3x with 0.5% Tween 20 in PBS (catalog# 18912014, Thermofisher Scientific). Slides were mounted with ProLong anti-fade mounting medium (catalog# P36934, Thermofisher Scientific). Images were acquired using either a ZEISS LSM 800 confocal microscope equipped with an Airyscan detector (Zeiss, Thornwood, NY) or a ZEISS Axioscan 7 Microscope Slide Scanner and analyzed using open source Qupath software version 0.5.1. and imageJ 1.54g.

### Protein array profiling analysis

Autoantigen microarrays were manufactured in the Microarray & Immune Phenotyping core Facility at the University of Texas Southwestern Medical Center (Dallas, TX, USA) and were processed as described previously (30, 31). A selection of autoantigens was made based on published literature of prior known autoantibodies (32, 33). Positive controls were also imprinted on the arrays. Each slide contained 16 identical arrays, enabling processing of 15 samples and PBS as a negative control. Arrays contained 80-120 autoantigens as well as internal controls. Mouse serum or tear samples were treated with DNAse I to remove free-DNA and then applied to autoantigen arrays at a 1:50 dilution. After incubation, autoantibody binding to the antigens on the array was detected at 532 nm (with Cy3 labeled anti-IgG) and, when IgA was analyzed, at 635 nm (with Cy5 labeled anti-IgA) to generate.tiff images. Genepix Pro 7.0 software was used to analyze the images and generate genepix report (GPR) files (Molecular Devices, Sunnyvale, California, USA). The net fluorescent intensity (NFI) of each antigen was generated by subtracting the local background and negative control (PBS) signal. The signal-to-noise ratio (SNR) was also generated for each antigen. Autoantibodies with SNR values of <3 in more than 90% of the samples were considered negative and excluded from further analysis. NFI was normalized with robust linear model using positive controls with different dilutions. NFI and SNR values as well as the R code used in analyzing the data are described in **Supplementary Methods S1.1-1.6**.

### Bioinformatics & statistical analysis

After filtering autoantibodies with SNR <3 for a given mouse group in each array, remaining NFIs were further normalized by fitting a quantile regression by using the Limma (34) voom package for *Studies 2 and 3* or the code provided in the **Supplementary Methods S1.1** based on Kiripolsky et. al (35). *for Study 1*, depending upon the distribution of the raw data. Normalized values were log transformed and scaled using the biweight midvariance method to account for variances in scaling. Differential autoantigen expression was determined using the Wilcoxon Rank Sum test and correction for multiple comparisons was done using the Benjamini and Hochberg procedure. The Spearman correlation coefficient was calculated for IgG vs IgA in mouse tears in *Study 3.* scRNAseq data was generated by Rattner et al. from LG of 12 week old male NOD.H2b (another NOD substrain lacking diabetes that develops SjD-associated symptoms in LG) (36), and age matched male BALB/c controls. Raw bam files were obtained from the European Nucleotide Archive (ENA: PRJNA548013) and raw fastqs were regenerated using ‘bcl2fastq’ and aligned to mouse genome GRCm39 using 10X’s cellRanger 8.0.1. Raw matrix files were analyzed using Seurat. R code used in analyzing the scRNAseq data are described in **Supplementary Methods S2**.

## Results

### TLS in LG of NOD and NOR

**Figure 1:**
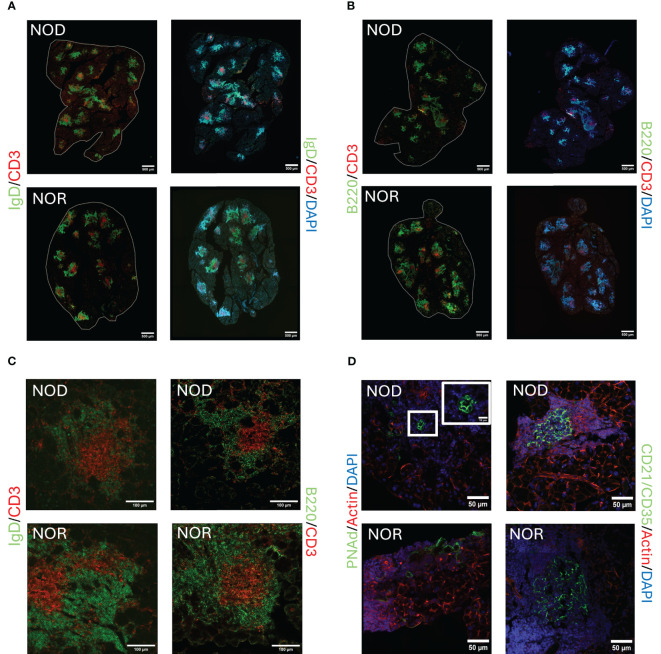
Representative images of tertiary lymphoid structures (TLS) in LG of male NOD and NOR mice with intermediate disease. Immunofluorescence (IF) images show components of TLS as follows:**(A)** Antibodies to IgD highlighting follicular B cells (green) and CD3 highlighting T cells (red). DAPI labels nuclei (blue). **(B)** Antibodies to B220 highlighting B cells, (green) and CD3 highlighting T cells (red). DAPI labels nuclei (blue). Scale bars in **(A, B)**, 500 μm. **(C)** Higher magnification images of representative TLS in NOD and NOR mouse LG. Left panel shows antibodies to IgD highlighting follicular B cells (green) and CD3 highlighting T cells (red). Right panel shows antibodies to B220 highlighting B cells (green) and CD3 highlighting T cells (red). Scale bar, 100 μm. **(D)** Left panel shows antibody to PNAd, highlighting high endothelial venules (green) and Alexa Fluor™ Plus 647 Phalloidin highlighting F-actin (red). DAPI labels nuclei (blue). Right panel shows antibodies to CD21/CD35, highlighting follicular dendritic cells (green) and Alexa Fluor™ Plus 647 Phalloidin highlighting F-actin (red). DAPI labels nuclei (blue). Scale bars, 50 μm.

TLS can form within target organs undergoing chronic inflammatory/autoimmune processes, whose primary function is not lymphogenic in nature. Like germinal centers (GC), they are lymphoid structures where memory lymphocytes and/or precursors can be re-stimulated by antigen which results in clonal expansion (37). We have also shown that miR-155-5p, a marker of GC development (38), is significantly upregulated in the LG of male NOD mice, particularly in the infiltrating lymphocytes (39). In this report, we evaluated the presence of TLS in LG of two murine models of AD in SjD, male NOD and NOR mice. **Figure 1** shows representative images from LG from mice of each strain with intermediate disease. Labeling of lymphocytic infiltrates with IgD, a marker of naive follicular B-cells (40, 41) (**Figure 1A**), and B220, a general marker for B cells (42) (**Figure 1B**), showed enriched B cell zones enveloping CD3e positive T cells (**Figures 1A, B**) in the LG. Some foci showed a clear segregation of T and B cells in a GC-like manner. Higher magnification images of TLS in LG from these mouse strains are shown in **Figure 1C**. These infiltrates were also enriched in other components of TLS including markers of high endothelial venules such as PNAd (43) (**Figure 1D**) and highly compartmentalized CD21/CD35 positive follicular dendritic cells (**Figure 1D**). TLS were detected in LG from both strains at early, intermediate and advanced disease, increasing gradually with age (data not shown). For reference, H&E stained LG sections from these same strains at the same age are shown in **Supplementary Figure S1**.

### Autoantibodies in serum of NOD and NOR mice

Three separate arrays were conducted to compare IgG autoantibody abundance in both tears and serum from male NOD and NOR mice versus healthy control BALB/c mice. A complete list of autoantigens, their abbreviations, and disease association is provided in the **Supplementary Data Sheet S1** file. *Study 1* compared serum autoantibody abundance from mice with early and advanced disease in male NOD mice versus age-matched healthy control BALB/c mice. NOD mice at both disease states exhibited significantly increased serum IgG reactivity against four common autoantigens relative to age-matched healthy male BALB/c mice (**Supplementary Table S1**). These included – Polymyositis/Scleroderma antigen 100 (Pm/Scl-100), Proliferating Cell Nuclear Antigen (PCNA), Anti-liver cytosolic antigen type 1 (LC-1), and Heterodimer Ku protein subunits 70 & 80 (KU P70/P80). Additionally, Small RNA Binding Exonuclease Protection Factor La (La/SSB), threonyl-tRNA synthetase (PL7), Thyroid Peroxidase (TPO), Intrinsic Factor (IF) and Mitochondrial Antigen had significantly elevated serum IgG reactivity at early disease, with greater variability in advanced disease (**Supplementary Table S1**). For each of the nine antigens above, NOD mice with early and advanced disease showed the same significant differences between healthy (BALB/c) and diseased (NOD) mice (**Supplementary Table S1**). Therefore, age was not considered a confounder for development of these autoantibodies. The common elevation of these autoantibodies across the spectrum of SjD-associated AD development suggests that they form early in disease and remain elevated. These findings further suggested that use of mice with intermediate disease might capture changes characteristic of AD in SjD, but avoid confounders associated with advanced disease and development of comorbidities occurring >20 weeks of age (27).

Other autoantibodies were elevated both in the serum of advanced disease NOD mice and age-matched BALB/c mice, namely Signal Recognition Particle 54 kDa protein (SRP54) and Major centromere autoantigen B (CENP-B), (**Supplementary Figure S2**). This suggests their formation is dependent on age rather than on disease progression. Additionally, a slight decrease was observed in PM/Scl-100 and Glycated Albumin autoantibodies with age (**Supplementary Figure S2**).

**Figure 2:**
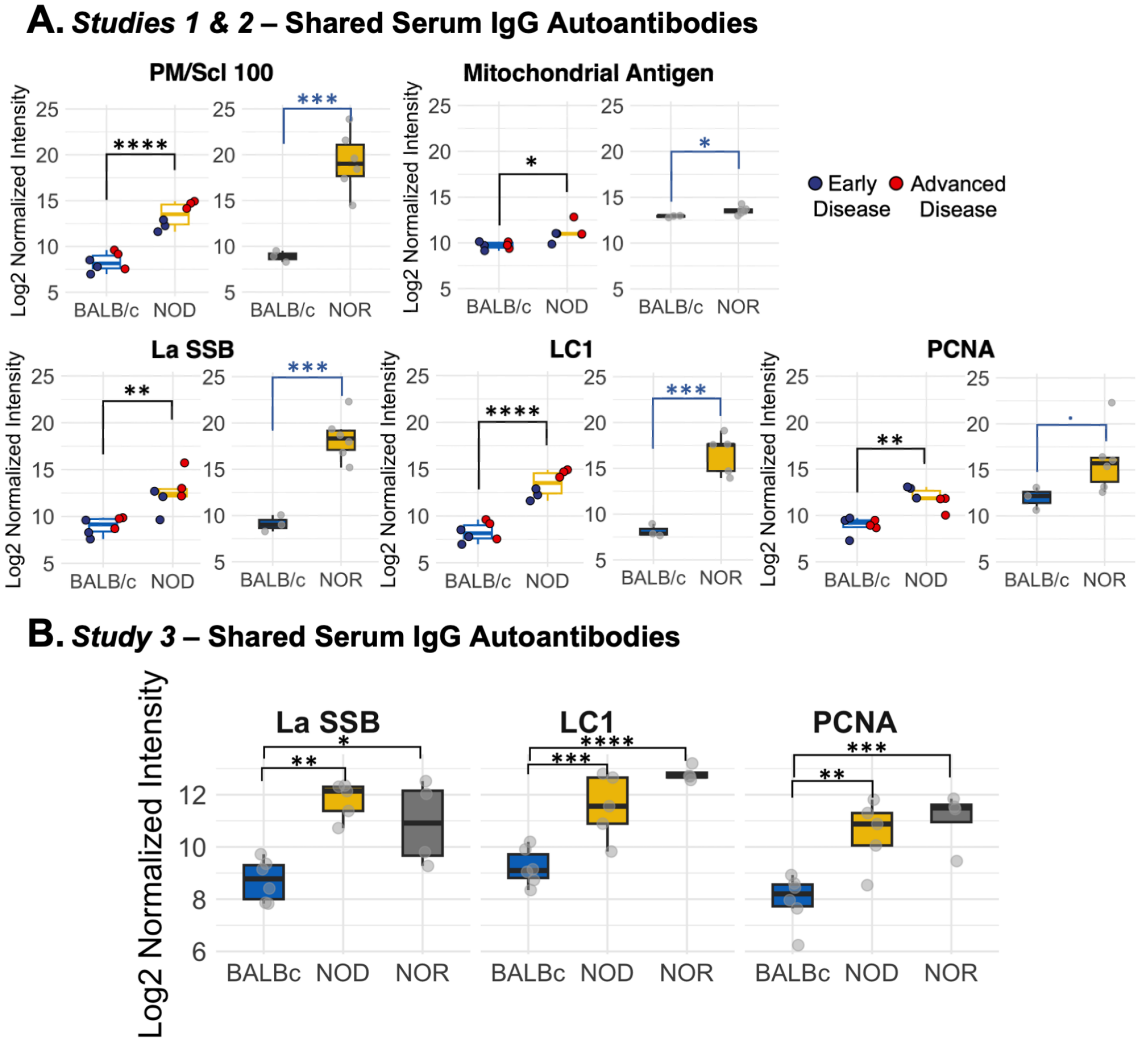
Differential IgG autoantibody expression in serum in male NOD and NOR mice. **(A)** Box plots showing autoantibodies significantly increased in serum of male NOD (N=6) mice as compared to male BALB/c (N=6) mice in two different age groups from *Study 1* (left graphs). Box plot showing log normalized intensities of autoantibodies significantly upregulated in serum of male NOR mice with intermediate disease (N=6) as compared to age-matched male BALB/c mice (N=3) from *Study 2* (right). **(B)** Box plots showing validated serum IgG autoantibodies increased in serum only from male NOD (n=5) and male NOR (n=4) mice with intermediate disease and age-matched male BALB/c mice (n=6) from *Study 3.* Moderated t-statistics estimated using Limma R package. (* p_adj_ < 0.05, ** p_adj_ < 0.01, *** p_adj_ < 0.001, **** p_adj_ < 0.0001). LC1, Anti-liver cytosolic antigen type 1; PM/Scl-100, polymyositis/Scleroderma antigen 100; PCNA, Proliferating Cell Nuclear Antigen; La/SSB, Small RNA Binding Exonuclease Protection Factor La.

In *Study 2*, we used NOR mice with intermediate disease. We identified increased immunoreactivity in NOR mouse serum relative to serum of healthy age-matched male BALB/c mice against sixteen autoantigens (**Supplementary Table S2**). **Figure 2A** shows the combined results of the analyses from *Studies 1 and 2*, which highlights elevations in serum autoantibodies common to both NOD and NOR mice. Common IgG autoantibodies distinct to serum included PM/Scl 100, LC1, La/SSB, Mitochondrial Antigen and PCNA, relative to serum from healthy BALB/c controls. In *Study 3*, we reconfirmed IgG and added IgA immunoreactivity against 80 autoantigens in serum from male NOD and NOR mice with intermediate disease relative to healthy BALB/c controls. This study validated significantly increased serum IgG reactivity against LC1, PCNA and La/SSB in both male NOD and NOR mice (**Figure 2B**) compared to BALB/c. Thus, these serum autoantibodies were common to NOD and NOR mouse serum across all studies. In addition, arrays used in *Study 3* had antigens not present in previous studies including several exclusive to serum (**Supplementary Figure S3**, **Supplementary Table S3**).

Compared to total serum IgG, total serum IgA was detected at a significantly lower level in male NOD and NOR mice (p < 10^-4^, One-way ANOVA, **Supplementary Figure S4A**). The only antigens with significantly elevated IgA reactivity in serum of both NOD and NOR mice with intermediate disease were intrinsic factor (IF) and single stranded DNA (ssDNA) (**Supplementary Figure S4B**). A modest increase was also observed for Cytochrome C (Cyt C), Fibrinogen and Glycoprotein 2 (GP2) (**Supplementary Table S3**).

### Autoantibodies in tears and serum of NOD and NOR mice

*Studies 2* and *3* included tear samples in addition to serum. Several IgG autoantibodies were upregulated in both serum and tears, including autoantibodies to PL7 and KU (P70/P80) in *Study 2* (**Supplementary Figure S5A**), and PL7, Glutamic Acid Decarboxylase 65 (GAD65), and Liver kidney microsome type 1 (LKM1) in *Study 3* (**Supplementary Figure S5B**). The only common autoantibody seen in both *Studies 2 and 3* in serum and tears was to PL7. Additional serum autoantibodies, including against intrinsic factor (IF) may be biologically relevant as they were increased across multiple assays and different antibody isotypes but not significant in *Study 2* (**Supplementary Table S2**).

### Autoantibodies in tears of NOD and NOR mice

**Figure 3:**
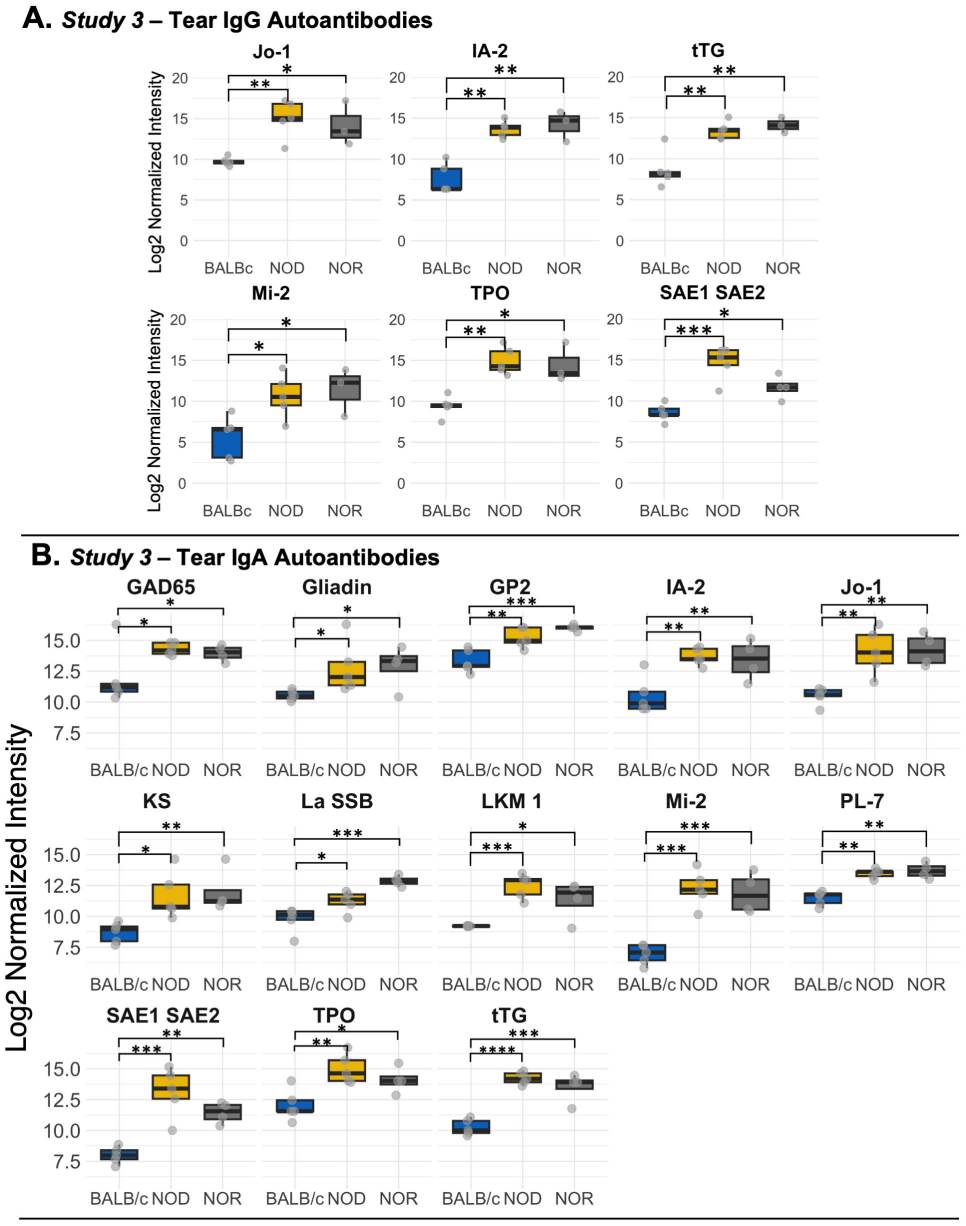
Differential autoantibody expression of autoantibodies unique to tears of male NOD and NOR mice. **(A)** IgG autoantibodies that are unique to tears from male NOD (N=5) and male NOR (N=3) mice with intermediate disease versus male BALB/c (N=3) mice from *Study 3*. Moderated t-statistics estimated using Limma R package. **(B)** Differentially expressed IgA autoantibodies in tears of the same male NOD, male NOR and BALB/c mice from *Study 3*. Moderated t-statistics estimated using Limma R package (ref). p-values adjusted by Benjamini and Hochberg procedure to account for multiple comparisons (* p_adj_ < 0.05, ** p_adj_ < 0.01, *** p_adj_ < 0.001, **** p_adj_ < 0.0001). Jo-1, Histidyl-tRNA synthetase; IA-2, protein tyrosine phosphatase-like Anti-Islet Cell Antigen 512; tTG, Tissue Transglutaminase 2; GAD65, Glutamic Acid Decarboxylase 65; LKM1, Liver kidney microsome type 1; Mi-2, Dermatomyositis specific autoantigen Mi-2; PL-7, threonyl-tRNA synthetase; TPO, Thyroid peroxidase; GP2, Glycoprotein 2; IF, Intrinsic Factor; KS, anti-asparaginyl-tRNA synthetase or KS; La/SSB, Small RNA Binding Exonuclease Protection Factor La; SAE1/SAE2, SUMO1 activating enzyme subunit 1/2.

We explored the tear autoantibody profile of IgG and IgA isotypes in tears of both male NOD and NOR mice with intermediate disease versus tears of healthy control BALB/c mice (**Table 1**). In *Study 3*, we found 6 IgG autoantibodies that were significantly upregulated in tears of both NOD and NOR mice. These include autoantibodies to Histidyl-tRNA synthetase (Jo-1), Anti-Islet Cell Antigen 512 (IA-2), Tissue Transglutaminase 2 (tTG), Dermatomyositis specific autoantigen Mi-2 (Mi-2), thyroid peroxidase (TPO) and SAE1/SAE2 (**Figure 3A**). In *Study 3*, IgA reactivity was tested against the same panel of 80 autoantigens. There was significantly higher IgA reactivity against 14 autoantigens in both male NOD and NOR mouse tears relative to healthy BALB/c tears, of which 13 were unique to tears. These included autoantibodies to tTG, Mi-2, Jo-1, IA-2, GP2, SAE1/SAE2, TPO, PL-7, KS, GAD65, LKM1, La/SSB and Gliadin (**Figure 3B**). While there was largely an agreement between the upregulated IgG and IgA autoantibody species (**Table 1**), tears of male NOR mice had significantly higher total IgA than total IgG (padj = 0.0495, two way ANOVA, post-hoc dunn's test), with comparable levels of IgA and IgG in the healthy control male BALB/c (**Supplementary Figure S6A**). Reactivity to IgA control was significantly higher than reactivity to IgG controls in tears of all mice but was relatively higher in the male NOD and NOR mice compared to BALB/c (**Supplementary Figure S6B**). Positive correlations between the signal intensities for IgGs and IgAs to the same autoantigens were observed for 70 out of 80 autoantigens tested (**Supplementary Methods S1.4**) 56 of which were statistically significant. The highest positive correlations were observed for IgGs and IgAs to the six shared epitopes in NOD and NOR, but not for BALB/c controls (**Supplementary Figure S7**).

**Table 1:** Differentially Expressed IgG & IgA Autoantibodies in Tears of Mice from *Study 3*.

	NOD vs BALB/c				NOR vs BALB/c			
	IgG		IgA		IgG		IgA	
	p_adj_	Log_2_ FC	p_adj_	Log_2_ FC	p_adj_	Log_2_ FC	p_adj_	Log_2_ FC
**tTG**	**7.53 x 10^-4^**	5.40 (42.34)	**3.00 x 10^-5^**	3.94 (15.32)	**0.0019**	5.14 (35.22)	**3.31 x 10^-4^**	3.36 (10.28)
**Mi-2**	**0.0083**	5.17 (36.13)	**1.02 x 10^-4^**	5.33 (40.13)	**0.0073**	5.54 (46.5)	**3.64 x 10^-4^**	5.08 (33.75)
**Jo-1**	**7.27 x 10^-4^**	5.77 (54.75)	**0.0017**	3.58 (11.98)	**0.0023**	5.16 (35.83)	**0.0019**	3.51 (11.36)
**IA-2**	**2.55 x 10^-4^**	6.81 (112.4)	**0.0025**	3.17 (9)	**0.0013**	6.18 (72.71)	**0.0042**	3.06 (8.32)
**GP2**	0.0901	2.22 (4.67)	**0.0075**	1.86 (3.63)	**0.0020**	4.27 (19.31)	**5.97 x 10^-4^**	2.73 (6.61)
**SAE1 SAE2**	**1 x 10^-4^**	9.21 (590.94)	**1.51 x 10^-4^**	5.04 (32.85)	**0.0470**	4.56 (23.56)	**0.0056**	3.48 (11.16)
**IF**	0.2036	1.96 (3.90)	0.0942	1.35 (2.54)	**0.0018**	5.20 (36.75)	**0.0011**	3.08 (8.44)
**TPO**	**7.63 x10^-4^**	5.58 (47.9)	**0.0021**	2.94 (7.67)	**0.0107**	4.3 (19.66)	**0.0190**	2.18 (4.53)
**PL-7**†	**0.0299**	3.19 (9.14)	**0.0030**	2 (4.01)	0.0862	2.63 (6.17)	**0.0015**	2.26 (4.77)
**KS**	0.1420	2.71 (6.54)	**0.0127**	3.07 (8.41)	**0.0131**	4.57 (23.7)	**0.0099**	3.41 (10.64)
**GAD65**	**0.0075**	4.12 (17.37)	**0.0150**	2.44 (5.41)	0.0728	2.81 (6.99)	**0.0434**	2.08 (4.22)
**GP210**	0.0927	4.44 (21.67)	0.5130	0.77 (1.71)	**0.0041**	5.86 (50.67)	**0.0010**	2.82 (7.06)
**LKM 1**	0.0926	2.39 (5.23)	**7.38 x 10^-4^**	3.24 (9.44)	0.1935	1.68 (3.20)	**0.0103**	2.39 (5.24)
**gDNA**	**0.0116**	2.99 (7.93)	0.204	0.99 (1.98)	**0.0078**	3.33 (10.05)	0.0579	2.87 (7.31)
**Gliadin**	0.0536	3.91 (14.99)	**0.0462**	2.32 (4.99)	0.0592	3.84 (14.27)	0.0503	2.37 (5.18)
**La SSB**†			**0.0419**	1.57 (2.97)	**0.0430**	2.97 (7.84)	**5.28 x 10^-4^**	3.2 (9.18)
**NXP2**	0.4657	1.33 (2.52)	–	–	**0.0010**	6.36 (82.09)	**0.0056**	3.55 (11.71)
**PM Scl75**	**0.0158**	5.73 (52.99)	0.0615	1.91 (3.76)	0.0627	4.5 (22.68)	–	–
**T1F1 g**	0.1704	3.61 (12.23)	–	–	0.1486	3.81 (14.04)	0.3067	0.80 (1.75)
**LC1**†	–	–	0.167	1.11 (2.16)	–	–	0.0695	1.55 (2.93)
**dsDNA**	–	–	0.0980	1.18 (2.26)	–	–	**0.0108**	1.89 (3.7)
**TNF a**	–	–	**0.0319**	3.08 (8.46)	–	–	0.288	1.67 (3.18)
**Cardiolipin**	0.0798	3.84 (14.33)	0.363	1.05 (2.07)	**0.0073**	5.87 (58.58)	0.0898	1.91 (3.76)
**SmD1**	**0.0025**	5.84 (57.16)	–	–	0.0592	3.67 (12.71)	–	–
**KU P70/P80**†	0.1778	1.97 (3.93)	0.0533	1.62 (3.08)	**-**	–	0.0862	1.53 (2.88)

p_adj_ <0.05 was considered significant, multiple comparisons p-values were adjusted by the Benjamini-Hochberg procedure. Statistically significant p-values are shown in bold.

†Also upregulated in serum of male NOD and NOR mice compared to healthy BALB/c. Fold Change = 2 ^ (Log2 Fold Change).

## Discussion

**Table 2** summarizes all of the significant autoantibody findings from the study. Serum expression of SSA and SSB autoantibodies is observed in ~25-75% of SjD patients (44, 45), respectively, but are also detected in patients with systemic lupus erythematosus and rheumatoid arthritis (9, 46). This lack of sensitivity and specificity is a challenge for SjD diagnosis. The identification of additional potential diagnostic autoantibodies in SjD has been previously investigated, with researchers reporting serum autoantibodies to SP1, Ca6 or PSP with better sensitivity than antibodies to Ro or La (47). In this study we show for the first time that two related murine disease models of SjD exhibiting AD, the male NOD and NOR mice, express characteristic serum and tear autoantibodies that are largely distinct. The autoantibodies unique to serum were primarily IgG autoantibodies but the autoantibodies unique to tears included IgG and IgA isotypes. Interestingly, the autoantigens recognized by tear IgGs were all represented in tear IgAs.

**Table 2:** Summary of upregulated IgG and IgA autoantibodies in serum only, tears only or in both serum and tears.

Autoantigen	IgG		IgA		Sub-Cellular Localization
	NOD	NOR	NOD	NOR	
BPI *Bactericidal/permeability-increasing protein*	S	S			Secreted
BCOADC-E2	S	S			Nucleoplasm
FBG IV – Fibrinogen IV	S	S			Secreted
FBG S – Fibrinogen S	S	S			Secreted
GAD65 *Glutamic Acid Decarboxylase 65*	S T	S T	T	T	Cytosolic
IA-2 *Islet Cell Antigen 512*	T	T	T	T	Secreted
IF *– Intrinsic Factor*		S	S	S	Secreted
Jo-1 *Histidyl-tRNA synthetase*	T	T	T	T	Cytosolic
KS – *anti-asparaginyl-tRNA synthetase*	S		T	T	Cytosolic
KU P70/P80 – *Heterodimer Ku protein*	S	S			Nuclear
La/SSB	S	S	T	T	Nucleoplasm
LKM 1 – *Liver kidney microsome type 1*	S T	S T			Cytosolic
LC1 – *Anti-liver cytosolic antigen type 1*	S	S			Cytosolic
Mitochondrial antigen	S	S			Mitochondria
Mi-2	T	T	T	T	Nuclear
PDC-E2 *Pyruvate dehydrogenase complex component E2*	S	S			Mitochondria Nucleoplasm
PCNA – *Proliferating Cell Nuclear Antigen*	S	S			Nucleoplasm
PM/Scl-100 – *Polymyositis Scleroderma antigen 100*	S	S			Nucleolus
PL-7 *– threonyl-tRNA synthetase*	S T	S T	T	T	Cytosolic
PM Scl75 – *Polymyositis Scleroderma antigen 75*	T	T	T		Nuclear
P0 – Ribo phospho protein P0	S	S			Cytosolic
SAE1/SAE2 – *SUMO1 activating enzyme subunit 1/2*	T	T	T	T	Nuclear
Sm RNP – small nuclear ribonucleoprotein	S	S			Nuclear
SP100 *– Anti sp100 nuclear antigen*	S	S			Nuclear
SmD1	T	T			Nuclear
ssDNA – Single Stranded DNA			S	S	Extracellular
T1F1 γ - Dermatomyositis Autoantigen	T	T			Nuclear
tTG – Tissue Transglutaminase	T	T	T	T	Cytosolic
TPO – Thyroid Peroxidase	T	T	T	T	Cytosolic, Nucleoplasm

T, Tear; S, Serum.

While murine models do not completely recapitulate all features of human disease, the NOD mouse in particular has been used to generate many findings validated in SjD patients, and/or to recapitulate findings made in SjD patients, particularly with respect to LG and SG disease. NOD mice exhibit significant AD and autoimmune sialoadenitis, as well as reduced tear and salivary flow (22, 48–52), which are defining characteristics of SjD patients. A series of novel autoantibodies to three salivary gland proteins, salivary gland protein 1 (SP-1), carbonic anhydrase 6 (CA6) and parotid secretory protein (PSP) identified in 2 SjD murine models including the NOD mouse were subsequently validated in 45% of SjD patients meeting clinical diagnostic criteria who lacked autoantibodies to Ro or La (47). In particular, these anti-CA6 autoantibodies appear to delineate a particular SjD population with DED having younger disease onset and more severe disease (53). NOD mice also provided initial evidence for elevated cathepsin S levels (29) and decreased cystatin C levels (17) in tears, both of which which were subsequently validated in SjS patients (17, 18). NOD mice also provided evidence for mislocalization of aquaporin 5 in exocrine glands (54), which again is seen in SjD patients (54). While exploration of the expression of these new autoantibodies identified in NOD and NOR mice in the tears and serum of SjD patients is a future goal, past similarities in disease manifestation and biomarker discovery and validation suggest our findings have potential for clinical impact.

Tear biomarkers are of increasing interest in vision science and other diseases, since changes in the tear film are correlated with multiple conditions ranging from SjD (17, 18), dry eye disease (55), Parkinson’s disease (56, 57) and Alzheimer’s disease (58). Tear biomarker tests offered in a clinical laboratory-based format could use collection methods already in clinical use in optometry and ophthalmology clinics. For instance, the Schirmer’s test uses a filter-paper strip marked in mm to measure tear flow, and many biomarker discovery studies are based on adaptation of this strip for sample collection, and subsequent elution and measurement of the marker of interest. The currently approved InflammaDry test is a clinically-approved point of care test (55) that measures increased matrix metalloproteinase 9 levels indicative of dry eye disease. The InflammaDry test uses microfiltration technology on a Schirmer’s-like collection strip combined with a simple colorimetric indicator that can be visually assessed relative to a standard, similar to a pregnancy or COVID-19 test. Such tests would not require special equipment and could be readily incorporated into specialties such as rheumatology, with minimal additional training. Ongoing challenges in the tear biomarker field include those of standardization, which may be specific to the nature of the biomarker (59). Given the limited volume available in patients with dry eye disorders, like SjD, sensitivity of detection of the biomarker is also important. We have focused on analysis of autoantibodies in mice of intermediate disease, after confirming similar patterns of serum autoantibody expression in early and late stage disease in *Study 1*. The common SjD serum autoantibodies (SSA, SSB, ANA, RF) are found in patients as early as 20 years prior to development of disease signs and symptoms. A study by Theander et al. showed that at least one autoantibody was detected pre-disease in 80% of patients (60). Although autoantibody titers may vary, the autoantibody profile is often stable over time (61). As we did not detect significant differences in the serum autoantibody profile between early and advanced disease groups in *Study 1*, we chose to focus on intermediate disease. We felt that mice at this stage would 1) have a more robust formation of LG TLS than at early stages, and 2) might exhibit fewer confounders associated with development of symptoms in other organs. Since tear autoantibodies are less well understood, it is possible that their autoantibody profile is more dynamic than in serum. Future studies will explore the time course of development of these autoantibodies across a more comprehensive disease range.

IgA is the second most common antibody isotype in serum, after IgG (62). Nearly 90% of the IgA found in serum is monomeric IgA1, produced in the bone marrow, which plays no role at the mucosal surface. However, the IgA found in mucosal tissues, such as the LG is secreted as dimeric IgA (dIgA), produced by resident plasma cells in the glandular interstitium. dIgA binds to polymeric Immunoglobulin Receptor (pIgR) expressed on the basolateral membrane of polarized LG acinar cells and is transcytosed to the apical surface where pIgR is cleaved and dIgA is released into tears as secretory IgA (sIgA) (63). In SjD, TLS are found in LG (64). IgA autoantibodies generated by solitary plasma cells at early disease stages as well as from local TLS in established disease would therefore be recovered in tears due to this robust active transport process.

IgG present in tears is either produced locally in the LG or originates from the circulation. Since distinct IgGs are present in tears relative to serum, it is unlikely that tear IgGs originate from the circulation. Previous studies have shown that the number of IgG producing plasma cells in salivary gland biopsies from SjD patients increases with disease progression (62), reducing the IgA to IgG ratio. We have analyzed published data from a single cell RNA-Seq study on LG from healthy male BALB/c and male NOD.H2b disease model mice (another NOD substrain lacking diabetes that develops SjD-associated LG disease) (36). As shown in **Supplementary Figures S8A, B**, the population of naïve B cells and plasma cells in the LG is markedly increased in NOD mouse, and NOD mouse plasma cells show a significant increase in IgG production (based on Ighg2b gene expression). Analysis of genes encoding IgA, IgM and IgG genes in different B cell populations from the LGs support the increased local expression of IgG (**Supplementary Figure S8C**). IgG is not known to be actively transported, and its presence in tears is likely due to passive paracellular transport, a pathway recently reported to occur in LG (63). Paracellular leakage may increase with SjD progression. Disruption of tight junctions is demonstrated in salivary glands of SjD patients and NOD mice, facilitated by proinflammatory cytokines such as Il-17A. degradation of extracellular matrix, epithelial cell apoptosis, and diminishing tissue organization (61).

The plausibility of detecting SjD autoantibodies in tears was previously shown in a small study by Toker et. al (12) that demonstrated that roughly 16 of the 28 patients positive for serum SSA and 14 of the patients positive for serum SSB were positive for SSA and SSB respectively, in tears. However, this study only investigated IgG autoantibodies. The Toker study and similar studies conducted in saliva have also shown the presence of SSA and SSB in tears or saliva of seronegative patients (12–14). To our knowledge, no investigations have studied the complete spectrum of autoantibodies present in tears, nor compared them to the spectrum of serum autoantibodies in SjD patients or SjD disease models. We found that the autoantibody repertoire of male NOD and NOR mice tears was quite distinct from that found in their serum. Increased IgG and IgA reactivity to Jo1, IA2, tTG, Mi-2, TPO and SAE1/SAE2 was seen only in tears, but not in serum.

In the healthy eye, conjunctiva associated lymphoid tissue (CALT) comprises a secondary lymphoid organ (SLO) that is crucial to ocular defense against pathogens. Changes in number and size of CALT in healthy mice are seen upon exposure to pollen (65), induction of dry eye disease (65) or with age (66). The autoantibodies observed in the tears of healthy male BALB/c mice in our study may be produced by CALT, in addition to resident LG plasma cells in order to maintain ocular health. On the other hand, TLS, while similar to SLO, develop in organ-specific autoimmune diseases such as SjD (37, 67) where they are not normally present. Their formation involves B-cell activation and differentiation into autoantibody producing plasma cells via a series of somatic hypermutation and class switch recombination of the Ig genes (1). These structures actively support molecular machinery responsible for pathogenic autoantibody production. Our data show the presence of characteristic TLS within LG of male NOR and NOD mice. We also observe expression of other components of TLS including PNAd, a marker of high endothelial venules in the dark zone (43), and CD21/CD35, a marker of follicular dendritic cells of the light zone (68). The tear-specific IgA and IgG autoantibodies seen in NOD and NOR mice may be largely produced from LG TLS, although a recent study has also suggested that CALT of patients with autoimmune eye-related diseases including SjD and graft-versus-host disease can produce tear autoantibodies to citrullinated proteins (69). In either case, tear IgA autoantibodies appear to be generated locally since they differ both in isotype and antigen from those expressed in serum in the same animals.

An important question is to understand how tear autoantibodies are related to disease development and progression in SjD. Many studies have suggested that the autoantigens triggering production of SSA and SSB autoantibodies, Ro and La, are released locally in exocrine tissue due to early disease triggers (viral infection, ER stress, apoptosis) where they can be processed in TLS to generate autoantibodies (70). Other anatomical features of TLS suggest that local TLS are most likely to express local autoantigens (37). For instance, early events in antigen presentation in exocrine glands are facilitated by the epithelial cells themselves, through the process of autoimmune epithelitis (71). TLS may have poorly developed lymphatics relative to the more organized SLO in lymphoid tissue. In the absence of these lymphatics, mature dendritic cells carrying antigen from other peripheral sites may not be able to reach TLS.

With this process in mind, we considered the common IgA and IgG tear autoantigens– Jo-1, IA2, tTG, Mi2, SAE1/SAE2 and TPO. Synergism of IgG and IgA autoantibodies to common autoantigens is known to potentiate interferon responses in systemic lupus erythematosus (72). Given the high degree of correlation observed between IgG and IgA for these six shared antigens in tears of SjD model mice, their role in synergistically propagating autoimmunity in LG requires further investigation. The same apoptotic processes that may lead to exposure of Ro and La may expose additional autoantigens leading to autoantibody development to Jo-1, tTG, Mi2 and SAE1/SAE2. Anti-Jo-1 IgG and IgA autoantibodies target histidyl-tRNA synthetase (73), a major biosynthetic enzyme. Serum IgG elevation of this antigen is associated with anti-synthetase syndrome (74–76). Mi2 autoantigen is a key marker of antimitochondrial autoantibodies. These antibodies have been strongly associated with primary biliary cholangitis (44). Notably, anti-Jo-1 and Mi2 IgG were found to be absent in sera of 26 SjD patients and were only detected in patients with myositis (77), Similarly, anti- SAE1/SAE2 IgG is reported to be exclusively found in serum of patients with Myositis. Tissue transglutaminase (tTG) is an abundant protein found in multiple tissues in both intracellular and extracellular spaces that catalyzes protein cross linking (78). IgA autoantibodies to tTG are commonly used to diagnose celiac disease in children (79). However, it is notable that autoantibodies to this protein are detected in saliva from SjD (80).

It is unclear how local autoantigens could contribute to tear autoantibodies to thyroid peroxidase (TPO), a tissue-restricted thyroid secretory protein important in generation of thyroglobulin or IA-2, an islet-specific autoantigen for type 1 diabetes (81, 82). Autoantibodies to TPO are commonly seen in autoimmune thyroid diseases such as Hashimoto’s disease (83). Interestingly, diabetes, autoimmune thyroiditis, myositis (84, 85) and primary biliary cholangitis (associated with autoantibody to Mi2, above) have been explored as potential co-morbidities in SjD (27, 86), Autoimmune thyroiditis is a co-morbidity in the NOD mouse in 5-15% of animals (27), while diabetes is a defining phenotype in the NOD mouse. While the presence of autoantibodies to these proteins is unsurprising given co-morbidities of the disease model, their concentration in tears instead of in serum is surprising.

Some non-traditional serum autoantibodies can be associated with SjD in patients with overlapping co-morbidities (87). What is unusual about our findings is the discovery of these autoantibodies in tears. All of our results suggest that tear autoantibodies are produced locally, implying that they originate in TLS present in the LG. LG TLS are unique to the SjD disease phenotype. Thus, the detection of these autoantibodies in tears should be highly specific for SjD while also suggesting the possible involvement of comorbidities in other organs. Although serum autoantibodies are a common finding in rheumatic autoimmune diseases, their exact role in pathogenesis remains unclear. Autoantibodies against extracellular regions of proteins can cause pathogenicity if they disturb the function of the target protein. In fact, this has been proposed as one way to distinguish the pathogenic versus non-pathogenic potential of antibodies, by evaluating whether autoantigen is exposed or present within cells (88). Other work even categorizes the types of pathogenicity associated with different types of autoantibody interactions with external epitopes (89). Most of the antigens identified in this study are directed against intracellular proteins, avoiding immediate categorization as potentially pathogenic. However, despite evidence that the autoantibodies present in serum, tears, and saliva are produced in the affected exocrine glands (71), the direct contribution of autoantibodies to exocrine gland dysfunction in SjD has not yet been shown. It is becoming increasingly clear that the exocrine epithelial cells play a crucial role in in the pathogenesis of the disease, as they accrue antigen-presenting properties. They may also facilitate exposure of intracellular antigens by secretion of exosomes containing autoantigenic proteins, such as Ro/SSA and La/SSB or through the release of autophagic blebs containing antigenic proteins due the enhanced cell death. The presence of certain autoantibodies have also been linked to specific clinical manifestations (87). Some autoantibodies present in tears may also have direct pathological effects on the ocular surface; for instance, Kwon et al. showed that anti-citrullinated protein antibodies in tears contribute to ocular surface inflammation in autoimmune-mediated dry eye (69).

Previously, studies have found salivary anti-muscarinic receptor autoantibodies had higher sensitivity in diagnosing younger patients with SjD at an earlier stage of disease progression (90). Another study validated salivary anti-histone and anti-tTG autoantibodies in 34 patients with SjD with an area under the receiver operator curve (AROC) of 0.87 or higher (80), which is significantly better than the AROC for serum SSA and SSB. Adding tTG and histone autoantibodies to salivary Ro and La gave an AROC of 0.99 (80). This study also found significant elevation of anti-cardiolipin autoantibodies in SjD patient saliva when compared to healthy controls and SLE patients. This lends further credence to the utility of testing local biofluids in SjD. Thus, we propose tear autoantibodies may outperform serum biomarkers in sensitivity and specificity in reflecting ocular manifestations of SjD. Future studies will explore additional aspects of tear autoantibody development in mice including variability in their time course of expression, and their sensitivity to treatments that reduce TLS in the LG. Importantly future studies will also explore their abundance in tears of SjD patients.

## Data Availability

The datasets analyzed for this study can be found in the GEO: GSE286502, GSE286503, GSE286504.
