# Supplementary material for: Serum and tear autoantibodies from NOD and NOR mice as potential diagnostic indicators of local and systemic inflammation in Sjögren’s disease
Source: Front Immunol. 2025 Jan 28;15:1516330. doi: 10.3389/fimmu.2024.1516330 (PMC11810956; doi:10.3389/fimmu.2024.1516330)
Supplement: Supplementary file 1 [file DataSheet1.docx]

| 1. NOD 20 weeks | 1. NOR 20 weeks |
| --- | --- |
| 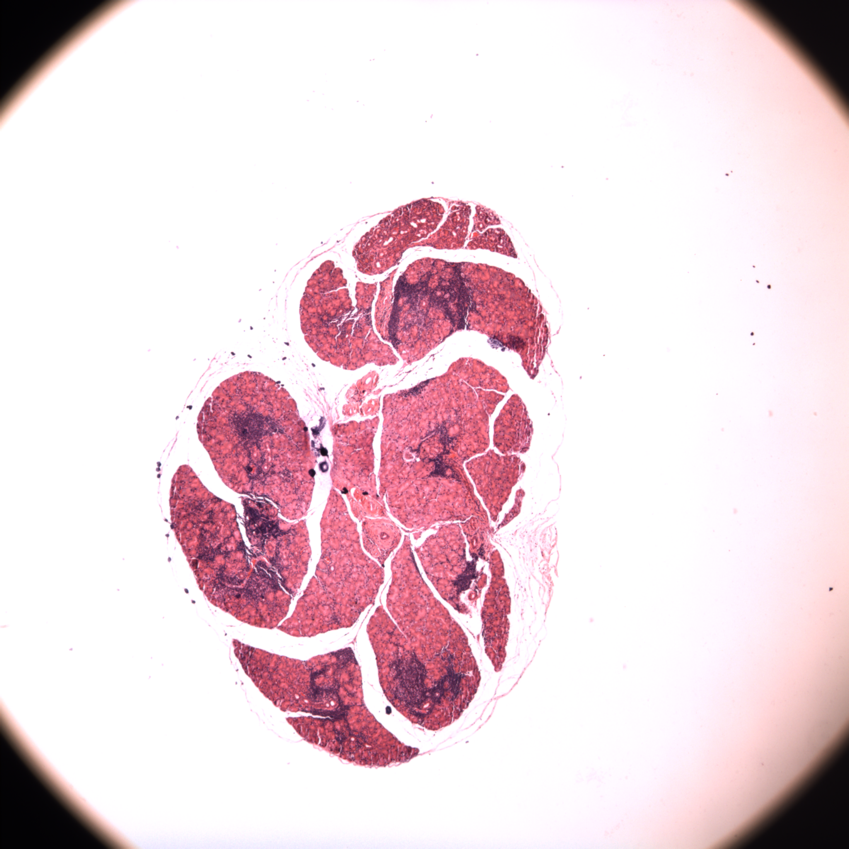 | *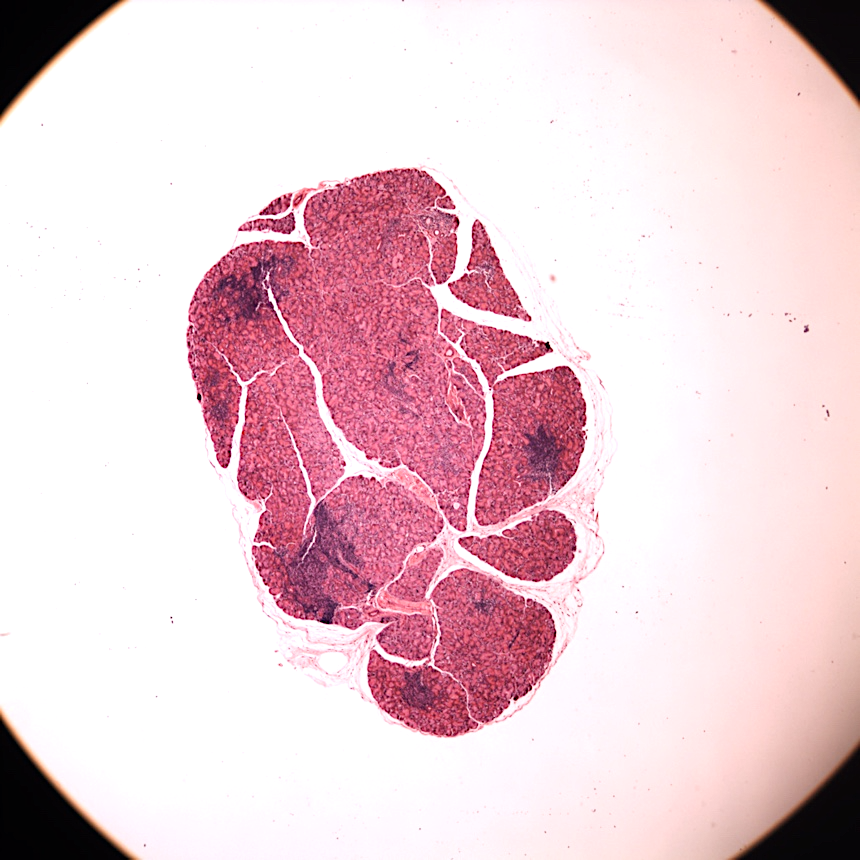* |
| **Supplemental Figure 1. Hematoxylin and Eosin (H&E) stained cross-sections of mouse lacrimal glands from** (A) 20 week old male NOD and (B) 20 week old male NOR mice, showing foci of infiltrating lymphocytes (in purple). The cross-section of the LG is approximately 4 mm long and 2 mm wide. | |

*Study 1 –* Serum IgG


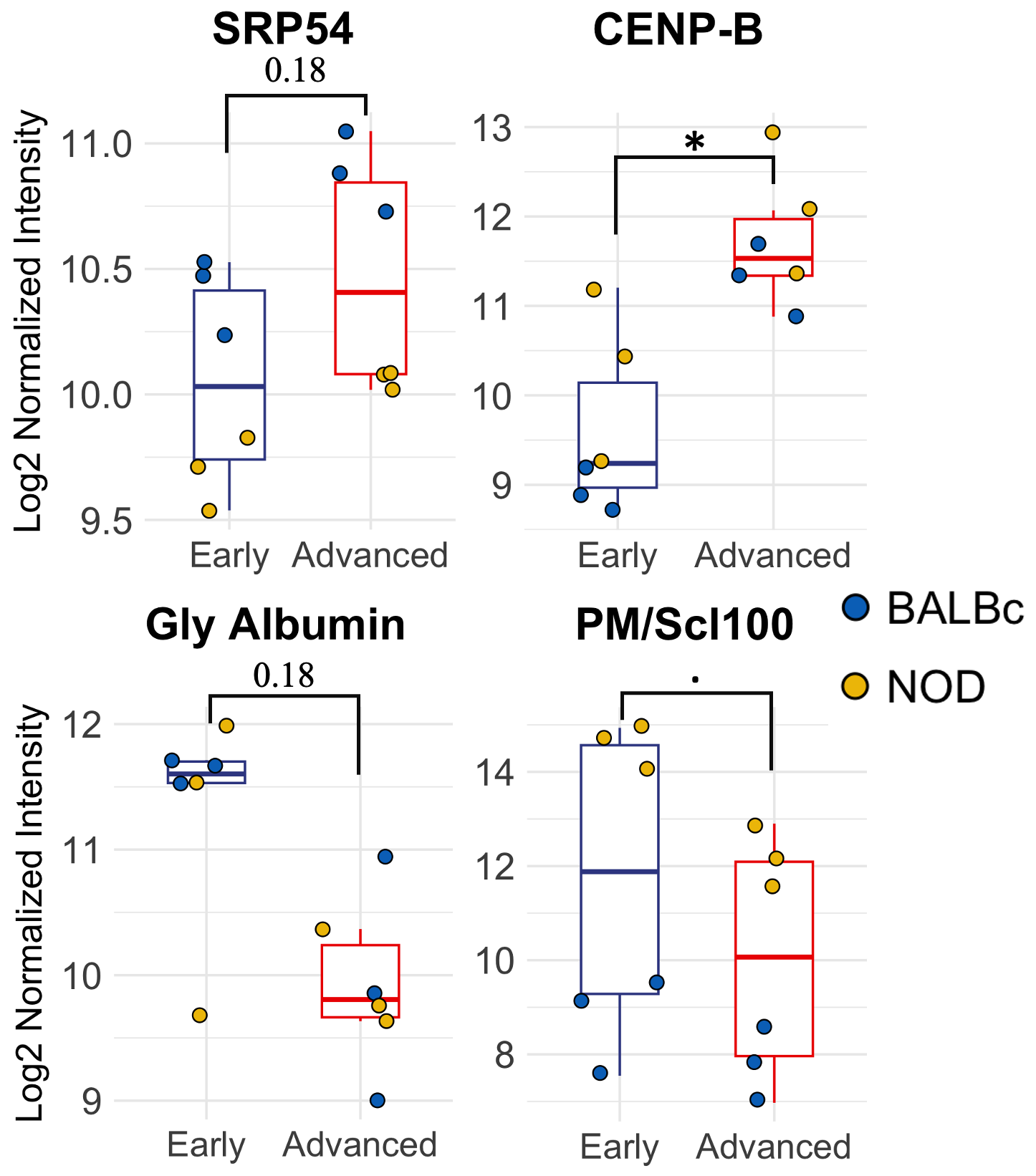


**Supplemental Figure 2.** Effect of age on serum IgG autoantibodies in male NOD mice with early disease (n=3) and advanced disease (n=3) versus BALB/c mice (n=3 per age group matched to NOD mice). Each point represents one mouse. Moderated t-statistics estimated using Limma R package. ( . padj < 0.1, * padj < 0.05, ** padj < 0.01, *** padj < 0.01).

*SRP54 – Signal Recognition Particle 54 kDa protein; CENP-B – Major centromere autoantigen B (Centromere protein B); PM/Scl-100 – polymyositis/ Scleroderma antigen 100; Gly Albumin – Glycated Albumin;*

| *Study 3* – Serum IgG  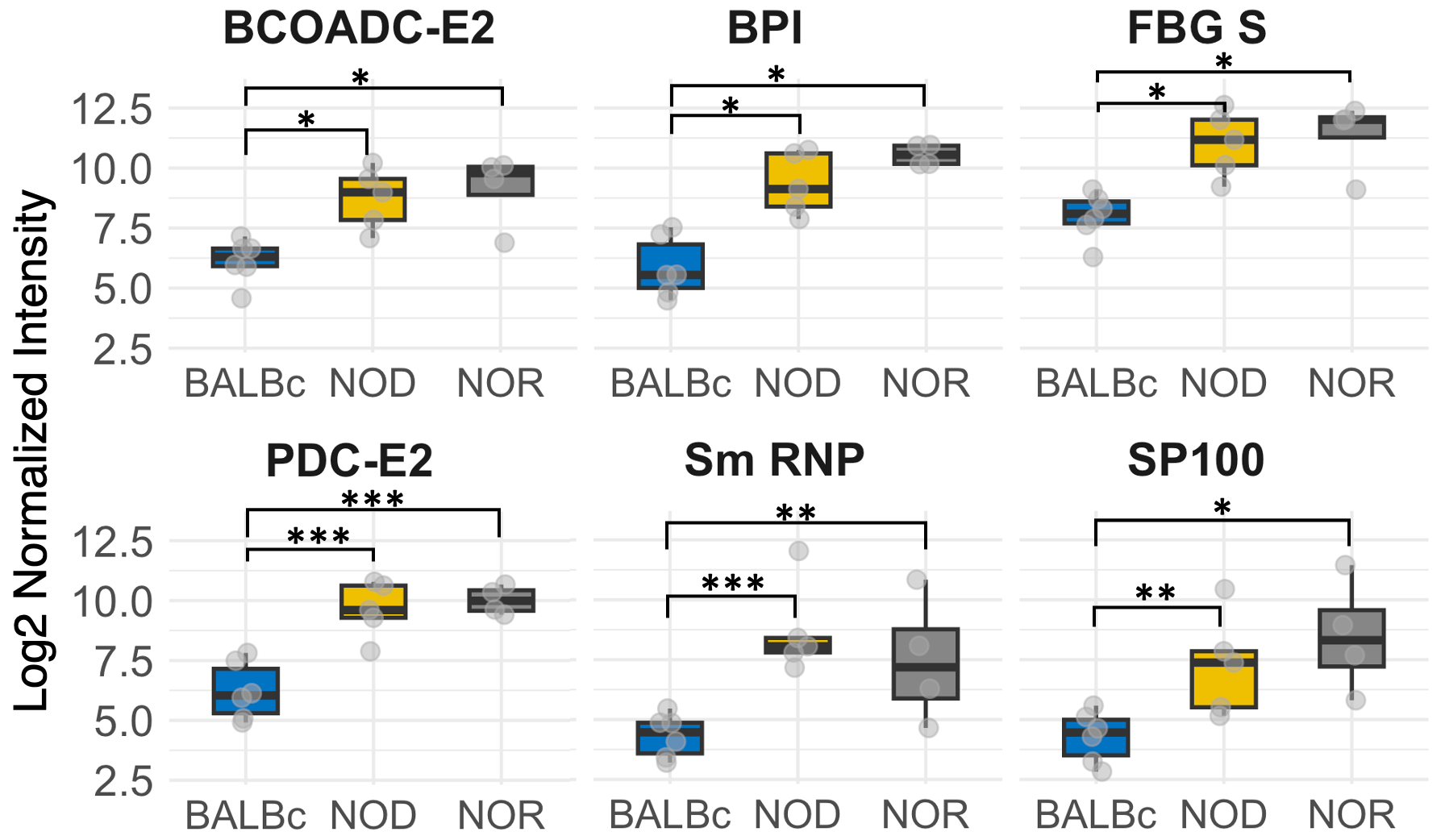 |
| --- |

**Supplemental Figure 3**. Additional significantly upregulated IgG autoantibodies from *Study 3* that were not assessed in the arrays from *Studies 1 and 2*. Each point represents one mouse with tears collected from male NOD mice (n=5) and male NOR mice (n=4) with intermediate disease versus age-matched BALB/c (n=6) mice. Moderated t-statistics estimated using Limma R package. ( **.** padj < 0.1, * padj < 0.05, ** padj < 0.01, *** padj < 0.001).

*BCOADC – Branched chain 2-oxo acid dehydrogenase complex; Bactericidal/permeability-increasing protein (BPI); FBG S – Fibrinogen Type I – S; PDC-E2 – Pyruvate dehydrogenase complex component E2; Sm RNP – Smith/Ribonuclear Protein Antibody (anti-ENA); SP 100 – Anti sp100 nuclear antigen*

| A. | *Study 3* – Total Serum IgG & IgA  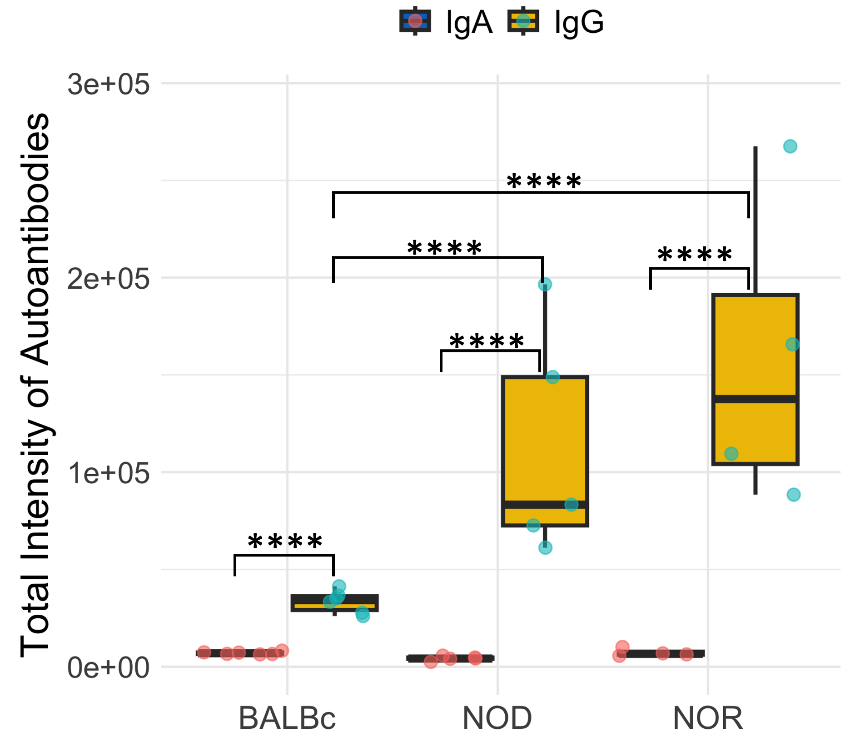 |
| --- | --- |
| **B.** | *Study 3* – Serum IgA  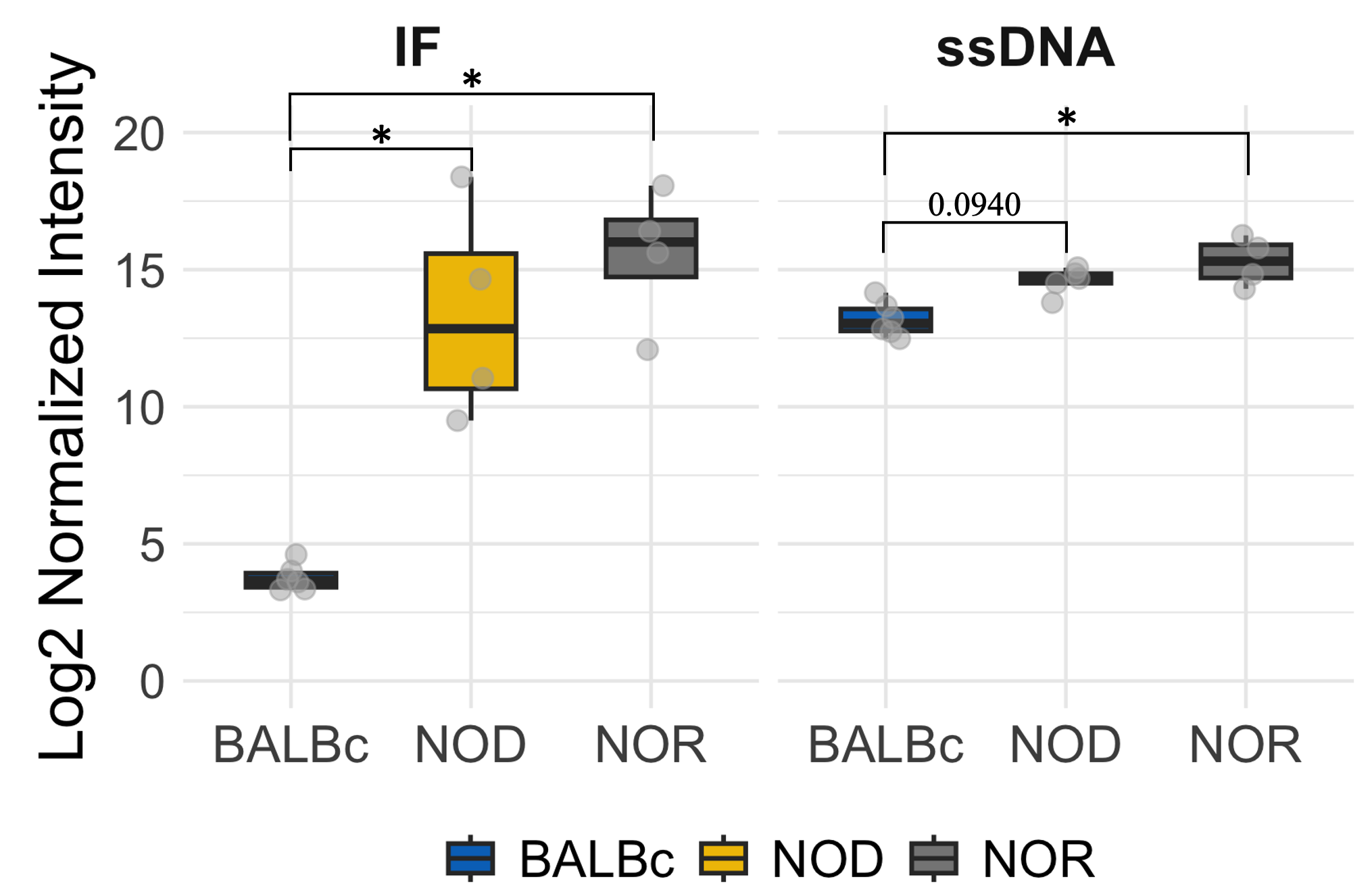 |
| **Supplemental Figure 4. Serum IgG and IgA levels per mouse strain from *Study 3.* (A)** Boxplots showing sum of raw signal intensity of Ig reactivity for 80 autoantibodies for isotypes IgA or IgG in mouse serum from *Study 3*. (**** p_adj_ < 0.0001, One-way ANOVA with Tukey’s Honest Significant Difference Test for multiple comparison correction). **(B)** Differentially expressed IgA autoantibodies in serum of adult male NOD, male NOR and BALB/c mice from *Study 3*. Each point represents one mouse, with serum and tears collected from male NOD mice (n=5) and male NOR (n=4) with intermediate disease versus age-matched male BALB/c (n=6) mice. Moderated t-statistics estimated using Limma R package. Adjusted p-values calculated using the Benjamin & Hochberg Procedure with alpha = 0.05 (* p < 0.05, ** p < 0.01, *** p < 0.001). *Abbreviations: IF – Intrinsic Factor; ssDNA – Single stranded DNA;* | |

| **A.** | *Study 2* – Tear & Serum IgG  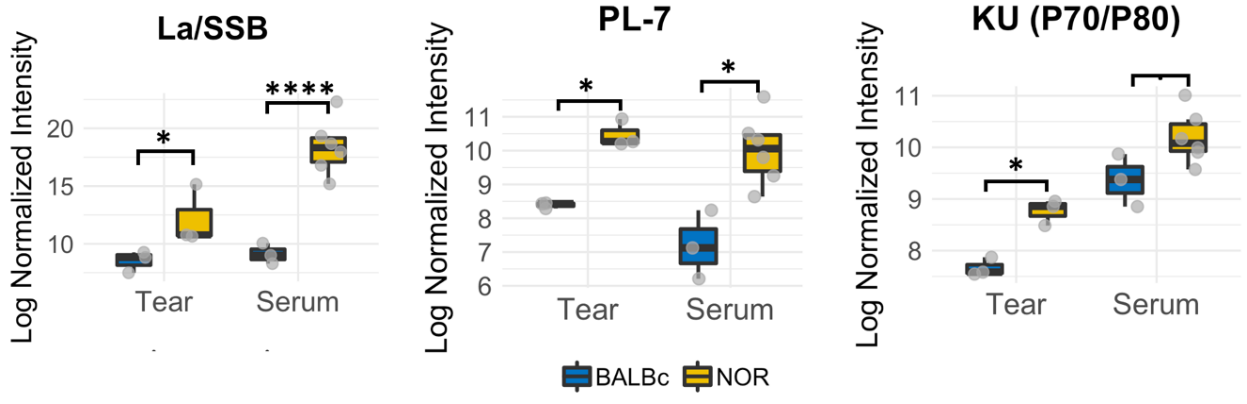 |
| --- | --- |
| **B.** | *Study 3* – Tear & Serum IgG  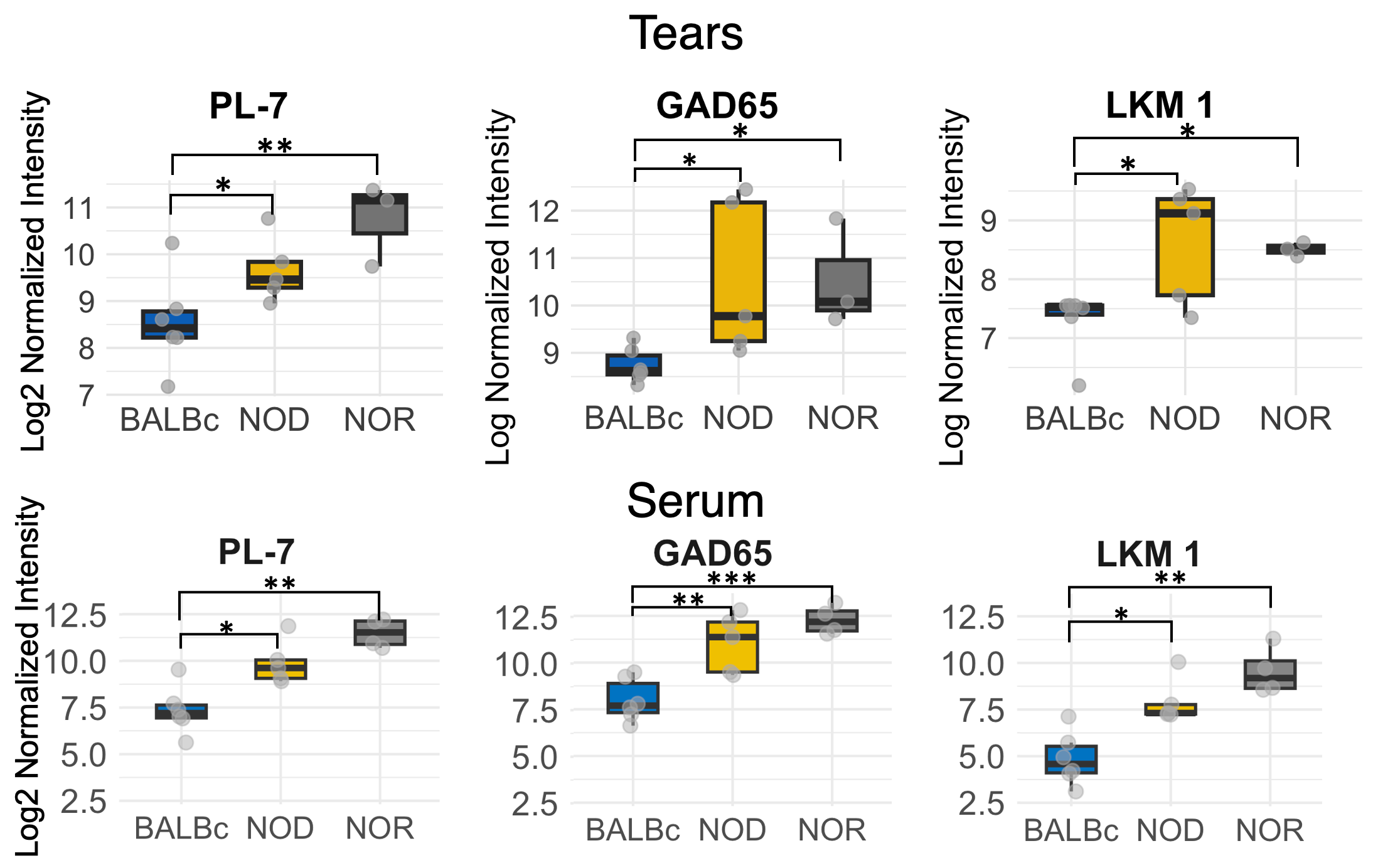 |
| **Supplemental Figure 5.** **IgG Autoantibodies common to serum & tears.** **(A)** IgG Autoantibodies upregulated in serum and tears of male NOR mice with intermediate disease relative to age-matched male BALB/c mice from *Study 2*. For serum collection, each point represents one mouse with serum collected from NOR (n=6) and BALB/c (n=3) mice. Tears were pooled from one, two or three NOR mice per sample (n=3 samples), with tears collected from one mouse per sample for BALB/c controls (n=3) **(B)** IgG Autoantibodies upregulated in tears and serum of male NOD and NOR mice from *Study 3.* Each point represents one mouse, with serum and tears collected from male NOD (n=5) and male NOR (n=4) mice with intermediate disease versus healthy male age-matched BALB/c (n=6) mice. Moderated t-statistics estimated using Limma R package. ( **.** padj < 0.1, * padj < 0.05, ** padj < 0.01, *** padj < 0.001).  *PL-7 – threonyl-tRNA synthetase; La; KU (P70/P80) – Heterodimer Ku protein subunits 70 and 80; GAD65 – Glutamic Acid Decarboxylase 65; LKM1 – Liver kidney microsome type 1;* | |

*Study 3* – Tear IgG

| **A** **Tears**  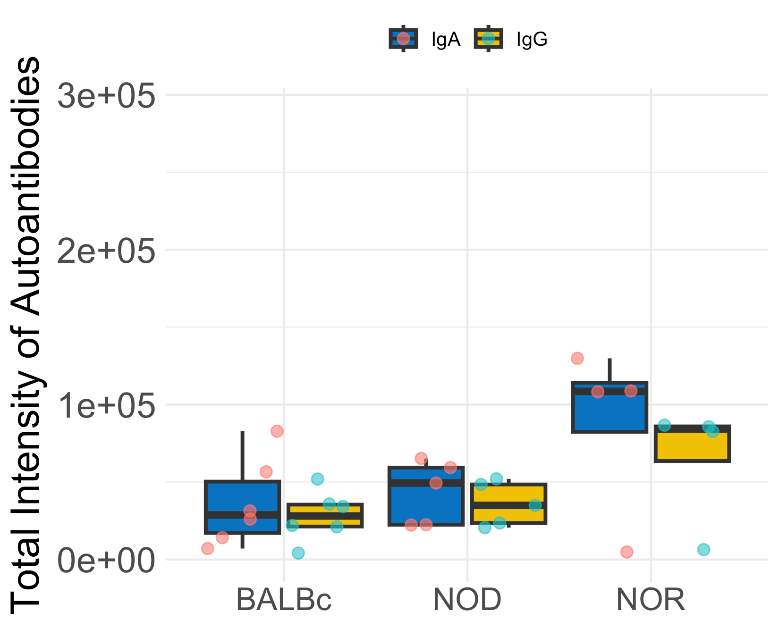 | **B** **Tears**  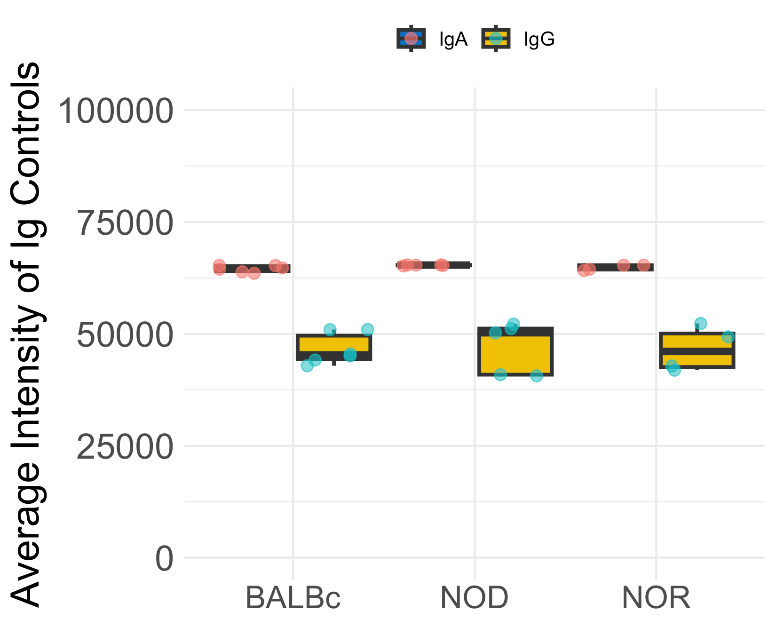 |
| --- | --- |
| **Supplemental Figure 6. IgG vs IgA signal intensity in mouse tears from *Study 3*. (A)** Boxplots showing sum of raw signal intensity of 80 autoantibodies for IgG and IgA in mouse tears. **(B)** Boxplots showing signal intensity of Ig reactivity towards mouse IgG or IgA control. The NOR and BALB/c mice with nearly 0 total signal intensity for both IgG and IgA in tears were removed from analysis. Each point represents one mouse, with serum and tears collected from male NOD (n=5) and male NOR (n=4) mice with intermediate disease versus healthy male BALB/c (n=6) mice. | |

*Study 3* – Tear IgG and IgA

| 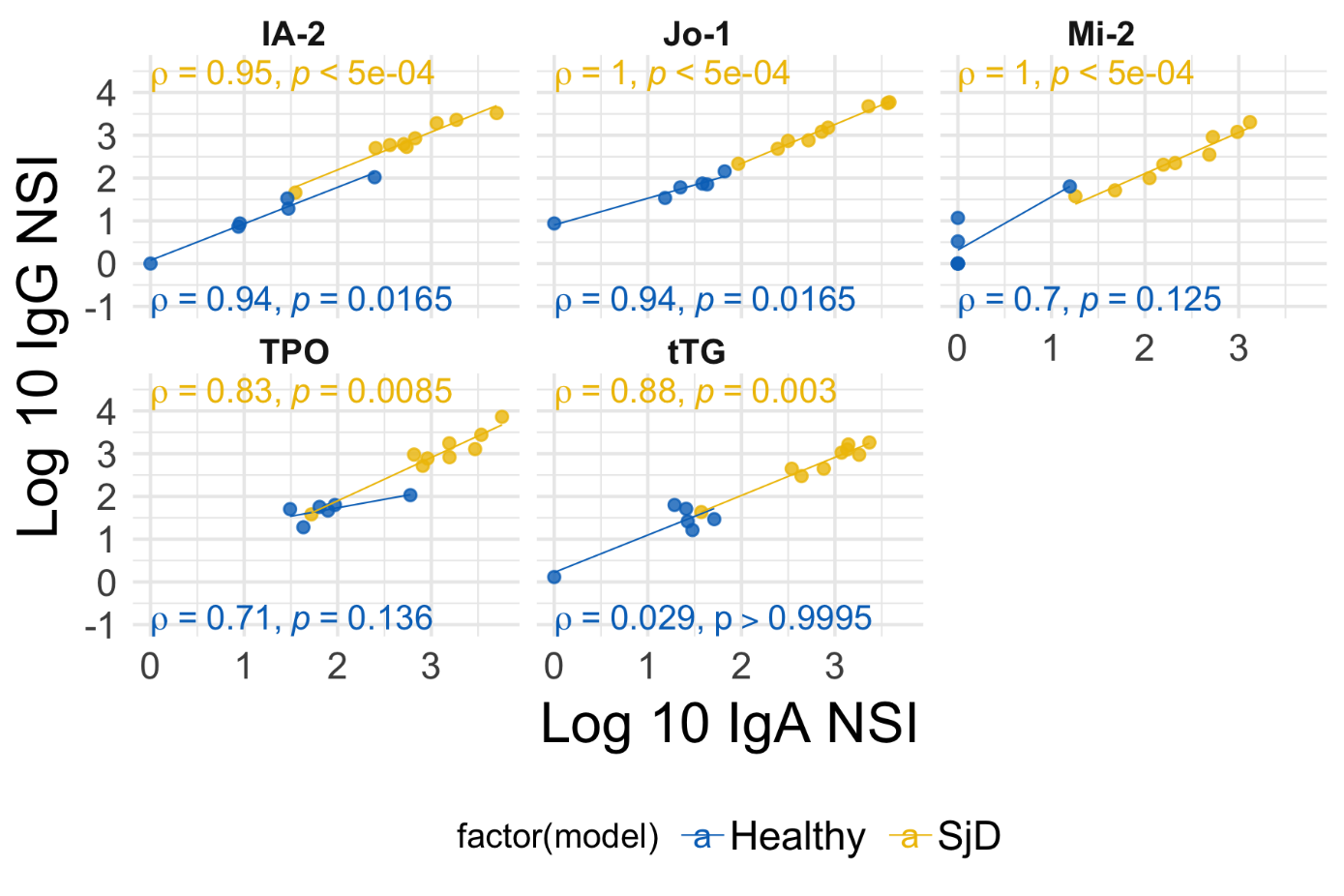 |
| --- |
| **Supplemental Figure 7. Correlation analysis of tear IgA vs IgG to the same autoantigen.** Scatterplots showing log transformed raw intensity values of autoantibodies to the same autoantigen with significantly high reactivity in both IgA and IgG in both male NOD and NOR mice with intermediate disease with respect to male BALB/c. Each point represents one mouse. The male NOD and NOR mice are shown in yellow and healthy control BALB/c is shown in blue. Spearman’s correlation coefficients for tears from SjD mice (yellow) and healthy mice (blue) with respective p-values are also shown. Only p < 0.01 is considered significant to account to correct for multiple correlation. No mice were excluded from analysis. |

| **A**  **B** | 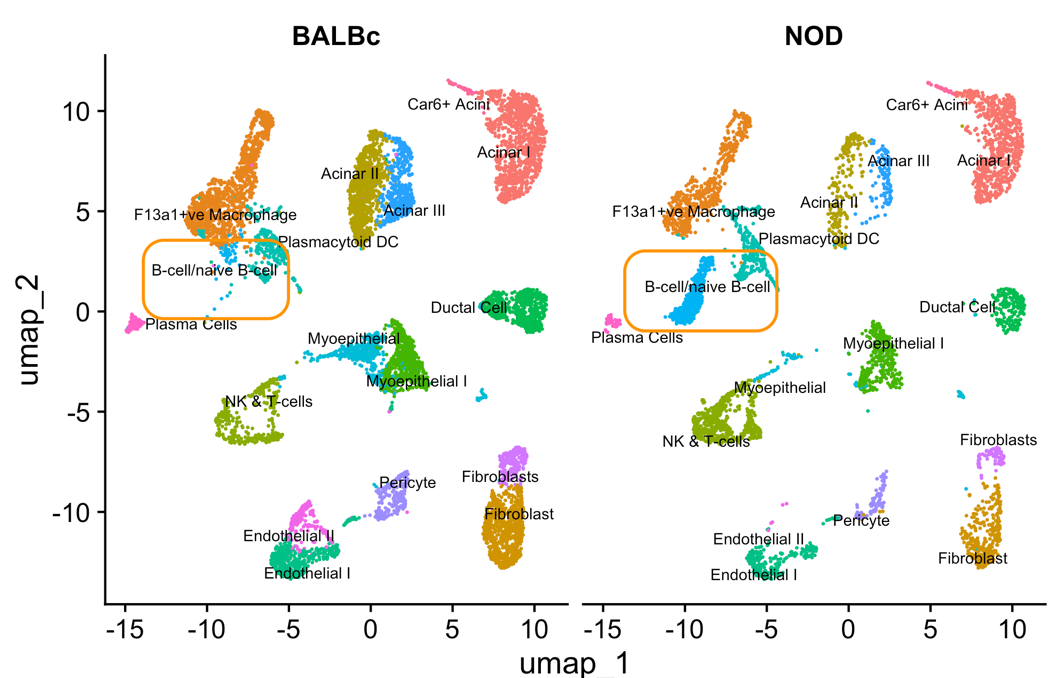 | | |
| --- | --- | --- | --- |
| 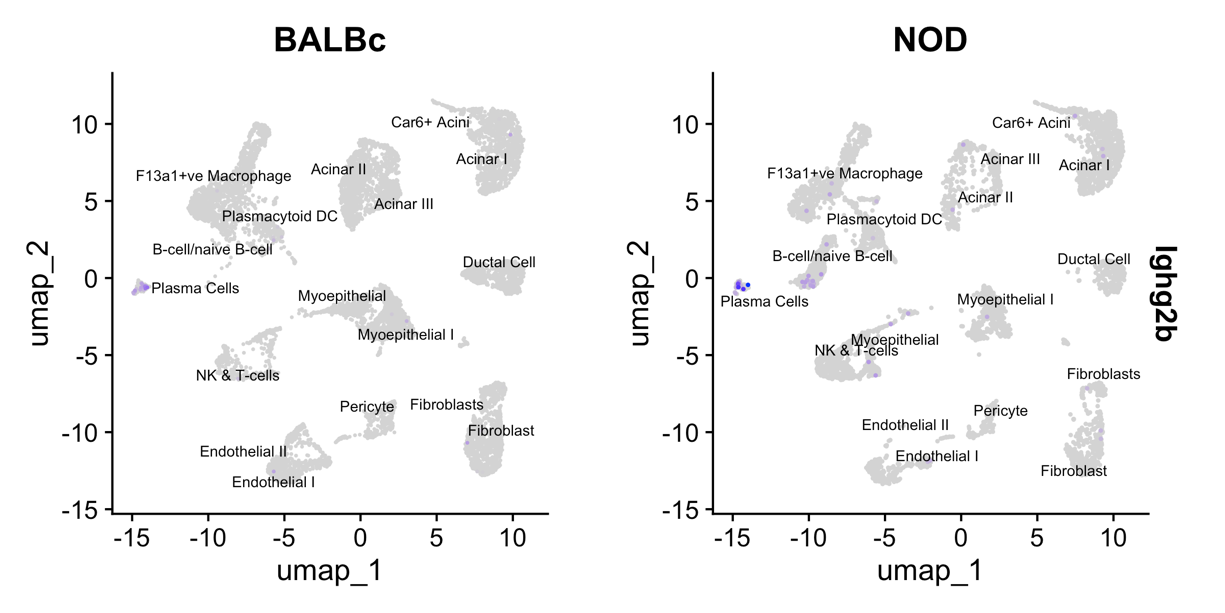 | | | |
| **C** | | | |
| 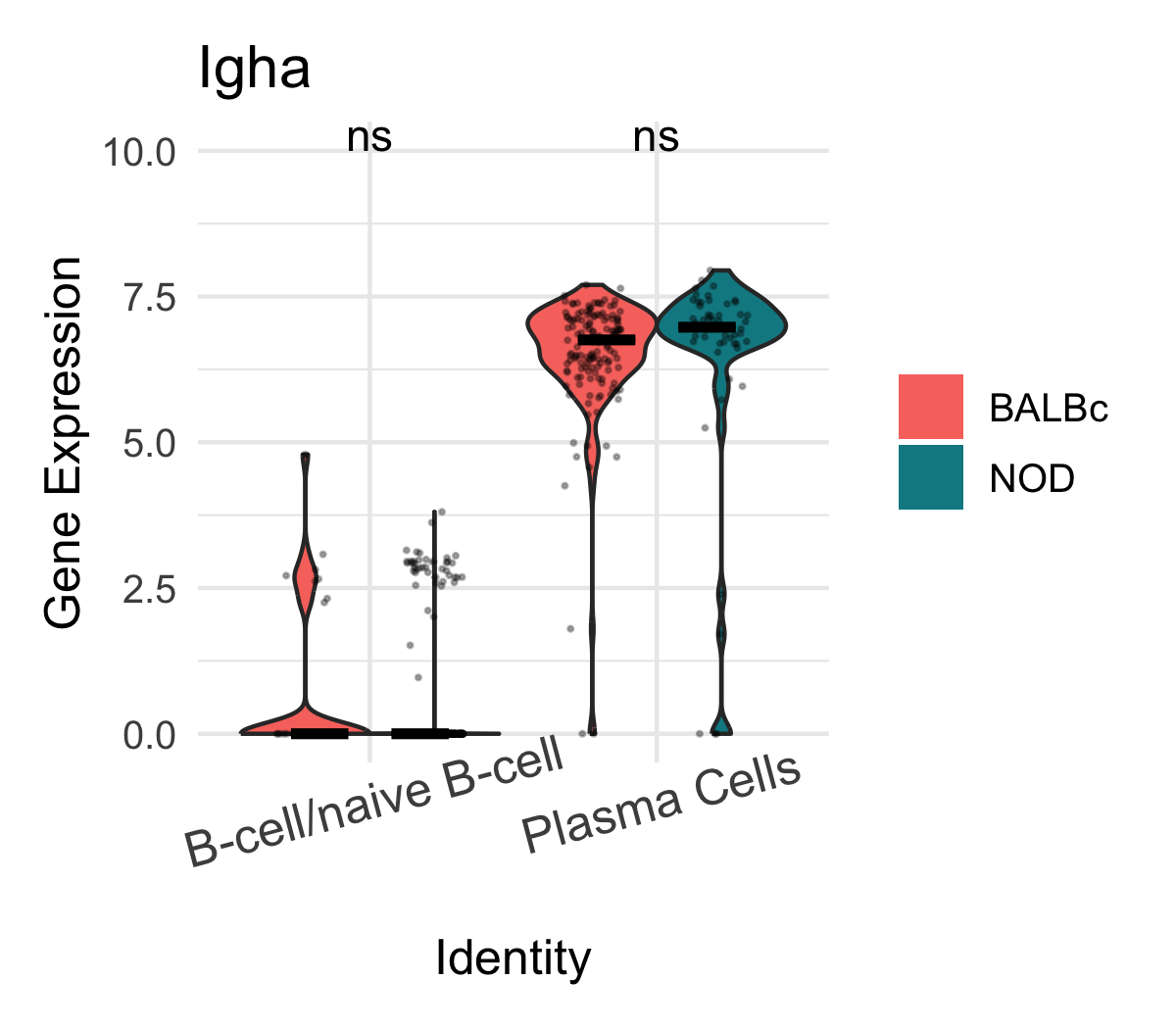 | | 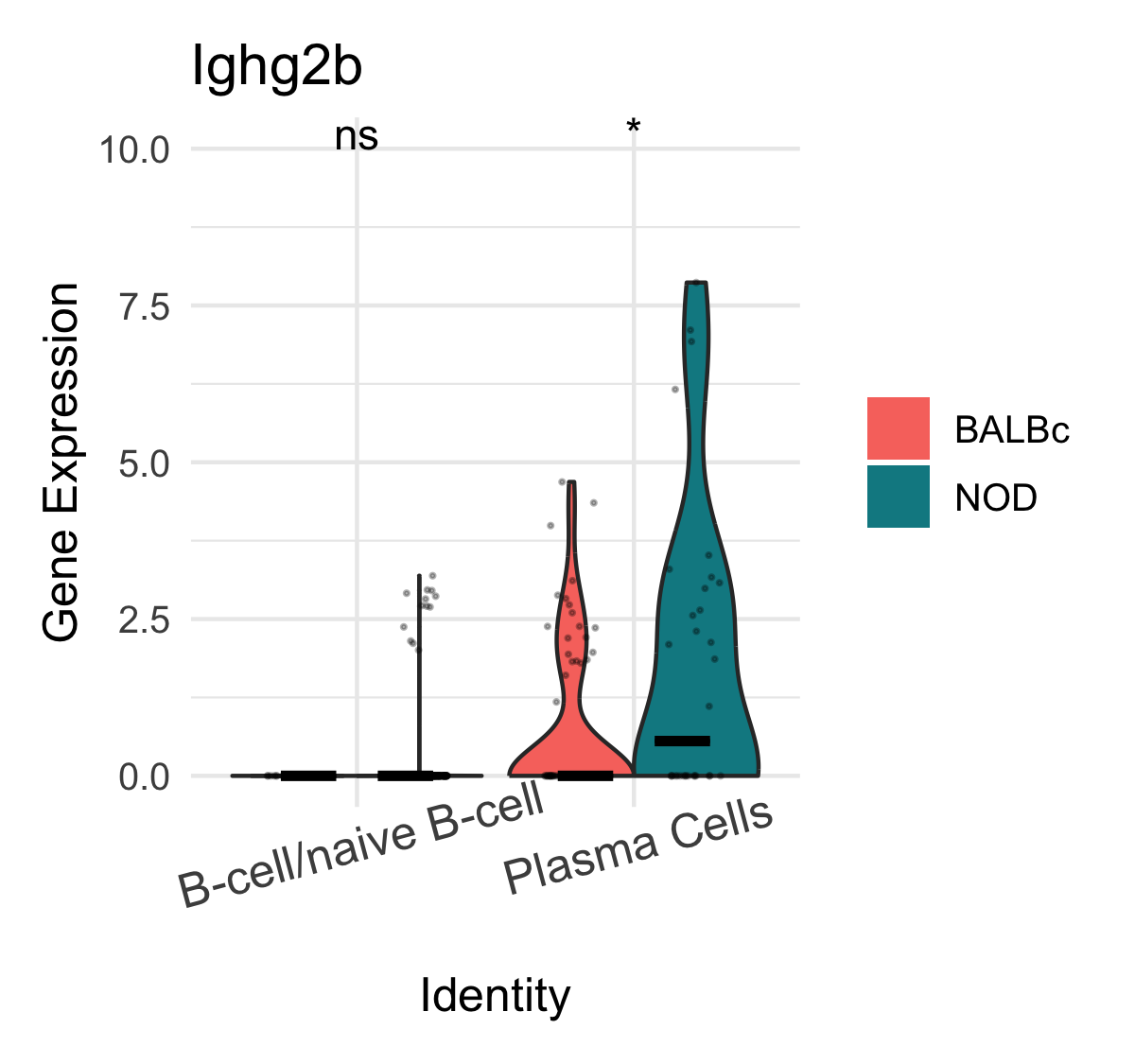 | 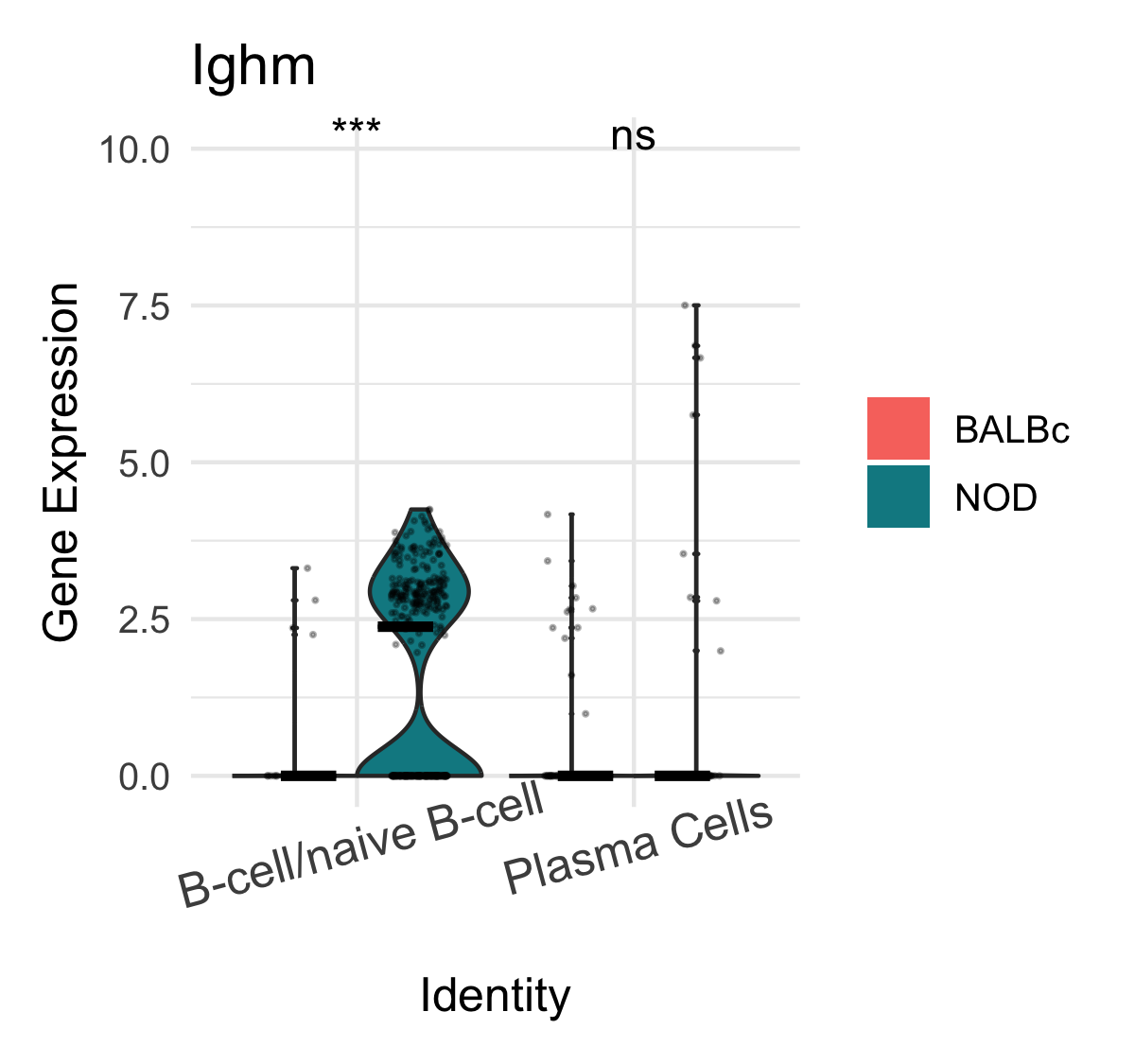 |
| **Supplemental Figure 8. Single Cell RNA sequencing analysis of 12 week old male NOD.H2b mice relative to age and sex matched BALB/c control mice.** (A) UMAP showing different clusters of single cells detected in mice LG. Higher numbers of B-cell/naïve B-cell were detected in LG of male NOD.H2b (boxed region). UMAP was generated from scRNA-Seq data sequenced from 8000 single cells lysed from LG of BALB/c control and 4414 single cells from LG of male NOD.H2b mice. Each dot represents one cell. (B) UMAP showing relative expression of mouse IgG producing gene Ighg2b being expressed uniquely in the ‘Plasma Cells’ and ‘B-cell’ clusters. Each dot represents one cell. (C) Violin plots showing relative expression of IgA, IgG and IgM genes in ‘Plasma Cells’ and ‘B-cell/naïve B-cell’ clusters. Each dot represents one cell. (***p<0.0001, **p<0.001, *p<0.01, One-way paired-ANOVA). Figures were created using Seurat from raw fastq data originally generated by PMID: 37812717. | | | |

**Supplemental Table 1. Differentially Expressed IgG Autoantibodies in Serum *of Study 1* (NOD vs BALB/c).**

|  | **NOD (N=3) vs BALB/c (N=3)**  Early Disease | | **NOD (N=3) vs BALB/c (N=3)**  Advanced Disease | | **Age** |
| --- | --- | --- | --- | --- | --- |
| **Auto Antigen** | **p_adj_** | **Log2 Fold-Change (Fold Change)** | **p_adj_** | **Log2 Fold-Change**  **(Fold Change)** | **p_adj_** |
| PL-7^†^ | **0.0209** | 6.38 (83.24) | 0.10 | 3.48 (11.17) | 0.94 |
| PM/Scl-100 | **2.54 x 10^-4^** | 5.82 (56.41) | **0.0011** | 4.50 (22.60) | 0.12 |
| La/SSB^†^ | **0.0256** | 4.19 (18.25) | 0.0913 | 2.98 (7.93) | 0.52 |
| LC1 | **0.0013** | 4.07 (16.78) | **0.0011** | 4.19 (18.25) | 0.52 |
| MAG-Fc | 0.0634 | 3.30 (9.82) | **0.0177** | 6.19 (487.92) | 0.63 |
| KU (P70/P80) | **0.0209** | 2.59 (6.02) | **0.0177** | 2.60 (6.07) | 0.87 |
| PCNA | **0.0471** | 2.47 (11.82) | **0.0105** | 3.79 (13.84) | 0.73 |
| Mitochondrial antigen | **0.0398** | 1.82 (3.53) | 0.2574 | 0.97 (1.95) | 0.73 |
| TPO | **0.0386** | 1.31 (2.48) | 0.1162 | 1.06 (2.89) | - |
| Intrinsic Factor | **0.0309** | 3.93 (15.30) | 0.44 | 1.19 (3.93) | - |
| CENP-B | 0.0686 | 1.37 (2.59) | - | (1.79) | **0.0196** |
| SRP54 ^>^ | **0.0262** | -0.72 (-1.64) | 0.03 | -0.70 (2.01) | 0.1827 |

*Mice with early disease were from 8-12 weeks while mice with advanced disease were >20 weeks as in Methods. BALB/c mice were age-matched to NOD mice*

*p_adj_ <0.05 was considered significant to account for multiple comparisons. Statistically significant p-values with fold change greater than 2 are shown in bold.*

^†^Also upregulated in tears of male NOD and NOR mice compared to healthy BALB/c

^>^IgG downregulated in tears of male NOD and NOR mice compared to healthy BALB/c

**Supplemental Table 2. Differentially Expressed IgG Autoantibodies in Serum of *Study 2* (NOR vs BALB/c).**

| **Auto Antigen** | **P adj** | **Log2 Fold-Change (Fold Change)** |
| --- | --- | --- |
| LC1 | **0.0123** | 10.42 (1372.16) |
| PM/Scl 100 | **0.0010** | 10.33 (1284.03) |
| Ribo Phosphoprotein P0 | **0.0131** | 9.37 (663.44) |
| Mito antigen | **0.0489** | 0.65 (1.57) |
| La/SSB | **0.0010** | 9.27 (616.03) |
| complement C3 | **0.0137** | 6.82 (112.79) |
| complement C4 | **0.0062** | 5.32 (40.01) |
| complement C5 | **0.0131** | 4.42 (21.48) |
| CRP | **0.0311** | 28.55 (3.92 x 10^6^) |
| >M2 | **0.0029** | 3.72 (13.18) |
| Sm/RNP | **0.0034** | 0.83 (1.78) |
| >SRP54 | **0.0131** | 0.57 (1.49) |
| TNF-α | **0.0311** | 5.20 (36.86) |
| Intrinsic Factor | **0.0159** | 2.82 (7.06) |
| PL-7 | 0.0799 | 6.34 (81.12) |
| PCNA | 0.1297 | 4.02 (16.25) |

*P adj <0.05 was considered significant to account for multiple comparisons.*

*Statistically significant p-values are shown in bold.*

^†^Also upregulated in tears of male NOD and NOR mice compared to healthy BALB/c

^>^ IgG downregulated in tears of male NOD and NOR mice compared to healthy BALB/c

**Supplemental Table 3. Differentially Expressed IgG and IgA Autoantibodies in Serum from *Study 3* (NOD, NOR vs BALB/c).**

|  | **NOD v BALB/c** | | | **NOR v BALB/c** | | |
| --- | --- | --- | --- | --- | --- | --- |
|  | **p_adj_** | **Log2 Fold Change (Fold Change)** | | **p_adj_** | **Log2 Fold Change**  **(Fold Change)** | |
| **Serum IgG only** | | | | | | |
| *LC1 – Anti-liver cytosolic antigen type 1* | **0.0050** | | 2.31 (4.95) | **2.59 x 10^-4^** | | 3.56 (11.76) |
| La SSB | **0.0020** | | 3.04 (8.24) | **0.0170** | | 2.17 (4.51) |
| PCNA – *Proliferating Cell Nuclear Antigen* | **0.0067** | | 2.52 (5.74) | **0.0029** | | 3.08 (8.43) |
| PDC-E2 *Pyruvate dehydrogenase complex component E2* | **0.0016** | | 2.8 (6.95) | **2.59 x 10^-4^** | | 3.37 (10.35) |
| BPI *Bactericidal/permeability-increasing protein* | **0.0021** | | 2.72 (6.57) | **1.54 x 10^-4^** | | 3.98 (15.83) |
| Sm RNP – Small nuclear ribonucleoprotein | **0.0045** | | 4.34 (20.25) | **0.0130** | | 3.73 (13.29) |
| FBG IV – Fibrinogen IV | **0.0042** | | 2.67 (6.38) | **0.0199** | | 2.09 (4.26) |
| FBG S – Fibrinogen S | **0.0067** | | 2.49 (5.62) | **0.0029** | | 3.02 (8.1) |
| SP100 *– Anti sp100 nuclear antigen* | **0.0081** | | 3.02 (8.1) | **0.0029** | | 3.85 (14.37) |
| BCOADC-E2 | **0.0276** | | 1.96 (3.89) | **0.0058** | | 2.7 (6.49) |
| **Serum & Tear IgG** |  | |  |  | |  |
| PL-7 *– threonyl-tRNA synthetase* | **0.0067** | | 2.29 (4.88) | **2.59 x 10^-4^** | | 3.65 (12.52) |
| GAD65 *Glutamic Acid Decarboxylase 65* | **0.0021** | | 2.6 (6.08) | **1.54 x 10^-4^** | | 3.78 (13.78) |
| LKM 1 *Liver kidney microsome type 1* | **0.0081** | | 2.31 (4.98) | **2.59 x 10^-4^** | | 3.81 (14.06) |
| **Serum IgA only** | | | | | | |
| IF *– Intrinsic Factor* | **0.0356** | | 11.09 (2187) | **0.0326** | | 11.82 (3618) |
| ssDNA | 0.0940 | | 1.29 (2.44) | **0.0180** | | 2.06 (4.17) |
| Cytochrome C | **0.0063** | | 6.03 (65.37) | 0.1710 | | 3.48 (11.17) |
| GP2 | 0.0940 | | 2.57 (5.95) | 0.1220 | | 2.43 (5.40) |
| FBG IV | 0.0940 | | 3.23 (9.38) | 0.1220 | | 3.23 (9.38) |

*p_adj_ <0.05 was considered significant to account for multiple comparisons. Statistically significant p-values are shown in bold.*
