# Supplementary material for: Serum and tear autoantibodies from NOD and NOR mice as potential diagnostic indicators of local and systemic inflammation in Sjögren’s disease
Source: Front Immunol. 2025 Jan 28;15:1516330. doi: 10.3389/fimmu.2024.1516330 (PMC11810956; doi:10.3389/fimmu.2024.1516330)
Supplement: Supplementary file 2 [file DataSheet2.pdf]

Supplemental Methods 1.1

Serum IgG Autoantibody 2021

ShrutiSinghKakan

2024-12-09

###0 Dataset ######

1. Visualizing raw data

```
#Boxplot of raw Signal Distribution, not normalized

mydata<-as.matrix(log2(data+0.5))

boxplot(as.data.frame(mydata),main="Signal distribution, Not normalized",xlab="Samples",
ylab="Log2 Intensity",cex=0.8)
```

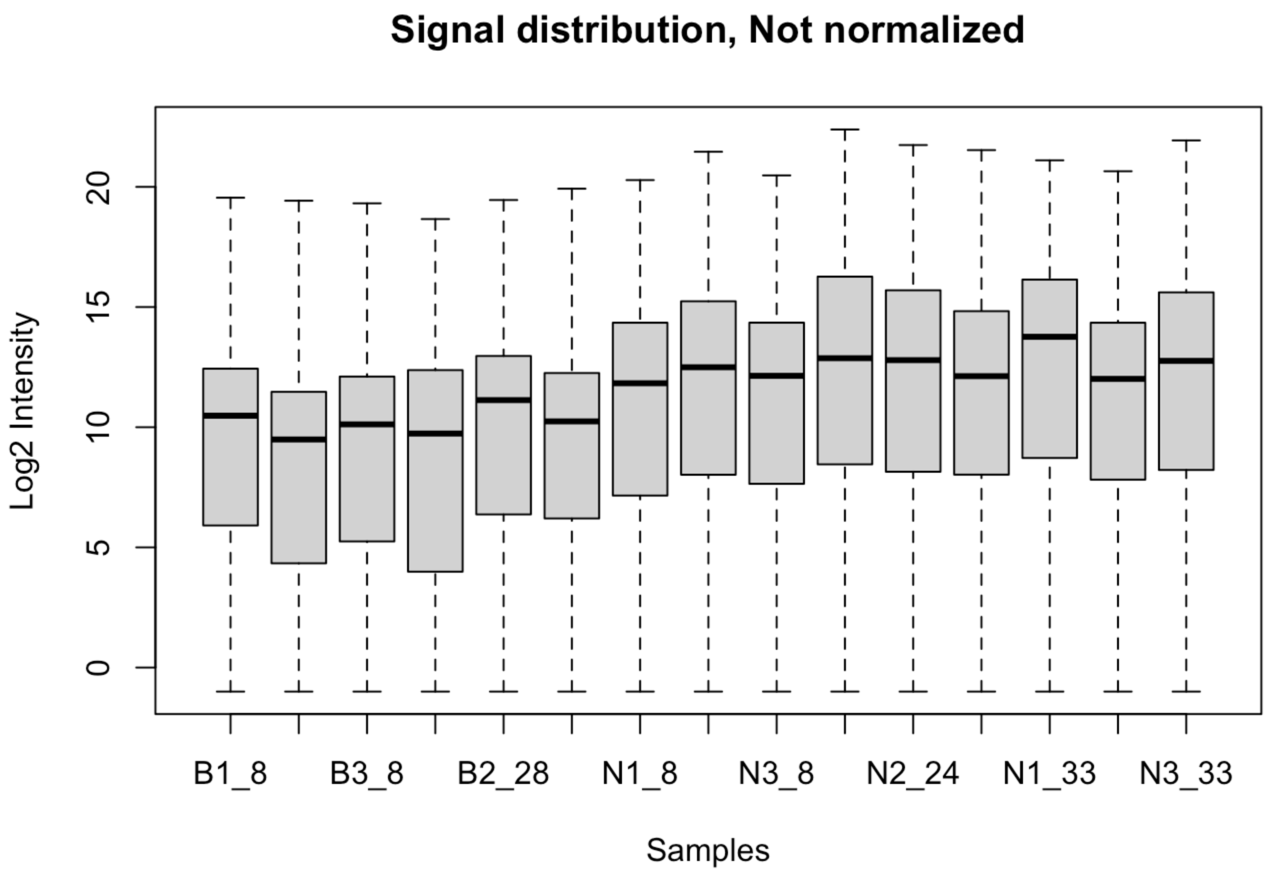

#####2. Clustering samples based on Pearson correlation ############

#####3. Data filtering and Normalization ########

####4. DGE analysis ##########

```
library(devtools)
install_github("dpgaile/AutoAntArrayExmpl")
#library(AutoAntArrayExmpl)

library(devtools)
#install_github("dpgaile/AutoAntArrayExmpl")
library(AutoAntArrayExmpl)
library(NMF)
library(quantreg)
library(asbio)
library(fdrtool)
#library(discreteMTP)
```

Reading in Raw Data

```
knitr::opts_chunk$set(
    echo = TRUE,
    message = FALSE,
    warning = FALSE,
    root.dir = '~/Documents/3_Parkinsons_disease/Autoantibody_Data/'
)

IgG_NSI <- read.csv("~/Documents/3_Parkinsons_disease/Autoantibody_Data/SerumAntibodies_
2021/IgG_MCF_NSI.csv",  header=T, row.names = 1)[,1:12]
IgG_SNR <- read.csv("~/Documents/3_Parkinsons_disease/Autoantibody_Data/SerumAntibodies_
2021/IgG_MCF_SNR.csv",  header=T, row.names = 1)[,1:12]
IgG_SNR$average <- rowMeans(IgG_SNR) #adding columns for average SNR for each row (autoa
ntibody)
IgG_SNR$med <- rowMedians(as.matrix(IgG_SNR)) #adding column for median SNR for each row
IgG_NSI <- IgG_NSI[which(IgG_SNR$average>3),]
IgG_SNR <- IgG_SNR[which(IgG_SNR$average>3),]
IgG_raw=list()
IgG_raw$NSI <- as.matrix(IgG_NSI)
IgG_raw$SNR <- as.matrix(IgG_SNR[,1:12])

colData0$Strain <- relevel(colData0$Strain, ref = "BALBc")
colData0$Age <- factor(colData0$Age, levels = c("8wks", "24wks","28wks"))#, "33wks"))
colData0$Age <- relevel(colData0$Age, ref="8wks")
IgG_raw$SInfo <- colData0

#Defining colors and point shapes
clrs=c(rep(pal_jco("default")(5), each=3))[1:12]
pchs=c(rep(3,3),rep(4,3), rep(5,3), rep(6,3), rep(7,3))[1:12]
```

Sample Spot and Background Distributions

```
#Centering and Scaling Differences Across Features
# Tukey Tri-mean for location
TriMnG=RowTriMeans(IgG_raw$NSI)
# Biweight midvariance for spread
bw.estG=unlist(apply(IgG_raw$NSI,1,r.bw))
```

Normalization First Stage

```
## Normalization
### First Stage

#enable to background correct
IgG_raw$NSI <- as.matrix(IgG_NSI[,1:12]) # Excluding the Nod 33 week sample
IgG_raw$SNR <- as.matrix(IgG_SNR[,1:12])
IgG_raw$NSI=IgG_raw$NSI * IgG_raw$SNR ##NSI changed permanently
IgG_raw$X_norm_1=IgG_raw$NSI
eps=.10
IgG_raw$QregFits=array(NA,dim=c(12,70))
TriMn=RowTriMeans(IgG_raw$X_norm_1)
ResMat=IgG_raw$X_norm_1-matrix(rep(TriMn,12),ncol=12)
SubMat=abs(ResMat)<quantile(as.vector(abs(ResMat)),prob=1-eps)

par(mfrow=c(2,2));par(mgp=c(1.5,.5,0));par(mar=c(2.75,2.75,1.5,0.25))
plot(rep(TriMn,12),as.vector(ResMat),
     xlim=quantile(TriMn,prob=c(0,.8)),
     ylim=quantile(as.vector(ResMat),prob=c(0.05,.95)),
     type="n",xlab="Raw Signal Tri-Mean",ylab="Raw Signal",
     main="IgG Raw Signal")
for(j in 1:12){
   points(TriMn,ResMat[,j],cex=0.5,pch=pchs[j],col=clrs[j])
   y=ResMat[,j]
   x=TriMn
   fitj <- rq(y ~ x, tau = .5,subset=which(SubMat[,j]))
   abline(coef(fitj),col=clrs[j])
   IgG_raw$QregFits[j,]=coef(fitj)
   prdct=coef(fitj)[1]+coef(fitj)[2]*TriMn
   IgG_raw$X_norm_1[,j]=IgG_raw$X_norm_1[,j]-prdct
} ###substracting the model fit straight line from the data
#Xnorm_1 here is the residual value

eps=0.10
q.eps=0.10
lambda=.10 # incremental change
nitr=99 # number of iterations -> this should help us optimize the lambda for best fit
sad=rep(0,nitr) # sum of absolute deviations
# re-init
IgG_raw$X_1step_norm_1=IgG_raw$X_norm_1 #calculated in the last loop
IgG_raw$X_norm_1=IgG_raw$NSI #reinitializing

for(i in 1:nitr){
   # snoop and grab invariants
   X=IgG_raw$X_norm_1
   iWRS=function(i) t.test(X[i,1:6],X[i,7:12])$p.value
   qMat=matrix(rep(mapply(iWRS,1:70),12),ncol=12)
   qMat[which(is.na(qMat[,1])==T),] <- 0 #all NANs replaced by 0
   qcut=quantile(qMat[,1],prob=q.eps)
   TriMn=RowTriMeans(IgG_raw$X_norm_1)
   ResMat=IgG_raw$X_norm_1-matrix(rep(TriMn,12),ncol=12)
```

```
    SubMat=abs(ResMat)<quantile(as.vector(abs(ResMat)),prob=1-eps) #all residuals with ab
solute values less than the cutoff of 90%
    SubMat[qMat<qcut]=FALSE #which p values are greater than the cutoffs
    for(j in 1:12){
      y=ResMat[,j]
      x=TriMn
      fitj <- rq(y ~ x, tau = .5,subset=which(SubMat[,j]))
      prdct=coef(fitj)[1]+coef(fitj)[2]*TriMn
      IgG_raw$X_norm_1[,j]=IgG_raw$X_norm_1[,j]-lambda*prdct
      sad[i]=sad[i]+sum(abs(2*lambda*prdct))
    }###substracting the model fit straight line from the data
}
```

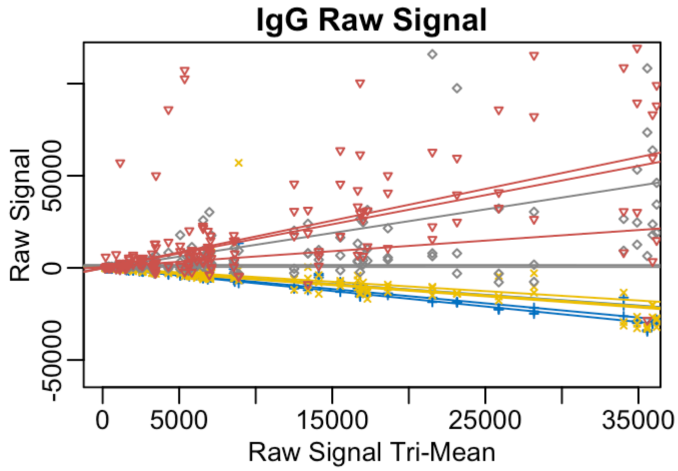

Second Stage of Normalization

```
### Second Stage ########
deltaG=min(as.vector(IgG_raw$X_norm_1))
IgG_raw$W_1=log(IgG_raw$X_norm_1-deltaG+1)
# get resids..
W_1=IgG_raw$W_1
RW=RowTriMeans(W_1)
# get residual matrices
RW_MAT=matrix(rep(RW,dim(W_1)[2]),ncol=dim(W_1)[2])
W_1=W_1-RW_MAT

W=W_1
bw.est=unlist(apply(W,2,r.bw))
# now, rescale
for(j in 1:12){
   W_1[,j]=W_1[,j]/sqrt(bw.est[j])
}

# now, get new values..
IgG_raw$W_1=RW_MAT+W_1
```

Formal Comparison of NOD and BALB/c AutoAntigen Expresion Profiles

```
mydata <- as.matrix(IgG_raw$W_1[,1:12])
#mydata <- as.matrix(IgG_raw$X_nrm_1)
colData <- colData0[1:12,] #removing male NOD 33 weeks sample
colData$Strain <- relevel(colData$Strain, ref = "BALBc")
colData$Age <- factor(colData$Age)

conditions<- paste(colData$Strain, colData$Age,sep=".")
conditions <- factor(conditions, levels=unique(conditions))
design <- model.matrix(~0+ conditions)
rownames(design) <- colnames(mydata)
colnames(design) <- levels(conditions)
fit <- lmFit(mydata, design)
summary(fit)
```

```
##                   Length Class  Mode
## coefficients     280    -none- numeric
## rank               1    -none- numeric
## assign             4    -none- numeric
## qr                 5    qr     list
## df.residual       70    -none- numeric
## sigma             70    -none- numeric
## cov.coefficients  16    -none- numeric
## stdev.unscaled   280    -none- numeric
## pivot              4    -none- numeric
## Amean             70    -none- numeric
## method             1    -none- character
## design            48    -none- numeric
```

```
cont.matrix<- makeContrasts(
   B8vsN8 = NOD.8wks - BALBc.8wks,
   B28vB8 = BALBc.28wks - BALBc.8wks,
   B28vN24 = NOD.24wks - BALBc.28wks,
   N24vN8 = NOD.24wks - NOD.8wks,
   Age = (NOD.24wks + BALBc.28wks)- (NOD.8wks+BALBc.8wks),
   levels = design)

fit.cont<- contrasts.fit(fit, cont.matrix)
fit.cont<- eBayes(fit.cont)
qqt(fit.cont$t,df=fit.cont$df.prior+fit.cont$df.residual,pch=16,cex=0.5)
abline(0,2)
```

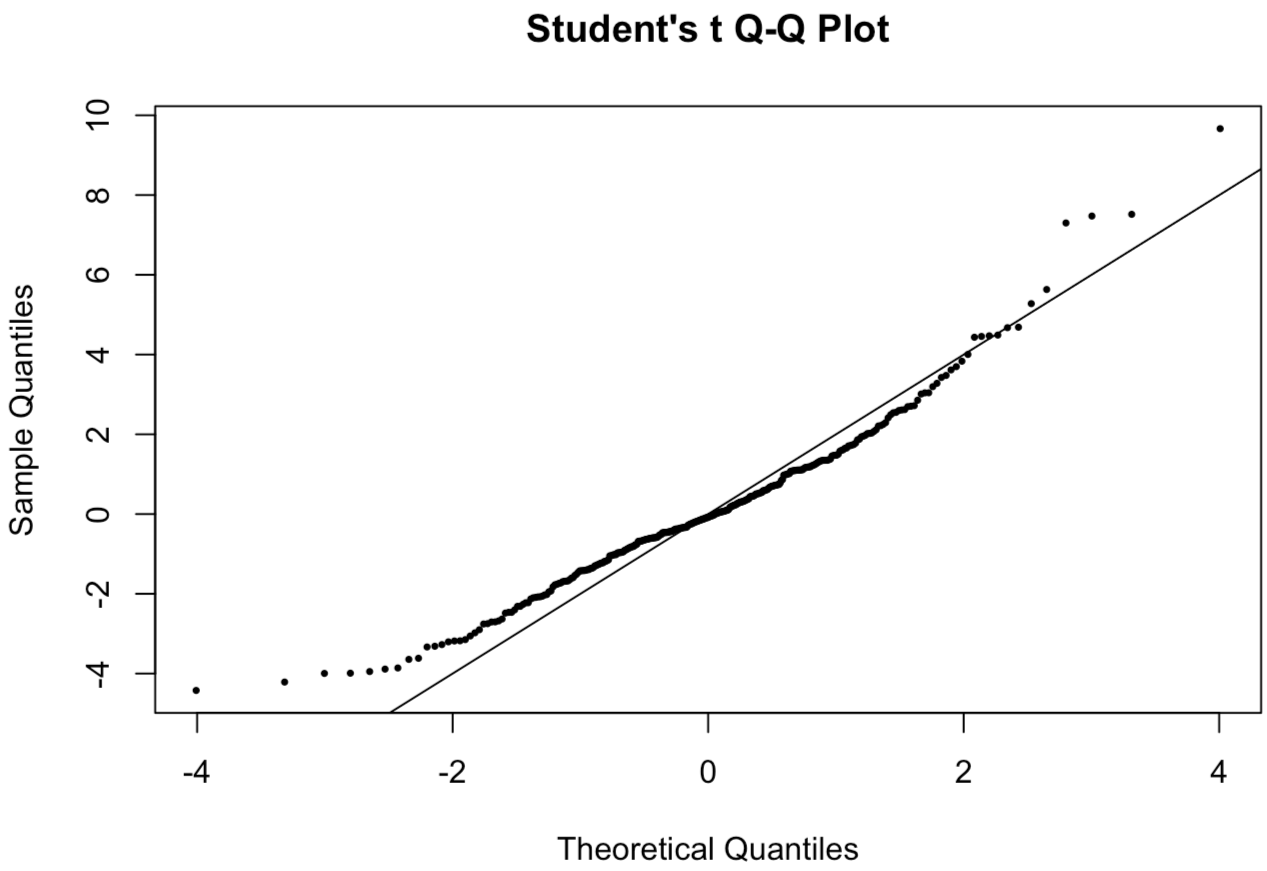


```
# NOD.8wks - BALBc.8wks
table1 <- topTable(fit.cont, adjust="BH", coef=1, number=35)
table1 <- table1[which(table1$logFC>0.5),]
knitr::kable(table1)
```

	logFC	AveExpr	t	P.Value	adj.P.Val	B
PM_Scl-100	5.817888	10.84645	9.664695	0.0000036	0.0002548	5.0028772
LC1	4.068983	10.47944	7.299426	0.0000377	0.0013195	2.6628170
PL-7	6.379304	11.00174	4.674082	0.0010538	0.0209833	-0.7615804
Myosin	4.576711	10.61942	4.470734	0.0014192	0.0209833	-1.0685694
KU (P70_P80)	2.589127	10.39385	4.433881	0.0014988	0.0209833	-1.1248270
La_SSB	4.190159	10.76352	4.003026	0.0028778	0.0256565	-1.7959536
Jo-1	5.056152	11.01126	3.833758	0.0037444	0.0262106	-2.0658661
Intrinsic Factor	3.935539	11.01001	3.614781	0.0052922	0.0308710	-2.4195328
TPO	1.313070	10.67730	3.425379	0.0071711	0.0386136	-2.7288673
Mitochondrial antige	1.821790	10.41210	3.278836	0.0090953	0.0397920	-2.9698914
PCNA	2.184028	10.41969	3.039597	0.0134639	0.0470942	-3.3653470

	logFC	AveExpr	t	P.Value	adj.P.Val	B
Thyroglobulin	3.741136	11.28488	3.010405	0.0141282	0.0470942	-3.4136830
TTG	3.293814	10.65669	2.717645	0.0229608	0.0634490	-3.8977305
MAG-FC	3.296065	11.60066	2.693813	0.0238901	0.0634490	-3.9369850
CENP-B	1.373516	10.66595	2.610708	0.0274373	0.0685932	-4.0735506
Beta 2-glycoprotein	1.164122	10.47419	2.248119	0.0501496	0.1170158	-4.6600521
Ig control	1.429525	11.75261	1.779554	0.1076597	0.2216406	-5.3754558

```
#R-Squared ....... goodness of fit
for (i in 1:70){
   sst <- rowSums(mydata^2)
   ssr <- sst - fit.cont$df.residual*(fit.cont$sigma^2)
   Rsq<- (ssr/sst)
}
print(Rsq)
```

```
##    Beta 2-glycoprotein                    BPI                    C1q
##              0.9974214              0.9967703              0.9893650
##                 CENP-B              Chromatin          Collagen IV
##              0.9974523              0.9992820              0.9991340
##            Collagen V            Collagen VI                  dsDNA
##              0.9992981              0.9563098              0.9835177
##                Elastin         Entaktin EDTA          Fibrinogen IV
##              0.9995576              0.9984009              0.9953537
##            Fibrinogen S            Fibronectin            Gliadin_IgG
##              0.9938828              0.9873169              0.9369789
##        Glycated Albumin                    GP2                   HSPG
##              0.9961969              0.9989907              0.9995944
##             Histone H3             Histone H4          Histone-total
##              0.9996499              0.9994917              0.9993119
##             Histone H1            Histone H2A            Histone H2B
##              0.9927950              0.9990940              0.9765595
##       Intrinsic Factor                   Jo-1          KU (P70_P80)
##              0.9891458              0.9842268              0.9966483
##                 La_SSB                Laminin                    LC1
##              0.9896749              0.9977862              0.9970874
##                     M2                 MAG-FC               Matrigel
##              0.9933836              0.9879291              0.9995119
##                    MBP                   Mi-2   Mitochondrial antige
##              0.9997809              0.9996474              0.9969686
##                    MPO                 Myosin              Nucleolin
##              0.9938882              0.9897827              0.9998120
##      Nucleosome antigen                  PCNA          Peroxiredoxin 1
##              0.9949244              0.9948615              0.9734480
##                   PL-7                  PL-12             PM_Scl-100
##              0.9834950              0.9995156              0.9968603
##                    PR3    Prothrombin protien Ribo phospho protein P1
##              0.9995447              0.9956277              0.9960267
## Ribo phospho protein P2 Ribo phospho protein P0           Ro_SSA_52KDa
##              0.9995372              0.9975367              0.9812335
##           Ro_SSA_60KDa                 Scl-70                     Sm
##              0.9992127              0.9995754              0.9982708
##                 Sm_RNP                    SmD                  SRP54
##              0.9950084              0.9971982              0.9998867
##                  ssDNA           Thyroglobulin        Topoisomerase I
##              0.9463729              0.9865792              0.9995306
##                    TPO                    TTG             U1-snRNP-68
##              0.9987531              0.9856119              0.9860814
##             U1-snRNP-A           U1-snRNP-BB'             U1-snRNP-C
##              0.2258075              0.9129221              0.9963701
##               Vimentin            Vitronectin                Anti-Ig
##              0.9995588              0.9906819              0.9969986
##             Ig control
##              0.9948211
```

```
#summary(lm.first)$r.squared
```

```
# NOD.24wks - BALBc.28wks
table2 <- topTable(fit.cont,number=35,adjust="BH", coef=3)
table2 <- table2[which(table2$logFC>0.5),]
knitr::kable(table2)
```

	logFC	AveExpr	t	P.Value	adj.P.Val	B
LC1	4.1903065	10.47944	7.517071	0.0000297	0.0010909	2.9043777
PM_Scl-100	4.4986816	10.84645	7.473225	0.0000312	0.0010909	2.8561843
PCNA	3.7914660	10.41969	5.276731	0.0004518	0.0105414	0.1128577
MAG-FC	5.7339871	11.60066	4.686281	0.0010354	0.0177568	-0.7433518
KU (P70_P80)	2.6014592	10.39385	4.455000	0.0014526	0.0177568	-1.0925652
Scl-70	0.9259089	10.23085	3.696402	0.0046487	0.0392775	-2.2871617
Ribo phospho protein P0	1.5574574	10.57755	3.040998	0.0134328	0.0783581	-3.3630278
La_SSB	2.9886562	10.76352	2.855183	0.0182681	0.0913403	-3.6706206
Fibrinogen S	2.0885718	10.36405	2.704410	0.0234723	0.1091160	-3.9195349
Ig control	2.1041195	11.75261	2.619329	0.0270460	0.1091160	-4.0594097
ssDNA	7.3249179	12.32737	2.597274	0.0280584	0.1091160	-4.0955735
PL-7	3.4813173	11.00174	2.550743	0.0303206	0.1114954	-4.1717212
TPO	0.9246239	10.67730	2.412048	0.0381995	0.1162594	-4.3972297
Ro_SSA_52KDa	2.7125929	10.74977	1.938562	0.0833524	0.1944890	-5.1401687
Mitochondrial antige	0.9687554	10.41210	1.743555	0.1140071	0.2574353	-5.4273999
TTG	2.0060465	10.65669	1.655140	0.1310759	0.2808947	-5.5526590
Ribo phospho protein P1	1.0161021	10.18396	1.648621	0.1324218	0.2808947	-5.5617571
Thyroglobulin	2.0037330	11.28488	1.612357	0.1401389	0.2863052	-5.6120112
CENP-B	0.8410793	10.66595	1.598680	0.1431526	0.2863052	-5.6308023

```
#Effect of Age
knitr::kable(topTable(fit.cont,number=35,adjust="BH", coef=5))
```

	logFC	AveExpr	t	P.Value	adj.P.Val	B
CENP-B	4.1903247	10.665952	5.6319320	0.0002810	0.0196674	0.7705583
PM_Scl-100	-3.3620683	10.846452	-3.9492471	0.0031275	0.1094633	-1.6277625
Glycated Albumin	-2.8550200	10.639371	-3.2047765	0.0102643	0.1827233	-2.8090951
SRP54	0.8432810	10.263046	3.1943266	0.0104413	0.1827233	-2.8259859

	logFC	AveExpr	t	P.Value	adj.P.Val	B
LC1	-1.7534120	10.479439	-2.2241897	0.0521756	0.5211652	-4.3850333
Ro_SSA_52KDa	4.3877940	10.749773	2.2173099	0.0527726	0.5211652	-4.3957478
Fibrinogen S	2.3092792	10.364045	2.1143875	0.0625360	0.5211652	-4.5548292
La_SSB	-3.1083443	10.763517	-2.0997714	0.0640566	0.5211652	-4.5772231
ssDNA	8.2653465	12.327370	2.0723403	0.0670069	0.5211652	-4.6191082
Thyroglobulin	-3.4275093	11.284882	-1.9502264	0.0817890	0.5725230	-4.8030959
Collagen VI	6.8377980	12.853634	1.8772550	0.0920430	0.5857279	-4.9108947
Histone H1	-2.0999268	10.659861	-1.7285240	0.1167576	0.6331087	-5.1248100
MAG-FC	2.9676283	11.600661	1.7150080	0.1192826	0.6331087	-5.1438243
U1-snRNP-C	-1.4256923	10.376472	-1.6771536	0.1266217	0.6331087	-5.1966702
Histone H2B	-3.3760939	10.687850	-1.5404490	0.1566412	0.7309922	-5.3821444
Ribo phospho protein P0	1.0637014	10.577545	1.4686041	0.1748211	0.7364013	-5.4759415
Ig control	-1.6079914	11.752614	-1.4154299	0.1894348	0.7364013	-5.5435830
Anti-Ig	-1.4344682	13.987165	-1.3930530	0.1958921	0.7364013	-5.5715740
Collagen V	-0.5704798	10.255497	-1.3598755	0.2058116	0.7364013	-5.6125408
SmD	0.9906531	10.262737	1.3271242	0.2160177	0.7364013	-5.6523386
Mitochondrial antige	-1.0204828	10.412097	-1.2987101	0.2252135	0.7364013	-5.6863344
Myosin	-1.7754095	10.619415	-1.2263342	0.2501136	0.7364013	-5.7706107
MPO	1.3347899	10.492455	1.2118696	0.2553506	0.7364013	-5.7870420
PCNA	1.1997172	10.419690	1.1806515	0.2669560	0.7364013	-5.8220223
Vitronectin	1.5903499	10.548803	1.1694615	0.2712177	0.7364013	-5.8343979
Prothrombin protien	-1.0661384	10.241538	-1.1634733	0.2735205	0.7364013	-5.8409850
Sm	0.6640545	10.271042	1.1025979	0.2978217	0.7706930	-5.9065057
dsDNA	1.9218730	10.424479	1.0775988	0.3082772	0.7706930	-5.9326350
U1-snRNP-A	-25.7146259	4.926705	-1.0177759	0.3344424	0.7989930	-5.9932605
U1-snRNP-68	1.6487074	10.480563	1.0002308	0.3424256	0.7989930	-6.0105205
KU (P70_P80)	-0.7876608	10.393849	-0.9537948	0.3642374	0.8224716	-6.0550311
Sm_RNP	0.8445276	10.238264	0.8656878	0.4083638	0.8623445	-6.1346649
Nucleosome antigen	0.8667530	10.691239	0.8460148	0.4187073	0.8623445	-6.1515572
Histone H2A	-0.3943238	10.398085	-0.8457401	0.4188531	0.8623445	-6.1517908

	logFC	AveExpr	t	P.Value	adj.P.Val	B
Collagen IV	-0.3713949	10.424952	-0.8078647	0.4392739	0.8785478	-6.1833649

Deciding criteria for p-value correction

boxplot of young mice

```
knitr::opts_chunk$set(
    echo = TRUE,
    message = FALSE,
    warning = FALSE,
    root.dir = '~/Documents/3_Parkinsons_disease/Autoantibody_Data/')

setwd("~/Documents/3_Parkinsons_disease/Autoantibody_Data/SerumAntibodies_2021/")
chart_design <- theme(
   plot.title = element_text(color = "Black", size = 19, face = "bold", margin = margin
(b=25), hjust=0.4),
   axis.text.x = element_text(size=15),
   axis.text.y = element_text(size=16),
   axis.title.x = element_blank(),
   legend.text = element_text(size=11, margin = margin(t=0, r=-1,l=-1)),
   legend.title = element_blank(),
   legend.position = "bottom",
   axis.title.y = element_text(size=15.5, margin = margin(r = 5)),
   strip.text.x = element_text(size =19, margin = margin(b=25), face='bold', hjust=0.4),
   strip.background = element_blank(),
   strip.placement = "outside")

mydata <- as.matrix(IgG_raw$W_1)[,1:12]
hits <- rownames(topTable(fit.cont,number=30,adjust="BH", coef=1))
Y=matrix(nrow=30,ncol=12)
for (i in 1:30) {
   Y[i,] <- mydata[hits[i],]
}
rownames(Y) <- hits
colnames(Y) <- colData$Sample

Y <- as.data.frame(t(Y))
Y$Strain <- colData$Strain
Y$Age <- colData$Age
Y$Group <- colData$Group
Y$AGE <- c(rep("Young", 3), rep("Old", 3), rep("Young", 3), rep("Old", 3))
Y$AGE <- as.factor(Y$AGE)

hits <- colnames(Y)[1:20] #change only colnames so figure headings are meaningful

#hits elements will form file names
colnames(Y)[30] <- "B2GP"
colnames(Y)[13] <- "IF"
colnames(Y)[5] <- "KU (P70/P80)"
colnames(Y)[7] <- "La/SSB"
colnames(Y)[16] <- "Mito Antigen"
colnames(Y)[1] <- "PM/Scl100"
colnames(Y)[23] <- "RPP P2"
#colnames(Y)[20] <- "RPP P0"

par(mfrow=c(2,5))
for (i in 1:30){
   filename <- paste(hits[i], "Serum_IgG.tiff", sep="")
```

```
   p<- ggplot(Y, aes(x=Strain, y=Y[,i])) +
      geom_boxplot(aes(color=Strain), outlier.shape = NA, width = 0.40, coef=1, varwidth
=F, show.legend = F, size=0.70, position = position_dodge(0.9) ) +
     geom_jitter(aes(fill=AGE), pch=21,size=2.5,  position=position_jitterdodge(0.40)) +
      scale_color_jco() +
     scale_fill_aaas() +
      guides(color="none") +
      theme_minimal() +
      chart_design +
         ylab("Log2 Normalized Intensity")  + ylim(5.5,24.5) +
      labs(title=colnames(Y[i]), hjust=0.5) +
      scale_x_discrete("Strain", labels=c("BALB/c", "NOD"))
   tiff(filename, units="in", width=2.35, height=3.6, res=300)
   print(p)
   dev.off()
}
```

boxplot of old mice

Figure S2

```
#### Boxplots of Age ###

mydata <- as.matrix(IgG_raw$W_1)[1:70,1:12]
aging <- topTable(fit.cont,number=30,adjust="BH", coef=5)
aging <- aging[which(aging$P.Value<0.1),]
hits <- rownames(aging)
Y=matrix(nrow=nrow(aging),ncol=12)
for (i in 1:nrow(aging)) {
   Y[i,] <- mydata[hits[i],]
}
rownames(Y) <- hits
colnames(Y) <- colData$Sample

Y <- as.data.frame(t(Y))
Y$Strain <- colData$Strain
Y$Age <- colData$Age
Y$Group <- colData$Group
Y$AGE <- c(rep("Early", 3), rep("Advanced", 3), rep("Early", 3), rep("Advanced", 3))
Y$AGE <- as.factor(Y$AGE)
Y$AGE <- relevel(Y$AGE, ref="Early")
#hits elements will form file names

colnames(Y)[3] <- "Gly Albumin"
colnames(Y)[2] <- "PM/Scl100"
colnames(Y)[6] <- "Ro/SSA 52"

#Y=Y[c(4:6, 10:12),]
par(mfrow=c(2,5))
for (i in 1:11){
   setwd("~/Documents/3_Parkinsons_disease/Autoantibody_Data/SerumAntibodies_2021/")
   filename <- paste(hits[i], "Age.IgG.tiff", sep="")
   p<- ggplot(Y, aes(x=AGE, y=Y[,i])) +
      geom_boxplot(aes(color=AGE), outlier.shape = NA, width = 0.5, coef=1, varwidth=F,
show.legend = F, size=0.5) +
     geom_jitter(aes(fill=Strain), size=2.5, pch=21, width = 0.25) +
      scale_fill_jco() +
     scale_color_aaas() +
      theme_minimal() +
      chart_design +
         ylab("Log2 Normalized Intensity")  +
      labs(title=colnames(Y[i]), hjust=0.5) +
      #scale_x_discrete("Strain", labels=c("BALB/c", "NOD")) +
     #ylim(1.5,24) +
      guides(color="none")
   tiff(filename, units="in", width=2.75, height=4, res=300)
   print(p)
   dev.off()
}
```

```
```

Heatmap of normalized data

Differential Expression

#Wilcoxon Rank Sum tests were utilized to formally test for differential autoantigen expression (with respect to NOD.B10 versus BL/10 animals). P-values were adjusted by the method of Benjamini and Hochberg [@bh1995] and the corresponding “q-values” are reported. Given the discrete nature of the underlying p-value distribution, the Benjamini and Hochberg FDR estimates can be considered as conservative. The more liberal mid-P [@heller2011false] modified Benjamini and Hochberg q-values were also calculated and are reported (although, the control of FDR for that approach is not strictly guaranteed).
