# Supplementary material for: Serum and tear autoantibodies from NOD and NOR mice as potential diagnostic indicators of local and systemic inflammation in Sjögren’s disease
Source: Front Immunol. 2025 Jan 28;15:1516330. doi: 10.3389/fimmu.2024.1516330 (PMC11810956; doi:10.3389/fimmu.2024.1516330)
Supplement: Supplementary file 3 [file DataSheet3.pdf]

Supplemental Methods 1.2

Tear and Serum IgG Autoantibodies - NOR mice

Shruti Singh Kakan

2024-12-11

```
library(car)
library(lsmeans)
library(calibrate)
library(dplyr)
#library(DEGreport)
library(DESeq2)
library(DEFormats)
library(edgeR)
library(ggpubr)
library(gridExtra)
library(pheatmap)
library(reshape2)
library(RColorBrewer)
library(rstatix)
library(tidyr)
library(magrittr)
#library(PCAtools)
library(tidyverse)
library(Biobase)
library(marray)

#library(PCAtools)
library(tidyverse)
library(Biobase)
#library(marray)
library(limma)
library(gplots)

library(devtools)
#install_github("dpgaile/AutoAntArrayExmpl")
library(AutoAntArrayExmpl)

library(devtools)
#install_github("dpgaile/AutoAntArrayExmpl")
#devtools::install_github('renozao/NMF@devel')
library(AutoAntArrayExmpl)
library(NMF)
library(quantreg)
library(asbio)
library(fdrtool)
#library(discreteMTP)
library(scales)
library(ggsci)
library(ggplot2)
```

Loading Raw datasets

```
IgG_NSI <- read.csv("../../Tear_Autoantibodies_2021/IgG_MCF_SH_292_NSI_nor.csv",  header
=T, row.names = 1)[1:120,1:15]
IgG_SNR <- read.csv("../../Tear_Autoantibodies_2021/IgG_MCF_SH_292_SNR.csv",  header=T,
row.names = 1)[1:120,1:15]
```

And creating meta-data df

```
Strain <- c(rep(c("NOR", "BALBc"), each=3), rep(c("NOR"), each=6), rep(c("BALBc"), each=
3))
Biofluid <- c(rep("Tears",6), rep("Serum", 9))
colData <- as.data.frame(cbind(c(colnames(IgG_NSI)), Strain, Biofluid))
colnames(colData) <- c('Sample', "Strain", "Biofluid")
rownames(colData) <- colData$Sample

colData$Strain <- factor(colData$Strain)
colData$Strain <- relevel(colData$Strain, ref = "BALBc")
colData$Biofluid <- factor(colData$Biofluid, levels = c("Tears", "Serum"))
```

Data Filtering Based on SNR

```
#Normalization

IgG_raw=list()
IgG_raw$NSI <- as.matrix(IgG_NSI)
IgG_raw$SNR <- as.matrix(IgG_SNR)[,1:15]
IgG_SNR$average <- rowMeans(IgG_SNR)
IgG_SNR$med <- rowMedians(as.matrix(IgG_SNR))
IgG_raw$NSI <- IgG_NSI[which(IgG_SNR$med>3),]
IgG_raw$SNR <- IgG_SNR[which(IgG_SNR$med>3),]

IgG_raw$SInfo <- colData
```

Data Visualization

```
clrs=c(rep(pal_jco("default")(4)[1:2], each=3), rep(pal_jco("default")(4)[3], each=6), r
ep(pal_jco("default")(4)[4], each=3))
pchs=c(rep(2,3),rep(4,3), rep(5,6), rep(8,3))

#Centering and Scaling Differences Across Features
# Tukey Tri-mean for location
TriMnG=RowTriMeans(IgG_raw$NSI) # 99 elements (same as num of rows in NSI)
# Biweight midvariance for spread
bw.estG=unlist(apply(IgG_raw$NSI,1,r.bw)) # 99 elements
```

Normalization

First Stage

```
#enable to background correct
IgG_raw$NSI <- as.matrix(IgG_NSI[which(IgG_SNR$med>3),])
IgG_raw$SNR <- as.matrix(IgG_SNR[which(IgG_SNR$med>3),])
IgG_raw$NSI = IgG_raw$NSI*IgG_raw$SNR[,1:15] ##NSI changed permanantly
IgG_raw$X_norm_1=IgG_raw$NSI
eps=.10
IgG_raw$QregFits=array(NA,dim=c(99,2))
TriMn=RowTriMeans(IgG_raw$X_norm_1) #new NSI Tukey tri mean
ResMat=c(IgG_raw$X_norm_1,IgG_raw$X_norm_1)-matrix(rep(TriMn,30),ncol=30) #New_NSI - New
NSI_TriMn
SubMat=abs(ResMat)<quantile(as.vector(abs(ResMat)),prob=1-eps)

par(mfrow=c(2,2));par(mgp=c(1.5,.5,0));par(mar=c(2.75,2.75,1.5,0.25))
plot(rep(TriMn,30),as.vector(ResMat),
     xlim=quantile(TriMn,prob=c(0,.8)),
     ylim=quantile(as.vector(ResMat),prob=c(0.05,.95)),
     type="n",xlab="Raw Signal Tri-Mean",ylab="Raw Signal",
     main="IgG Raw Signal")
for(j in 1:15){
   points(TriMn,ResMat[,j],cex=0.5,pch=pchs[j],col=clrs[j])
   points(TriMn,ResMat[,15+j],cex=0.5,pch=pchs[j],col=clrs[j])
   y=c(ResMat[,j], ResMat[,15+j])
   x=c(TriMn, TriMn)
   fitj <- rq(y ~ x, tau = .5,subset=which(c(SubMat[,j], SubMat[,15+j])))
   abline(coef(fitj),col=clrs[j])
   IgG_raw$QregFits[j,]=coef(fitj)
   prdct=coef(fitj)[1]+coef(fitj)[2]*TriMn
   IgG_raw$X_norm_1[,j]=IgG_raw$X_norm_1[,j]-prdct
} ###substracting the model fit straight line from the data
#Xnorm_1 here is the residual value
```

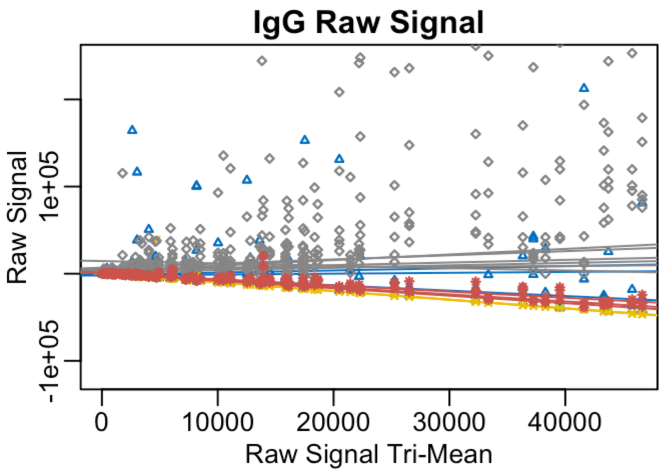
IgG Raw Signal
Raw Signal
1e+05
-1e+05
0
10000
20000
30000
40000
Raw Signal Tri-Mean

```
eps=0.10
q.eps=0.10
lambda=.10 # incremental change
nitr=75 # number of iterations -> this should help us optimize the lambda for best fit
sad=rep(0,nitr) # sum of absolute deviations
# re-init
IgG_raw$X_1step_norm_1=IgG_raw$X_norm_1 #calculated in the last loop
IgG_raw$X_norm_1=IgG_raw$NSI #reinitializing


for(i in 1:nitr){
   # snoop and grab invariants
   X=IgG_raw$X_norm_1
   iWRS=function(i) t.test(X[i,c(1:3,7:12)],X[i,c(4:5,13:15)])$p.value
   qMat=matrix(rep(mapply(iWRS,1:99),30),ncol=30)
   qMat[which(is.na(qMat[,1])==T),] <- 0 #all NANs replaced by 0
   qcut=quantile(qMat[,1],prob=q.eps)

   TriMn=RowTriMeans(IgG_raw$X_norm_1)
   ResMat=c(IgG_raw$X_norm_1,IgG_raw$X_norm_1)-matrix(rep(TriMn,30),ncol=30)
   SubMat=abs(ResMat)<quantile(as.vector(abs(ResMat)),prob=1-eps) #all residuals with ab
solute values less than the cutoff of 90%
   SubMat[qMat<qcut]=FALSE #which p values are greater than the cutoffs
   for(j in 1:15){
      y=c(ResMat[,j], ResMat[,15+j])
      x=c(TriMn, TriMn)
      fitj <- rq(y ~ x, tau = .5,subset=which(c(SubMat[,j], SubMat[,15+j])))
      prdct=coef(fitj)[1]+coef(fitj)[2]*TriMn
      IgG_raw$X_norm_1[,j]=IgG_raw$X_norm_1[,j]-lambda*prdct
      sad[i]=sad[i]+sum(abs(2*lambda*prdct))
   }###substracting the model fit straight line from the data
   print(sad)
   print(qcut)
}
```

```
##  [1] 13163746        0        0        0        0        0        0        0
##  [9]        0        0        0        0        0        0        0        0
## [17]        0        0        0        0        0        0        0        0
## [25]        0        0        0        0        0        0        0        0
## [33]        0        0        0        0        0        0        0        0
## [41]        0        0        0        0        0        0        0        0
## [49]        0        0        0        0        0        0        0        0
## [57]        0        0        0        0        0        0        0        0
## [65]        0        0        0        0        0        0        0        0
## [73]        0        0        0
##         10%
## 0.003000558
##  [1] 13163746 11530251        0        0        0        0        0        0
##  [9]        0        0        0        0        0        0        0        0
## [17]        0        0        0        0        0        0        0        0
## [25]        0        0        0        0        0        0        0        0
## [33]        0        0        0        0        0        0        0        0
## [41]        0        0        0        0        0        0        0        0
## [49]        0        0        0        0        0        0        0        0
## [57]        0        0        0        0        0        0        0        0
## [65]        0        0        0        0        0        0        0        0
## [73]        0        0        0
##         10%
## 0.004158497
##  [1] 13163746 11530251 10700883        0        0        0        0        0
##  [9]        0        0        0        0        0        0        0        0
## [17]        0        0        0        0        0        0        0        0
## [25]        0        0        0        0        0        0        0        0
## [33]        0        0        0        0        0        0        0        0
## [41]        0        0        0        0        0        0        0        0
## [49]        0        0        0        0        0        0        0        0
## [57]        0        0        0        0        0        0        0        0
## [65]        0        0        0        0        0        0        0        0
## [73]        0        0        0
##         10%
## 0.005511344
##  [1] 13163746 11530251 10700883 10213563        0        0        0        0
##  [9]        0        0        0        0        0        0        0        0
## [17]        0        0        0        0        0        0        0        0
## [25]        0        0        0        0        0        0        0        0
## [33]        0        0        0        0        0        0        0        0
## [41]        0        0        0        0        0        0        0        0
## [49]        0        0        0        0        0        0        0        0
## [57]        0        0        0        0        0        0        0        0
## [65]        0        0        0        0        0        0        0        0
## [73]        0        0        0
##         10%
## 0.007059937
##  [1] 13163746 11530251 10700883 10213563  9140283        0        0        0
##  [9]        0        0        0        0        0        0        0        0
## [17]        0        0        0        0        0        0        0        0
## [25]        0        0        0        0        0        0        0        0
```

```
## [33]        0        0        0        0        0        0        0        0
## [41]        0        0        0        0        0        0        0        0
## [49]        0        0        0        0        0        0        0        0
## [57]        0        0        0        0        0        0        0        0
## [65]        0        0        0        0        0        0        0        0
## [73]        0        0        0
##         10% 
## 0.009082488 
##  [1] 13163746 11530251 10700883 10213563  9140283 12193513        0        0
##  [9]        0        0        0        0        0        0        0        0
## [17]        0        0        0        0        0        0        0        0
## [25]        0        0        0        0        0        0        0        0
## [33]        0        0        0        0        0        0        0        0
## [41]        0        0        0        0        0        0        0        0
## [49]        0        0        0        0        0        0        0        0
## [57]        0        0        0        0        0        0        0        0
## [65]        0        0        0        0        0        0        0        0
## [73]        0        0        0
##        10% 
## 0.01171794 
##  [1] 13163746 11530251 10700883 10213563  9140283 12193513 10594154        0
##  [9]        0        0        0        0        0        0        0        0
## [17]        0        0        0        0        0        0        0        0
## [25]        0        0        0        0        0        0        0        0
## [33]        0        0        0        0        0        0        0        0
## [41]        0        0        0        0        0        0        0        0
## [49]        0        0        0        0        0        0        0        0
## [57]        0        0        0        0        0        0        0        0
## [65]        0        0        0        0        0        0        0        0
## [73]        0        0        0
##        10% 
## 0.01401411 
##  [1] 13163746 11530251 10700883 10213563  9140283 12193513 10594154  6183481
##  [9]        0        0        0        0        0        0        0        0
## [17]        0        0        0        0        0        0        0        0
## [25]        0        0        0        0        0        0        0        0
## [33]        0        0        0        0        0        0        0        0
## [41]        0        0        0        0        0        0        0        0
## [49]        0        0        0        0        0        0        0        0
## [57]        0        0        0        0        0        0        0        0
## [65]        0        0        0        0        0        0        0        0
## [73]        0        0        0
##        10% 
## 0.01504494 
##  [1] 13163746 11530251 10700883 10213563  9140283 12193513 10594154  6183481
##  [9]  5760563        0        0        0        0        0        0        0
## [17]        0        0        0        0        0        0        0        0
## [25]        0        0        0        0        0        0        0        0
## [33]        0        0        0        0        0        0        0        0
## [41]        0        0        0        0        0        0        0        0
## [49]        0        0        0        0        0        0        0        0
## [57]        0        0        0        0        0        0        0        0
```

```
## [65]        0        0        0        0        0        0        0        0
## [73]        0        0        0
##        10%
## 0.01703065
##  [1] 13163746 11530251 10700883 10213563  9140283 12193513 10594154  6183481
##  [9]  5760563  5373388        0        0        0        0        0        0
## [17]        0        0        0        0        0        0        0        0
## [25]        0        0        0        0        0        0        0        0
## [33]        0        0        0        0        0        0        0        0
## [41]        0        0        0        0        0        0        0        0
## [49]        0        0        0        0        0        0        0        0
## [57]        0        0        0        0        0        0        0        0
## [65]        0        0        0        0        0        0        0        0
## [73]        0        0        0
##        10%
## 0.02205581
##  [1] 13163746 11530251 10700883 10213563  9140283 12193513 10594154  6183481
##  [9]  5760563  5373388  4968686        0        0        0        0        0
## [17]        0        0        0        0        0        0        0        0
## [25]        0        0        0        0        0        0        0        0
## [33]        0        0        0        0        0        0        0        0
## [41]        0        0        0        0        0        0        0        0
## [49]        0        0        0        0        0        0        0        0
## [57]        0        0        0        0        0        0        0        0
## [65]        0        0        0        0        0        0        0        0
## [73]        0        0        0
##        10%
## 0.02558162
##  [1] 13163746 11530251 10700883 10213563  9140283 12193513 10594154  6183481
##  [9]  5760563  5373388  4968686  4533293        0        0        0        0
## [17]        0        0        0        0        0        0        0        0
## [25]        0        0        0        0        0        0        0        0
## [33]        0        0        0        0        0        0        0        0
## [41]        0        0        0        0        0        0        0        0
## [49]        0        0        0        0        0        0        0        0
## [57]        0        0        0        0        0        0        0        0
## [65]        0        0        0        0        0        0        0        0
## [73]        0        0        0
##        10%
## 0.02739815
##  [1] 13163746 11530251 10700883 10213563  9140283 12193513 10594154  6183481
##  [9]  5760563  5373388  4968686  4533293  6454849        0        0        0
## [17]        0        0        0        0        0        0        0        0
## [25]        0        0        0        0        0        0        0        0
## [33]        0        0        0        0        0        0        0        0
## [41]        0        0        0        0        0        0        0        0
## [49]        0        0        0        0        0        0        0        0
## [57]        0        0        0        0        0        0        0        0
## [65]        0        0        0        0        0        0        0        0
## [73]        0        0        0
##        10%
## 0.03168104
```

```
##  [1] 13163746 11530251 10700883 10213563  9140283 12193513 10594154  6183481
##  [9]  5760563  5373388  4968686  4533293  6454849  5874892        0        0
## [17]        0        0        0        0        0        0        0        0
## [25]        0        0        0        0        0        0        0        0
## [33]        0        0        0        0        0        0        0        0
## [41]        0        0        0        0        0        0        0        0
## [49]        0        0        0        0        0        0        0        0
## [57]        0        0        0        0        0        0        0        0
## [65]        0        0        0        0        0        0        0        0
## [73]        0        0        0
##      10%
## 0.035036
##  [1] 13163746 11530251 10700883 10213563  9140283 12193513 10594154  6183481
##  [9]  5760563  5373388  4968686  4533293  6454849  5874892  5460017        0
## [17]        0        0        0        0        0        0        0        0
## [25]        0        0        0        0        0        0        0        0
## [33]        0        0        0        0        0        0        0        0
## [41]        0        0        0        0        0        0        0        0
## [49]        0        0        0        0        0        0        0        0
## [57]        0        0        0        0        0        0        0        0
## [65]        0        0        0        0        0        0        0        0
## [73]        0        0        0
##        10%
## 0.04248363
##  [1] 13163746 11530251 10700883 10213563  9140283 12193513 10594154  6183481
##  [9]  5760563  5373388  4968686  4533293  6454849  5874892  5460017  4964788
## [17]        0        0        0        0        0        0        0        0
## [25]        0        0        0        0        0        0        0        0
## [33]        0        0        0        0        0        0        0        0
## [41]        0        0        0        0        0        0        0        0
## [49]        0        0        0        0        0        0        0        0
## [57]        0        0        0        0        0        0        0        0
## [65]        0        0        0        0        0        0        0        0
## [73]        0        0        0
##        10%
## 0.04044143
##  [1] 13163746 11530251 10700883 10213563  9140283 12193513 10594154  6183481
##  [9]  5760563  5373388  4968686  4533293  6454849  5874892  5460017  4964788
## [17]  4711793        0        0        0        0        0        0        0
## [25]        0        0        0        0        0        0        0        0
## [33]        0        0        0        0        0        0        0        0
## [41]        0        0        0        0        0        0        0        0
## [49]        0        0        0        0        0        0        0        0
## [57]        0        0        0        0        0        0        0        0
## [65]        0        0        0        0        0        0        0        0
## [73]        0        0        0
##        10%
## 0.04441182
##  [1] 13163746 11530251 10700883 10213563  9140283 12193513 10594154  6183481
##  [9]  5760563  5373388  4968686  4533293  6454849  5874892  5460017  4964788
## [17]  4711793  4824656        0        0        0        0        0        0
## [25]        0        0        0        0        0        0        0        0
```

```
## [33]        0        0        0        0        0        0        0        0
## [41]        0        0        0        0        0        0        0        0
## [49]        0        0        0        0        0        0        0        0
## [57]        0        0        0        0        0        0        0        0
## [65]        0        0        0        0        0        0        0        0
## [73]        0        0        0
##        10%
## 0.04693339
##  [1] 13163746 11530251 10700883 10213563  9140283 12193513 10594154  6183481
##  [9]  5760563  5373388  4968686  4533293  6454849  5874892  5460017  4964788
## [17]  4711793  4824656  3771106        0        0        0        0        0
## [25]        0        0        0        0        0        0        0        0
## [33]        0        0        0        0        0        0        0        0
## [41]        0        0        0        0        0        0        0        0
## [49]        0        0        0        0        0        0        0        0
## [57]        0        0        0        0        0        0        0        0
## [65]        0        0        0        0        0        0        0        0
## [73]        0        0        0
##        10%
## 0.04766985
##  [1] 13163746 11530251 10700883 10213563  9140283 12193513 10594154  6183481
##  [9]  5760563  5373388  4968686  4533293  6454849  5874892  5460017  4964788
## [17]  4711793  4824656  3771106  3751338        0        0        0        0
## [25]        0        0        0        0        0        0        0        0
## [33]        0        0        0        0        0        0        0        0
## [41]        0        0        0        0        0        0        0        0
## [49]        0        0        0        0        0        0        0        0
## [57]        0        0        0        0        0        0        0        0
## [65]        0        0        0        0        0        0        0        0
## [73]        0        0        0
##      10%
## 0.048124
##  [1] 13163746 11530251 10700883 10213563  9140283 12193513 10594154  6183481
##  [9]  5760563  5373388  4968686  4533293  6454849  5874892  5460017  4964788
## [17]  4711793  4824656  3771106  3751338  3583000        0        0        0
## [25]        0        0        0        0        0        0        0        0
## [33]        0        0        0        0        0        0        0        0
## [41]        0        0        0        0        0        0        0        0
## [49]        0        0        0        0        0        0        0        0
## [57]        0        0        0        0        0        0        0        0
## [65]        0        0        0        0        0        0        0        0
## [73]        0        0        0
##        10%
## 0.04974899
##  [1] 13163746 11530251 10700883 10213563  9140283 12193513 10594154  6183481
##  [9]  5760563  5373388  4968686  4533293  6454849  5874892  5460017  4964788
## [17]  4711793  4824656  3771106  3751338  3583000  3376129        0        0
## [25]        0        0        0        0        0        0        0        0
## [33]        0        0        0        0        0        0        0        0
## [41]        0        0        0        0        0        0        0        0
## [49]        0        0        0        0        0        0        0        0
## [57]        0        0        0        0        0        0        0        0
```

```
## [65]        0        0        0        0        0        0        0        0
## [73]        0        0        0
##       10% 
## 0.0530918 
##  [1] 13163746 11530251 10700883 10213563  9140283 12193513 10594154  6183481
##  [9]  5760563  5373388  4968686  4533293  6454849  5874892  5460017  4964788
## [17]  4711793  4824656  3771106  3751338  3583000  3376129  3306988        0
## [25]        0        0        0        0        0        0        0        0
## [33]        0        0        0        0        0        0        0        0
## [41]        0        0        0        0        0        0        0        0
## [49]        0        0        0        0        0        0        0        0
## [57]        0        0        0        0        0        0        0        0
## [65]        0        0        0        0        0        0        0        0
## [73]        0        0        0
##        10% 
## 0.05376584 
##  [1] 13163746 11530251 10700883 10213563  9140283 12193513 10594154  6183481
##  [9]  5760563  5373388  4968686  4533293  6454849  5874892  5460017  4964788
## [17]  4711793  4824656  3771106  3751338  3583000  3376129  3306988  3880374
## [25]        0        0        0        0        0        0        0        0
## [33]        0        0        0        0        0        0        0        0
## [41]        0        0        0        0        0        0        0        0
## [49]        0        0        0        0        0        0        0        0
## [57]        0        0        0        0        0        0        0        0
## [65]        0        0        0        0        0        0        0        0
## [73]        0        0        0
##        10% 
## 0.05435619 
##  [1] 13163746 11530251 10700883 10213563  9140283 12193513 10594154  6183481
##  [9]  5760563  5373388  4968686  4533293  6454849  5874892  5460017  4964788
## [17]  4711793  4824656  3771106  3751338  3583000  3376129  3306988  3880374
## [25]  3759932        0        0        0        0        0        0        0
## [33]        0        0        0        0        0        0        0        0
## [41]        0        0        0        0        0        0        0        0
## [49]        0        0        0        0        0        0        0        0
## [57]        0        0        0        0        0        0        0        0
## [65]        0        0        0        0        0        0        0        0
## [73]        0        0        0
##        10% 
## 0.05452308 
##  [1] 13163746 11530251 10700883 10213563  9140283 12193513 10594154  6183481
##  [9]  5760563  5373388  4968686  4533293  6454849  5874892  5460017  4964788
## [17]  4711793  4824656  3771106  3751338  3583000  3376129  3306988  3880374
## [25]  3759932  3067262        0        0        0        0        0        0
## [33]        0        0        0        0        0        0        0        0
## [41]        0        0        0        0        0        0        0        0
## [49]        0        0        0        0        0        0        0        0
## [57]        0        0        0        0        0        0        0        0
## [65]        0        0        0        0        0        0        0        0
## [73]        0        0        0
##        10% 
## 0.05435993
```

```
##  [1] 13163746 11530251 10700883 10213563  9140283 12193513 10594154  6183481
##  [9]  5760563  5373388  4968686  4533293  6454849  5874892  5460017  4964788
## [17]  4711793  4824656  3771106  3751338  3583000  3376129  3306988  3880374
## [25]  3759932  3067262  2982153        0        0        0        0        0
## [33]        0        0        0        0        0        0        0        0
## [41]        0        0        0        0        0        0        0        0
## [49]        0        0        0        0        0        0        0        0
## [57]        0        0        0        0        0        0        0        0
## [65]        0        0        0        0        0        0        0        0
## [73]        0        0        0
##        10%
## 0.05508538
##  [1] 13163746 11530251 10700883 10213563  9140283 12193513 10594154  6183481
##  [9]  5760563  5373388  4968686  4533293  6454849  5874892  5460017  4964788
## [17]  4711793  4824656  3771106  3751338  3583000  3376129  3306988  3880374
## [25]  3759932  3067262  2982153  2559632        0        0        0        0
## [33]        0        0        0        0        0        0        0        0
## [41]        0        0        0        0        0        0        0        0
## [49]        0        0        0        0        0        0        0        0
## [57]        0        0        0        0        0        0        0        0
## [65]        0        0        0        0        0        0        0        0
## [73]        0        0        0
##        10%
## 0.05652202
##  [1] 13163746 11530251 10700883 10213563  9140283 12193513 10594154  6183481
##  [9]  5760563  5373388  4968686  4533293  6454849  5874892  5460017  4964788
## [17]  4711793  4824656  3771106  3751338  3583000  3376129  3306988  3880374
## [25]  3759932  3067262  2982153  2559632  2616106        0        0        0
## [33]        0        0        0        0        0        0        0        0
## [41]        0        0        0        0        0        0        0        0
## [49]        0        0        0        0        0        0        0        0
## [57]        0        0        0        0        0        0        0        0
## [65]        0        0        0        0        0        0        0        0
## [73]        0        0        0
##        10%
## 0.05790029
##  [1] 13163746 11530251 10700883 10213563  9140283 12193513 10594154  6183481
##  [9]  5760563  5373388  4968686  4533293  6454849  5874892  5460017  4964788
## [17]  4711793  4824656  3771106  3751338  3583000  3376129  3306988  3880374
## [25]  3759932  3067262  2982153  2559632  2616106  2618145        0        0
## [33]        0        0        0        0        0        0        0        0
## [41]        0        0        0        0        0        0        0        0
## [49]        0        0        0        0        0        0        0        0
## [57]        0        0        0        0        0        0        0        0
## [65]        0        0        0        0        0        0        0        0
## [73]        0        0        0
##        10%
## 0.05977726
##  [1] 13163746 11530251 10700883 10213563  9140283 12193513 10594154  6183481
##  [9]  5760563  5373388  4968686  4533293  6454849  5874892  5460017  4964788
## [17]  4711793  4824656  3771106  3751338  3583000  3376129  3306988  3880374
## [25]  3759932  3067262  2982153  2559632  2616106  2618145  2633284        0
```

```
## [33]        0        0        0        0        0        0        0        0
## [41]        0        0        0        0        0        0        0        0
## [49]        0        0        0        0        0        0        0        0
## [57]        0        0        0        0        0        0        0        0
## [65]        0        0        0        0        0        0        0        0
## [73]        0        0        0
##        10%
## 0.06161463
##  [1] 13163746 11530251 10700883 10213563  9140283 12193513 10594154  6183481
##  [9]  5760563  5373388  4968686  4533293  6454849  5874892  5460017  4964788
## [17]  4711793  4824656  3771106  3751338  3583000  3376129  3306988  3880374
## [25]  3759932  3067262  2982153  2559632  2616106  2618145  2633284  2548234
## [33]        0        0        0        0        0        0        0        0
## [41]        0        0        0        0        0        0        0        0
## [49]        0        0        0        0        0        0        0        0
## [57]        0        0        0        0        0        0        0        0
## [65]        0        0        0        0        0        0        0        0
## [73]        0        0        0
##        10%
## 0.06304936
##  [1] 13163746 11530251 10700883 10213563  9140283 12193513 10594154  6183481
##  [9]  5760563  5373388  4968686  4533293  6454849  5874892  5460017  4964788
## [17]  4711793  4824656  3771106  3751338  3583000  3376129  3306988  3880374
## [25]  3759932  3067262  2982153  2559632  2616106  2618145  2633284  2548234
## [33]  2679168        0        0        0        0        0        0        0
## [41]        0        0        0        0        0        0        0        0
## [49]        0        0        0        0        0        0        0        0
## [57]        0        0        0        0        0        0        0        0
## [65]        0        0        0        0        0        0        0        0
## [73]        0        0        0
##        10%
## 0.06343837
##  [1] 13163746 11530251 10700883 10213563  9140283 12193513 10594154  6183481
##  [9]  5760563  5373388  4968686  4533293  6454849  5874892  5460017  4964788
## [17]  4711793  4824656  3771106  3751338  3583000  3376129  3306988  3880374
## [25]  3759932  3067262  2982153  2559632  2616106  2618145  2633284  2548234
## [33]  2679168  2752559        0        0        0        0        0        0
## [41]        0        0        0        0        0        0        0        0
## [49]        0        0        0        0        0        0        0        0
## [57]        0        0        0        0        0        0        0        0
## [65]        0        0        0        0        0        0        0        0
## [73]        0        0        0
##        10%
## 0.06199342
##  [1] 13163746 11530251 10700883 10213563  9140283 12193513 10594154  6183481
##  [9]  5760563  5373388  4968686  4533293  6454849  5874892  5460017  4964788
## [17]  4711793  4824656  3771106  3751338  3583000  3376129  3306988  3880374
## [25]  3759932  3067262  2982153  2559632  2616106  2618145  2633284  2548234
## [33]  2679168  2752559  2778658        0        0        0        0        0
## [41]        0        0        0        0        0        0        0        0
## [49]        0        0        0        0        0        0        0        0
## [57]        0        0        0        0        0        0        0        0
```

```
## [65]        0        0        0        0        0        0        0        0
## [73]        0        0        0
##        10%
## 0.06068105
##  [1] 13163746 11530251 10700883 10213563  9140283 12193513 10594154  6183481
##  [9]  5760563  5373388  4968686  4533293  6454849  5874892  5460017  4964788
## [17]  4711793  4824656  3771106  3751338  3583000  3376129  3306988  3880374
## [25]  3759932  3067262  2982153  2559632  2616106  2618145  2633284  2548234
## [33]  2679168  2752559  2778658  2798455        0        0        0        0
## [41]        0        0        0        0        0        0        0        0
## [49]        0        0        0        0        0        0        0        0
## [57]        0        0        0        0        0        0        0        0
## [65]        0        0        0        0        0        0        0        0
## [73]        0        0        0
##        10%
## 0.05976674
##  [1] 13163746 11530251 10700883 10213563  9140283 12193513 10594154  6183481
##  [9]  5760563  5373388  4968686  4533293  6454849  5874892  5460017  4964788
## [17]  4711793  4824656  3771106  3751338  3583000  3376129  3306988  3880374
## [25]  3759932  3067262  2982153  2559632  2616106  2618145  2633284  2548234
## [33]  2679168  2752559  2778658  2798455  3038104        0        0        0
## [41]        0        0        0        0        0        0        0        0
## [49]        0        0        0        0        0        0        0        0
## [57]        0        0        0        0        0        0        0        0
## [65]        0        0        0        0        0        0        0        0
## [73]        0        0        0
##        10%
## 0.05989633
##  [1] 13163746 11530251 10700883 10213563  9140283 12193513 10594154  6183481
##  [9]  5760563  5373388  4968686  4533293  6454849  5874892  5460017  4964788
## [17]  4711793  4824656  3771106  3751338  3583000  3376129  3306988  3880374
## [25]  3759932  3067262  2982153  2559632  2616106  2618145  2633284  2548234
## [33]  2679168  2752559  2778658  2798455  3038104  3117171        0        0
## [41]        0        0        0        0        0        0        0        0
## [49]        0        0        0        0        0        0        0        0
## [57]        0        0        0        0        0        0        0        0
## [65]        0        0        0        0        0        0        0        0
## [73]        0        0        0
##        10%
## 0.05988542
##  [1] 13163746 11530251 10700883 10213563  9140283 12193513 10594154  6183481
##  [9]  5760563  5373388  4968686  4533293  6454849  5874892  5460017  4964788
## [17]  4711793  4824656  3771106  3751338  3583000  3376129  3306988  3880374
## [25]  3759932  3067262  2982153  2559632  2616106  2618145  2633284  2548234
## [33]  2679168  2752559  2778658  2798455  3038104  3117171  2811762        0
## [41]        0        0        0        0        0        0        0        0
## [49]        0        0        0        0        0        0        0        0
## [57]        0        0        0        0        0        0        0        0
## [65]        0        0        0        0        0        0        0        0
## [73]        0        0        0
##        10%
## 0.06099316
```

```
##  [1] 13163746 11530251 10700883 10213563  9140283 12193513 10594154  6183481
##  [9]  5760563  5373388  4968686  4533293  6454849  5874892  5460017  4964788
## [17]  4711793  4824656  3771106  3751338  3583000  3376129  3306988  3880374
## [25]  3759932  3067262  2982153  2559632  2616106  2618145  2633284  2548234
## [33]  2679168  2752559  2778658  2798455  3038104  3117171  2811762  3067392
## [41]        0        0        0        0        0        0        0        0
## [49]        0        0        0        0        0        0        0        0
## [57]        0        0        0        0        0        0        0        0
## [65]        0        0        0        0        0        0        0        0
## [73]        0        0        0
##        10% 
## 0.06037154
##  [1] 13163746 11530251 10700883 10213563  9140283 12193513 10594154  6183481
##  [9]  5760563  5373388  4968686  4533293  6454849  5874892  5460017  4964788
## [17]  4711793  4824656  3771106  3751338  3583000  3376129  3306988  3880374
## [25]  3759932  3067262  2982153  2559632  2616106  2618145  2633284  2548234
## [33]  2679168  2752559  2778658  2798455  3038104  3117171  2811762  3067392
## [41]  2826807        0        0        0        0        0        0        0
## [49]        0        0        0        0        0        0        0        0
## [57]        0        0        0        0        0        0        0        0
## [65]        0        0        0        0        0        0        0        0
## [73]        0        0        0
##        10% 
## 0.06075595
##  [1] 13163746 11530251 10700883 10213563  9140283 12193513 10594154  6183481
##  [9]  5760563  5373388  4968686  4533293  6454849  5874892  5460017  4964788
## [17]  4711793  4824656  3771106  3751338  3583000  3376129  3306988  3880374
## [25]  3759932  3067262  2982153  2559632  2616106  2618145  2633284  2548234
## [33]  2679168  2752559  2778658  2798455  3038104  3117171  2811762  3067392
## [41]  2826807  3168293        0        0        0        0        0        0
## [49]        0        0        0        0        0        0        0        0
## [57]        0        0        0        0        0        0        0        0
## [65]        0        0        0        0        0        0        0        0
## [73]        0        0        0
##        10% 
## 0.06044656
##  [1] 13163746 11530251 10700883 10213563  9140283 12193513 10594154  6183481
##  [9]  5760563  5373388  4968686  4533293  6454849  5874892  5460017  4964788
## [17]  4711793  4824656  3771106  3751338  3583000  3376129  3306988  3880374
## [25]  3759932  3067262  2982153  2559632  2616106  2618145  2633284  2548234
## [33]  2679168  2752559  2778658  2798455  3038104  3117171  2811762  3067392
## [41]  2826807  3168293  2742779        0        0        0        0        0
## [49]        0        0        0        0        0        0        0        0
## [57]        0        0        0        0        0        0        0        0
## [65]        0        0        0        0        0        0        0        0
## [73]        0        0        0
##        10% 
## 0.06151933
##  [1] 13163746 11530251 10700883 10213563  9140283 12193513 10594154  6183481
##  [9]  5760563  5373388  4968686  4533293  6454849  5874892  5460017  4964788
## [17]  4711793  4824656  3771106  3751338  3583000  3376129  3306988  3880374
## [25]  3759932  3067262  2982153  2559632  2616106  2618145  2633284  2548234
```

```
## [33]  2679168  2752559  2778658  2798455  3038104  3117171  2811762  3067392
## [41]  2826807  3168293  2742779  3174400        0        0        0        0
## [49]        0        0        0        0        0        0        0        0
## [57]        0        0        0        0        0        0        0        0
## [65]        0        0        0        0        0        0        0        0
## [73]        0        0        0
##        10%
## 0.06069285
##  [1] 13163746 11530251 10700883 10213563  9140283 12193513 10594154  6183481
##  [9]  5760563  5373388  4968686  4533293  6454849  5874892  5460017  4964788
## [17]  4711793  4824656  3771106  3751338  3583000  3376129  3306988  3880374
## [25]  3759932  3067262  2982153  2559632  2616106  2618145  2633284  2548234
## [33]  2679168  2752559  2778658  2798455  3038104  3117171  2811762  3067392
## [41]  2826807  3168293  2742779  3174400  2848242        0        0        0
## [49]        0        0        0        0        0        0        0        0
## [57]        0        0        0        0        0        0        0        0
## [65]        0        0        0        0        0        0        0        0
## [73]        0        0        0
##        10%
## 0.06201448
##  [1] 13163746 11530251 10700883 10213563  9140283 12193513 10594154  6183481
##  [9]  5760563  5373388  4968686  4533293  6454849  5874892  5460017  4964788
## [17]  4711793  4824656  3771106  3751338  3583000  3376129  3306988  3880374
## [25]  3759932  3067262  2982153  2559632  2616106  2618145  2633284  2548234
## [33]  2679168  2752559  2778658  2798455  3038104  3117171  2811762  3067392
## [41]  2826807  3168293  2742779  3174400  2848242  2705983        0        0
## [49]        0        0        0        0        0        0        0        0
## [57]        0        0        0        0        0        0        0        0
## [65]        0        0        0        0        0        0        0        0
## [73]        0        0        0
##        10%
## 0.06131319
##  [1] 13163746 11530251 10700883 10213563  9140283 12193513 10594154  6183481
##  [9]  5760563  5373388  4968686  4533293  6454849  5874892  5460017  4964788
## [17]  4711793  4824656  3771106  3751338  3583000  3376129  3306988  3880374
## [25]  3759932  3067262  2982153  2559632  2616106  2618145  2633284  2548234
## [33]  2679168  2752559  2778658  2798455  3038104  3117171  2811762  3067392
## [41]  2826807  3168293  2742779  3174400  2848242  2705983  2898746        0
## [49]        0        0        0        0        0        0        0        0
## [57]        0        0        0        0        0        0        0        0
## [65]        0        0        0        0        0        0        0        0
## [73]        0        0        0
##        10%
## 0.06284301
##  [1] 13163746 11530251 10700883 10213563  9140283 12193513 10594154  6183481
##  [9]  5760563  5373388  4968686  4533293  6454849  5874892  5460017  4964788
## [17]  4711793  4824656  3771106  3751338  3583000  3376129  3306988  3880374
## [25]  3759932  3067262  2982153  2559632  2616106  2618145  2633284  2548234
## [33]  2679168  2752559  2778658  2798455  3038104  3117171  2811762  3067392
## [41]  2826807  3168293  2742779  3174400  2848242  2705983  2898746  2579708
## [49]        0        0        0        0        0        0        0        0
## [57]        0        0        0        0        0        0        0        0
```

```
## [65]        0        0        0        0        0        0        0        0
## [73]        0        0        0
##       10%
## 0.06188026
##  [1] 13163746 11530251 10700883 10213563  9140283 12193513 10594154  6183481
##  [9]  5760563  5373388  4968686  4533293  6454849  5874892  5460017  4964788
## [17]  4711793  4824656  3771106  3751338  3583000  3376129  3306988  3880374
## [25]  3759932  3067262  2982153  2559632  2616106  2618145  2633284  2548234
## [33]  2679168  2752559  2778658  2798455  3038104  3117171  2811762  3067392
## [41]  2826807  3168293  2742779  3174400  2848242  2705983  2898746  2579708
## [49]  2523268        0        0        0        0        0        0        0
## [57]        0        0        0        0        0        0        0        0
## [65]        0        0        0        0        0        0        0        0
## [73]        0        0        0
##       10%
## 0.06404576
##  [1] 13163746 11530251 10700883 10213563  9140283 12193513 10594154  6183481
##  [9]  5760563  5373388  4968686  4533293  6454849  5874892  5460017  4964788
## [17]  4711793  4824656  3771106  3751338  3583000  3376129  3306988  3880374
## [25]  3759932  3067262  2982153  2559632  2616106  2618145  2633284  2548234
## [33]  2679168  2752559  2778658  2798455  3038104  3117171  2811762  3067392
## [41]  2826807  3168293  2742779  3174400  2848242  2705983  2898746  2579708
## [49]  2523268  2470103        0        0        0        0        0        0
## [57]        0        0        0        0        0        0        0        0
## [65]        0        0        0        0        0        0        0        0
## [73]        0        0        0
##       10%
## 0.06433495
##  [1] 13163746 11530251 10700883 10213563  9140283 12193513 10594154  6183481
##  [9]  5760563  5373388  4968686  4533293  6454849  5874892  5460017  4964788
## [17]  4711793  4824656  3771106  3751338  3583000  3376129  3306988  3880374
## [25]  3759932  3067262  2982153  2559632  2616106  2618145  2633284  2548234
## [33]  2679168  2752559  2778658  2798455  3038104  3117171  2811762  3067392
## [41]  2826807  3168293  2742779  3174400  2848242  2705983  2898746  2579708
## [49]  2523268  2470103  2364393        0        0        0        0        0
## [57]        0        0        0        0        0        0        0        0
## [65]        0        0        0        0        0        0        0        0
## [73]        0        0        0
##       10%
## 0.06676779
##  [1] 13163746 11530251 10700883 10213563  9140283 12193513 10594154  6183481
##  [9]  5760563  5373388  4968686  4533293  6454849  5874892  5460017  4964788
## [17]  4711793  4824656  3771106  3751338  3583000  3376129  3306988  3880374
## [25]  3759932  3067262  2982153  2559632  2616106  2618145  2633284  2548234
## [33]  2679168  2752559  2778658  2798455  3038104  3117171  2811762  3067392
## [41]  2826807  3168293  2742779  3174400  2848242  2705983  2898746  2579708
## [49]  2523268  2470103  2364393  2407274        0        0        0        0
## [57]        0        0        0        0        0        0        0        0
## [65]        0        0        0        0        0        0        0        0
## [73]        0        0        0
##       10%
## 0.06724093
```

```
##  [1] 13163746 11530251 10700883 10213563  9140283 12193513 10594154  6183481
##  [9]  5760563  5373388  4968686  4533293  6454849  5874892  5460017  4964788
## [17]  4711793  4824656  3771106  3751338  3583000  3376129  3306988  3880374
## [25]  3759932  3067262  2982153  2559632  2616106  2618145  2633284  2548234
## [33]  2679168  2752559  2778658  2798455  3038104  3117171  2811762  3067392
## [41]  2826807  3168293  2742779  3174400  2848242  2705983  2898746  2579708
## [49]  2523268  2470103  2364393  2407274  2381102        0        0        0
## [57]        0        0        0        0        0        0        0        0
## [65]        0        0        0        0        0        0        0        0
## [73]        0        0        0
##        10%
## 0.06767843
##  [1] 13163746 11530251 10700883 10213563  9140283 12193513 10594154  6183481
##  [9]  5760563  5373388  4968686  4533293  6454849  5874892  5460017  4964788
## [17]  4711793  4824656  3771106  3751338  3583000  3376129  3306988  3880374
## [25]  3759932  3067262  2982153  2559632  2616106  2618145  2633284  2548234
## [33]  2679168  2752559  2778658  2798455  3038104  3117171  2811762  3067392
## [41]  2826807  3168293  2742779  3174400  2848242  2705983  2898746  2579708
## [49]  2523268  2470103  2364393  2407274  2381102  2344408        0        0
## [57]        0        0        0        0        0        0        0        0
## [65]        0        0        0        0        0        0        0        0
## [73]        0        0        0
##        10%
## 0.06851963
##  [1] 13163746 11530251 10700883 10213563  9140283 12193513 10594154  6183481
##  [9]  5760563  5373388  4968686  4533293  6454849  5874892  5460017  4964788
## [17]  4711793  4824656  3771106  3751338  3583000  3376129  3306988  3880374
## [25]  3759932  3067262  2982153  2559632  2616106  2618145  2633284  2548234
## [33]  2679168  2752559  2778658  2798455  3038104  3117171  2811762  3067392
## [41]  2826807  3168293  2742779  3174400  2848242  2705983  2898746  2579708
## [49]  2523268  2470103  2364393  2407274  2381102  2344408  2295237        0
## [57]        0        0        0        0        0        0        0        0
## [65]        0        0        0        0        0        0        0        0
## [73]        0        0        0
##        10%
## 0.06923382
##  [1] 13163746 11530251 10700883 10213563  9140283 12193513 10594154  6183481
##  [9]  5760563  5373388  4968686  4533293  6454849  5874892  5460017  4964788
## [17]  4711793  4824656  3771106  3751338  3583000  3376129  3306988  3880374
## [25]  3759932  3067262  2982153  2559632  2616106  2618145  2633284  2548234
## [33]  2679168  2752559  2778658  2798455  3038104  3117171  2811762  3067392
## [41]  2826807  3168293  2742779  3174400  2848242  2705983  2898746  2579708
## [49]  2523268  2470103  2364393  2407274  2381102  2344408  2295237  2334578
## [57]        0        0        0        0        0        0        0        0
## [65]        0        0        0        0        0        0        0        0
## [73]        0        0        0
##       10%
## 0.0708052
##  [1] 13163746 11530251 10700883 10213563  9140283 12193513 10594154  6183481
##  [9]  5760563  5373388  4968686  4533293  6454849  5874892  5460017  4964788
## [17]  4711793  4824656  3771106  3751338  3583000  3376129  3306988  3880374
## [25]  3759932  3067262  2982153  2559632  2616106  2618145  2633284  2548234
```

```
## [33]  2679168  2752559  2778658  2798455  3038104  3117171  2811762  3067392
## [41]  2826807  3168293  2742779  3174400  2848242  2705983  2898746  2579708
## [49]  2523268  2470103  2364393  2407274  2381102  2344408  2295237  2334578
## [57]  2259662        0        0        0        0        0        0        0
## [65]        0        0        0        0        0        0        0        0
## [73]        0        0        0
##        10%
## 0.07316952
##  [1] 13163746 11530251 10700883 10213563  9140283 12193513 10594154  6183481
##  [9]  5760563  5373388  4968686  4533293  6454849  5874892  5460017  4964788
## [17]  4711793  4824656  3771106  3751338  3583000  3376129  3306988  3880374
## [25]  3759932  3067262  2982153  2559632  2616106  2618145  2633284  2548234
## [33]  2679168  2752559  2778658  2798455  3038104  3117171  2811762  3067392
## [41]  2826807  3168293  2742779  3174400  2848242  2705983  2898746  2579708
## [49]  2523268  2470103  2364393  2407274  2381102  2344408  2295237  2334578
## [57]  2259662  2192297        0        0        0        0        0        0
## [65]        0        0        0        0        0        0        0        0
## [73]        0        0        0
##        10%
## 0.07548793
##  [1] 13163746 11530251 10700883 10213563  9140283 12193513 10594154  6183481
##  [9]  5760563  5373388  4968686  4533293  6454849  5874892  5460017  4964788
## [17]  4711793  4824656  3771106  3751338  3583000  3376129  3306988  3880374
## [25]  3759932  3067262  2982153  2559632  2616106  2618145  2633284  2548234
## [33]  2679168  2752559  2778658  2798455  3038104  3117171  2811762  3067392
## [41]  2826807  3168293  2742779  3174400  2848242  2705983  2898746  2579708
## [49]  2523268  2470103  2364393  2407274  2381102  2344408  2295237  2334578
## [57]  2259662  2192297  2146916        0        0        0        0        0
## [65]        0        0        0        0        0        0        0        0
## [73]        0        0        0
##        10%
## 0.07785424
##  [1] 13163746 11530251 10700883 10213563  9140283 12193513 10594154  6183481
##  [9]  5760563  5373388  4968686  4533293  6454849  5874892  5460017  4964788
## [17]  4711793  4824656  3771106  3751338  3583000  3376129  3306988  3880374
## [25]  3759932  3067262  2982153  2559632  2616106  2618145  2633284  2548234
## [33]  2679168  2752559  2778658  2798455  3038104  3117171  2811762  3067392
## [41]  2826807  3168293  2742779  3174400  2848242  2705983  2898746  2579708
## [49]  2523268  2470103  2364393  2407274  2381102  2344408  2295237  2334578
## [57]  2259662  2192297  2146916  2105493        0        0        0        0
## [65]        0        0        0        0        0        0        0        0
## [73]        0        0        0
##        10%
## 0.07891247
##  [1] 13163746 11530251 10700883 10213563  9140283 12193513 10594154  6183481
##  [9]  5760563  5373388  4968686  4533293  6454849  5874892  5460017  4964788
## [17]  4711793  4824656  3771106  3751338  3583000  3376129  3306988  3880374
## [25]  3759932  3067262  2982153  2559632  2616106  2618145  2633284  2548234
## [33]  2679168  2752559  2778658  2798455  3038104  3117171  2811762  3067392
## [41]  2826807  3168293  2742779  3174400  2848242  2705983  2898746  2579708
## [49]  2523268  2470103  2364393  2407274  2381102  2344408  2295237  2334578
## [57]  2259662  2192297  2146916  2105493  2076372        0        0        0
```

```
## [65]        0        0        0        0        0        0        0        0
## [73]        0        0        0
##        10%
## 0.08034813
##  [1] 13163746 11530251 10700883 10213563  9140283 12193513 10594154  6183481
##  [9]  5760563  5373388  4968686  4533293  6454849  5874892  5460017  4964788
## [17]  4711793  4824656  3771106  3751338  3583000  3376129  3306988  3880374
## [25]  3759932  3067262  2982153  2559632  2616106  2618145  2633284  2548234
## [33]  2679168  2752559  2778658  2798455  3038104  3117171  2811762  3067392
## [41]  2826807  3168293  2742779  3174400  2848242  2705983  2898746  2579708
## [49]  2523268  2470103  2364393  2407274  2381102  2344408  2295237  2334578
## [57]  2259662  2192297  2146916  2105493  2076372  2051449        0        0
## [65]        0        0        0        0        0        0        0        0
## [73]        0        0        0
##        10%
## 0.08148968
##  [1] 13163746 11530251 10700883 10213563  9140283 12193513 10594154  6183481
##  [9]  5760563  5373388  4968686  4533293  6454849  5874892  5460017  4964788
## [17]  4711793  4824656  3771106  3751338  3583000  3376129  3306988  3880374
## [25]  3759932  3067262  2982153  2559632  2616106  2618145  2633284  2548234
## [33]  2679168  2752559  2778658  2798455  3038104  3117171  2811762  3067392
## [41]  2826807  3168293  2742779  3174400  2848242  2705983  2898746  2579708
## [49]  2523268  2470103  2364393  2407274  2381102  2344408  2295237  2334578
## [57]  2259662  2192297  2146916  2105493  2076372  2051449  2060165        0
## [65]        0        0        0        0        0        0        0        0
## [73]        0        0        0
##        10%
## 0.08244703
##  [1] 13163746 11530251 10700883 10213563  9140283 12193513 10594154  6183481
##  [9]  5760563  5373388  4968686  4533293  6454849  5874892  5460017  4964788
## [17]  4711793  4824656  3771106  3751338  3583000  3376129  3306988  3880374
## [25]  3759932  3067262  2982153  2559632  2616106  2618145  2633284  2548234
## [33]  2679168  2752559  2778658  2798455  3038104  3117171  2811762  3067392
## [41]  2826807  3168293  2742779  3174400  2848242  2705983  2898746  2579708
## [49]  2523268  2470103  2364393  2407274  2381102  2344408  2295237  2334578
## [57]  2259662  2192297  2146916  2105493  2076372  2051449  2060165  2069633
## [65]        0        0        0        0        0        0        0        0
## [73]        0        0        0
##        10%
## 0.08338369
##  [1] 13163746 11530251 10700883 10213563  9140283 12193513 10594154  6183481
##  [9]  5760563  5373388  4968686  4533293  6454849  5874892  5460017  4964788
## [17]  4711793  4824656  3771106  3751338  3583000  3376129  3306988  3880374
## [25]  3759932  3067262  2982153  2559632  2616106  2618145  2633284  2548234
## [33]  2679168  2752559  2778658  2798455  3038104  3117171  2811762  3067392
## [41]  2826807  3168293  2742779  3174400  2848242  2705983  2898746  2579708
## [49]  2523268  2470103  2364393  2407274  2381102  2344408  2295237  2334578
## [57]  2259662  2192297  2146916  2105493  2076372  2051449  2060165  2069633
## [65]  2028246        0        0        0        0        0        0        0
## [73]        0        0        0
##        10%
## 0.08421872
```

```
##  [1] 13163746 11530251 10700883 10213563  9140283 12193513 10594154  6183481
##  [9]  5760563  5373388  4968686  4533293  6454849  5874892  5460017  4964788
## [17]  4711793  4824656  3771106  3751338  3583000  3376129  3306988  3880374
## [25]  3759932  3067262  2982153  2559632  2616106  2618145  2633284  2548234
## [33]  2679168  2752559  2778658  2798455  3038104  3117171  2811762  3067392
## [41]  2826807  3168293  2742779  3174400  2848242  2705983  2898746  2579708
## [49]  2523268  2470103  2364393  2407274  2381102  2344408  2295237  2334578
## [57]  2259662  2192297  2146916  2105493  2076372  2051449  2060165  2069633
## [65]  2028246  2075048        0        0        0        0        0        0
## [73]        0        0        0
##        10%
## 0.08450288
##  [1] 13163746 11530251 10700883 10213563  9140283 12193513 10594154  6183481
##  [9]  5760563  5373388  4968686  4533293  6454849  5874892  5460017  4964788
## [17]  4711793  4824656  3771106  3751338  3583000  3376129  3306988  3880374
## [25]  3759932  3067262  2982153  2559632  2616106  2618145  2633284  2548234
## [33]  2679168  2752559  2778658  2798455  3038104  3117171  2811762  3067392
## [41]  2826807  3168293  2742779  3174400  2848242  2705983  2898746  2579708
## [49]  2523268  2470103  2364393  2407274  2381102  2344408  2295237  2334578
## [57]  2259662  2192297  2146916  2105493  2076372  2051449  2060165  2069633
## [65]  2028246  2075048  2081453        0        0        0        0        0
## [73]        0        0        0
##        10%
## 0.08503388
##  [1] 13163746 11530251 10700883 10213563  9140283 12193513 10594154  6183481
##  [9]  5760563  5373388  4968686  4533293  6454849  5874892  5460017  4964788
## [17]  4711793  4824656  3771106  3751338  3583000  3376129  3306988  3880374
## [25]  3759932  3067262  2982153  2559632  2616106  2618145  2633284  2548234
## [33]  2679168  2752559  2778658  2798455  3038104  3117171  2811762  3067392
## [41]  2826807  3168293  2742779  3174400  2848242  2705983  2898746  2579708
## [49]  2523268  2470103  2364393  2407274  2381102  2344408  2295237  2334578
## [57]  2259662  2192297  2146916  2105493  2076372  2051449  2060165  2069633
## [65]  2028246  2075048  2081453  2044513        0        0        0        0
## [73]        0        0        0
##       10%
## 0.0854514
##  [1] 13163746 11530251 10700883 10213563  9140283 12193513 10594154  6183481
##  [9]  5760563  5373388  4968686  4533293  6454849  5874892  5460017  4964788
## [17]  4711793  4824656  3771106  3751338  3583000  3376129  3306988  3880374
## [25]  3759932  3067262  2982153  2559632  2616106  2618145  2633284  2548234
## [33]  2679168  2752559  2778658  2798455  3038104  3117171  2811762  3067392
## [41]  2826807  3168293  2742779  3174400  2848242  2705983  2898746  2579708
## [49]  2523268  2470103  2364393  2407274  2381102  2344408  2295237  2334578
## [57]  2259662  2192297  2146916  2105493  2076372  2051449  2060165  2069633
## [65]  2028246  2075048  2081453  2044513  2087802        0        0        0
## [73]        0        0        0
##        10%
## 0.08532888
##  [1] 13163746 11530251 10700883 10213563  9140283 12193513 10594154  6183481
##  [9]  5760563  5373388  4968686  4533293  6454849  5874892  5460017  4964788
## [17]  4711793  4824656  3771106  3751338  3583000  3376129  3306988  3880374
## [25]  3759932  3067262  2982153  2559632  2616106  2618145  2633284  2548234
```

```
## [33]  2679168  2752559  2778658  2798455  3038104  3117171  2811762  3067392
## [41]  2826807  3168293  2742779  3174400  2848242  2705983  2898746  2579708
## [49]  2523268  2470103  2364393  2407274  2381102  2344408  2295237  2334578
## [57]  2259662  2192297  2146916  2105493  2076372  2051449  2060165  2069633
## [65]  2028246  2075048  2081453  2044513  2087802  2049961        0        0
## [73]        0        0        0
##        10%
## 0.08576632
##  [1] 13163746 11530251 10700883 10213563  9140283 12193513 10594154  6183481
##  [9]  5760563  5373388  4968686  4533293  6454849  5874892  5460017  4964788
## [17]  4711793  4824656  3771106  3751338  3583000  3376129  3306988  3880374
## [25]  3759932  3067262  2982153  2559632  2616106  2618145  2633284  2548234
## [33]  2679168  2752559  2778658  2798455  3038104  3117171  2811762  3067392
## [41]  2826807  3168293  2742779  3174400  2848242  2705983  2898746  2579708
## [49]  2523268  2470103  2364393  2407274  2381102  2344408  2295237  2334578
## [57]  2259662  2192297  2146916  2105493  2076372  2051449  2060165  2069633
## [65]  2028246  2075048  2081453  2044513  2087802  2049961  2086942        0
## [73]        0        0        0
##        10%
## 0.08565986
##  [1] 13163746 11530251 10700883 10213563  9140283 12193513 10594154  6183481
##  [9]  5760563  5373388  4968686  4533293  6454849  5874892  5460017  4964788
## [17]  4711793  4824656  3771106  3751338  3583000  3376129  3306988  3880374
## [25]  3759932  3067262  2982153  2559632  2616106  2618145  2633284  2548234
## [33]  2679168  2752559  2778658  2798455  3038104  3117171  2811762  3067392
## [41]  2826807  3168293  2742779  3174400  2848242  2705983  2898746  2579708
## [49]  2523268  2470103  2364393  2407274  2381102  2344408  2295237  2334578
## [57]  2259662  2192297  2146916  2105493  2076372  2051449  2060165  2069633
## [65]  2028246  2075048  2081453  2044513  2087802  2049961  2086942  2051504
## [73]        0        0        0
##        10%
## 0.08617353
##  [1] 13163746 11530251 10700883 10213563  9140283 12193513 10594154  6183481
##  [9]  5760563  5373388  4968686  4533293  6454849  5874892  5460017  4964788
## [17]  4711793  4824656  3771106  3751338  3583000  3376129  3306988  3880374
## [25]  3759932  3067262  2982153  2559632  2616106  2618145  2633284  2548234
## [33]  2679168  2752559  2778658  2798455  3038104  3117171  2811762  3067392
## [41]  2826807  3168293  2742779  3174400  2848242  2705983  2898746  2579708
## [49]  2523268  2470103  2364393  2407274  2381102  2344408  2295237  2334578
## [57]  2259662  2192297  2146916  2105493  2076372  2051449  2060165  2069633
## [65]  2028246  2075048  2081453  2044513  2087802  2049961  2086942  2051504
## [73]  2110887        0        0
##       10%
## 0.0860345
##  [1] 13163746 11530251 10700883 10213563  9140283 12193513 10594154  6183481
##  [9]  5760563  5373388  4968686  4533293  6454849  5874892  5460017  4964788
## [17]  4711793  4824656  3771106  3751338  3583000  3376129  3306988  3880374
## [25]  3759932  3067262  2982153  2559632  2616106  2618145  2633284  2548234
## [33]  2679168  2752559  2778658  2798455  3038104  3117171  2811762  3067392
## [41]  2826807  3168293  2742779  3174400  2848242  2705983  2898746  2579708
## [49]  2523268  2470103  2364393  2407274  2381102  2344408  2295237  2334578
## [57]  2259662  2192297  2146916  2105493  2076372  2051449  2060165  2069633
```

```
## [65]  2028246  2075048  2081453  2044513  2087802  2049961  2086942  2051504
## [73]  2110887  2052021        0
##        10%
## 0.08645297
##  [1] 13163746 11530251 10700883 10213563  9140283 12193513 10594154  6183481
##  [9]  5760563  5373388  4968686  4533293  6454849  5874892  5460017  4964788
## [17]  4711793  4824656  3771106  3751338  3583000  3376129  3306988  3880374
## [25]  3759932  3067262  2982153  2559632  2616106  2618145  2633284  2548234
## [33]  2679168  2752559  2778658  2798455  3038104  3117171  2811762  3067392
## [41]  2826807  3168293  2742779  3174400  2848242  2705983  2898746  2579708
## [49]  2523268  2470103  2364393  2407274  2381102  2344408  2295237  2334578
## [57]  2259662  2192297  2146916  2105493  2076372  2051449  2060165  2069633
## [65]  2028246  2075048  2081453  2044513  2087802  2049961  2086942  2051504
## [73]  2110887  2052021  2109858
##      10%
## 0.086279
```

```
plot(1:nitr,sad,main="iterative adj IgG")
```

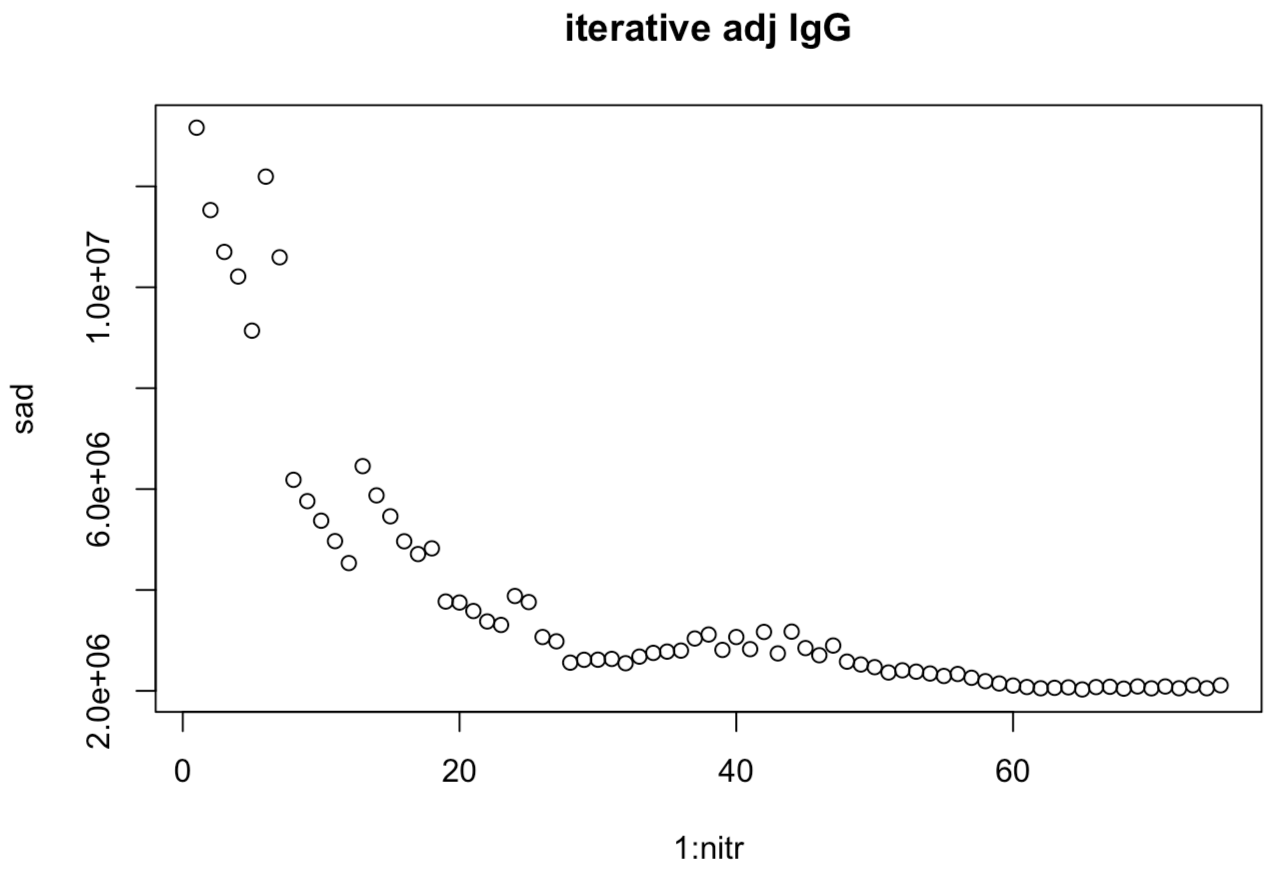


```
smoothScatter(IgG_raw$X_1step_norm_1,IgG_raw$X_norm_1, nbin = 500)
abline(0,1,col="steelblue")
```

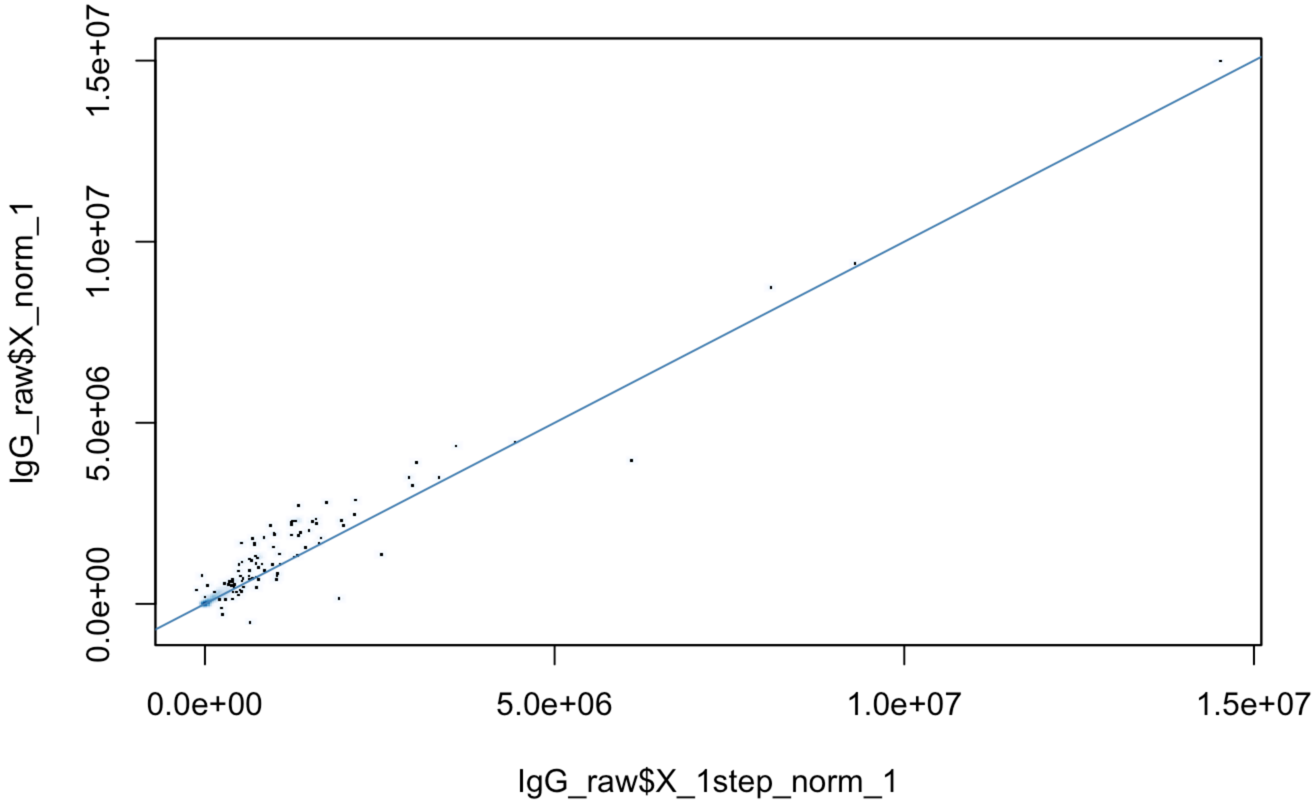

Second Stage

```
deltaG=min(as.vector(IgG_raw$X_norm_1))
IgG_raw$W_1=log(IgG_raw$X_norm_1-deltaG+1)
# get resids..
W_1=IgG_raw$W_1
RW=RowTriMeans(W_1)
# get residual matrices
RW_MAT=matrix(rep(RW,dim(W_1)[2]),ncol=dim(W_1)[2])
W_1=W_1-RW_MAT

W=W_1
bw.est=unlist(apply(W,2,r.bw))
# now, rescale
for(j in 1:15){
   W_1[,j]=W_1[,j]/sqrt(bw.est[j])
}
# now, get new values..
IgG_raw$W_1=RW_MAT+W_1
```

Formal Comparison of NOR and Balb/c

AutoAntigen Expresion Profiles

First Stage with visualizations

Normalization Second Stage

Density Plot of Normalized Data

```
plot(density(log2(IgG_raw$W_1[,1]-min(IgG_raw$W_1))-6.66), main="(logW_1) ", sub=" . ",
type="l")
for(j in 2:15) {
   dens<-density(log2(IgG_raw$W_1[,j]-min(IgG_raw$W_1))-6.66)
   lines(dens, cex=0.5,pch=pchs[j],col=clrs[j],cex.main=1)
} #165.4 = -1* min(IgG_raw$W_1); 6.25 = log2(max(IgG_raw$W_1))
```

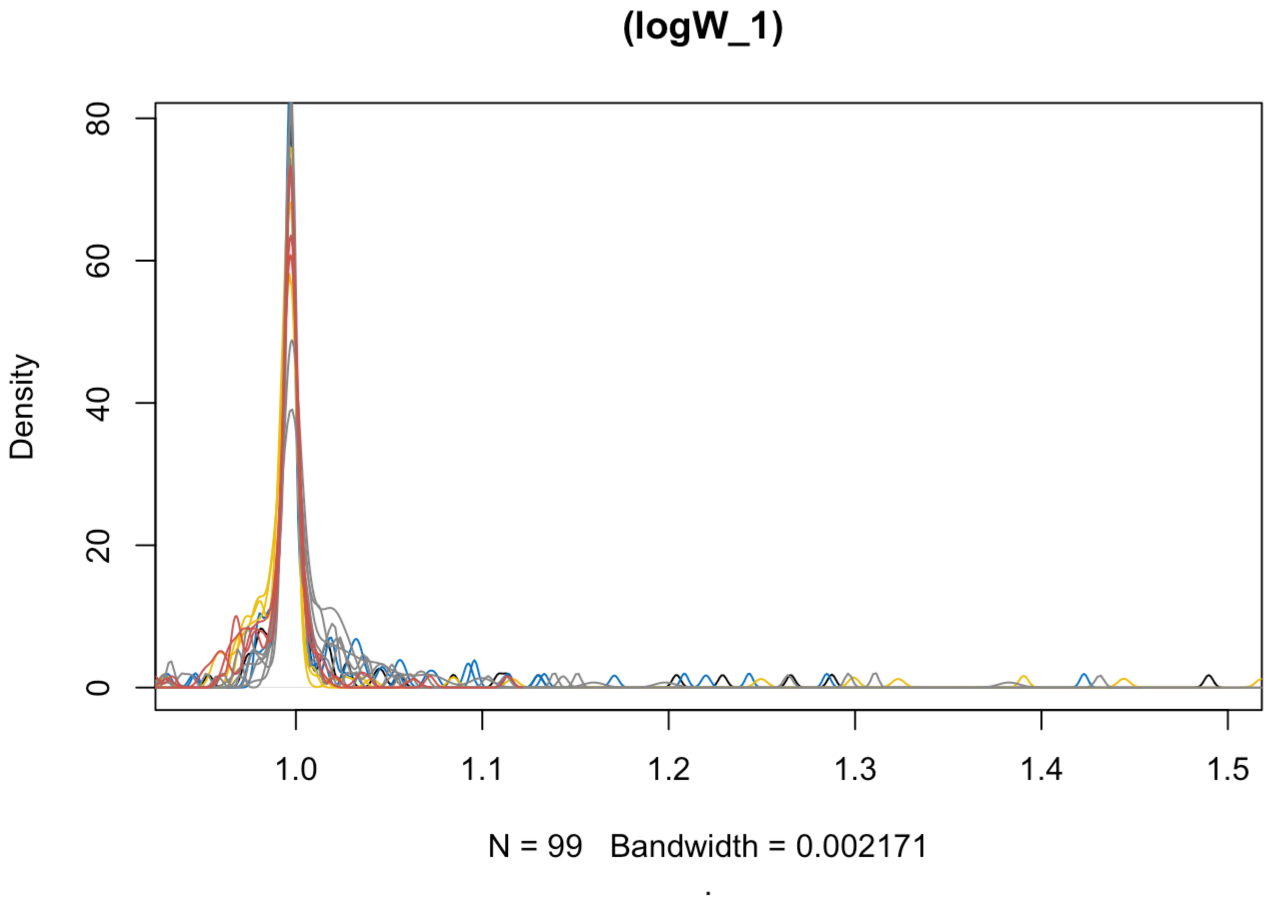

Heatmap of Normalized Data

```
RnkSmplW_1=matrix(nrow=99,ncol=15)
RnkSmplW_1[,1]=IgG_raw$W_1[,1]
for(j in 2:15) RnkSmplW_1[,j]=IgG_raw$W_1[,j]
for(i in 1:99) RnkSmplW_1[i,]=rank(RnkSmplW_1[i,])/(15)
colnames(RnkSmplW_1)=as.character((colData$Sample))
rownames(RnkSmplW_1) <- rownames(IgG_raw$W_1)

#tiff("fig8.tiff", units="in", width=6, height=14, res=500)
pheatmap(RnkSmplW_1,annCol=factor(colData$Strain),main="Rank-it (Across Samples) Normali
zed Signal IgG",dist="euclidean",hclust="ward", cutree_rows = 6, cutree_cols = 3)
```

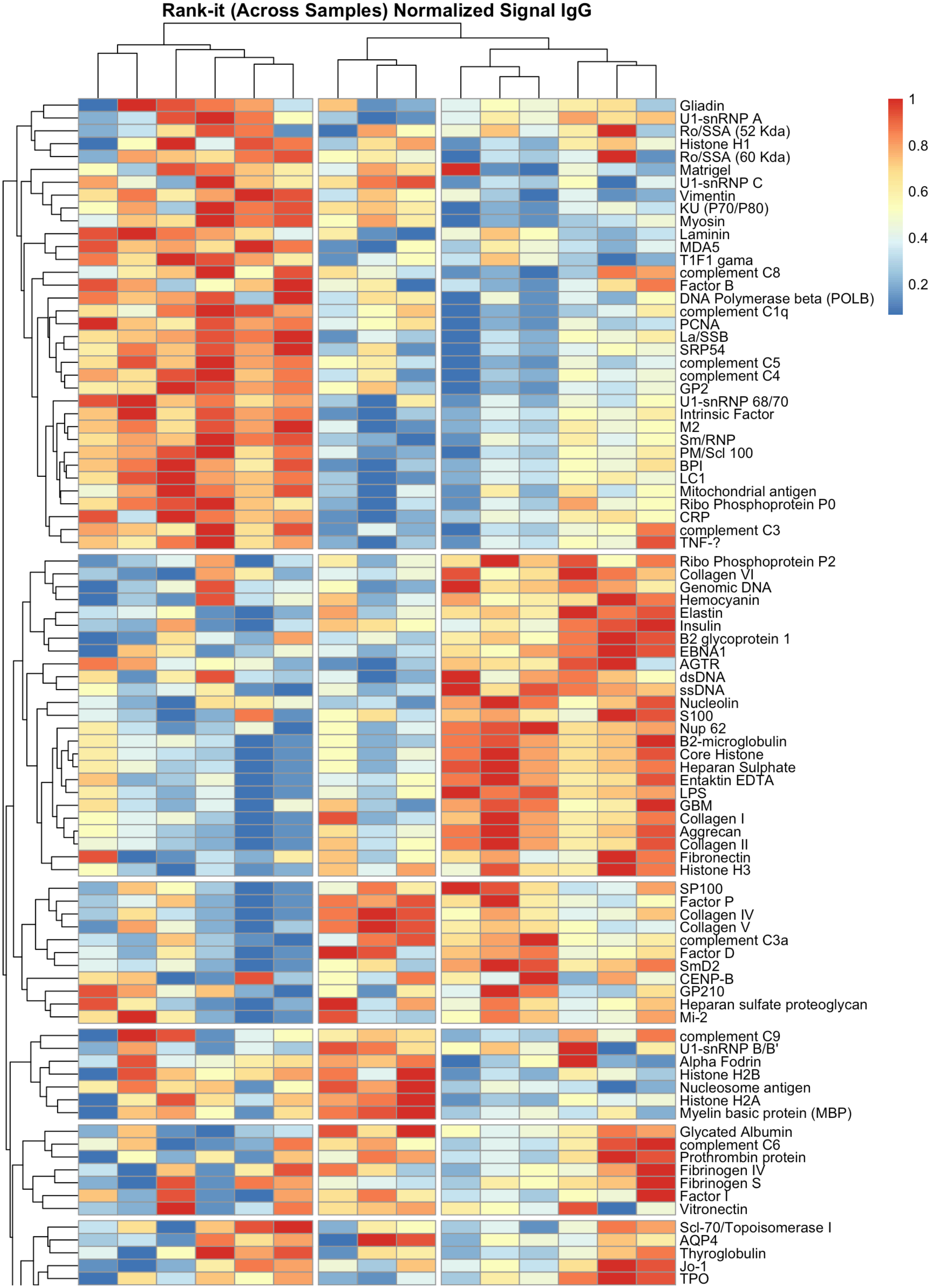
Rank-it (Across Samples) Normalized Signal IgG
Gliadin
U1-snRNP A
Ro/SSA (52 Kda)
Histone H1
Ro/SSA (60 Kda)
Matrigel
U1-snRNP C
Vimentin
KU (P70/P80)
Myosin
Laminin
MDA5
T1F1 gama
complement C8
Factor B
DNA Polymerase beta (POLB)
complement C1q
PCNA
La/SSB
SRP54
complement C5
complement C4
GP2
U1-snRNP 68/70
Intrinsic Factor
M2
Sm/RNP
PM/Scl 100
BPI
LC1
Mitochondrial antigen
Ribo Phosphoprotein P0
CRP
complement C3
TNF-?
Ribo Phosphoprotein P2
Collagen VI
Genomic DNA
Hemocyanin
Elastin
Insulin
B2 glycoprotein 1
EBNA1
AGTR
dsDNA
ssDNA
Nucleolin
S100
Nup 62
B2-microglobulin
Core Histone
Heparan Sulphate
Entaktin EDTA
LPS
GBM
Collagen I
Aggrecan
Collagen II
Fibronectin
Histone H3
SP100
Factor P
Collagen IV
Collagen V
complement C3a
Factor D
SmD2
CENP-B
GP210
Heparan sulfate proteoglycan
Mi-2
complement C9
U1-snRNP B/B'
Alpha Fodrin
Histone H2B
Nucleosome antigen
Histone H2A
Myelin basic protein (MBP)
Glycated Albumin
complement C6
Prothrombin protein
Fibrinogen IV
Fibrinogen S
Factor I
Vitronectin
Scl-70/Topoisomerase I
AQP4
Thyroglobulin
Jo-1
TPO
1
0.8
0.6
0.4
0.2

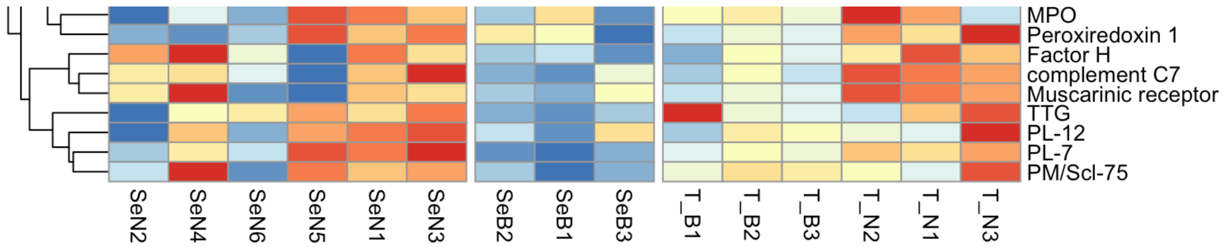


```
#dev.off()
```

Formal Comparison of NOR and Balb AutoAntigen Expresion Profiles

```
mydata <- as.matrix(IgG_raw$W_1)
#mydata <- as.matrix(IgG_raw$X_nrm_1)
colData$Strain <- relevel(colData$Strain, ref = "BALBc")
colData$Biofluid <- factor(colData$Biofluid)

conditions<- paste(colData$Strain, colData$Biofluid,sep=".")
conditions <- factor(conditions, levels=unique(conditions))
design <- model.matrix(~0+ conditions)
rownames(design) <- colnames(mydata)
colnames(design) <- levels(conditions)
fit <- lmFit(mydata, design)
#sqrt(anova.MAList(fit)["Residuals","Mean Sq"]) #???
summary(fit)
```

```
##                  Length Class  Mode
## coefficients     396    -none- numeric
## rank               1    -none- numeric
## assign             4    -none- numeric
## qr                 5    qr     list
## df.residual       99    -none- numeric
## sigma             99    -none- numeric
## cov.coefficients  16    -none- numeric
## stdev.unscaled   396    -none- numeric
## pivot              4    -none- numeric
## Amean             99    -none- numeric
## method             1    -none- character
## design            60    -none- numeric
```

```
cont.matrix<- makeContrasts(
   NTvsBT =  NOR.Tears - BALBc.Tears,
   NSvBS = NOR.Serum - BALBc.Serum,
   NTvNS = NOR.Tears - NOR.Serum,
   levels = design)

fit.cont<- contrasts.fit(fit, cont.matrix)
fit.cont<- eBayes(fit.cont)
qqt(fit.cont$t,df=fit.cont$df.prior+fit.cont$df.residual,cex=1)
abline(0,2)
```

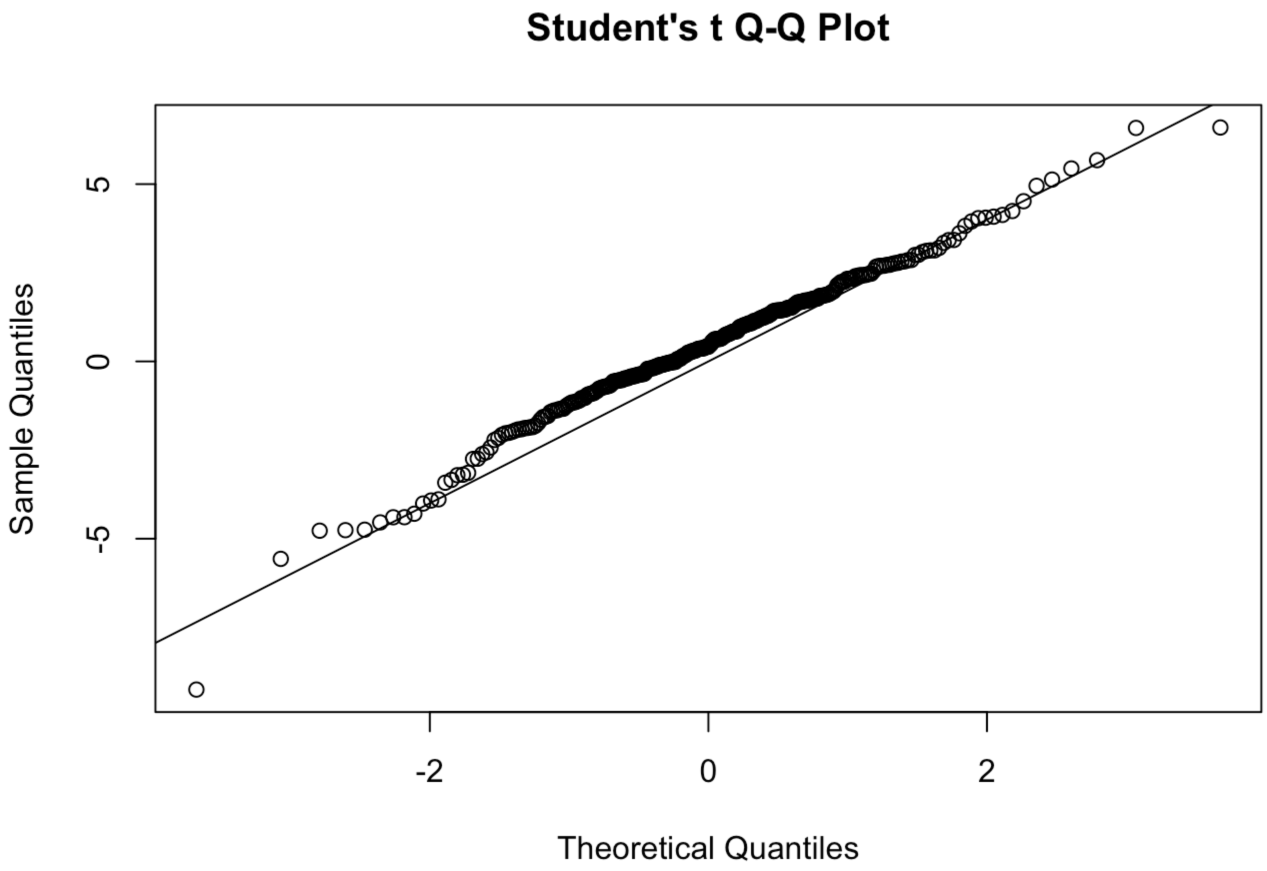


```
topTable(fit.cont,number=23,adjust="BH")
```

```
##                                NTvsBT       NSvBS       NTvNS  AveExpr
## Collagen V                -0.07038969  -0.90916256  0.01482945 13.29854
## PM/Scl 100                 1.87011347  10.32646638 -8.71748160 13.30225
## La/SSB                     3.67062968   9.26685522 -6.18703226 13.31754
## M2                         0.59978187   3.72028076 -2.97650860 13.32384
## complement C4              1.06599172   5.32235978 -5.11504829 13.12386
## complement C5              1.43905186   4.42497997 -5.18203376 12.97633
## Sm/RNP                     0.12635785   0.83423924 -0.59654575 13.13599
## SRP54                      0.20850996   0.57315350 -0.55091449 13.14266
## EBNA1                      3.71531518   0.85450638  5.77995781 13.82567
## Nucleosome antigen        -0.07777548  -1.05198767 -0.37586479 13.35894
## Myelin basic protein (MBP)  -0.02116834  -2.33095007 -0.18789263 13.51833
## LC1                        1.76213063  10.42223664 -7.85269218 12.99791
## B2 glycoprotein 1         23.90249097   1.93090745 24.46555828 16.26465
## DNA Polymerase beta (POLB)   0.45656096   1.26751521 -1.59312557 13.26543
## Ribo Phosphoprotein P0     3.77884097   9.37381384 -5.02547353 12.94322
## complement C3              3.68812703   6.81751782 -3.31007126 13.85892
## Nup 62                    -0.10793438  -0.02146837  0.14554683 13.17288
## PCNA                       1.07050685   4.02231597 -5.59521475 12.72061
## Glycated Albumin           6.86739009 -13.62262474  9.42079668 15.34936
## Collagen IV               -0.01926200  -0.35168868  0.10657590 13.18397
## LPS                       -0.23577020  -0.05260488  0.22383595 13.19888
## Insulin                    3.01863176  -0.32052681  3.15885966 13.70488
## TNF-?                      3.01285416   5.20379844 -1.73616430 13.31045
##                                    F      P.Value   adj.P.Val
## Collagen V                 32.664952 6.216939e-06 0.000615477
## PM/Scl 100                 24.305363 2.752940e-05 0.001067273
## La/SSB                     23.522553 3.234162e-05 0.001067273
## M2                         16.734187 1.641577e-04 0.003554918
## complement C4              16.213455 1.898929e-04 0.003554918
## complement C5              15.772425 2.154496e-04 0.003554918
## Sm/RNP                     13.754198 3.987711e-04 0.005639762
## SRP54                      12.657053 5.739973e-04 0.007103217
## EBNA1                      11.267297 9.432834e-04 0.010376117
## Nucleosome antigen         10.906968 1.080725e-03 0.010699173
## Myelin basic protein (MBP)   9.890412 1.615118e-03 0.014536064
## LC1                         8.870577 2.490098e-03 0.020543308
## B2 glycoprotein 1           8.453360 3.001444e-03 0.022847978
## DNA Polymerase beta (POLB)   8.119831 3.500143e-03 0.022847978
## Ribo Phosphoprotein P0      8.056526 3.605422e-03 0.022847978
## complement C3               8.005720 3.692603e-03 0.022847978
## Nup 62                      7.119876 5.693532e-03 0.033156449
## PCNA                        6.935902 6.255077e-03 0.034402921
## Glycated Albumin            6.483801 7.933705e-03 0.038276824
## Collagen IV                 6.448995 8.083499e-03 0.038276824
## LPS                         6.440781 8.119326e-03 0.038276824
## Insulin                     6.117237 9.690414e-03 0.043606863
## TNF-?                       5.470665 1.403067e-02 0.060392883
```

```
nTvsBT <- topTable(fit.cont, number=30,coef=1, adjust.method = 'BH')
nSvsBS <- topTable(fit.cont, number=30,coef=2, adjust.method = 'BH')
nTvsBT$Antigen <- row.names(nTvsBT)
nSvsBS$Antigen <- row.names(nSvsBS)
NTvnTvBT <- full_join(nTvsBT, nSvsBS, by="Antigen", suffix = c(".Tear", ".Serum"))
rownames(NTvnTvBT) <- NTvnTvBT$Antigen
#NTvnTvBT[is.na(NTvnTvBT)] <- 0.5
NTvnTvBT <- NTvnTvBT[c(which(NTvnTvBT$P.Value.Serum < 0.1 & NTvnTvBT$logFC.Serum > 0.
5)),]

NTvnTvBT
```

```
##                            logFC.Tear AveExpr.Tear   t.Tear P.Value.Tear
## La/SSB                       3.670630     13.31754 2.258592   0.04410169
## complement C3                3.688127     13.85892 1.849519   0.09009262
## TNF-?                        3.012854     13.31045 1.715172   0.11295863
## PL-7                         3.980278     12.67258 1.464658   0.16966767
## PM/Scl 100                         NA           NA       NA           NA
## M2                                 NA           NA       NA           NA
## Sm/RNP                             NA           NA       NA           NA
## complement C4                      NA           NA       NA           NA
## LC1                                NA           NA       NA           NA
## Ribo Phosphoprotein P0             NA           NA       NA           NA
## SRP54                              NA           NA       NA           NA
## complement C5                      NA           NA       NA           NA
## CRP                                NA           NA       NA           NA
## Mitochondrial antigen              NA           NA       NA           NA
## Intrinsic Factor                   NA           NA       NA           NA
## BPI                                NA           NA       NA           NA
## DNA Polymerase beta (POLB)         NA           NA       NA           NA
## U1-snRNP 68/70                     NA           NA       NA           NA
## PCNA                               NA           NA       NA           NA
## U1-snRNP A                         NA           NA       NA           NA
##                            adj.P.Val.Tear    B.Tear
## La/SSB                          0.4106702 -3.716716
## complement C3                   0.4862132 -4.294441
## TNF-?                           0.4862132 -4.472600
## PL-7                            0.5190220 -4.784113
## PM/Scl 100                             NA        NA
## M2                                     NA        NA
## Sm/RNP                                 NA        NA
## complement C4                          NA        NA
## LC1                                    NA        NA
## Ribo Phosphoprotein P0                 NA        NA
## SRP54                                  NA        NA
## complement C5                          NA        NA
## CRP                                    NA        NA
## Mitochondrial antigen                  NA        NA
## Intrinsic Factor                       NA        NA
## BPI                                    NA        NA
## DNA Polymerase beta (POLB)             NA        NA
## U1-snRNP 68/70                         NA        NA
## PCNA                                   NA        NA
## U1-snRNP A                             NA        NA
##                                              Antigen logFC.Serum
## La/SSB                                        La/SSB   9.2668552
## complement C3                          complement C3   6.8175178
## TNF-?                                          TNF-?   5.2037984
## PL-7                                            PL-7   6.3420258
## PM/Scl 100                                PM/Scl 100  10.3264664
## M2                                                M2   3.7202808
## Sm/RNP                                        Sm/RNP   0.8342392
## complement C4                          complement C4   5.3223598
## LC1                                              LC1  10.4222366
```

```
## Ribo Phosphoprotein P0          Ribo Phosphoprotein P0   9.3738138
## SRP54                                            SRP54   0.5731535
## complement C5                            complement C5   4.4249800
## CRP                                                CRP  28.5472805
## Mitochondrial antigen           Mitochondrial antigen   0.6471833
## Intrinsic Factor                     Intrinsic Factor  10.3517712
## BPI                                                BPI   8.0190689
## DNA Polymerase beta (POLB)  DNA Polymerase beta (POLB)    1.2675152
## U1-snRNP 68/70                          U1-snRNP 68/70   1.5578799
## PCNA                                              PCNA   4.0223160
## U1-snRNP A                                  U1-snRNP A   1.4764511
##                            AveExpr.Serum  t.Serum P.Value.Serum
## La/SSB                          13.31754 6.584139  3.106912e-05
## complement C3                   13.85892 3.947740  2.075446e-03
## TNF-?                           13.31045 3.420734  5.334761e-03
## PL-7                            12.67258 2.694760  2.005642e-02
## PM/Scl 100                      13.30225 6.596628  3.052601e-05
## M2                              13.32384 5.675088  1.186951e-04
## Sm/RNP                          13.13599 5.440759  1.708320e-04
## complement C4                   13.12386 4.951787  3.744450e-04
## LC1                             12.99791 4.240582  1.243137e-03
## Ribo Phosphoprotein P0          12.94322 4.131191  1.503717e-03
## SRP54                           13.14266 4.081338  1.640718e-03
## complement C5                   12.97633 4.052915  1.724564e-03
## CRP                             12.71956 3.424604  5.297482e-03
## Mitochondrial antigen           13.19187 3.082465  9.876731e-03
## Intrinsic Factor                13.26876 2.821519  1.591367e-02
## BPI                             13.66722 2.814242  1.612665e-02
## DNA Polymerase beta (POLB)      13.26543 2.721126  1.911495e-02
## U1-snRNP 68/70                  13.18928 2.691621  2.017151e-02
## PCNA                            12.72061 2.402329  3.408541e-02
## U1-snRNP A                      13.22930 2.188065  4.999253e-02
##                            adj.P.Val.Serum    B.Serum
## La/SSB                         0.001025281  2.4722748
## complement C3                  0.013697944 -1.8686933
## TNF-?                          0.031067136 -2.8264721
## PL-7                           0.079879197 -4.1430099
## PM/Scl 100                     0.001025281  2.4905812
## M2                             0.002937704  1.0811248
## Sm/RNP                         0.003382473  0.7036153
## complement C4                  0.006178342 -0.1085938
## LC1                            0.012307056 -1.3443290
## Ribo Phosphoprotein P0         0.013133214 -1.5393185
## SRP54                          0.013133214 -1.6285468
## complement C5                  0.013133214 -1.6795168
## CRP                            0.031067136 -2.8194053
## Mitochondrial antigen          0.048889819 -3.4436923
## Intrinsic Factor               0.072569915 -3.9160578
## BPI                            0.072569915 -3.9291403
## DNA Polymerase beta (POLB)     0.079879197 -4.0959793
## U1-snRNP 68/70                 0.079879197 -4.1486023
```

```
## PCNA                          0.129786739 -4.6566070
## U1-snRNP A                    0.176759289 -5.0205177
```

```
knitr::kable(NTvnTvBT[c(1:2,5,8,12)])
```

	logFC.Tear	AveExpr.Tear	adj.P.Val.Tear	logFC.Serum	adj.P.Val.Serum
La/SSB	3.670630	13.31754	0.4106702	9.2668552	0.0010253
complement C3	3.688127	13.85892	0.4862132	6.8175178	0.0136979
TNF-?	3.012854	13.31045	0.4862132	5.2037984	0.0310671
PL-7	3.980278	12.67258	0.5190220	6.3420258	0.0798792
PM/Scl 100	NA	NA	NA	10.3264664	0.0010253
M2	NA	NA	NA	3.7202808	0.0029377
Sm/RNP	NA	NA	NA	0.8342392	0.0033825
complement C4	NA	NA	NA	5.3223598	0.0061783
LC1	NA	NA	NA	10.4222366	0.0123071
Ribo Phosphoprotein P0	NA	NA	NA	9.3738138	0.0131332
SRP54	NA	NA	NA	0.5731535	0.0131332
complement C5	NA	NA	NA	4.4249800	0.0131332
CRP	NA	NA	NA	28.5472805	0.0310671
Mitochondrial antigen	NA	NA	NA	0.6471833	0.0488898
Intrinsic Factor	NA	NA	NA	10.3517712	0.0725699
BPI	NA	NA	NA	8.0190689	0.0725699
DNA Polymerase beta (POLB)	NA	NA	NA	1.2675152	0.0798792
U1-snRNP 68/70	NA	NA	NA	1.5578799	0.0798792
PCNA	NA	NA	NA	4.0223160	0.1297867
U1-snRNP A	NA	NA	NA	1.4764511	0.1767593

```
Result_Serum_NOR_IgG <- as.data.frame(
   cbind(rownames(NTvnTvBT),
         round(NTvnTvBT$adj.P.Val.Serum, 6),
         paste0(round(NTvnTvBT$logFC.Serum, 2),
                " (",
                (round(2^NTvnTvBT$logFC.Serum, 2)),
                ")")
         )
   )

setwd("~/Documents/3_Parkinsons_disease/Autoantibody_Data/Tear_Autoantibodies_2021/")
colnames(Result_Serum_NOR_IgG) <- c("Antigen", "p adj NOR", "Log2FC (FC) NOR")
Result_Serum_NOR_IgG
```

```
##                        Antigen p adj NOR      Log2FC (FC) NOR
## 1                       La/SSB  0.001025        9.27 (616.03)
## 2                complement C3  0.013698        6.82 (112.79)
## 3                        TNF-?  0.031067         5.2 (36.86)
## 4                         PL-7  0.079879        6.34 (81.12)
## 5                  PM/Scl 100  0.001025      10.33 (1284.03)
## 6                           M2  0.002938        3.72 (13.18)
## 7                       Sm/RNP  0.003382         0.83 (1.78)
## 8                complement C4  0.006178        5.32 (40.01)
## 9                          LC1  0.012307      10.42 (1372.16)
## 10      Ribo Phosphoprotein P0  0.013133        9.37 (663.44)
## 11                       SRP54  0.013133         0.57 (1.49)
## 12               complement C5  0.013133        4.42 (21.48)
## 13                         CRP  0.031067 28.55 (392272366.33)
## 14       Mitochondrial antigen   0.04889         0.65 (1.57)
## 15            Intrinsic Factor   0.07257      10.35 (1306.75)
## 16                         BPI   0.07257        8.02 (259.41)
## 17 DNA Polymerase beta (POLB)   0.079879         1.27 (2.41)
## 18              U1-snRNP 68/70  0.079879         1.56 (2.94)
## 19                        PCNA  0.129787        4.02 (16.25)
## 20                  U1-snRNP A  0.176759         1.48 (2.78)
```

```
write.csv(Result_Serum_NOR_IgG, file="NOR_Serum_IgG_I.csv", sep=',')
```

```
## Warning in write.csv(Result_Serum_NOR_IgG, file = "NOR_Serum_IgG_I.csv", :
## attempt to set 'sep' ignored
```

R-Squared .... goodness of fit

```
for (i in 1:99){
   sst <- rowSums(mydata^2)
   ssr <- sst - fit.cont$df.residual*(fit.cont$sigma^2)
   Rsq<- (ssr/sst)
}
plot(1:99, Rsq)
```

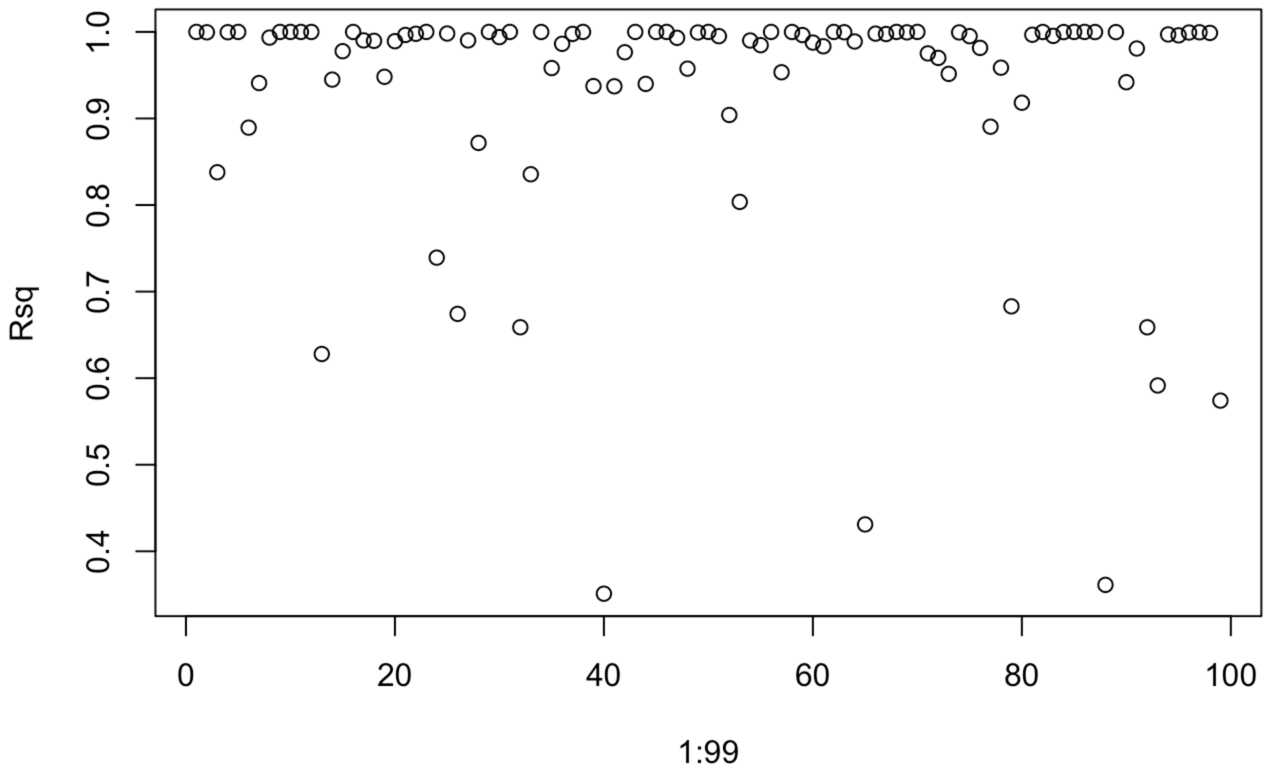


```
which(Rsq<0.5)
```

```
##            Gliadin Muscarinic receptor              ssDNA
##                 40                  65                 88
```

```
summary(fit.cont$r.squared)
```

```
## Length  Class   Mode
##      0   NULL   NULL
```

Determining which tests to use

DE Analysis

#Generate outputs for IgG Datasets

Serum boxplot

#Identifying Serum hits for Serum hits

```
fitObjres <- NTvnTvBT[which(NTvnTvBT$P.Value.Serum < 0.05 & abs(NTvnTvBT$logFC.Serum) > 
0.5), ]
mydata <- as.matrix(IgG_raw$W_1)[1:99,]
hits <- rownames(fitObjres)
```

Serum boxplots

```
library(ggplot2)
library(scales)
chart_design <- theme(
   plot.title = element_text(color = "Black", size = 17, face = "bold", margin = margin
(b=25), hjust=0.4),
   axis.text.x = element_text(size=15),
   axis.text.y = element_text(size=14),
   axis.title.x = element_blank(),
   legend.text = element_blank(),
   legend.title = element_blank(),
   legend.position = "",
   axis.title.y = element_text(size=15, margin = margin(r = 5)),
   strip.text.x = element_text(size =17, margin = margin(b=25), face='bold', hjust=0.4),
   strip.background = element_blank(),
   strip.placement = "outside")

hits
```

```
##  [1] "La/SSB"                       "complement C3"
##  [3] "TNF-?"                        "PL-7"
##  [5] "PM/Scl 100"                   "M2"
##  [7] "Sm/RNP"                       "complement C4"
##  [9] "LC1"                          "Ribo Phosphoprotein P0"
## [11] "SRP54"                        "complement C5"
## [13] "CRP"                          "Mitochondrial antigen"
## [15] "Intrinsic Factor"             "BPI"
## [17] "DNA Polymerase beta (POLB) " "U1-snRNP 68/70"
## [19] "PCNA"                         "U1-snRNP A"
```

```
Y=matrix(nrow=length(hits),ncol=15)
for (i in 1:length(hits)) {
   Y[i,] <- mydata[hits[i],]
}
hits
```


```
##  [1] "La/SSB"                       "complement C3"
##  [3] "TNF-?"                        "PL-7"
##  [5] "PM/Scl 100"                   "M2"
##  [7] "Sm/RNP"                       "complement C4"
##  [9] "LC1"                          "Ribo Phosphoprotein P0"
## [11] "SRP54"                        "complement C5"
## [13] "CRP"                          "Mitochondrial antigen"
## [15] "Intrinsic Factor"             "BPI"
## [17] "DNA Polymerase beta (POLB) " "U1-snRNP 68/70"
## [19] "PCNA"                         "U1-snRNP A"
```

```
rownames(Y) <- hits
colnames(Y) <- colData$Sample

Y <- as.data.frame(t(Y))
Y$Strain <- colData$Strain
Y$Biofluid <- colData$Biofluid


hits <- colnames(Y)[1:(ncol(Y)-2)]
Y_Ser <- Y[which(Y$Biofluid=="Serum"),]

colnames(Y_Ser)[1] <- "La SSB"
colnames(Y_Ser)[2] <- "C3"
colnames(Y_Ser)[3] <- "TNF alpha"
colnames(Y_Ser)[5] <- "PM Scl100"

colnames(Y_Ser)[7] <- "Sm Rnp"
colnames(Y_Ser)[8] <- "C4"
colnames(Y_Ser)[10] <- "RPP0"
colnames(Y_Ser)[14] <- "Mito Antigen"
colnames(Y_Ser)[15] <- "IF"
colnames(Y_Ser)[17] <- "DNA POLB"
colnames(Y_Ser)[18] <- "U1snRNP"
colnames(Y_Ser)[18] <- "U1snRNP A"
hits <- colnames(Y_Ser)[1:(ncol(Y_Ser)-2)]

for (i in 1:length(hits)){
   setwd("~/Documents/3_Parkinsons_disease/Autoantibody_Data/Tear_Autoantibodies_2021/")
   filename <- paste(hits[i],"__Serum_IgG.tiff", sep="")
   p <- ggplot(Y_Ser, aes(x=Strain, y=(Y_Ser[,i]), fill=Strain)) +
      geom_boxplot(outlier.shape = NA, width = 0.35, coef=1, varwidth=F, show.legend =
T, size=0.75) +
      geom_jitter(color = "darkgray", alpha =0.7, size=2.1, show.legend = F, position=po
sition_jitterdodge(0.55))+
      scale_color_manual(values=c("black", "navy")) +
      theme_minimal() +
      chart_design +
      ylab("Log 2 Norm Intensity")  +
      labs(title=colnames(Y_Ser[i]), hjust=0.5) +
      #scale_x_discrete("Biofluid", labels=c("Tear", "Serum")) +
      scale_fill_jco() #+ ylim(5.5,28)
      #scale_y_continuous(label=scientific_10)
   tiff(filename, units="in", width=2.35, height=3.2, res=300)
   print(p)
   dev.off()
}
```

Boxplots facetted

```
#facetted plot
library(tidyr)
Y_Ser_facet <- gather(Y_Ser, key="Autoantigen", value = "Norm_Intensity", 1:18)
Y_Ser_facet$Autoantigen <- factor(Y_Ser_facet$Autoantigen)
for (i in 1:3) {
   p <- ggplot(Y_Ser_facet[,], aes(x=Strain, y=(Norm_Intensity), fill=Strain)) +
      geom_boxplot(outlier.shape = NA, width = 0.35, coef=1, varwidth=F, show.legend =
T, size=0.75) +
      geom_jitter(color = "darkgray", alpha =0.7, size=2.1, show.legend = F, position=po
sition_jitterdodge(0.55))+
      scale_color_manual(values=c("black", "navy")) +
      theme_minimal() +
      chart_design +
      ylab("Log Norm Intensity")  +
   facet_wrap(facets=Y_Ser_facet$Autoantigen, nrow=3, ncol=6, scales="free") +
      labs(title=colnames(Y_Ser[i]), hjust=0.5) +
      #scale_x_discrete("Biofluid", labels=c("Tear", "Serum")) +
      scale_fill_jco()
   print(p)
}
```

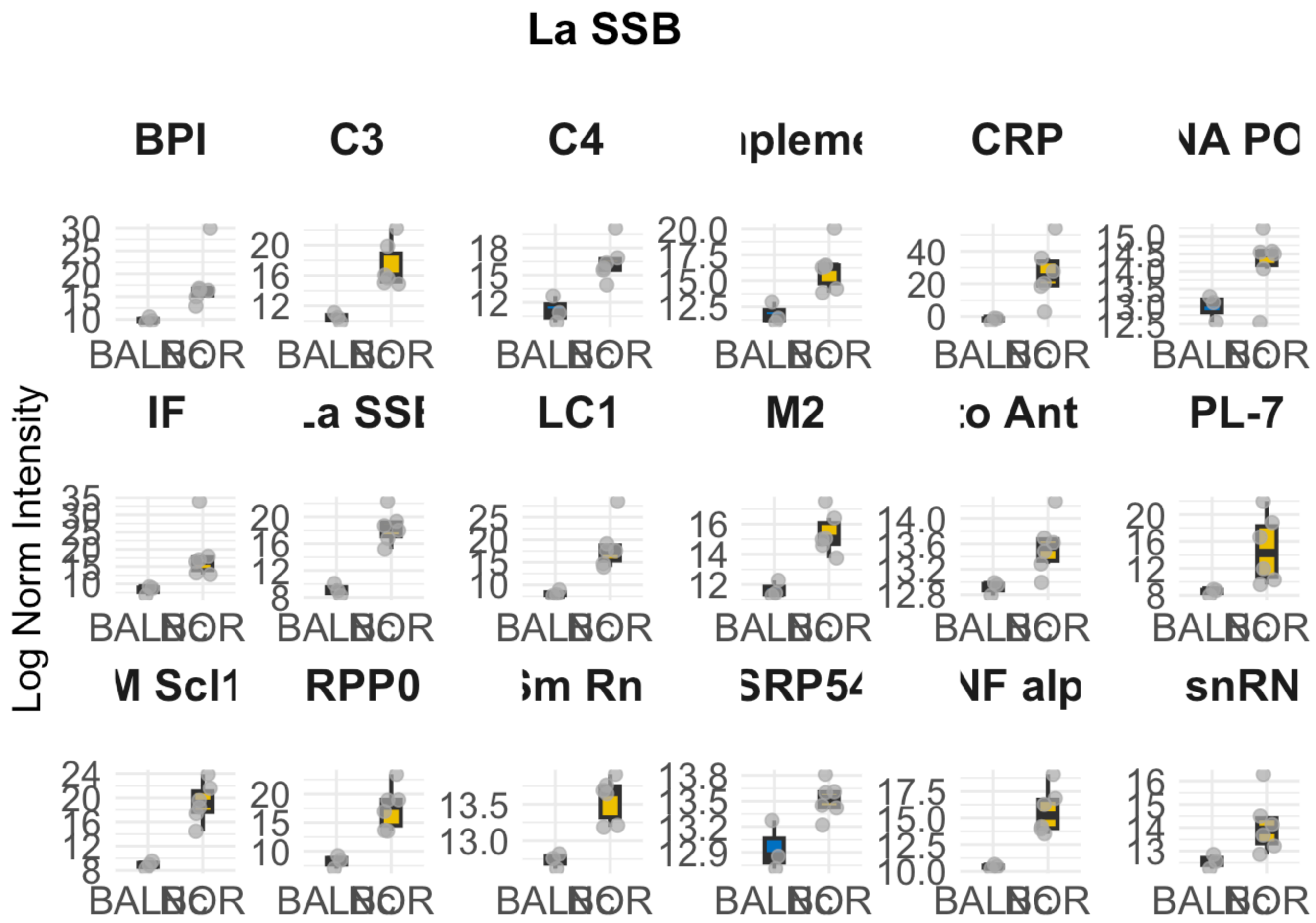
La SSB
BPI
C3
C4
CRP
IF
LC1
M2
PL-7
RPP0
snRN
Log Norm Intensity
BAL
NOR

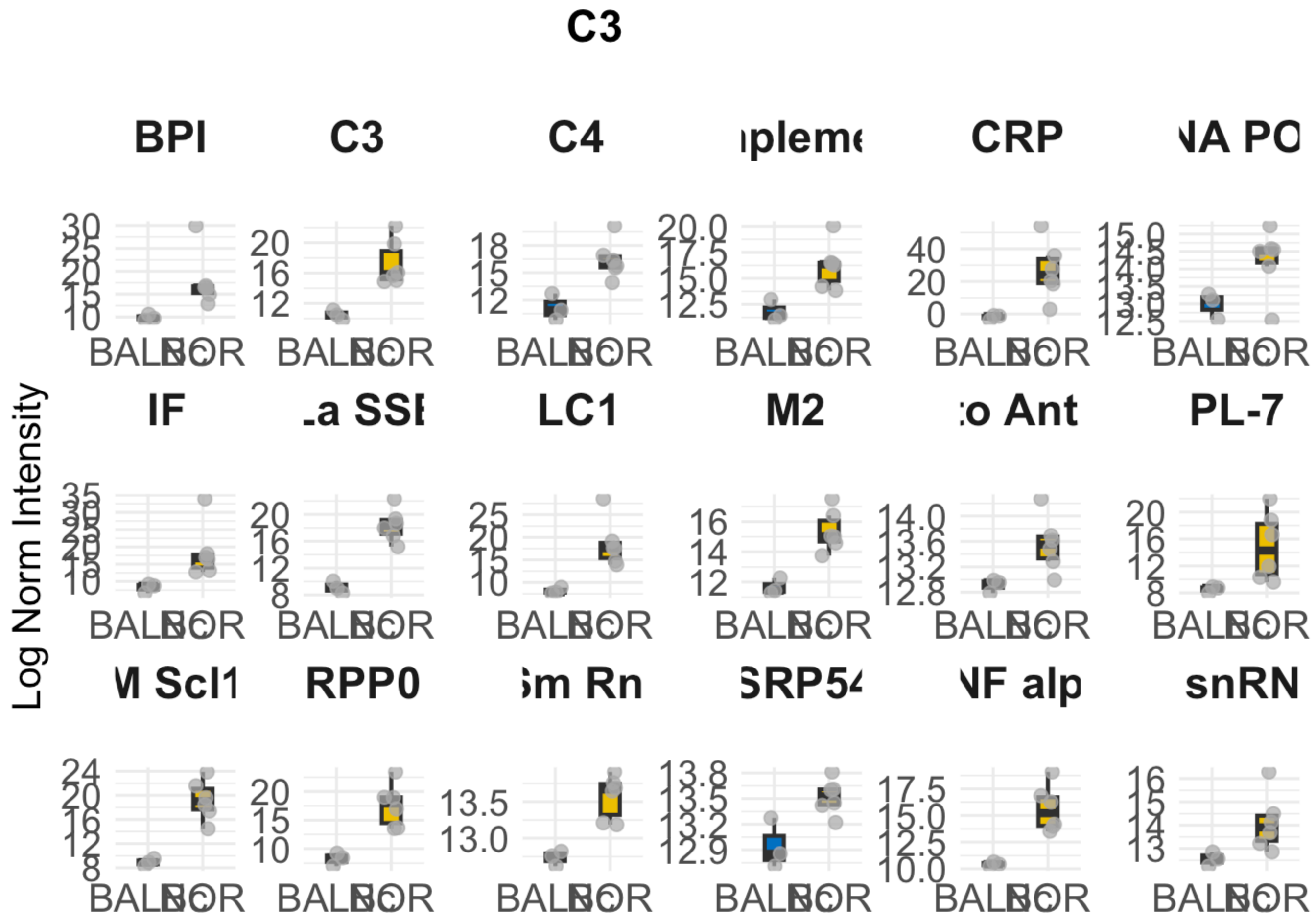
C3
BPI
C3
C4
CRP
IF
LC1
M2
PL-7
RPP0
snRN
Log Norm Intensity
BAL
NOR

TNF alpha

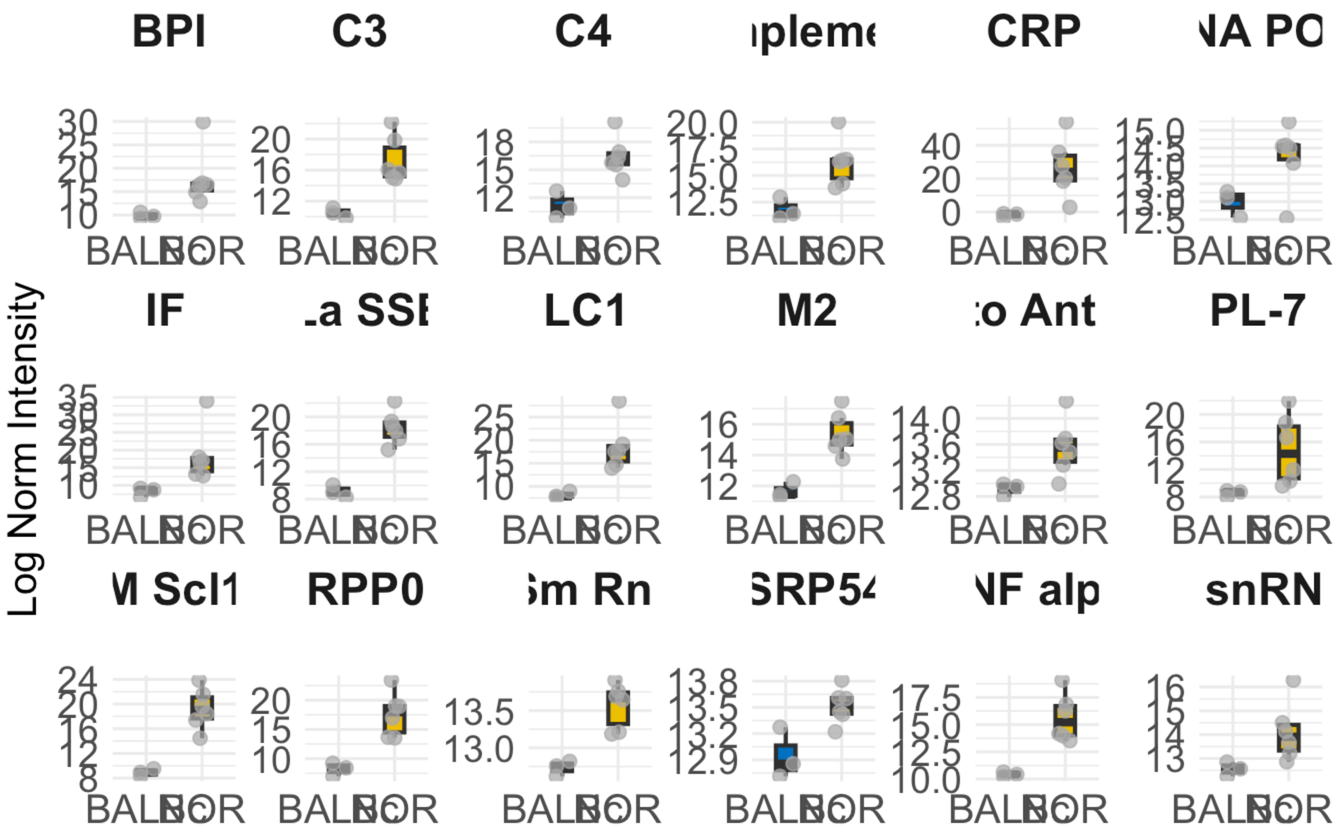

Code for Boxplots of tears hits
