# Supplementary material for: Serum and tear autoantibodies from NOD and NOR mice as potential diagnostic indicators of local and systemic inflammation in Sjögren’s disease
Source: Front Immunol. 2025 Jan 28;15:1516330. doi: 10.3389/fimmu.2024.1516330 (PMC11810956; doi:10.3389/fimmu.2024.1516330)
Supplement: Supplementary file 4 [file DataSheet4.pdf]

Supplemental Methods 1.3

Tear IgG Autoantibodies - NOD & NOR mice

Shruti Singh Kakan

2024-12-11

- QC Plots
- Voom Normalization After Removing outliers (BALB/c M6)
    - Voom Normalized Results - Table 1
    - Boxplots from voom normalized counts Figure 3A

```
knitr::opts_chunk$set(
    echo = TRUE,
    message = FALSE,
    warning = FALSE,
    root.dir = '~/Documents/3_Parkinsons_disease/Autoantibody_Data/Tear_Auto_Validation_
2022/'
)

setwd('~/Documents/3_Parkinsons_disease/Autoantibody_Data/Tear_Auto_Validation_2022/')
IgG_NSI <- read.csv("IgG_MCF_SSK_546_Tear_NSI.csv",  header=T, nrows=80)[1:16]#, row.nam
es = 1)
IgG_SNR <- read.csv("IgG_MCF_SSK_546_Tear_SNR.csv",  header=T, nrows=80)[1:16]#, row.nam
es = 1)
Strain <- c(rep("NOD", each=5),rep("NOR", each=3), rep("BALBc", each=6))
```

#Adding Auto-antigen id names

```
#Adding Auto-antigen id names
Antigen_ID <- read.csv("~/Documents/3_Parkinsons_disease/Autoantibody_Data/Tear_Auto_Val
idation_2022/Antigen_ID.csv",  header=T)[1:80,1:2]

#colnames(IgA_NSI)[1] <- colnames(Antigen_ID)[2]
#colnames(IgA_SNR)[1] <- colnames(Antigen_ID)[2]

Antigen_ID[68,"ID"] <- IgG_NSI[68,"ID"]
IgG_NSI <- full_join(IgG_NSI, Antigen_ID, by="ID")
IgG_SNR <- full_join(IgG_SNR, Antigen_ID, by="ID")
rownames(IgG_NSI) <- IgG_NSI$Antigen_ID
rownames(IgG_SNR) <- IgG_NSI$Antigen_ID

IgG_NSI <- IgG_NSI[,-c(1, 17)]
IgG_SNR <- IgG_SNR[,-c(1, 17)]
```

```
####Setting up column metadata

Strain <- c(rep("NOD", each=5),rep("NOR", each=4), rep("BALBc", each=6))
colData <- as.data.frame(cbind(c(colnames(IgG_NSI[,1:15])), Strain))
colnames(colData) <- c('Sample', "Strain")
rownames(colData) <- colData$Sample
colData$Strain <- factor(colData$Strain)
#colData$Strain <- relevel(colData$Strain, ref = "BALBc")
#Biofluid <- c( rep("Tear", each=11), rep("Serum", each=11))

#colData <- as.data.frame(cbind(c(colnames(IgA_NSI)), Strain, Biofluid))
```

Filtering Data based on low Signal to Noise ratio

```
IgG_raw=list()
IgG_SNR$average <- apply(cbind(rowMeans(as.matrix(IgG_SNR)[,1:9]), rowMeans(as.matrix(Ig
G_SNR)[,10:15])), 1, max)
IgG_SNR$med <- apply(cbind(rowMedians(as.matrix(IgG_SNR)[,1:9]), rowMedians(as.matrix(Ig
G_SNR)[,10:15])), 1, max)

#IgG_raw$NSI <- as.matrix(IgG_NSI[which(IgG_SNR$med>2),1:15])
#IgG_raw$SNR <- as.matrix(IgG_SNR[which(IgG_SNR$med>2),1:15])[,1:15]

IgG_raw$NSI <- as.matrix(IgG_NSI[which(rowSums(IgG_SNR[,1:15]>2.8) > 6),])
IgG_raw$SNR <- as.matrix(IgG_SNR[which(rowSums(IgG_SNR[,1:15]>2.8) > 6),][,1:15])
```

Visualizing Filtered Data

```
dataN <- log2(IgG_NSI[,1:15]  + 0.5)
countData = as.data.frame(dataN)

df_dseq = melt(countData, variable.name = "Samples", value.name = "count")# reshape the
matrix
mycolors <- colorRampPalette(brewer.pal(8, "Set1"))(15)

ggplot(df_dseq, aes(x = count, color=Samples)) +
   geom_density(alpha = 0.5, size = 0.8) +
   #facet_wrap(~Strain, ncol=2) +
   theme_minimal() + #xlim(-1.5,6) +
   scale_colour_manual(values=mycolors, name="") +
   guides(fill="none")
```

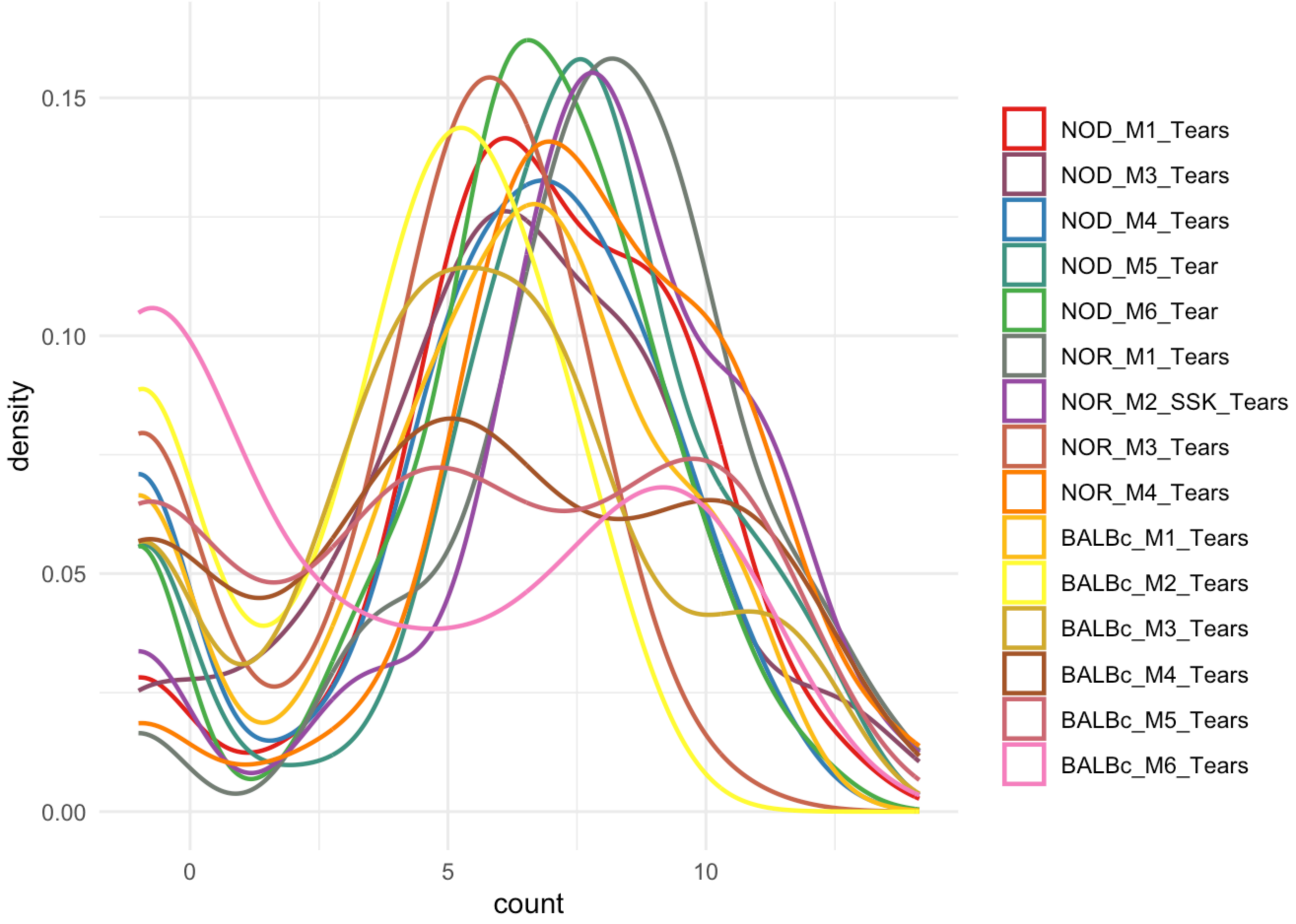


```
boxplot(as.data.frame(dataN),main="IgG normalization")#,col=Sample)
```

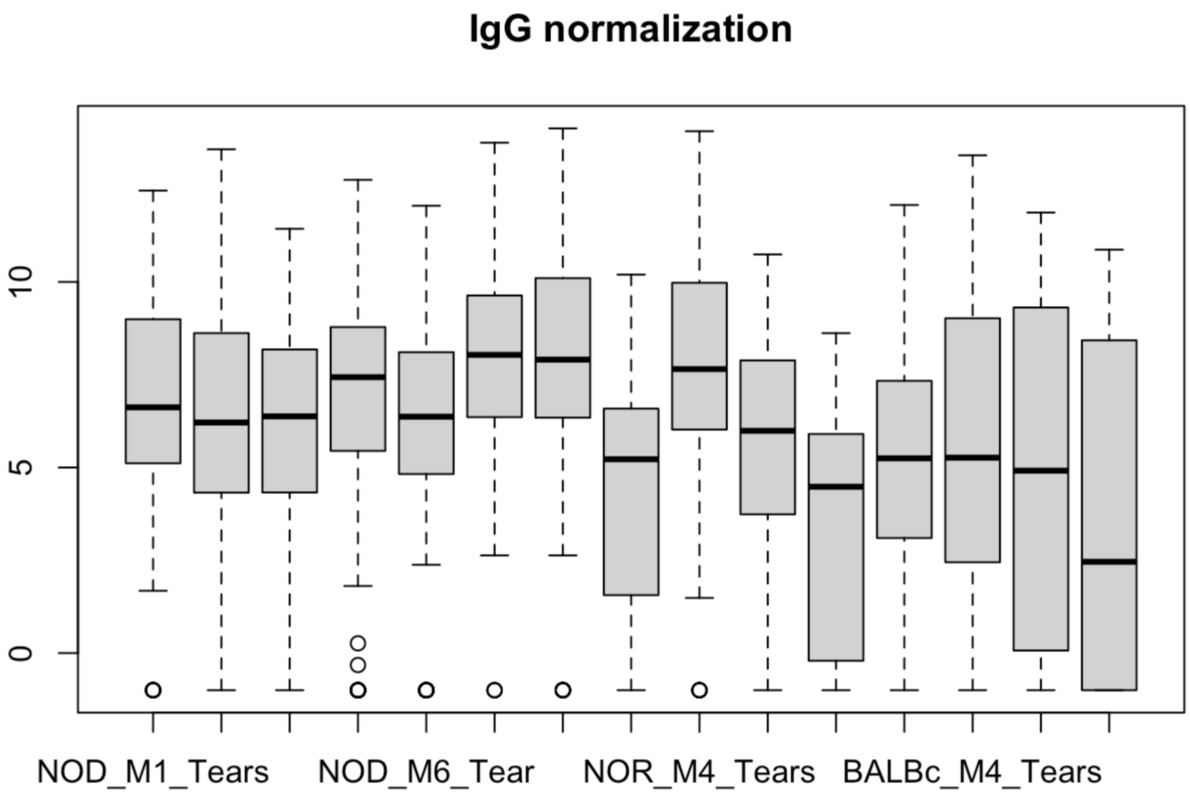


```
dataN <- (IgG_NSI)[,1:15]
countData = as.data.frame(dataN)
boxplot(as.data.frame(dataN),main="NSI")
```

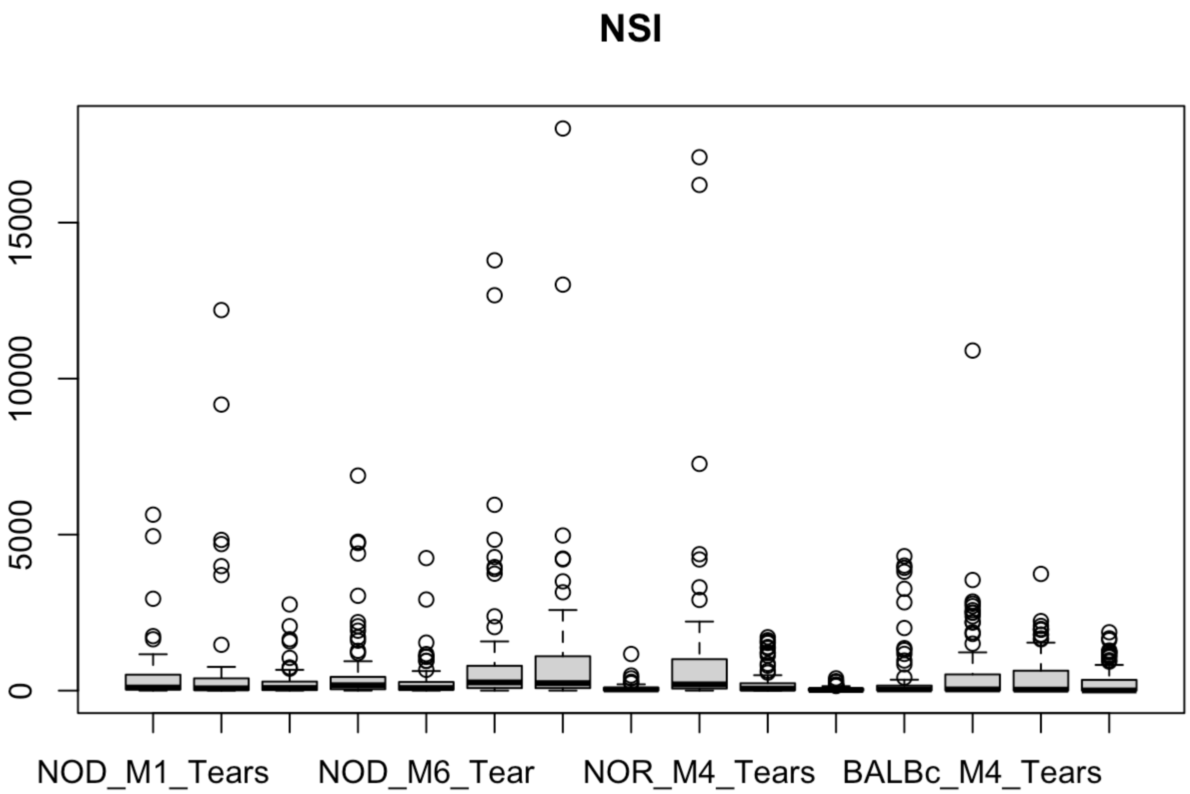


```
dataN <- log2(IgG_raw$NSI +0.5)
countData = as.data.frame(dataN)
boxplot(as.data.frame(dataN),main="Log2 NSI")
```

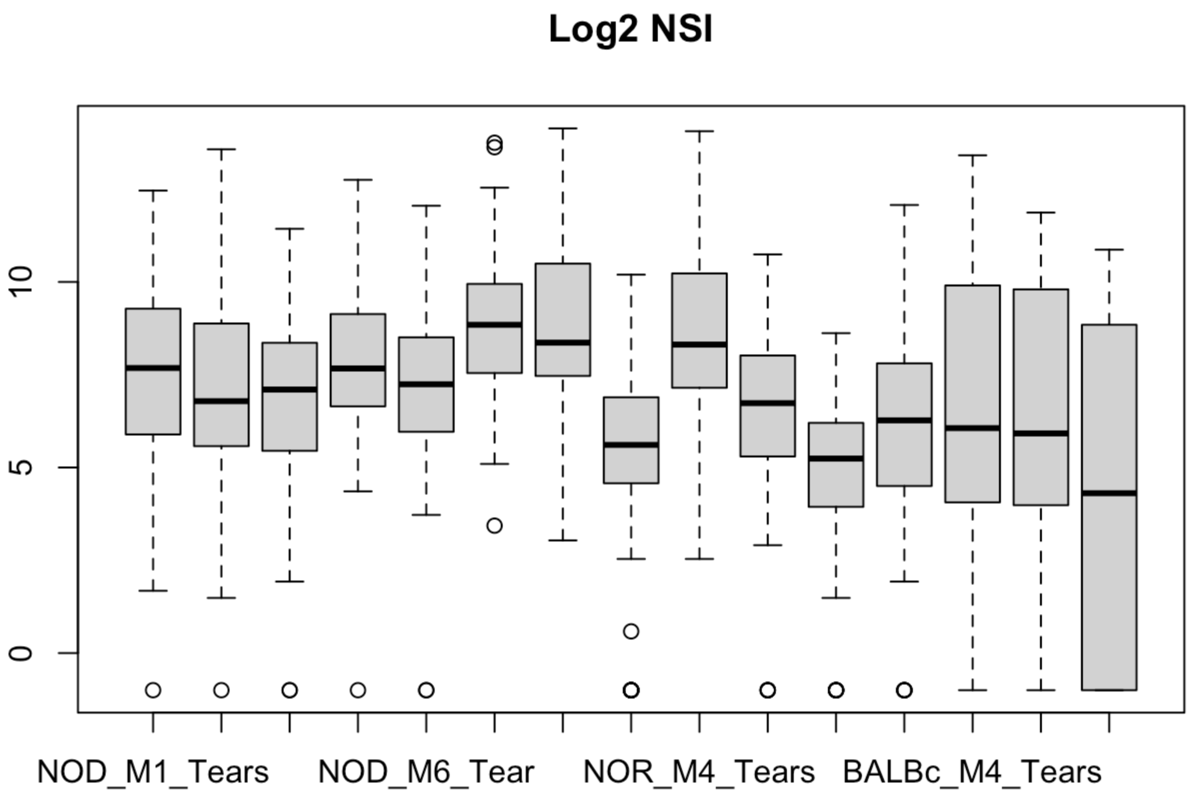

#Limma Based Quantile Normalization

```
#Removing male BALB/c 6
library(limma)
dataN <- log2(IgG_raw$NSI[,]*10000/colSums(IgG_raw$NSI[,]) + 1)
mydata <- as.matrix(dataN)

conditions<- paste(colData$Strain[],sep=".")
conditions <- factor(conditions, levels=unique(conditions))
design <- model.matrix(~0+ conditions)
colnames(design) <- levels(conditions)
fit <- lmFit(mydata, design)

cont.matrix<- makeContrasts(
   NTvBT =  NOD - BALBc,
   nTvBT = NOR - BALBc,
   levels = design)
fit.cont<- contrasts.fit(fit, cont.matrix)
fit.cont<- eBayes(fit.cont)

NTvsBT <- topTable(fit.cont, coef=1, p.value=1, number=45, adjust.method = 'BH')
nTvsBT <- topTable(fit.cont, coef=2, p.value=1, number=45, adjust.method = 'BH')
NTvsBT$Antigen <- row.names(NTvsBT)
nTvsBT$Antigen <- row.names(nTvsBT)
NTvnTvBT <- full_join(NTvsBT, nTvsBT, by="Antigen", suffix = c(".NOD", ".NOR"))
rownames(NTvnTvBT) <- NTvnTvBT$Antigen

which(NTvnTvBT$adj.P.Val.NOD > 0.05 & NTvnTvBT$adj.P.Val.NOR > 0.05)
```

```
## [1] 26 27 34 35 36 38 41 42 44
```

```
NTvnTvBT[is.na(NTvnTvBT)] <- 0.5
NTvnTvBT <- NTvnTvBT[c(which(NTvnTvBT$adj.P.Val.NOD < 0.05 & NTvnTvBT$adj.P.Val.NOR < 0.
05)),]

knitr::kable(NTvnTvBT[which(NTvnTvBT$logFC.NOD>1),c(1:2,5,8,12)])
```

	logFC.NOD	AveExpr.NOD	adj.P.Val.NOD	logFC.NOR	adj.P.Val.NOR
PM Scl75	6.290114	3.633837	0.0002240	5.284447	0.0015327
SAE1 SAE2	6.691624	4.338235	0.0002524	4.699777	0.0057872
IA-2	5.332941	5.336901	0.0011405	5.145079	0.0024400
Mi-2	5.916087	5.021786	0.0011405	6.841943	0.0008666
TPO	4.470946	6.254731	0.0035264	4.335345	0.0047366
tTG	4.423625	5.277892	0.0037859	4.329721	0.0047366
SmD1	5.350090	5.038448	0.0046969	5.067537	0.0073797
Jo-1	4.738576	7.877154	0.0079395	5.140714	0.0047366

	logFC.NOD	AveExpr.NOD	adj.P.Val.NOD	logFC.NOR	adj.P.Val.NOR
GP210	4.596590	3.763182	0.0154286	6.451313	0.0020896
GAD65	3.678729	6.746115	0.0154286	3.118074	0.0395742
Gliadin	3.755070	5.554156	0.0154286	4.214085	0.0079606
KS	3.297850	4.357408	0.0168054	5.057959	0.0015327
Cardiolipin	3.417464	4.407645	0.0264553	4.725097	0.0047366
KU P70P80	2.702332	4.483986	0.0333116	3.412002	0.0120393
BPI	2.800616	3.560657	0.0357401	4.660285	0.0024728
PL-7	3.017324	5.640085	0.0357407	3.382809	0.0286036
IF	2.792100	4.551336	0.0463934	4.786661	0.0031683

QC Plots

```
plot.new()
#plotMDS(fit.cont,col=as.numeric(Strain))
plotMD(fit.cont, col=2)
```

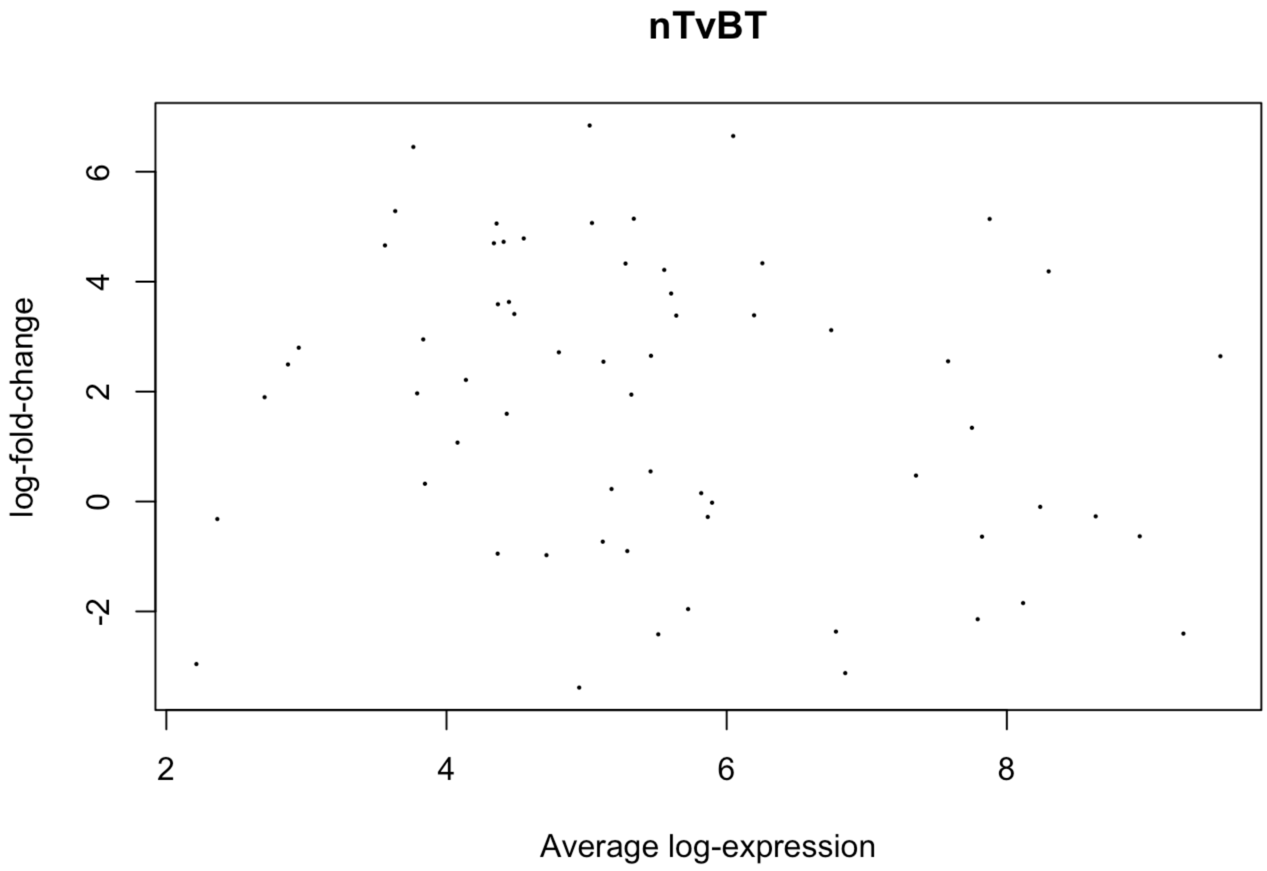

```
qqt(fit.cont$t,df=fit.cont$df.prior+fit.cont$df.residual)
```

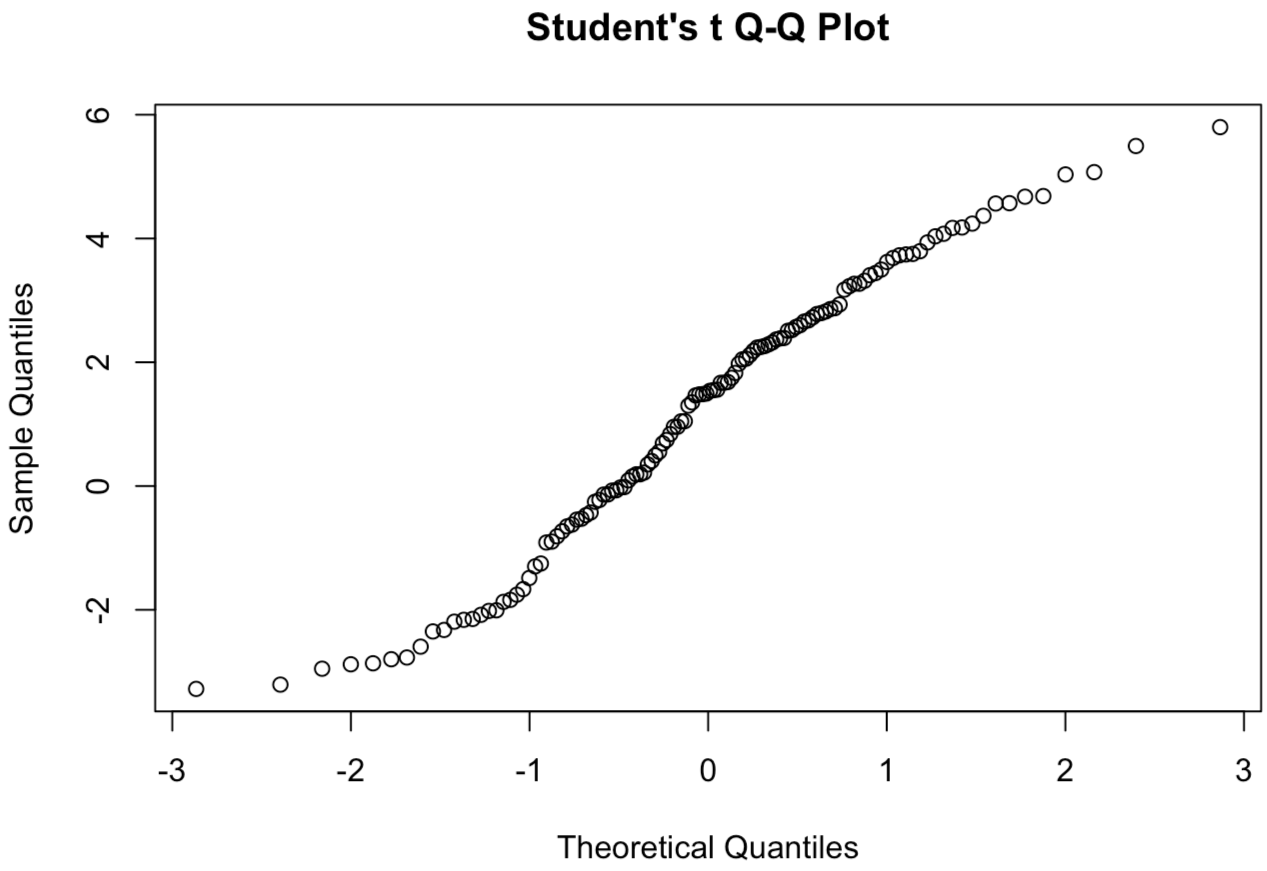

Voom Normalization After Removing outliers (BALB/c M6)

```
#After removing outlier samples

###### Voom normalization with quantiles
conditions<- paste(colData$Strain[],sep=".")
conditions <- factor(conditions, levels=unique(conditions))
design <- model.matrix(~0+ conditions)
colnames(design) <- levels(conditions)

v <- voom(counts=(IgG_raw$NSI[,]*IgG_raw$SNR[,]), design, plot=TRUE, normalize="quantil
e")
```

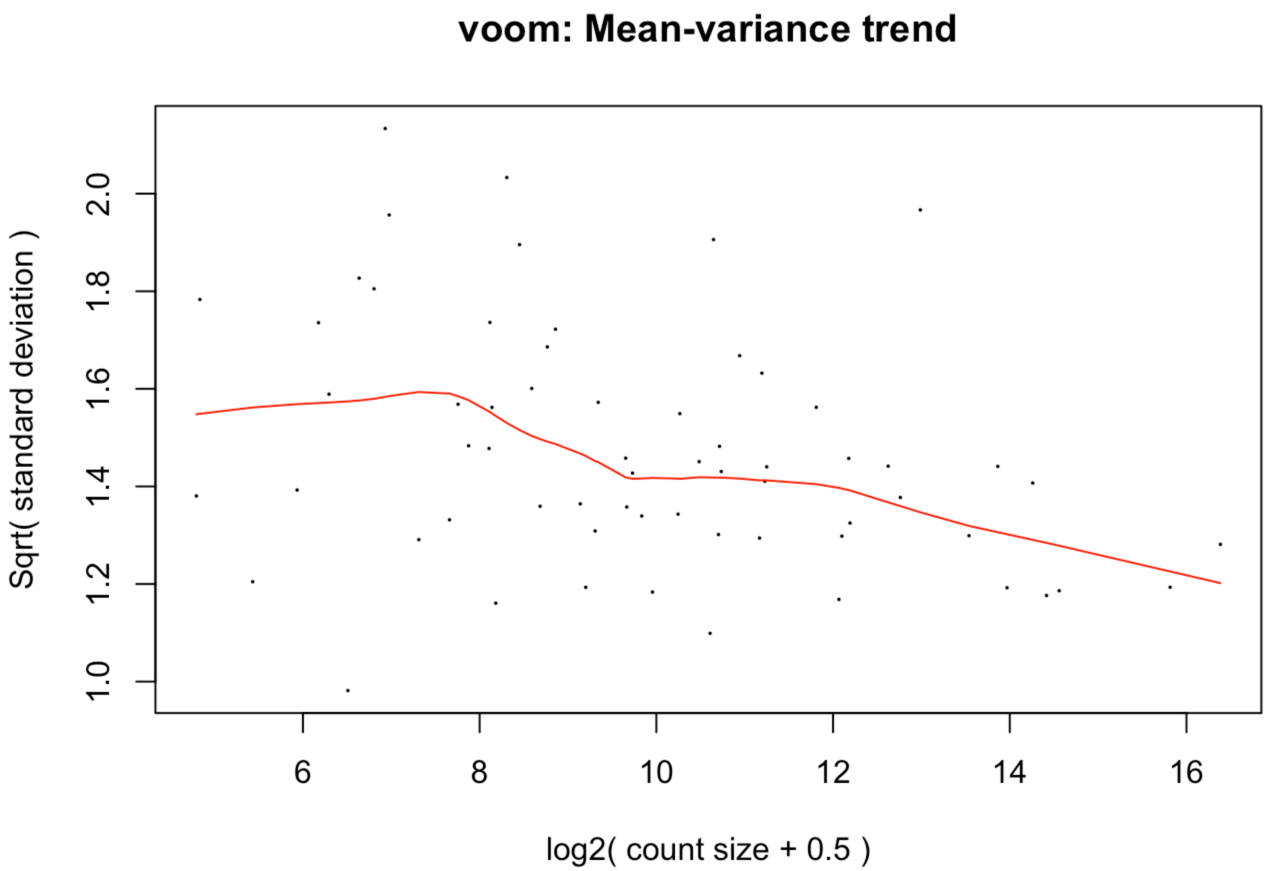


```
#v <- voom(dae, design, plot=TRUE)
fit <- lmFit(v, design)
fit.cont<- contrasts.fit(fit, cont.matrix)
fit.cont<- eBayes(fit.cont, robust=TRUE)
topTable(fit.cont)
```

```
##                NTvBT      nTvBT   AveExpr        F      P.Value    adj.P.Val
## Scl70      -7.421154  -6.483344 12.125990 31.84984 3.886532e-07 1.842418e-05
## CytC       -8.084894  -8.790225  4.820094 30.25334 5.848947e-07 1.842418e-05
## B2GP1      -8.389948  -5.821087 12.649254 26.50292 1.632745e-06 3.428764e-05
## MBP        -6.384337  -5.235675 10.634185 25.31323 2.310943e-06 3.639736e-05
## COL3       -8.919112 -10.503421  8.884504 24.67459 2.973068e-06 3.746065e-05
## SRP54      -5.181210  -6.849610  9.982099 23.34225 4.217624e-06 4.428505e-05
## snRNP C    -6.687267  -9.553305  8.142212 19.04683 1.783326e-05 1.604993e-04
## SAE1 SAE2   9.206879   4.558506  9.368146 17.18070 3.716178e-05 2.926490e-04
## IA-2        6.812489   6.184083 10.740183 16.60217 4.460320e-05 3.122224e-04
## COL5       -4.241697  -4.956964 12.092494 16.11891 5.398600e-05 3.401118e-04
```

```
#topTable(fit, coef=ncol(design))
```

Voom Normalized Results - Table 1

```
NTvsBT <- topTable(fit.cont, coef=1, p.value=1, number=55, adjust.method = 'BH')
nTvsBT <- topTable(fit.cont, coef=2, p.value=1, number=55, adjust.method = 'BH')
NTvsBT$Antigen <- row.names(NTvsBT)
nTvsBT$Antigen <- row.names(nTvsBT)
NTvnTvBT <- full_join(NTvsBT, nTvsBT, by="Antigen", suffix = c(".NOD", ".NOR"))
rownames(NTvnTvBT) <- NTvnTvBT$Antigen

NTvnTvBT[is.na(NTvnTvBT)] <- 0.5
NTvnTvBT <- NTvnTvBT[c(which(NTvnTvBT$adj.P.Val.NOD < 0.18 & NTvnTvBT$adj.P.Val.NOR < 0.
18 & NTvnTvBT$logFC.NOD>0.5)),]
#write.csv(NTvnTvBT, file="NTvnTvBT_Tear_IgG.csv", sep=',')

knitr::kable(NTvnTvBT[which(NTvnTvBT$logFC.NOD>0.5),c(1:2,5,8,12)])
```

	logFC.NOD	AveExpr.NOD	adj.P.Val.NOD	logFC.NOR	adj.P.Val.NOR
SAE1 SAE2	9.206879	9.368146	0.0000995	4.558506	0.0469918
IA-2	6.812489	10.740183	0.0002546	6.184083	0.0012583
Jo-1	5.774805	12.787246	0.0007273	5.163181	0.0023470
tTG	5.403999	11.254272	0.0007534	5.138353	0.0019353
TPO	5.581821	12.203745	0.0007632	4.297296	0.0106901
SmD1	5.836973	7.781770	0.0024958	3.667343	0.0591562
GAD65	4.118464	11.275201	0.0074892	2.806177	0.0727650
Mi-2	5.174936	8.615684	0.0082790	5.539206	0.0073212
gDNA	2.987238	13.994425	0.0116446	3.329558	0.0077535
PM Scl75	5.727549	6.661913	0.0158259	4.503572	0.0626973
PL-7	3.192643	10.760423	0.0298835	2.625342	0.0861977
Gliadin	3.905719	11.220371	0.0536445	3.835417	0.0591562
Cardiolipin	3.841207	8.792060	0.0797793	5.872399	0.0073212
GP210	4.437515	7.002317	0.0926538	7.672371	0.0053073
GP2	2.224255	13.564362	0.0901207	4.271604	0.0019526
KS	2.709501	8.132424	0.1420426	4.566656	0.0131207
T1F1 g	3.612743	8.333134	0.1704457	3.811570	0.1486130

```
Result_IgG <- as.data.frame(
   cbind(rownames(NTvnTvBT),
         round(NTvnTvBT$adj.P.Val.NOD, 6),
         paste0(round(NTvnTvBT$logFC.NOD, 2),
                " (",
                (round(2^NTvnTvBT$logFC.NOD, 2)),
                ")"),
         round(NTvnTvBT$adj.P.Val.NOR, 6),
         paste0(round(NTvnTvBT$logFC.NOR, 2),
                " (",
                (round(2^NTvnTvBT$logFC.NOR, 2)),
                ")")
         )
   )
Result_IgG
```

```
##              V1       V2             V3       V4            V5
## 1   SAE1 SAE2     1e-04 9.21 (590.94) 0.046992  4.56 (23.56)
## 2          IA-2 0.000255  6.81 (112.4) 0.001258  6.18 (72.71)
## 3          Jo-1 0.000727  5.77 (54.75) 0.002347  5.16 (35.83)
## 4           tTG 0.000753   5.4 (42.34) 0.001935  5.14 (35.22)
## 5           TPO 0.000763   5.58 (47.9)  0.01069   4.3 (19.66)
## 6          SmD1 0.002496  5.84 (57.16) 0.059156  3.67 (12.71)
## 7         GAD65 0.007489  4.12 (17.37) 0.072765   2.81 (6.99)
## 8          Mi-2 0.008279  5.17 (36.13) 0.007321   5.54 (46.5)
## 9          gDNA 0.011645   2.99 (7.93) 0.007753  3.33 (10.05)
## 10    PM Scl75 0.015826  5.73 (52.99) 0.062697   4.5 (22.68)
## 11         PL-7 0.029884   3.19 (9.14) 0.086198   2.63 (6.17)
## 12      Gliadin 0.053644  3.91 (14.99) 0.059156  3.84 (14.27)
## 13 Cardiolipin 0.079779  3.84 (14.33) 0.007321  5.87 (58.58)
## 14        GP210 0.092654  4.44 (21.67) 0.005307 7.67 (203.99)
## 15          GP2 0.090121   2.22 (4.67) 0.001953  4.27 (19.31)
## 16           KS 0.142043   2.71 (6.54) 0.013121   4.57 (23.7)
## 17       T1F1 g 0.170446  3.61 (12.23) 0.148613  3.81 (14.04)
```

```
setwd("~/Documents/3_Parkinsons_disease/Autoantibody_Data/Tear_Auto_Validation_2022/")
colnames(Result_IgG) <- c("Antigen", "p adj NOD", "Log2FC (FC) NOD", "p adj NOR", "Log2F
C (FC) NOR")
write.csv(Result_IgG, file="Results_Tear_IgG.csv", sep=',')
```

```
plotMDS(v,col=as.numeric(Strain))
```

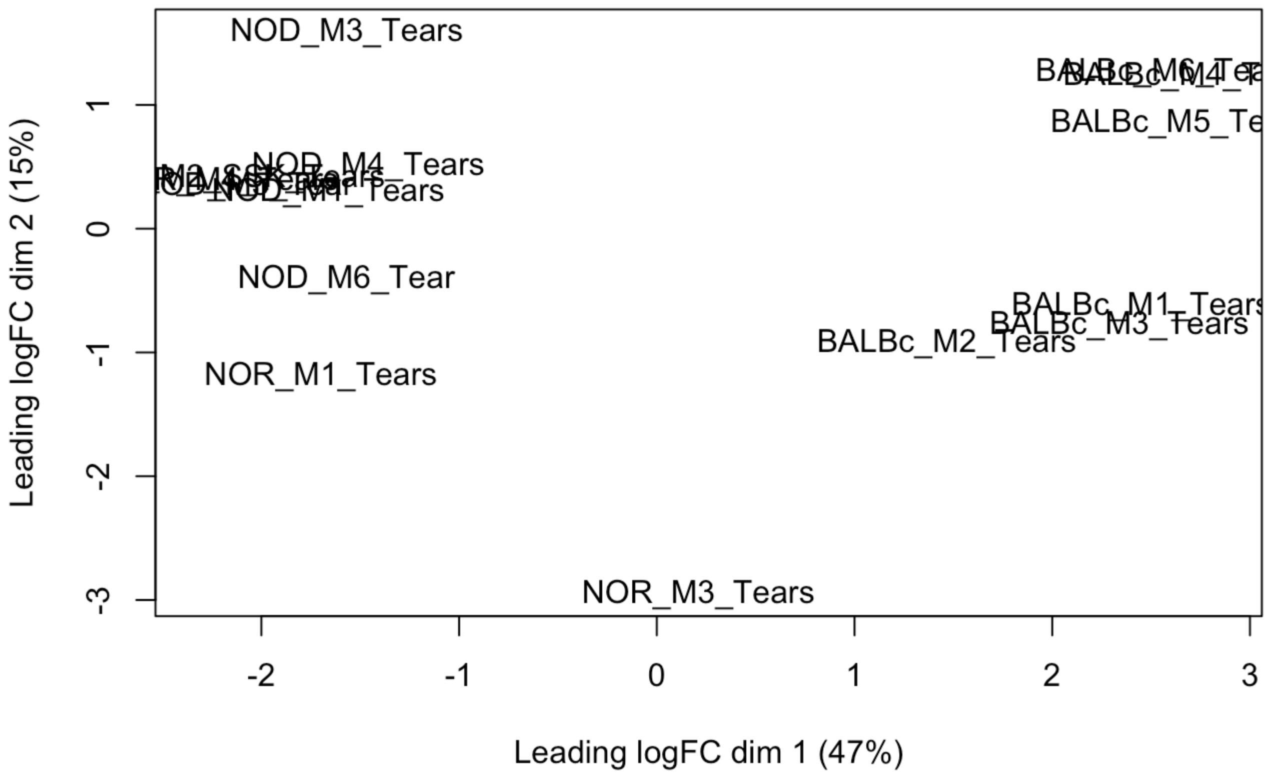


```
#plot.new()
#plotMD(fit.cont, col=2)
#plotMD(fit.cont, col=1)
qqt(fit.cont$t,df=fit.cont$df.prior+fit.cont$df.residual)
```

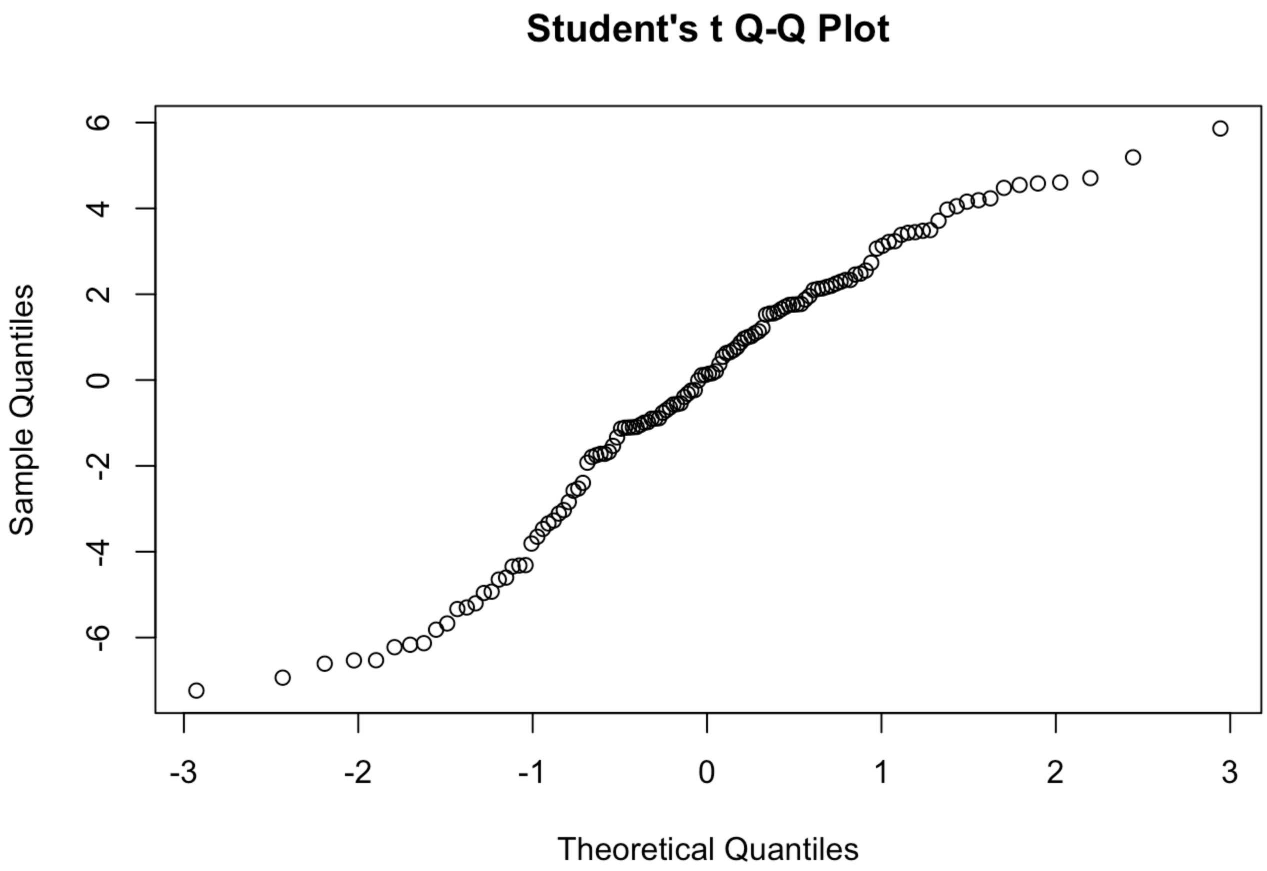


```
plotDensities(v, group=Strain, col=c("orange","green", "blue"), log=TRUE)
```

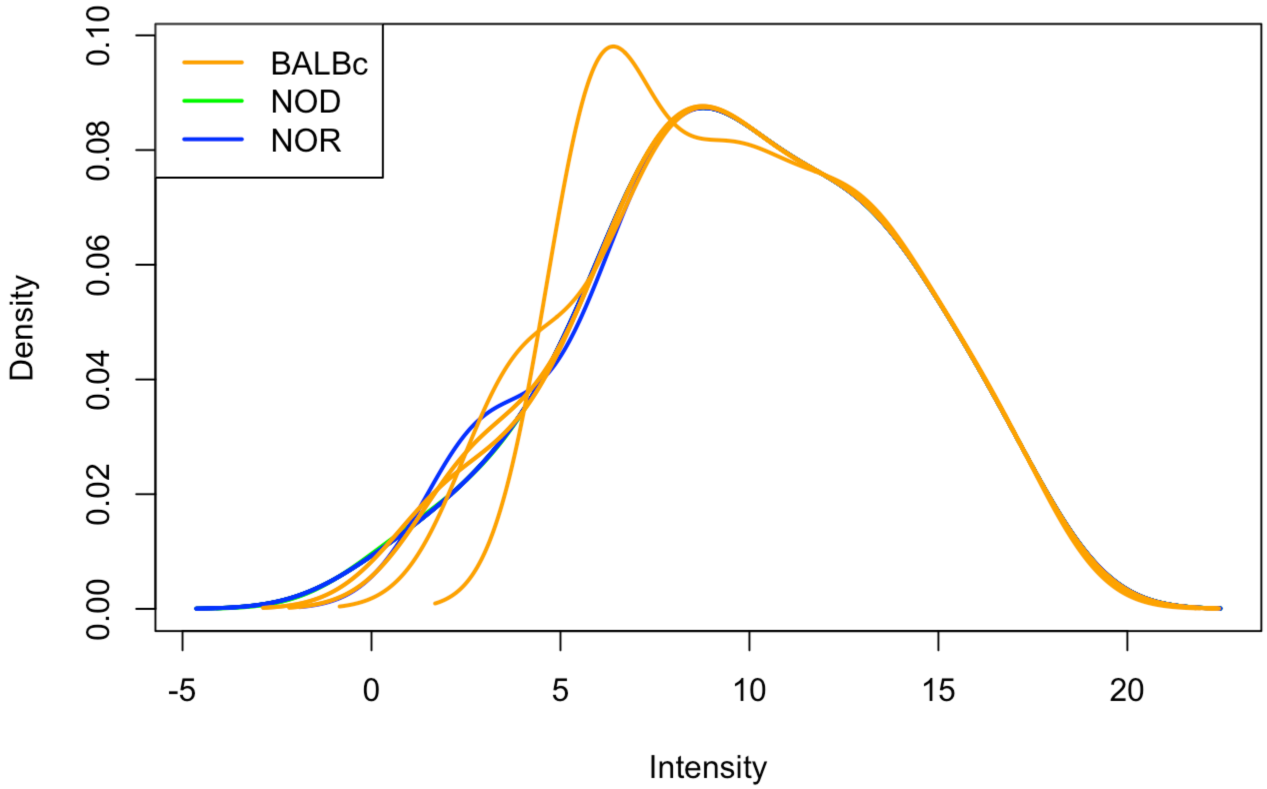
BALBc
NOD
NOR
Density
0.00
0.02
0.04
0.06
0.08
0.10
-5
0
5
10
15
20
Intensity

```
#Boxplots

#Density plots

dae <- DGEList(counts=(IgG_raw$NSI+0.5))
keep <- filterByExpr(dae, design)
dae <- dae[keep,,keep.lib.sizes=FALSE]
dae <- calcNormFactors(dae)
logCPM <- cpm(dae, log=TRUE, prior.count=2)

dataN <- list()
dataN[["Before"]]<- as.data.frame(log2(IgG_raw$NSI[,1:15]  + 1))
dataN[["After_Normalization"]] <- as.data.frame(v$E)

for (i in c("Before", "After_Normalization")) {
   df_dseq = melt(dataN[[i]], variable.name = "Samples", value.name = "count")
   #df_dseq$Strain <- factor(substr(df_dseq$Var2, 1,3))
   mycolors <- colorRampPalette(brewer.pal(8,"Set1"))(15)
   p <- ggplot(df_dseq, aes(x = count, color=Samples)) +
      geom_density(alpha = 0.5, size = 0.8) +
      #facet_wrap(~Strain, ncol=2) +
      theme_minimal() + xlim(-5, 25) +
      scale_colour_manual(values=mycolors, name="") +
   guides(fill="none")
   print(p)
   rm(df_dseq)
}
```

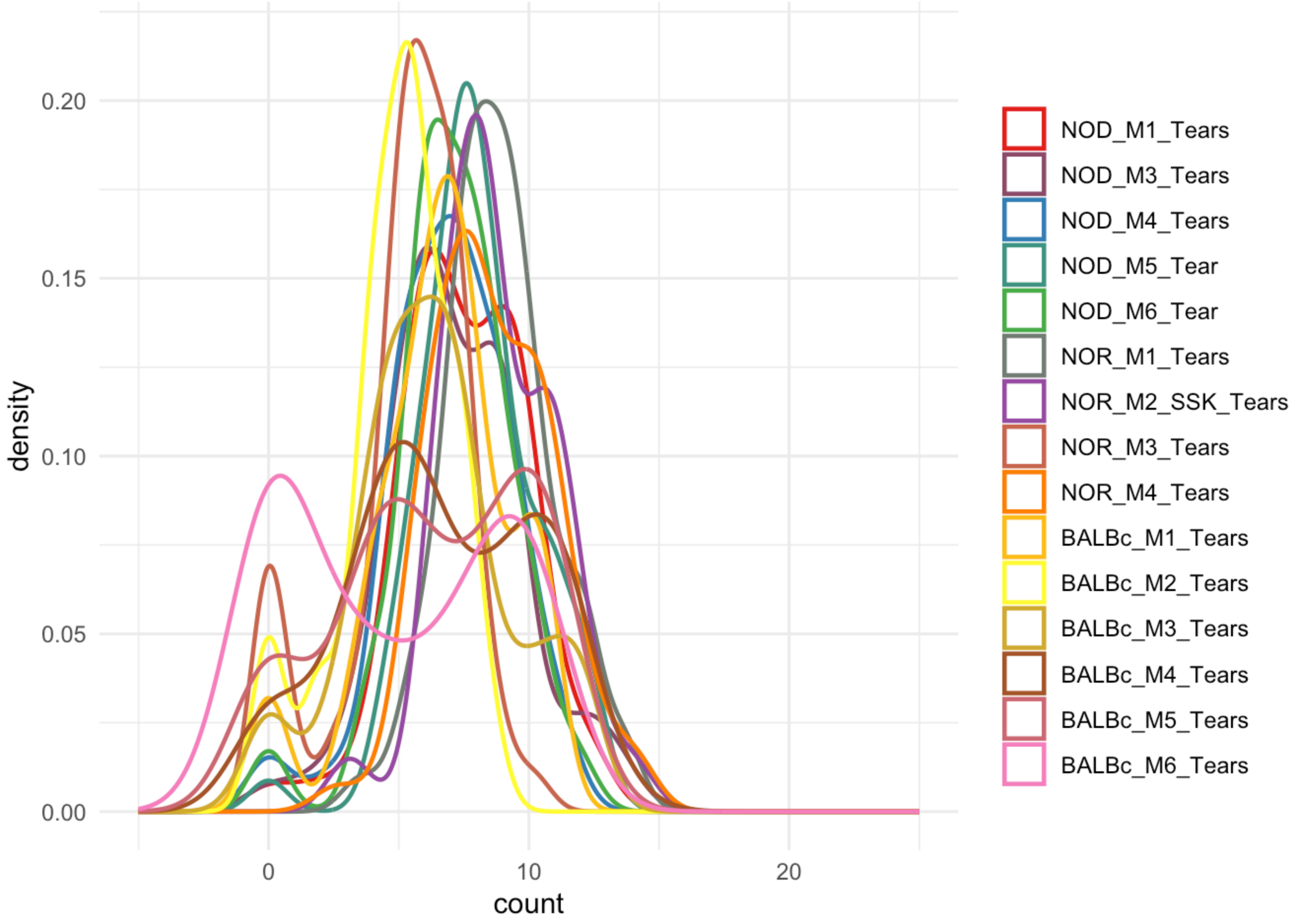
0.20
0.15
0.10
0.05
0.00
density
0
10
20
count
NOD_M1_Tears
NOD_M3_Tears
NOD_M4_Tears
NOD_M5_Tear
NOD_M6_Tear
NOR_M1_Tears
NOR_M2_SSK_Tears
NOR_M3_Tears
NOR_M4_Tears
BALBc_M1_Tears
BALBc_M2_Tears
BALBc_M3_Tears
BALBc_M4_Tears
BALBc_M5_Tears
BALBc_M6_Tears

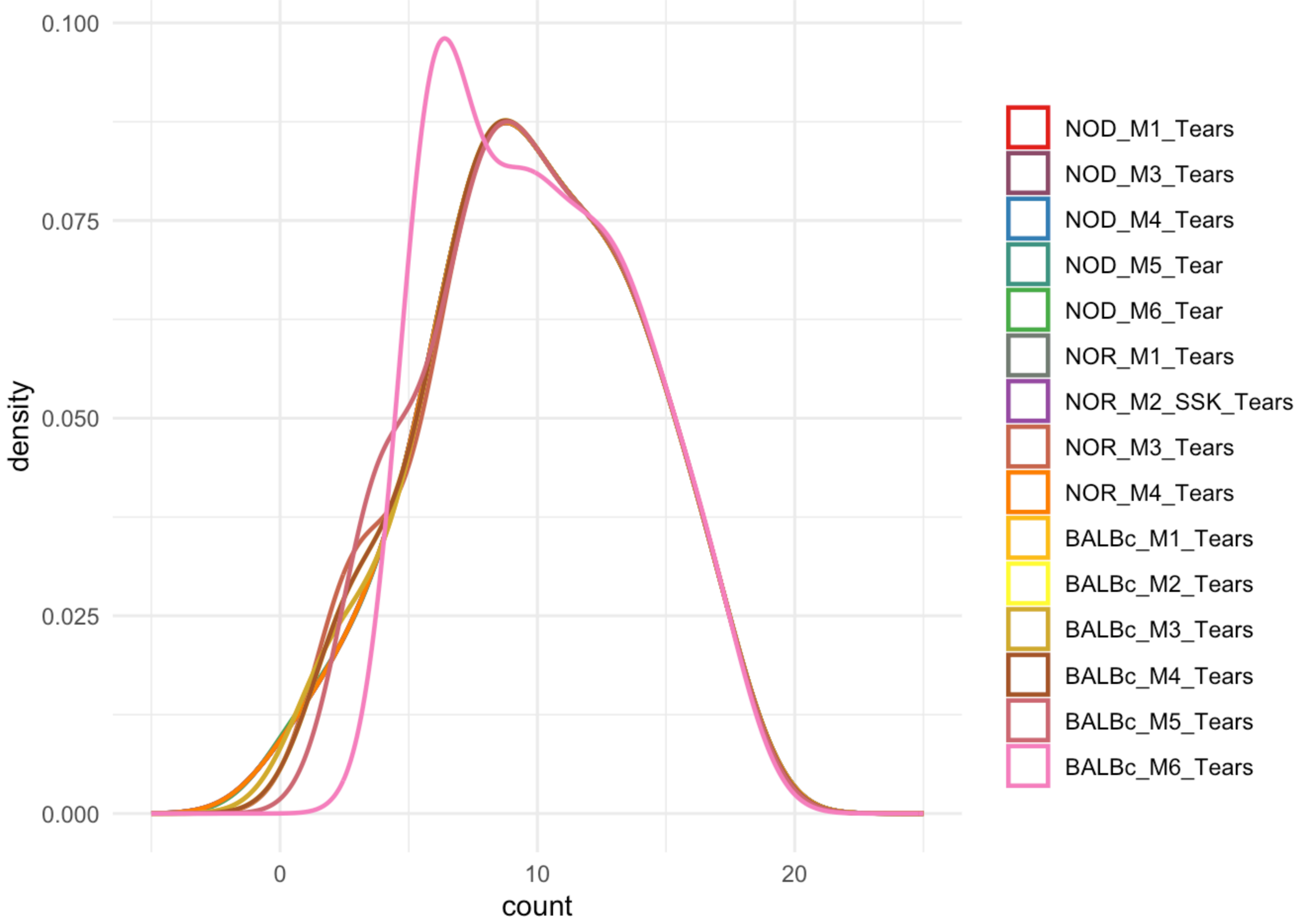
0.100
0.075
0.050
0.025
0.000
density
0
10
20
count
NOD_M1_Tears
NOD_M3_Tears
NOD_M4_Tears
NOD_M5_Tear
NOD_M6_Tear
NOR_M1_Tears
NOR_M2_SSK_Tears
NOR_M3_Tears
NOR_M4_Tears
BALBc_M1_Tears
BALBc_M2_Tears
BALBc_M3_Tears
BALBc_M4_Tears
BALBc_M5_Tears
BALBc_M6_Tears

```
#R-Squared ....... goodness of fit
for (i in 1:70){
   sst <- rowSums(v$E^2)
   ssr <- sst - fit.cont$df.residual*(fit.cont$sigma^2)
   Rsq<- (ssr/sst)
}
plot(1:nrow(IgG_raw$NSI), Rsq)
```

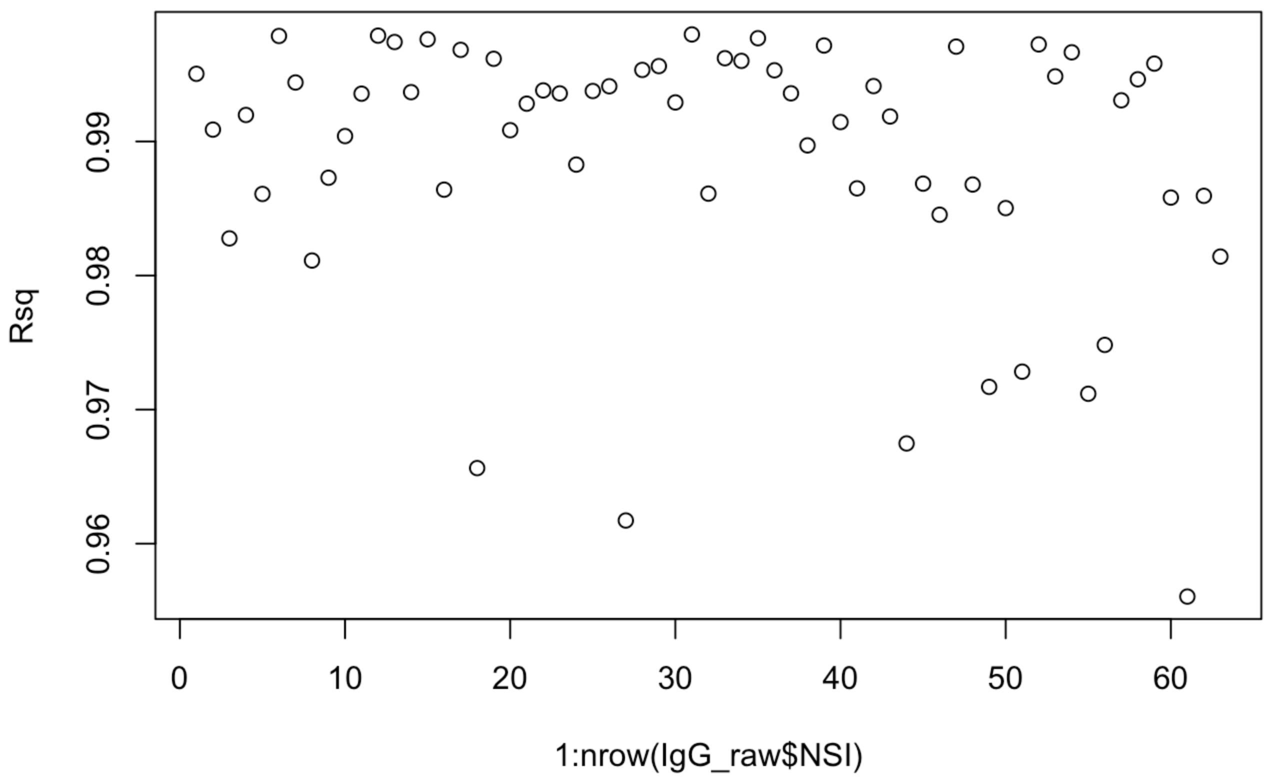


```
which(Rsq<0.90)
```

```
## named integer(0)
```

```
summary(fit.cont$r.squared)
```

```
## Length  Class   Mode
##      0   NULL   NULL
```

DE Analysis using voom normalized counts

Boxplots from voom normalized counts Figure 3A

```
chart_design <- theme(
   #plot.title = element_text(color = "Black", size = 16, face = "bold", margin = margin
(b=15), hjust=0.4),
   axis.text.x = element_text(size=15),
   axis.text.y = element_text(size=14),
   axis.title.x = element_blank(),
   legend.text = element_blank(),
   legend.title = element_blank(),
   legend.position = "right",
   axis.title.y = element_text(size=19, margin=margin(r=5)),
   strip.text.x = element_text(size=16, margin=margin(b=20), face='bold', hjust=0.4),
   strip.background = element_blank(),
   strip.placement = "outside")

mydata <- (v$E)

hits <- rownames(NTvnTvBT[c(which(NTvnTvBT$adj.P.Val.NOD < 0.05 & NTvnTvBT$adj.P.Val.NOR
< 0.05 & NTvnTvBT$logFC.NOD>3)),])

Y=matrix(nrow=length(hits),ncol=ncol(v$E))
for (i in 1:length(hits)) {
   Y[i,] <- mydata[hits[i],]
}
rownames(Y) <- hits
colnames(Y) <- colData$Sample[]

Y <- as.data.frame(t(Y))
Y$Strain <- colData$Strain[]
Y$Sample <- paste0(Y$Strain, c(1:5,1:4, 1:5))
setwd("~/Documents/3_Parkinsons_disease/Autoantibody_Data/Tear_Auto_Validation_2022/")
Y_combined <- Y
#Y_combined <- Y[,-which(colnames(Y) %in% c( "GAD65", "PM Scl75", "PL-7"))]

Y_combined <- gather(Y_combined, "Antigen", "V Counts", 1:(ncol(Y_combined)-2))

#tiff("Tear_IgG_hits.tiff", units="in", width=11.5, height=8, res=300)
ggplot(Y_combined, aes(x=Strain, y=`V Counts`, fill=Strain)) +
      geom_boxplot(outlier.shape = NA, width = 0.6, coef=1, varwidth=F, show.legend = T,
size=0.7, position = position_dodge(0.9)) +
      geom_jitter(color = "darkgray", alpha =0.7, size=2.2, show.legend = F, position =
position_jitterdodge(dodge.width=0.9))+
   facet_wrap(~Antigen, ncol=3) +
      theme_minimal() +
      chart_design + ylim(0,20) +
      ylab("Log Normalized Intensity")  +
      labs(title=colnames(Y[i]), hjust=0.5) +
      scale_fill_jco()
```

Mi-2

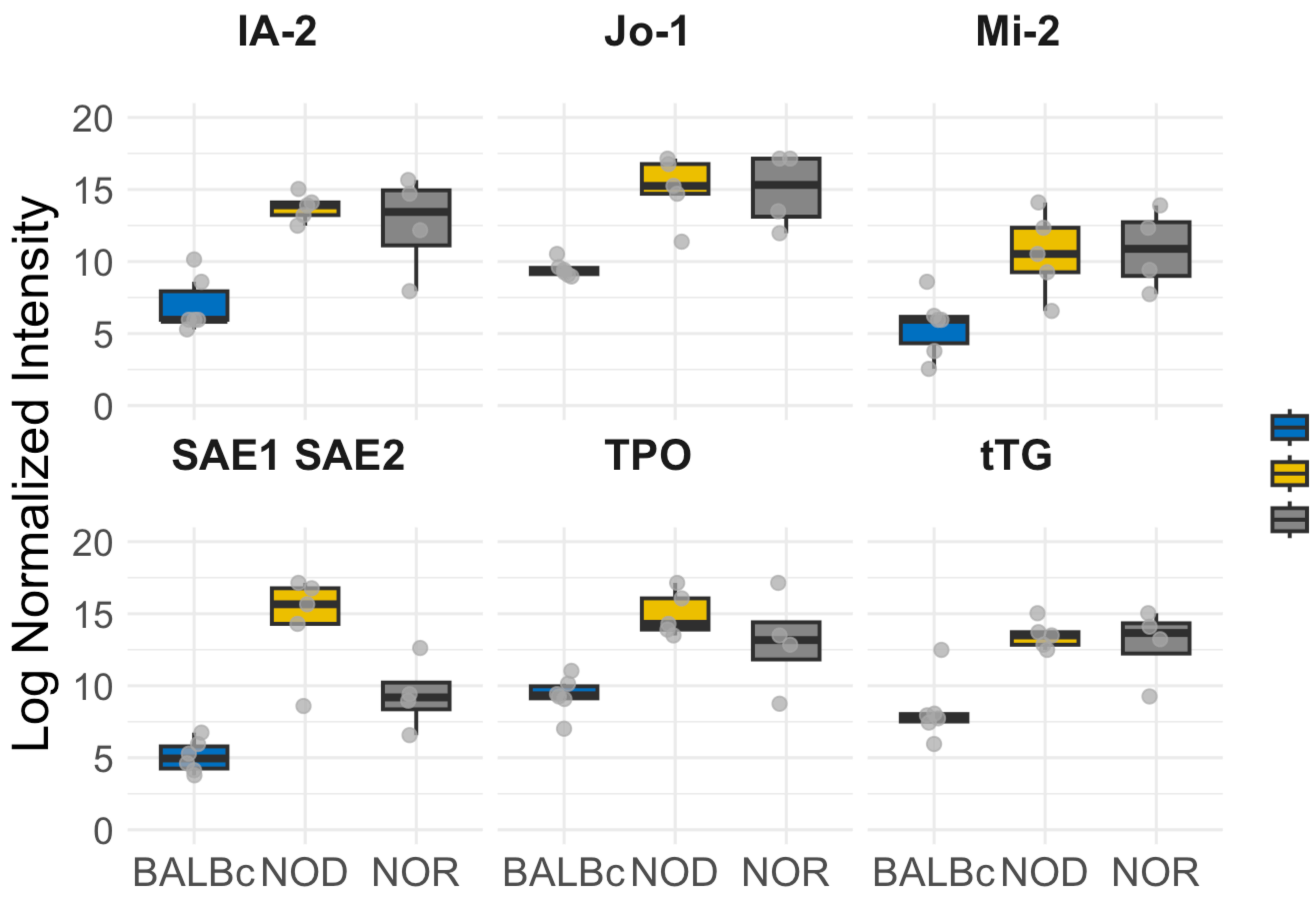
IA-2
Jo-1
Mi-2
SAE1 SAE2
TPO
tTG
Log Normalized Intensity
0
5
10
15
20
BALBc
NOD
NOR

```
#dev.off()
```
