# Supplementary material for: Serum and tear autoantibodies from NOD and NOR mice as potential diagnostic indicators of local and systemic inflammation in Sjögren’s disease
Source: Front Immunol. 2025 Jan 28;15:1516330. doi: 10.3389/fimmu.2024.1516330 (PMC11810956; doi:10.3389/fimmu.2024.1516330)
Supplement: Supplementary file 5 [file DataSheet5.pdf]

Supplemental Methods 1.4

Study 3 - Tear IgA Analysis

Shruti Singh Kakan

2025-01-10

- 1. Loading Libraries
- 2. Reading in Raw Tear Data
- 3. Filtering Data based on low Signal to Noise ratio
- 4. Limma Based Default Normalization & DE Analysis
- 5. Voom Normalization After Removing outliers
- 6. Plotting Differentially Expressed Autoantibodies
- 7. Code for Comparing Total Tear IgG and IgA Signal Intensity (ColSums)
- 8. Tear IgG vs IgA Correlation Analysis

1. Loading Libraries

```
knitr::opts_chunk$set(
    echo = TRUE,
    message = FALSE,
    warning = FALSE,
    root.dir = "~/Documents/3_Parkinsons_disease/Autoantibody_Data/Tear_Auto_Validation_
2022/"
)
library(car)
library(lsmeans)
library(calibrate)
library(dplyr)
#library(DEGreport)
library(DESeq2)
library(DEFormats)
library(edgeR)
library(ggpubr)
library(ggsci)
library(ggplot2)
library(gridExtra)
library(pheatmap)
library(reshape2)
library(RColorBrewer)
library(scales)
library(rstatix)
library(tidyr)
library(magrittr)
#library(PCAtools)
library(tidyverse)
library(Biobase)
#library(marray)
library(limma)
library(gplots)

library(devtools)
#install_github("dpgaile/AutoAntArrayExmpl")
library(AutoAntArrayExmpl)

library(devtools)
#install_github("dpgaile/AutoAntArrayExmpl")
#devtools::install_github('renozao/NMF@devel')
library(AutoAntArrayExmpl)
library(NMF)
library(quantreg)
library(asbio)
library(fdrtool)
#library(discreteMTP)
library(scales)
library(ggsci)
library(ggplot2)
```

2. Reading in Raw Tear Data

```
knitr::opts_chunk$set(
    echo = TRUE,
    message = FALSE,
    warning = FALSE,
    root.dir = '~/Documents/3_Parkinsons_disease/Autoantibody_Data/Tear_Auto_Validation_
2022/'
)

setwd('~/Documents/3_Parkinsons_disease/Autoantibody_Data/Tear_Auto_Validation_2022/')
#IgA_NSI <- read.csv("IgA_data/IgA_MCF_SSK_546_Tear_NSI_norm.csv",  header=T)[1:80,]
IgA_NSI <- read.csv("IgA_data/IgA_MCF_SSK_546_Tear_NSI.csv",  header=T)[1:80,]
IgA_SNR <- read.csv("IgA_data/IgA_MCF_SSK_546_Tear_SNR.csv",  header=T)[1:80,]
Strain <- c(rep("NOD", each=5),rep("NOR", each=3), rep("BALBc", each=6))
```

2.1 Adding Auto-antigen Id names

```
#Adding Auto-antigen id names
Antigen_ID <- read.csv("~/Documents/3_Parkinsons_disease/Autoantibody_Data/Tear_Auto_Val
idation_2022/Antigen_ID.csv",  header=T)[1:80,1:2]

#colnames(IgA_NSI)[1] <- colnames(Antigen_ID)[2]
#colnames(IgA_SNR)[1] <- colnames(Antigen_ID)[2]

Antigen_ID[68,"ID"] <- IgA_NSI[68,"ID"]
IgA_NSI <- full_join(IgA_NSI, Antigen_ID, by="ID")
IgA_SNR <- full_join(IgA_SNR, Antigen_ID, by="ID")
rownames(IgA_NSI) <- IgA_NSI$Antigen_ID
rownames(IgA_SNR) <- IgA_SNR$Antigen_ID

IgA_NSI <- IgA_NSI[,-c(1, 17)]
IgA_SNR <- IgA_SNR[,-c(1, 17)]
```

2.2 Setting up column Metadata

```
Strain <- c(rep("NOD", each=5),rep("NOR", each=4), rep("BALBc", each=6))

colData <- as.data.frame(cbind(c(colnames(IgA_NSI)), Strain))
colnames(colData) <- c('Sample', "Strain")
rownames(colData) <- colData$Sample
colData$Strain <- factor(colData$Strain)
#colData$Strain <- relevel(colData$Strain, ref = "BALBc")
#Biofluid <- c( rep("Tear", each=11), rep("Serum", each=11))

#colData <- as.data.frame(cbind(c(colnames(IgA_NSI)), Strain, Biofluid))
```

3. Filtering Data based on low Signal to Noise ratio

Rows with low signal to noise ratio of less than rowmeans 2.8 were removed. Additionally rows with low overall signal intensity were also removed.

```
IgA_raw=list()
IgA_SNR$average <- rowMeans(as.matrix(IgA_SNR))
IgA_SNR$med <- rowMedians(as.matrix(IgA_SNR))

IgA_raw$NSI <- as.matrix(IgA_NSI[which(IgA_SNR$average>2.8),])
IgA_raw$SNR <- as.matrix(IgA_SNR[which(IgA_SNR$average>2.8),])[,1:15]
#IgA_raw$NSI <- as.matrix(IgA_NSI[which(rowSums(IgA_SNR[,1:15]>2.8) > 6),])
#IgA_raw$SNR <- as.matrix(IgA_SNR[which(rowSums(IgA_SNR[,1:15]>2.8) > 6),][,1:15])

log2(colSums(IgA_raw$NSI+0.5))
```

```
##     NOD_M1_Tears     NOD_M3_Tears     NOD_M4_Tears      NOD_M5_Tear
##         15.58217         15.99049         14.43786         15.85415
##      NOD_M6_Tear     NOR_M1_Tears NOR_M2_SSK_Tears     NOR_M3_Tears
##         14.43494         16.98300         16.72778         12.24237
##     NOR_M4_Tears   BALBc_M1_Tears   BALBc_M2_Tears   BALBc_M3_Tears
##         16.70994         14.66195         12.77064         15.78438
##   BALBc_M4_Tears   BALBc_M5_Tears   BALBc_M6_Tears
##         16.33585         14.93757         13.78248
```

This reduced the total number of Autoantibodies (rows) included in the analysis from 80 to 69.

3.1 Visualizing Filtered Data

```
dataN <- log2(IgA_NSI + 0.5)
countData = as.data.frame(dataN)

df_dseq = melt(countData, variable.name = "Samples", value.name = "count")# reshape the
matrix

mycolors <- colorRampPalette(brewer.pal(8, "Set1"))(15)

ggplot(df_dseq, aes(x = count, color=Samples)) +
   geom_density(alpha = 0.5, size = 0.8) +
   #facet_wrap(~Strain, ncol=2) +
   theme_minimal() + #xlim(-1.5,6) +
   scale_colour_manual(values=mycolors, name="") +
   guides(fill="none")
```

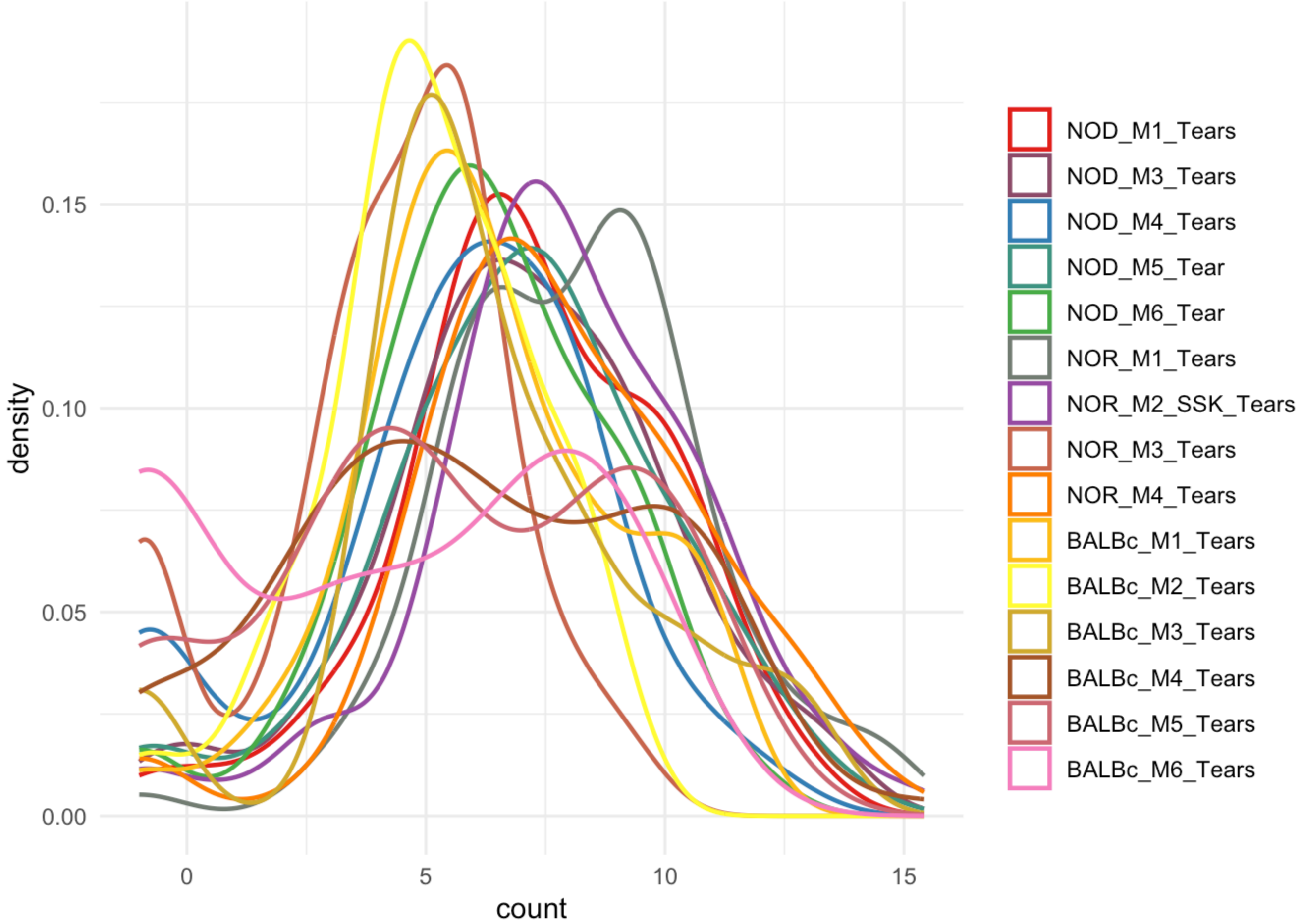

BALB/c Sample M6 appears to have extremely low signal intensity values across the board.

```
boxplot(as.data.frame((dataN)),main="Tear IgA Signal Intensity Prior to Row filtering")
#,col=Sample)
```

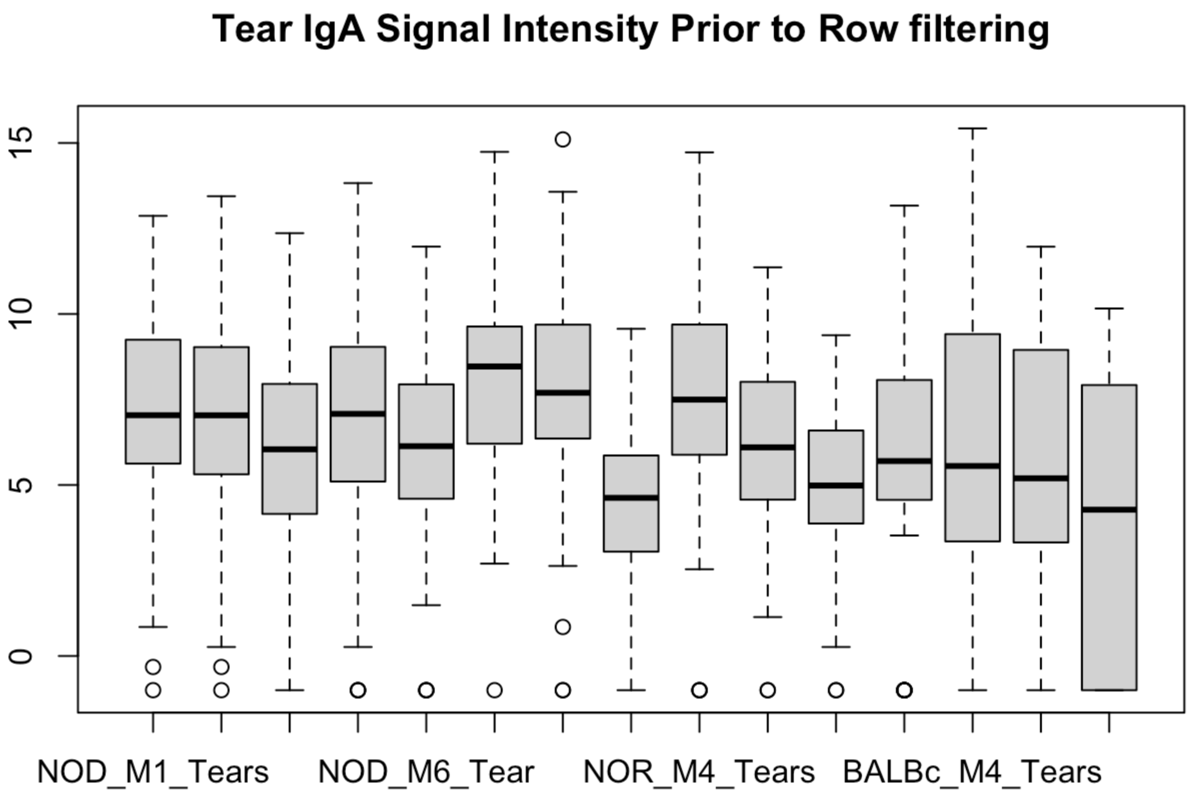


```
#dataN <- (IgA_NSI * IgA_SNR[,1:15] )
dataN <- (IgA_NSI)
countData = as.data.frame(dataN)
#boxplot(as.data.frame((dataN)),main="NSI")

#dataN <- log2(IgA_raw$NSI * IgA_raw$SNR +0.5)
dataN <- log2(IgA_raw$NSI+0.5)
countData = as.data.frame(dataN)
boxplot(as.data.frame((dataN)),main="Tear IgA Signal Intensity After Row filtering")
```

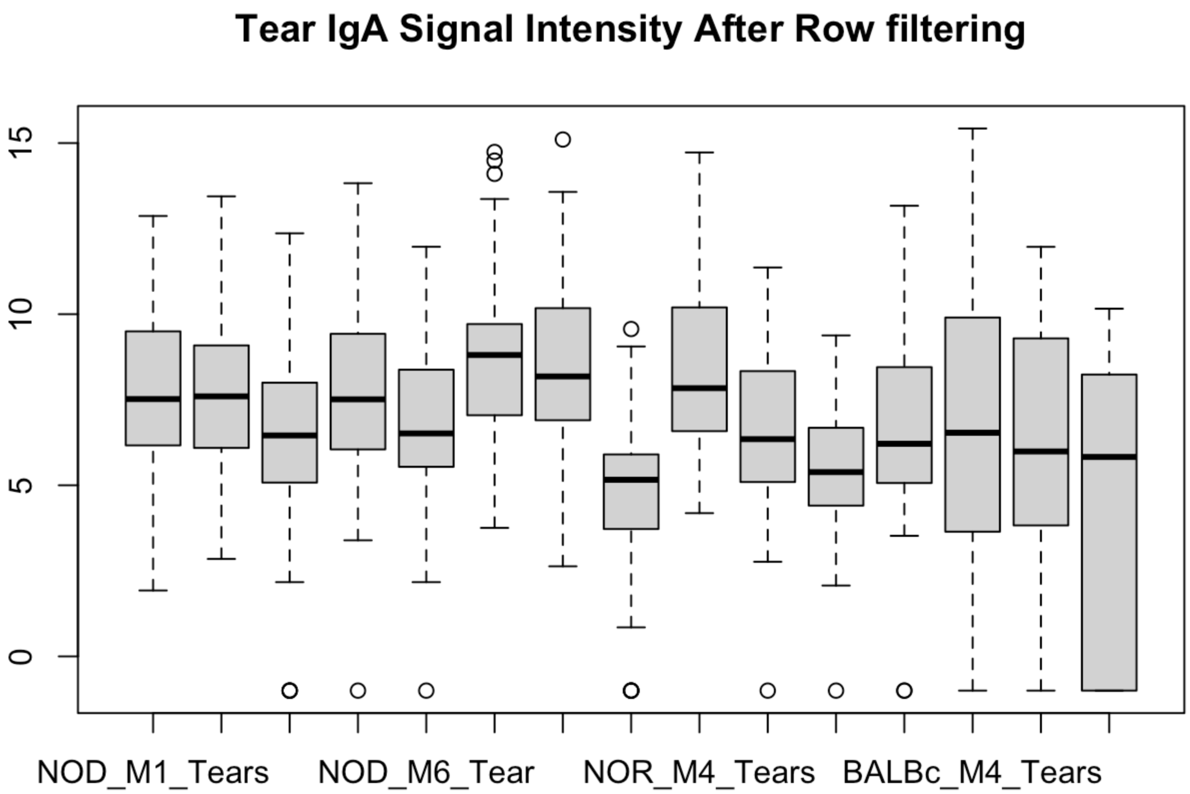

4. Limma Based Default Normalization & DE Analysis

Sample BALB/c M6 was excluded from normalization and differential expression analysis.

```
library(limma)
#dataN <- log2(IgA_raw$NSI * IgA_raw$SNR +0.5)[,-15]
dataN <- log2(IgA_raw$NSI +0.5)[,-15] #Sample BALB/c M6 removed
mydata <- as.matrix(dataN)

conditions<- paste(colData$Strain[-15],sep=".")
conditions <- factor(conditions, levels=unique(conditions))
design <- model.matrix(~0+ conditions)
colnames(design) <- levels(conditions)
fit <- lmFit(mydata, design)

cont.matrix<- makeContrasts(
   NTvBT =  NOD - BALBc,
   nTvBT = NOR - BALBc,
   levels = design)
fit.cont<- contrasts.fit(fit, cont.matrix)
fit.cont<- eBayes(fit.cont)
```

4.1 Default Limma Normalization QC

```
#MA plots showing relationship between average expression of autoantibodies and their lo
g fold−changes
plot.new()
plotMD(fit.cont, col=1) # NOD v BALB/c
```

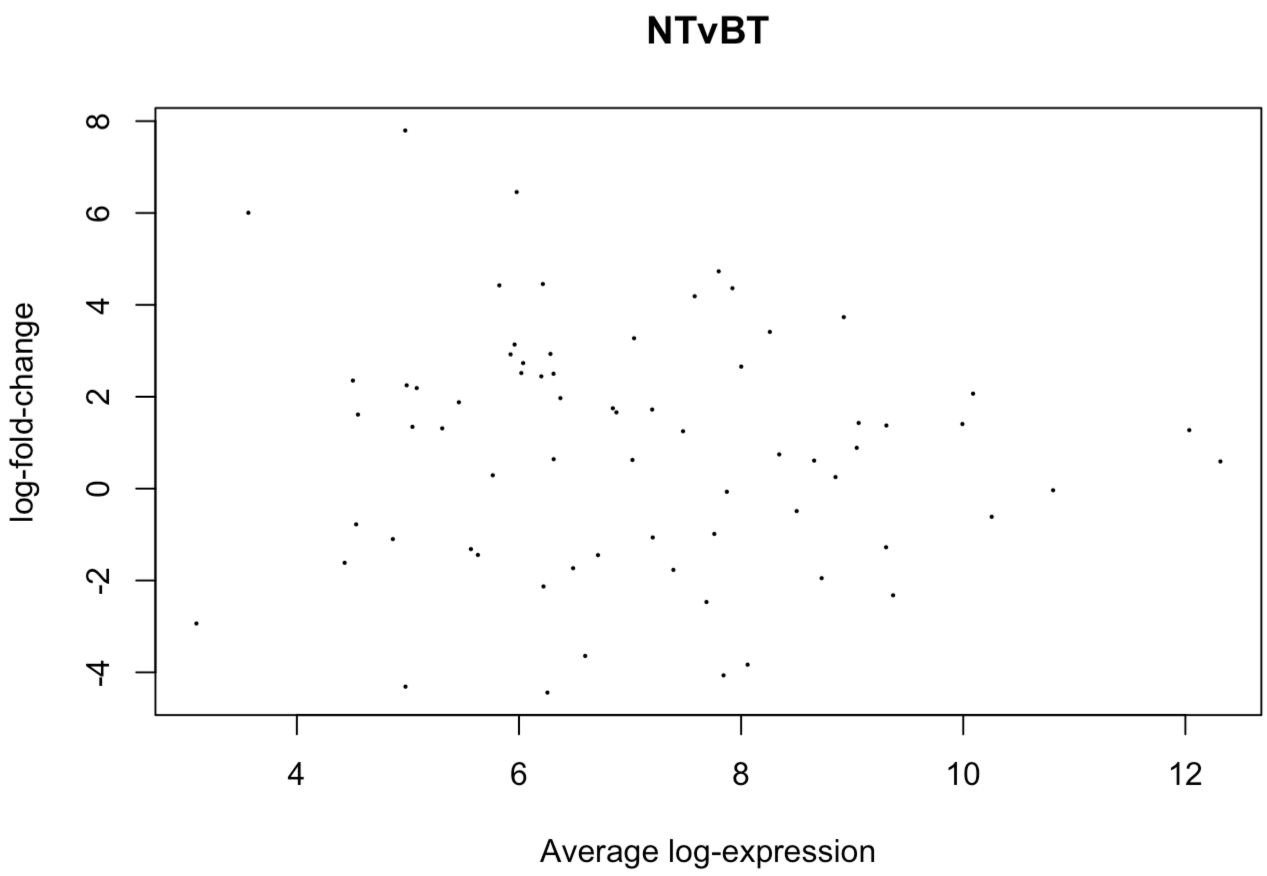


```
plotMD(fit.cont, col=2) # NOR v BALB/c
```

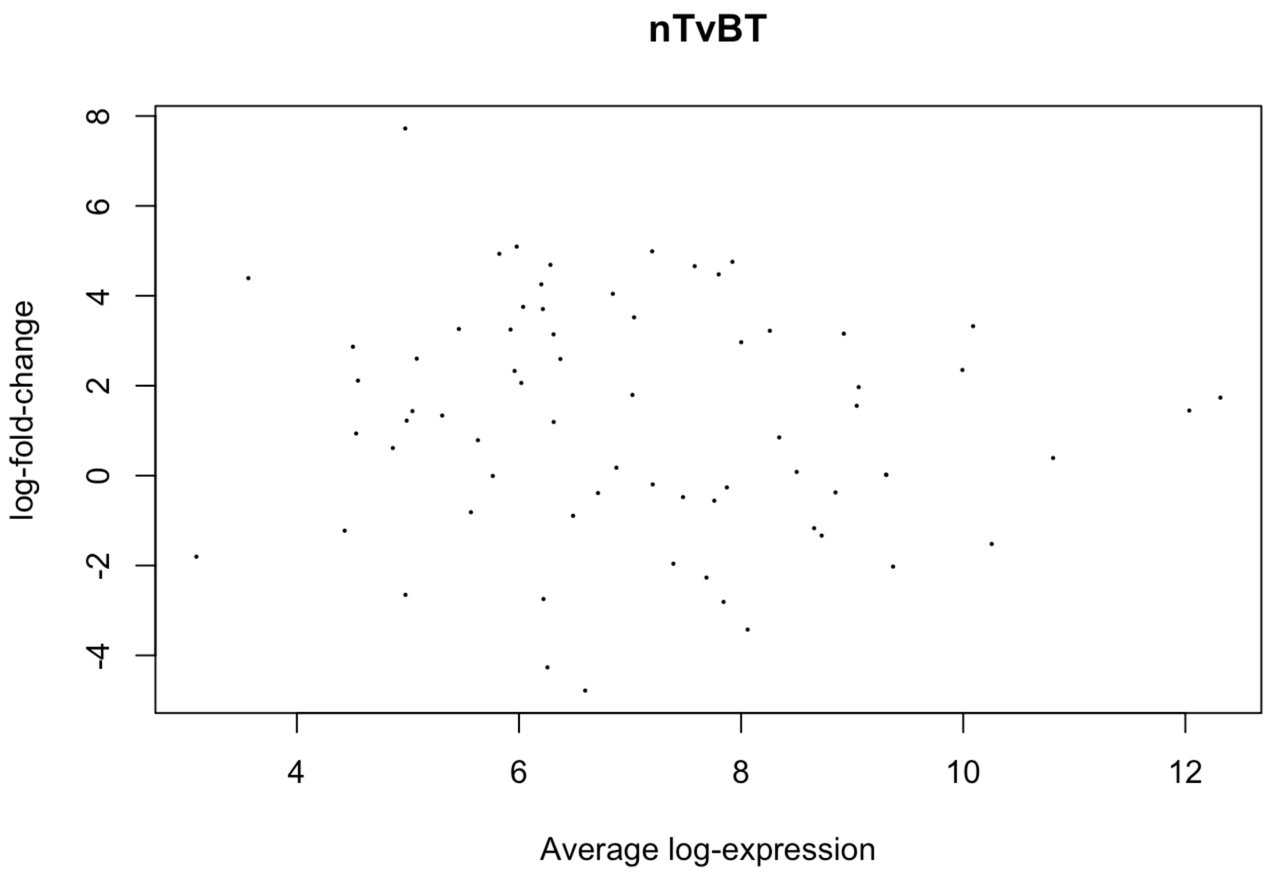


```
# QQ Plots of normalized data
qqt(fit.cont$t,df=fit.cont$df.prior+fit.cont$df.residual)
```

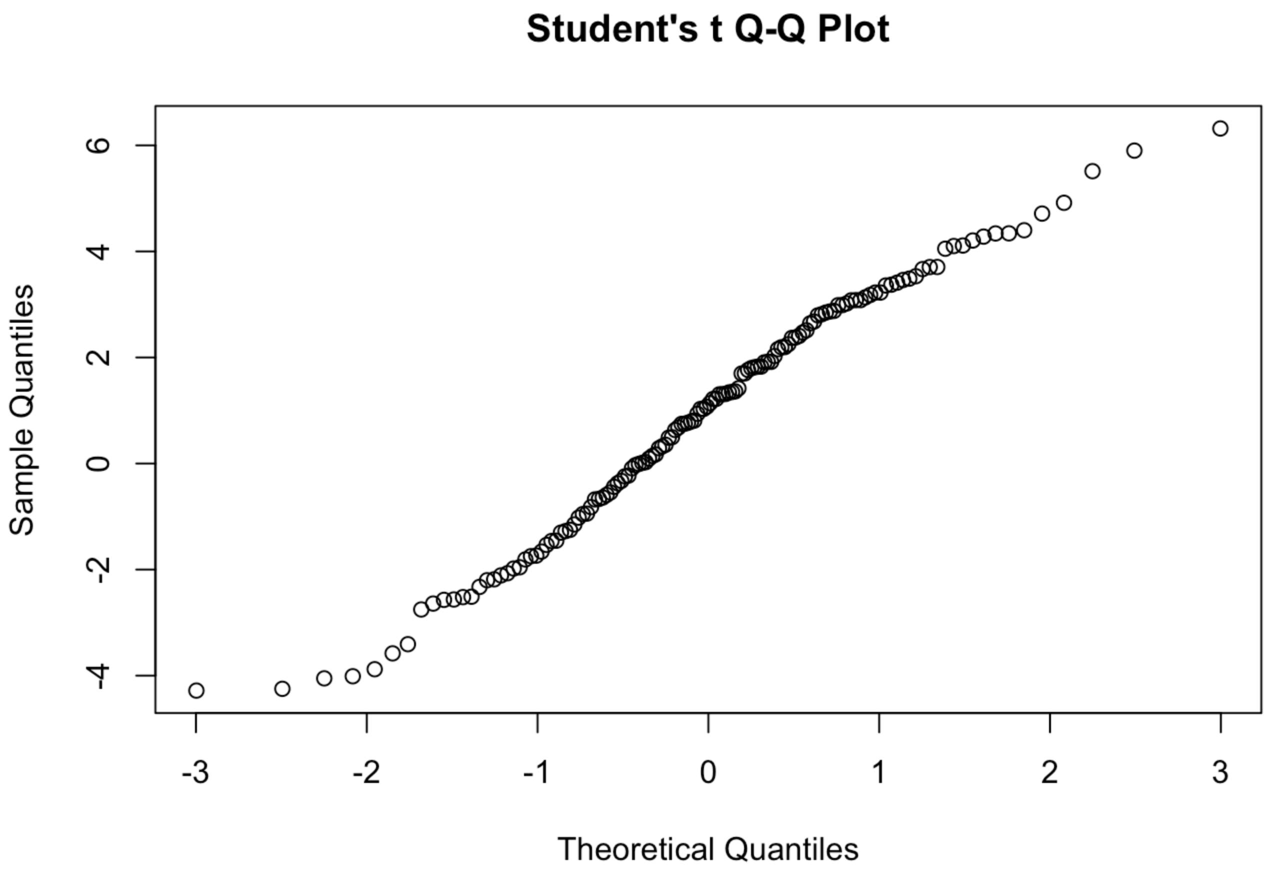


```
#Density plots of normalized Data
plotDensities(fit)
```

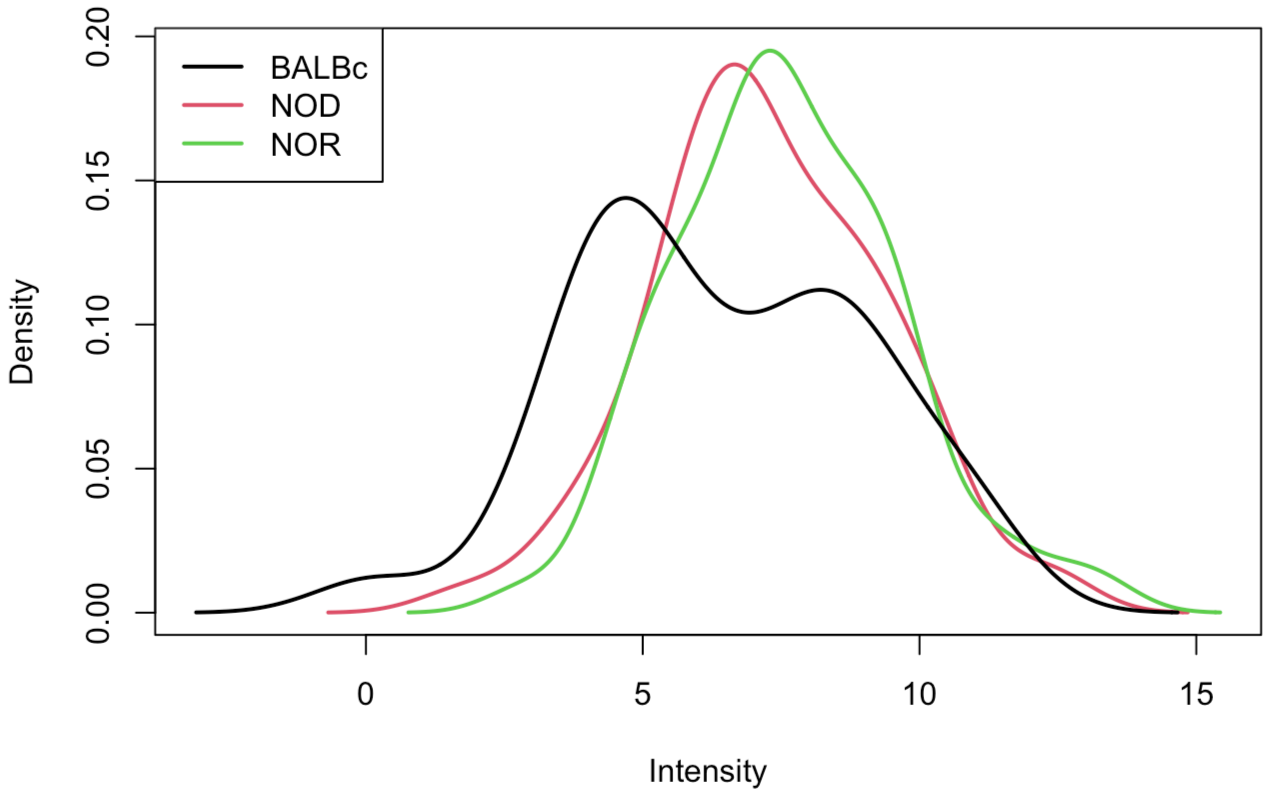

While this normalization works fairly well, most of the BALB/c samples show a relatively low signal intensity across the board. While this my be a true biological effect, we have to consider it as a potential batch effect owing to the fact that BALB/c is a closely matched control of NOD and NOR.

```
#Generating results data frames using Limma's topTable() function
setwd("~/Documents/3_Parkinsons_disease/Autoantibody_Data/Tear_Auto_Validation_2022/IgA_
data/")
NTvsBT <- topTable(fit.cont, coef=1, p.value=1, number=50, adjust.method = 'BH')
nTvsBT <- topTable(fit.cont, coef=2, p.value=1, number=50, adjust.method = 'BH')
NTvsBT$Antigen <- row.names(NTvsBT)
nTvsBT$Antigen <- row.names(nTvsBT)
NTvnTvBT <- full_join(NTvsBT, nTvsBT,
                      by="Antigen",
                      suffix = c(".NOD", ".NOR"))
rownames(NTvnTvBT) <- NTvnTvBT$Antigen
```

4.2 Default Limma Results Table

```
#Generating output file
NTvnTvBT[is.na(NTvnTvBT)] <- 0.5
NTvnTvBT <- NTvnTvBT[-c(which(NTvnTvBT$adj.P.Val.NOD > 0.1 & NTvnTvBT$adj.P.Val.NOR > 0.
1)),]
write.csv(NTvnTvBT, file="Tear_IgA_Limma.csv", sep=',')

print(NTvnTvBT[which(NTvnTvBT$logFC.NOD>1),c(1:2,5,8,12)])
```

```
##             logFC.NOD AveExpr.NOD adj.P.Val.NOD logFC.NOR adj.P.Val.NOR
## Mi-2         7.795830    4.975896  0.0002787797  7.722650   0.000683158
## SAE1 SAE2    6.455780    5.979389  0.0008008029  5.094235   0.005698873
## tTG          4.730122    7.798166  0.0032457358  4.476692   0.005224389
## Jo-1         4.362181    7.921660  0.0047276686  4.756630   0.004607082
## LKM 1        4.453778    6.214859  0.0049074651  3.705070   0.017644014
## TNF a        6.004939    3.563230  0.0049074651  4.392796   0.032703291
## IA-2         4.187879    7.581873  0.0134489150  4.659095   0.009911925
## KS           4.424495    5.822803  0.0134489150  4.934300   0.009911925
## GAD65        3.411583    8.258565  0.0134489150  3.221565   0.020878584
## TPO          3.732875    8.924370  0.0158256914  3.157495   0.040459601
## Gliadin      3.272999    7.036221  0.0221382634  3.520608   0.017644014
## PM Scl75     3.136187    5.960308  0.0259014278  2.328724   0.091275690
## KU P70P80    2.921768    5.924015  0.0299842041  3.248915   0.020472567
## La SSB       2.932182    6.283174  0.0348995668  4.688158   0.004607082
## PL-7         2.656189    8.000905  0.0348995668  2.967767   0.022646584
## LC1          2.500449    6.310963  0.0348995668  3.141955   0.014259124
## IF           2.443567    6.200971  0.0494558660  4.255189   0.004607082
## PCNA         2.188723    5.079314  0.0555662482  2.601838   0.032513431
## SmD1         2.516116    6.020889  0.0582921581  2.061221   0.132603261
## PL-12        1.970215    6.372643  0.0644397656  2.593297   0.023301083
## Cardiolipin  2.732643    6.037356  0.0662231843  3.753691   0.020878584
## GP2          2.067020   10.088505  0.0831644166  3.324054   0.014121733
## BPI          1.611897    4.550018  0.1379397699  2.112938   0.069630435
## NXP2         1.721346    7.199159  0.1588001089  4.989552   0.003059614
## GP210        1.748597    6.845041  0.1725706594  4.044152   0.009911925
## C3           1.879431    5.458823  0.2870342822  3.261196   0.089106039
```

16 IgA autoantibodies are significantly upregulated in both NOD & NOR as compared to male BALB/c with adjusted p value < 0.05

5. Voom Normalization After Removing outliers

Using the Limma Voom package and a quantile normalization approach, we hope to completely remove the batch effect. This should give us a more conservative result.

```
#After removing outlier samples BALB/c 6

###### Voom normalization with quantiles
conditions<- paste(colData$Strain[-15],sep=".")
conditions <- factor(conditions, levels=unique(conditions))
design <- model.matrix(~0+ conditions)
colnames(design) <- levels(conditions)

v <- voom(counts=(IgA_raw$NSI[,-15]+0.5), design, plot=TRUE, normalize="quantile")
```

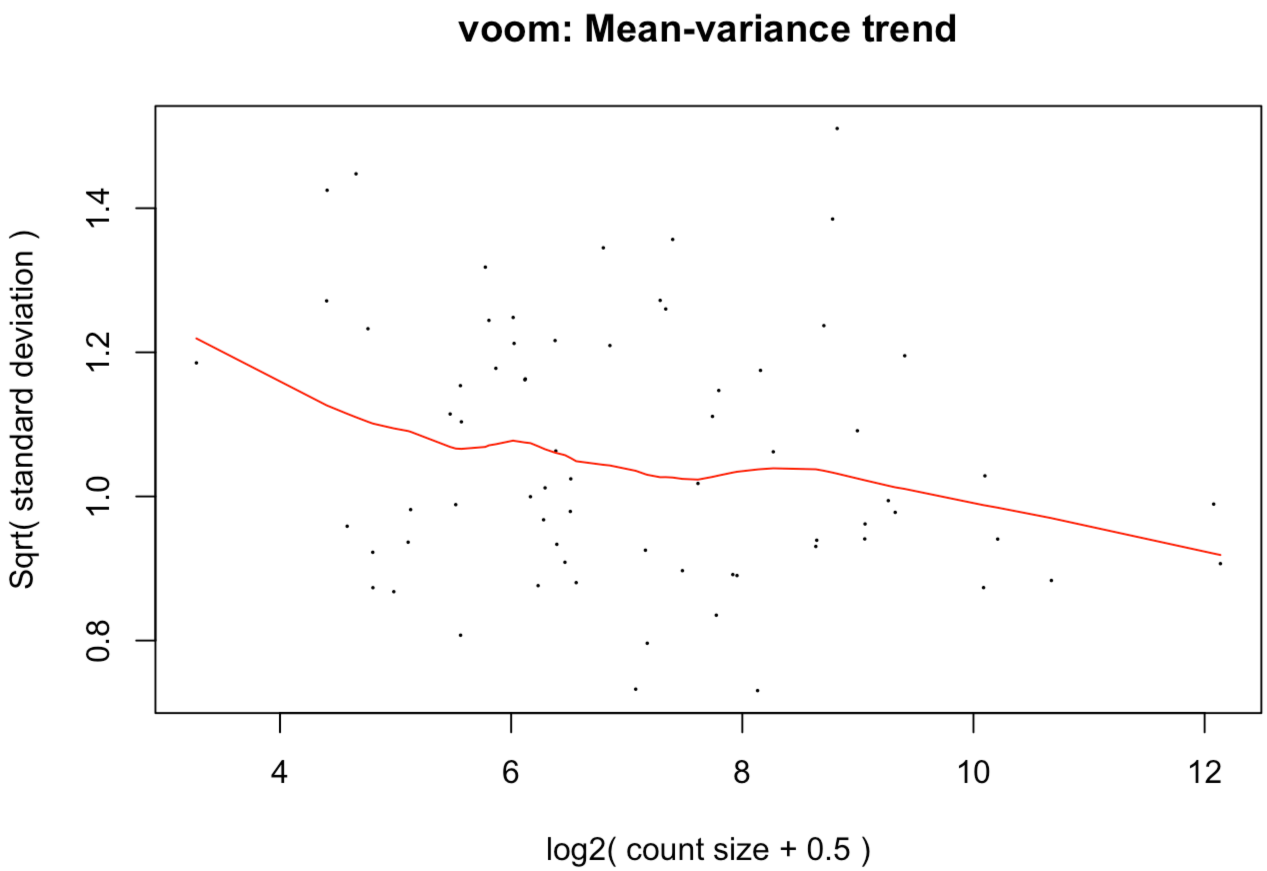


```
#v <- voom(dae, design, plot=TRUE)
fit <- lmFit(v, design)
fit.cont<- contrasts.fit(fit, cont.matrix)
fit.cont<- eBayes(fit.cont)
#plot(x=colSums(IgA_raw$NSI), y=colSums(IgG_raw$NSI))
```

5.1 QC plots of analysis

```
#PC1 separates Diseases and healthy samples
plotMDS(v,col=as.numeric(Strain))
```

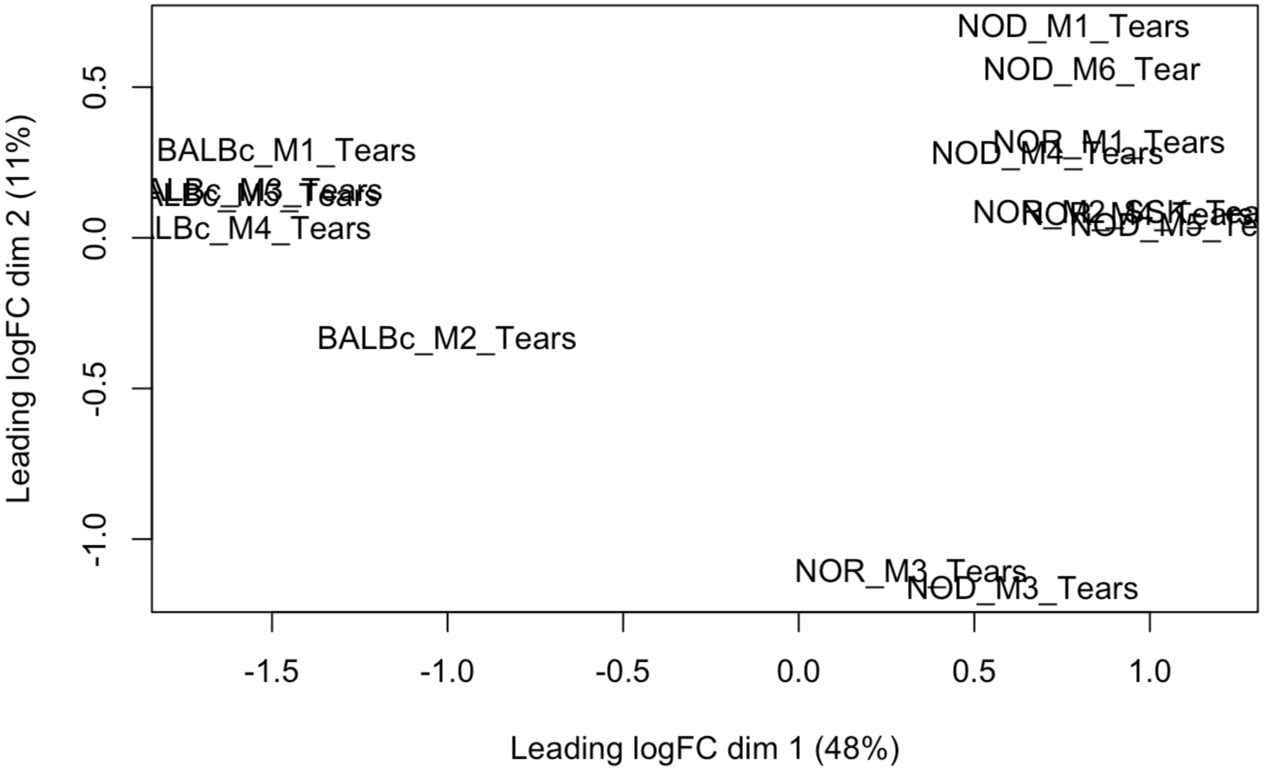


```
#MA plots showing relationship between average expression of autoantibodies and their lo
g fold-changes
plot.new()
plotMD(fit.cont, col=1) # NOD v BALB/c
```

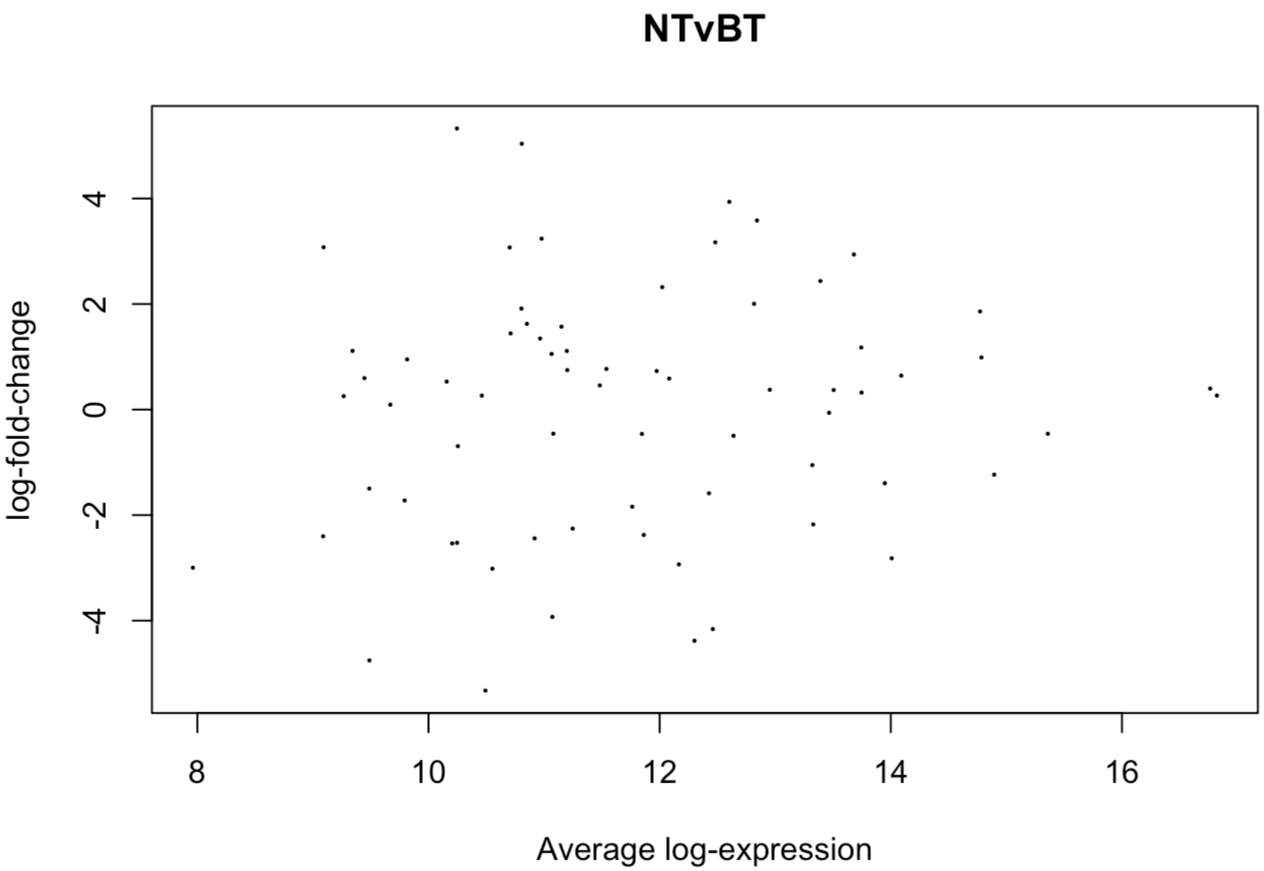


```
plotMD(fit.cont, col=2) # NOR v BALB/c
```

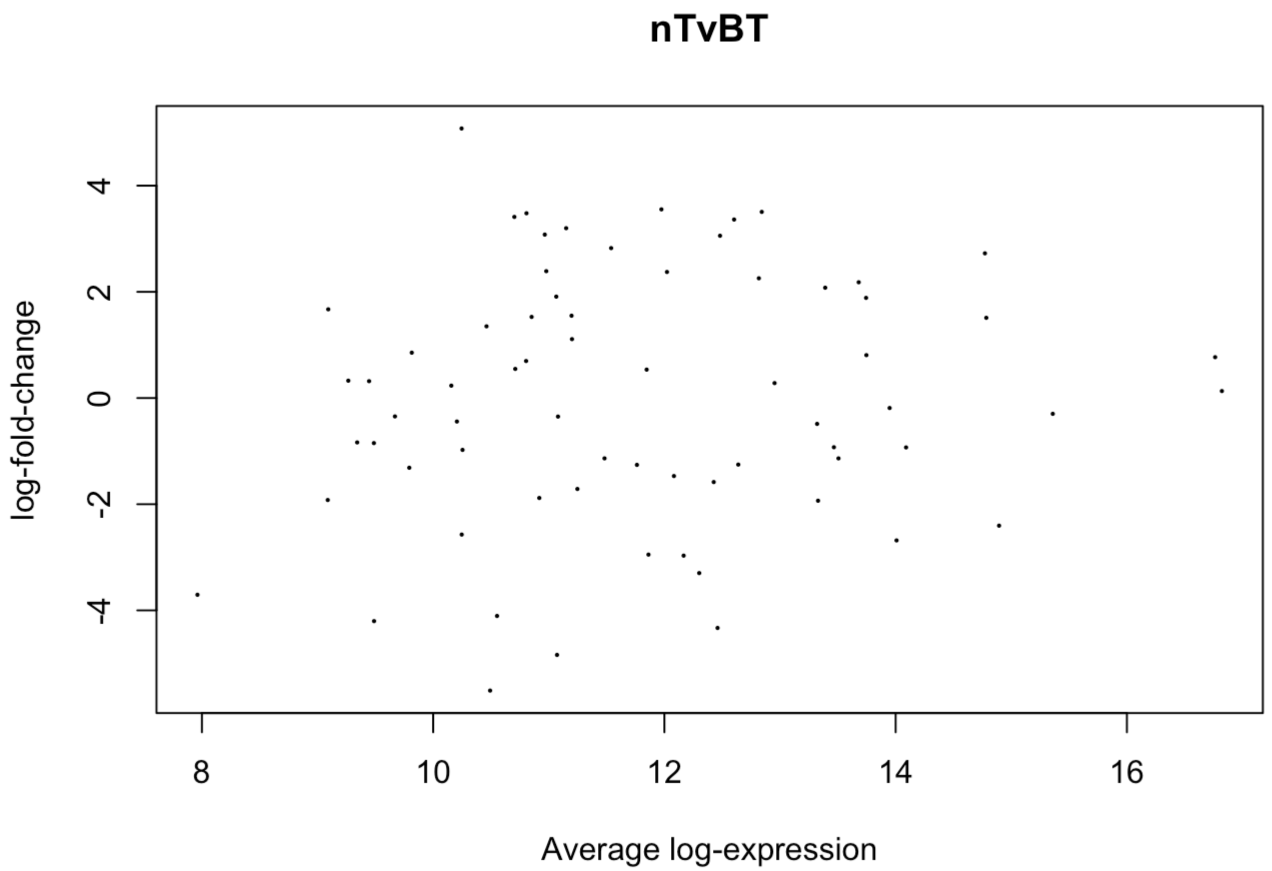


```
# The Q-Q plot is a fairly straight line
qqt(fit.cont$t,df=fit.cont$df.prior+fit.cont$df.residual)
```

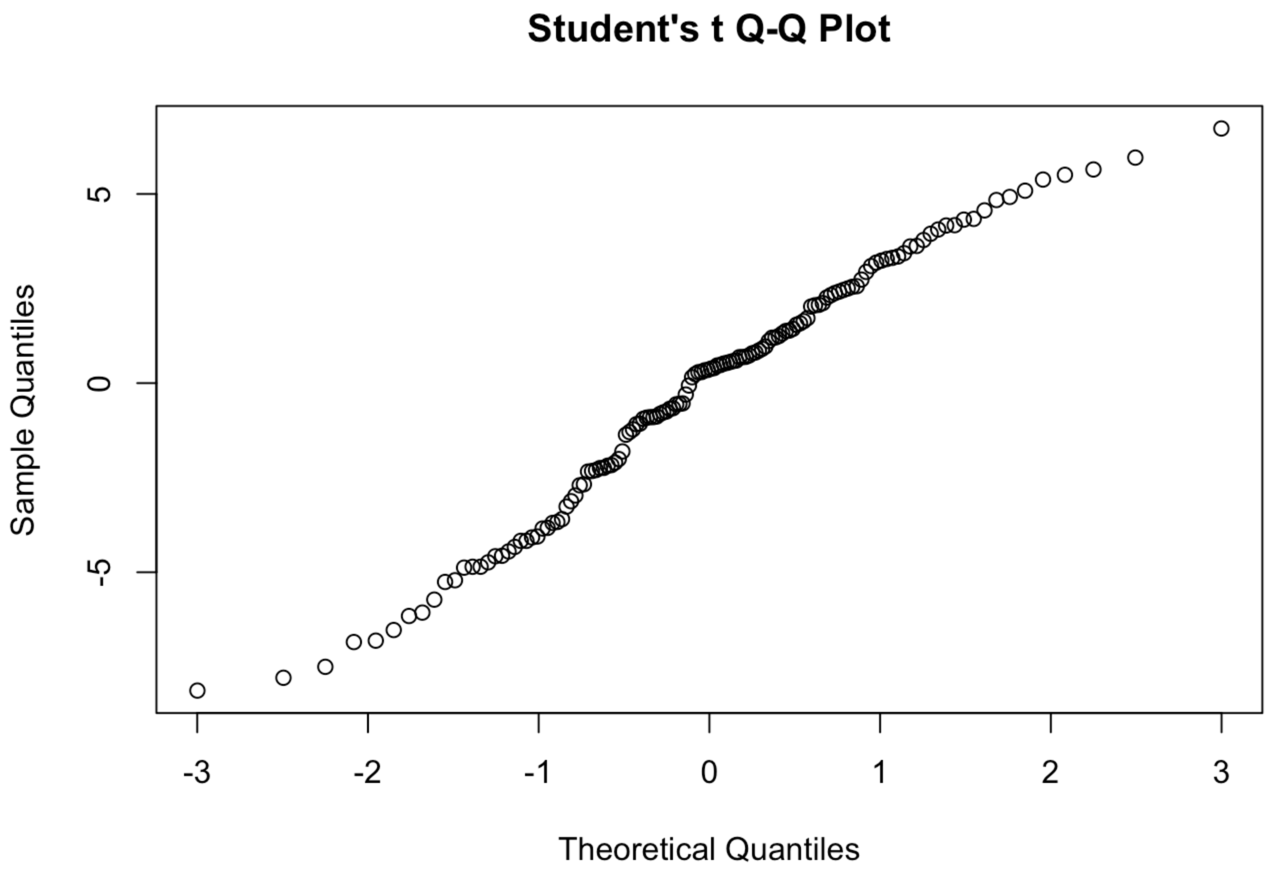


```
# The Density plots of all three groups overlap fairly well, confirming that the batch e
ffect has been largely normalized
plotDensities(v, group=Strain, col=c("orange","green", "blue"), log=TRUE)
```

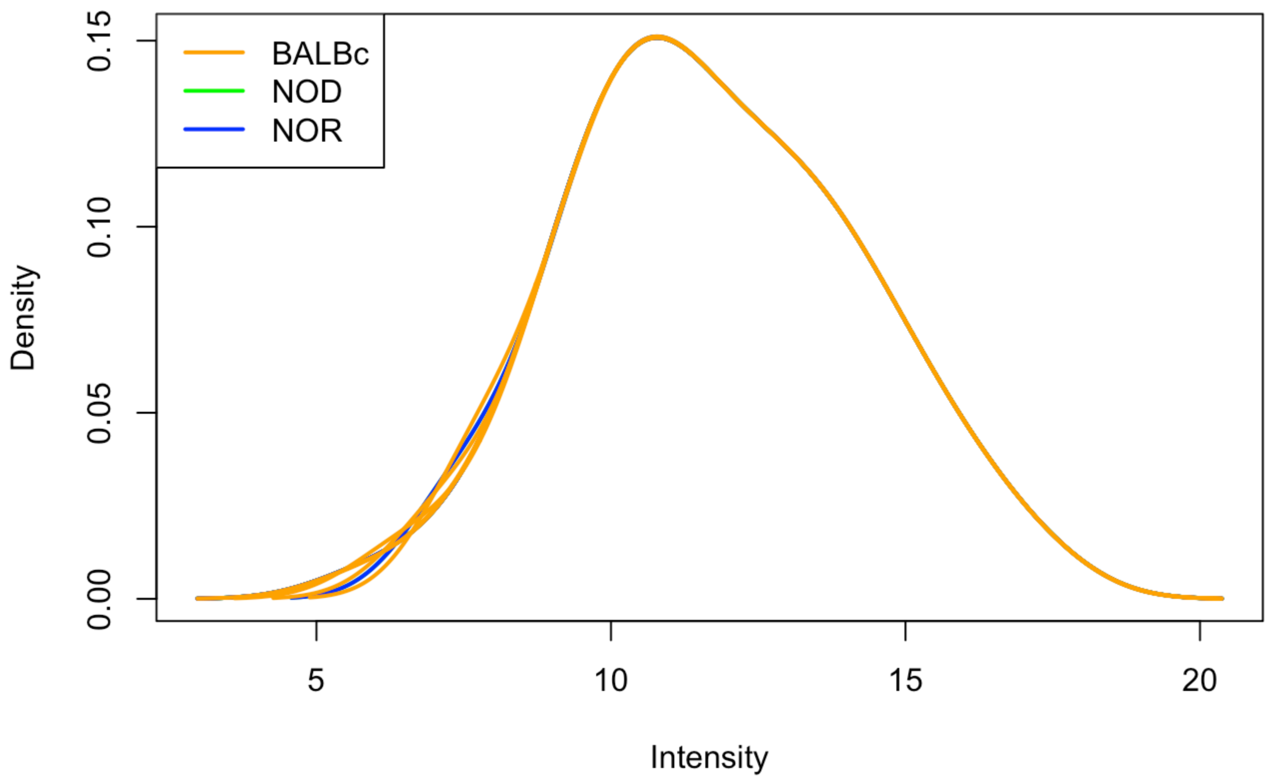

5.2 Density plots of normalized data

```
#Density plots

df_dseq = melt(v$E, variable.name = "Samples", value.name = "count")# reshape the matrix
df_dseq$Strain <- factor(substr(df_dseq$Var2, 1,3))
mycolors <- colorRampPalette(brewer.pal(8,"Set1"))(15)

ggplot(df_dseq, aes(x = count, color=Var2)) +
   geom_density(alpha = 0.5, size = 0.8) +
   facet_wrap(~Strain, ncol=2) +
   theme_minimal() + xlim(-5, 25) +
   scale_colour_manual(values=mycolors, name="") +
   guides(fill="none")
```

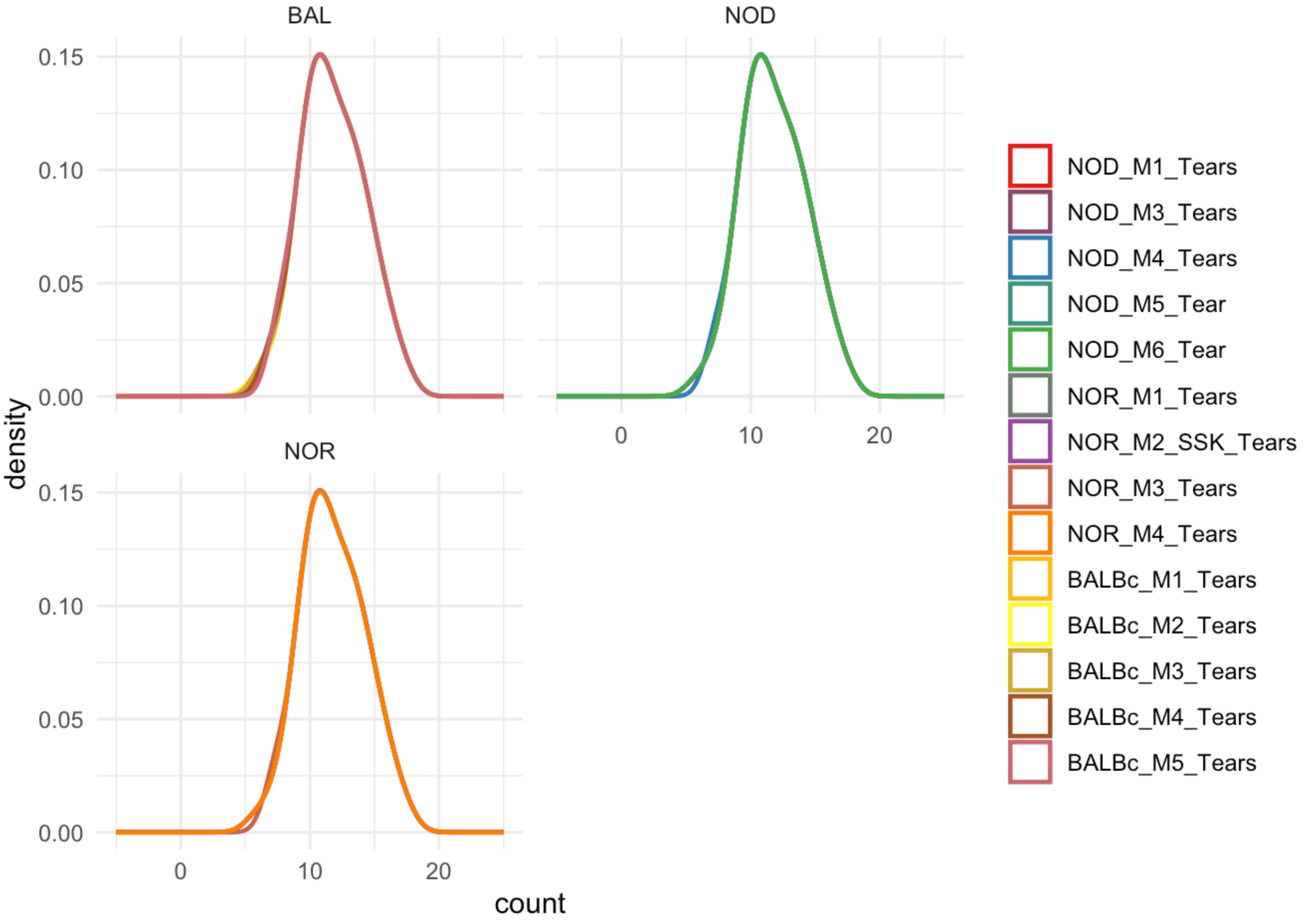

Sample wise Density plots facetted by the mouse strain show that voom normalized Log2 transformed NSI data of each sample overlap quite well and follow normal distribution.

5.3 QC: Assessing Goodness of fit

```
#R-Squared ....... goodness of fit
for (i in 1:69){
   sst <- rowSums(v$E^2)
   ssr <- sst - fit.cont$df.residual*(fit.cont$sigma^2)
   Rsq<- (ssr/sst)
}
plot(1:69, Rsq)
```

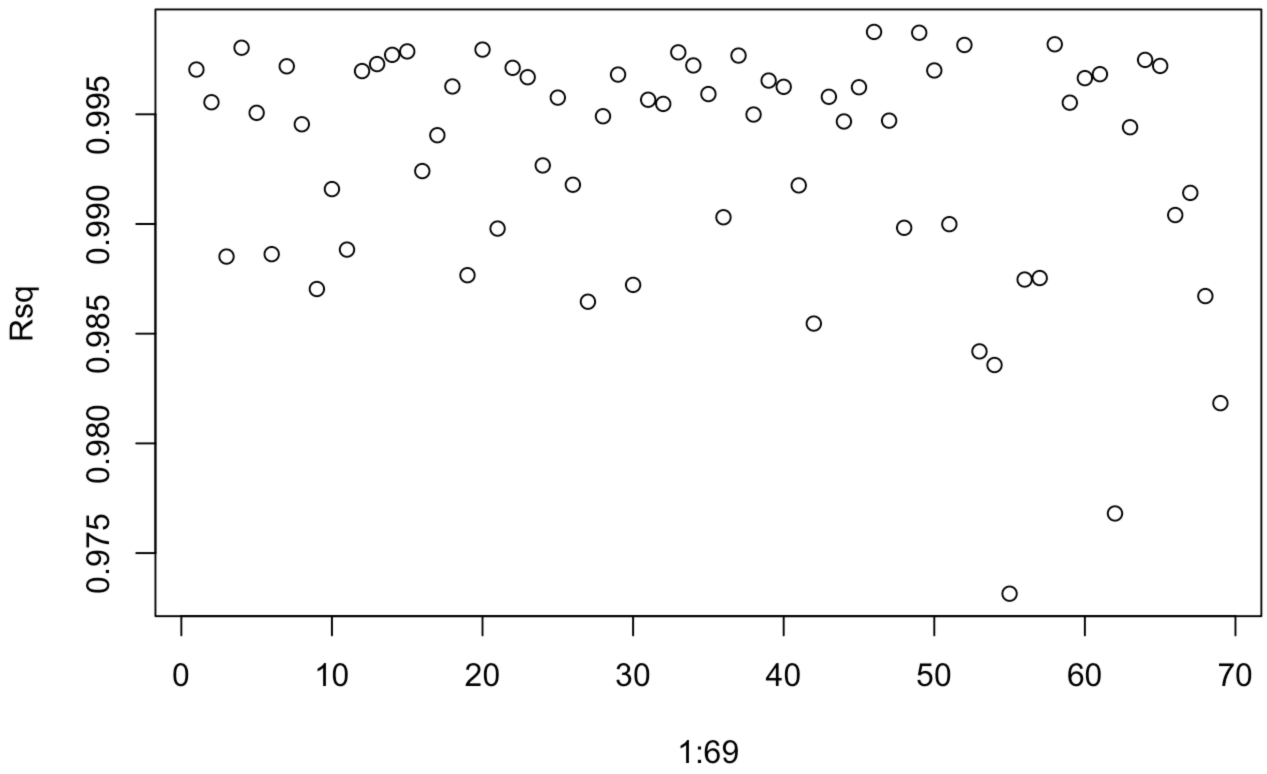


```
which(Rsq<0.90)
```

```
## named integer(0)
```

```
summary(fit.cont$r.squared)
```

```
## Length  Class   Mode
##      0   NULL   NULL
```

5.4 Results Table: Limma-Voom (Quantile) Normalized DE

```
setwd("~/Documents/3_Parkinsons_disease/Autoantibody_Data/Tear_Auto_Validation_2022/IgA_
data/")
NTvsBT <- topTable(fit.cont, coef=1, p.value=1, number=50, adjust.method = 'BH')
nTvsBT <- topTable(fit.cont, coef=2, p.value=1, number=50, adjust.method = 'BH')
NTvsBT$Antigen <- row.names(NTvsBT)
nTvsBT$Antigen <- row.names(nTvsBT)
NTvnTvBT <- full_join(NTvsBT, nTvsBT,
                      by="Antigen",
                      suffix = c(".NOD", ".NOR"))
rownames(NTvnTvBT) <- NTvnTvBT$Antigen
NTvnTvBT[is.na(NTvnTvBT)] <- 0.5
NTvnTvBT <- NTvnTvBT[c(which(NTvnTvBT$adj.P.Val.NOD < 0.1 & NTvnTvBT$adj.P.Val.NOR < 0.1
& NTvnTvBT$logFC.NOD > 0)),]
write.csv(NTvnTvBT, file="Tear_IgA_voom.csv", sep=',')
knitr::kable(NTvnTvBT[which(NTvnTvBT$logFC.NOD>1),c(1:2,5,8,12)])
```

	logFC.NOD	AveExpr.NOD	adj.P.Val.NOD	logFC.NOR	adj.P.Val.NOR
tTG	3.937176	12.60275	0.0000301	3.361831	0.0003309
Mi-2	5.326484	10.24579	0.0001019	5.076768	0.0003639
SAE1 SAE2	5.037635	10.80710	0.0001513	3.479841	0.0056485
LKM 1	3.238098	10.97834	0.0007383	2.389130	0.0103069
Jo-1	3.582995	12.84206	0.0016525	3.505987	0.0019247
TPO	2.938684	13.68044	0.0021488	2.180326	0.0190204
IA-2	3.169350	12.48119	0.0024533	3.056493	0.0042021
PL-7	2.003696	12.81699	0.0030162	2.255281	0.0015144
GP2	1.859388	14.77217	0.0074657	2.725116	0.0005968
KS	3.072038	10.70215	0.0126520	3.411389	0.0098839
GAD65	2.435629	13.39060	0.0149507	2.077640	0.0434380
La SSB	1.571117	11.15028	0.0419061	3.198636	0.0005283
Gliadin	2.319357	12.02238	0.0461896	2.374340	0.0502645
KU P70P80	1.624575	10.85163	0.0532829	1.527862	0.0861559
IF	1.345973	10.96544	0.0942128	3.077987	0.0010621
dsDNA	1.176245	13.74426	0.0979555	1.886362	0.0108055

5.5 Results Output

```
Result_IgA <- as.data.frame(
   cbind(rownames(NTvnTvBT),
         round(NTvnTvBT$adj.P.Val.NOD, 6),
         paste0(round(NTvnTvBT$logFC.NOD, 2),
                " (",
                (round(2^NTvnTvBT$logFC.NOD, 2)),
                ")"),
         round(NTvnTvBT$adj.P.Val.NOR, 6),
         paste0(round(NTvnTvBT$logFC.NOR, 2),
                " (",
                (round(2^NTvnTvBT$logFC.NOR, 2)),
                ")")
         )
   )

setwd("~/Documents/3_Parkinsons_disease/Autoantibody_Data/Tear_Auto_Validation_2022/IgA_
data/")
colnames(Result_IgA) <- c("Antigen", "p adj NOD", "Log2FC (FC) NOD", "p adj NOR", "Log2F
C (FC) NOR")
Result_IgA
```

```
##       Antigen p adj NOD Log2FC (FC) NOD p adj NOR Log2FC (FC) NOR
## 1         tTG     3e-05    3.94 (15.32)  0.000331    3.36 (10.28)
## 2        Mi-2  0.000102    5.33 (40.13)  0.000364    5.08 (33.75)
## 3  SAE1 SAE2   0.000151    5.04 (32.85)  0.005648    3.48 (11.16)
## 4       LKM 1  0.000738     3.24 (9.44)  0.010307     2.39 (5.24)
## 5        Jo-1  0.001652    3.58 (11.98)  0.001925    3.51 (11.36)
## 6         TPO  0.002149     2.94 (7.67)   0.01902     2.18 (4.53)
## 7        IA-2  0.002453        3.17 (9)  0.004202     3.06 (8.32)
## 8        PL-7  0.003016        2 (4.01)  0.001514     2.26 (4.77)
## 9         GP2  0.007466     1.86 (3.63)  0.000597     2.73 (6.61)
## 10         KS  0.012652     3.07 (8.41)  0.009884    3.41 (10.64)
## 11      GAD65  0.014951     2.44 (5.41)  0.043438     2.08 (4.22)
## 12     La SSB  0.041906     1.57 (2.97)  0.000528      3.2 (9.18)
## 13    Gliadin   0.04619     2.32 (4.99)  0.050265     2.37 (5.18)
## 14  KU P70P80  0.053283     1.62 (3.08)  0.086156     1.53 (2.88)
## 15         IF  0.094213     1.35 (2.54)  0.001062     3.08 (8.44)
## 16      dsDNA  0.097956     1.18 (2.26)  0.010805      1.89 (3.7)
```

```
write.csv(Result_IgA, file="Results_Tear_IgA.csv", sep=',')
```

Thirteen Auto-antibodies are significantly upregulated in tears of both male NOD & male NOR as compared to tears of healthy male BALB/c.

6. Plotting Differentially Expressed Autoantibodies

6.1 Boxplots from voom normalized counts

```
chart_design <- theme(
   plot.title = element_text(color = "Black", size = 16, face = "bold", margin = margin
(b=15), hjust=0.4),
   axis.text.x = element_text(size=15),
   axis.text.y = element_text(size=14),
   axis.title.x = element_blank(),
   legend.text = element_text(size=15),
   legend.title = element_blank(),
   legend.position = "bottom",
   axis.title.y = element_text(size=18, margin = margin(r = 5)),
   strip.text.x = element_text(size =16, margin = margin(b=20), face='bold', hjust=0.4),
   strip.background = element_blank(),
   strip.placement = "outside")

mydata <- as.matrix(v$E)
hits <- rownames(NTvnTvBT)
hits[(length(hits)+1)] <- "Ro SSA 52"
#hits[(length(hits)+1)] <- "RoSSA 60"

Y=matrix(nrow=length(hits),ncol=14)

for (i in 1:length(hits)) {
   Y[i,] <- mydata[hits[i],]
}
rownames(Y) <- hits
colnames(Y) <- colData$Sample[-15]

Y <- as.data.frame(t(Y))
Y$Strain <- colData$Strain[-15]
Y$Sample <- paste0(Y$Strain, c(1:5,1:4, 1:5))

setwd("~/Documents/3_Parkinsons_disease/Autoantibody_Data/Tear_Auto_Validation_2022/IgA_
data/")
for (i in length(hits)){
   filename <- paste(hits[i],"IgA_Tears.tiff", sep="")
   p<- ggplot(Y, aes(x=Strain, y=Y[,i], fill=Strain)) +
      geom_boxplot(outlier.shape = NA, width = 0.5, coef=1, varwidth=F, show.legend = T,
size=0.9, position = position_dodge(0.9)) +
      geom_jitter(color = "darkgray", alpha =0.7, size=2.5, show.legend = F, position =
position_jitterdodge(dodge.width=0.9))+
      scale_color_manual(values=c("black", "navy")) +
      theme_minimal() +
      chart_design +
      ylab("Log Norm Intensity")  +
      scale_x_discrete(labels= c("BALB/c", "NOD", "NOR")) +
      labs(title=colnames(Y[i]), hjust=0.5) +
      scale_fill_jco()
   tiff(filename, units="in", width=3.45, height=3.5, res=300)
   print(p)
   dev.off()
```

```
}
#print(p)
```

6.3 Combined Boxplots of Upregulated hits

```
chart_design <- theme(
   #plot.title = element_text(color = "Black", size = 16, face = "bold", margin = margin
(b=15), hjust=0.4),,
   axis.text.x = element_text(size=16, margin = margin(b=5)),
   axis.text.y = element_text(size=18),
   axis.title.x = element_blank(),
   legend.text = element_blank(),
   legend.title = element_blank(),
   legend.position = "top",
   axis.title.y = element_text(size=24, margin = margin(r = 5)),
   strip.text.x = element_text(size =19, margin = margin(b=15), face='bold', hjust=0.4),
   strip.background = element_blank(),
   strip.placement = "outside")

#Generating Data Frame for plotting Combined boxplot
Y_combined <- Y[,c(NTvnTvBT$Antigen[which(NTvnTvBT$adj.P.Val.NOD<0.051 & NTvnTvBT$adj.P.
Val.NOR < 0.051 & NTvnTvBT$logFC.NOD>0)])]
#Y_combined[,14:15] <- Y[,c("IF", "La SSB")]
Y_combined[,(ncol(Y_combined)+1):(ncol(Y_combined)+2)] <- Y[,(ncol(Y)-1):(ncol(Y))]
Y_combined <- gather(Y_combined, "Antigen", "V Counts", 1:(ncol(Y_combined)-2))
#Y_combined[which(Y_combined$Strain=="BALBc"),"Strain"] = factor("BALB/c")



#Generating combined boxplot of 13 autoantibodies using ggplot()
setwd("~/Documents/3_Parkinsons_disease/Autoantibody_Data/Tear_Auto_Validation_2022/IgA_
data/")
tiff("Tear_IgA_hits.tiff", units="in", width=12.2, height=8.5, res=300)
q <- ggplot(Y_combined, aes(x=Strain, y=`V Counts`, fill=Strain)) +
      geom_boxplot(outlier.shape = NA, width = 0.7, coef=1, varwidth=F, show.legend = F,
size=0.9, position = position_dodge(0.9)) +
      geom_jitter(color = "darkgray", alpha =0.6, size=3.3, show.legend = F, position =
position_jitterdodge(dodge.width=1.2))+
   facet_wrap(~Antigen, ncol=5, scales="free_x") +
      theme_minimal() +
      chart_design +
      ylab("Log Normalized Intensity")  +
   scale_x_discrete(labels= c("BALB/c", "NOD", "NOR")) +
      labs(title=factor(Y_combined$Antigen), hjust=0.5) +
      scale_fill_jco()
print(q)
dev.off()
```

```
## quartz_off_screen
##                 2
```

```
(q)
```

tTG

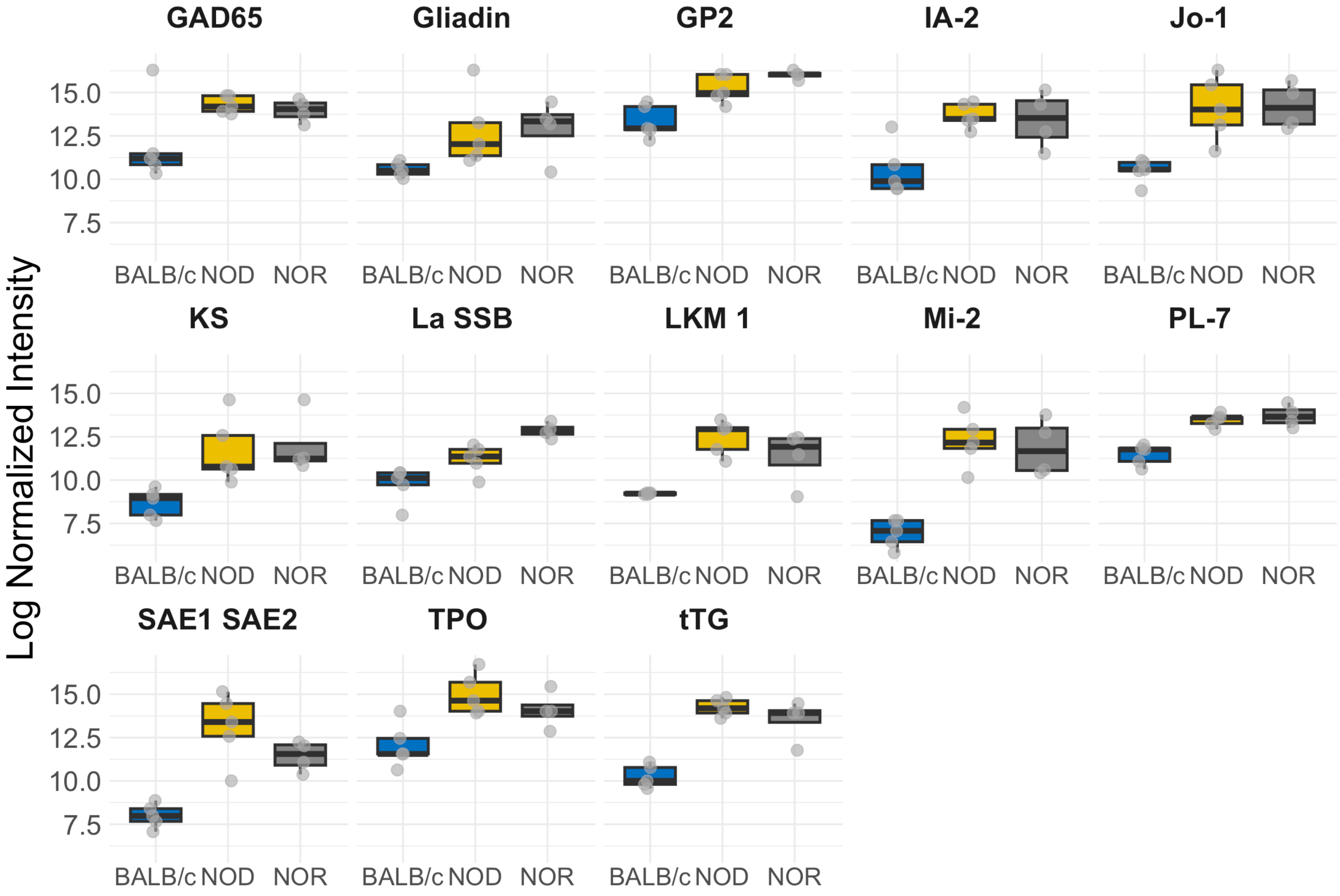

7. Code for Comparing Total Tear IgG and IgA Signal Intensity

(ColSums)

```
Tear_IgG_NSI_raw <- read.csv("~/Documents/3_Parkinsons_disease/Autoantibody_Data/Tear_Au
to_Validation_2022/IgG_MCF_SSK_546_Tear_NSI.csv", header=TRUE, row.names = 1, nrows=88)
Tear_IgG_NSI_raw <- Tear_IgG_NSI_raw[,1:15]
Tear_IgG_SNR_raw <- read.csv("~/Documents/3_Parkinsons_disease/Autoantibody_Data/Tear_Au
to_Validation_2022/IgG_MCF_SSK_546_Tear_SNR.csv", header=TRUE, row.names = 1, , nrows=8
8)

Tear_IgA_NSI_raw <- read.csv("~/Documents/3_Parkinsons_disease/Autoantibody_Data/Tear_Au
to_Validation_2022/IgA_data/IgA_MCF_SSK_546_Tear_NSI.csv", header=TRUE, row.names = 1)
Tear_IgA_NSI_raw <- Tear_IgA_NSI_raw[1:88,]

x = colSums(Tear_IgG_NSI_raw[1:80,1:14])
y = colSums(Tear_IgA_NSI_raw[1:80,1:14])

data <- as.data.frame(cbind(x,y))
colnames(data) <- c("IgG","IgA")
Strain <- c(rep("NOD", each=5),rep("NOR", each=4), rep("BALBc", each=5))
data$Strain <- factor(Strain)
data$Sample <- rownames(data)
data$delta <- data$IgA-data$IgG
delta <- ggplot(data, aes(x=Strain, y=(delta), fill=Strain)) +
   geom_boxplot(outlier.shape = NA, width = 0.8, coef=1, varwidth=F, show.legend = T, si
ze=0.9, position = position_dodge(0.9)) +
   geom_jitter(alpha =0.6, size=2.5, show.legend = T, position = position_jitterdodge(do
dge.width=0.9)) +
   scale_fill_jco() +
   theme_minimal() +
   ylab("Tear IgA - Tear IgG") +
   chart_design #+
   #ylim(1,(290000))
delta
```

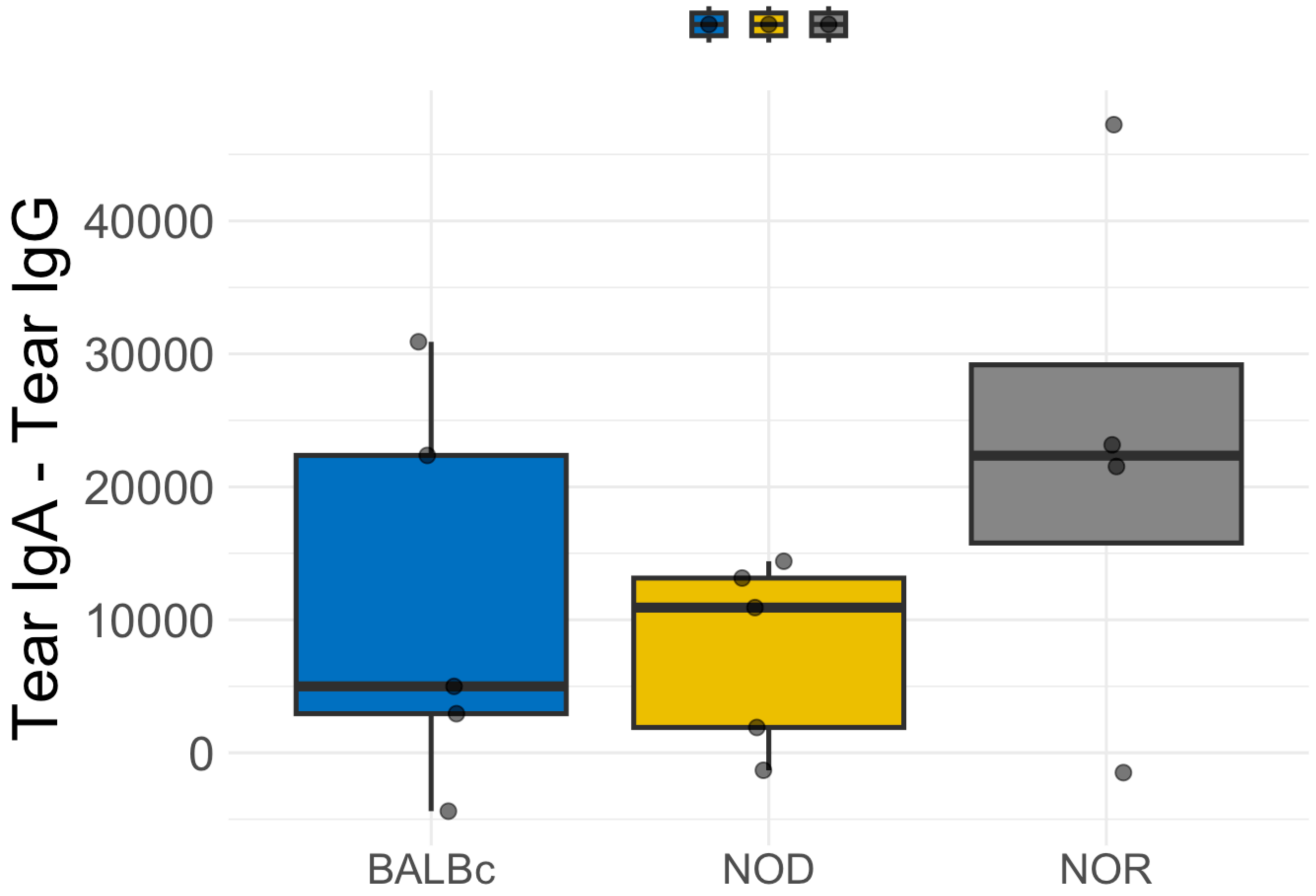


```
data <- gather(data, "Ig", "Intensity", 1:2)
```

7.1 Total tear IgG total IgA intensity for 80 antigens (column sum)

```
chart_design <- theme(
   #plot.title = element_text(color = "Black", size = 16, face = "bold", margin = margin
(b=15), hjust=0.4),,
   axis.text.x = element_text(size=16, margin = margin(b=5)),
   axis.text.y = element_text(size=18),
   axis.title.x = element_blank(),
   legend.text = element_text(size=10),
   legend.title = element_blank(),
   legend.position = "top",
   axis.title.y = element_text(size=20, margin = margin(r = 5)),
   strip.text.x = element_text(size =19, margin = margin(b=15), face='bold', hjust=0.4),
   strip.background = element_blank(),
   strip.placement = "outside")

head(data)
```

```
##   Strain       Sample   delta  Ig Intensity
## 1    NOD NOD_M1_Tears 14404.8 IgG   34919.4
## 2    NOD NOD_M3_Tears 13141.7 IgG   52060.1
## 3    NOD NOD_M4_Tears  1904.6 IgG   20535.2
## 4    NOD  NOD_M5_Tear 10921.4 IgG   48398.8
## 5    NOD  NOD_M6_Tear -1317.7 IgG   23553.9
## 6    NOR NOR_M1_Tears 47244.1 IgG   82615.4
```

```
data <- data[-which(data$Sample %in% c("NOR_M3_Tears")),]
p <- ggplot(data, aes(x=Strain, y=(Intensity), fill=Ig)) +
   geom_boxplot(outlier.shape = NA, width = 0.8, coef=1, varwidth=F, show.legend = T, si
ze=0.9, position = position_dodge(0.9)) +
   geom_jitter(aes(colour = Ig), alpha =0.6, size=2.5, show.legend = T, position = posit
ion_jitterdodge(dodge.width=0.9)) +
   scale_fill_jco() +
   theme_minimal() +
   ylab("Total Intensity of Autoantibodies") +
   chart_design #+
   #ylim(1,(290000))

tiff("ColSum_IgA_vs_IgG.tiff", units="in", width=5.5, height=4.5, res=300)
setwd("~/Documents/3_Parkinsons_disease/Autoantibody_Data/Tear_Auto_Validation_2022/")
print(p)
dev.off()
```

```
## quartz_off_screen
##                 2
```

```
print(p)
```

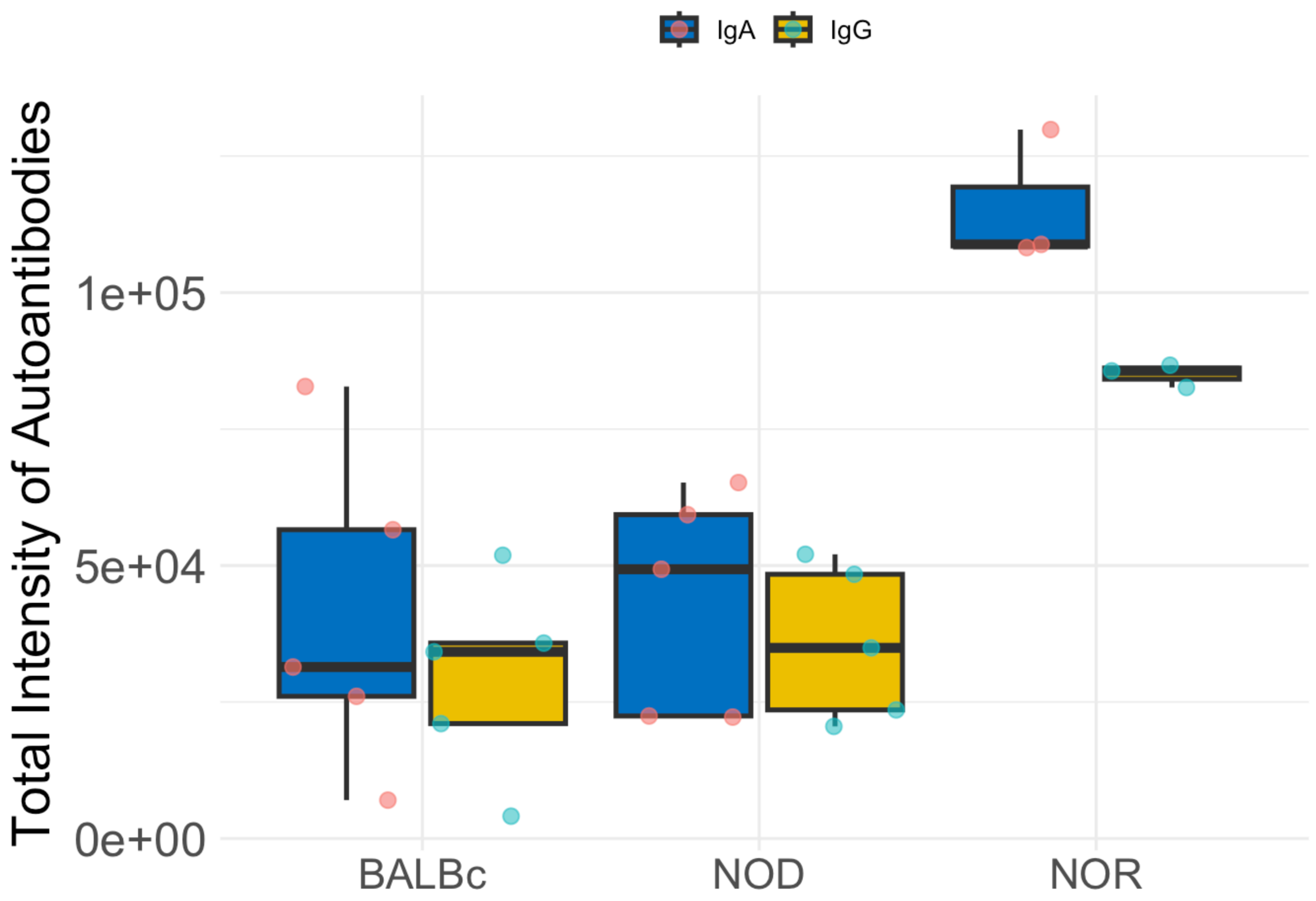

While sum of intensity of 80 autoantigens for IgA is higher in NOR mice tears than IgG, this increase is not statistically significant.

No significant difference was observed between the mean signal IgG intensity as compared to IgA signal intensity for the 80 autoantigens tested in either male NOD or BALB/c mice.

7.1.1 Statistics on ColSums(Normalized Intensity)

```
mod <- lm(log(Intensity) ~ Strain + Ig, data)
aov(mod)
```

```
## Call:
##    aov(formula = mod)
##
## Terms:
##                    Strain       Ig Residuals
## Sum of Squares  6.830875 0.422103  9.429600
## Deg. of Freedom        2        1        22
##
## Residual standard error: 0.6546894
## Estimated effects may be unbalanced
```

```
summary(aov(mod))
```

```
##             Df Sum Sq Mean Sq F value  Pr(>F)
## Strain       2  6.831   3.415   7.968 0.00249 **
## Ig           1  0.422   0.422   0.985 0.33181
## Residuals   22  9.430   0.429
## ---
## Signif. codes:  0 '***' 0.001 '**' 0.01 '*' 0.05 '.' 0.1 ' ' 1
```

```
TukeyHSD(aov(mod))
```

```
##   Tukey multiple comparisons of means
##     95% family-wise confidence level
##
## Fit: aov(formula = mod)
##
## $Strain
##                diff        lwr      upr     p adj
## NOD-BALBc 0.3315339 -0.4039630 1.067031 0.5049407
## NOR-BALBc 1.3323640  0.4830853 2.181643 0.0019346
## NOR-NOD   1.0008301  0.1515514 1.850109 0.0190217
##
## $Ig
##               diff        lwr       upr     p adj
## IgG-IgA -0.2548313 -0.7873817 0.2777192 0.3318066
```

```
plot(mod, which = 2)
```

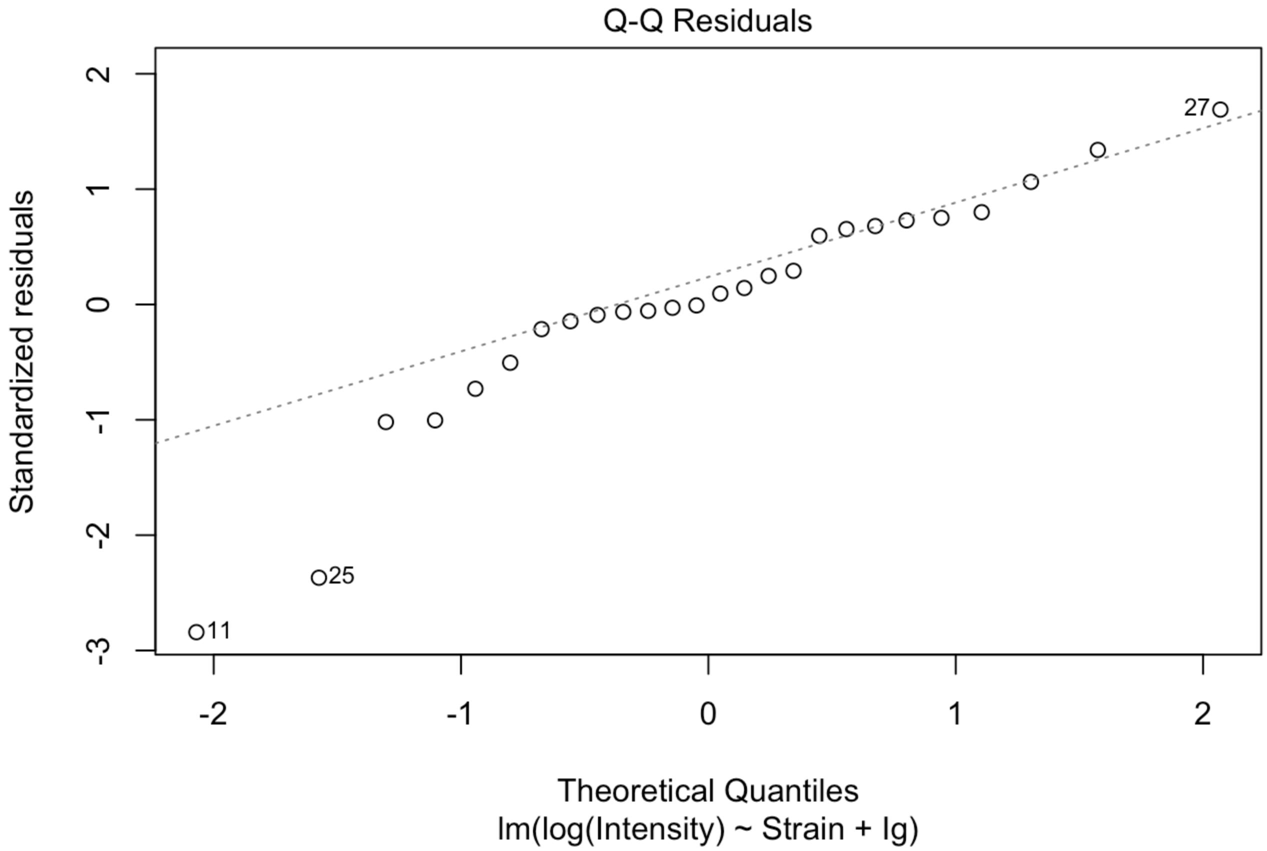


```
hist(mod$residuals)
```

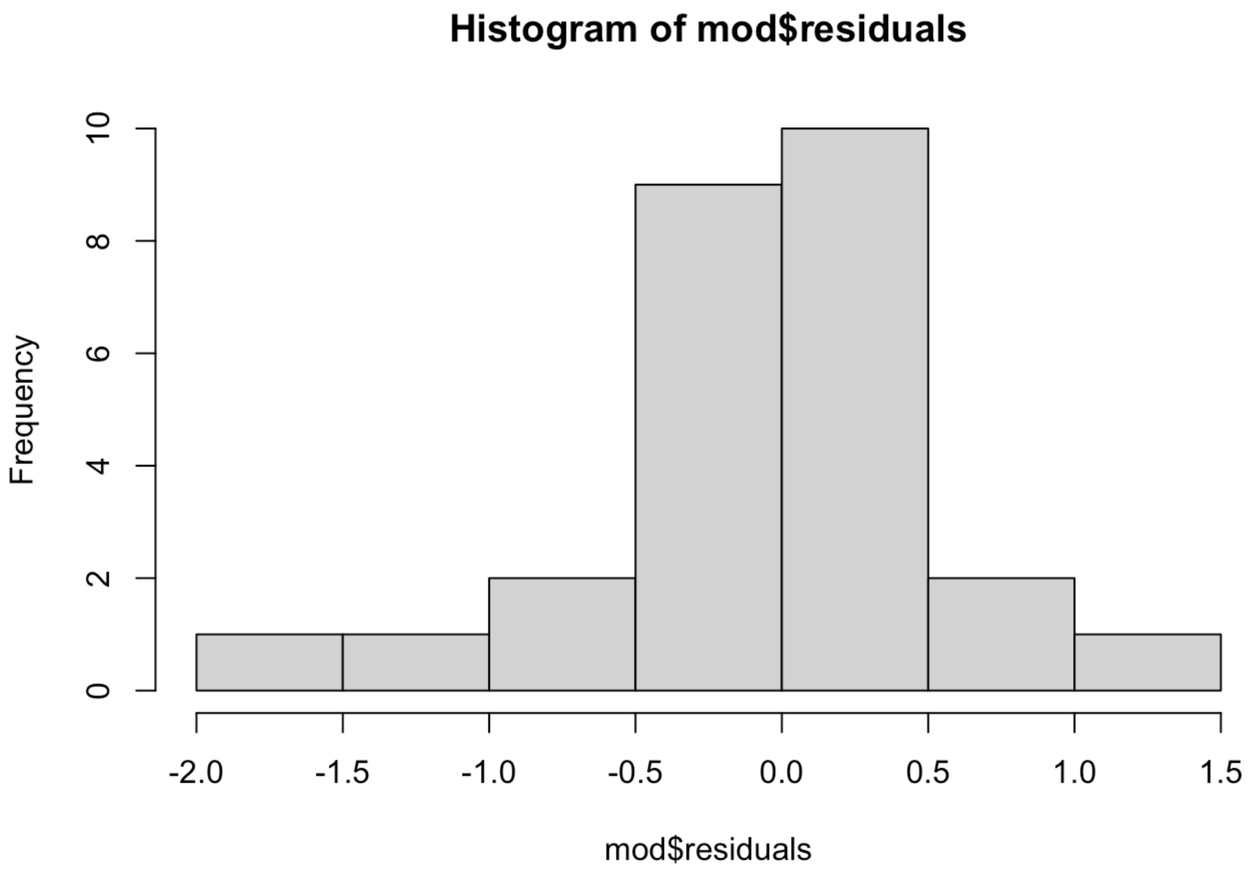


```
shapiro.test(mod$residuals) #data are non-normal
```

```
##
##  Shapiro-Wilk normality test
##
## data:  mod$residuals
## W = 0.90795, p-value = 0.02367
```

```
plot(mod, which = 3)
```

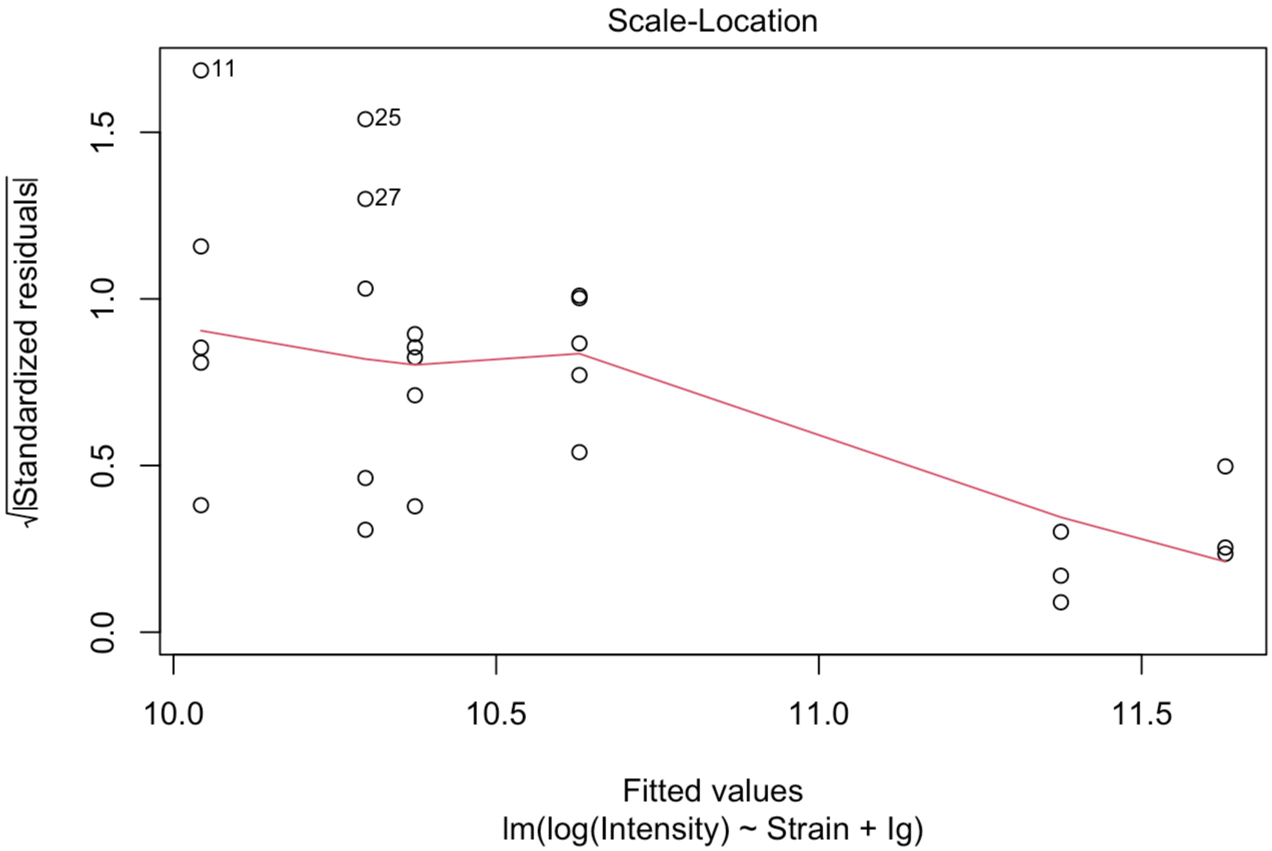


```
#leveneTest(aov(Intensity ~ Strain + Ig, data=data))

data %>%
   group_by(Strain) %>%
   dunn_test(Intensity ~ Ig) %>%
   knitr::kable()
```

Strain	.y.	group1	group2	n1	n2	statistic	p	p.adj	p.adj.signif
BALBc	Intensity	IgA	IgG	5	5	-0.5222330	0.6015081	0.6015081	ns
NOD	Intensity	IgA	IgG	5	5	-0.7311262	0.4647021	0.4647021	ns
NOR	Intensity	IgA	IgG	3	3	-1.9639610	0.0495346	0.0495346	*

Given that the data is non- normally distributed, a non-parametric post-hoc test will be done to identify the differences between group Non-parametric Dunn's test shows that for the male NOR mice, tear IgA is significantly higher than tear IgG.

7.2 Chunk for plotting mouse IgG vs IgA secondary control for each samples

```
#x1 = unlist(Tear_IgG_NSI_raw[87,2:16])
#x2 = unlist(Tear_IgG_NSI_raw[88,2:16])
#y1 = unlist(Tear_IgA_NSI_raw[87,])
#y2 = unlist(Tear_IgA_NSI_raw[88,])
Strain <- c(rep("NOD", each=5),rep("NOR", each=4), rep("BALBc", each=6))
x = unlist(lapply(Tear_IgG_NSI_raw[87:88,], mean, 2))
y = unlist(lapply(Tear_IgA_NSI_raw[87:88,], mean, 2))
data <- as.data.frame(cbind(x,y))
colnames(data) <- c("IgG","IgA")
data$Strain <- factor(Strain)
data$Sample <- rownames(data)
data <- gather(data, "Ig", "Intensity", 1:2)

tiff("Tear_IgA_vs_IgG.tiff", units="in", width=5.5, height=4.5, res=300)
setwd("~/Documents/3_Parkinsons_disease/Autoantibody_Data/Tear_Auto_Validation_2022/")
q <- ggplot(data, aes(x=Strain, y=(Intensity), fill=Ig)) +
   geom_boxplot(outlier.shape = NA, width = 0.8, coef=1, varwidth=F, show.legend = T, si
ze=0.9, position = position_dodge(0.9)) +
   geom_jitter(aes(colour = Ig), alpha =0.6, size=2.5, show.legend = T, position = posit
ion_jitterdodge(dodge.width=0.9)) +
   scale_fill_jco() +
   theme_minimal() +
   ylab("Average Intensity of Ig Controls") +
   chart_design +
   ylim(60000,67000)
print(q)
dev.off()
```

```
## quartz_off_screen
##                 2
```

```
print(q)
```

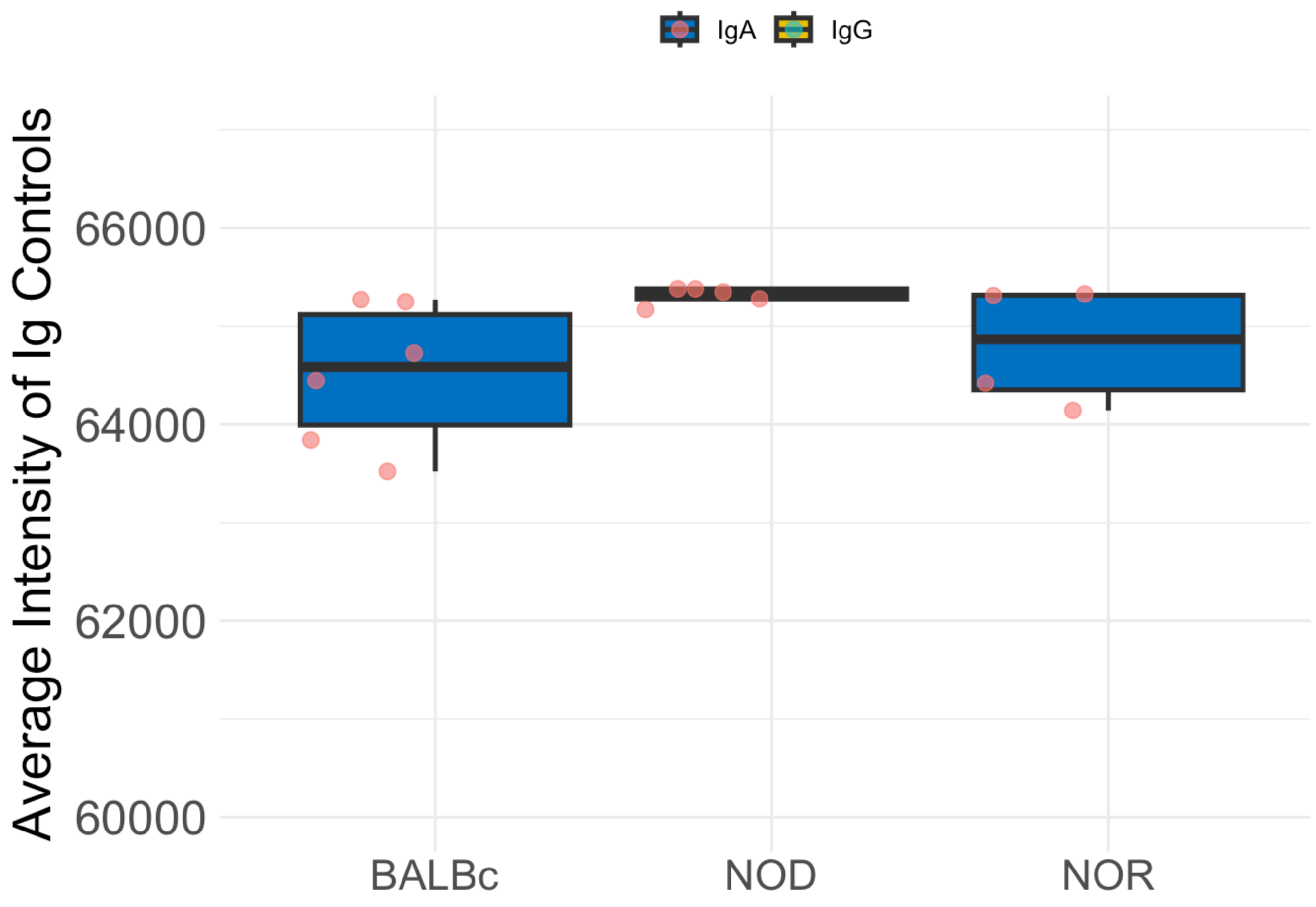


```
#+ ylim(10,12)
```

Signal intensity observed for mouse IgA secondary control was significantly higher than the intensity observed for mouse IgG secondary control in the tears of male NOD, NOR and BALB/c mice. Levels of signal intensity for either IgG or IgA were consistent between the three mouse groups.

7.2.1 Statistics for mouse IgG and IgA controls

```
#Statistics
mod <- lm((Intensity) ~ Strain*Ig, data)
aov(mod)
```

```
## Call:
##    aov(formula = mod)
##
## Terms:
##                      Strain         Ig  Strain:Ig  Residuals
## Sum of Squares      2188792 2464619432     208842  274448157
## Deg. of Freedom           2          1          2         24
##
## Residual standard error: 3381.618
## Estimated effects may be unbalanced
```

```
summary(aov(mod))
```

```
##             Df    Sum Sq   Mean Sq F value   Pr(>F)    
## Strain       2 2.189e+06 1.094e+06   0.096    0.909    
## Ig           1 2.465e+09 2.465e+09 215.527 1.73e-13 ***
## Strain:Ig    2 2.088e+05 1.044e+05   0.009    0.991    
## Residuals   24 2.744e+08 1.144e+07                     
## ---
## Signif. codes:  0 '***' 0.001 '**' 0.01 '*' 0.05 '.' 0.1 ' ' 1
```

```
TukeyHSD(aov(mod))
```

```
##   Tukey multiple comparisons of means
##     95% family-wise confidence level
## 
## Fit: aov(formula = mod)
## 
## $Strain
##                diff       lwr      upr     p adj
## NOD-BALBc  617.6833 -2998.194 4233.561 0.9049489
## NOR-BALBc  145.2708 -3709.268 3999.809 0.9951292
## NOR-NOD   -472.4125 -4478.166 3533.341 0.9534036
## 
## $Ig
##              diff       lwr       upr p adj
## IgG-IgA -18127.77 -20676.25 -15579.28     0
## 
## $`Strain:Ig`
##                            diff        lwr        upr     p adj
## NOD:IgA-BALBc:IgA      802.2917  -5528.967   7133.550 0.9986460
## NOR:IgA-BALBc:IgA      291.7292  -6457.415   7040.874 0.9999932
## BALBc:IgG-BALBc:IgA -17926.5833 -23963.202 -11889.965 0.0000000
## NOD:IgG-BALBc:IgA   -17493.5083 -23824.767 -11162.250 0.0000001
## NOR:IgG-BALBc:IgA   -17927.7708 -24676.915 -11178.626 0.0000003
## NOR:IgA-NOD:IgA       -510.5625  -7524.479   6503.354 0.9999093
## BALBc:IgG-NOD:IgA   -18728.8750 -25060.134 -12397.616 0.0000000
## NOD:IgG-NOD:IgA     -18295.8000 -24908.584 -11683.016 0.0000001
## NOR:IgG-NOD:IgA     -18730.0625 -25743.979 -11716.146 0.0000003
## BALBc:IgG-NOR:IgA   -18218.3125 -24967.457 -11469.168 0.0000002
## NOD:IgG-NOR:IgA     -17785.2375 -24799.154 -10771.321 0.0000006
## NOR:IgG-NOR:IgA     -18219.5000 -25612.817 -10826.183 0.0000010
## NOD:IgG-BALBc:IgG      433.0750  -5898.184   6764.334 0.9999333
## NOR:IgG-BALBc:IgG       -1.1875  -6750.332   6747.957 1.0000000
## NOR:IgG-NOD:IgG       -434.2625  -7448.179   6579.654 0.9999593
```

```
plot(mod, which = 2)
```

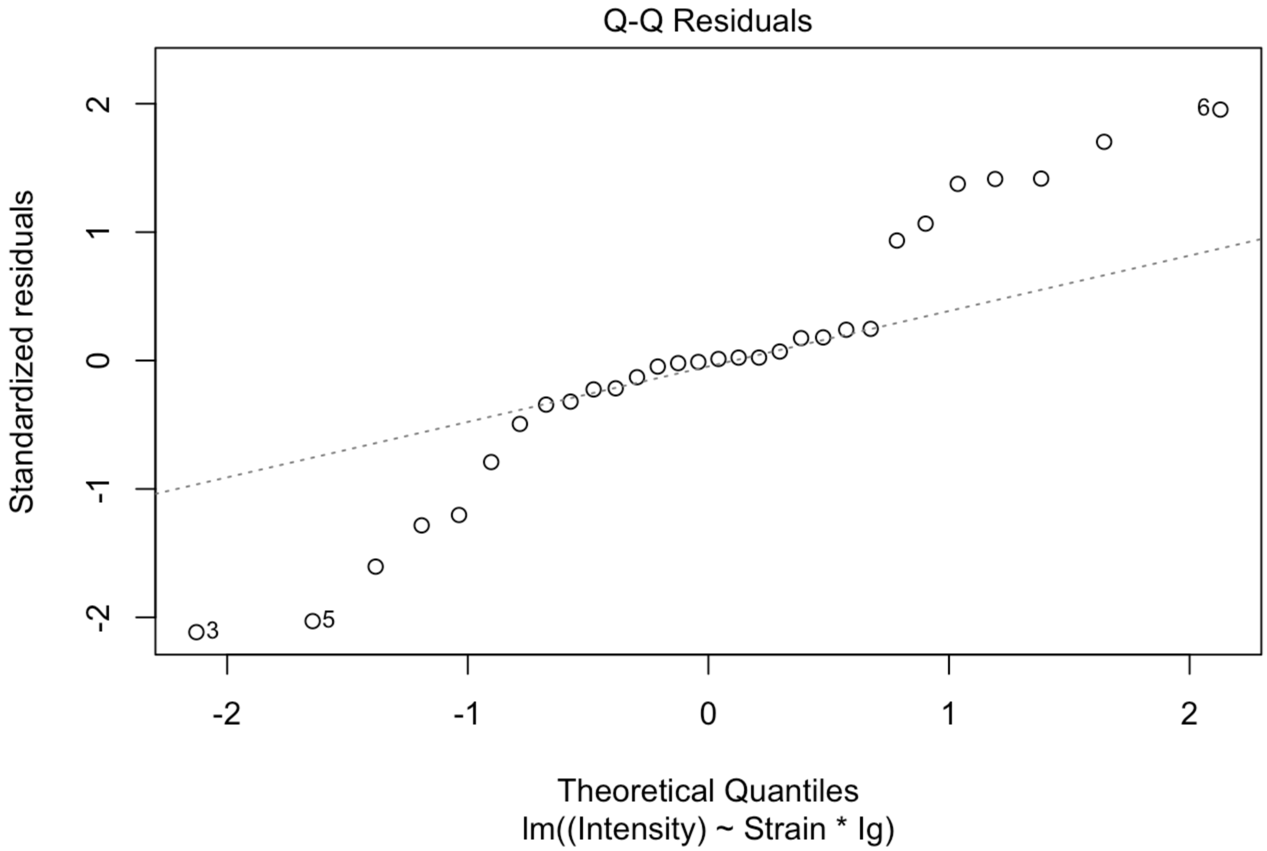


```
hist(mod$residuals)
```

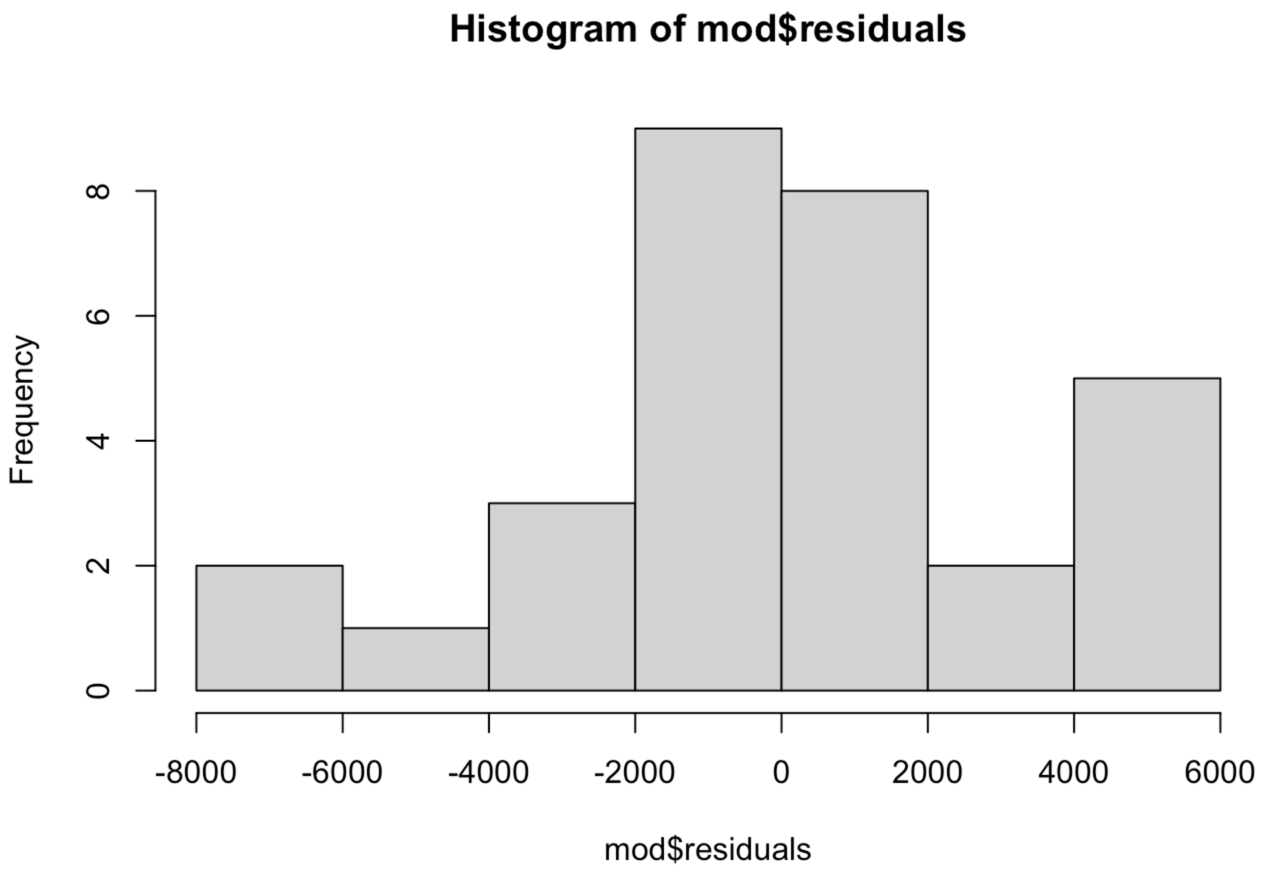


```
shapiro.test(mod$residuals) #data are normally distributed
```

```
##
##  Shapiro-Wilk normality test
##
## data:  mod$residuals
## W = 0.94833, p-value = 0.1525
```

```
plot(mod, which = 3)
```

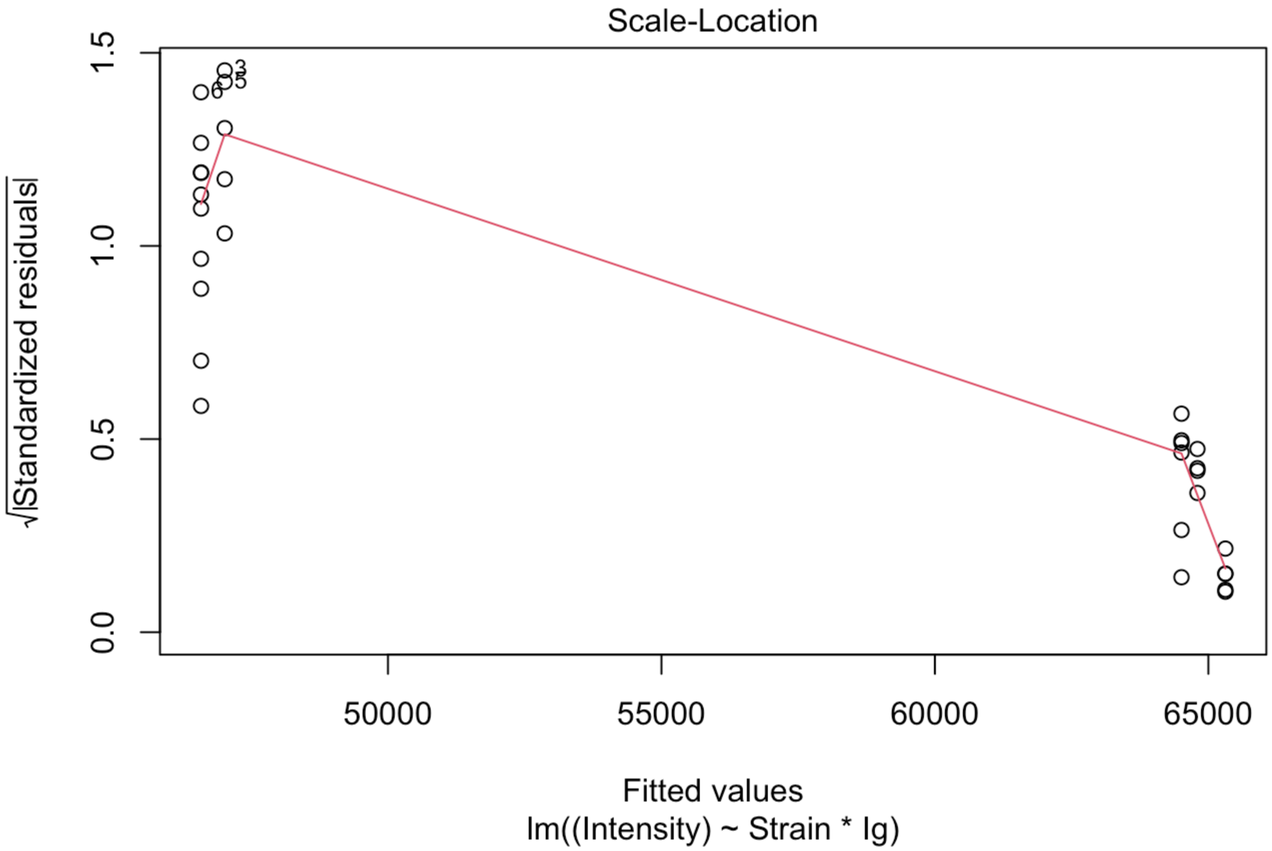

8. Tear IgG vs IgA Correlation Analysis

Scatterplots showing raw signal intensity for IgG vs IgA of each autoantigen in the tears of NOD, NOR and BALB/c mice.

```
#which((rowSums(Tear_IgG_SNR_raw[1:80,1:15])/15) < 2.8)
#which((rowSums(IgA_SNR[1:80,])/15) < 2.8)

Tear_IgA_NSI <- Tear_IgA_NSI_raw[1:80,]
Tear_IgA_NSI <- Tear_IgA_NSI[-c(which((rowSums(Tear_IgG_SNR_raw[1:80,1:15])/15) < 2.8 &
(rowSums(IgA_SNR[1:80,])/15) < 2.8)),]
Tear_IgA_NSI$Antigen <- rownames(Tear_IgA_NSI)
Tear_IgA_NSI <- gather(Tear_IgA_NSI, "Sample", "Intensity", 1:(ncol(Tear_IgA_NSI)-1))
Tear_IgA_NSI$Strain <- str_sub(Tear_IgA_NSI$Sample, 1, (str_locate(Tear_IgA_NSI$Sample,
"_")[,1] -1))
Tear_IgA_NSI$Ig <- "IgA"

Tear_IgG_NSI <- Tear_IgG_NSI_raw[1:80,]
Tear_IgG_NSI <- Tear_IgG_NSI[-c(which((rowSums(Tear_IgG_SNR_raw[1:80,])/15) < 2.8 & (row
Sums(IgA_SNR[1:80,])/15) < 2.8)),]
Tear_IgG_NSI$Antigen <- rownames(Tear_IgG_NSI)
Tear_IgG_NSI <- gather(Tear_IgG_NSI, "Sample", "Intensity", 1:(ncol(Tear_IgG_NSI)-1))
Tear_IgG_NSI$Strain <- str_sub(Tear_IgG_NSI$Sample, 1, (str_locate(Tear_IgG_NSI$Sample,
"_")[,1] -1))
Tear_IgG_NSI$Ig <- "IgG"

data_mat <- dplyr::bind_rows(Tear_IgG_NSI, Tear_IgA_NSI)

###Plot for NODs & NORs
data_mat2 <- dplyr::full_join(Tear_IgA_NSI, Tear_IgG_NSI, by = c("Antigen", "Sample"),su
ffix = c(".IgA", ".IgG"),)

### Plot for NORs
ggplot(data = data_mat2[which(data_mat2$Strain.IgA=="NOR"),]) +
   geom_point(aes(x=(Intensity.IgA), y=(Intensity.IgG), fill=factor(Antigen), color=fact
or(Antigen)), show.legend = F) +
   facet_wrap(~factor(Antigen))
```

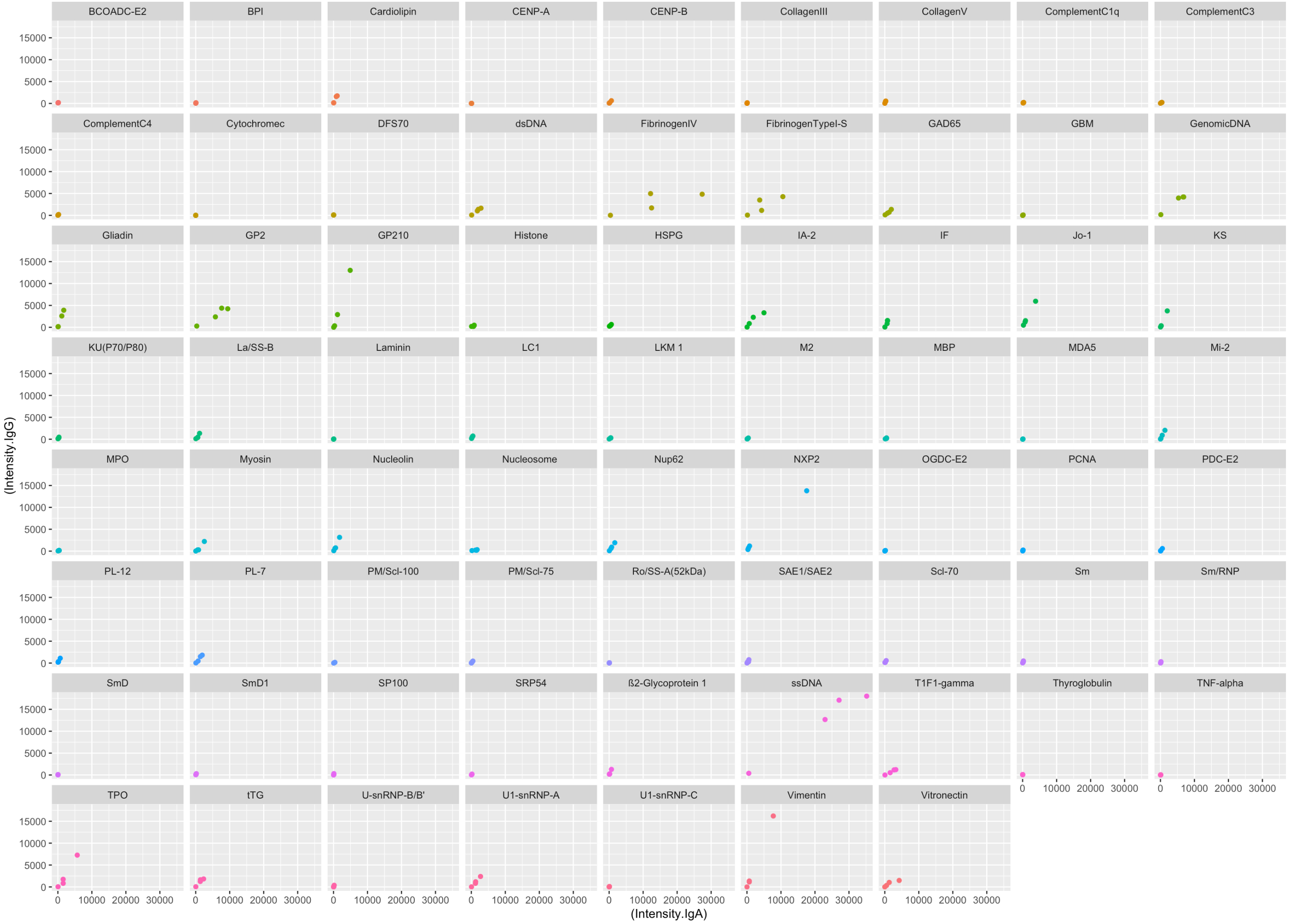

There is a need to normalize the NSI on x and y axes. In the next plot, we run correlation on log10 normalized NSI for IgG and IgA.

8.1 Correlation Scatterplots of log 10 IgG NSI vs log log IGA NSI

8.2 Correlation Analysis Figure

Sctterplots of IgG vs IgA for NOD, NOR and BALB/c. Each point represents one mouse. Spearman correlation coefficient rho and p-values are shown. $p < 0.01$ is considered significant.

```
### Plot for NOD, NOR & BALB/c
data_mat2$model <- ifelse(data_mat2$Strain.IgA=="BALBc", "Healthy", "SjD")

p <- ggplot(data = data_mat2, aes(x=log10(Intensity.IgA+1), y=log10(Intensity.IgG+1))) +
   geom_point(aes( color=factor(model), fill=factor(Antigen)), show.legend = F, alpha =
0.8) +
   geom_smooth(aes(color = factor(model)),method=lm, se=FALSE, fullrange=FALSE, linewidt
h=0.25) +
   scale_color_jco(guide="legend") +
   guides(fill="none") +
   theme_minimal() + ylim(-1, 4.6) +
   xlab("Log 10 IgA NSI") +
   ylab("Log 10 IgG NSI") +
   stat_cor(aes(color=factor(model)), method = "spearman", r.digits=2, p.accuracy=0.000
5, label.y = c(-0.9,4.4), cor.coef.name="rho") +
   facet_wrap(~factor(Antigen),nrow=7) +
   chart_design

print(p)
```

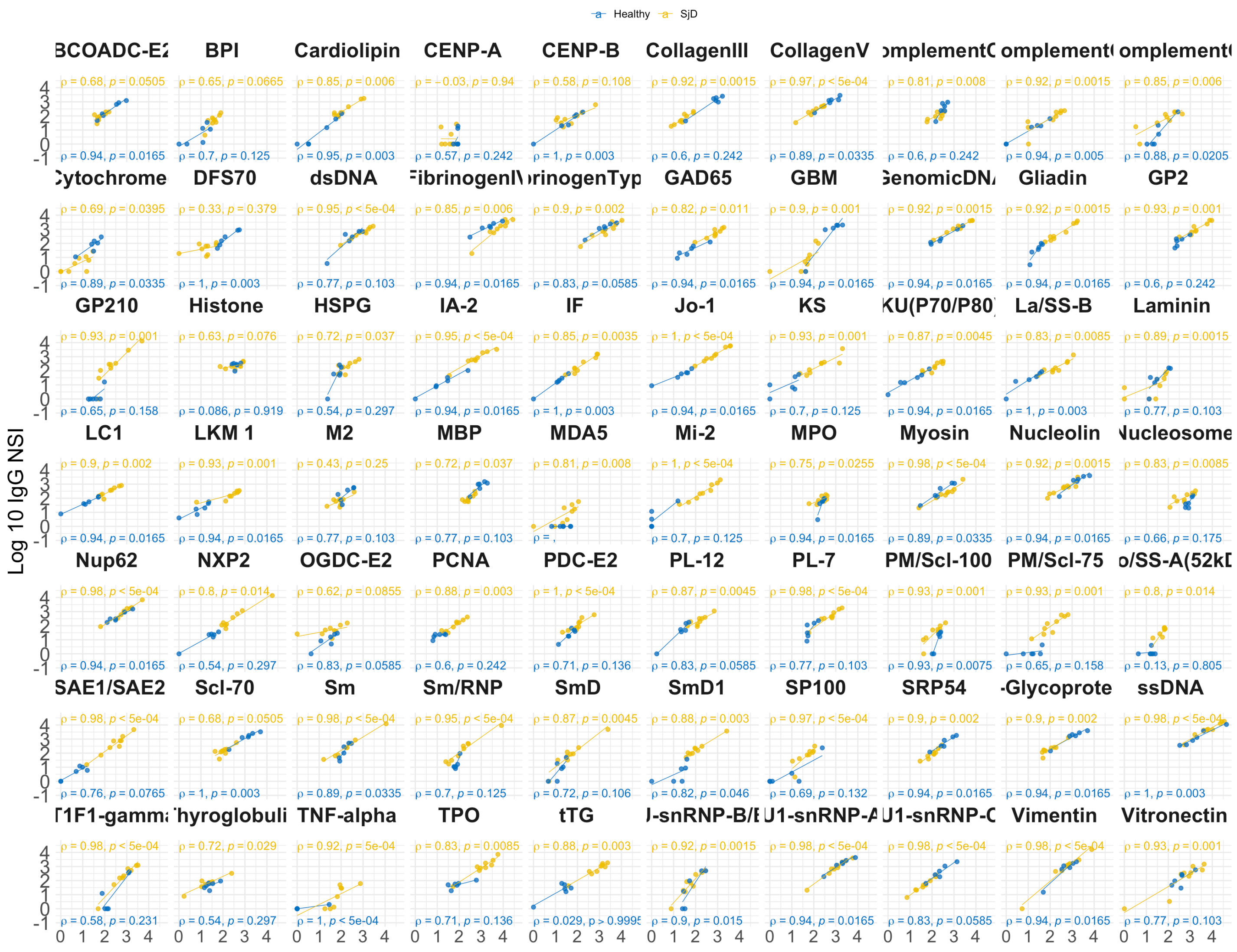

```
setwd("~/Documents/3_Parkinsons_disease/Autoantibody_Data/Tear_Auto_Validation_2022")
tiff("All_IgGvsIgA_Tears.jpeg", units="in", width=16, height=10, res=300)
print(p)
dev.off()
```

```
## quartz_off_screen
##                 2
```

8.3 Scatterplot of shared epitopes between upregulated IgG and IgA autoantibodies

Scatterplot of Log10 IgG vs Log10 IgA NSI of shares epitopes of autoantibodies that are significantly upregulated in male NOD and male NOR mice tears when compared to BALB/c controls. NOD and NOR are shown in yellow (referred to an SjD) and BALB/c are shown in blue (referred to as Healthy).

```
data_mat3 <- data_mat2[which(data_mat2$Antigen %in% c("tTG", "TPO", "Mi-2", "Jo-1", "IA-
2", "SAE1/SAE2 ")),]
p <- ggplot(data = data_mat3, aes(x=log10(Intensity.IgA+1), y=log10(Intensity.IgG+1))) +
   geom_point(aes( color=factor(model), fill=factor(Antigen)), show.legend = T, alpha =
0.8) +
   geom_smooth(aes(color = factor(model)),method=lm, se=FALSE, fullrange=FALSE, linewidt
h=0.25) +
   scale_color_jco(guide="legend") +
   guides(fill="none") +
   theme_minimal() + ylim(-1, 4.6) +
   xlab("Log 10 IgA NSI") +
   ylab("Log 10 IgG NSI") +
   stat_cor(aes(color=factor(model)), method = "spearman", r.digits=2, p.accuracy=0.000
5, label.y = c(-0.9,4.4), cor.coef.name="rho") +
   facet_wrap(~factor(Antigen),nrow=2) +
   chart_design

print(p)
```

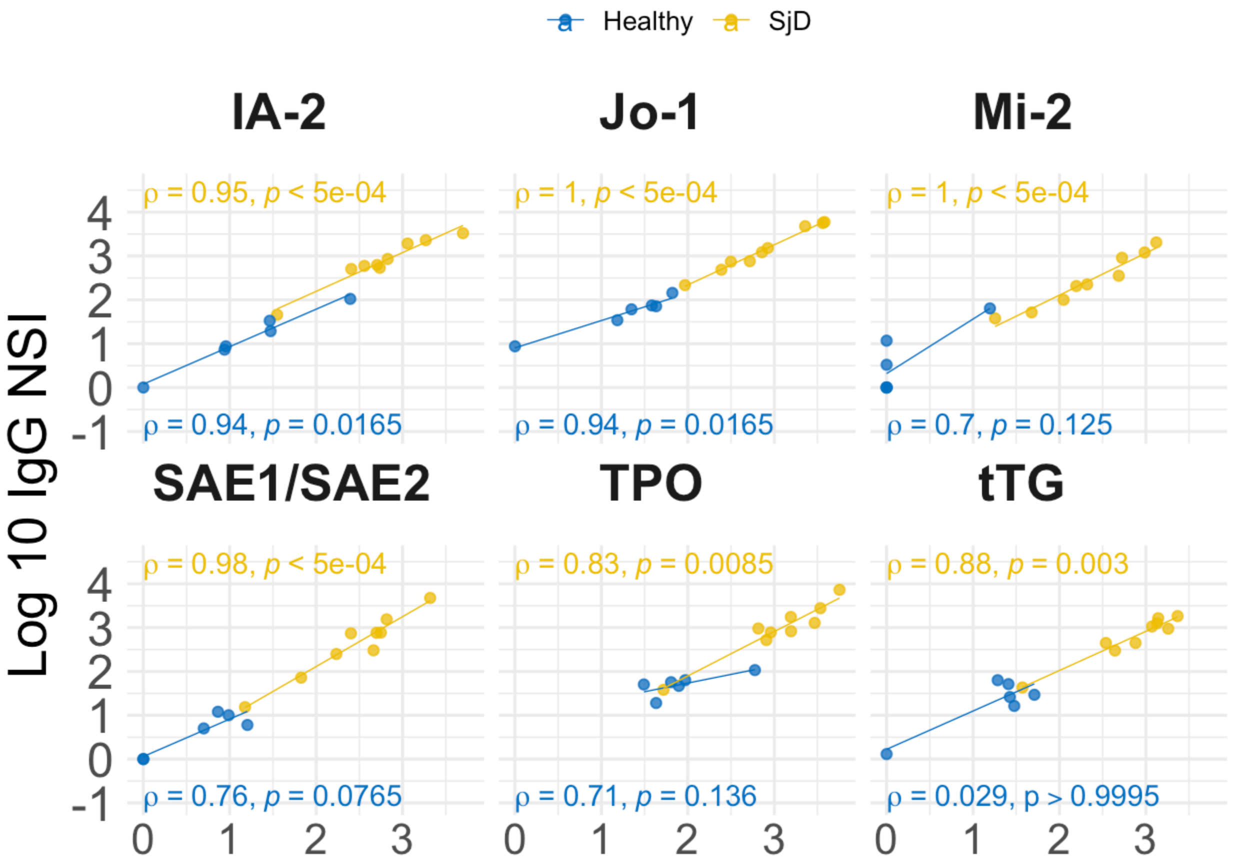


```
setwd("~/Documents/3_Parkinsons_disease/Autoantibody_Data/Tear_Auto_Validation_2022")
tiff("Supp_Figure_6.jpeg", units="in", width=6.5, height=4.8, res=300)
print(p)
dev.off()
```

```
## quartz_off_screen
##                 2
```
