# Supplementary material for: Serum and tear autoantibodies from NOD and NOR mice as potential diagnostic indicators of local and systemic inflammation in Sjögren’s disease
Source: Front Immunol. 2025 Jan 28;15:1516330. doi: 10.3389/fimmu.2024.1516330 (PMC11810956; doi:10.3389/fimmu.2024.1516330)
Supplement: Supplementary file 6 [file DataSheet6.pdf]

Supplemental Methods 1.5-1.6

Serum_IgA_2022 & Serum_IgG_2022

Shruti Kakan

2024-11-27

- Serum IgA Analysis
  - Adding Auto-antigen ID Names
  - Defining Column Metadata
  - Filtering Data based on low Signal to Noise Ratio (SNR)
  - Limma Normalization and DE analysis (least squares method)
  - Limma Voom Normalization & DE Analysis
  - QC plots: Serum IgA Limma Voom
  - R-Squared goodness of fit for Serum IgA
  - Limma Voom Differentially Expressed IgA auantibodies - Table 2
  - Boxplots from voom normalized counts
  - Combined Boxplot of Serum IgA (Supp Figure 7)
- Serum IgG Analysis
  - Reading in Raw IgG Datasets
  - Filtering Data based on low Signal to Noise ratio and Signal Intensity
  - Visualizing Filtered Data
  - Limma Least Squares Normalization
  - QC Plots: Serum IgG Limma Robust
  - Density plots post normalization
  - Upregulated Serum IgG Autoantibodies - Table 2
  - Boxplots from library normalized counts
  - Study 1 Validation plot - Figure 2B
  - Common to tears and serum IgG - Supplemental Figure 5B
  - Supplemental Figure 3

Loading Libraries

```
library(car)
library(lsmeans)
library(calibrate)
library(dplyr)
#library(DEGreport)
library(DESeq2)
library(DEFormats)
library(edgeR)
library(ggpubr)
library(ggsci)
library(ggplot2)
library(gridExtra)
library(pheatmap)
library(reshape2)
library(RColorBrewer)
library(scales)
library(rstatix)
library(tidyr)
library(magrittr)
#library(PCAtools)
library(tidyverse)
library(Biobase)
#library(marray)
library(limma)
library(gplots)

library(devtools)
#install_github("dpgaile/AutoAntArrayExmpl")
library(AutoAntArrayExmpl)

library(devtools)
#install_github("dpgaile/AutoAntArrayExmpl")
#devtools::install_github('renozao/NMF@devel')
library(AutoAntArrayExmpl)
library(NMF)
library(quantreg)
library(asbio)
library(fdrtool)
#library(discreteMTP)
library(scales)
library(ggsci)
library(ggplot2)
```

Serum IgA Analysis

Adding Auto-antigen ID Names

This chunk corrects for the special character beta in the labeling of antigen beta-glycoprotein 1

```
#Adding Auto-antigen ID names
Antigen_ID <- read.csv("~/Documents/3_Parkinsons_disease/Autoantibody_Data/Tear_Auto_Val
idation_2022/Antigen_ID.csv",  header=T)[1:80,1:2]

#colnames(IgA_NSI)[1] <- colnames(Antigen_ID)[2]
#colnames(IgA_SNR)[1] <- colnames(Antigen_ID)[2]

IgA_NSI[68,"ID"] <- Antigen_ID[68,"ID"]
IgA_SNR[68,"ID"] <- Antigen_ID[68,"ID"]
IgA_NSI <- full_join(IgA_NSI, Antigen_ID, by="ID")
IgA_SNR <- full_join(IgA_SNR, Antigen_ID, by="ID")
rownames(IgA_NSI) <- IgA_NSI$Antigen_ID
rownames(IgA_SNR) <- IgA_SNR$Antigen_ID

IgA_NSI <- IgA_NSI[,-c(1, 17)]
IgA_SNR <- IgA_SNR[,-c(1, 17)]
```

Defining Column Metadata

We have serum samples from 5 male NOD, 4 male NOR and 6 male BALB/c mice

```
Strain <- c(rep("NOD", each=5),rep("NOR", each=4), rep("BALBc", each=6))

colData <- as.data.frame(cbind(c(colnames(IgA_NSI)), Strain))
colnames(colData) <- c('Sample', "Strain")
rownames(colData) <- colData$Sample
colData$Strain <- factor(colData$Strain)
#colData$Strain <- relevel(colData$Strain, ref = "BALBc")
#Biofluid <- c( rep("Tear", each=11), rep("Serum", each=11))
#colData <- as.data.frame(cbind(c(colnames(IgA_NSI)), Strain, Biofluid))
```

Filtering Data based on low Signal to Noise Ratio (SNR)

Here, we have calculated the means and medians for each antigen based on sample group, and selected the highest SNR/NSI row median and mean of the three groups - NOD, NOR and BALB/c. This max values is then used to filter out rows with very low intensity values and very high noise.

This ensures that rows with group specific expression are not excluded due to a low overall row mean or row median.

```
head(IgA_NSI)
```

```
##             NOD_M1_serum NOD_M3_serum NOD_M4_serum NOD_M5_serum NOD_M6_serum
## ACAN                 3.3          8.3          6.3           10         12.3
## AGTR1                0.0          0.0          0.0            0          0.0
## BCOADC-E2            0.0          0.0          0.0            0         23.7
## BPI                  0.0          0.0          0.0            0          0.0
## Cardiolipin          0.0          0.0          0.0            0          0.0
## CENP-A               0.0          0.0          0.0            0         34.3
##             NOR_M1_serum NOR_M2_serum NOR_M3_serum NOR_M4_serum BALBc_M1_serum
## ACAN                14.7          7.0          9.3          8.3           15.0
## AGTR1                2.3          0.0          0.3          0.0            0.0
## BCOADC-E2           15.0         39.7         40.7         67.7           62.0
## BPI                  0.0          3.7          0.0         14.3            0.0
## Cardiolipin          0.0          0.0         10.7          0.0            0.0
## CENP-A              49.7         80.0         62.0        125.7           79.7
##             BALBc_M2_serum BALBc_M3_serum BALBc_M4_serum BALBc_M5_serum
## ACAN                  22.7           19.3           13.3           16.0
## AGTR1                  0.0            0.3            0.0            0.0
## BCOADC-E2            113.0           81.3           89.3           78.7
## BPI                   30.7            7.3           21.0           18.3
## Cardiolipin           34.7            0.0            0.0            0.0
## CENP-A               233.7          108.7          172.3          152.3
##             BALBc_M6_serum
## ACAN                  14.7
## AGTR1                  0.0
## BCOADC-E2             76.0
## BPI                   15.0
## Cardiolipin            0.0
## CENP-A               191.7
```

```
IgA_raw=list()
IgA_SNR$average <- apply(cbind(rowMeans(as.matrix(IgA_SNR)[,2:9]), rowMeans(as.matrix(Ig
A_SNR)[,10:14])), 1, max)
IgA_SNR$med <- apply(cbind(rowMedians(as.matrix(IgA_SNR)[,2:9]), rowMedians(as.matrix(Ig
A_SNR)[,10:14])), 1, max)

IgA_NSI$average <- apply(cbind(rowMeans(as.matrix(IgA_NSI)[,2:9]), rowMeans(as.matrix(Ig
A_NSI)[,10:14])), 1, max)
IgA_NSI$med <- apply(cbind(rowMedians(as.matrix(IgA_NSI)[,2:9]), rowMedians(as.matrix(Ig
A_NSI)[,10:14])), 1, max)


IgA_raw$NSI <- as.matrix(IgA_NSI[which(IgA_SNR$med>3 & IgA_NSI$med>5),])[,1:15]
IgA_raw$SNR <- as.matrix(IgA_SNR[which(IgA_SNR$med>3 & IgA_NSI$med>5),])[,1:15]
```

After filtering, we are left with 43 antigens. 37 rows have been filtered out.

Visualizing Filtered Data

```
dataN <- list()
dataN[["Raw"]]<- log2(IgA_NSI[,1:15]  + 0.5)
dataN[["filtered"]] <- log2(IgA_raw$NSI + 0.5)

for (i in 1:2) {
   countData = as.data.frame(dataN[[i]])
   df_dseq = melt(countData, variable.name = "Samples", value.name = "count")# reshape t
he matrix
   mycolors <- colorRampPalette(brewer.pal(8, "Set1"))(15)
   p <- ggplot(df_dseq, aes(x = count, color=Samples)) +
      geom_density(alpha = 0.5, size = 0.8) +
   #facet_wrap(~Strain, ncol=2) +
      theme_minimal() + #xlim(-1.5,6) +
      scale_colour_manual(values=mycolors, name="") +
      guides(fill="none") +
      labs(title=names(dataN)[i])
   plot(p)
}
```

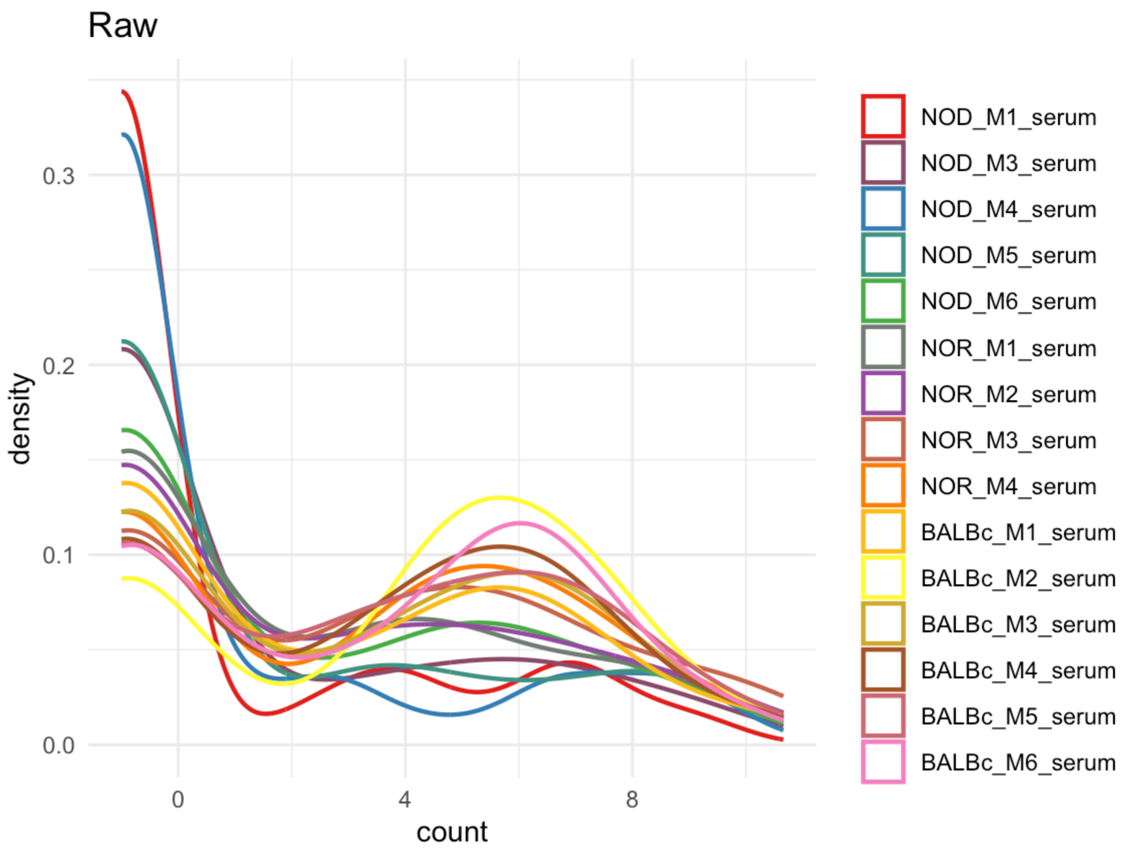

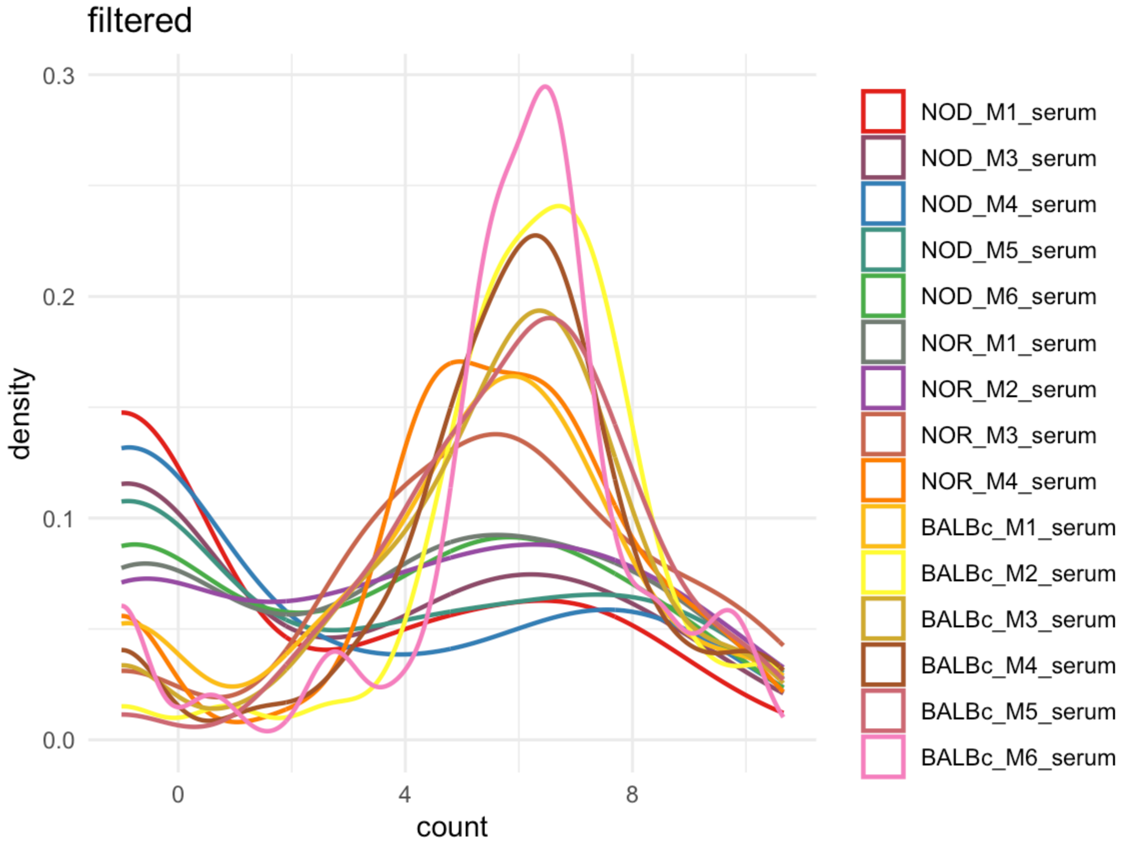

NOD M4 appears to be the most variable, and was considered for exclusion. Among controls, BALB M6 has the most extreme distribution

Boxplots of Raw and SNR<3 Filtered Data

```
#boxplot(as.data.frame((dataN$filtered)),main="IgA normalization")#,col=Sample)

dataN <- (IgA_NSI[,1:15] )
countData = as.data.frame(dataN)
boxplot(as.data.frame((dataN)),main="Raw IgA NSI")
```

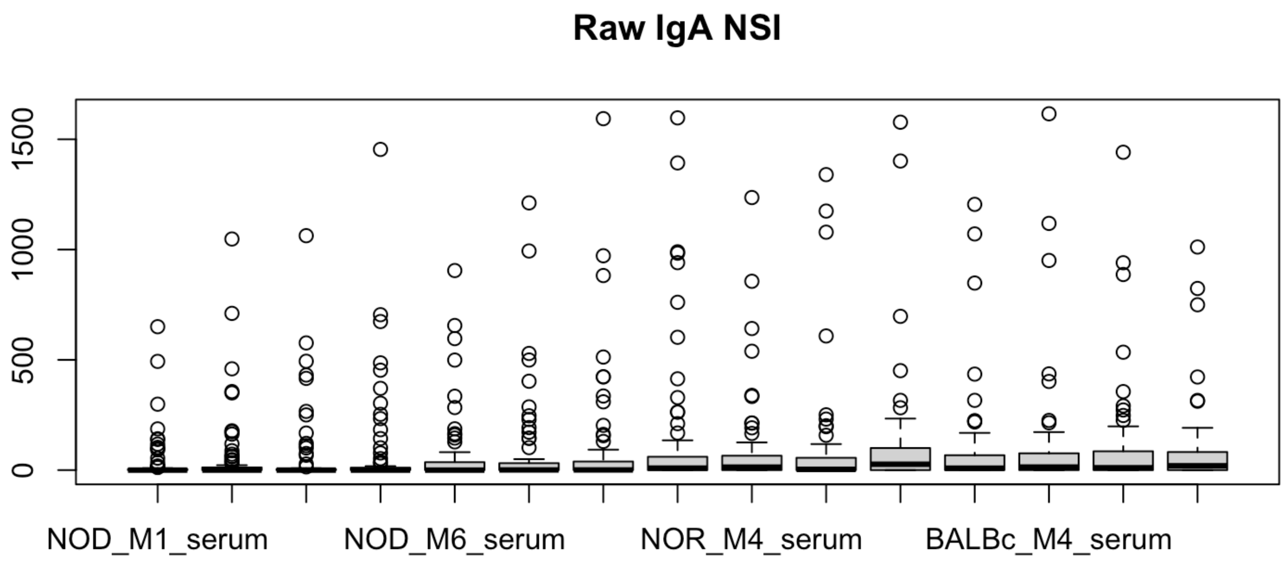


```
dataN <- log2(IgA_raw$NSI  +0.5)
countData = as.data.frame(dataN)
boxplot(as.data.frame((dataN)),main="Log IgA NSI (filtered)")
```

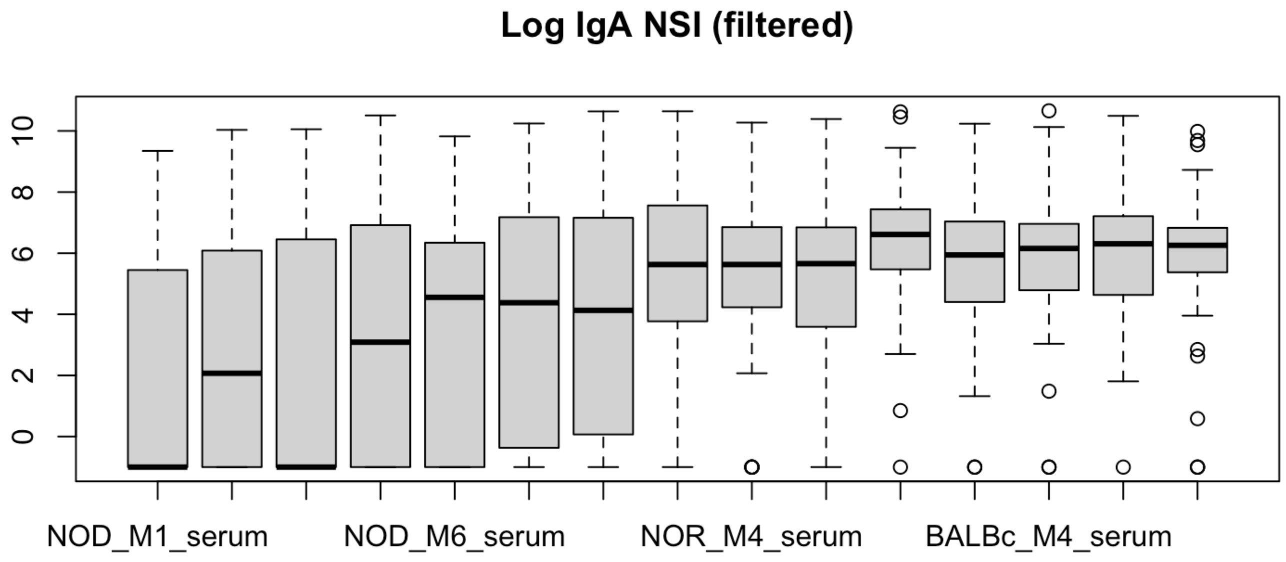

IgA levels appear to be much lower in serum than in tears, especially in male NOD mice. Despite filtering, samples NOD M1 and NOD M4 appear to be highly variable with low signal intensity for most autoantigens.

Limma Normalization and DE analysis (least squares method)

```
library(limma)
dataN <- log2(IgA_raw$NSI +
                0.5)[,]
mydata <- as.matrix(dataN)

conditions<- paste(colData$Strain[],sep=".")
conditions <- factor(conditions, levels=unique(conditions))
design <- model.matrix(~0+ conditions)
colnames(design) <- levels(conditions)
fit <- lmFit(mydata, design, plot=TRUE, method="ls")

#plot.new()

cont.matrix<- makeContrasts(
   NTvBT =  NOD - BALBc,
   nTvBT = NOR - BALBc,
   levels = design)
fit.cont<- contrasts.fit(fit, cont.matrix)
fit.cont<- eBayes(fit.cont, robust=TRUE)
plotMDS(fit, col=as.numeric(conditions))
```

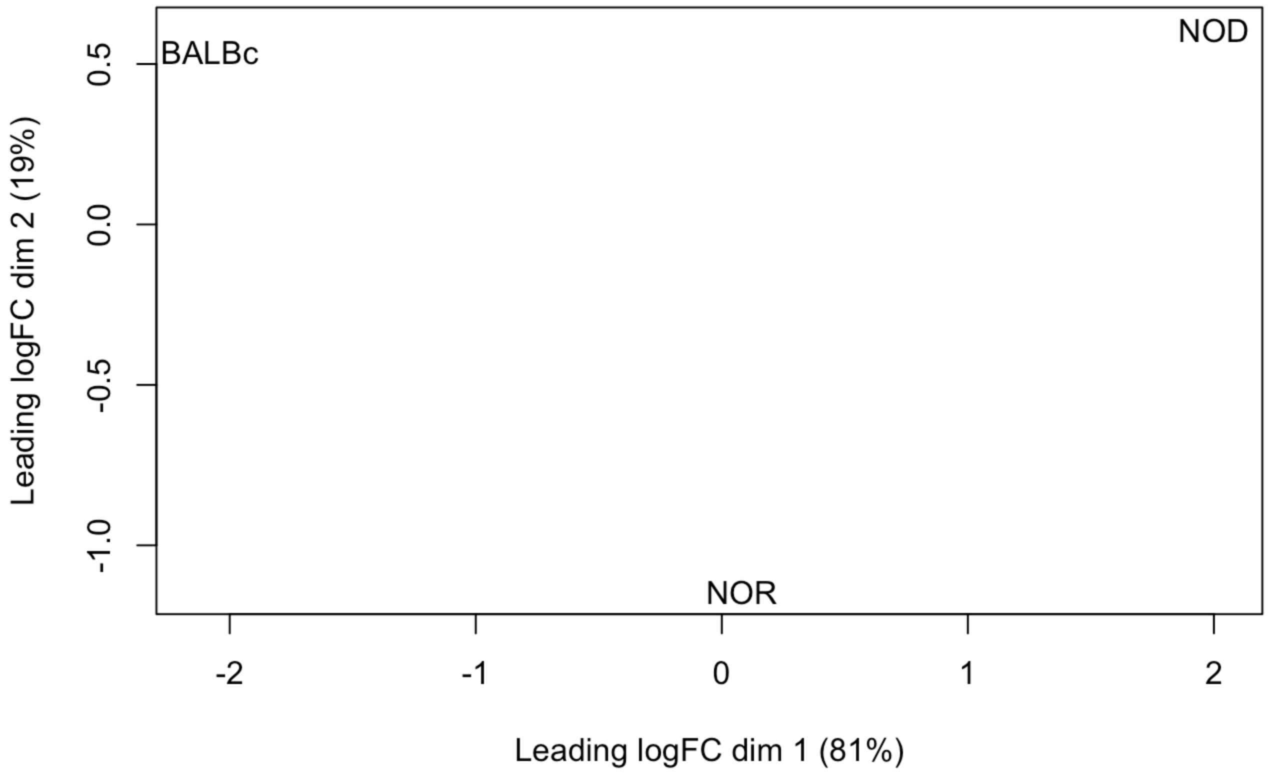

```
#qqt(fit.cont$t,df=fit.cont$df.prior+fit.cont$df.residual)
qqnorm(fit.cont$t,df=fit.cont$df.prior+fit.cont$df.residual)
```

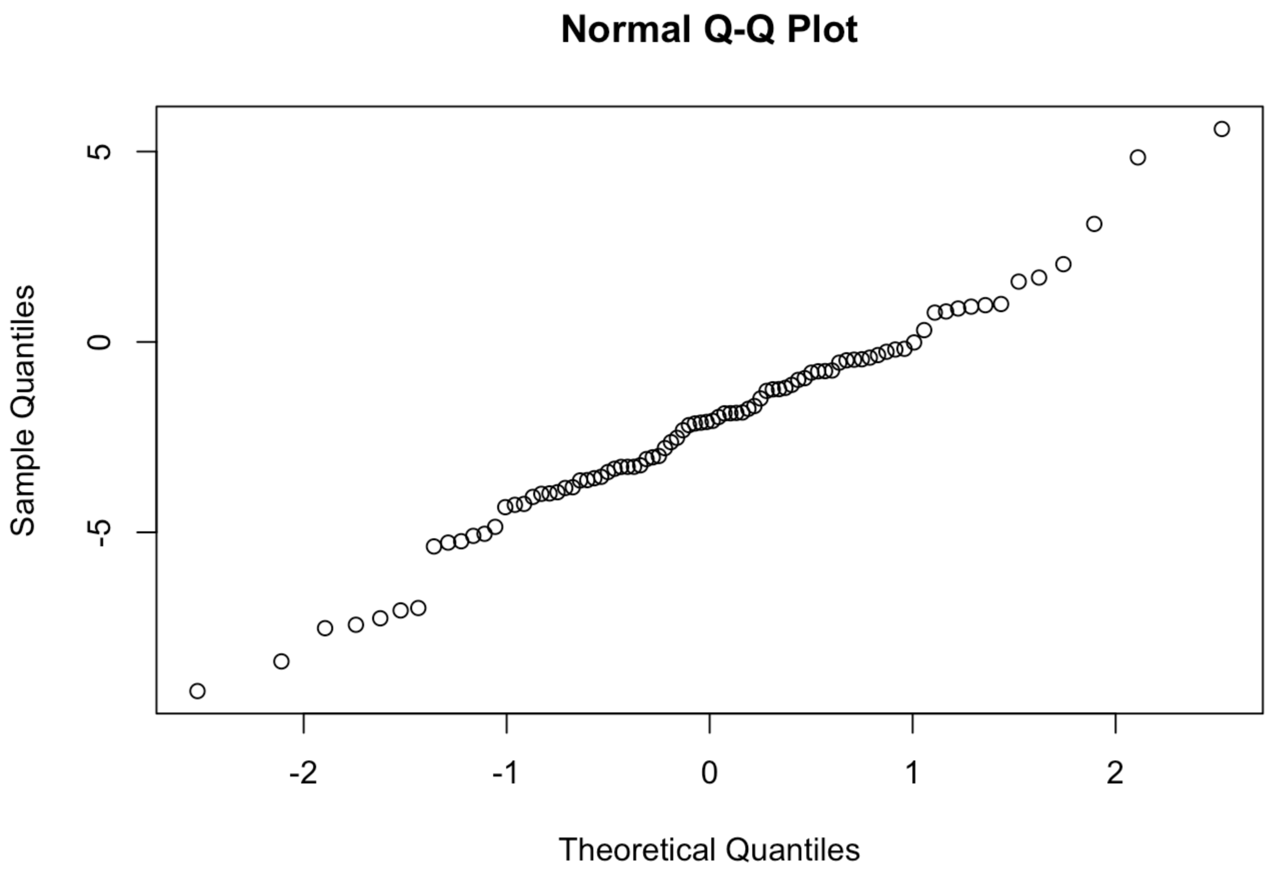

Differentially Expressed IgA Autoantibodies in Serum

```
NTvsBT <- topTable(fit.cont, coef=1, number=30, adjust.method = 'BH')
nTvsBT <- topTable(fit.cont, coef=2, number=30, adjust.method = 'BH')
NTvsBT$Antigen <- row.names(NTvsBT)
nTvsBT$Antigen <- row.names(nTvsBT)
NTvnTvBT <- full_join(NTvsBT, nTvsBT, by="Antigen",suffix = c(".NOD", ".NOR"))
rownames(NTvnTvBT) <- NTvnTvBT$Antigen
NTvnTvBT[is.na(NTvnTvBT)] <- 0.5
NTvnTvBT <- NTvnTvBT[c(which(NTvnTvBT$adj.P.Val.NOD < 0.1 & NTvnTvBT$adj.P.Val.NOR < 0.1
& NTvnTvBT$logFC.NOD > 0.5)),]

NTvnTvBT
```

```
##    logFC.NOD AveExpr.NOD    t.NOD P.Value.NOD adj.P.Val.NOD   B.NOD Antigen
## IF  5.045289    3.269836 3.099317 0.008050684    0.01442414 -2.6545      IF
##    logFC.NOR AveExpr.NOR    t.NOR  P.Value.NOR adj.P.Val.NOR    B.NOR
## IF  9.705272    3.269836 5.592796 7.300902e-05   0.003139388 1.888283
```

Only IF appears to be up-regulated significantly in serum of both NOD & NOR mice.

Limma Voom Normalization & DE Analysis

```
###### Voom normalization with quantiles
conditions<- paste(colData$Strain[],sep=".")
conditions <- factor(conditions, levels=unique(conditions))
design <- model.matrix(~0+ conditions)
colnames(design) <- levels(conditions)

v <- voom(counts=(IgA_raw$NSI[,] ), design, plot=TRUE, normalize="cyclicloess")
```

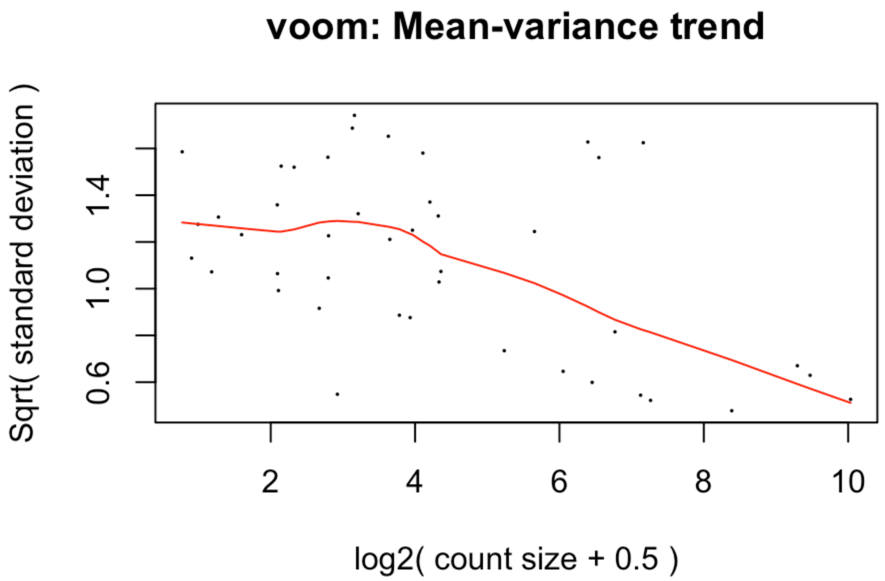


```
#v <- voom(dae, design, plot=TRUE)
fit <- lmFit(v, design)
fit.cont<- contrasts.fit(fit, cont.matrix)
fit.cont<- eBayes(fit.cont, robust=FALSE)
#topTable(fit.cont, number=20)
#topTable(fit, coef=ncol(design))
```

QC plots: Serum IgA Limma Voom

```
plotMDS(v,col=as.numeric(Strain))
```

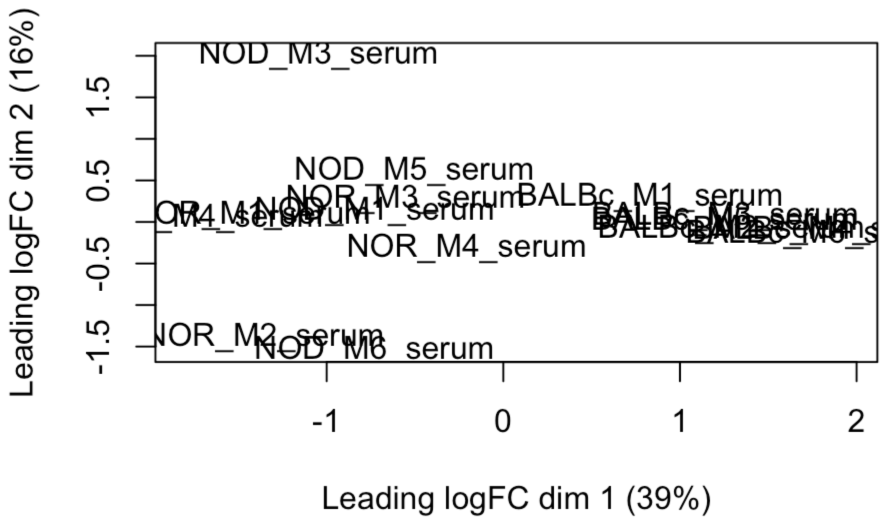


```
qqt(fit.cont$t,df=fit.cont$df.prior+fit.cont$df.residual)
```

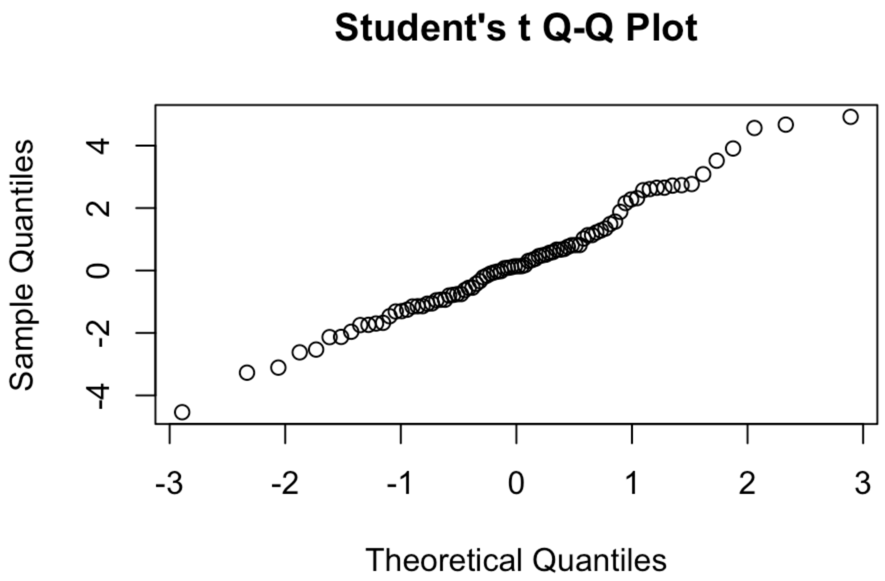


```
plotDensities(v, group=Strain, col=c("orange","green", "blue"), log=TRUE)
```

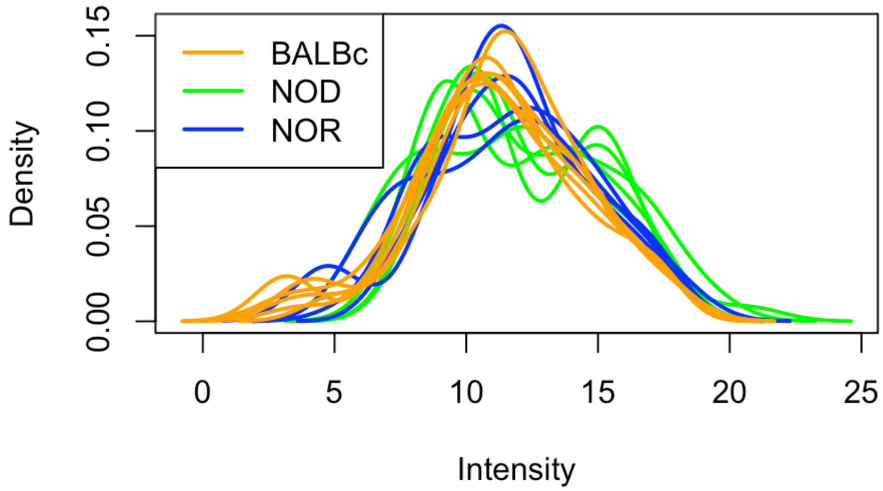

Density plots post voom normalization

```
df_dseq = melt(v$E, variable.name = "Samples", value.name = "count")# reshape the matrix
#df_dseq = melt(logCPM, variable.name = "Samples", value.name = "count")# reshape the ma
trix
df_dseq$Strain <- factor(substr(df_dseq$Var2, 1,3))
mycolors <- colorRampPalette(brewer.pal(8,"Set1"))(15)

ggplot(df_dseq, aes(x = count, color=Var2)) +
   geom_density(alpha = 0.5, size = 0.8) +
   #facet_wrap(~Strain, ncol=2) +
   theme_minimal() + xlim(-5, 25) +
   scale_colour_manual(values=mycolors, name="") +
   guides(fill="none")
```

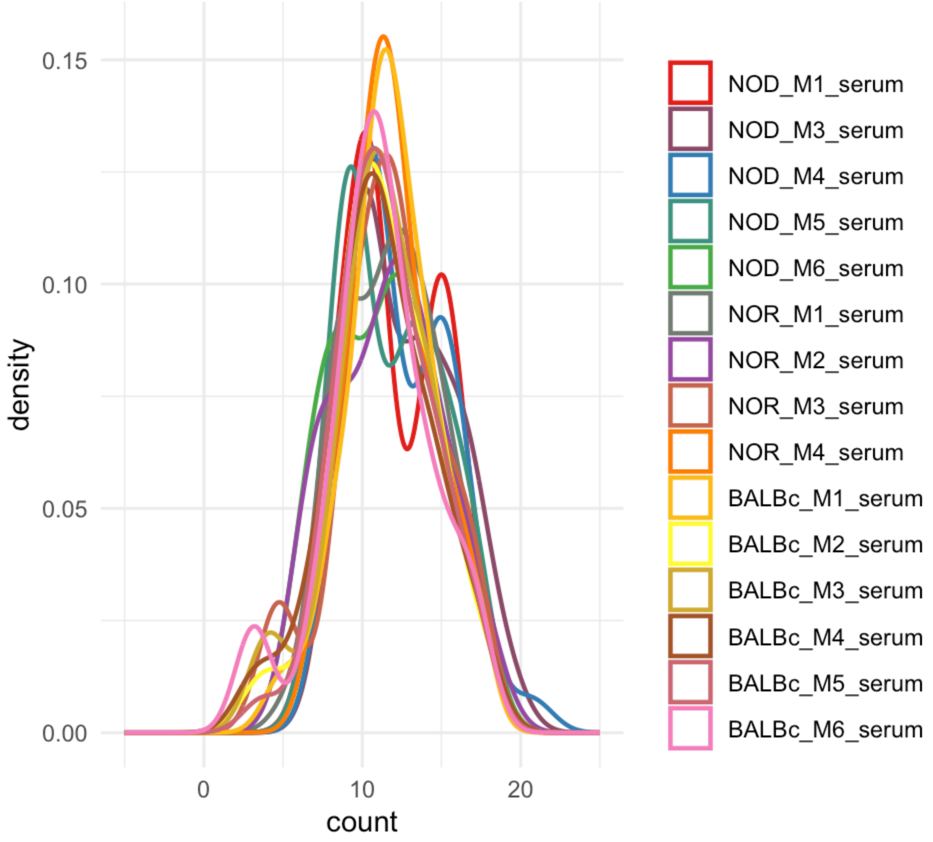

R-Squared goodness of fit for Serum IgA

```
for (i in 1:nrow(IgA_raw$NSI)){
   sst <- rowSums(v$E^2)
   ssr <- sst - fit.cont$df.residual*(fit.cont$sigma^2)
   Rsq<- (ssr/sst)
}
plot(1:nrow(IgA_raw$NSI), Rsq)
```

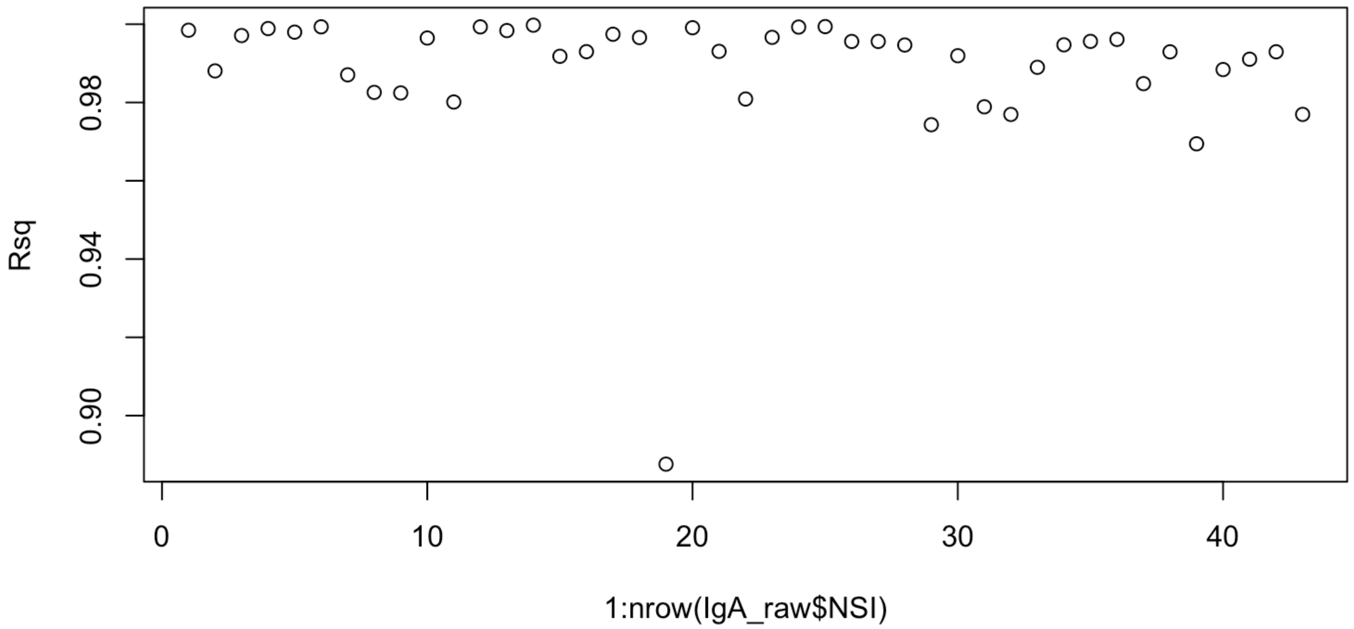


```
which(Rsq<0.8)
```

```
## named integer(0)
```

```
summary(fit.cont$r.squared)
```

```
## Length  Class   Mode
##      0   NULL   NULL
```

Limma Voom Differentially Expressed IgA auantibodies - Table 2

```
NTvsBT <- topTable(fit.cont, coef=1, number=30, adjust.method = 'BH')
nTvsBT <- topTable(fit.cont, coef=2, number=30, adjust.method = 'BH')
NTvsBT$Antigen <- row.names(NTvsBT)
nTvsBT$Antigen <- row.names(nTvsBT)
NTvnTvBT <- full_join(NTvsBT, nTvsBT, by="Antigen", suffix = c(".NOD", ".NOR"))
rownames(NTvnTvBT) <- NTvnTvBT$Antigen
NTvnTvBT[is.na(NTvnTvBT)] <- 0.5
NTvnTvBT <- NTvnTvBT[c(which(NTvnTvBT$adj.P.Val.NOD < 0.1 & NTvnTvBT$adj.P.Val.NOR < 0.
1)),]
write.csv(NTvnTvBT, file="NTvnTvBT_Serum_IgA.csv", sep=',')

knitr::kable(NTvnTvBT[c(1:2,5,8,12)])
```

	logFC.NOD	AveExpr.NOD	adj.P.Val.NOD	logFC.NOR	adj.P.Val.NOR
IF	11.095294	10.60414	0.0356583	11.821158	0.0326580
ssDNA	1.290165	14.21388	0.0940239	2.058677	0.0180012

###Determining DE genes

####DE Analysis using voom normalized counts

Boxplots from voom normalized counts

```
mydata <- as.matrix(v$E)
#mydata <- as.matrix(dataN)
hits <- rownames(NTvnTvBT)
Y=matrix(nrow=length(hits),ncol=15)
for (i in 1:length(hits)) {
   Y[i,] <- mydata[hits[i],]
}
rownames(Y) <- hits
colnames(Y) <- colData$Sample[]
Y <- as.data.frame(t(Y))
Y$Strain <- colData$Strain[]
Y$Sample <- paste0(Y$Strain, c(1:5,1:4, 1:6))

Y_combined <- Y[,c(NTvnTvBT$Antigen[which(NTvnTvBT$adj.P.Val.NOD<0.1 & NTvnTvBT$adj.P.Va
l.NOR < 0.1 & NTvnTvBT$logFC.NOD>1 & NTvnTvBT$logFC.NOR>1)])]

Y_combined[,(ncol(Y_combined)+1):(ncol(Y_combined)+2)] <- Y[,(ncol(Y)-1):ncol(Y)]
Y_combined <- gather(Y_combined, "Antigen", "V Counts", 1:(ncol(Y_combined)-2))
```

Combined Boxplot of Serum IgA (Supp Figure 7)

```
chart_design <- theme(
   #plot.title = element_text(color = "Black", size = 16, face = "bold", margin = margin
(b=15), hjust=0.4),
   axis.text.x = element_text(size=15),
   axis.text.y = element_text(size=14),
   axis.title.x = element_blank(),
   legend.text = element_text(size=15),
   legend.title = element_blank(),
   legend.position = "bottom",
   axis.title.y = element_text(size=18, margin = margin(r = 5)),
   strip.text.x = element_text(size =16, margin = margin(b=10), face='bold', hjust=0.4),
   strip.background = element_blank(),
   strip.placement = "outside")
```

```
#tiff("Serum_IgG_hits.tiff", units="in", width=5.5, height=4, res=300)
setwd("~/Documents/3_Parkinsons_disease/Autoantibody_Data/Tear_Auto_Validation_2022")
ggplot(Y_combined, aes(x=Strain, y=`V Counts`, fill=Strain)) +
      geom_boxplot(outlier.shape = NA, width = 0.5, coef=1, varwidth=F, show.legend = T,
size=0.65, position = position_dodge(0.9)) +
      geom_jitter(color = "darkgray", alpha =0.5, size=2.5, show.legend = F, position =
position_jitterdodge(dodge.width=0.9))+
   facet_wrap(~Antigen, ncol=2) +
      theme_minimal() +
      chart_design +
      ylab("Log2 Normalized Intensity")  +
      labs(title=colnames(Y[i]), hjust=0.5) +
      scale_fill_jco() + ylim(0,20)
```

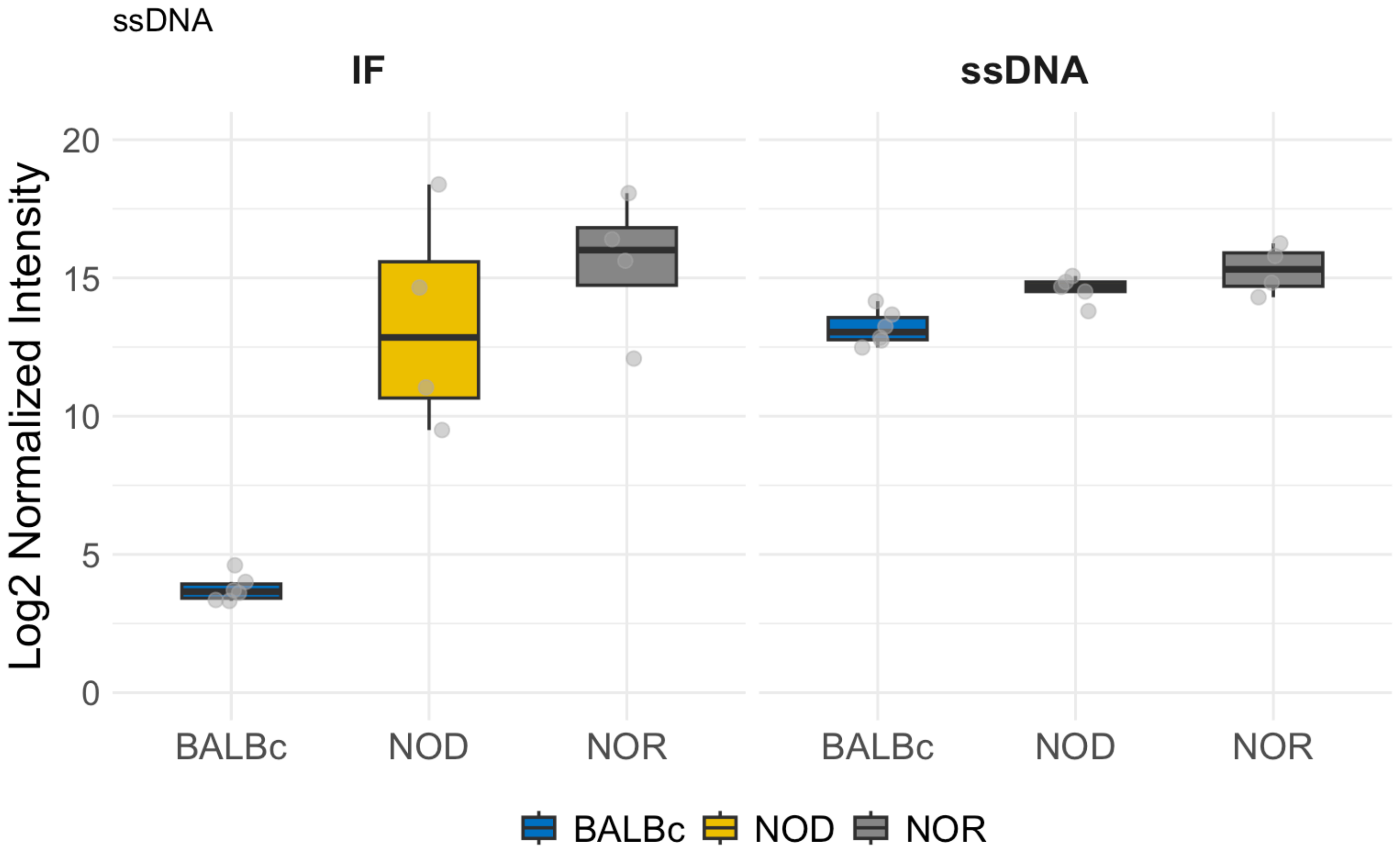


```
#dev.off()
```

Serum IgG Analysis

```
knitr::opts_chunk$set(
    echo = TRUE,
    message = FALSE,
    warning = FALSE,
    root.dir = '~/Documents/3_Parkinsons_disease/Autoantibody_Data/Tear_Auto_Validation_
2022/'
)
```

Reading in Raw IgG Datasets

```
setwd('~/Documents/3_Parkinsons_disease/Autoantibody_Data/Tear_Auto_Validation_2022/')
IgG_NSI <- read.csv("IgG_MCF_SSK_546_NSI_serum.csv",  header=T)[1:80,]
IgG_SNR <- read.csv("IgG_MCF_SSK_546_serum_SNR.csv",  header=T)[1:80,]
```

Autoantigen ID names

```
#Adding Auto-antigen id names
Antigen_ID <- read.csv("~/Documents/3_Parkinsons_disease/Autoantibody_Data/Tear_Auto_Val
idation_2022/Antigen_ID.csv",  header=T)[1:80,1:2]

#colnames(IgG_NSI)[1] <- colnames(Antigen_ID)[2]
#colnames(IgG_SNR)[1] <- colnames(Antigen_ID)[2]

Antigen_ID[68,"ID"] <- IgG_NSI[68,"ID"]
IgG_NSI <- full_join(IgG_NSI, Antigen_ID, by="ID")
IgG_SNR <- full_join(IgG_SNR, Antigen_ID, by="ID")
rownames(IgG_NSI) <- IgG_NSI$Antigen_ID
rownames(IgG_SNR) <- IgG_SNR$Antigen_ID

IgG_NSI <- IgG_NSI[,-c(1, 17)]
IgG_SNR <- IgG_SNR[,-c(1, 17)]
```

Setting up column metadata

```
Strain <- c(rep("NOD", each=5),rep("NOR", each=4), rep("BALBc", each=6))

colData <- as.data.frame(cbind(c(colnames(IgG_NSI)), Strain))
colnames(colData) <- c('Sample', "Strain")
rownames(colData) <- colData$Sample
colData$Strain <- factor(colData$Strain)
#colData$Strain <- relevel(colData$Strain, ref = "BALBc")
#Biofluid <- c( rep("Tear", each=11), rep("Serum", each=11))
#colData <- as.data.frame(cbind(c(colnames(IgG_NSI)), Strain, Biofluid))
```

Filtering Data based on low Signal to Noise ratio and Signal

Intensity

```
IgG_raw=list()
IgG_SNR$average <- rowMeans(IgG_SNR)
IgG_SNR$med <- rowMedians(as.matrix(IgG_SNR))

IgG_NSI$average <- rowMeans(IgG_NSI)
IgG_NSI$med <- rowMedians(as.matrix(IgG_NSI))

IgG_raw$NSI <- as.matrix(IgG_NSI[which(IgG_NSI$med>50 & IgG_SNR$med>2),])[,1:15]
IgG_raw$SNR <- as.matrix(IgG_SNR[which(IgG_NSI$med>50 & IgG_SNR$med>2),])[,1:15]
IgG_raw$Ab_Score <- as.matrix(log2((IgG_raw$NSI*IgG_raw$NSI) + 1))
```

Visualizing Filtered Data

```
dataN <- log2(IgG_raw$NSI + 0.5) #* IgG_raw$SNR + 1)
countData = as.data.frame(dataN)

df_dseq = melt(countData, variable.name = "Samples", value.name = "count")# reshape the
matrix

mycolors <- colorRampPalette(brewer.pal(8, "Set1"))(15)

ggplot(df_dseq, aes(x = count, color=Samples)) +
   geom_density(alpha = 0.5, size = 0.8) +
   #facet_wrap(~Strain, ncol=2) +
   theme_minimal() + #xlim(-1.5,6) +
   scale_colour_manual(values=mycolors, name="") +
   guides(fill="none")
```

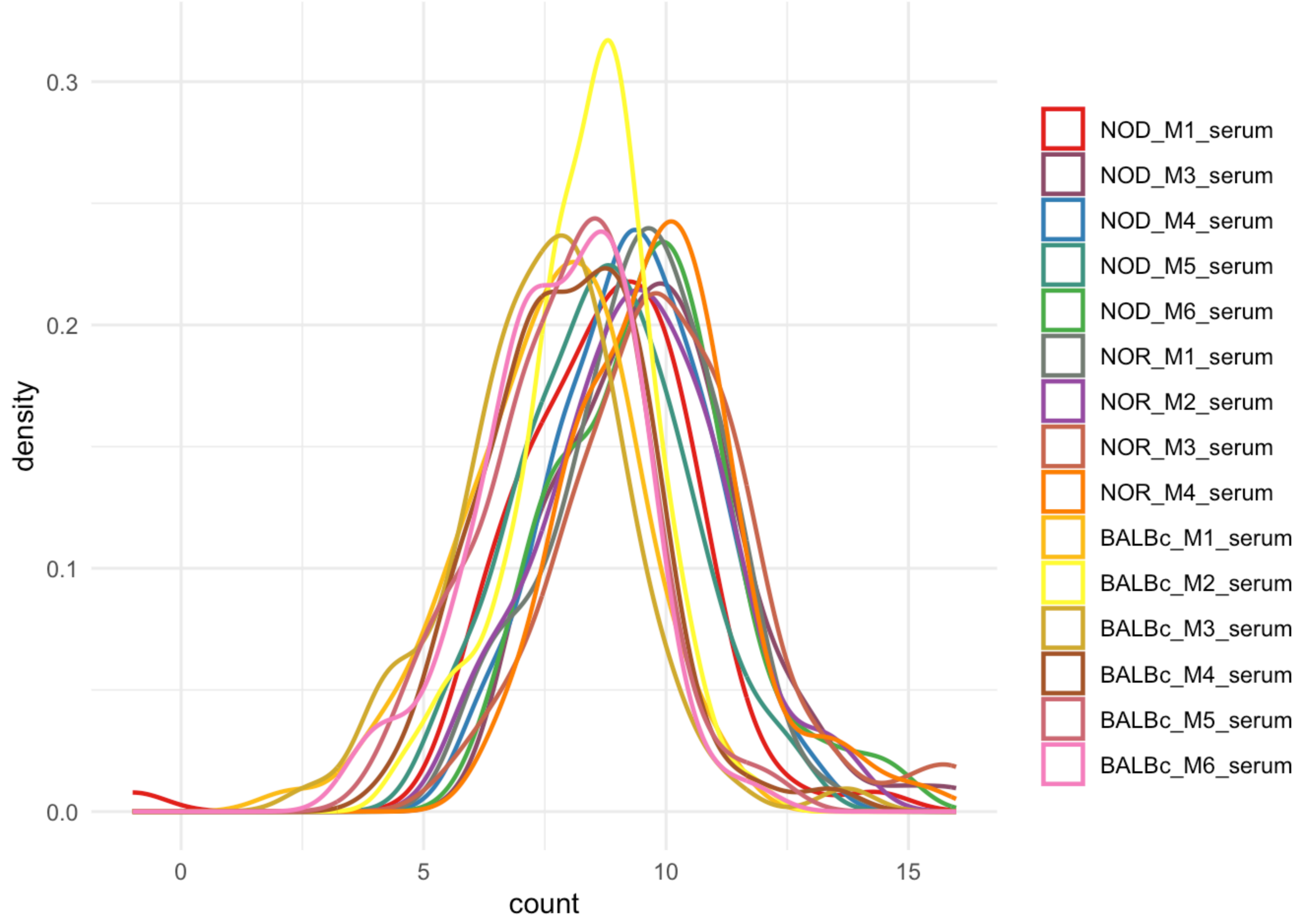

There do not appear to be any extreme outliers.

Boxplots of raw and SNR based Filtered Data

```
boxplot(as.data.frame((dataN)),main="IgG normalization")#,col=Sample)
```

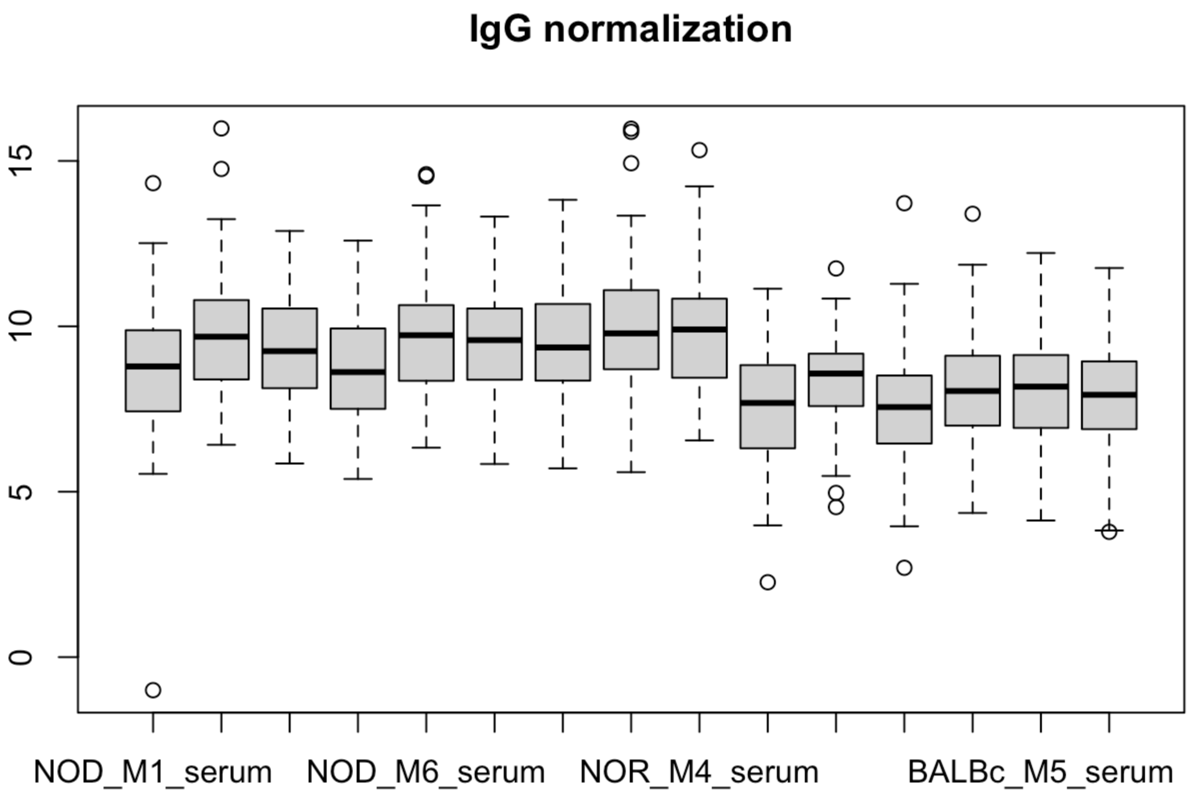


```
dataN <- (IgG_raw$NSI + 1)
countData = as.data.frame(dataN)
boxplot(as.data.frame((dataN)),main="NSI")
```

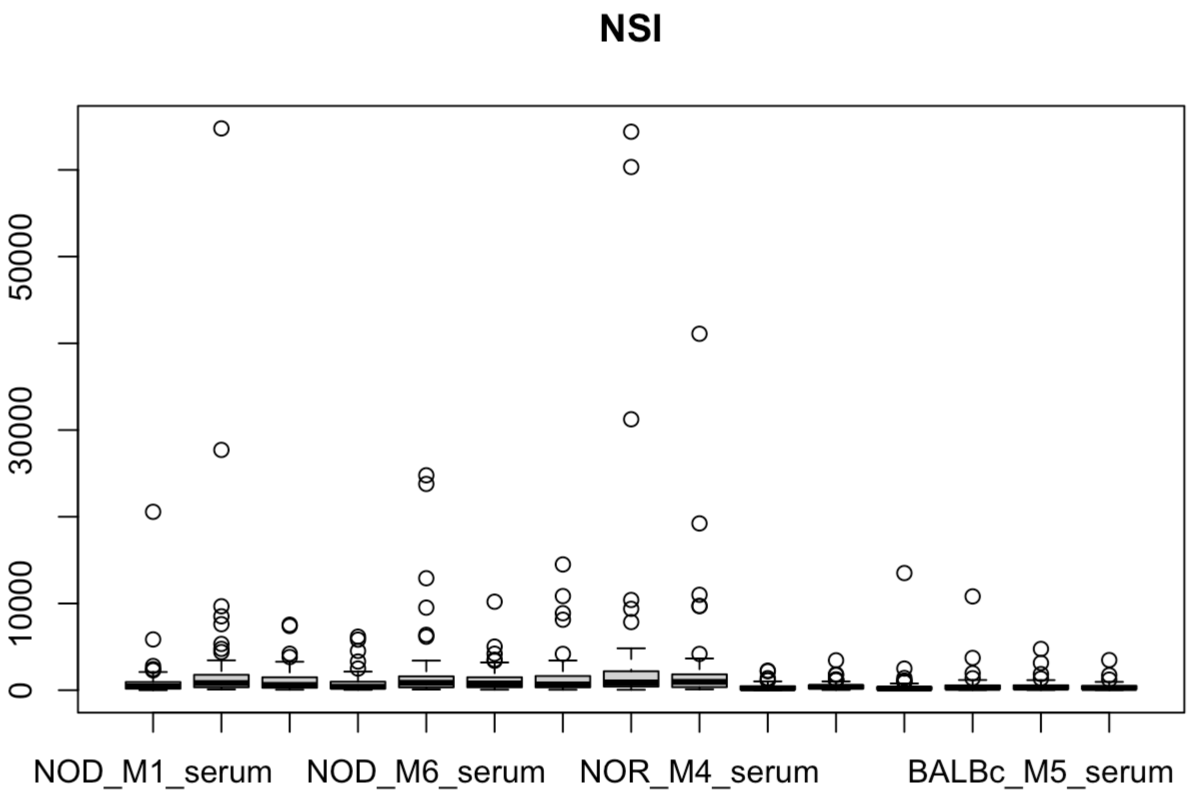


```
dataN <- log2((IgG_raw$NSI*100000)/colSums(IgG_raw$NSI)+0.5)
countData = as.data.frame(dataN)
boxplot(as.data.frame((dataN)),main="Column Normalized NSI")
```

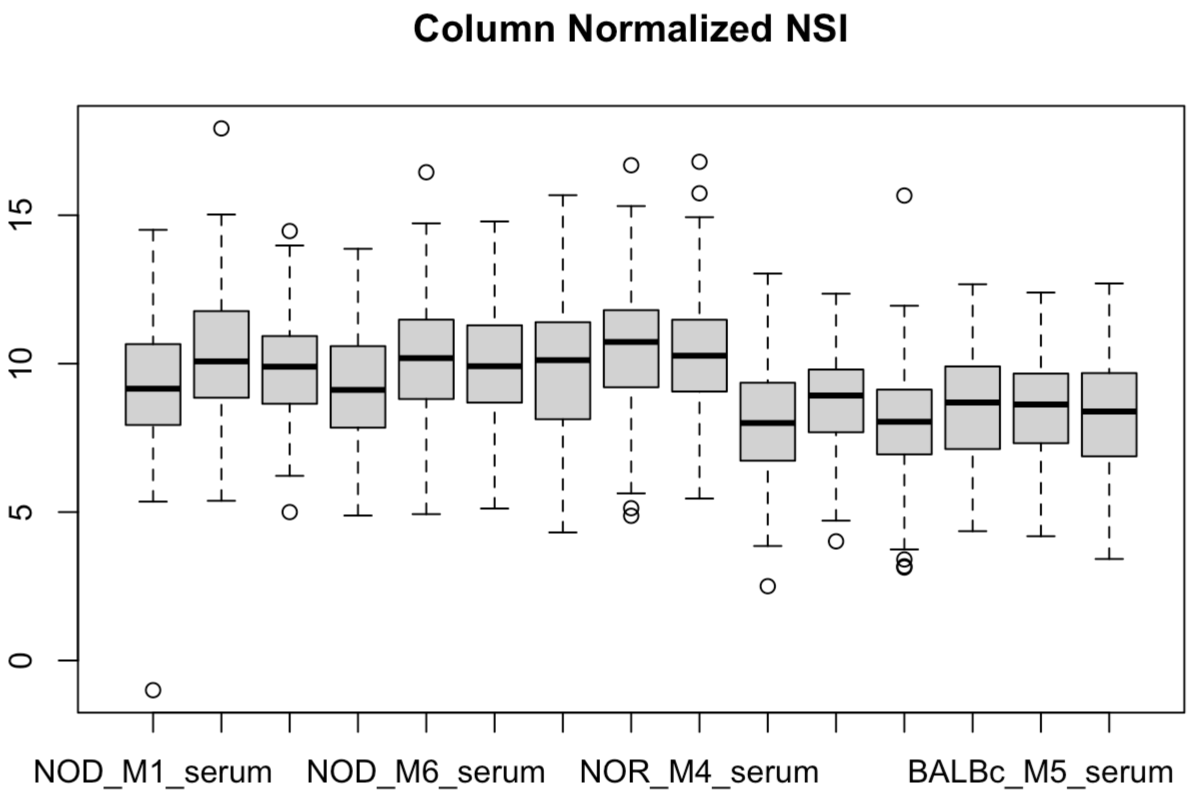

We see global differences between groups, with BALB/c medians being lower than both NOD and NOR.

Limma Least Squares Normalization

```
conditions<- paste(colData$Strain,sep=".")
conditions <- factor(conditions, levels=unique(conditions))
design <- model.matrix(~0+ conditions)
colnames(design) <- levels(conditions)

library(limma)
#dataN <- log((IgG_raw$NSI*IgG_raw$SNR*1000000)/colSums(IgG_raw$NSI*IgG_raw$SNR)+1)
dataN <- log2((IgG_raw$NSI*10000)/colSums(IgG_raw$NSI)+0.5)
v <- voom(2^(dataN))
#dataN <- log(IgG_raw$NSI*RowTriMeans(IgG_raw$NSI) +1)
#dataN <- log(IgG_raw$NSI + 1)
plotMD(dataN) #
```

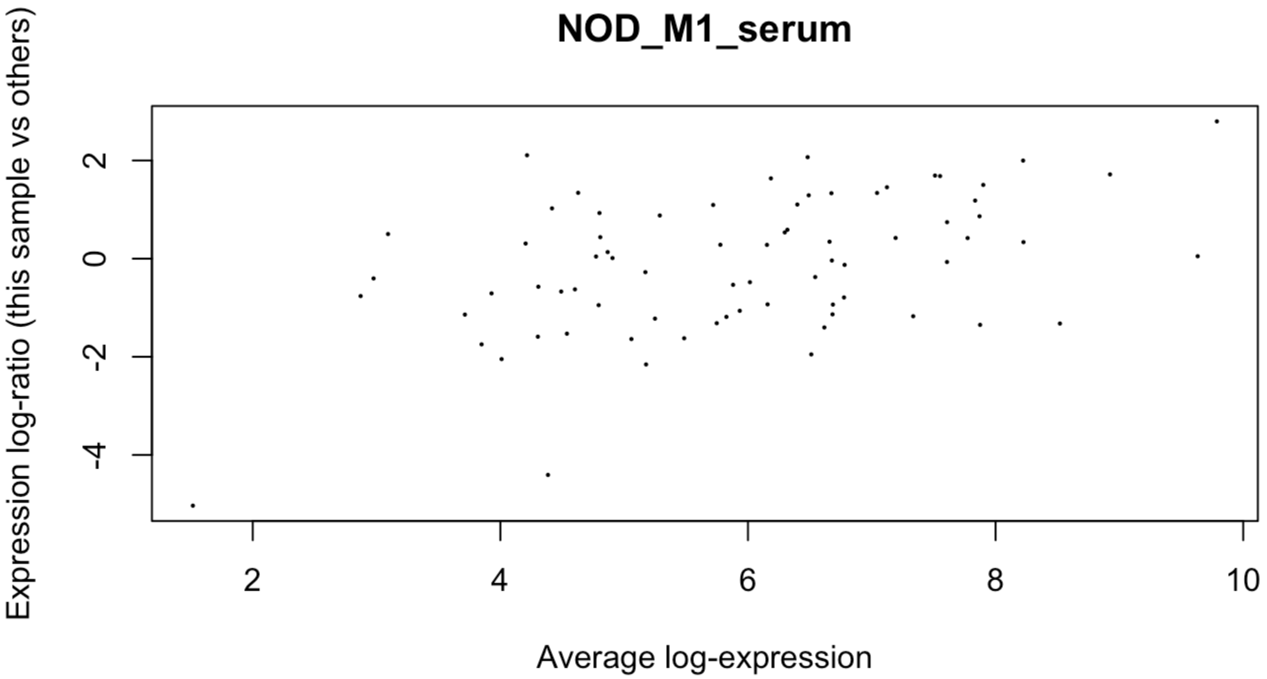


```
#block <- as.factor(c(rep("N",9), rep("B",6)))
#duplicateCorrelation(dataN, design)
fit <- lmFit(dataN, design, plot=TRUE, method="ls")
plotMD(fit, column=1)
```

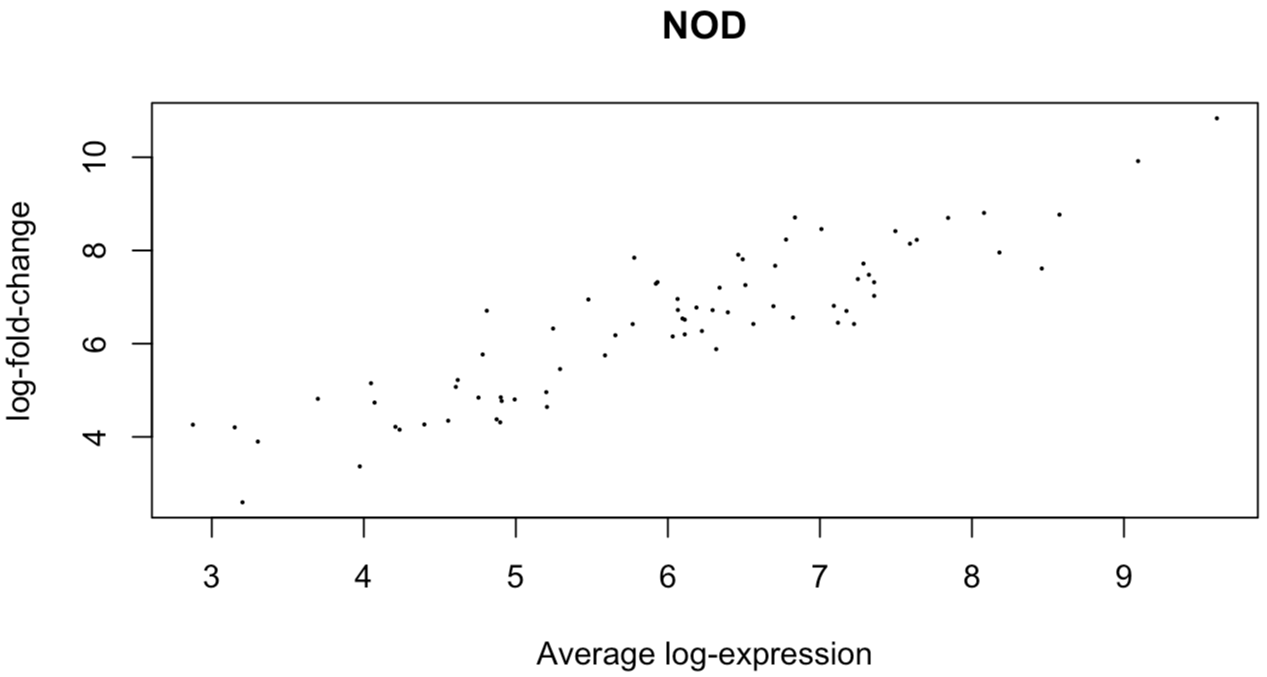

```
cont.matrix<- makeContrasts(
   NTvBT =  NOD - BALBc,
   nTvBT = NOR - BALBc,
   levels = design)
fit.cont<- contrasts.fit(fit, cont.matrix)
fit.cont<- eBayes(fit.cont, robust=TRUE)
results <- decideTests(fit.cont)

vennCounts(results)
```

```
##   NTvBT nTvBT Counts
## 1     0     0     33
## 2     0     1     14
## 3     1     0     10
## 4     1     1     17
## attr(,"class")
## [1] "VennCounts"
```

```
topTable(fit.cont, number=30)
```

```
##                NTvBT     nTvBT  AveExpr         F      P.Value    adj.P.Val
## BPI       2.7153155 3.9847588 6.509511 24.128935 5.220733e-06 0.0003493743
## GAD65     2.6029155 3.7847185 8.079380 22.136126 9.442548e-06 0.0003493743
## PDC-E2    2.7971342 3.3710767 6.705534 20.487482 1.586537e-05 0.0003913457
## LC1       2.3084461 3.5560433 7.636970 17.700586 4.082079e-05 0.0007551847
## PL-7      2.2868183 3.6461616 7.592331 16.664180 5.946035e-05 0.0008230138
## LKM 1     2.3148071 3.8138363 5.654447 16.354209 6.673085e-05 0.0008230138
## KU P70P80 2.8647361 1.7002950 6.777125 14.014662 1.670339e-04 0.0017657870
## OGDC-E2   2.9133107 1.7655011 5.476714 13.176786 2.372004e-04 0.0021941040
## La SSB    3.0427731 2.1730012 7.009738 11.905528 4.141544e-04 0.0034052692
## PCNA      2.5211906 3.0759066 6.340015 11.618817 4.717489e-04 0.0034909416
## FBG S     2.4909692 3.0187642 7.843072 11.355595 5.324728e-04 0.0035820899
## SP100     3.0178026 3.8452350 4.781685 10.967245 6.383964e-04 0.0038109719
## P0        2.8244344 1.8640119 2.875482 10.866618 6.694951e-04 0.0038109719
## Sm RNP    4.3398729 3.7322627 4.808732 10.361254 1.136946e-03 0.0053390195
## B2GP1     1.2206407 2.7191526 6.110770  9.744127 1.156778e-03 0.0053390195
## KS        2.4801426 0.7813100 6.462629  9.656202 1.209000e-03 0.0053390195
## FBG IV    2.6725322 2.0904414 9.611157  9.627621 1.226532e-03 0.0053390195
## PM Scl75  1.3677673 3.0869959 4.754052  8.937521 1.747843e-03 0.0071855778
## GBM       1.2288584 2.6097028 6.031718  8.365258 2.368228e-03 0.0090802178
## BCOADC-E2 1.9604911 2.6975702 6.187737  8.275799 2.485543e-03 0.0090802178
## CENP-A    1.2692537 3.4740010 4.235195  8.209357 2.576819e-03 0.0090802178
## NXP2      2.3678440 0.7962399 5.920747  8.118080 2.708292e-03 0.0091097085
## PL-12     0.8984517 2.7702054 6.562015  7.735251 3.346219e-03 0.0107660974
## SRP54     1.9822756 2.5121316 5.768365  6.997538 5.097648e-03 0.0157177486
## VTN       2.0980779 0.3011786 6.492271  6.788805 5.761781e-03 0.0165892218
## Laminin   1.6899971 2.4719391 4.605027  6.769274 5.828646e-03 0.0165892218
## M2        1.6049688 2.9667978 6.394281  6.692161 6.101126e-03 0.0167216038
## Elastin   2.0899548 1.0842695 4.046980  6.410293 7.223315e-03 0.0190901891
## ACAN      2.1996301 1.5450683 3.150579  6.181292 8.303398e-03 0.0207243931
## Nup62     2.5466393 1.1589470 5.931458  6.162085 8.401781e-03 0.0207243931
```

QC Plots: Serum IgG Limma Robust

```
#plotMD(fit)
plotMDS(v$E,col=as.numeric(Strain[]))
```

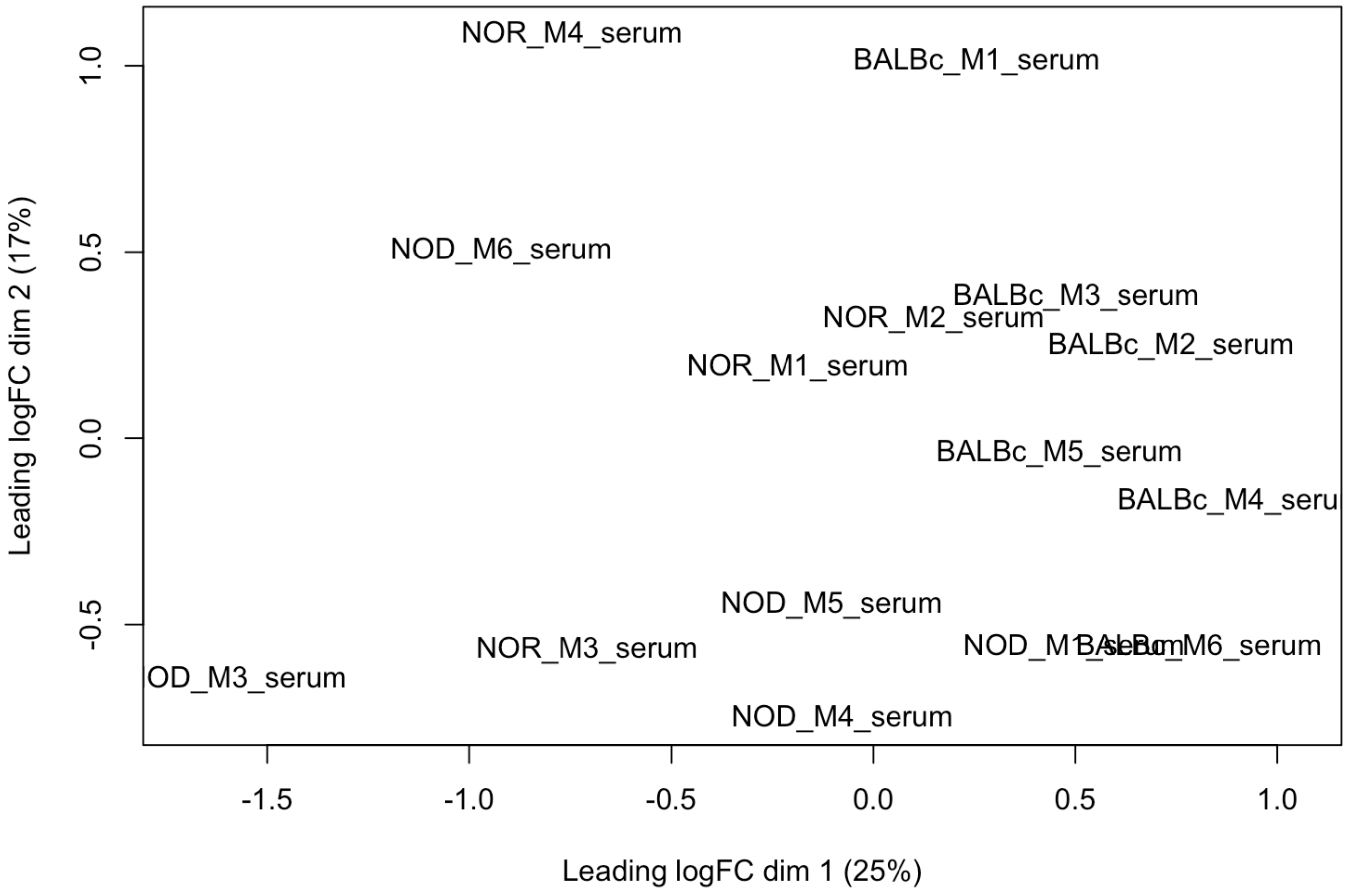


```
plotMD(fit.cont)
```

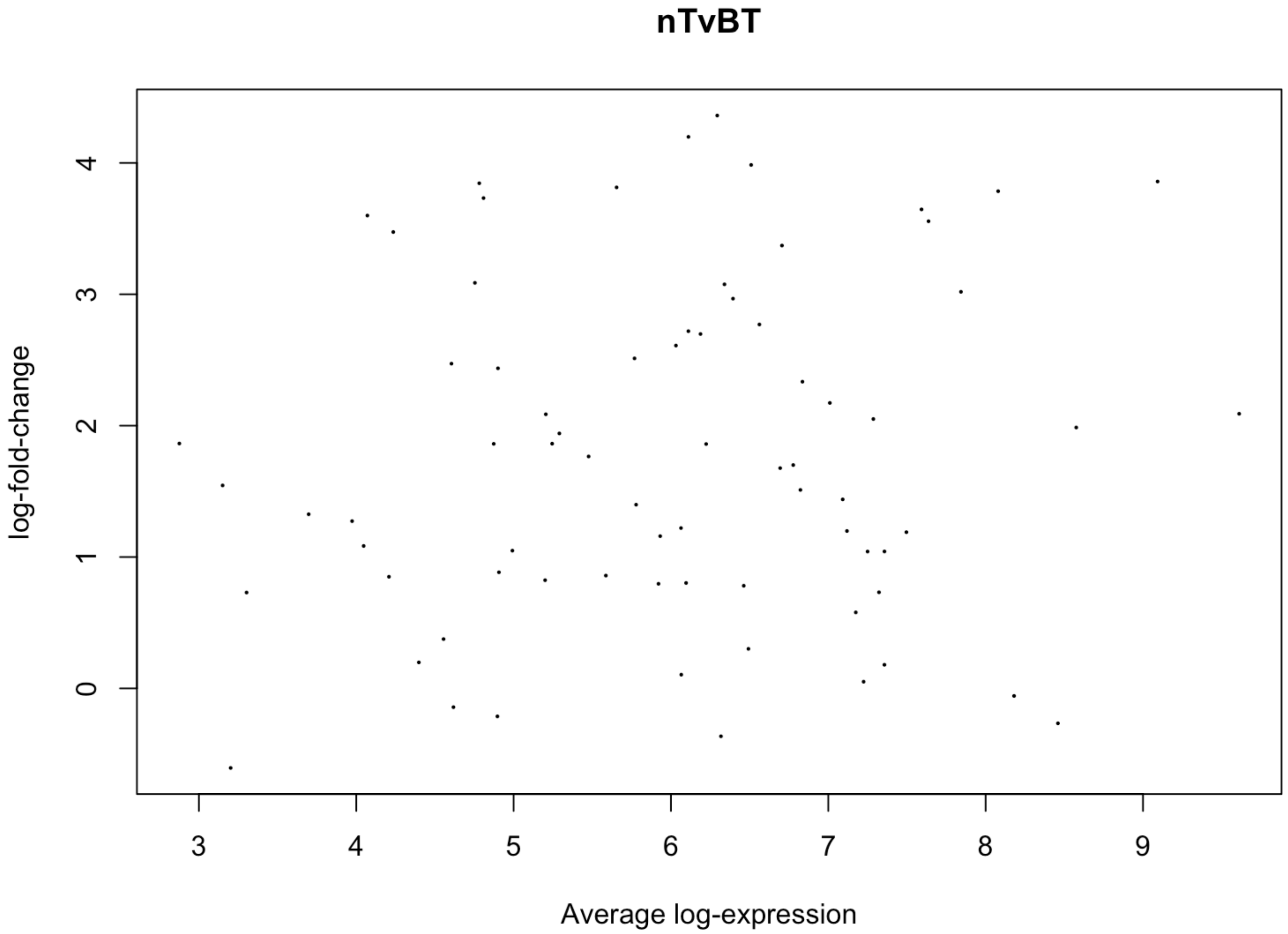


```
qqnorm(fit.cont$t,df=(fit.cont$df.prior+fit.cont$df.residual))
```

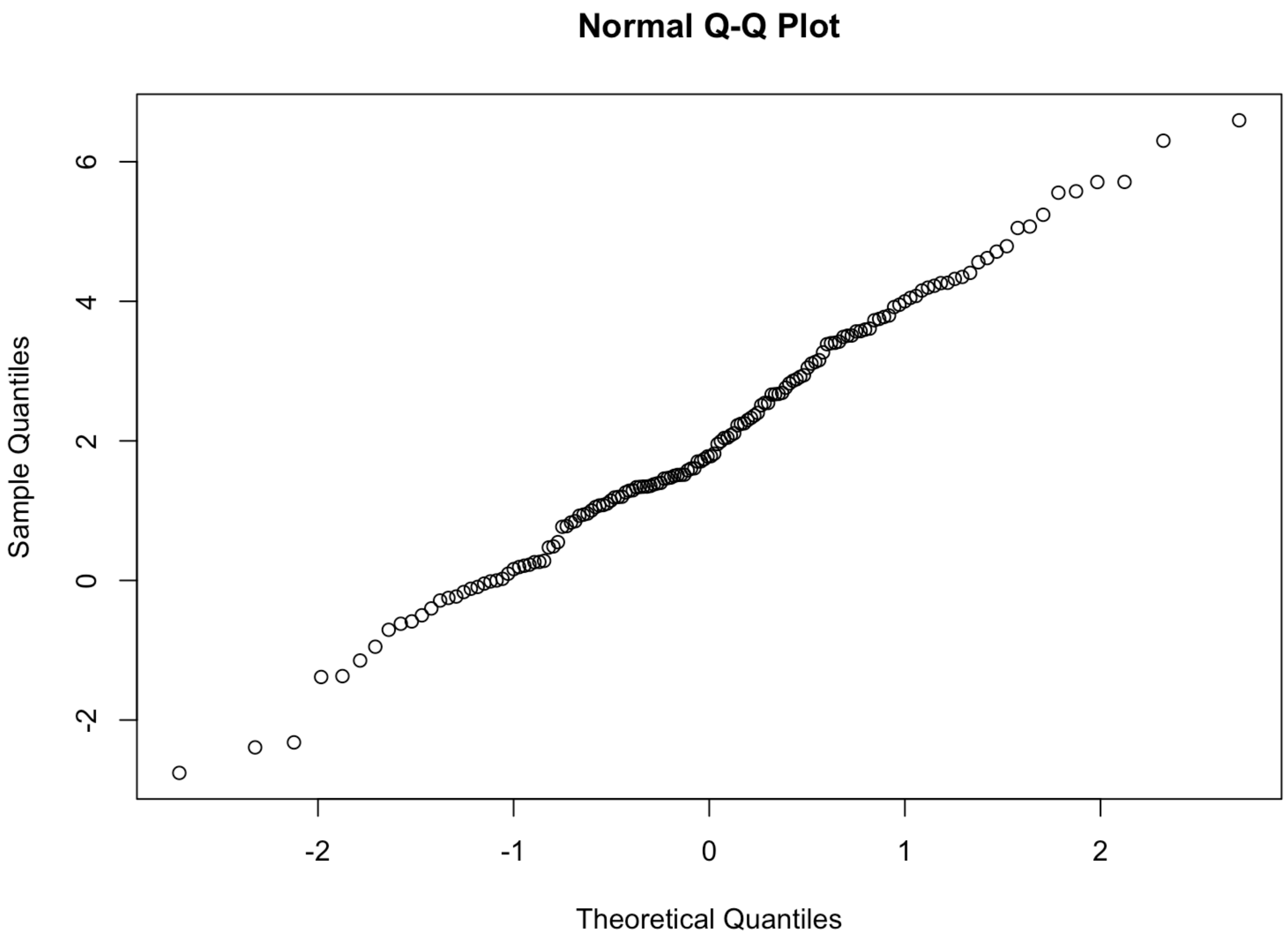


```
#ggqqplot(fit.cont)
#Top ten upregulated autoantigens
volcanoplot(fit.cont, highlight = 10, names = rownames(as.data.frame(fit.cont$Amean)))
```

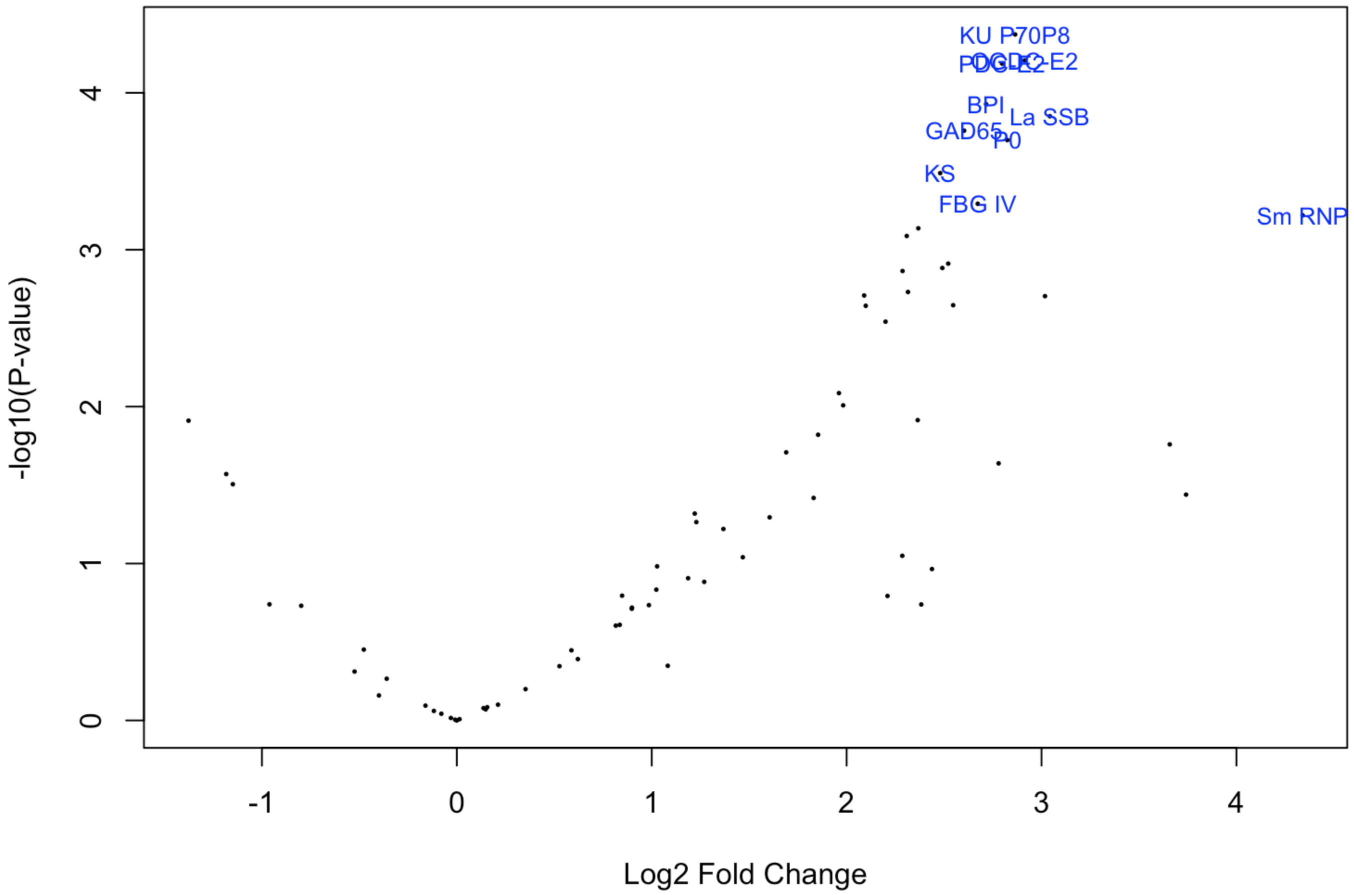

NODM1 appears to be an outlier

Density plots post normalization

```
dataN = log2((IgG_NSI[,1:15])+0.5)
df_dseq = melt(dataN, variable.name = "Samples", value.name = "count")# reshape the matr
ix
df_dseq$Strain <- factor(substr(df_dseq$Samples, 1,3))
mycolors <- colorRampPalette(brewer.pal(8,"Set1"))(15)
ggplot(df_dseq, aes(x = count, color=Samples)) +
   geom_density(alpha = 0.5, size = 0.8) +
   #facet_wrap(~Strain, ncol=2) +
   theme_minimal() + xlim(-5, 25) +
   scale_colour_manual(values=mycolors, name="") +
   guides(fill="none")
```

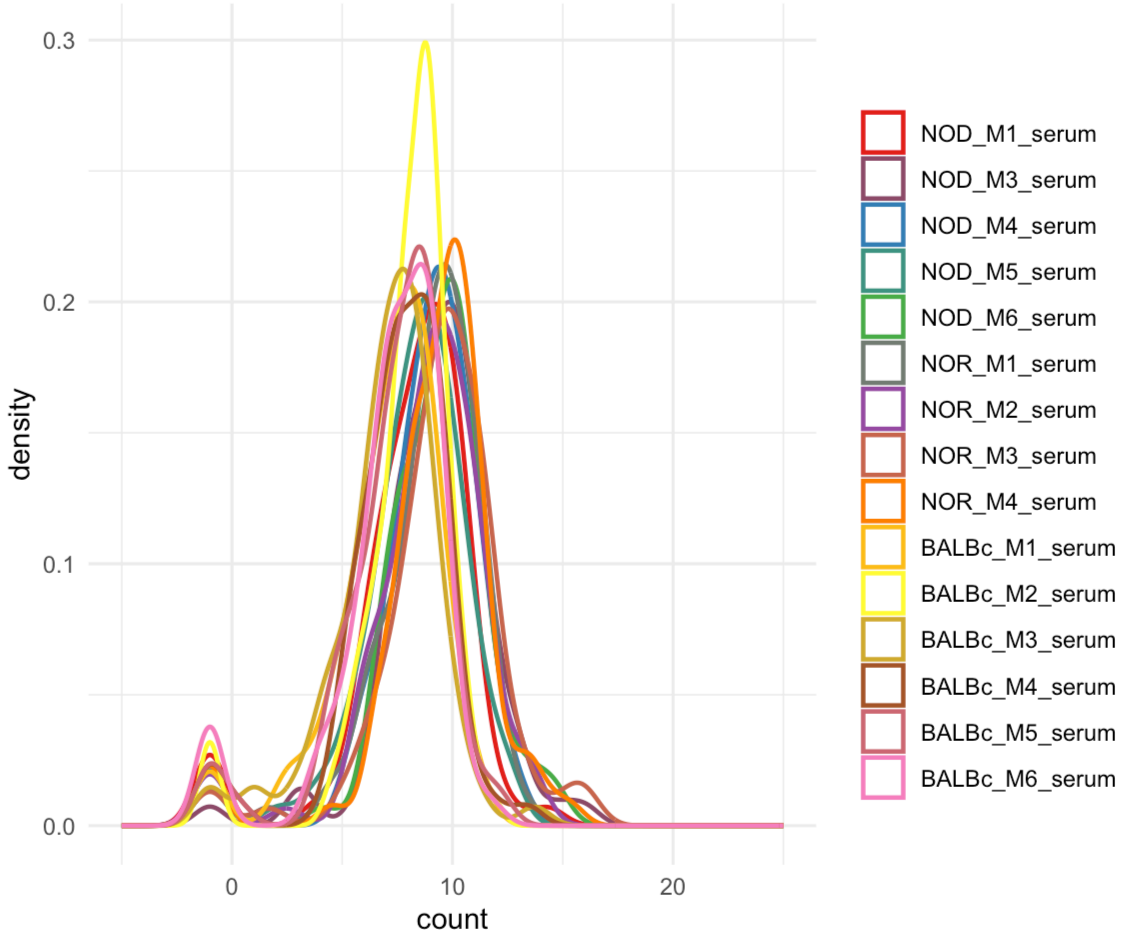


```
rm(dataN)
rm(df_dseq)
dataN = log2(as.data.frame(IgG_raw$NSI*10000)/colSums(IgG_raw$NSI)+0.5)
df_dseq = melt(dataN, variable.name = "Samples", value.name = "count")# reshape the matr
ix
df_dseq$Strain <- factor(substr(df_dseq$Samples, 1,3))
mycolors <- colorRampPalette(brewer.pal(8,"Set1"))(15)
ggplot(df_dseq, aes(x = count, color=Samples)) +
   geom_density(alpha = 0.5, size = 0.8) +
   #facet_wrap(~Strain, ncol=2) +
   theme_minimal() + xlim(-5, 25) +
   scale_colour_manual(values=mycolors, name="") +
   guides(fill="none")
```

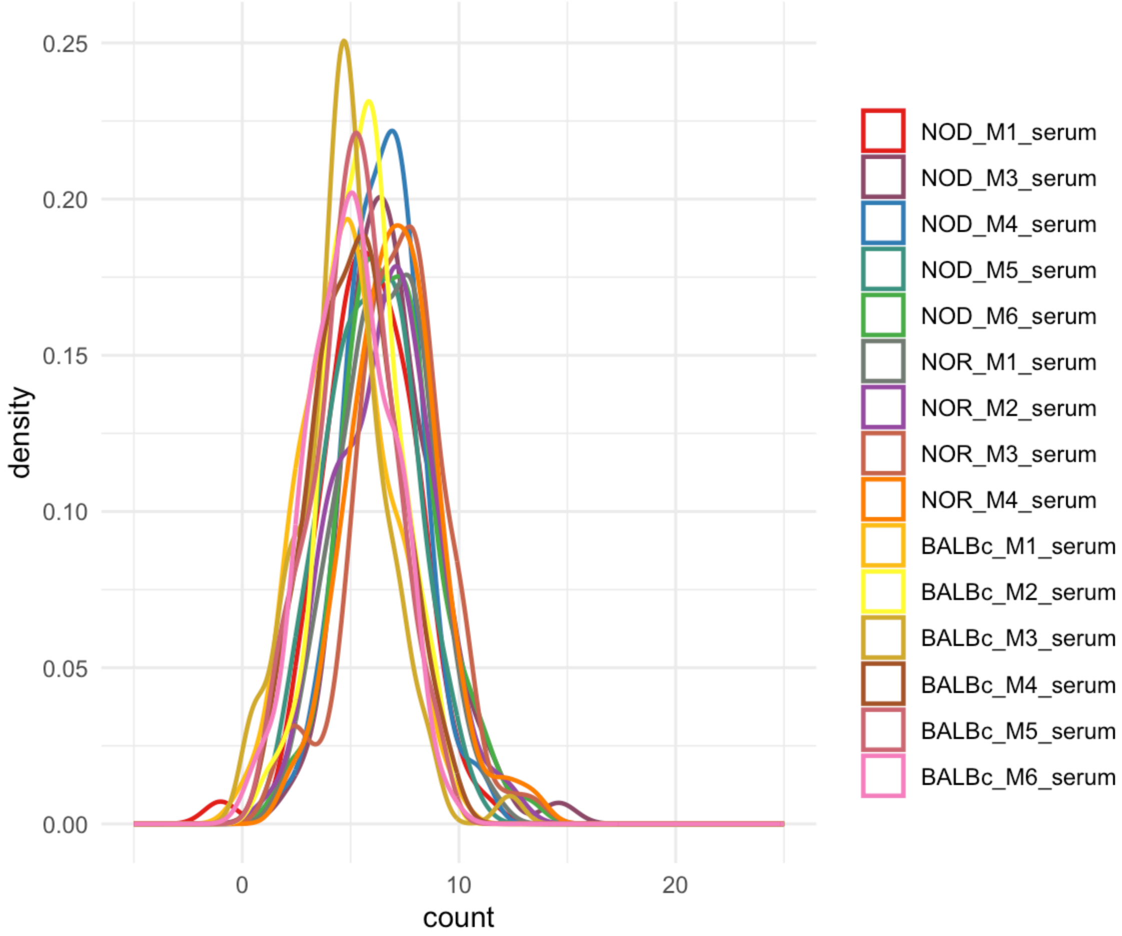


```
rm(dataN)
rm(df_dseq)
dataN <- v$E
df_dseq = melt(dataN, variable.name = "Samples", value.name = "count")# reshape the matr
ix
df_dseq$Strain <- factor(substr(df_dseq$Var2, 1,3))
ggplot(df_dseq, aes(x = count, color=Var2)) +
   geom_density(alpha = 0.5, size = 0.8) +
   #facet_wrap(~Strain, ncol=2) +
   theme_minimal() + xlim(-5, 25) +
   scale_colour_manual(values=mycolors, name="") +
   guides(fill="none")
```

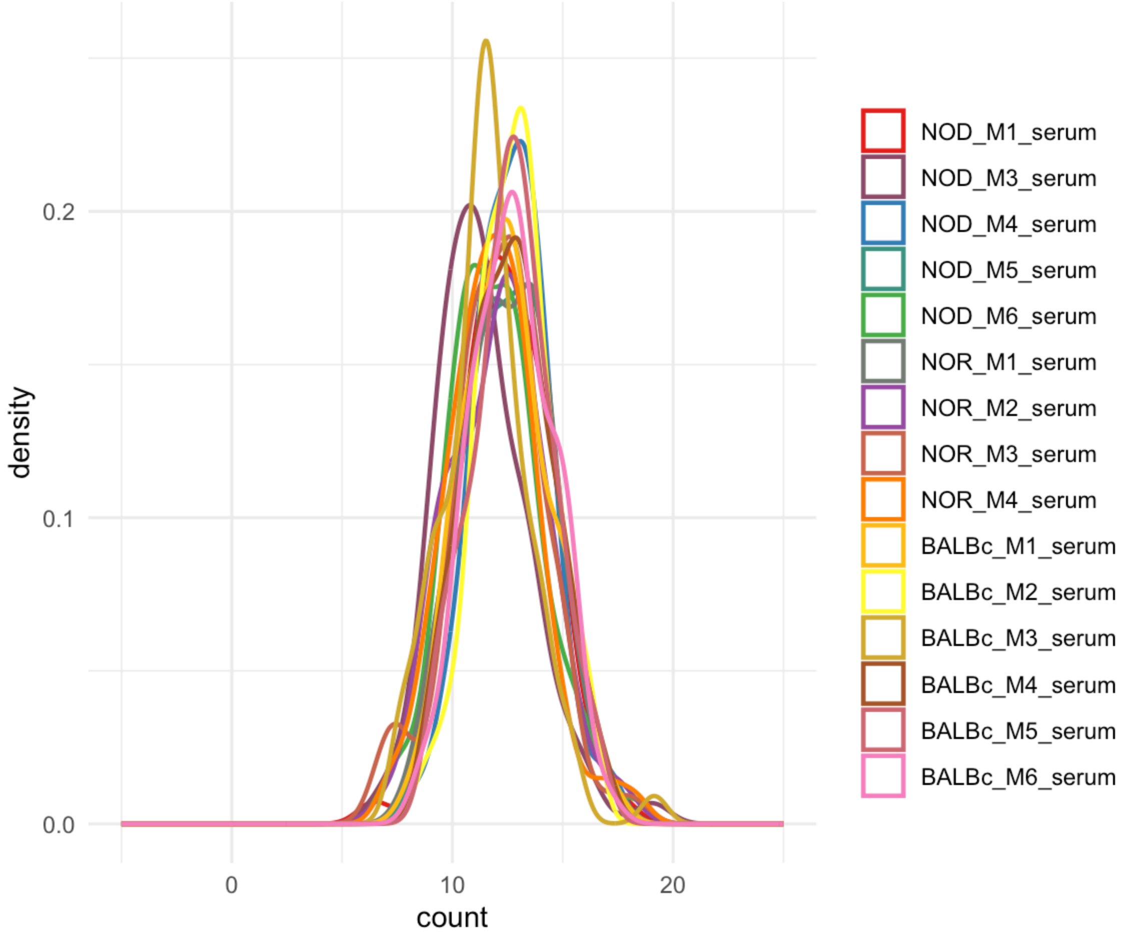

Quantile Normalization is over-normalizing this dataset. Therefore we will do a simple log Normalization followed by limma based DE analysis.

Library Normalization appears to be the best recourse

Upregulated Serum IgG Autoantibodies - Table 2

```
NTvsBT <- topTable(fit.cont, coef=1, number=30, adjust.method = 'BH')
nTvsBT <- topTable(fit.cont, coef=2, number=30, adjust.method = 'BH')
NTvsBT$Antigen <- row.names(NTvsBT)
nTvsBT$Antigen <- row.names(nTvsBT)
NTvnTvBT <- full_join(NTvsBT, nTvsBT, by="Antigen", suffix = c(".NOD", ".NOR"))
rownames(NTvnTvBT) <- NTvnTvBT$Antigen
NTvnTvBT[is.na(NTvnTvBT)] <- 0.5

NTvnTvBT <- NTvnTvBT[c(which(NTvnTvBT$adj.P.Val.NOD < 0.03 & NTvnTvBT$adj.P.Val.NOR < 0.
03 & NTvnTvBT$logFC.NOD > 1.9 & NTvnTvBT$logFC.NOR > 1.9)),]
setwd("~/Documents/3_Parkinsons_disease/Autoantibody_Data/Tear_Auto_Validation_2022/")
write.csv(NTvnTvBT, file="NTvnTvBT_Serum_IgG_2022.csv", sep=',')

knitr::kable(NTvnTvBT[c(1:2,5,8,12)])
```

	logFC.NOD	AveExpr.NOD	adj.P.Val.NOD	logFC.NOR	adj.P.Val.NOR
PDC-E2	2.797134	6.705534	0.0016054	3.371077	0.0002586

	logFC.NOD	AveExpr.NOD	adj.P.Val.NOD	logFC.NOR	adj.P.Val.NOR
BPI	2.715315	6.509511	0.0020873	3.984759	0.0001537
La SSB	3.042773	7.009738	0.0020873	2.173001	0.0170166
GAD65	2.602915	8.079380	0.0021152	3.784718	0.0001537
FBG IV	2.672532	9.611157	0.0041900	2.090441	0.0198938
Sm RNP	4.339873	4.808732	0.0044686	3.732263	0.0129824
LC1	2.308446	7.636970	0.0050418	3.556043	0.0002586
PCNA	2.521191	6.340015	0.0067419	3.075907	0.0029311
FBG S	2.490969	7.843071	0.0067419	3.018764	0.0029311
PL-7	2.286818	7.592331	0.0067419	3.646162	0.0002586
LKM 1	2.314807	5.654446	0.0081250	3.813836	0.0002586
SP100	3.017803	4.781685	0.0081250	3.845235	0.0029311
BCOADC-E2	1.960491	6.187737	0.0275818	2.697570	0.0057598

#####Voom Normalization After Removing outliers ####

####R-Squared ……. goodness of fit ####

#####Determining DE genes##

#####DE Analysis using voom normalized counts

Boxplots from library normalized counts

```
mydata <- as.matrix(log2((IgG_raw$NSI*10000)/colSums(IgG_raw$NSI) + 1))
hits <- rownames(NTvnTvBT)
Y=matrix(nrow=length(hits),ncol=15)
for (i in 1:length(hits)) {
   Y[i,] <- mydata[hits[i],]
}
rownames(Y) <- hits
colnames(Y) <- colData$Sample
Y <- as.data.frame(t(Y))
Y$Strain <- colData$Strain
Y$Sample <- paste0(Y$Strain, c(1:5,1:4, 1:6))
Y_combined <- Y[,c(NTvnTvBT$Antigen)]

Y_combined[,(ncol(Y_combined)+1):(ncol(Y_combined)+2)] <- Y[,(ncol(Y)-1):ncol(Y)]
Y_combined <- gather(Y_combined, "Antigen", "V Counts", 1:(ncol(Y_combined)-2))
```

```
chart_design <- theme(
   #plot.title = element_text(color = "Black", size = 16, face = "bold", margin = margin
(b=15), hjust=0.4),
   axis.text.x = element_text(size=13),
   axis.text.y = element_text(size=14),
   axis.title.x = element_blank(),
   legend.text = element_text(size=15),
   legend.title = element_blank(),
   legend.position = "bottom",
   axis.title.y = element_text(size=17, margin = margin(r = 5)),
   strip.text.x = element_text(size =16, margin = margin(b=10), face='bold', hjust=0.4),
   strip.background = element_blank(),
   strip.placement = "outside")
```

Study 1 Validation plot - Figure 2B

```
Y_combined_I <- Y_combined[which(Y_combined$Antigen %in% c('La SSB', 'LC1', 'PCNA')),]

#tiff("Serum_IgG_Figure2B.tiff", units="in", width=6.5, height=3, res=300)
setwd("~/Documents/3_Parkinsons_disease/Autoantibody_Data/Tear_Auto_Validation_2022/")
ggplot(Y_combined_I, aes(x=Strain, y=`V Counts`, fill=Strain)) +
      geom_boxplot(outlier.shape = NA, width = 0.5, coef=1, varwidth=F, show.legend = T,
size=0.65, position = position_dodge(0.9)) +
      geom_jitter(color = "darkgray", alpha =0.5, size=2.5, show.legend = F, position =
position_jitterdodge(dodge.width=0.9))+
   facet_wrap(~Antigen, ncol=5, scales="free_x") +
      theme_minimal() +
      chart_design +
      ylab("Log2 Normalized Intensity")  +
      labs(title=colnames(Y[i]), hjust=0.5) +
      scale_fill_jco()
```

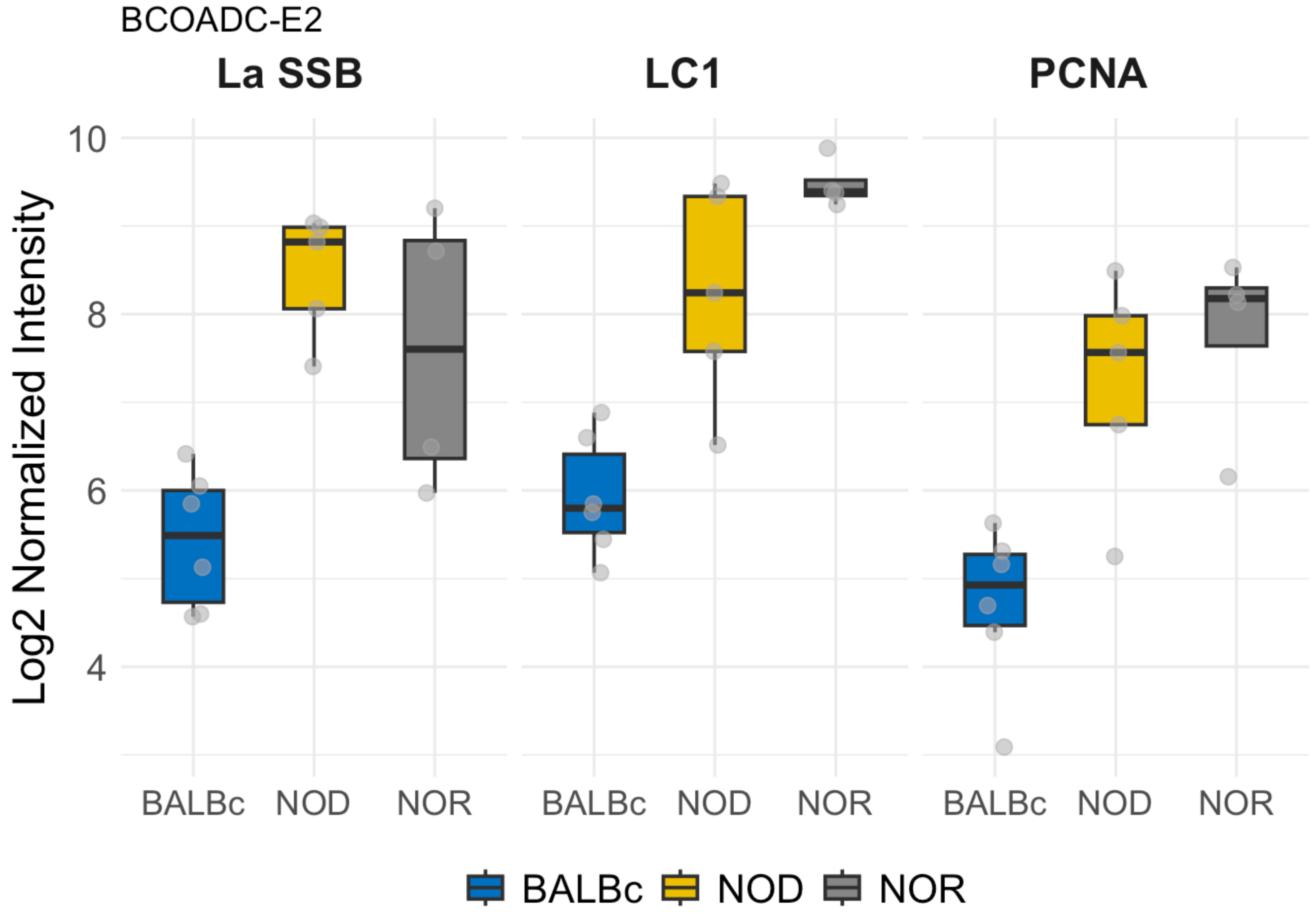


```
#dev.off()
```

Common to tears and serum IgG - Supplemental Figure 5B

```
Y_combined_II <- Y_combined[which(Y_combined$Antigen %in% c('PL-7', 'GAD65', 'LKM 1')),]

#tiff("Serum_Tear_IgG_common_hits.tiff", units="in", width=7, height=3, res=300)
setwd("~/Documents/3_Parkinsons_disease/Autoantibody_Data/Tear_Auto_Validation_2022/")
ggplot(Y_combined_II, aes(x=Strain, y=`V Counts`, fill=Strain)) +
      geom_boxplot(outlier.shape = NA, width = 0.5, coef=1, varwidth=F, show.legend = T,
size=0.65, position = position_dodge(0.9)) +
      geom_jitter(color = "darkgray", alpha =0.5, size=2.5, show.legend = F, position =
position_jitterdodge(dodge.width=0.9))+
   facet_wrap(~Antigen, ncol=5, scales="free_y") +
      theme_minimal() + ylim(2.5,12.15) +
      chart_design +
      ylab("Log2 Normalized Intensity")  +
      labs(title=colnames(Y[i]), hjust=0.5) +
      scale_fill_jco()
```

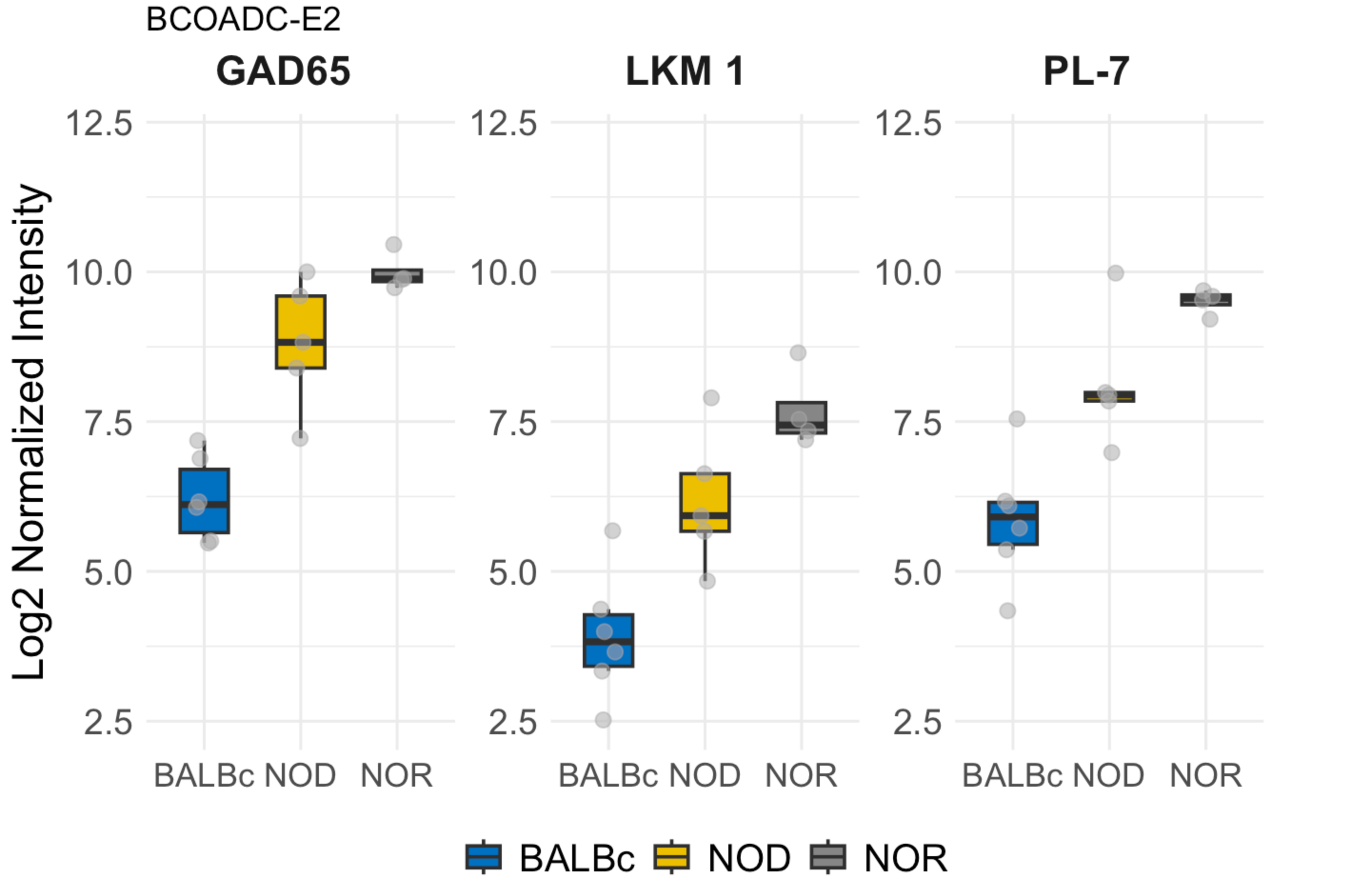


```
#dev.off()
```

Supplemental Figure 3

Unique to serum IgG in Study 3

```
Y_combined_2 <- Y_combined[-which(Y_combined$Antigen %in% c("PCNA", "La SSB", "LC1", "PL
-7", "GAD65", "LKM 1", "FBG IV")),]

#tiff("Serum_only_IgG_hits.tiff", units="in", width=6.5, height=3, res=300)
setwd("~/Documents/3_Parkinsons_disease/Autoantibody_Data/Tear_Auto_Validation_2022/")
ggplot(Y_combined_2, aes(x=Strain, y=`V Counts`, fill=Strain)) +
      geom_boxplot(outlier.shape = NA, width = 0.5, coef=1, varwidth=F, show.legend = T,
size=0.65, position = position_dodge(0.9)) +
      geom_jitter(color = "darkgray", alpha =0.5, size=2.5, show.legend = F, position =
position_jitterdodge(dodge.width=0.9))+
   facet_wrap(~Antigen, ncol=3, scales="free_x") +
      theme_minimal() +
      chart_design + ylim(2.5,12.5) +
      ylab("Log2 Normalized Intensity")  +
      #labs(title=colnames(Y[i]), hjust=0.5) +
      scale_fill_jco()
```

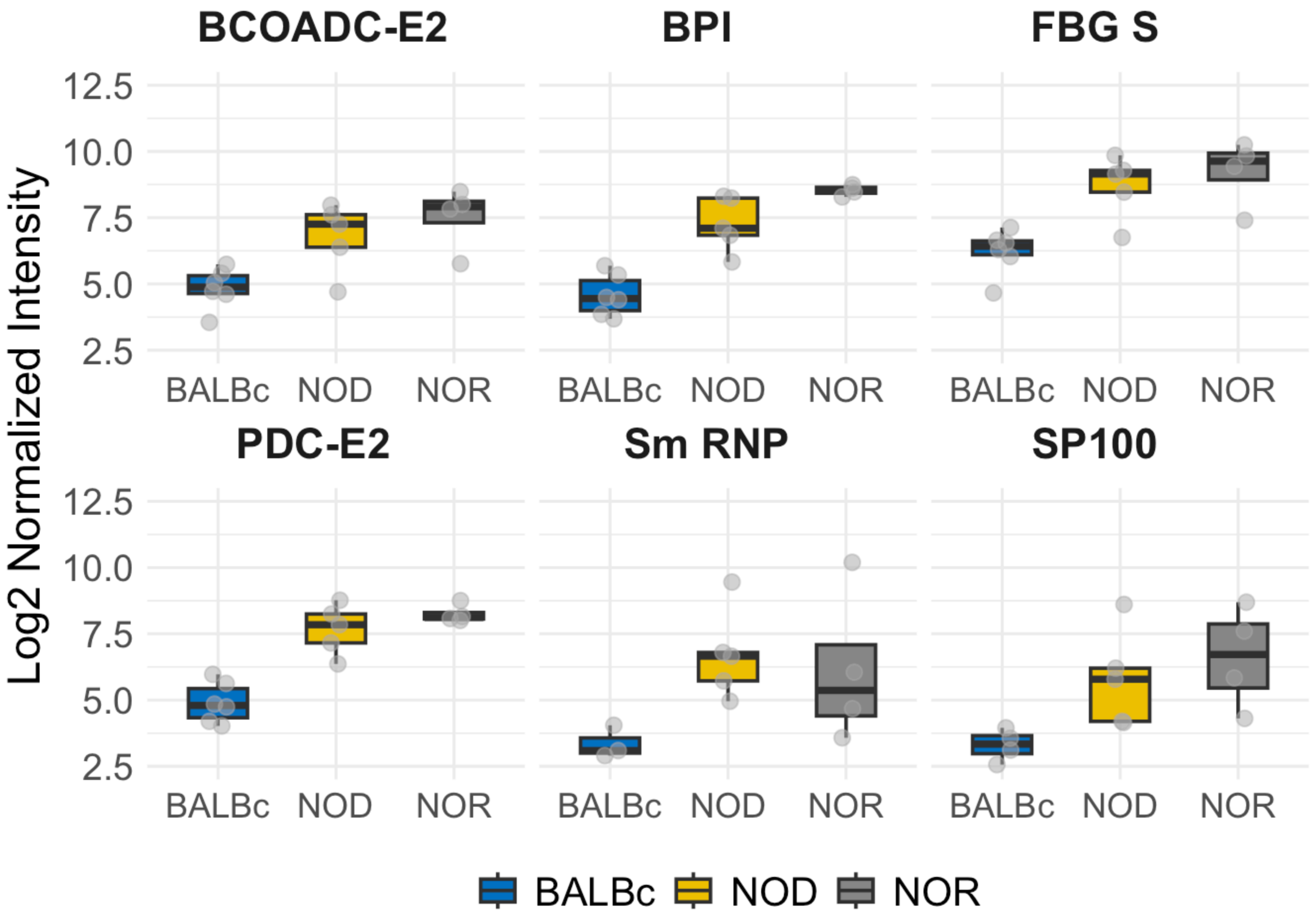
BCOADC-E2
BPI
FBG S
PDC-E2
Sm RNP
SP100
Log2 Normalized Intensity
12.5
10.0
7.5
5.0
2.5
BALBc
NOD
NOR
BALBc
NOD
NOR

```
#dev.off()
```
