# Supplementary material for: Serum and tear autoantibodies from NOD and NOR mice as potential diagnostic indicators of local and systemic inflammation in Sjögren’s disease
Source: Front Immunol. 2025 Jan 28;15:1516330. doi: 10.3389/fimmu.2024.1516330 (PMC11810956; doi:10.3389/fimmu.2024.1516330)
Supplement: Supplementary file 7 [file DataSheet7.pdf]

scRNAseq_NOD_LG

Shruti Kakan

2024-11-25

```
setwd("~/Documents/3_Parkinsons_disease/2019/scRNAseq/")

# Create a Seurat object for each sample
#for (file in c("NOD_H2b_filtered_feature_bc_matrix", "WT_male_filtered_feature_bc_matri
x"))
#
for (file in c("NOD_H2b_JH48", "NOD_H2b_JH50", "WT_male_JH47", "WT_male_JH49", "WT_male_
JH55")){
        seurat_data <- Read10X(data.dir = paste0("data/", file))
        seurat_obj <- CreateSeuratObject(counts = seurat_data,
                                         min.features = 100,
                                         project = file)
        assign(file, seurat_obj)
}
```

orig.ident: this often contains the sample identity if known, but will default to “SeuratProject” nCount_RNA: number of UMIs per cell nFeature_RNA: number of genes detected per cell

```
# Check the metadata in the new Seurat objects
head(NOD_H2b_JH48@meta.data)
```

```
##                       orig.ident nCount_RNA nFeature_RNA
## AAACCTGAGACAATAC-1 NOD_H2b_JH48       4115          299
## AAACCTGAGACTGGGT-1 NOD_H2b_JH48        452          306
## AAACCTGAGAGCTTCT-1 NOD_H2b_JH48        517          377
## AAACCTGAGCACCGCT-1 NOD_H2b_JH48        801          461
## AAACCTGAGGCATGGT-1 NOD_H2b_JH48      28424         1681
## AAACCTGAGGCCCGTT-1 NOD_H2b_JH48      34035         1499
```

```
head(WT_male_JH55@meta.data)
```

```
##                       orig.ident nCount_RNA nFeature_RNA
## AAACCTGAGGGCTCTC-1 WT_male_JH55       1734          169
## AAACCTGCACAGTCGC-1 WT_male_JH55       4227         1501
## AAACCTGGTAAGGGAA-1 WT_male_JH55       1060          127
## AAACCTGGTACCGGCT-1 WT_male_JH55     135413         4708
## AAACCTGGTAGAAGGA-1 WT_male_JH55        118          111
## AAACCTGGTCTTCAAG-1 WT_male_JH55       2782         1458
```

```
# Create a merged Seurat object
merged_seurat_NOD <- merge(x = NOD_H2b_JH48,
                       y = NOD_H2b_JH50,
                       add.cell.id = c("NOD_I", "NOD_II"))

merged_seurat_BALB <- merge(x = WT_male_JH47,
                       y = c(WT_male_JH49, WT_male_JH55),
                       add.cell.id = c("BALBc_I", "BALBc_II", "BALBcIII"))

# Concatenate the count matrices of both samples together
merged_seurat_NOD <- JoinLayers(merged_seurat_NOD)
merged_seurat_BALB <- JoinLayers(merged_seurat_BALB)

merged_seurat <- merge(x = merged_seurat_NOD,
                       y = merged_seurat_BALB,
                       add.cell.id = c("NOD", "BALBc"))

# Concatenate the count matrices of both samples together
merged_seurat <- JoinLayers(merged_seurat)

median(merged_seurat_NOD@meta.data$nCount_RNA)
```

```
## [1] 1692
```

```
median(merged_seurat_BALB@meta.data$nCount_RNA)
```

```
## [1] 1365
```

Generating Quality metrics

```
# Explore merged metadata
View(merged_seurat@meta.data)

# Add number of genes per UMI for each cell to metadata
merged_seurat$log10GenesPerUMI <- log10(merged_seurat$nFeature_RNA) / log10(merged_seura
t$nCount_RNA)
```

Calculating Mitochondrial Ratio

```
# Compute percent mito ratio
merged_seurat$mitoRatio <- PercentageFeatureSet(object = merged_seurat, pattern = "^mt-
")
merged_seurat$mitoRatio <- merged_seurat@meta.data$mitoRatio/100

head(merged_seurat$mitoRatio)
```

```
## NOD_NOD_I_AAACCTGAGACAATAC-1 NOD_NOD_I_AAACCTGAGACTGGGT-1
##                 0.0002430134                 0.0110619469
## NOD_NOD_I_AAACCTGAGAGCTTCT-1 NOD_NOD_I_AAACCTGAGCACCGCT-1
##                 0.0270793037                 0.0099875156
## NOD_NOD_I_AAACCTGAGGCATGGT-1 NOD_NOD_I_AAACCTGAGGCCCGTT-1
##                 0.0006684492                 0.0005288673
```

```
 # Create metadata dataframe
metadata <- merged_seurat@meta.data
# Add cell IDs to metadata
metadata$cells <- rownames(metadata)
# create a new column indicating which condition each cell is classfied under

# Create sample column
metadata$sample <- NA
metadata$sample[which(str_detect(metadata$cells, "^NOD_"))] <- "NOD"
metadata$sample[which(str_detect(metadata$cells, "^BALBc_"))] <- "BALBc"
# Rename columns
metadata <- metadata %>%
        dplyr::rename(seq_folder = orig.ident,
                      nUMI = nCount_RNA,
                      nGene = nFeature_RNA)
```

```
setwd("~/Documents/3_Parkinsons_disease/2019/scRNAseq/")

# Add metadata back to Seurat object
merged_seurat@meta.data <- metadata

# Create .RData object to load at any time
save(merged_seurat, file="data/merged_filtered_seurat.RData")
```

Assessing the quality metrics

```
# Visualize the number of cell counts per sample
metadata %>%
    ggplot(aes(x=sample, fill=sample)) +
    geom_bar() +
    theme_classic() +
    theme(axis.text.x = element_text(angle = 45, vjust = 1, hjust=1)) +
    theme(plot.title = element_text(hjust=0.5, face="bold")) +
    ggtitle("NCells")
```

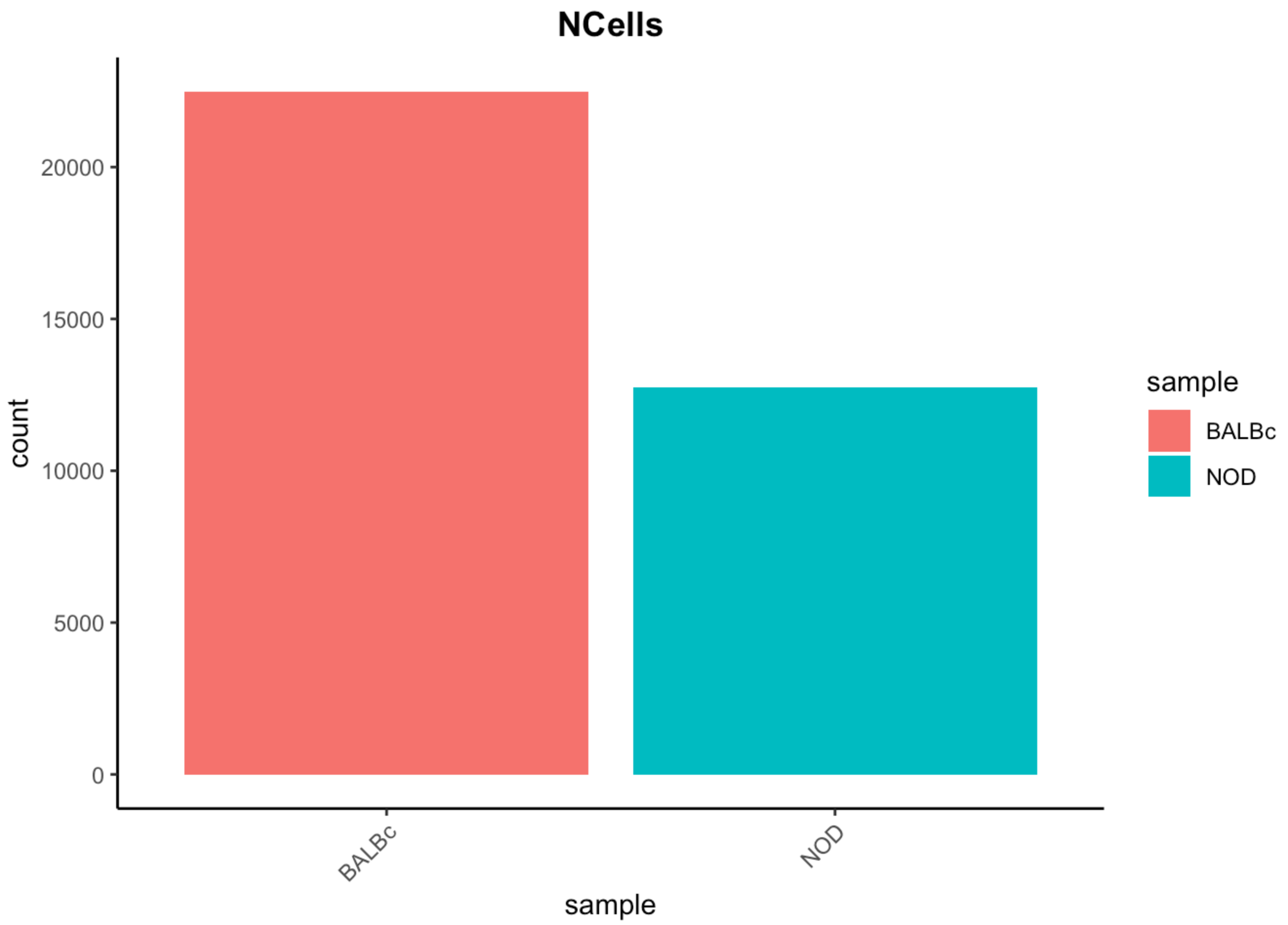

####

UMI counts (transcripts) per cell

```
# Visualize the number UMIs/transcripts per cell
metadata %>%
    ggplot(aes(color=sample, x=nUMI, fill= sample)) +
    geom_density(alpha = 0.2) +
    scale_x_log10() +
    theme_classic() +
    ylab("Cell density") +
    geom_vline(xintercept = 500)
```

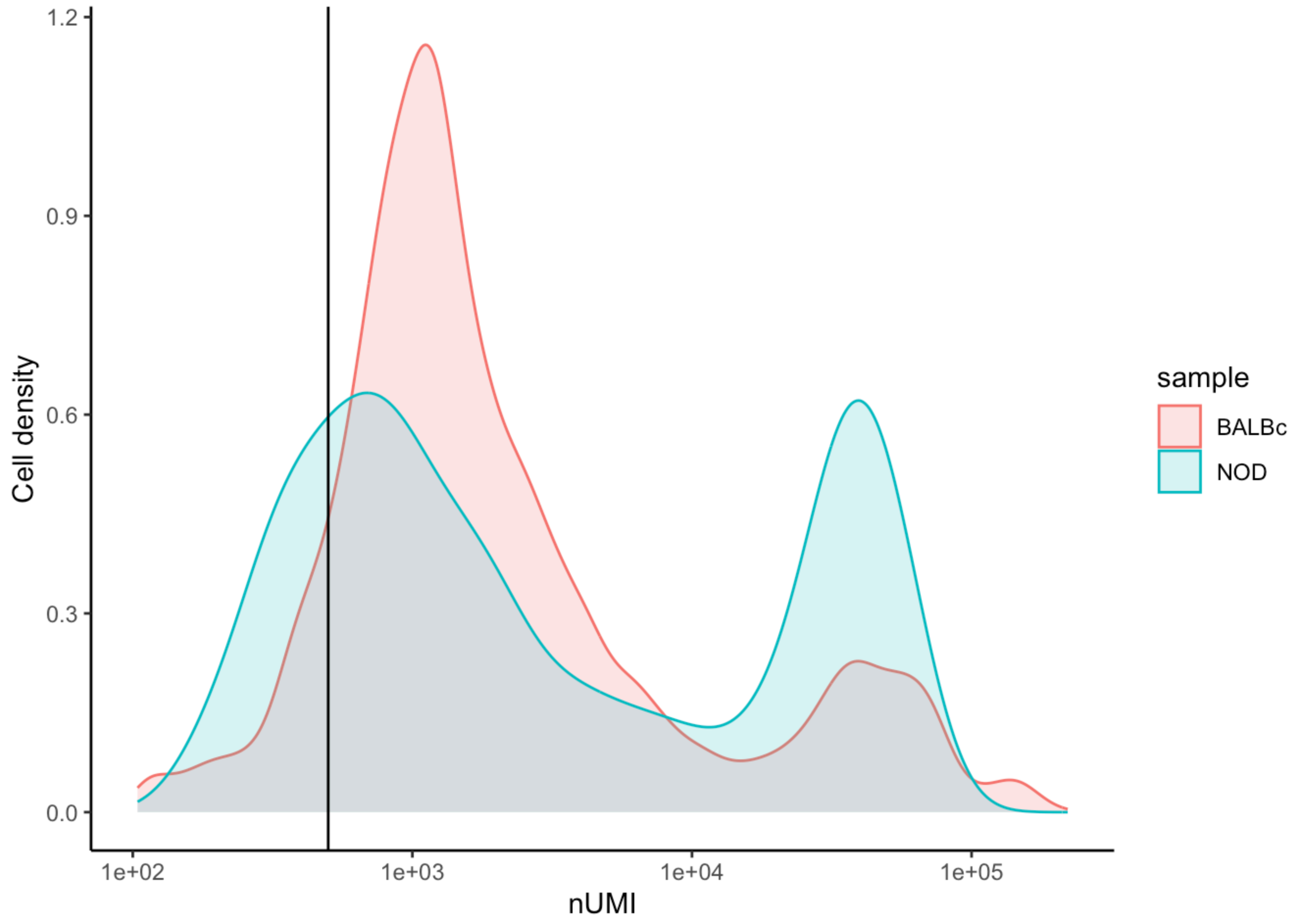

Genes detected per cell

```
# Visualize the distribution of genes detected per cell via histogram
metadata %>%
    ggplot(aes(color=sample, x=nGene, fill= sample)) +
    geom_density(alpha = 0.2) +
    theme_classic() +
    scale_x_log10() +
    geom_vline(xintercept = 300)
```

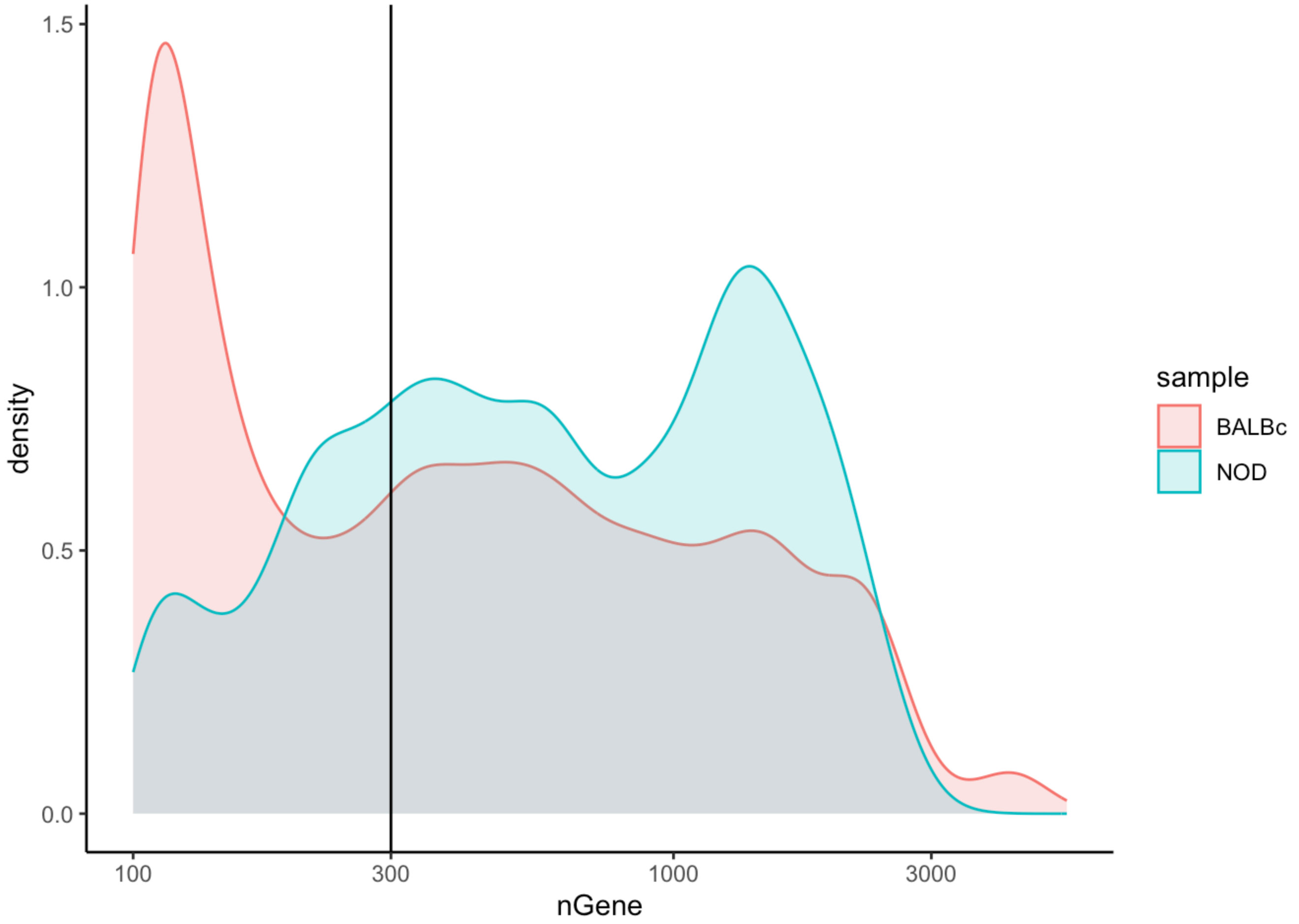

Complexity

```
# Visualize the overall complexity of the gene expression by visualizing the genes detec
ted per UMI (novelty score)
metadata %>%
    ggplot(aes(x=log10GenesPerUMI, color = sample, fill=sample)) +
    geom_density(alpha = 0.2) +
    theme_classic() +
    geom_vline(xintercept = 0.8)
```

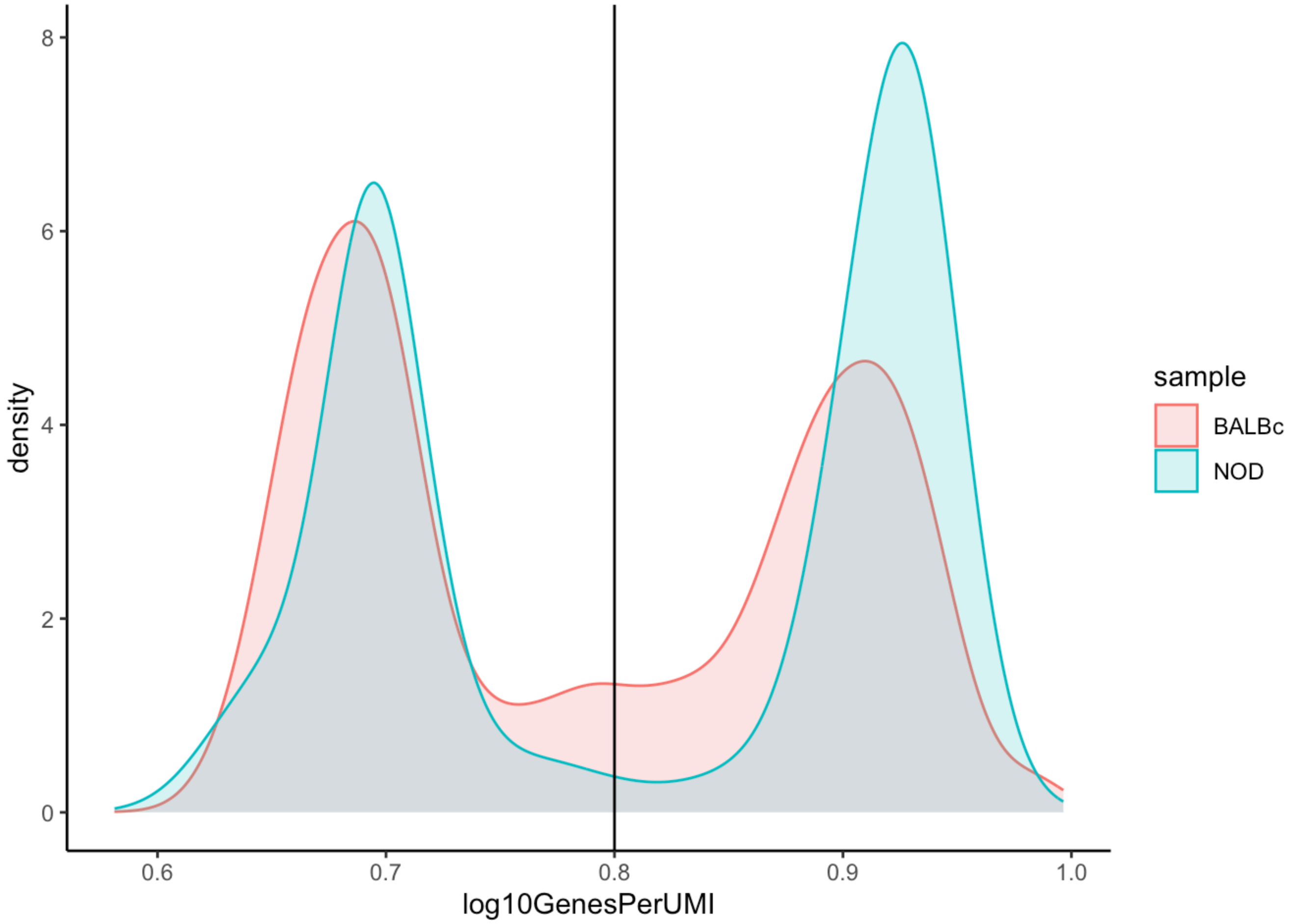

Mitochondrial counts ratio

```
# Visualize the distribution of mitochondrial gene expression detected per cell
metadata %>%
    ggplot(aes(color=sample, x=mitoRatio, fill=sample)) +
    geom_density(alpha = 0.2) +
    scale_x_log10() +
    theme_classic() +
    geom_vline(xintercept = 0.2)
```

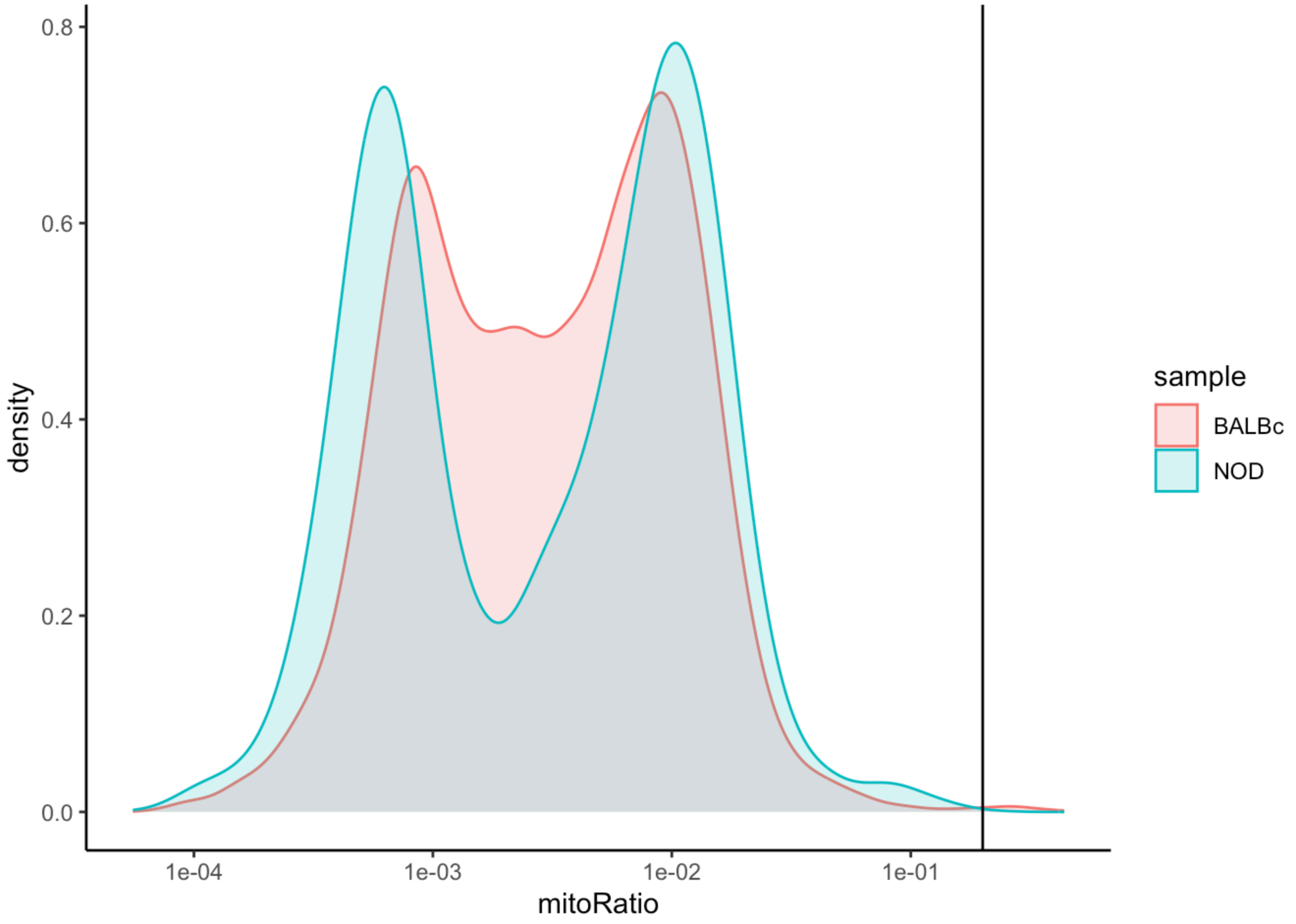

Joint filtering effects

```
# Visualize the correlation between genes detected and number of UMIs and determine whet
her strong presence of cells with low numbers of genes/UMIs
metadata %>%
    ggplot(aes(x=nUMI, y=nGene, color=mitoRatio)) +
    geom_point() +
    scale_colour_gradient(low = "gray90", high = "black") +
    stat_smooth(method=lm) +
    scale_x_log10() +
    scale_y_log10() +
    theme_classic() +
    geom_vline(xintercept = 500) +
    geom_hline(yintercept = 250) +
    facet_wrap(~sample)
```

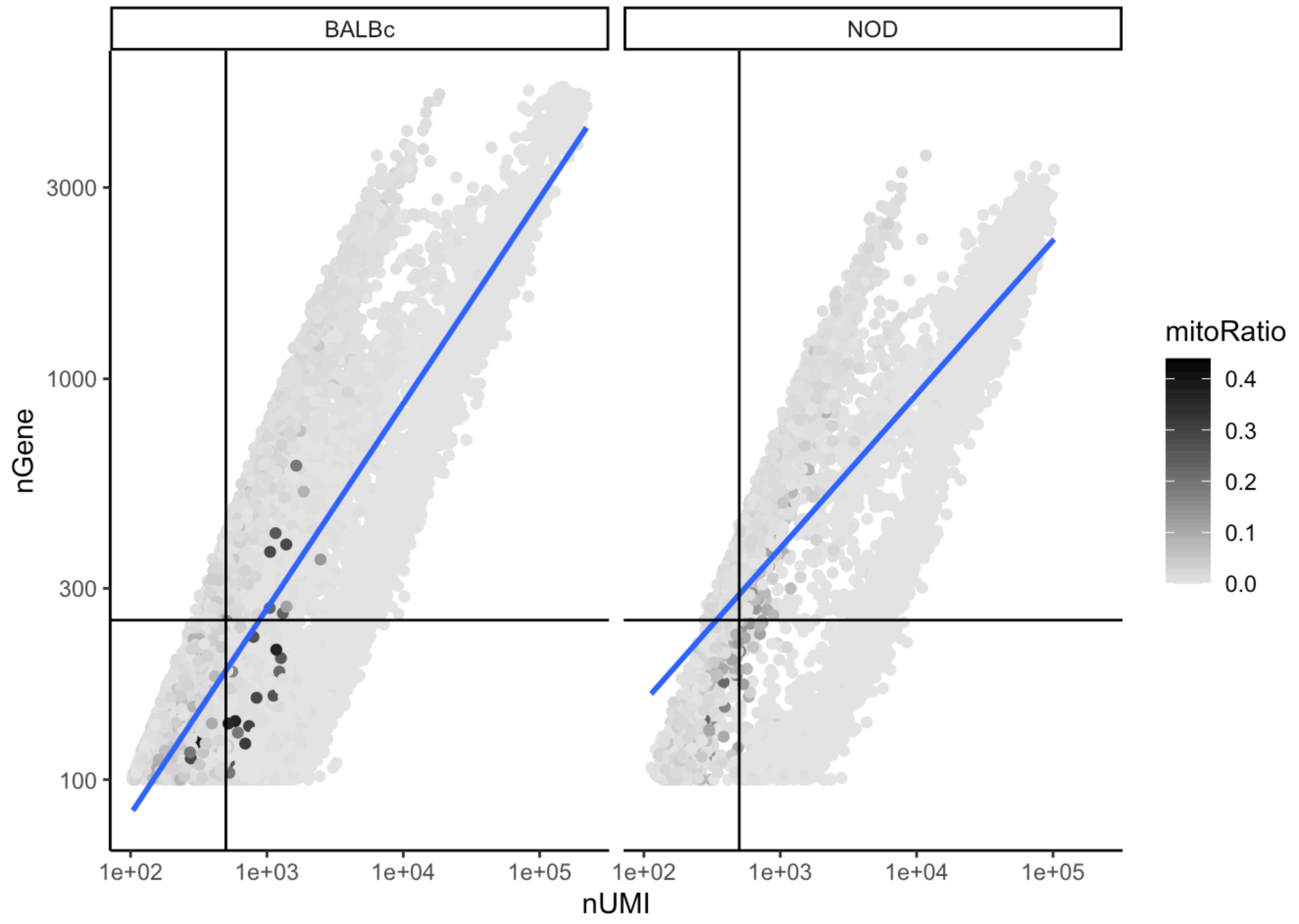

####

Cell-level filtering

- nUMI > 500
- nGene > 250
- log10GenesPerUMI > 0.8
- mitoRatio < 0.25

```
# Filter out low quality cells using selected thresholds - these will change with experi
ment
filtered_seurat <- subset(x = merged_seurat,
                         subset= (nUMI >= 450) &
                           (nGene >= 200) &
                           (log10GenesPerUMI > 0.8) &
                           (mitoRatio < 0.20))
```

Gene-level filtering

```
# Extract counts
counts <- GetAssayData(object = filtered_seurat, layer = "counts")

# Output a logical matrix specifying for each gene on whether or not there are more than
zero counts per cell
nonzero <- counts > 0
```

Keep only genes which are expressed in 10 or more cells.

```
# Sums all TRUE values and returns TRUE if more than 8 TRUE values per gene
keep_genes <- Matrix::rowSums(nonzero) >= 8

# Only keeping those genes expressed in more than 8 cells
filtered_counts <- counts[keep_genes, ]

# Reassign to filtered Seurat object
filtered_seurat <- CreateSeuratObject(filtered_counts, meta.data = filtered_seurat@meta.
data)
```

```
# Save filtered subset to new metadata
metadata_clean <- filtered_seurat@meta.data

# Add cell IDs to metadata_clean
metadata_clean$cells <- rownames(metadata_clean)

# create a new column indicating which condition each cell is classfied under

# Create sample column
#metadata_clean$sample <- NA
#metadata_clean$sample[which(str_detect(metadata_clean$cells, "^NOD_"))] <- "NOD"
#metadata_clean$sample[which(str_detect(metadata_clean$cells, "^BALBc_"))] <- "BALBc"

# Rename columns
#metadata_clean <- metadata_clean %>%
#        dplyr::rename(seq_folder = orig.ident,
#                      nUMI = nCount_RNA,
#                      nGene = nFeature_RNA)
```

```
setwd("~/Documents/3_Parkinsons_disease/2019/scRNAseq/")

# Add metadata back to Seurat object
filtered_seurat@meta.data <- metadata_clean

# Create .RData object to load at any time
save(filtered_seurat, file="data/seurat_filtered.RData")
```

Assessing the quality metrics

```
# Visualize the number of cell counts per sample
metadata_clean %>%
    ggplot(aes(x=sample, fill=sample)) +
    geom_bar() +
    theme_classic() +
    theme(axis.text.x = element_text(angle = 45, vjust = 1, hjust=1)) +
    theme(plot.title = element_text(hjust=0.5, face="bold")) +
    ggtitle("NCells")
```

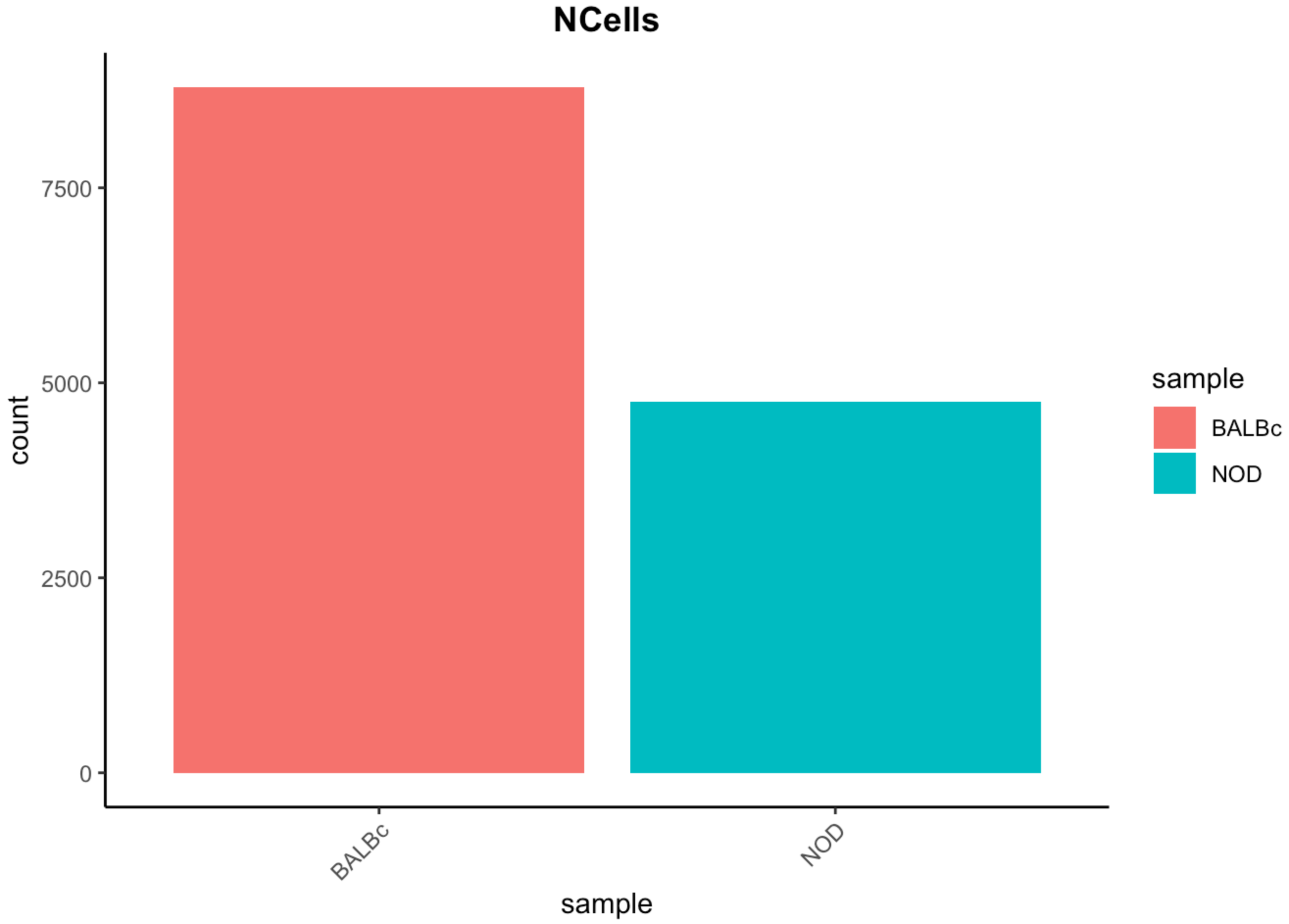

UMI counts (transcripts) per cell

```
# Visualize the number UMIs/transcripts per cell
metadata_clean %>%
    ggplot(aes(color=sample, x=nUMI, fill= sample)) +
    geom_density(alpha = 0.2) +
    scale_x_log10() +
    theme_classic() +
    ylab("Cell density") +
    geom_vline(xintercept = 500)
```

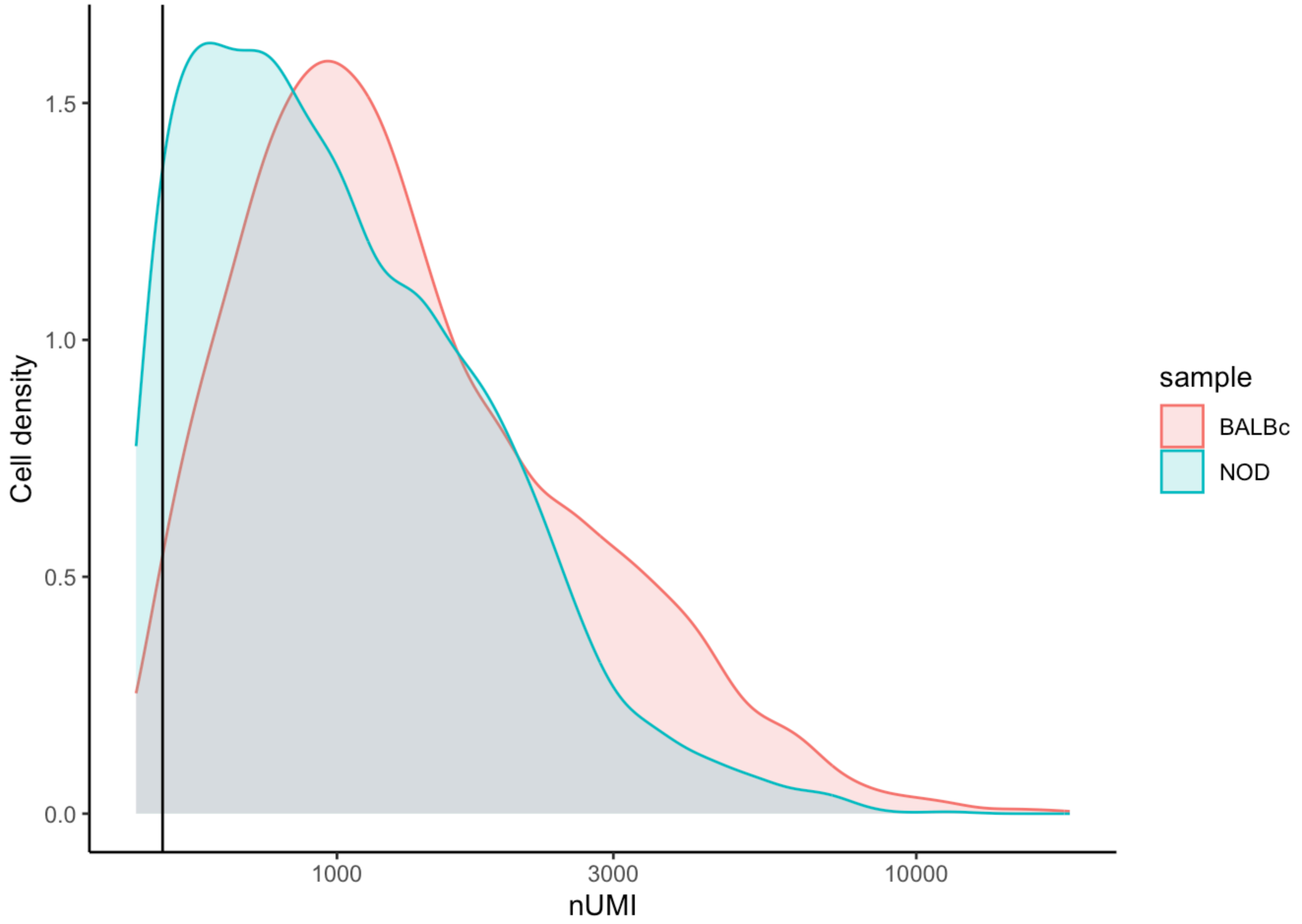

Genes detected per cell

```
# Visualize the distribution of genes detected per cell via histogram
metadata_clean %>%
    ggplot(aes(color=sample, x=nGene, fill= sample)) +
    geom_density(alpha = 0.2) +
    theme_classic() +
    scale_x_log10() +
    geom_vline(xintercept = 300)
```

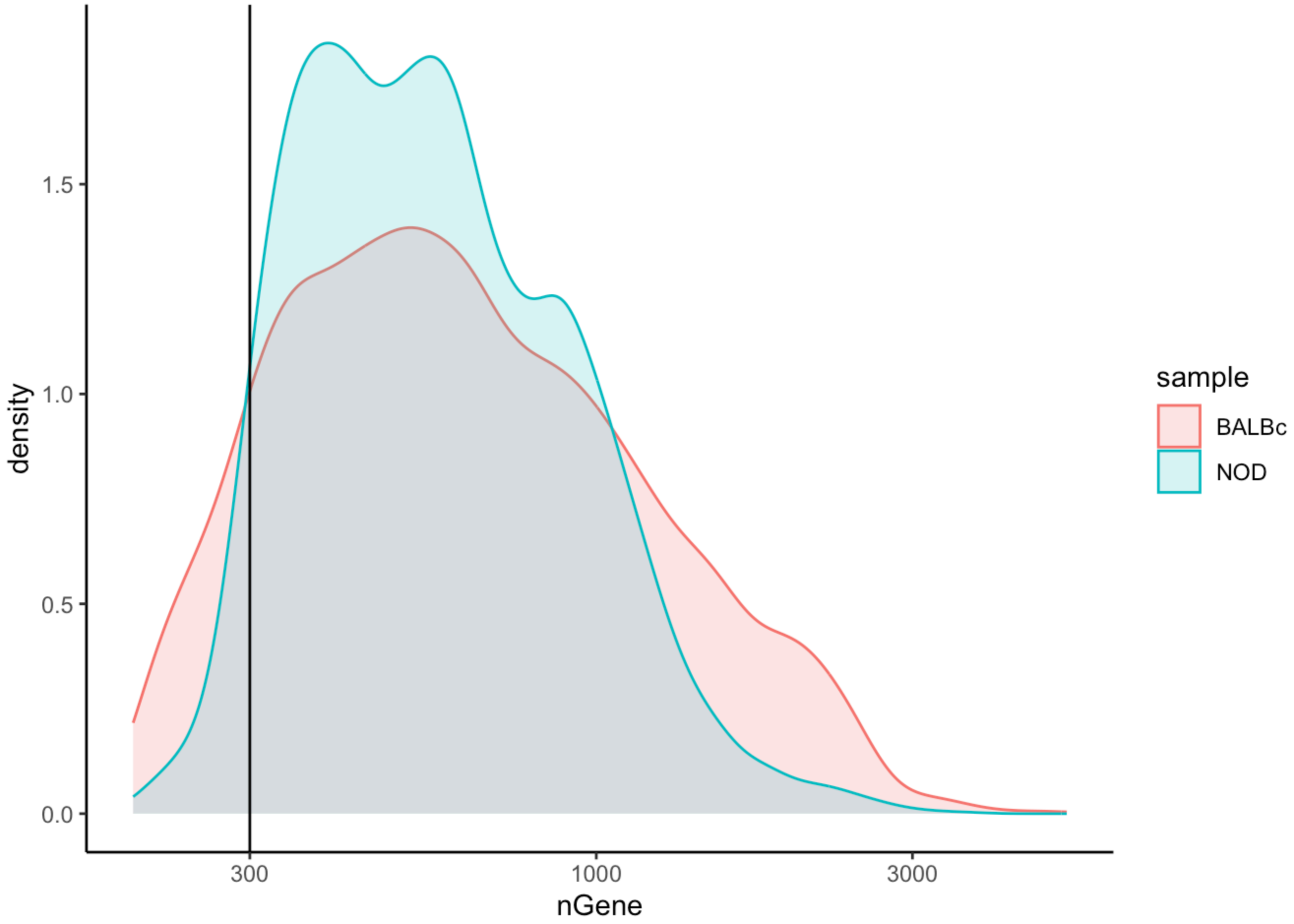

Complexity

```
# Visualize the overall complexity of the gene expression by visualizing the genes detec
ted per UMI (novelty score)
metadata_clean %>%
    ggplot(aes(x=log10GenesPerUMI, color = sample, fill=sample)) +
    geom_density(alpha = 0.2) +
    theme_classic() +
    geom_vline(xintercept = 0.8)
```

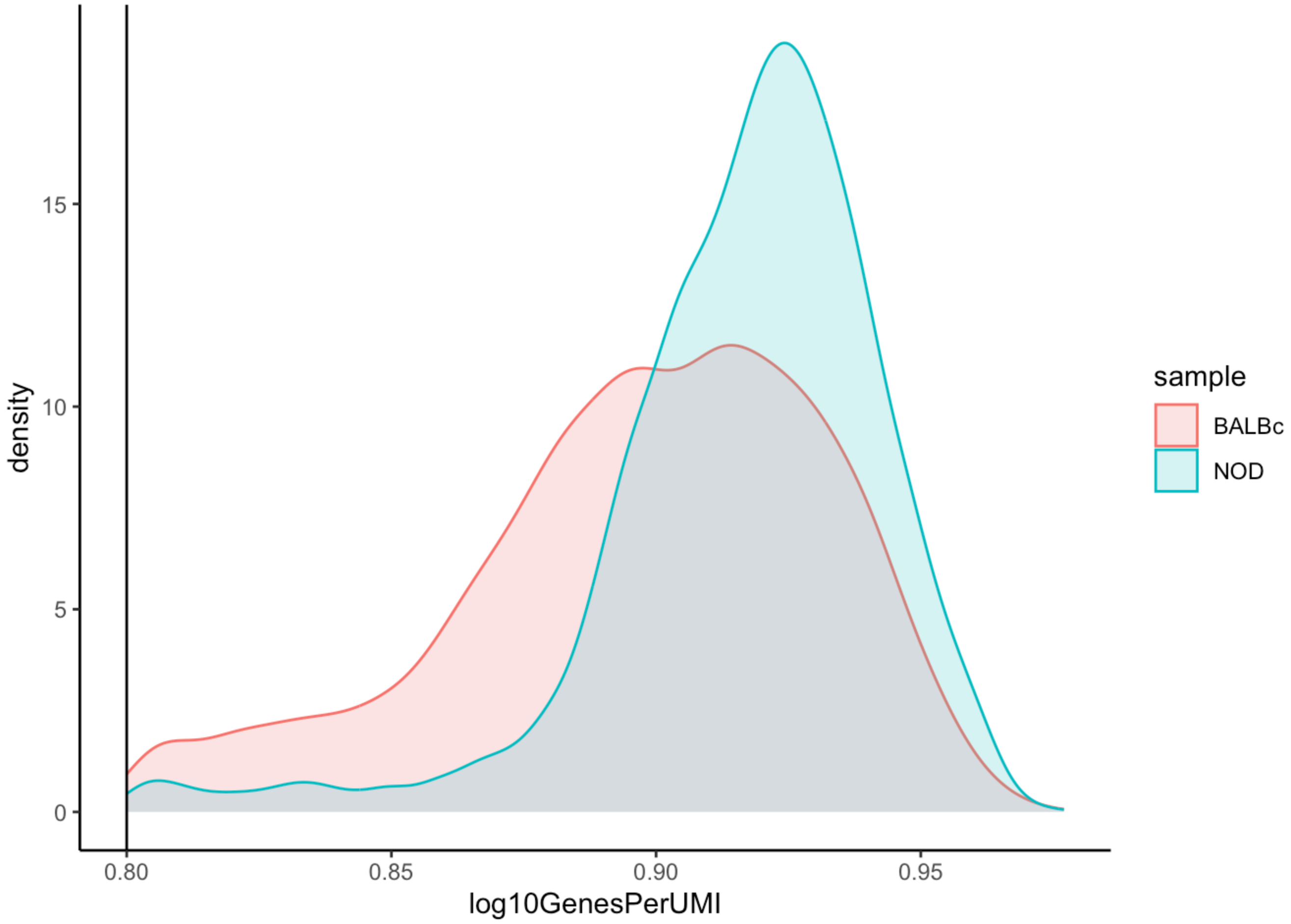

Mitochondrial counts ratio

```
# Visualize the distribution of mitochondrial gene expression detected per cell
metadata_clean %>%
    ggplot(aes(color=sample, x=mitoRatio, fill=sample)) +
    geom_density(alpha = 0.2) +
    #scale_x_log10() +
    theme_classic() +
    geom_vline(xintercept = 0.2)
```

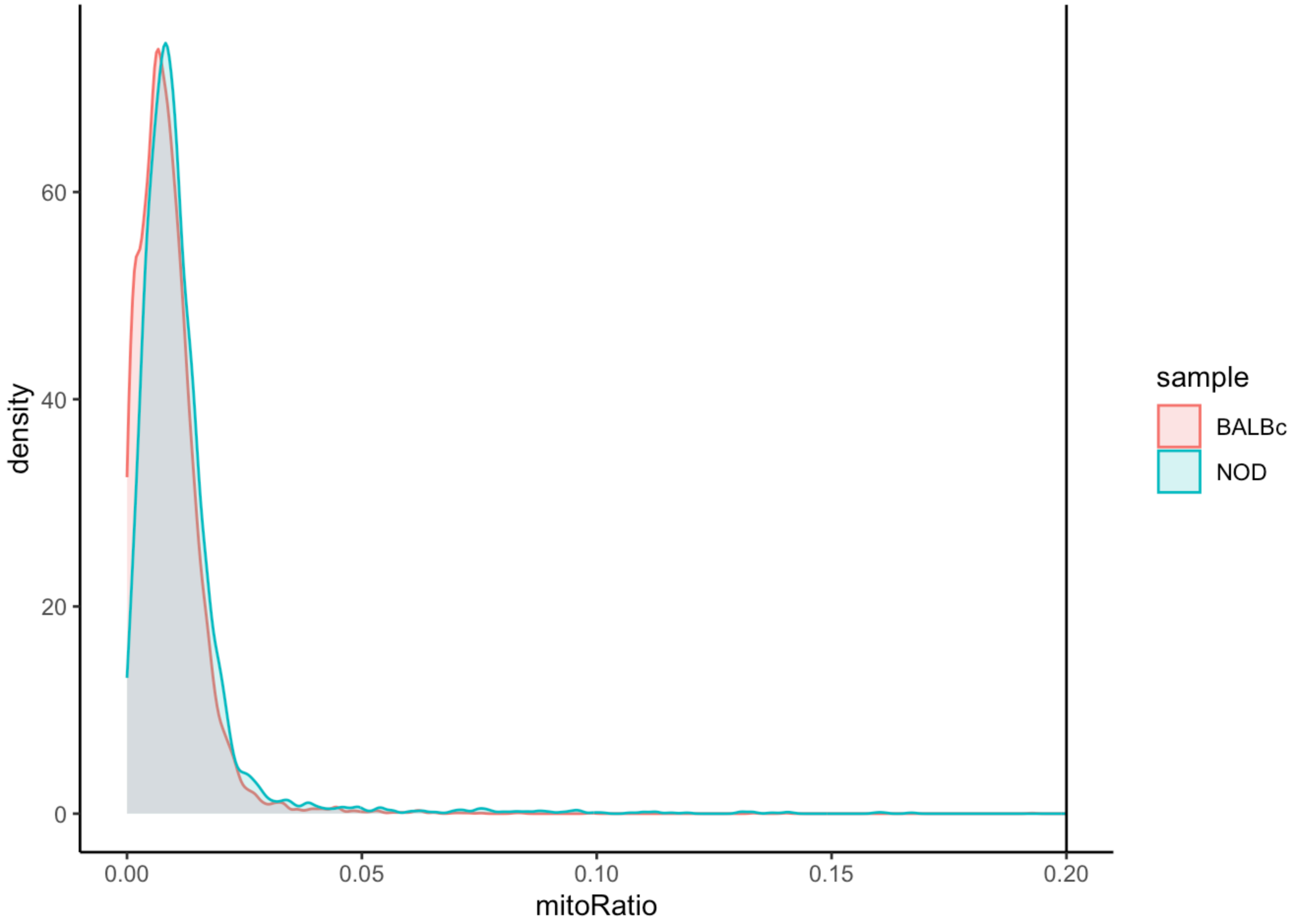

Joint filtering effects

```
# Visualize the correlation between genes detected and number of UMIs and determine whet
her strong presence of cells with low numbers of genes/UMIs
metadata_clean %>%
    ggplot(aes(x=nUMI, y=nGene, color=mitoRatio)) +
    geom_point() +
    scale_colour_gradient(low = "gray90", high = "black") +
    stat_smooth(method=lm) +
    scale_x_log10() +
    scale_y_log10() +
    theme_classic() +
    geom_vline(xintercept = 500) +
    geom_hline(yintercept = 250) +
    facet_wrap(~sample)
```

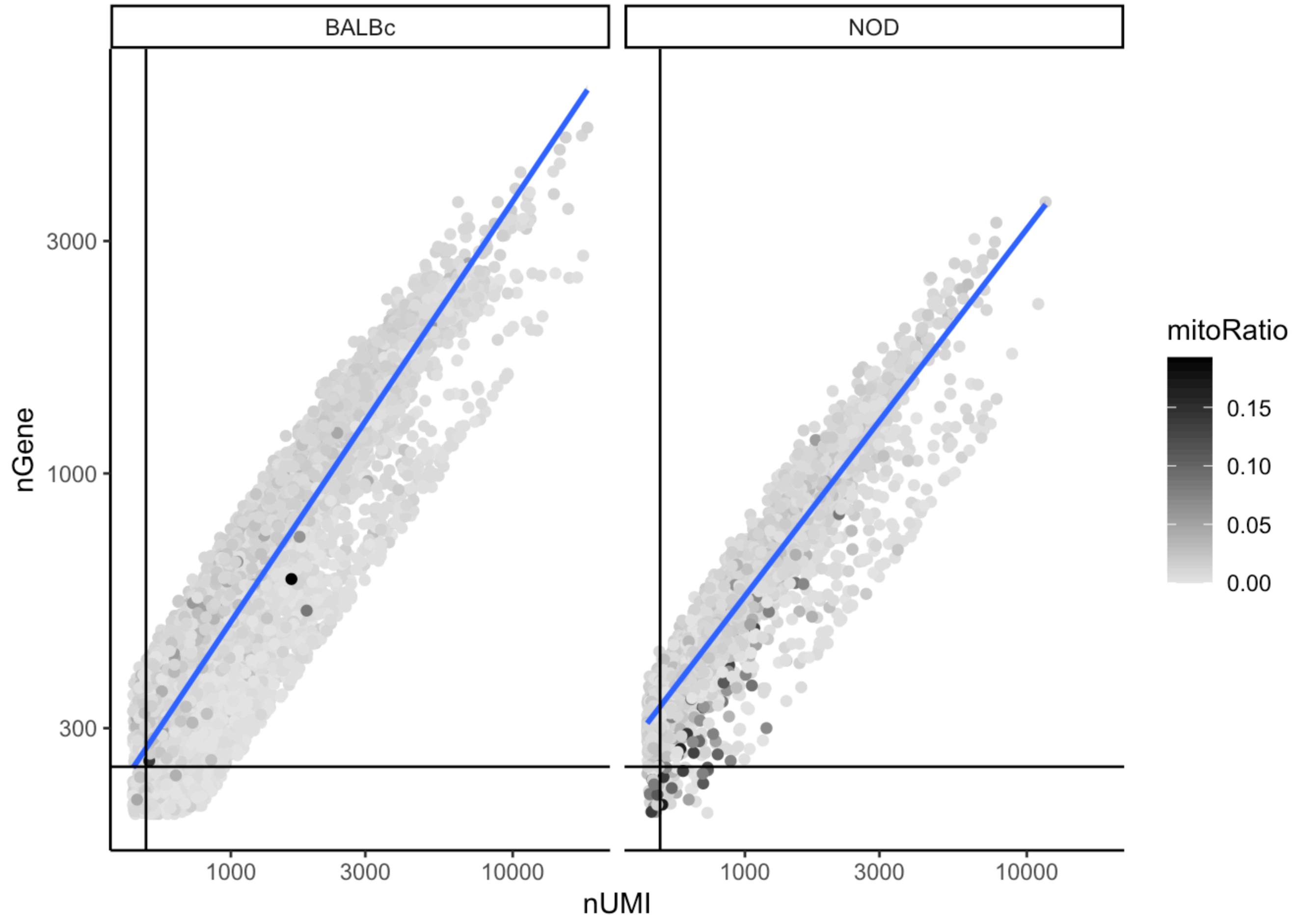


```
# Create .RData object to load at any time
setwd("~/Documents/3_Parkinsons_disease/2019/scRNAseq/")
save(filtered_seurat, file="data/seurat_filtered.RData")
```

Normalization

Apply a simple normalization.

```
# Normalize the counts
seurat_phase <- NormalizeData(filtered_seurat)
```

Regressing out Variation from cell cycle variation

Evaluating effects of cell cycle

```
# Perform cell cycle scoring
seurat_phase <- CellCycleScoring(seurat_phase,
                                 g2m.features = g2m_genes,
                                 s.features = s_genes)
# View cell cycle scores and phases assigned to cells
View(seurat_phase@meta.data)
```

Determine whether cell cycle is a major source of variation in our dataset using PCA.

```
# Identify the most variable genes if it hasn't been run
seurat_phase <- FindVariableFeatures(seurat_phase,
                     selection.method = "vst",
                     nfeatures = 3000,
                     verbose = FALSE)
# Scale the counts
seurat_phase <- ScaleData(seurat_phase)
```

```
# Identify the 15 most highly variable genes
ranked_variable_genes <- VariableFeatures(seurat_phase)
top_genes <- ranked_variable_genes[1:25]

# Plot the average expression and variance of these genes
# With labels to indicate which genes are in the top 15
p <- VariableFeaturePlot(seurat_phase)
LabelPoints(plot = p, points = top_genes, repel = TRUE)
```

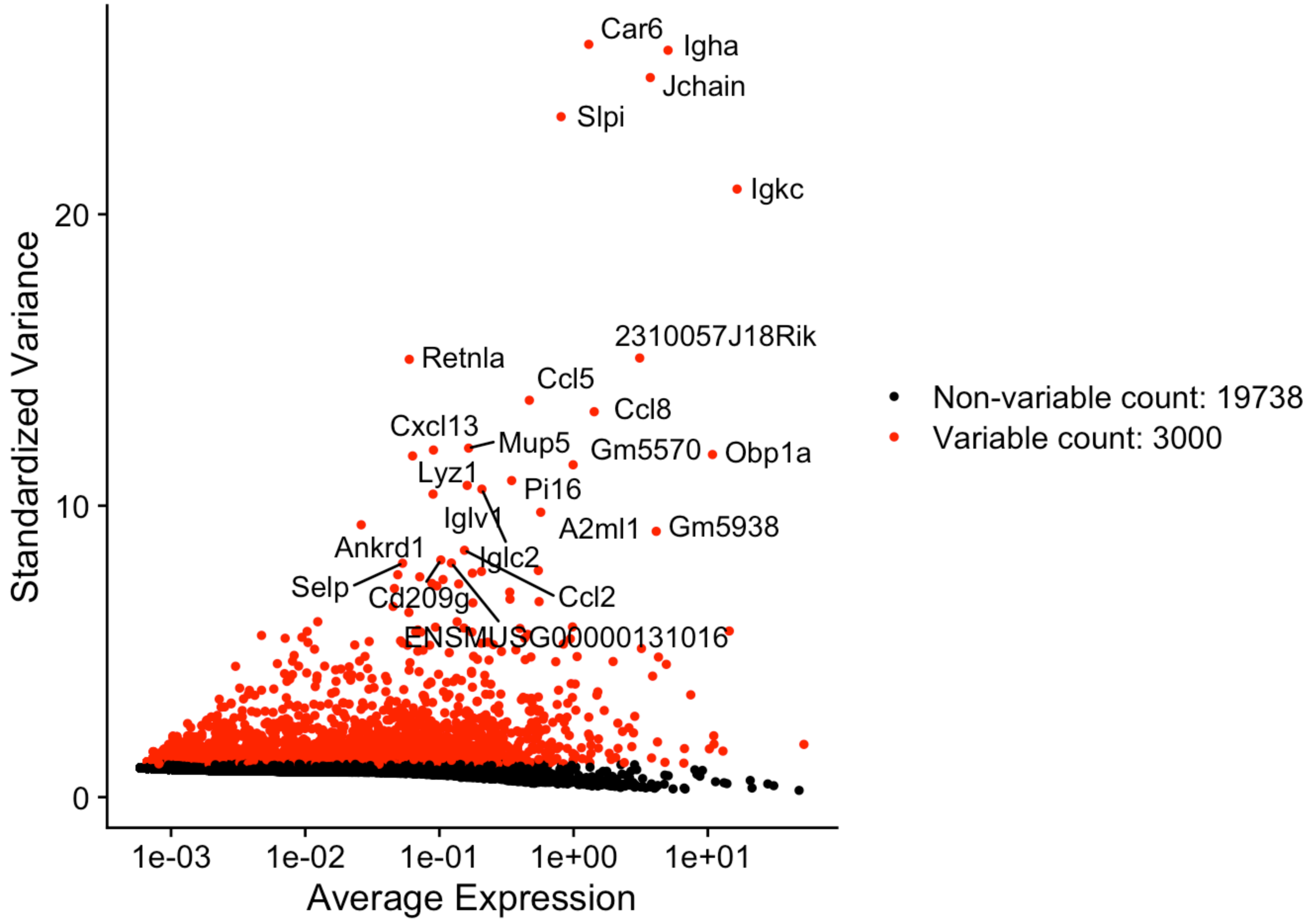

```
# Perform PCA and color by cell cycle phase
seurat_phase <- RunPCA(seurat_phase)

# Visualize the PCA, grouping by cell cycle phase
DimPlot(seurat_phase,
        reduction = "pca",
        group.by= "Phase",
        split.by = "Phase")
```

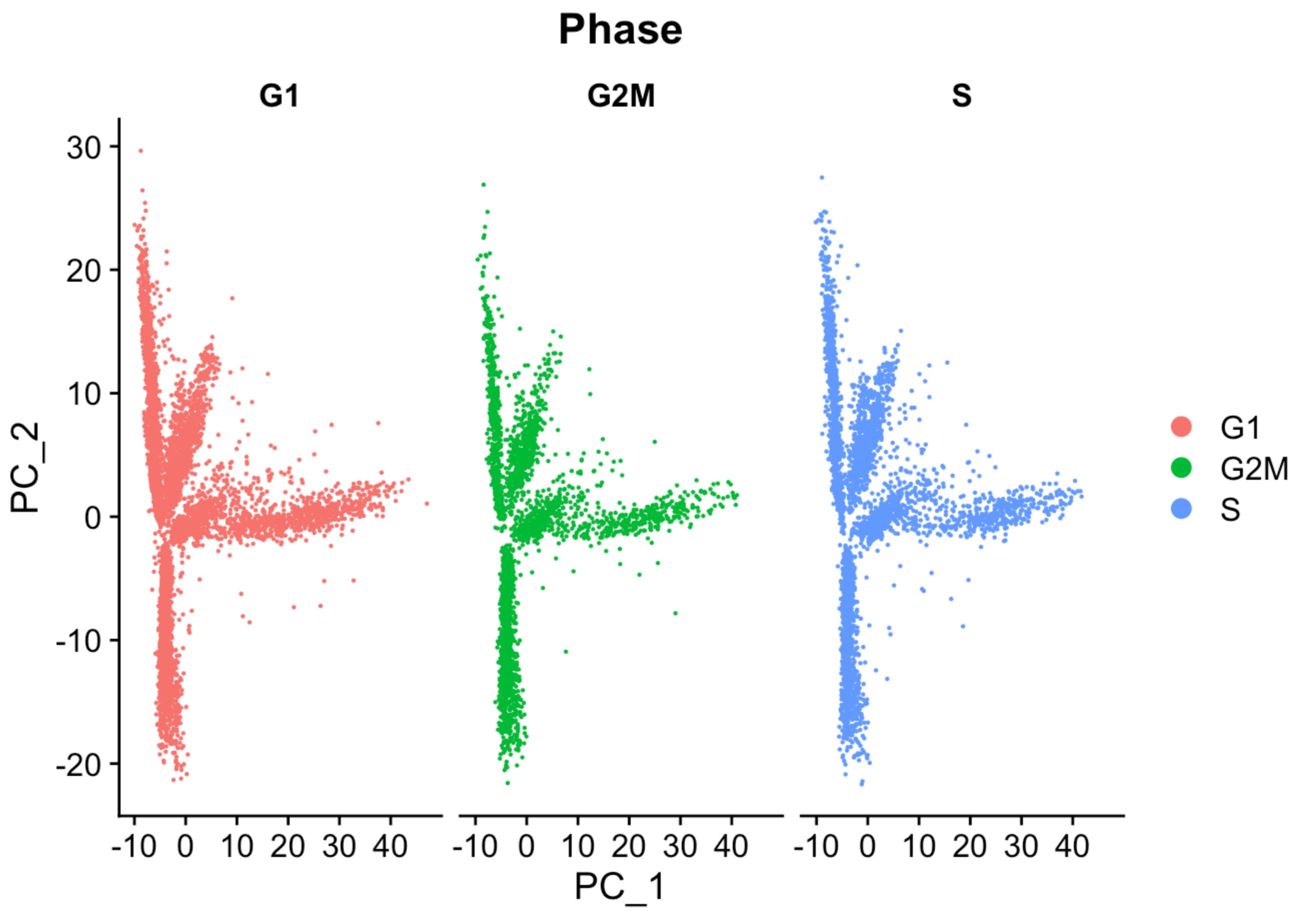


```
# Visualize the PCA, grouping by cell cycle phase
DimPlot(seurat_phase,
        reduction = "pca",
        group.by= "Phase",
        split.by = "sample")
```

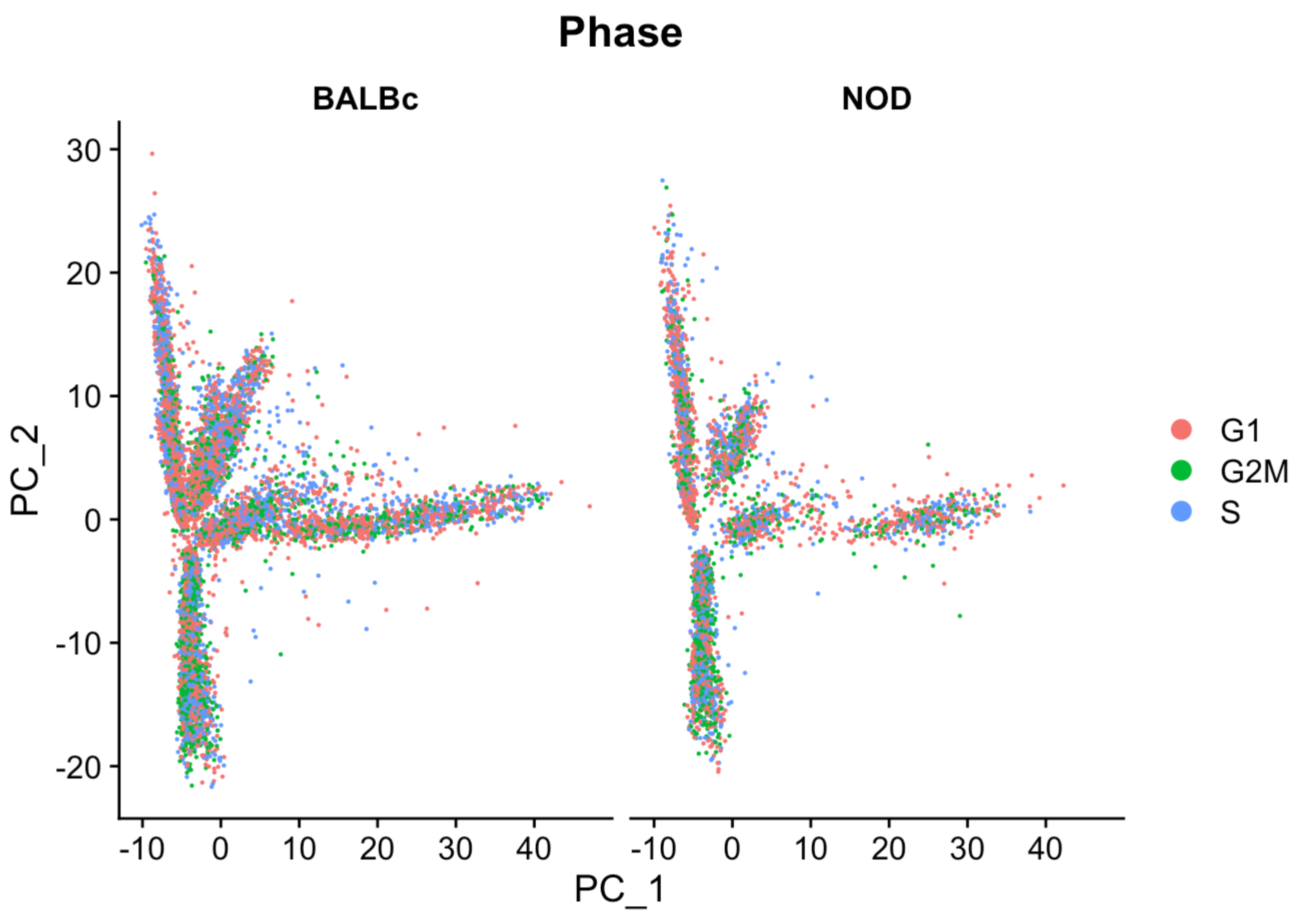

Evaluating effects of mitochondrial expression

```
# Check quartile values
summary(seurat_phase@meta.data$mitoRatio)
```

```
##     Min.  1st Qu.   Median     Mean  3rd Qu.     Max. 
## 0.000000 0.004902 0.008382 0.009825 0.012484 0.192683
```

```
# Turn mitoRatio into categorical factor vector based on quartile values
seurat_phase@meta.data$mitoFr <- cut(seurat_phase@meta.data$mitoRatio, 
                   breaks=c(-Inf, 0.00944, 0.0159, 0.0240, Inf), 
                   labels=c("Low","Medium","Medium high", "High"))


# Perform PCA and color by cell cycle phase & MitoFr
seurat_phase <- RunPCA(seurat_phase)
# Visualize the PCA, grouping by cell cycle phase & MitoFr
DimPlot(seurat_phase,
        reduction = "pca",
        group.by= "sample",
        split.by = "mitoFr")
```

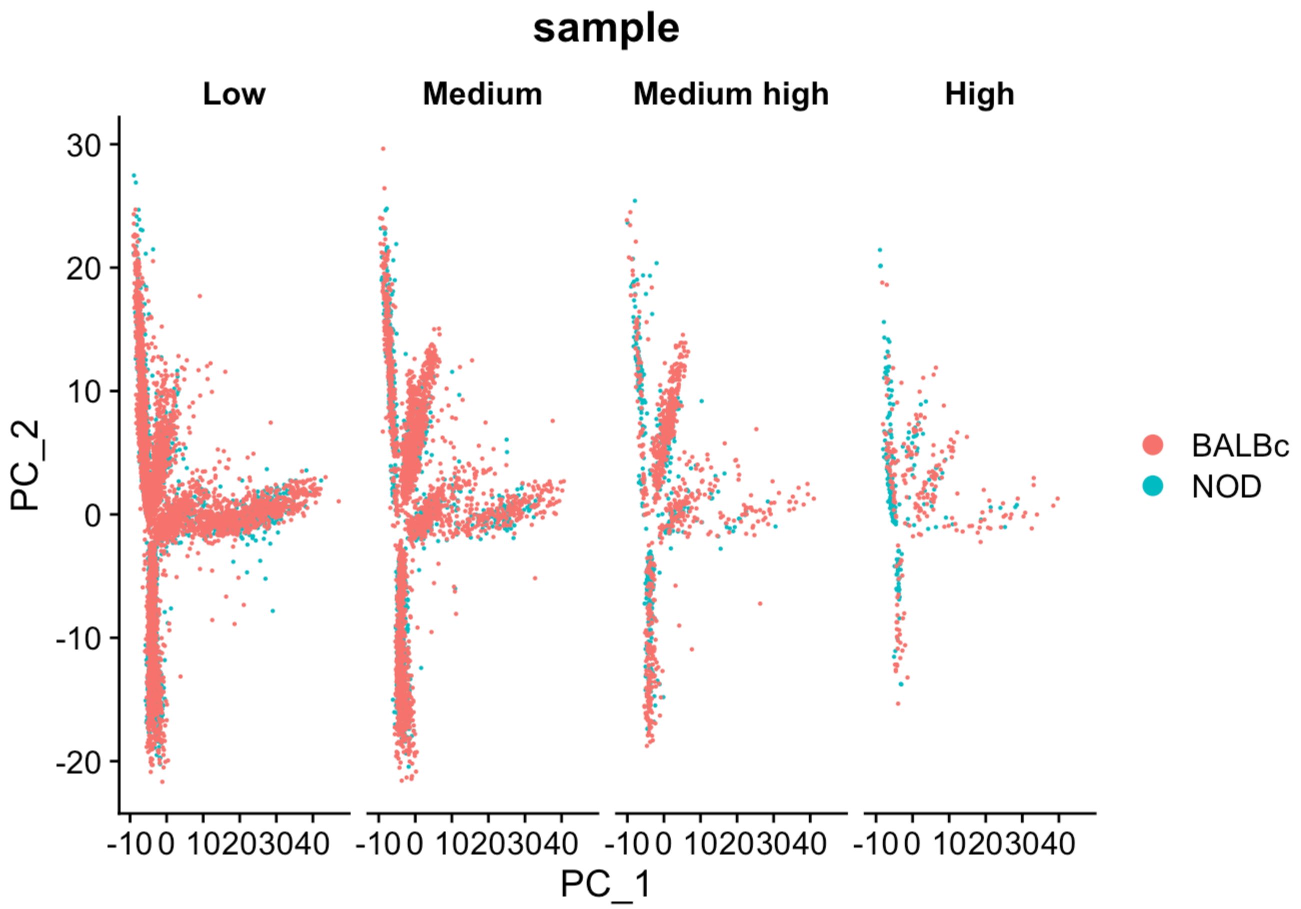


```
# Split seurat object by condition to perform cell cycle scoring and SCT on all samples
split_seurat <- SplitObject(seurat_phase, split.by = "sample")

options(future.globals.maxSize = 8000 * 1024^2)

#This takes 10 minutes to run
for (i in 1:length(split_seurat)) {
    split_seurat[[i]] <- SCTransform(split_seurat[[i]], vst.flavor = "v2", ncells=4000)
}

# Check which assays are stored in objects
split_seurat$BALBc@assays
```

```
## $RNA
## Assay (v5) data with 22738 features for 8792 cells
## Top 10 variable features:
##  Car6, Igha, Jchain, Slpi, Igkc, 2310057J18Rik, Retnla, Ccl5, Ccl8, Mup5
## Layers:
##  counts, data, scale.data
##
## $SCT
## SCTAssay data with 22230 features for 8792 cells, and 1 SCTModel(s)
## Top 10 variable features:
##  Wfdc18, Cd74, Igkc, Jchain, Igha, Ltf, Gsn, Apoe, Ccl8, Dcn
```

```
split_seurat$NOD@assays
```

```
## $RNA
## Assay (v5) data with 22738 features for 4762 cells
## Top 10 variable features:
##  Car6, Igha, Jchain, Slpi, Igkc, 2310057J18Rik, Retnla, Ccl5, Ccl8, Mup5
## Layers:
##  counts, data, scale.data
##
## $SCT
## SCTAssay data with 19035 features for 4762 cells, and 1 SCTModel(s)
## Top 10 variable features:
##  Wfdc18, Ccl8, Ltf, Gsn, Apoe, Dcn, Igkc, Ccl5, Jchain, Cd74
```

```
# Save the split seurat object
setwd("~/Documents/3_Parkinsons_disease/2019/scRNAseq/")
saveRDS(split_seurat, "data/split_seurat.rds")
```

Integrate or align samples across conditions using shared highly variable genes

```
# Select the most variable features to use for integration
setwd("~/Documents/3_Parkinsons_disease/2019/scRNAseq/")
split_seurat <- readRDS("data/split_seurat.rds")

integ_features <- SelectIntegrationFeatures(object.list = split_seurat, nfeatures = 300
0)

# Prepare the SCT list object for integration
split_seurat <- PrepSCTIntegration(object.list = split_seurat,
                                   anchor.features = integ_features)

# Find best buddies - can take a while to run
integ_anchors <- FindIntegrationAnchors(object.list = split_seurat,
                                        normalization.method ="SCT",
                                        anchor.features = integ_features)
```

```
# Integrate across conditions
seurat_integrated <- IntegrateData(anchorset = integ_anchors, normalization.method = "SC
T")
```

```
## [1] 1
```

```
## [1] 2
```

```
#seurat_integrated <- IntegrateLayers(object = split_seurat, method = "CCAIntegration",
orig = "pca", new.reduction="integrated.cca", verbose = FALSE)

# Rejoin the layers in the RNA assay that we split earlier
seurat_integrated[["RNA"]] <- JoinLayers(seurat_integrated[["RNA"]])
```

```
# Run PCA
seurat_integrated <- RunPCA(object = seurat_integrated)

# Plot PCA
PCAPlot(seurat_integrated,
        split.by = "sample")
```

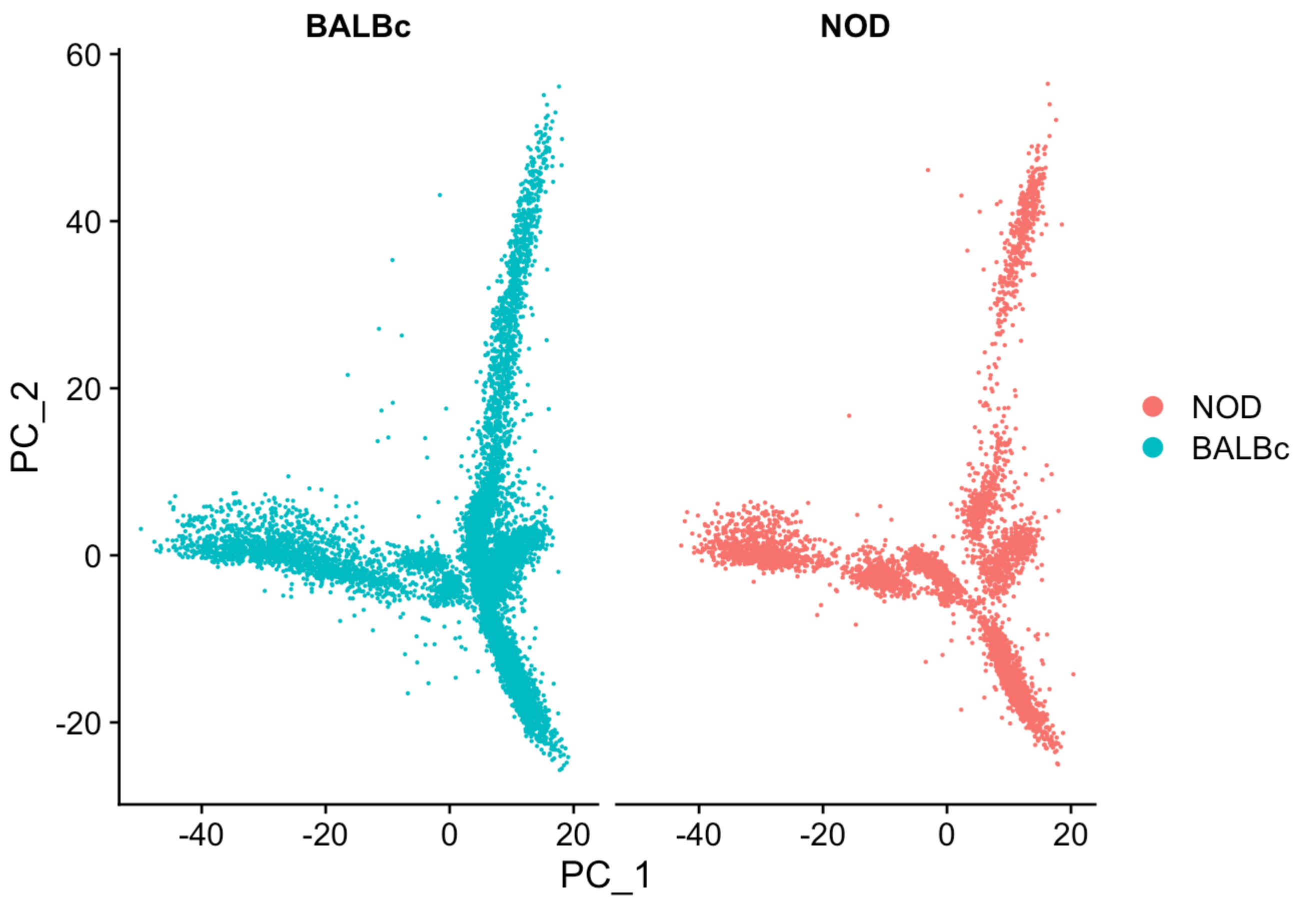

UMAP

```
# Set seed
set.seed(123456)

# Run UMAP
seurat_phase <- RunPCA(object = seurat_phase)
seurat_phase <- RunUMAP(seurat_phase,
                             dims = 1:50,
                 reduction = "pca")
#JackStraw(object = seurat_phase)

# Plot UMAP
DimPlot(seurat_phase)
```

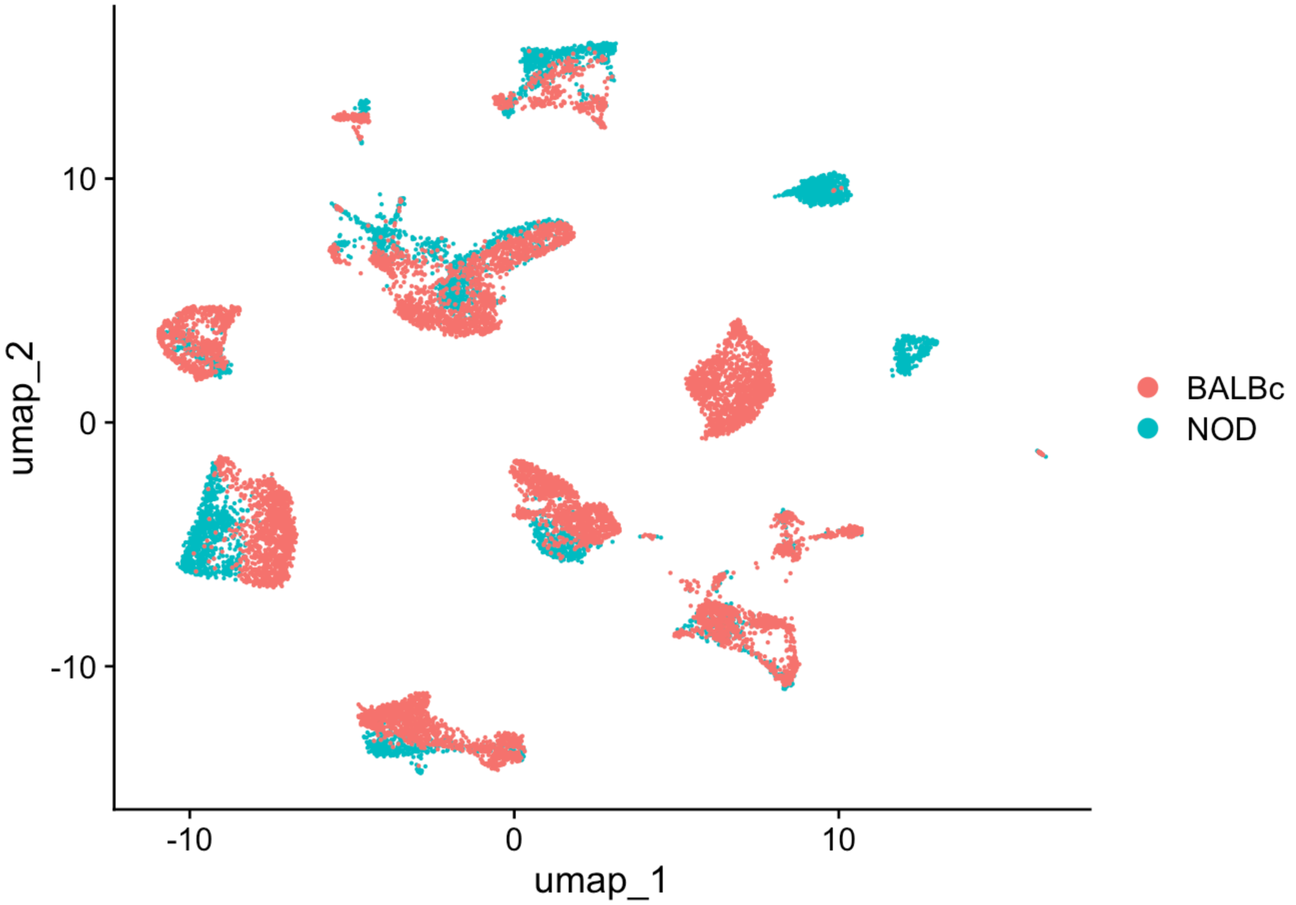


```
# Run UMAP
# Set seed
set.seed(123456)
seurat_integrated <- RunUMAP(seurat_integrated,
                             dims = 1:30,
                 reduction = "pca")

# Plot UMAP
DimPlot(seurat_integrated)
```

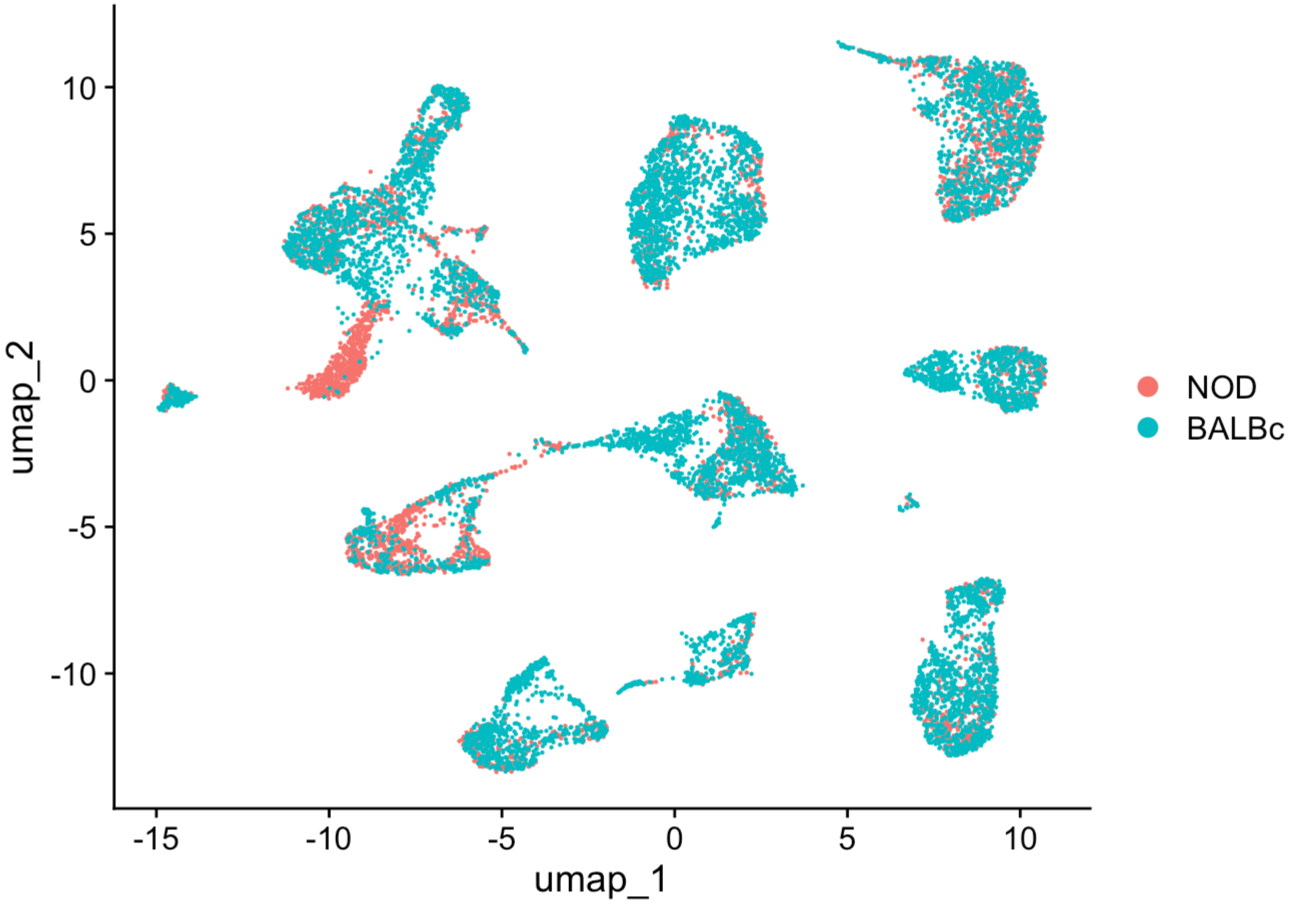


```
# Plot UMAP split by sample
DimPlot(seurat_integrated,
        split.by = "sample")
```

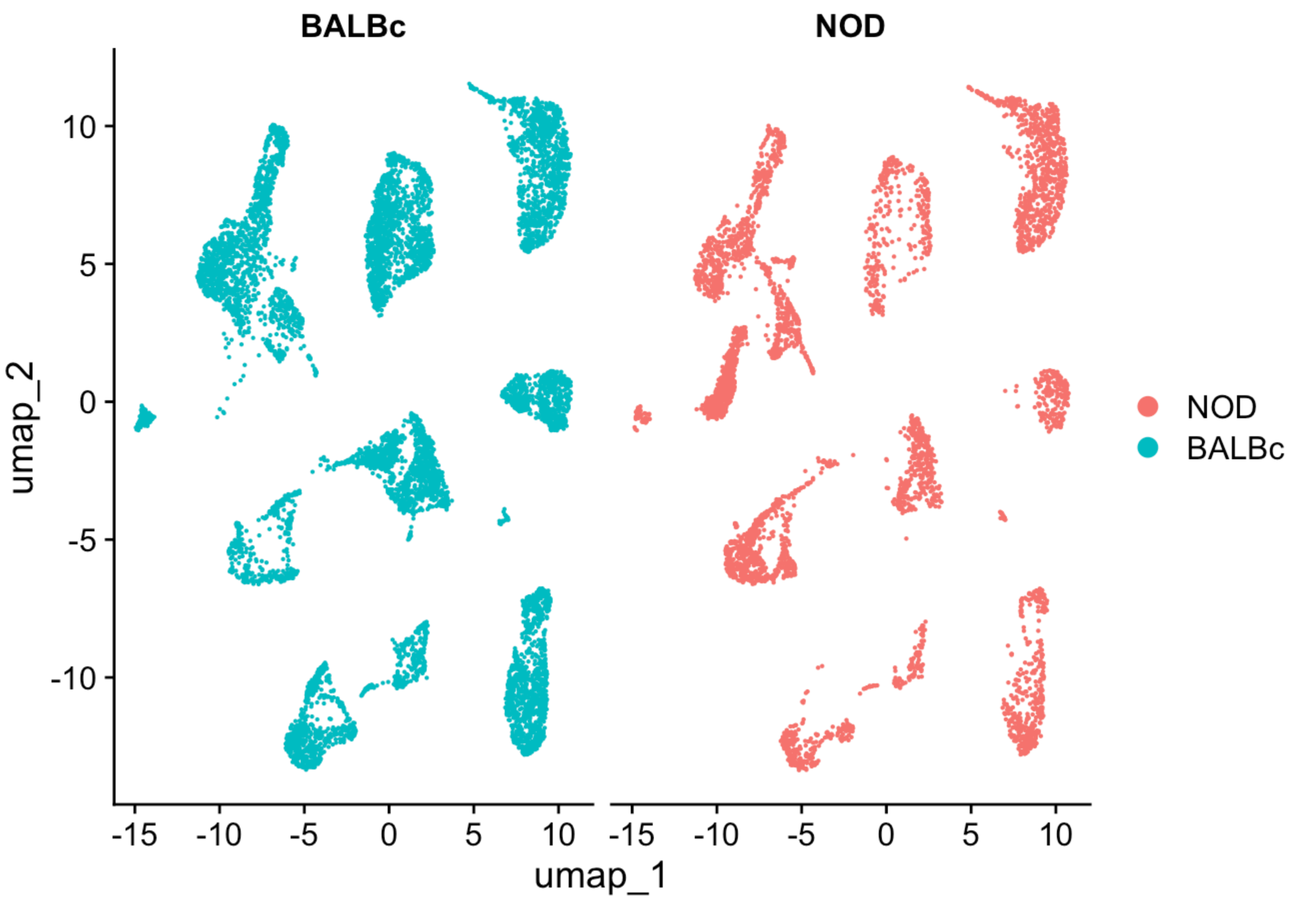


```
# Save integrated seurat object
setwd("~/Documents/3_Parkinsons_disease/2019/scRNAseq/")
saveRDS(seurat_integrated, "data/integrated_seurat.rds")
```

Marker Identification

```
# Single-cell RNA-seq - clustering

# Load libraries
library(Seurat)
library(tidyverse)
library(RCurl)
library(cowplot)
library(multtest)
library(metap)

setwd("~/Documents/3_Parkinsons_disease/2019/scRNAseq/")
seurat_integrated=readRDS("results/integrated_seurat.rds")
```

```
# Explore heatmap of PCs
DimHeatmap(seurat_integrated,
           dims = 1:10,
           cells = 1000,
           balanced = TRUE)
```

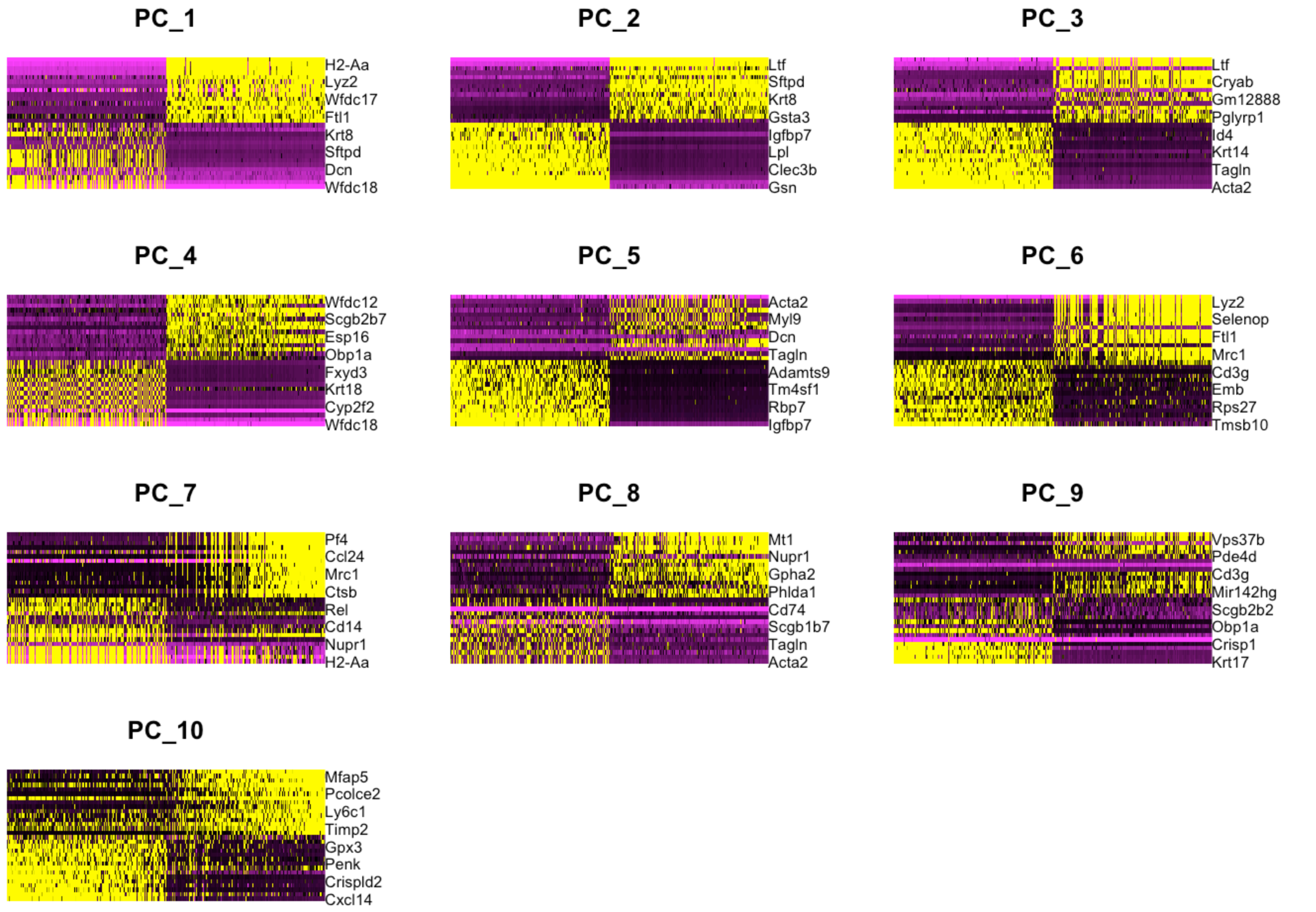


```
# Printing out the most variable genes driving PCs
print(x = seurat_integrated[["pca"]],
      dims = 1:17,
      nfeatures = 5)
```

```
## PC_ 1
## Positive:  Wfdc18, Ltf, Nupr1, Gsn, Dcn
## Negative:  Cd74, H2-Aa, H2-Eb1, H2-Ab1, Cst3
## PC_ 2
## Positive:  Gsn, Dcn, Col3a1, Apod, Clec3b
## Negative:  Wfdc18, Ltf, Nupr1, Krt18, Esp6
## PC_ 3
## Positive:  Acta2, Tpm2, Myl9, Cck, Tagln
## Negative:  Wfdc18, Ltf, Gsn, Cyp2f2, Sftpd
## PC_ 4
## Positive:  Wfdc18, Ltf, Cryab, Apoe, Cyp2f2
## Negative:  Esp15, Wfdc12, Scgb1b7, Pde4d, Gm5570
## PC_ 5
## Positive:  Igfbp7, Flt1, Plvap, Egfl7, Rbp7
## Negative:  Apoe, Acta2, Tpm2, Esp15, Cck
## PC_ 6
## Positive:  Tmsb10, Cd52, Vps37b, Cytip, Rps27
## Negative:  Apoe, Lyz2, Pf4, Ccl8, C1qa
## PC_ 7
## Positive:  H2-Aa, Cd74, H2-Eb1, H2-Ab1, Nupr1
## Negative:  Ccl8, Pf4, Selenop, Cd209g, Lyz2
## PC_ 8
## Positive:  Acta2, Myl9, Esp6, Tpm2, Tagln
## Negative:  Krt17, Mt1, Mt2, Krt14, Crisp1
## PC_ 9
## Positive:  Krt17, Mt2, Krt14, Mt1, Crisp1
## Negative:  Tmsb10, Vps37b, Nupr1, Hcst, Emb
## PC_ 10
## Positive:  Cxcl14, Apod, Crabp1, C7, Crispld2
## Negative:  Pi16, Mfap5, Fn1, Igfbp6, Glp2r
## PC_ 11
## Positive:  Cck, Apod, Apoe, Dcn, Nrg1
## Negative:  Rgs5, Gm13889, Crip1, Ndufa4l2, Sncg
## PC_ 12
## Positive:  C1qa, C1qb, C1qc, Apoe, Rgs1
## Negative:  Wfdc17, Cd209a, Mgl2, Lyz2, H2-Ab1
## PC_ 13
## Positive:  Obp1a, Esp6, Scgb1b7, 2310057J18Rik, Mucl2
## Negative:  Igkc, Igha, Jchain, Iglc2, Slpi
## PC_ 14
## Positive:  Pglyrp1, Cyp2a5, Fxyd3, Esp6, Esp4
## Negative:  Nupr1, Ccn1, Atf3, Ier3, Cldn4
## PC_ 15
## Positive:  Mctp1, Selp, Lrg1, Aqp1, Fabp4
## Negative:  Rbp7, Igfbp7, Ly6c1, Gpihbp1, Krt17
## PC_ 16
## Positive:  Bglap3, Mt1, Cxcl1, 2310057J18Rik, Car6
## Negative:  Cd74, H2-Ab1, Apoe, H2-Aa, H2-Eb1
## PC_ 17
## Positive:  Mt1, Mt2, Cxcl2, Cd83, Cxcl1
## Negative:  Nupr1, Lyz2, Lgals3, Bglap3, Tmsb4x
```

```
# Plot the elbow plot
ElbowPlot(object = seurat_integrated,
          ndims = 30)
```

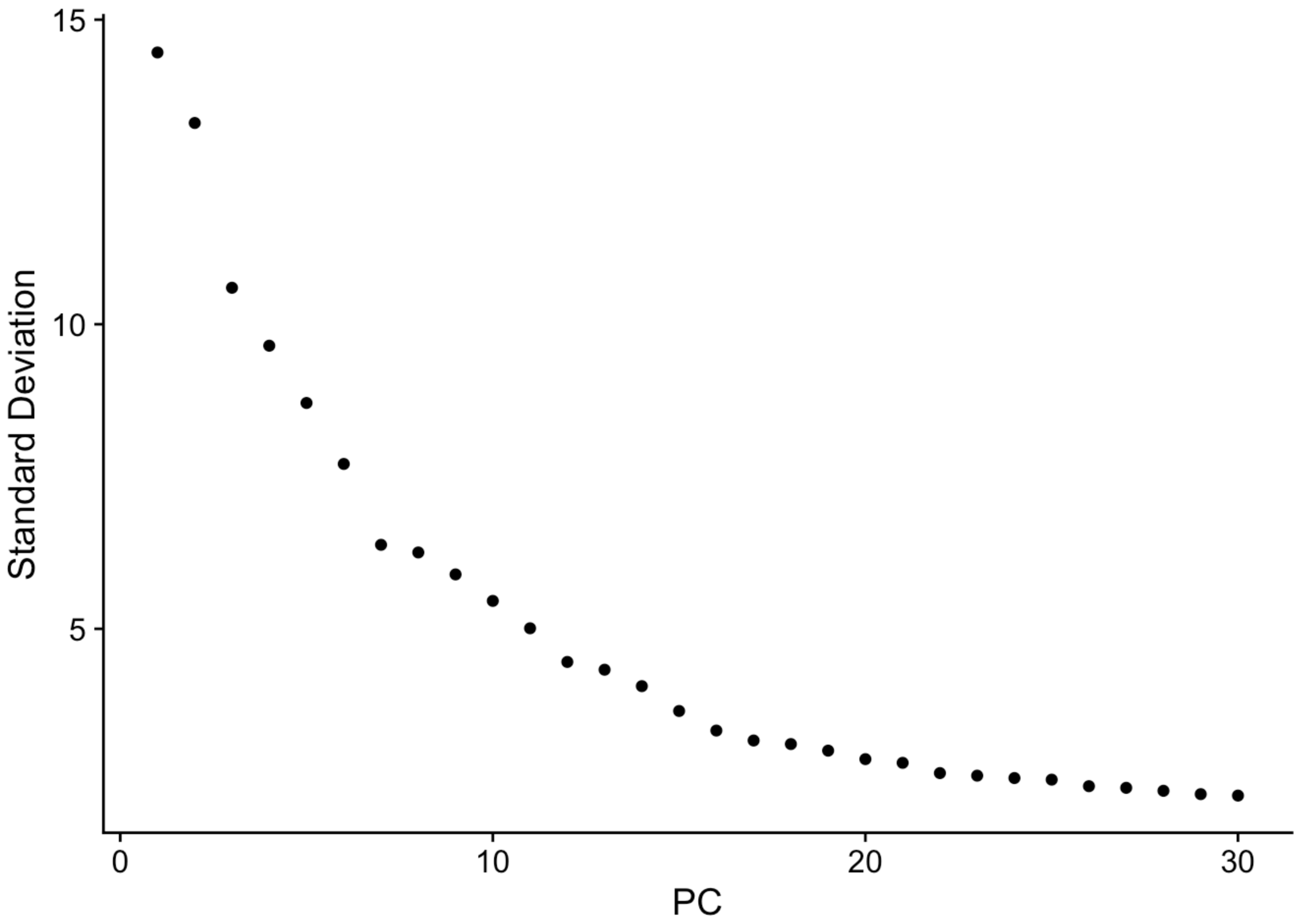


```
# Determine the K-nearest neighbor graph
seurat_integrated <- FindNeighbors(object = seurat_integrated,
                                   dims = 1:40)
```

```
## Computing nearest neighbor graph
```

```
## Computing SNN
```

```
# Determine the clusters for various resolutions
seurat_integrated <- FindClusters(object = seurat_integrated,
                                  resolution = c(0.4, 0.6, 0.8, 1.0, 1.4))
```

```
## Modularity Optimizer version 1.3.0 by Ludo Waltman and Nees Jan van Eck
##
## Number of nodes: 13554
## Number of edges: 496410
##
## Running Louvain algorithm...
## Maximum modularity in 10 random starts: 0.9560
## Number of communities: 17
## Elapsed time: 0 seconds
## Modularity Optimizer version 1.3.0 by Ludo Waltman and Nees Jan van Eck
##
## Number of nodes: 13554
## Number of edges: 496410
##
## Running Louvain algorithm...
## Maximum modularity in 10 random starts: 0.9419
## Number of communities: 20
## Elapsed time: 1 seconds
## Modularity Optimizer version 1.3.0 by Ludo Waltman and Nees Jan van Eck
##
## Number of nodes: 13554
## Number of edges: 496410
##
## Running Louvain algorithm...
## Maximum modularity in 10 random starts: 0.9304
## Number of communities: 23
## Elapsed time: 0 seconds
## Modularity Optimizer version 1.3.0 by Ludo Waltman and Nees Jan van Eck
##
## Number of nodes: 13554
## Number of edges: 496410
##
## Running Louvain algorithm...
## Maximum modularity in 10 random starts: 0.9195
## Number of communities: 25
## Elapsed time: 0 seconds
## Modularity Optimizer version 1.3.0 by Ludo Waltman and Nees Jan van Eck
##
## Number of nodes: 13554
## Number of edges: 496410
##
## Running Louvain algorithm...
## Maximum modularity in 10 random starts: 0.9009
## Number of communities: 31
## Elapsed time: 1 seconds
```

```
# Explore resolutions
seurat_integrated@meta.data %>%
        View()

# Assign identity of clusters
Idents(object = seurat_integrated) <- "integrated_snn_res.0.4"

# Plot the UMAP
DimPlot(seurat_integrated,
        reduction = "umap",
        label = TRUE,
        label.size = 6)
```

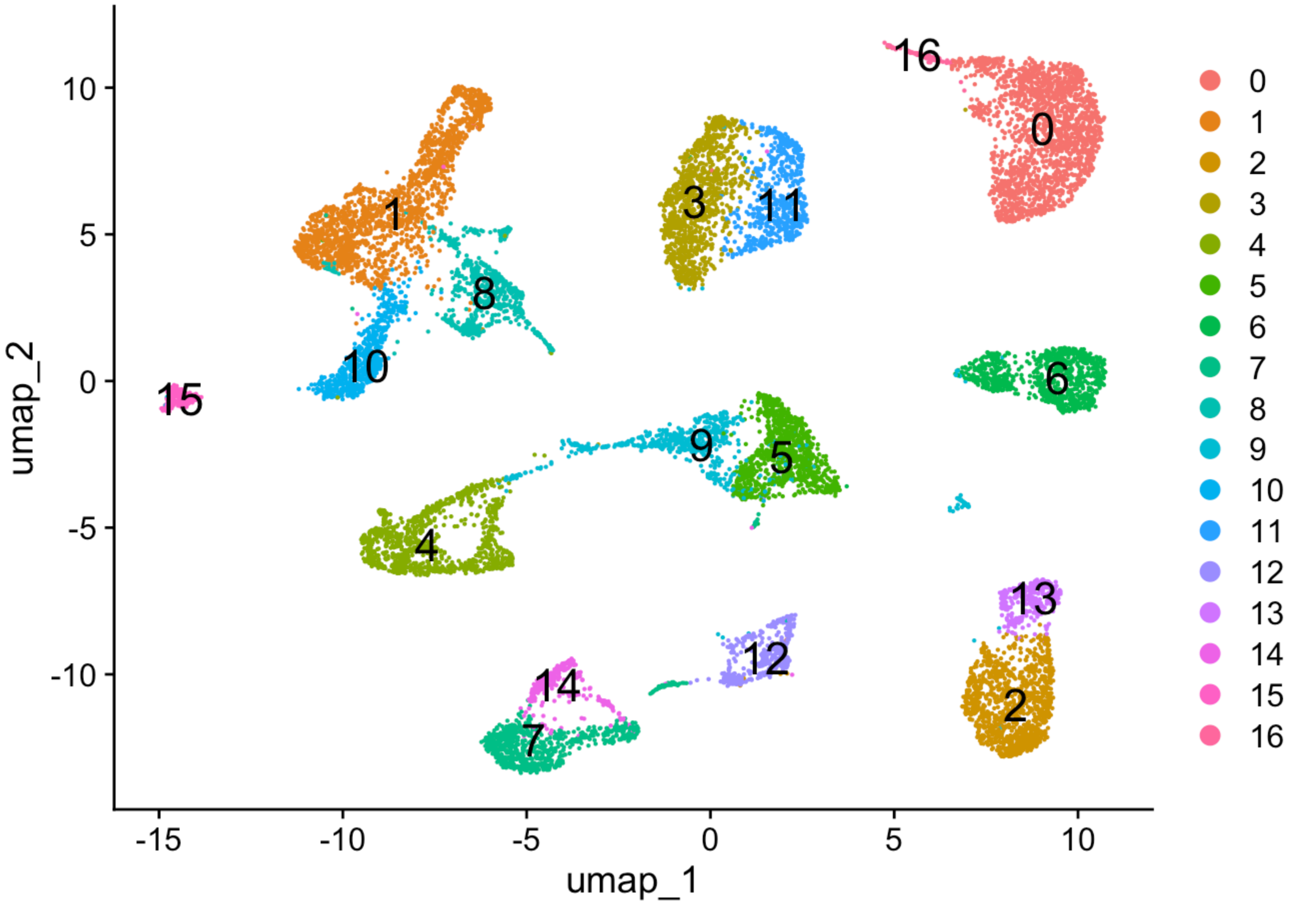

Segregation of clusters by sample

```
# Extract identity and sample information from seurat object to determine the number of
cells per cluster per sample
n_cells <- FetchData(seurat_integrated,
                     vars = c("ident", "sample")) %>%
        dplyr::count(ident, sample)

n_cells$n_edit <- ifelse(n_cells$sample=="NOD", (n_cells$n*2), n_cells$n)

# Barplot of number of cells per cluster by sample
ggplot(n_cells, aes(x=ident, y=n_edit, fill=sample)) +
    geom_bar(position=position_dodge(), stat="identity") +
    geom_text(aes(label=n_edit), vjust = -.2, position=position_dodge(1))
```

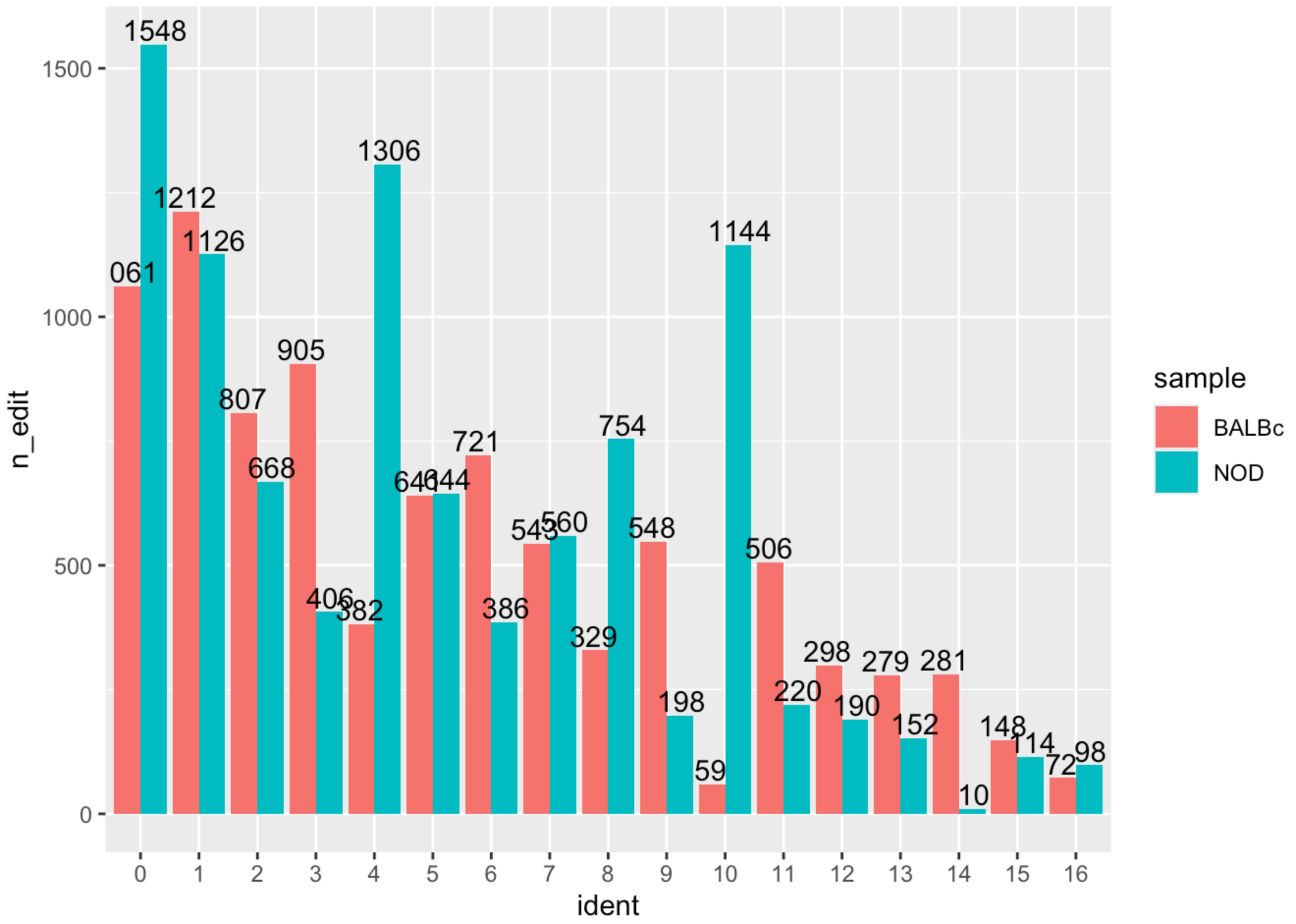


```
# UMAP of cells in each cluster by sample
DimPlot(seurat_integrated,
        label = TRUE,
        split.by = "sample")  + NoLegend()
```

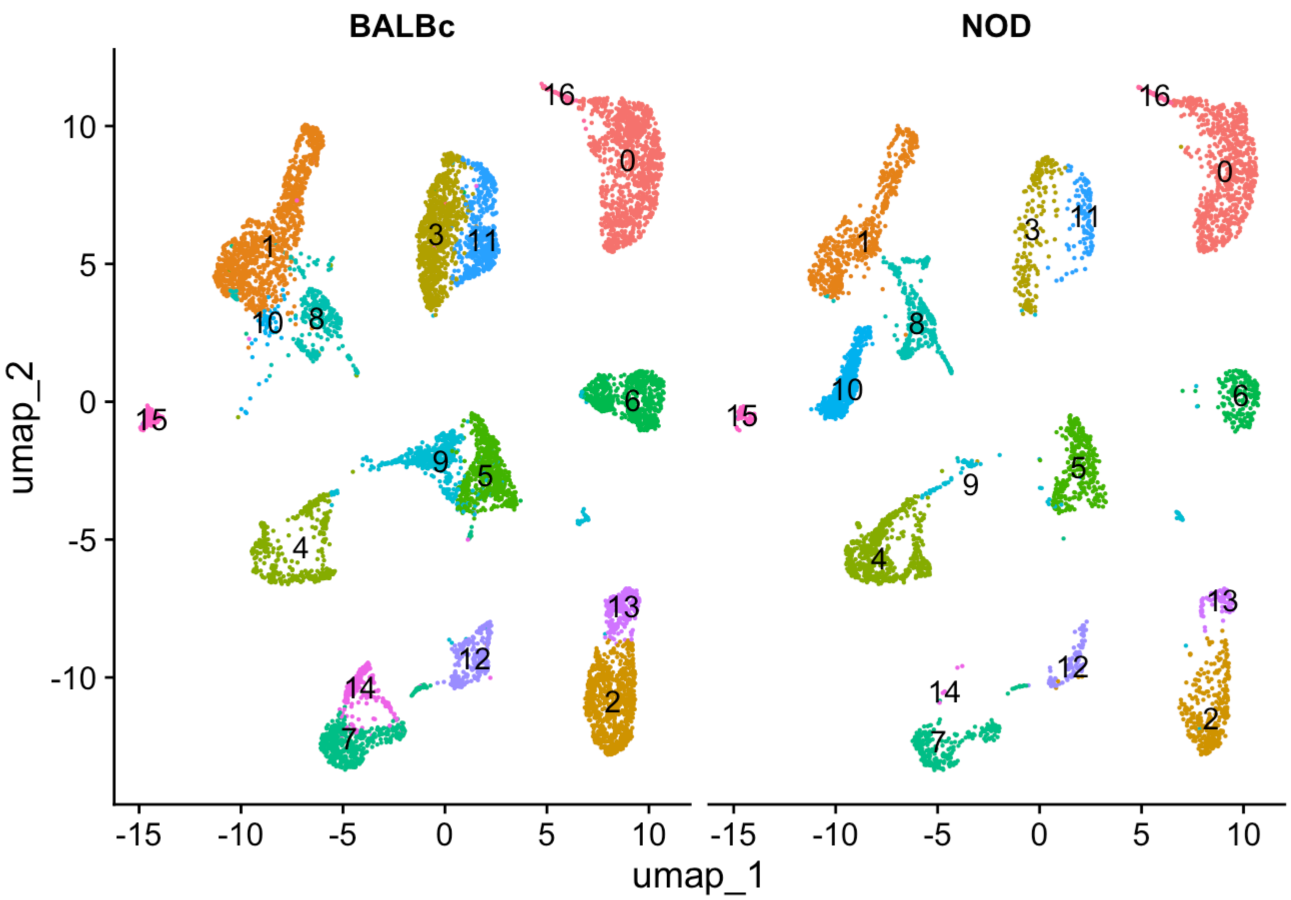

Proportion of cells from a sample in each cluster

```
# Barplot of proportion of cells in each cluster by sample
ggplot(seurat_integrated@meta.data) +
    geom_bar(aes(x=integrated_snn_res.0.4, fill=sample), position=position_fill())
```

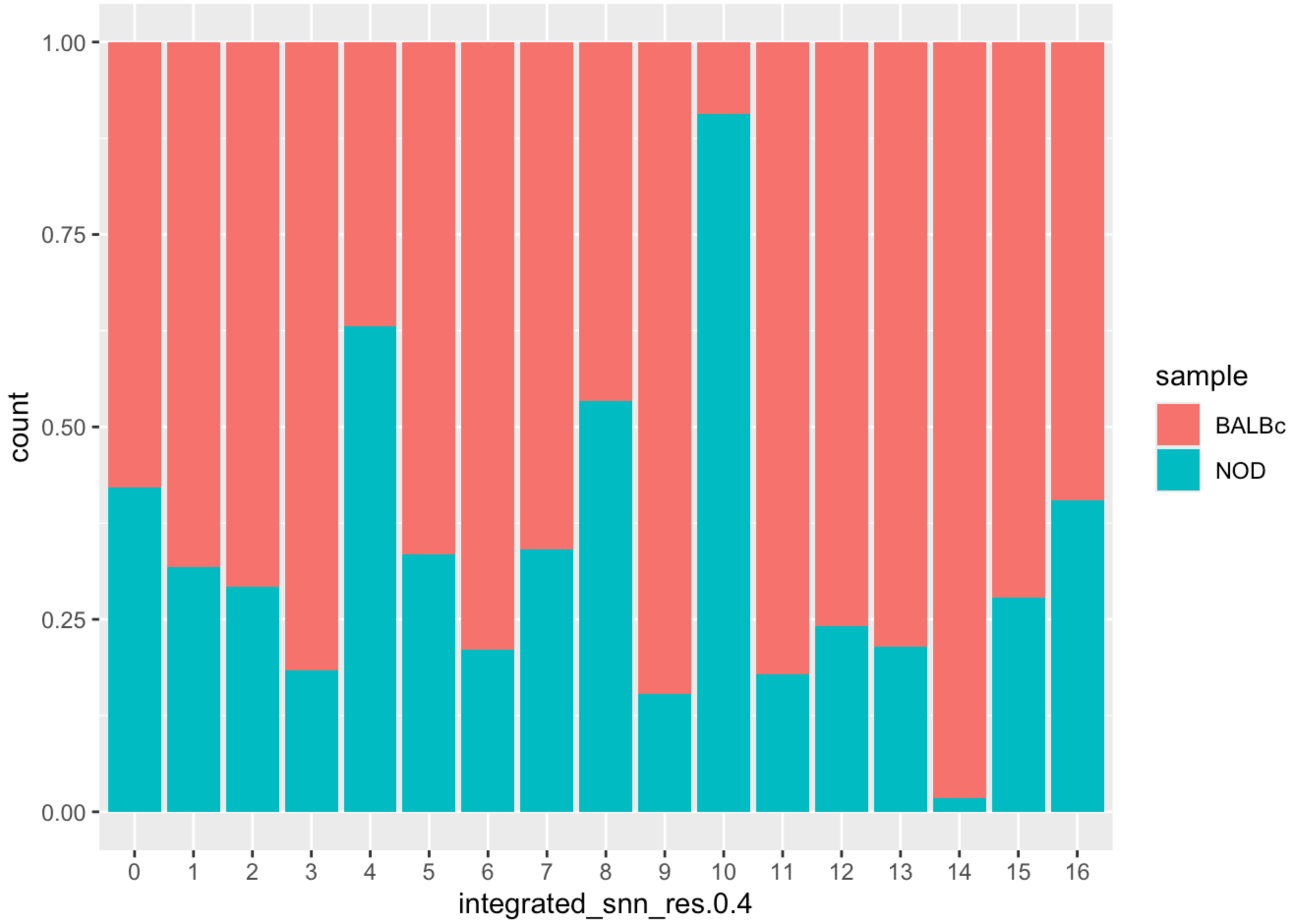


```
# Explore whether clusters segregate by cell cycle phase
DimPlot(seurat_integrated,
        label = TRUE,
        split.by = "Phase")  + NoLegend()
```

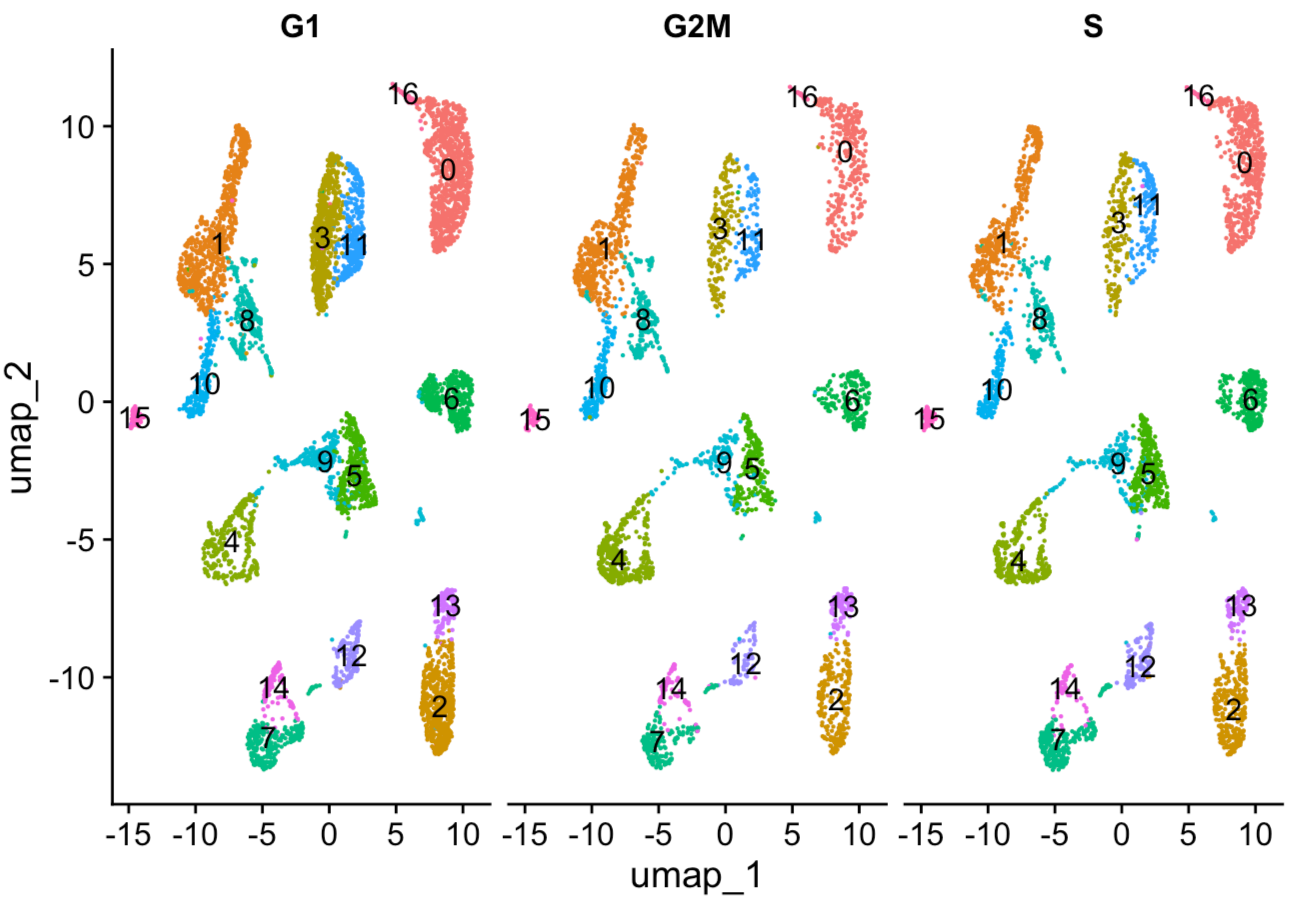

We do not see much clustering by cell cycle score, so we can proceed with the QC.

```
# Determine metrics to plot present in seurat_integrated@meta.data
metrics <-  c("nUMI", "nGene", "S.Score", "G2M.Score", "mitoRatio")

FeaturePlot(seurat_integrated,
            reduction = "umap",
            features = metrics,
            pt.size = 0.4,
            order = TRUE,
            min.cutoff = 'q10',
            label = TRUE)
```

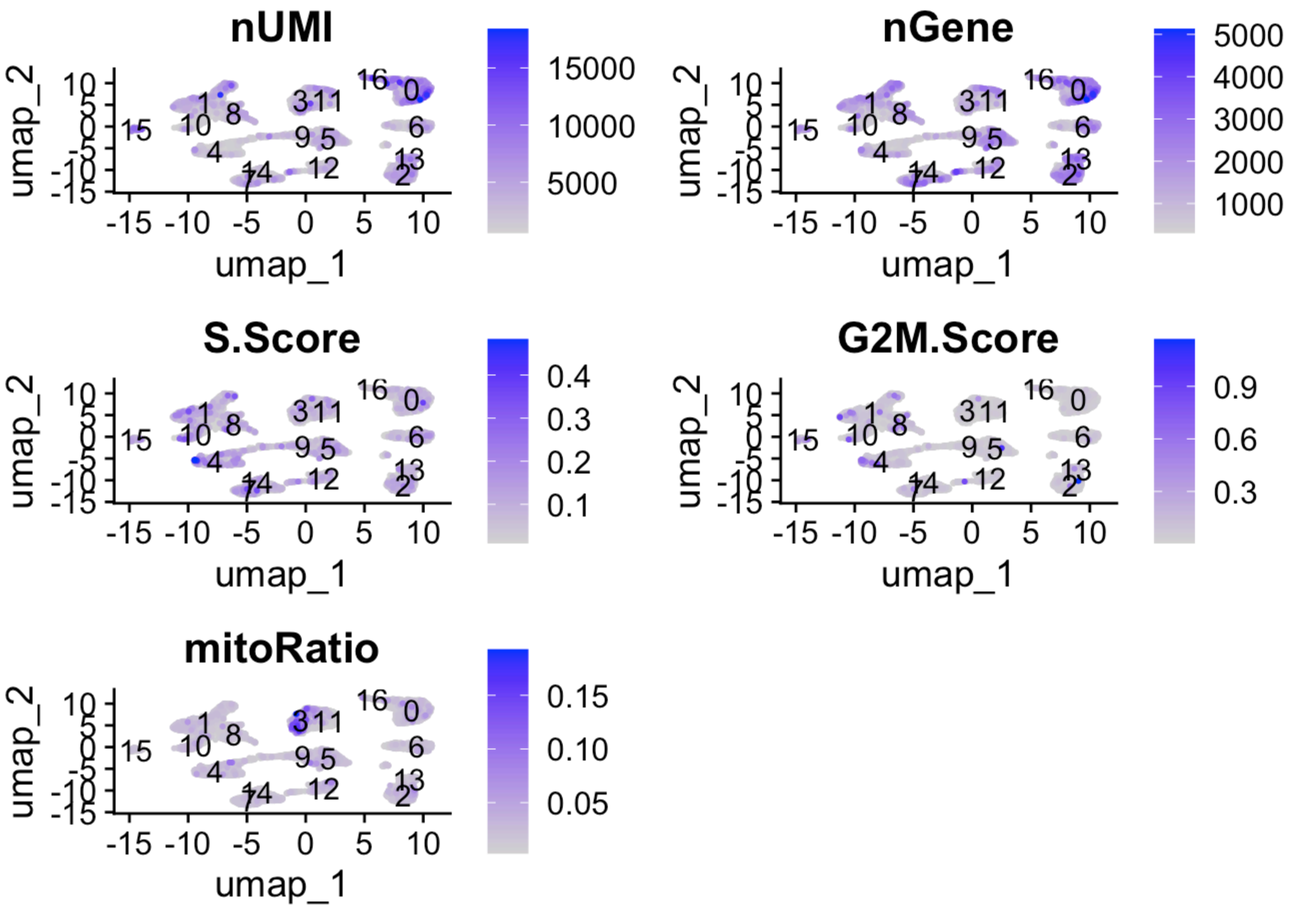

```
# Boxplot of nGene per cluster
ggplot(seurat_integrated@meta.data) +
    geom_boxplot(aes(x=integrated_snn_res.0.4, y=nGene, fill=sample)) +
    NoLegend()
```

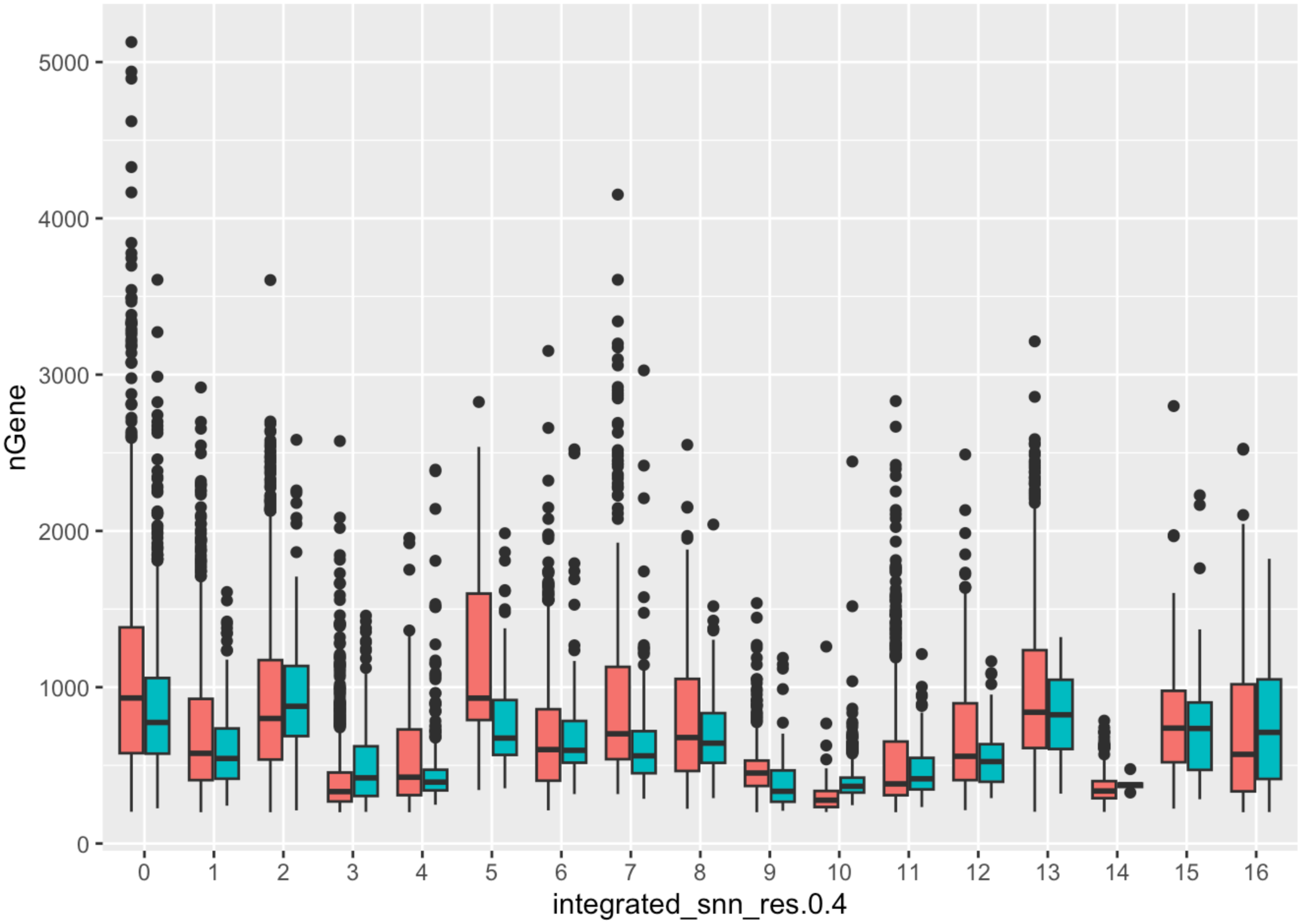

Exploration of the PCs driving the different clusters

```
# Defining the information in the seurat object of interest
columns <- c(paste0("PC_", 1:17),
             "ident",
             "umap_1", "umap_2")

# Extracting this data from the seurat object
pc_data <- FetchData(seurat_integrated,
                     vars = columns)

# Extract the UMAP coordinates for the first 10 cells
seurat_integrated@reductions$umap@cell.embeddings[1:10, 1:2]
```

```
##                                  umap_1     umap_2
## NOD_NOD_I_AAACCTGAGACTGGGT-1  -6.015708  -5.693978
## NOD_NOD_I_AAACCTGAGAGCTTCT-1   2.147268  -8.356813
## NOD_NOD_I_AAACCTGAGCACCGCT-1 -10.497005   5.863803
## NOD_NOD_I_AAACCTGGTCTCGTTC-1   1.038229  -3.490349
## NOD_NOD_I_AAACCTGGTTGGACCC-1   2.075727  -3.166387
## NOD_NOD_I_AAACCTGGTTGGAGGT-1   9.410112   6.673950
## NOD_NOD_I_AAACGGGGTCGACTGC-1   7.647453 -12.199286
## NOD_NOD_I_AAACGGGGTGTAACGG-1   9.043525  -7.153637
## NOD_NOD_I_AAACGGGGTTAGATGA-1  -7.086182   7.605258
## NOD_NOD_I_AAACGGGTCACCACCT-1   7.395932  11.013045
```

```
# Adding cluster label to center of cluster on UMAP
umap_label <- FetchData(seurat_integrated,
                        vars = c("ident", "umap_1", "umap_2"))  %>%
  group_by(ident) %>%
  dplyr::summarise(x=mean(umap_1), y=mean(umap_2))

# Plotting a UMAP plot for each of the PCs
map(paste0("PC_", 1:17), function(pc){
        ggplot(pc_data,
               aes(umap_1, umap_2)) +
                geom_point(aes_string(color=pc),
                           alpha = 0.7) +
                scale_color_gradient(guide = "none",
                                     low = "grey90",
                                     high = "blue")  +
                geom_text(data=umap_label,
                          aes(label=ident, x, y)) +
                ggtitle(pc)
}) %>%
        plot_grid(plotlist = .)
```

```
## Warning: `aes_string()` was deprecated in ggplot2 3.0.0.
## ℹ Please use tidy evaluation idioms with `aes()`.
## ℹ See also `vignette("ggplot2-in-packages")` for more information.
## This warning is displayed once every 8 hours.
## Call `lifecycle::last_lifecycle_warnings()` to see where this warning was
## generated.
```

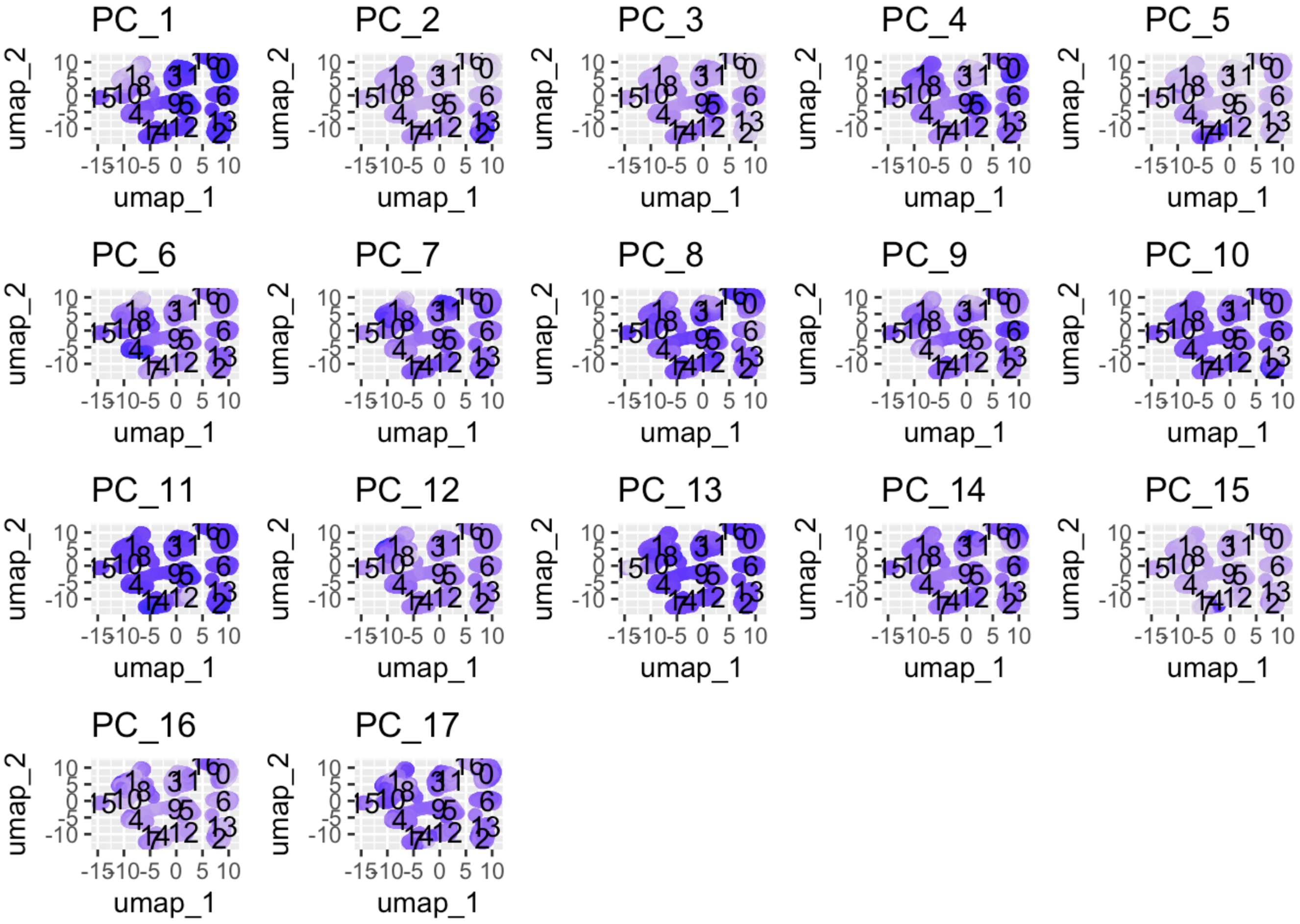


```
# Examine PCA results
print(seurat_integrated[["pca"]], dims = 1:17, nfeatures = 5)
```

```
## PC_ 1
## Positive:  Wfdc18, Ltf, Nupr1, Gsn, Dcn
## Negative:  Cd74, H2-Aa, H2-Eb1, H2-Ab1, Cst3
## PC_ 2
## Positive:  Gsn, Dcn, Col3a1, Apod, Clec3b
## Negative:  Wfdc18, Ltf, Nupr1, Krt18, Esp6
## PC_ 3
## Positive:  Acta2, Tpm2, Myl9, Cck, Tagln
## Negative:  Wfdc18, Ltf, Gsn, Cyp2f2, Sftpd
## PC_ 4
## Positive:  Wfdc18, Ltf, Cryab, Apoe, Cyp2f2
## Negative:  Esp15, Wfdc12, Scgb1b7, Pde4d, Gm5570
## PC_ 5
## Positive:  Igfbp7, Flt1, Plvap, Egfl7, Rbp7
## Negative:  Apoe, Acta2, Tpm2, Esp15, Cck
## PC_ 6
## Positive:  Tmsb10, Cd52, Vps37b, Cytip, Rps27
## Negative:  Apoe, Lyz2, Pf4, Ccl8, C1qa
## PC_ 7
## Positive:  H2-Aa, Cd74, H2-Eb1, H2-Ab1, Nupr1
## Negative:  Ccl8, Pf4, Selenop, Cd209g, Lyz2
## PC_ 8
## Positive:  Acta2, Myl9, Esp6, Tpm2, Tagln
## Negative:  Krt17, Mt1, Mt2, Krt14, Crisp1
## PC_ 9
## Positive:  Krt17, Mt2, Krt14, Mt1, Crisp1
## Negative:  Tmsb10, Vps37b, Nupr1, Hcst, Emb
## PC_ 10
## Positive:  Cxcl14, Apod, Crabp1, C7, Crispld2
## Negative:  Pi16, Mfap5, Fn1, Igfbp6, Glp2r
## PC_ 11
## Positive:  Cck, Apod, Apoe, Dcn, Nrg1
## Negative:  Rgs5, Gm13889, Crip1, Ndufa4l2, Sncg
## PC_ 12
## Positive:  C1qa, C1qb, C1qc, Apoe, Rgs1
## Negative:  Wfdc17, Cd209a, Mgl2, Lyz2, H2-Ab1
## PC_ 13
## Positive:  Obp1a, Esp6, Scgb1b7, 2310057J18Rik, Mucl2
## Negative:  Igkc, Igha, Jchain, Iglc2, Slpi
## PC_ 14
## Positive:  Pglyrp1, Cyp2a5, Fxyd3, Esp6, Esp4
## Negative:  Nupr1, Ccn1, Atf3, Ier3, Cldn4
## PC_ 15
## Positive:  Mctp1, Selp, Lrg1, Aqp1, Fabp4
## Negative:  Rbp7, Igfbp7, Ly6c1, Gpihbp1, Krt17
## PC_ 16
## Positive:  Bglap3, Mt1, Cxcl1, 2310057J18Rik, Car6
## Negative:  Cd74, H2-Ab1, Apoe, H2-Aa, H2-Eb1
## PC_ 17
## Positive:  Mt1, Mt2, Cxcl2, Cd83, Cxcl1
## Negative:  Nupr1, Lyz2, Lgals3, Bglap3, Tmsb4x
```

```
# Select the RNA counts slot to be the default assay
DefaultAssay(seurat_integrated) <- "RNA"

# Normalize RNA data for visualization purposes
seurat_integrated <- NormalizeData(seurat_integrated, verbose = FALSE)
```

```
#CD14+ monocyte markers
setwd("~/Documents/3_Parkinsons_disease/2019/scRNAseq/results/")
tiff(filename = "IgA_IgG_IgM.tiff", height=7, width=8, res=300, units = "in")
FeaturePlot(seurat_integrated,
            reduction = "umap",
            features = c("Igha", "Ighm", "Ighg2b"),
            order = TRUE,
            min.cutoff = 'q5',
            label = TRUE,
            split.by = "sample")
dev.off()
```

```
## quartz_off_screen
##                 2
```

```
# List of known celltype markers

# B cells:  CD79a, Ms4a1
# Plasma Cells: Igkc, Igha, Jchain,
# Mucous/Serous Glandular Cells:  Ltf, Krt18, Cryab, Dmbt1
# Glandular & Luminal Cells: Wfdc18,
# Smooth Muscle Cells: Acta2, Tpm2, Tagln, Igfbp7, Rgs5
# Fibroblast: Igfbp7, Dcn
# Ductal Cells : Krt18
# Macrophages: H2-Eb1, H2-Ab1, H2-Aa, CD74

markers <- list()
markers[["B cells"]] <- c("Cd79a", "Ms4a1")
markers[["T cells"]] <- c("Cd3d", "Cd3e")
markers[["Monocytes"]] <- c("Il1b")
markers[["Plasma cells"]] <- c("Igkc", "Jchain")
markers[["Serous Glandular"]] <- c("Ltf", "Dmbt1")
markers[["Mucous Glandular"]] <- c("Aqp5")
markers[["Endothelial"]] <- c("Aqp1")
markers[["Myoepithelial"]] <- c("Acta2", "Tpm2", "Tagln")
markers[["Macrophages"]] <- c("H2-Eb1", "H2-Ab1", "H2-Aa")
markers[["Ductal Cells"]] <- c("Krt18", "Cxcl17")

# Create dotplot based on RNA expression
DotPlot(seurat_integrated, markers, assay="RNA")
```

```
## Warning: The `facets` argument of `facet_grid()` is deprecated as of ggplot2 2.2.0.
## ℹ Please use the `rows` argument instead.
## ℹ The deprecated feature was likely used in the Seurat package.
##   Please report the issue at <https://github.com/satijalab/seurat/issues>.
## This warning is displayed once every 8 hours.
## Call `lifecycle::last_lifecycle_warnings()` to see where this warning was
## generated.
```

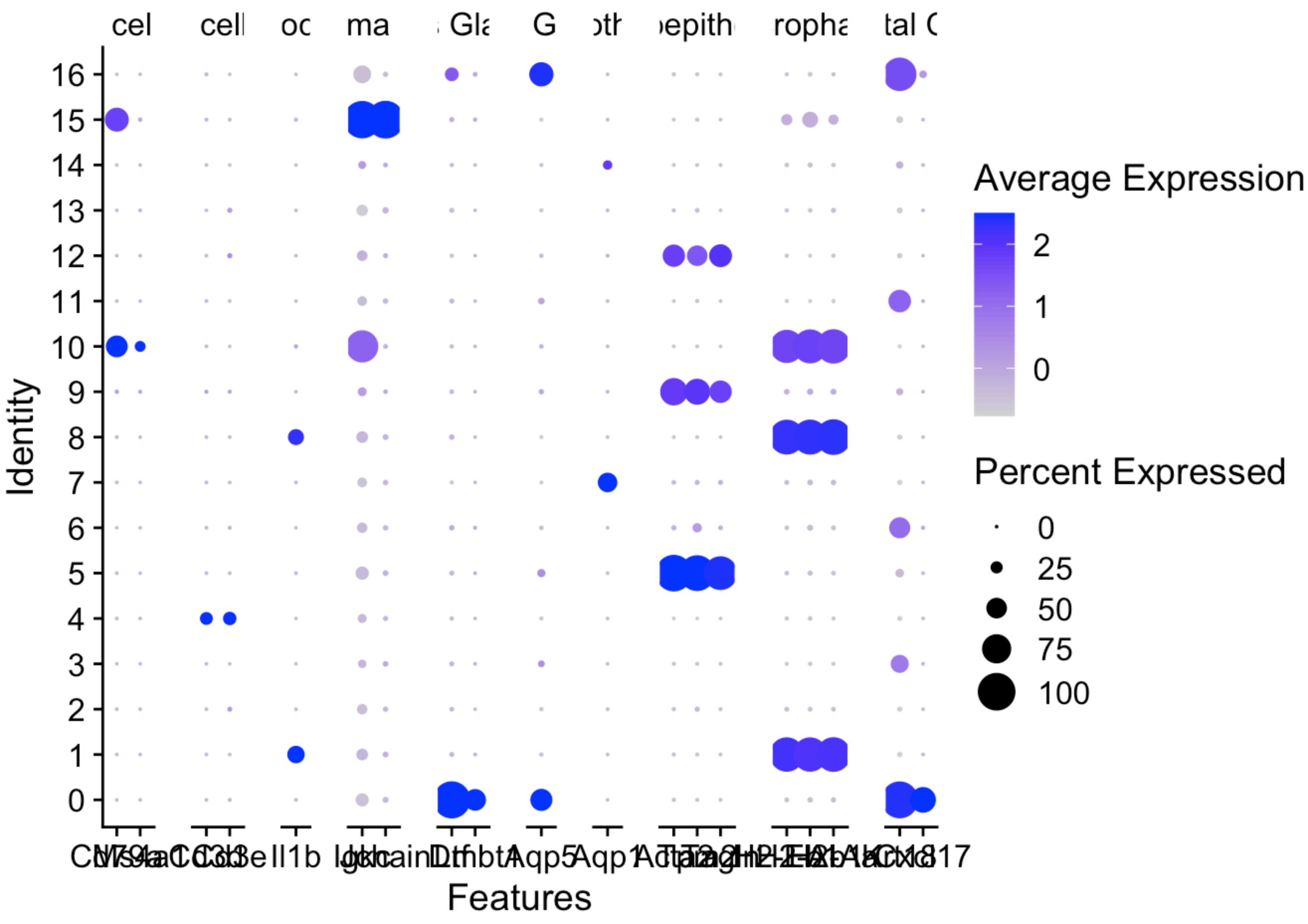


```
cluster10_conserved_markers <- FindConservedMarkers(seurat_integrated,
                              ident.1 = 10,
                              grouping.var = "sample",
                              only.pos = TRUE,
                      logfc.threshold = 0.25)
```

```
## Testing group NOD: (10) vs (4, 12, 1, 5, 9, 0, 2, 13, 15, 8, 11, 6, 7, 3, 16, 14)
```

```
## Testing group BALBc: (10) vs (1, 11, 4, 7, 8, 13, 12, 6, 0, 2, 14, 15, 5, 16, 3, 9)
```

```
#Bank1 is a memory/Naive B-cell marker
```

```
# Connect to AnnotationHub
ah <- AnnotationHub()

# Access the Ensembl database for organism
ahDb <- query(ah,
              pattern = c("Mus musculus", "EnsDb"),
              ignore.case = TRUE)

# Acquire the latest annotation files
id <- ahDb %>%
        mcols() %>%
        rownames() %>%
        tail(n = 1)

# Download the appropriate Ensembldb database
edb <- ah[[id]]
```

```
## loading from cache
```

```
# Extract gene-level information from database
annotations <- genes(edb,
                     return.type = "data.frame")

# Select annotations of interest
annotations <- annotations %>%
        dplyr::select(gene_id, gene_name, seq_name, gene_biotype, description)
```

```
# Combine markers with gene descriptions
cluster10_ann_markers <- cluster10_conserved_markers %>%
                rownames_to_column(var="gene") %>%
                left_join(y = unique(annotations[, c("gene_name", "description")]),
                          by = c("gene" = "gene_name"))

View(cluster10_ann_markers)
#Cluster 14 is Plasma cells

# Iglv1 found in unique cluster of IgG+ plasma cells
#Ref Hollern,D.P.et al. (2019) B cellsand T follicular helper cells mediate response to
checkpoint inhibitors in high mutation burden mouse models of breast cancer. Cell 179, 1
191–1206

#Prg2 Marker of Progenitor Cell

#Ccr10 marker of Plasma cell
```

```
get_conserved <- function(cluster){
  FindConservedMarkers(seurat_integrated,
                       ident.1 = cluster,
                       grouping.var = "sample",
                       only.pos = TRUE) %>%
    rownames_to_column(var = "gene") %>%
    left_join(y = unique(annotations[, c("gene_name", "description")]),
              by = c("gene" = "gene_name")) %>%
    cbind(cluster_id = cluster, .)
  }

# Iterate function across desired clusters
conserved_markers <- map_dfr(c(0,1,2,3,4,5,6,7,8,9,10,11,12,13, 14,15, 16), get_conserve
d)
```

```
## Testing group NOD: (0) vs (4, 12, 1, 5, 9, 2, 13, 15, 8, 11, 6, 7, 3, 10, 16, 14)
```

```
## Testing group BALBc: (0) vs (1, 11, 4, 7, 8, 13, 12, 6, 2, 14, 15, 5, 16, 3, 9, 10)
```

```
## Testing group NOD: (1) vs (4, 12, 5, 9, 0, 2, 13, 15, 8, 11, 6, 7, 3, 10, 16, 14)
```

```
## Testing group BALBc: (1) vs (11, 4, 7, 8, 13, 12, 6, 0, 2, 14, 15, 5, 16, 3, 9, 10)
```

```
## Testing group NOD: (2) vs (4, 12, 1, 5, 9, 0, 13, 15, 8, 11, 6, 7, 3, 10, 16, 14)
```

```
## Testing group BALBc: (2) vs (1, 11, 4, 7, 8, 13, 12, 6, 0, 14, 15, 5, 16, 3, 9, 10)
```

```
## Testing group NOD: (3) vs (4, 12, 1, 5, 9, 0, 2, 13, 15, 8, 11, 6, 7, 10, 16, 14)
```

```
## Testing group BALBc: (3) vs (1, 11, 4, 7, 8, 13, 12, 6, 0, 2, 14, 15, 5, 16, 9, 10)
```

```
## Testing group NOD: (4) vs (12, 1, 5, 9, 0, 2, 13, 15, 8, 11, 6, 7, 3, 10, 16, 14)
```

```
## Testing group BALBc: (4) vs (1, 11, 7, 8, 13, 12, 6, 0, 2, 14, 15, 5, 16, 3, 9, 10)
```

```
## Testing group NOD: (5) vs (4, 12, 1, 9, 0, 2, 13, 15, 8, 11, 6, 7, 3, 10, 16, 14)
```

```
## Testing group BALBc: (5) vs (1, 11, 4, 7, 8, 13, 12, 6, 0, 2, 14, 15, 16, 3, 9, 10)
```

```
## Testing group NOD: (6) vs (4, 12, 1, 5, 9, 0, 2, 13, 15, 8, 11, 7, 3, 10, 16, 14)
```

```
## Testing group BALBc: (6) vs (1, 11, 4, 7, 8, 13, 12, 0, 2, 14, 15, 5, 16, 3, 9, 10)
```

```
## Testing group NOD: (7) vs (4, 12, 1, 5, 9, 0, 2, 13, 15, 8, 11, 6, 3, 10, 16, 14)
```

```
## Testing group BALBc: (7) vs (1, 11, 4, 8, 13, 12, 6, 0, 2, 14, 15, 5, 16, 3, 9, 10)
```

```
## Testing group NOD: (8) vs (4, 12, 1, 5, 9, 0, 2, 13, 15, 11, 6, 7, 3, 10, 16, 14)
```

```
## Testing group BALBc: (8) vs (1, 11, 4, 7, 13, 12, 6, 0, 2, 14, 15, 5, 16, 3, 9, 10)
```

```
## Testing group NOD: (9) vs (4, 12, 1, 5, 0, 2, 13, 15, 8, 11, 6, 7, 3, 10, 16, 14)
```

```
## Testing group BALBc: (9) vs (1, 11, 4, 7, 8, 13, 12, 6, 0, 2, 14, 15, 5, 16, 3, 10)
```

```
## Testing group NOD: (10) vs (4, 12, 1, 5, 9, 0, 2, 13, 15, 8, 11, 6, 7, 3, 16, 14)
```

```
## Testing group BALBc: (10) vs (1, 11, 4, 7, 8, 13, 12, 6, 0, 2, 14, 15, 5, 16, 3, 9)
```

```
## Testing group NOD: (11) vs (4, 12, 1, 5, 9, 0, 2, 13, 15, 8, 6, 7, 3, 10, 16, 14)
```

```
## Testing group BALBc: (11) vs (1, 4, 7, 8, 13, 12, 6, 0, 2, 14, 15, 5, 16, 3, 9, 10)
```

```
## Testing group NOD: (12) vs (4, 1, 5, 9, 0, 2, 13, 15, 8, 11, 6, 7, 3, 10, 16, 14)
```

```
## Testing group BALBc: (12) vs (1, 11, 4, 7, 8, 13, 6, 0, 2, 14, 15, 5, 16, 3, 9, 10)
```

```
## Testing group NOD: (13) vs (4, 12, 1, 5, 9, 0, 2, 15, 8, 11, 6, 7, 3, 10, 16, 14)
```

```
## Testing group BALBc: (13) vs (1, 11, 4, 7, 8, 12, 6, 0, 2, 14, 15, 5, 16, 3, 9, 10)
```

```
## Testing group NOD: (14) vs (4, 12, 1, 5, 9, 0, 2, 13, 15, 8, 11, 6, 7, 3, 10, 16)
```

```
## Testing group BALBc: (14) vs (1, 11, 4, 7, 8, 13, 12, 6, 0, 2, 15, 5, 16, 3, 9, 10)
```

```
## Testing group NOD: (15) vs (4, 12, 1, 5, 9, 0, 2, 13, 8, 11, 6, 7, 3, 10, 16, 14)
```

```
## Testing group BALBc: (15) vs (1, 11, 4, 7, 8, 13, 12, 6, 0, 2, 14, 5, 16, 3, 9, 10)
```

```
## Testing group NOD: (16) vs (4, 12, 1, 5, 9, 0, 2, 13, 15, 8, 11, 6, 7, 3, 10, 14)
```

```
## Testing group BALBc: (16) vs (1, 11, 4, 7, 8, 13, 12, 6, 0, 2, 14, 15, 5, 3, 9, 10)
```

Evaluating marker genes

```
# Extract top 10 markers per cluster
top20 <- conserved_markers %>%
  mutate(avg_fc = (BALBc_avg_log2FC + NOD_avg_log2FC) /2) %>%
  group_by(cluster_id) %>%
  top_n(n = 20,
        wt = avg_fc)

# Visualize top 10 markers per cluster
View(top20)
```

Cluster 0 is Glandular epithelial cells/Acinar cells

```
# Vln plot - cluster 0
VlnPlot(object = seurat_integrated,
        features = c("Ltf", "Dmbt1", "Cxcl17", "Slco4c1", "Tac1"),
        split.by = "sample",
        fill.by = "ident")
```

```
## The default behaviour of split.by has changed.
## Separate violin plots are now plotted side-by-side.
## To restore the old behaviour of a single split violin,
## set split.plot = TRUE.
##
## This message will be shown once per session.
```

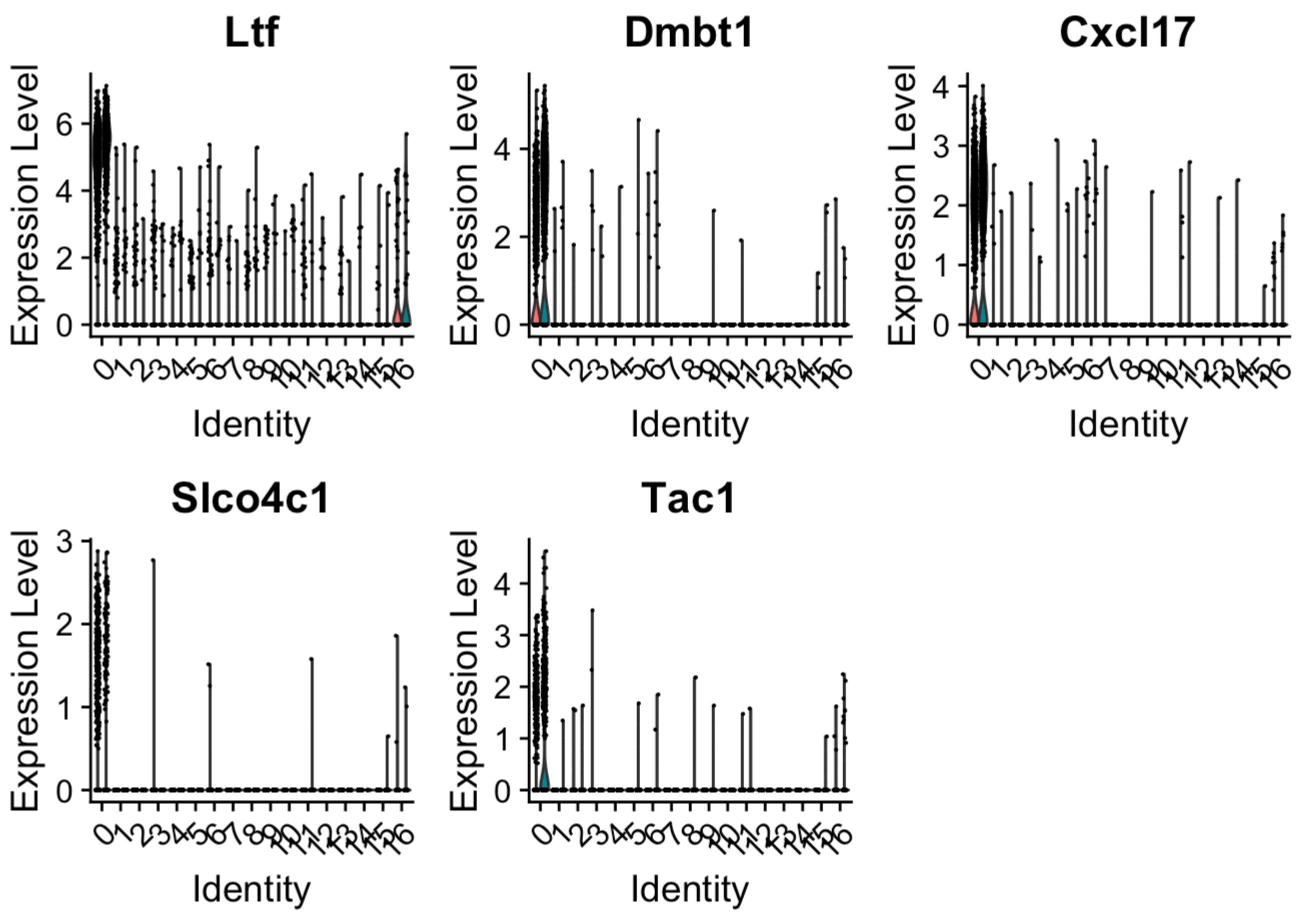


```
VlnPlot(object = seurat_integrated,
        features = c("Ltf", "Ctsl", "Ctss", "Ctsd", "Cst3"),
        split.by = "sample",
        fill.by = "ident")
```

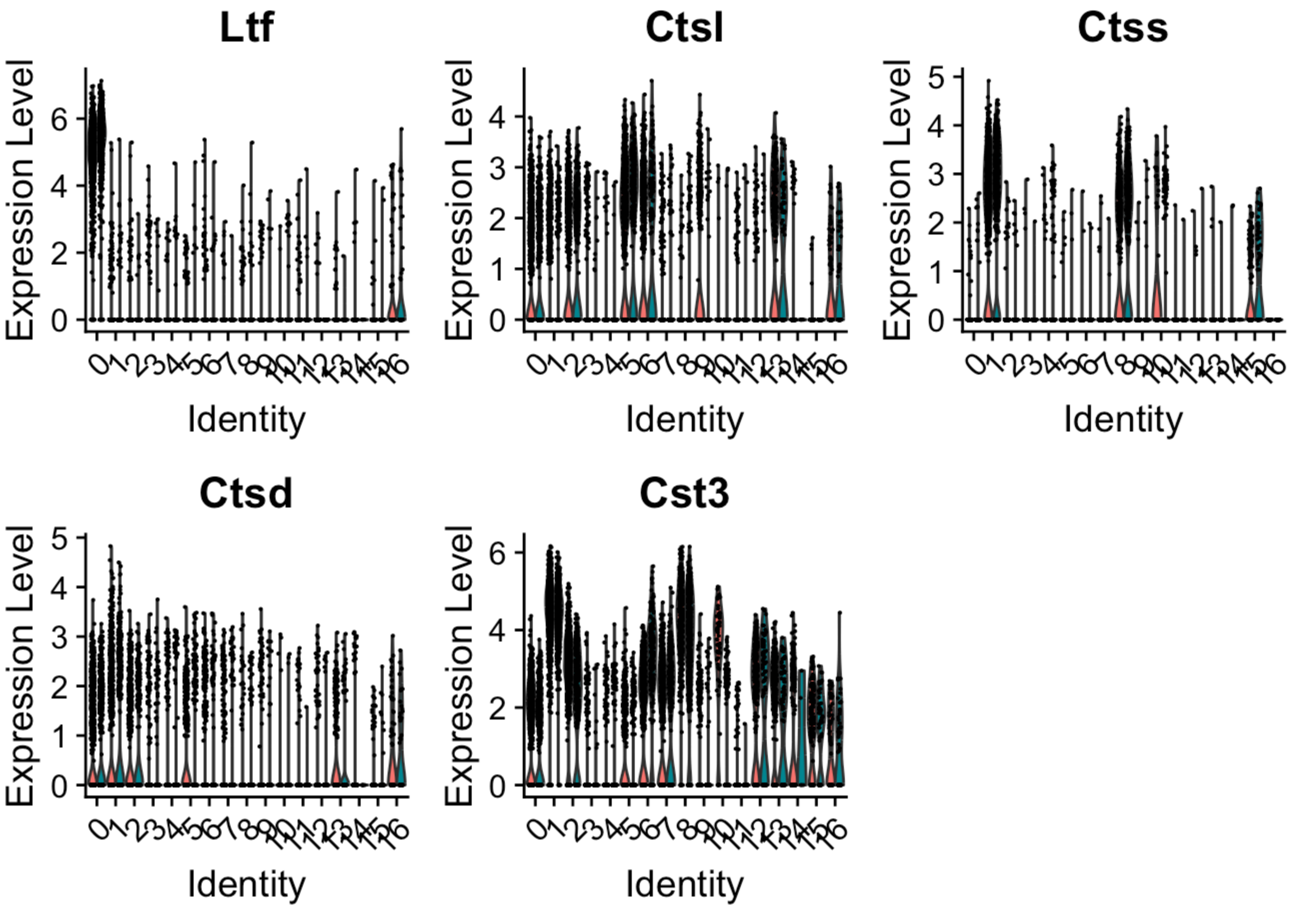


```
VlnPlot(object = seurat_integrated,
        features = c("Ltf", "Ctss"),
        split.by = "sample",
        fill.by = "ident")
```

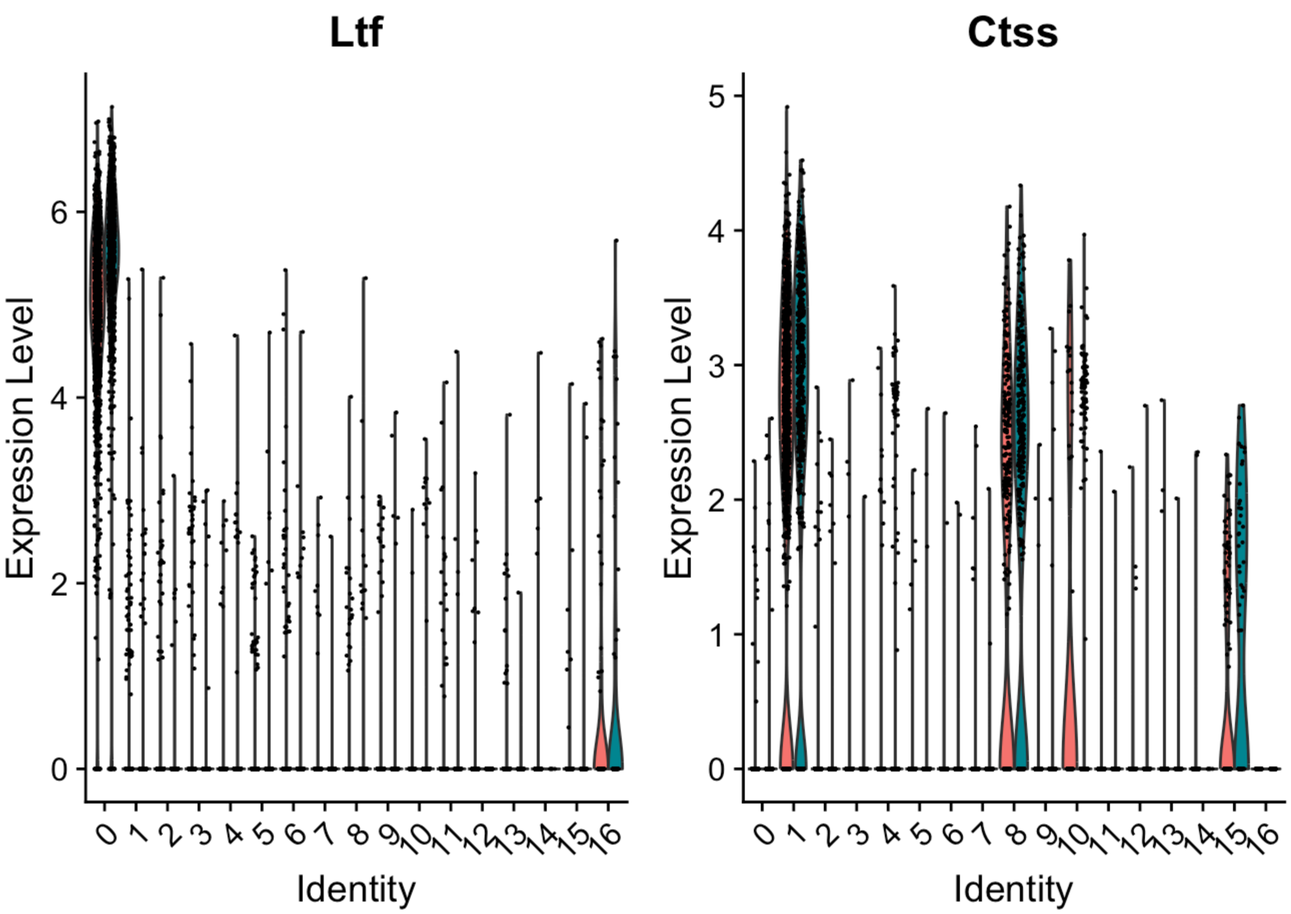


```
#Slco4c1 Transmembrane Transport marker; Secretory cells
#Tac1 Secretory neurons, glandular cells and enteroendocine cells
```

Cluster 1 is Macrophage plasmacytoid DC/ Non-Classical Monocyte Likely Macrophage

```
# Vln plot - cluster 1
VlnPlot(object = seurat_integrated,
        features = c("C1qa", "C1qb", "C1qc", "Ms4a7"))
```

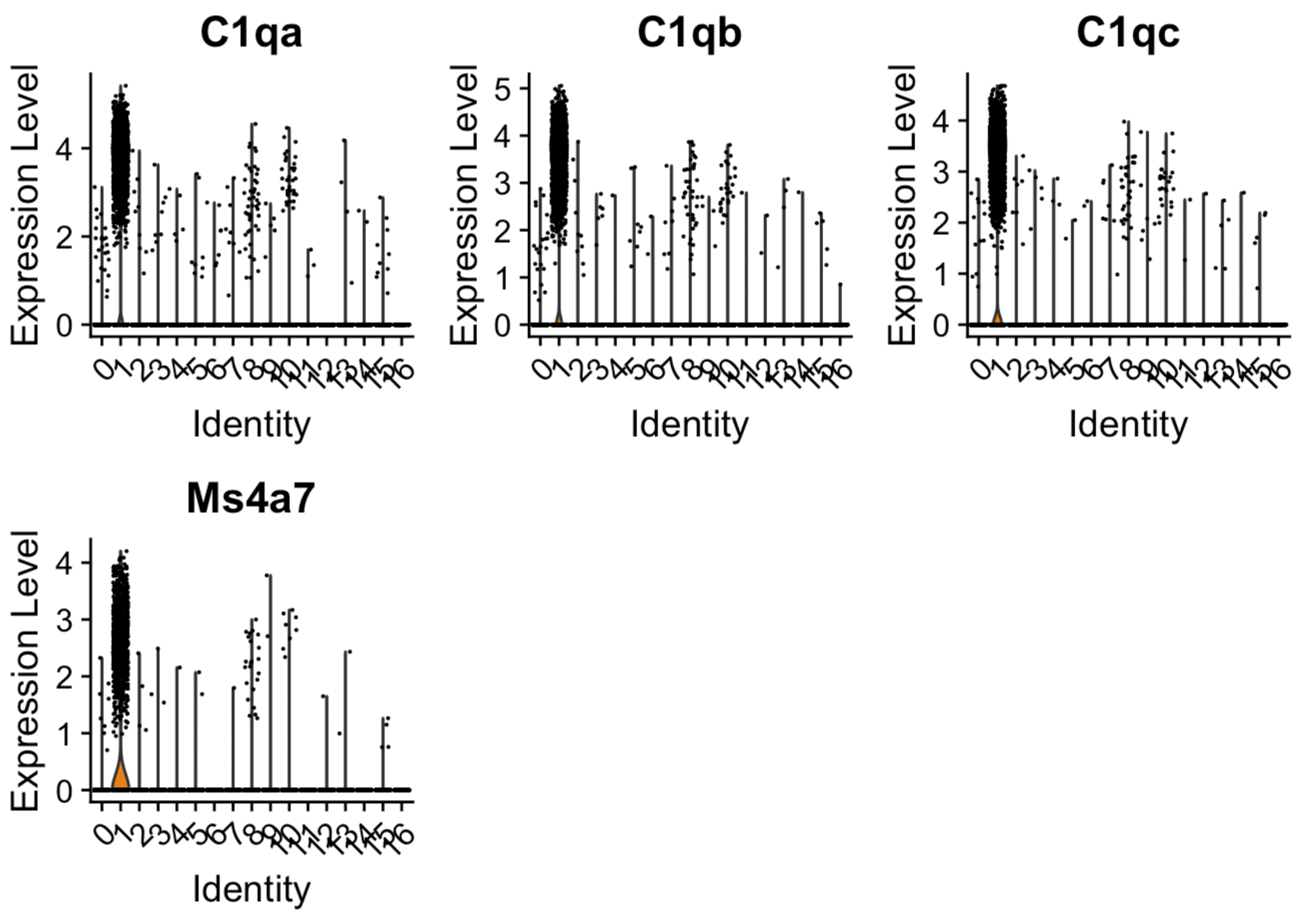


```
VlnPlot(object = seurat_integrated,
        features = c("Cx3cr1", "Dcstamp", "Zmynd15"))
```

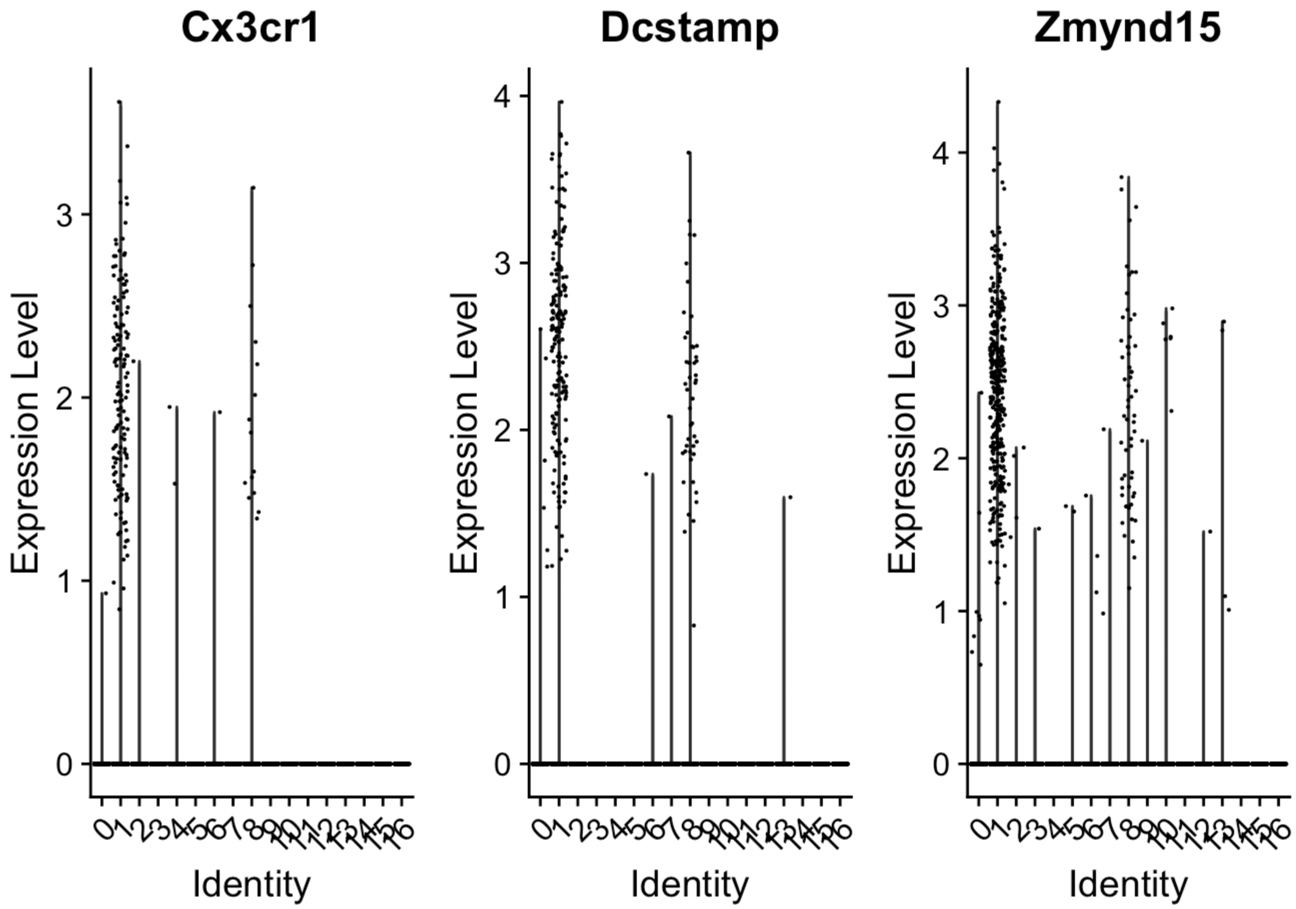


```
#clusters 2 and 9 are different types of Macrophages

#Trem2 is present in cluster 9 also. Found in classical Monocyte, and plasmacytoid DC
#C1qa, C1qc is a macrophage marker (highly selective)
```

Cluster 1 is classical monocyte/F13a1+ve Macrophage Activated Macrophage

```
# Vln plot - cluster 1
VlnPlot(object = seurat_integrated,
        features = c("Pf4", "Ccl8", "F13a1", "Cd209d"))
```

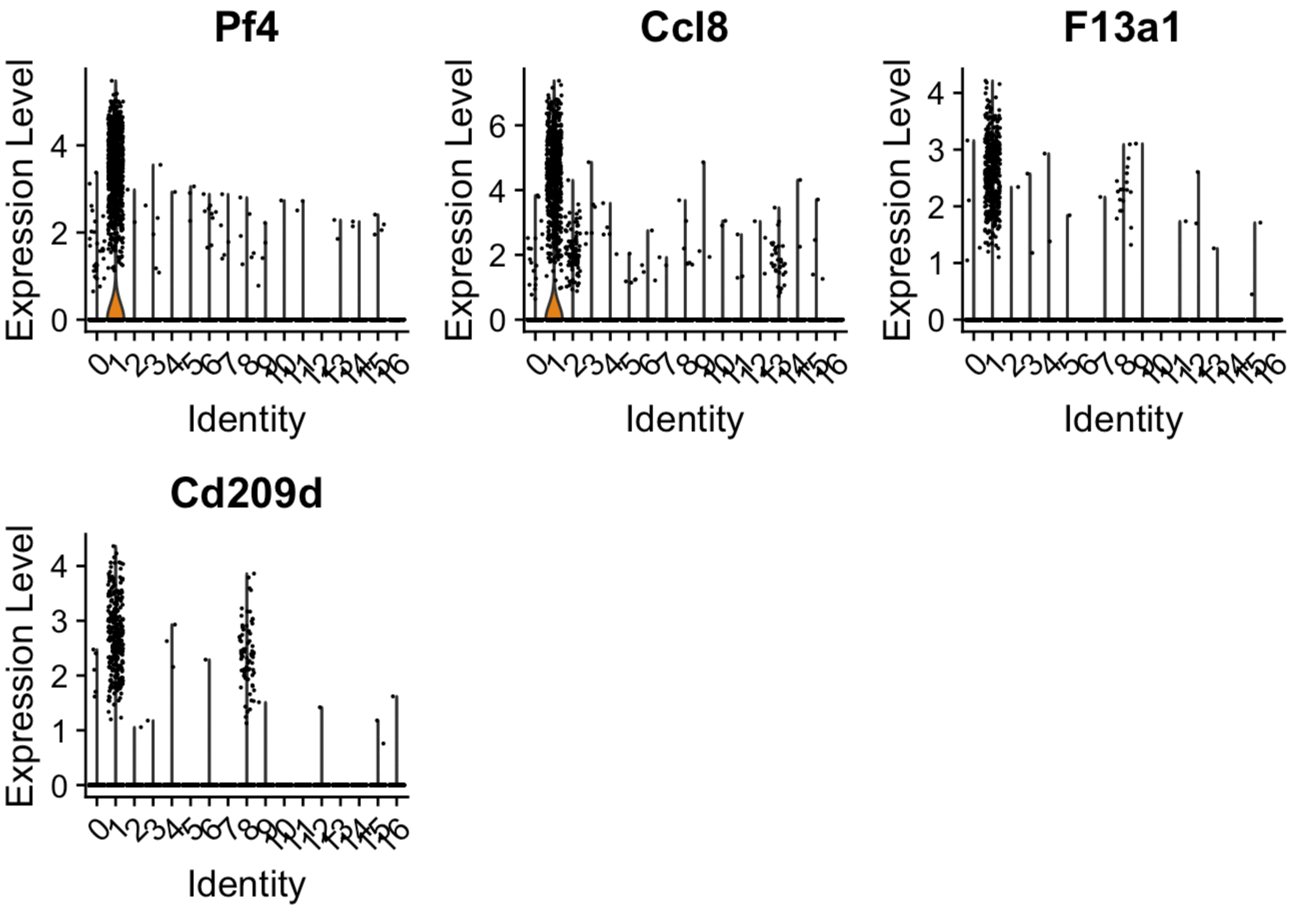

Cluster 2 is Fibroblast (Fibroblast + Smooth Muscle)

```
# Vln plot - cluster 2
VlnPlot(object = seurat_integrated,
        features = c("Cxcl14", "Crabp1", "C7", "Fbln7","Fgf10"))
```

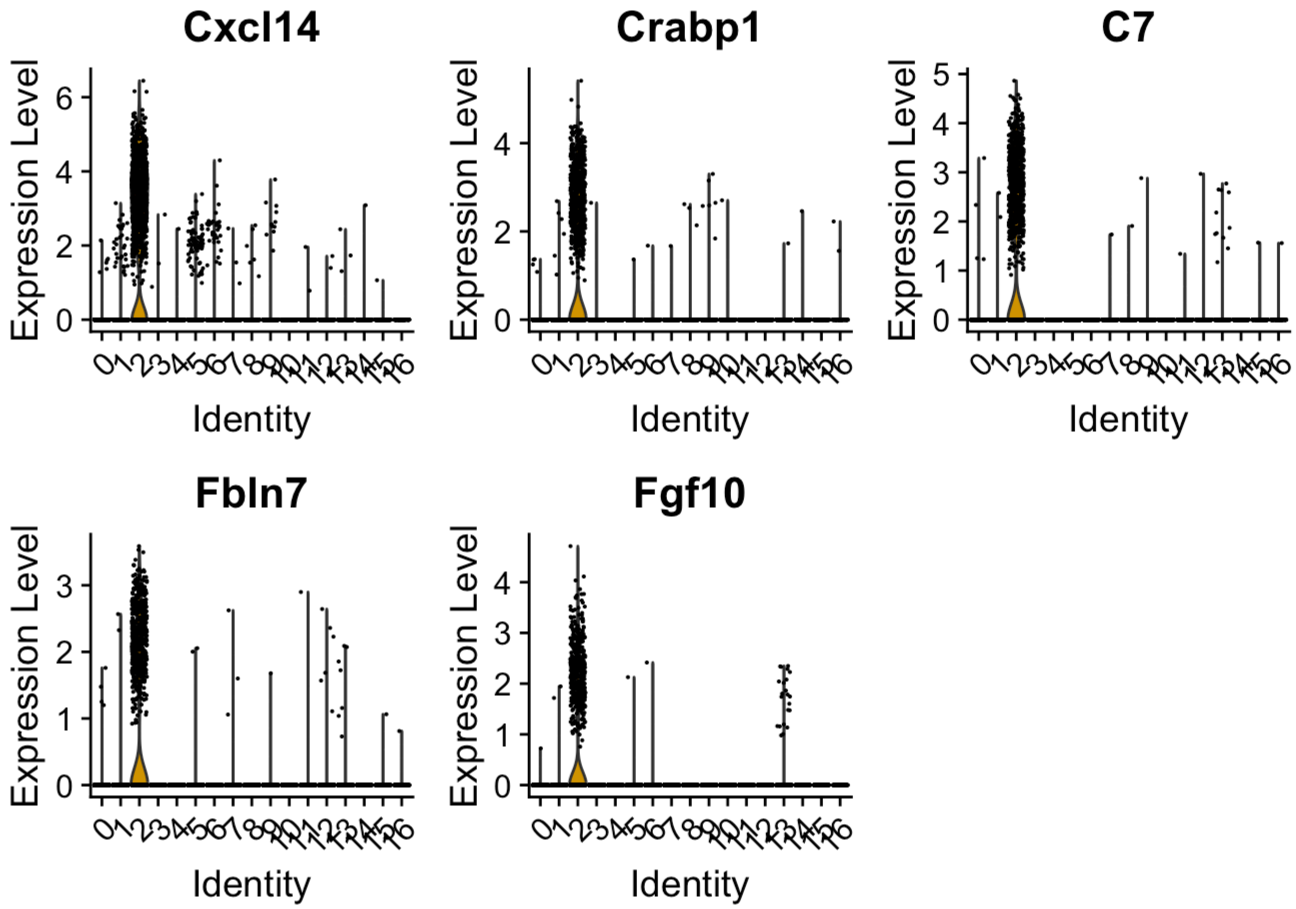


```
#Cxcl14 chemoattractant for Neutrophils, Serous Glandular Epithelium / Myoepithelial cel
ls. Also present in Fibroblasts
#C7 in Fibroblasts, Endothelial,
#Crabp1 in Muller Glial cells
#Fbln7: Fibroblast
#Fgf10: Fibroblast
```

Cluster 3 is Gm12876+ve Serous glandular cells I Gm12876, Gm49815, Gm19724 found upregulated in Rabies Virus strain PB4 infected Salivary glands (parotid glands) Ref: PMID: 38380102

```
# Vln plot - cluster 3

VlnPlot(object = seurat_integrated,
        features = c("Slco1a7", "Erbb4", "Arfgef3", "Tox3"))
```

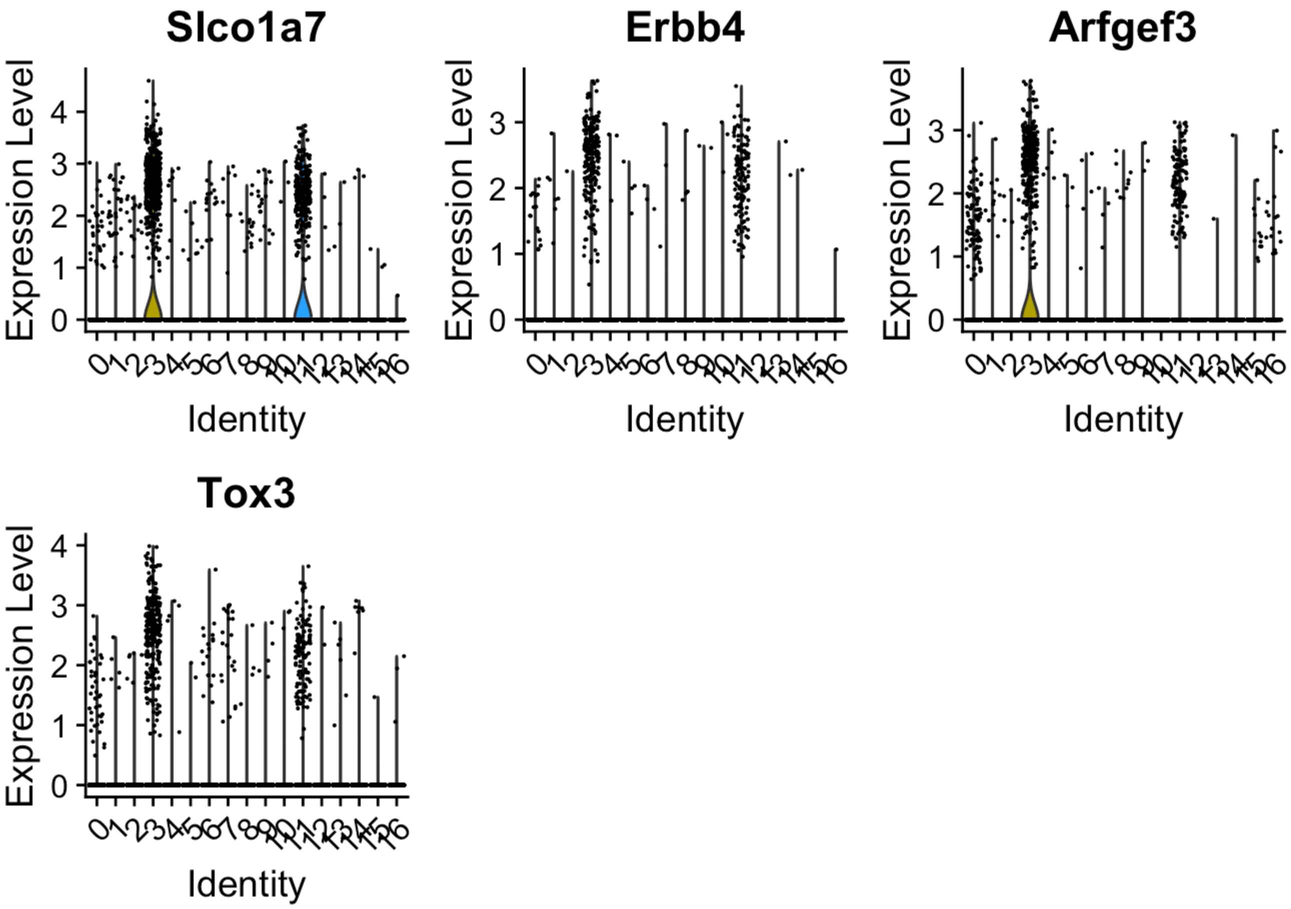


```
VlnPlot(object = seurat_integrated,
        features = c("Gm12876", "Gm49815", "A4gnt", "Extl1", "Slc27a2"))
```

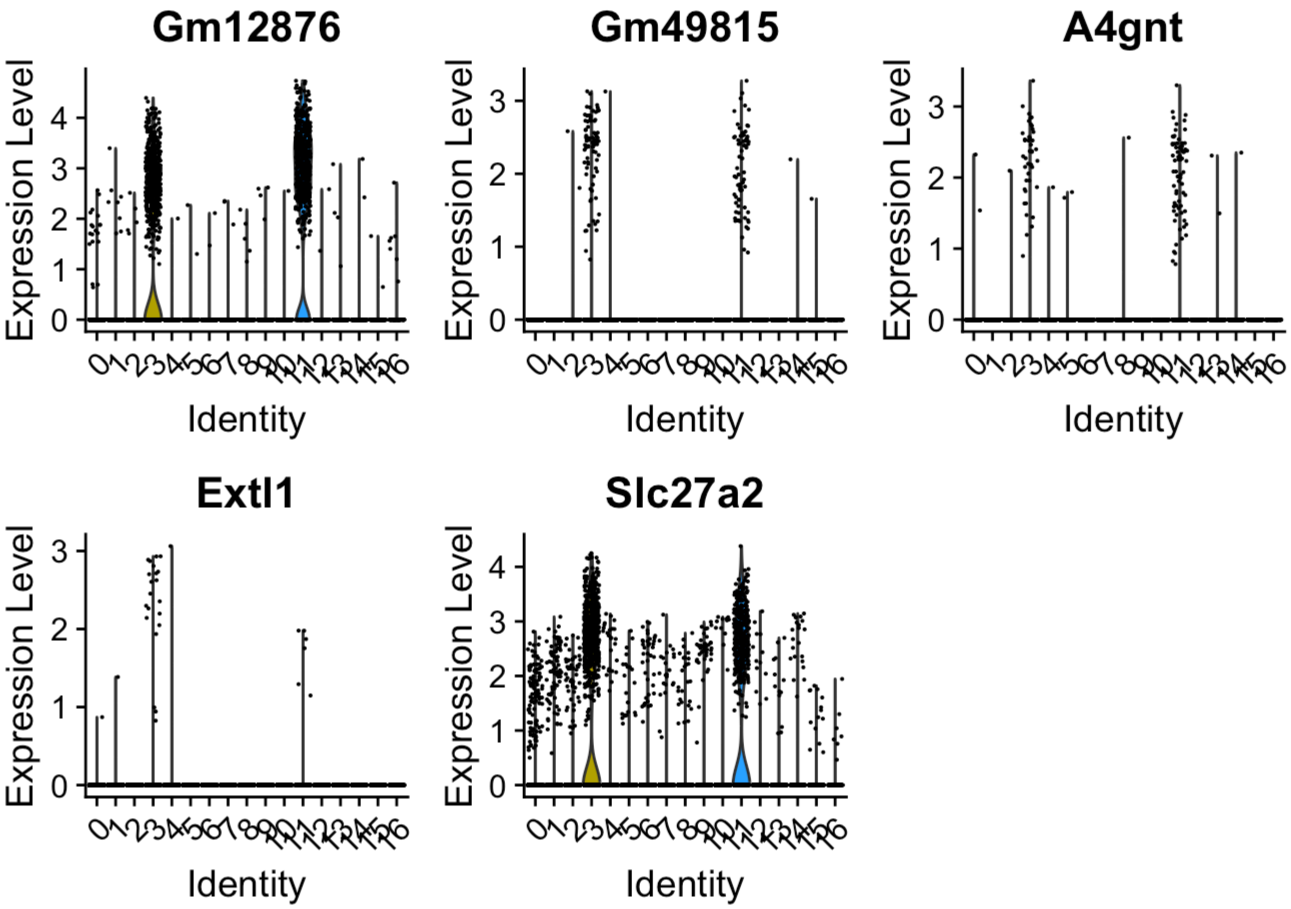


```
#Present in clusters 3 and 11
#Tox3 Breast Glandular Cells, Eccrine sweat glands
#Also a marker of Muller Glial Cells
#Arfgef3 Salivary Serous Glandular cells, also present in salivary smooth muscle cells
```

Cluster 4 is T cell Trbc1 is enriched in NK cells and T-regs Nkg7 is enriched in NK-cells Vps37b is enriched in Nk-cells

```
# Vln plot - cluster 4
VlnPlot(object = seurat_integrated,
        features = c("Cd3g", "Itk", "Trbc2", "Lat", "Nkg7"))
```

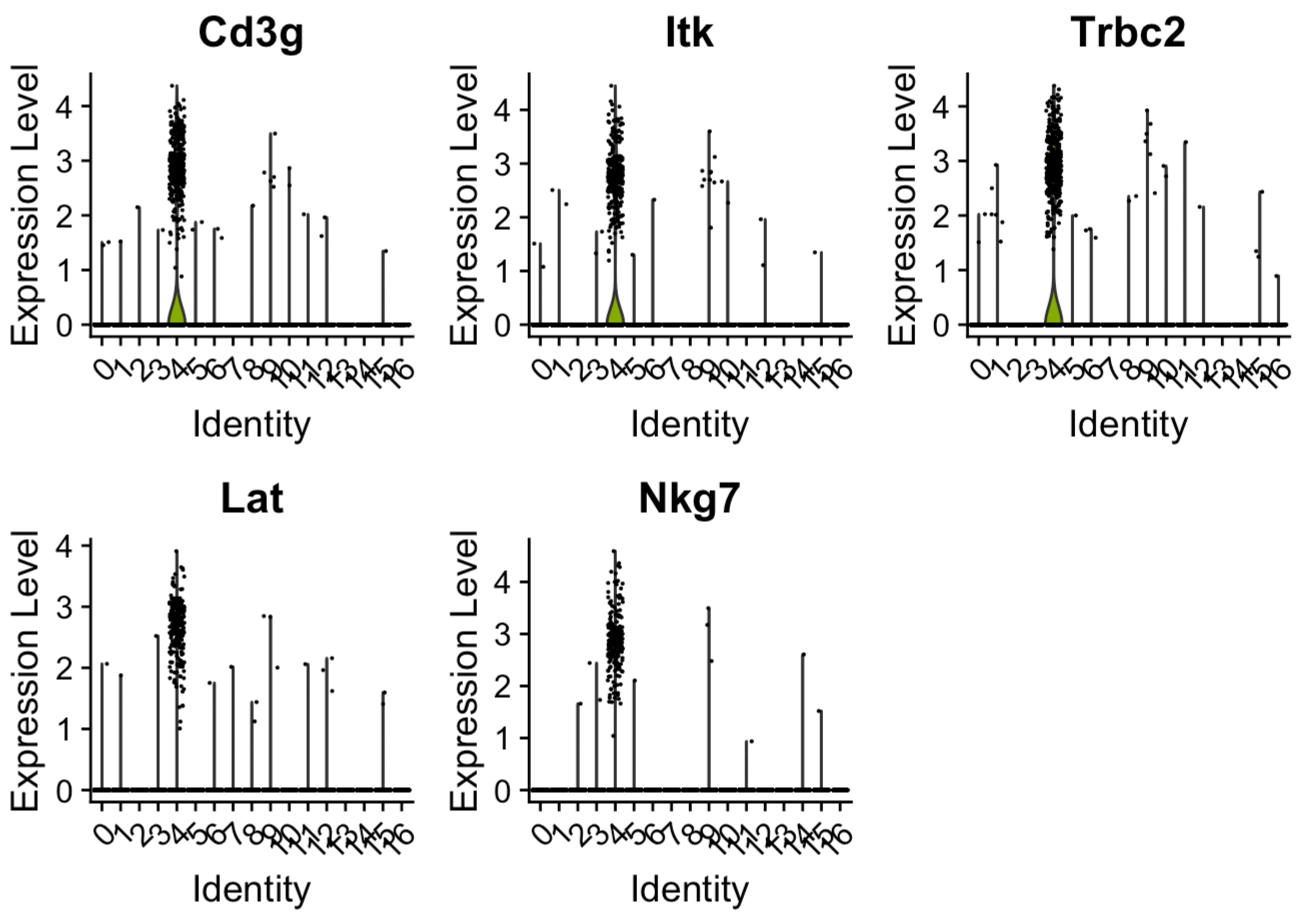


```
#Cd3g: T cells & NK Cells
#Itk: T cells & NK Cells
##Trbc1: T cells & NK Cells
```

Cluster 5 is Bipolar cell (Smooth Muscle/Myoepithelial cells) Collecting Duct Cells/myoepithelial cells & 9 is myoepithelial cells

```
# Vln plot - cluster 5
VlnPlot(object = seurat_integrated,
        features = c("Cck", "Cnn1", "Igfbp2", "Pcp4", "Lama1"))
```

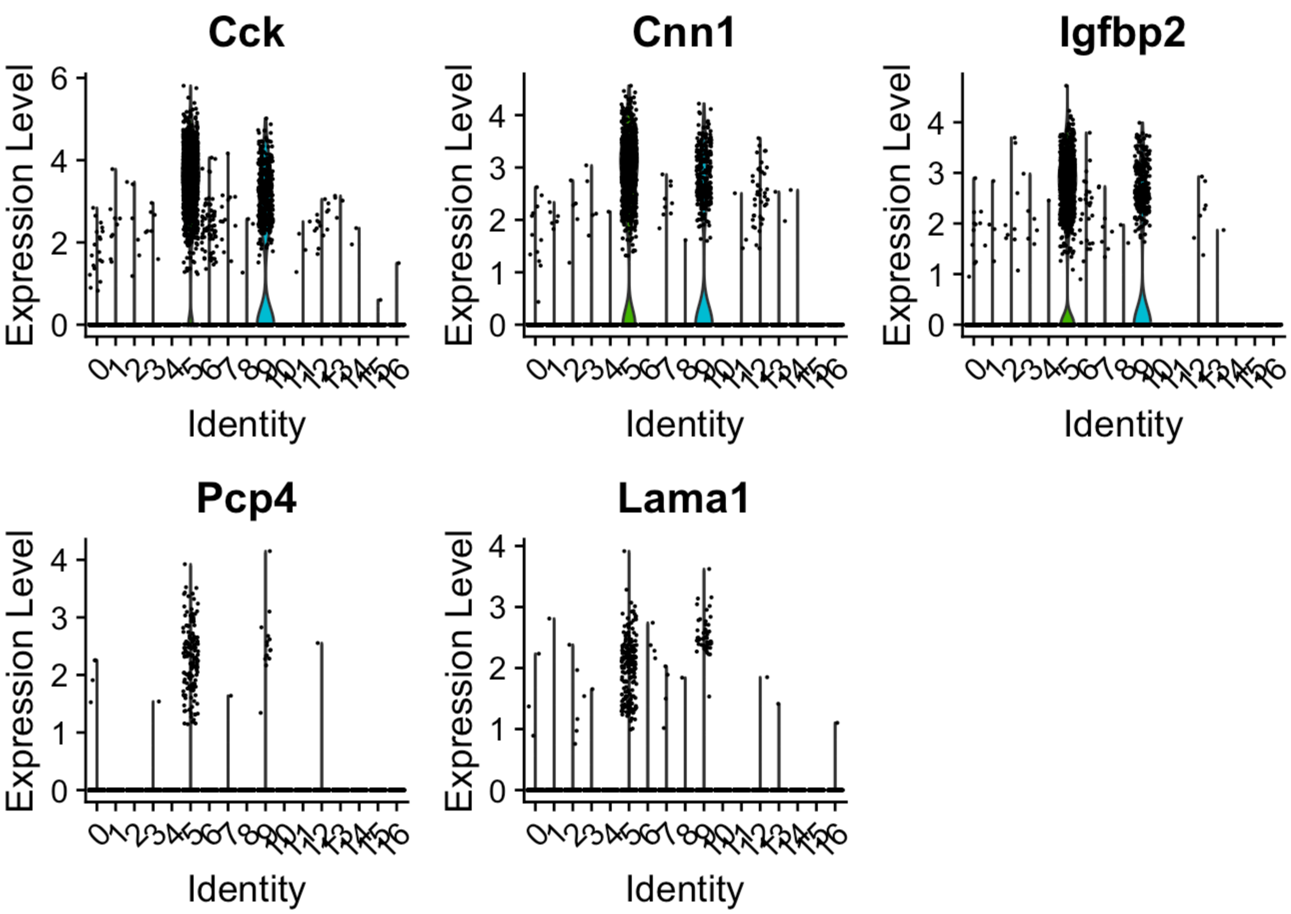


```
#Lama1 is a basement membrane marker; glandular epithelial cells, fibroblast
#Pcp4+ Smooth Muscle
#Cnn1 smooth muscle and myoepithelial cells
#Igfbp2 Endometrial stromal cells, Granulosa cells, Breast myoepithelial cells, Hepatocy
tes
```

Cluster 6 is Ductal Cells

```
# Vln plot - cluster 6
VlnPlot(object = seurat_integrated,
        features = c("Gpha2", "Sfn", "Pdzrn4", "Krt15"))
```

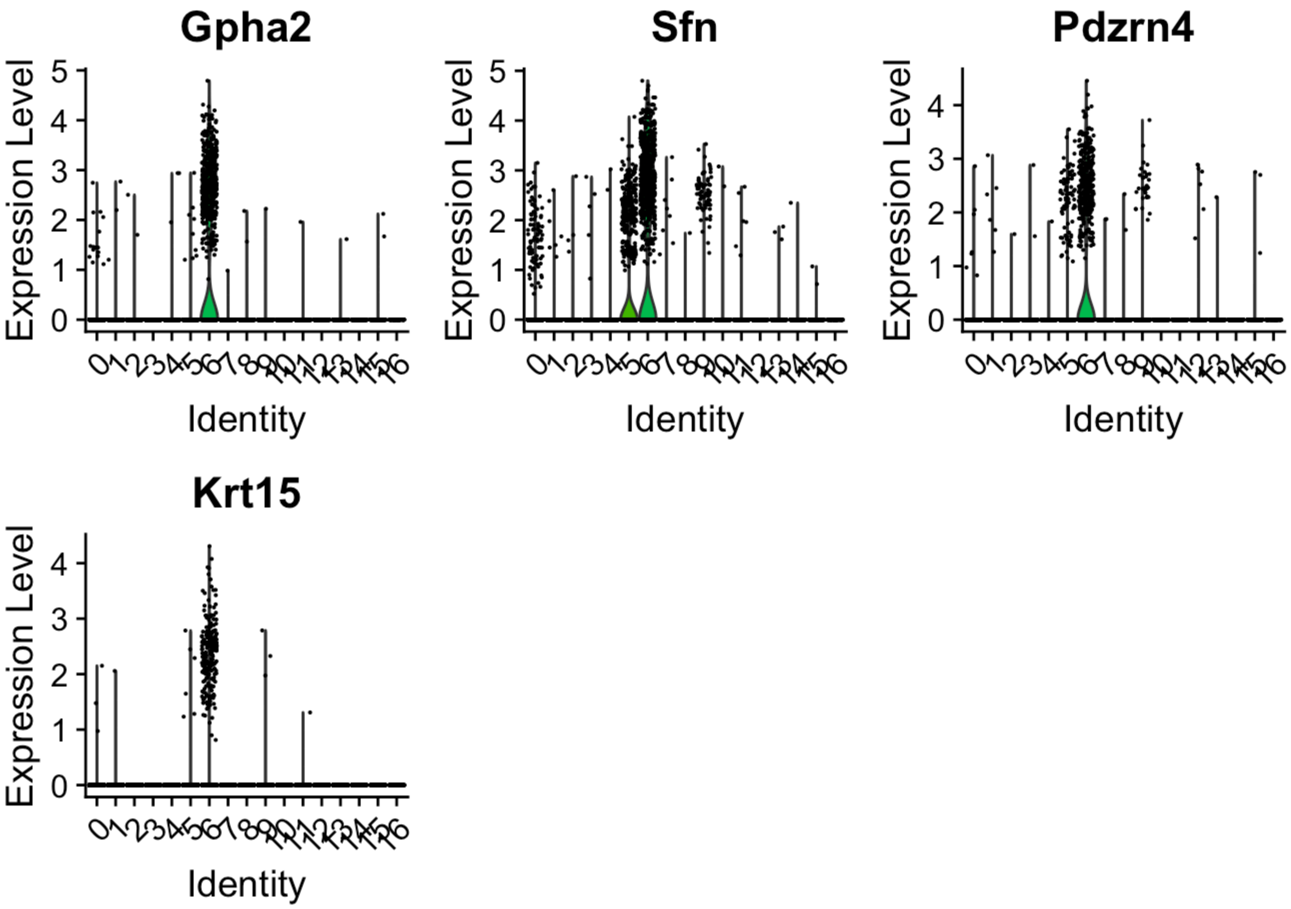


```
#Gpha2 and Crisp1 are more specific to cluster 6

VlnPlot(object = seurat_integrated,
        features = c("Icam1", "Aqp5"))
```

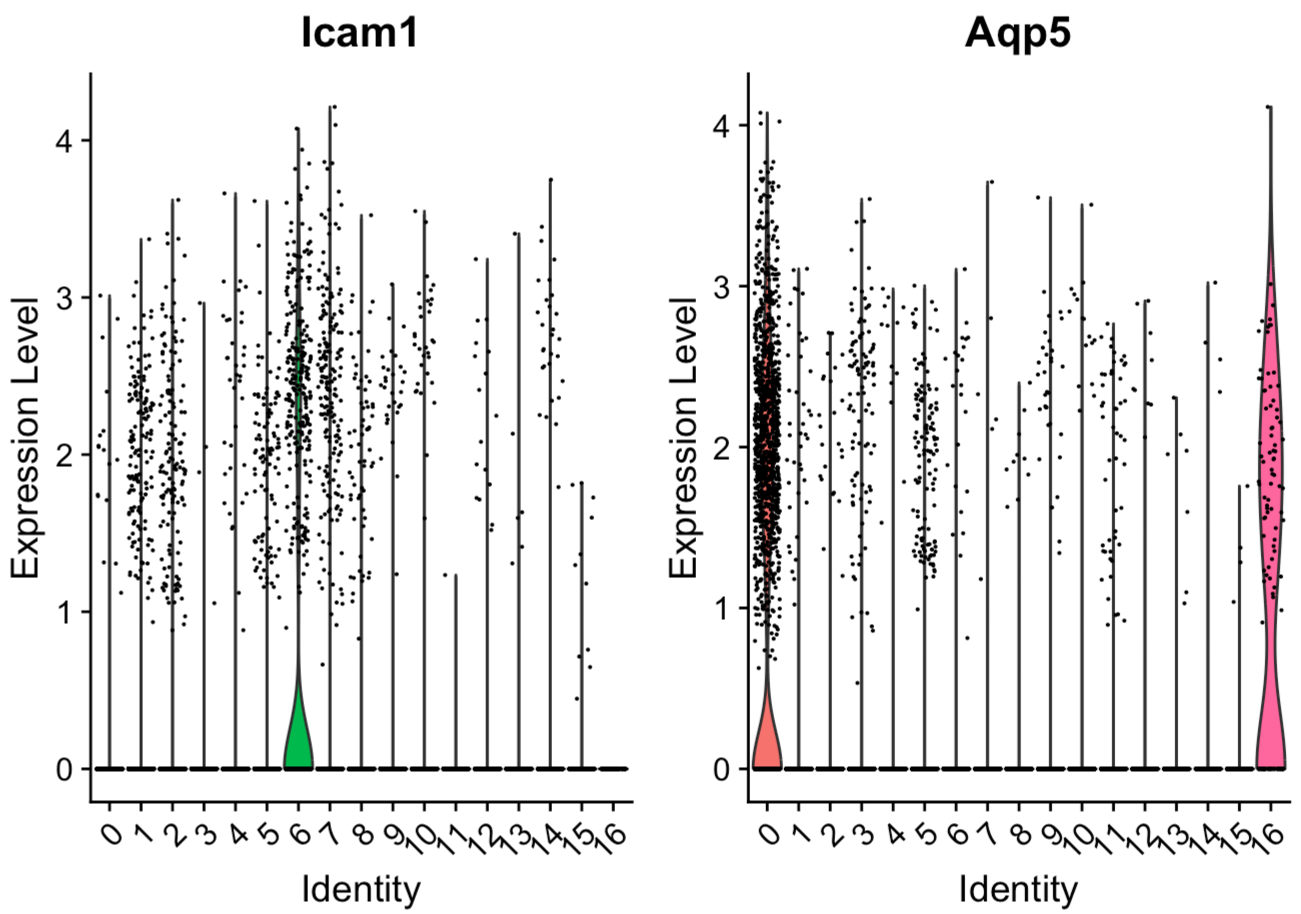


```
#Gpha2: Exocrine glandular cells
#Crisp1: Salivary glandular cells
#Krt17: Salivary ductal cells; is indicative of a certain type of epithelial 'stem cell
s'; Breast Myoepithelial cells
#Sfn: Mucus Glandular Cells, Salivary Duct cells
#Pdzrn4 Smooth muscle cell marker
#Krt15 : Salivary ductal cells
```

Cluster 7 is Endothelial

```
# Vln plot - cluster 7
VlnPlot(object = seurat_integrated,
        features = c("Rbp7", "Gpihbp1", "Col13a1", "Cyyr1", "Ptprb", "Shank3", "Myct1"))
```

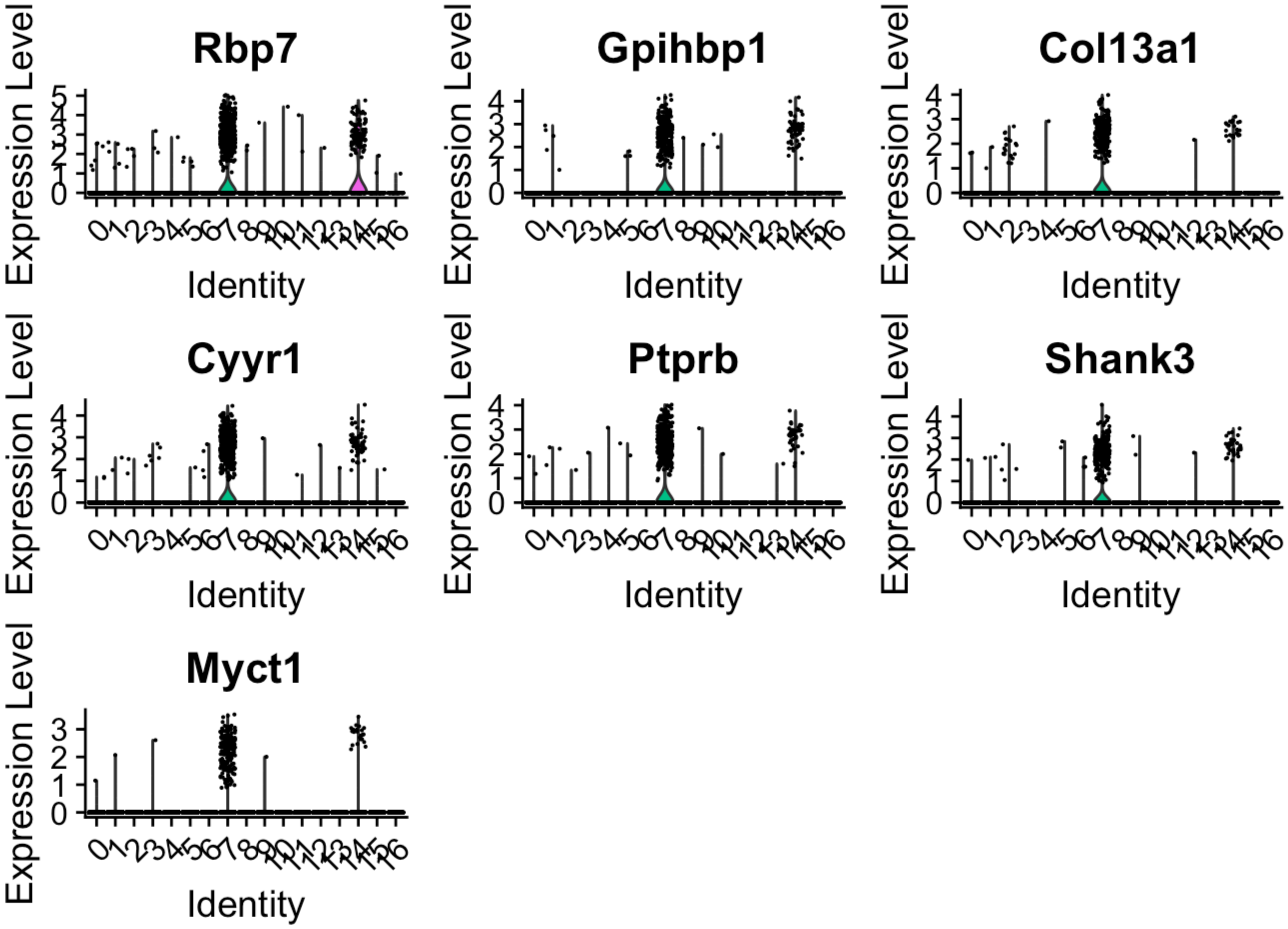


```
#Rbp7: Adipocytes, Endothelial
#Gpihbp1: Endothelial(Adipocytes)
# Cyyr1: Core cell type enriched (Endothelial cells); also in plasmacytoid DC, cluster 1
4 may be DC
```

Cluster 7 is Endothelial Cell

```
# Vln plot - cluster 7
VlnPlot(object = seurat_integrated,
        features = c( "Aqp1", "Fabp4", "Lrg1", "Vwf", "Ackr1"))
```

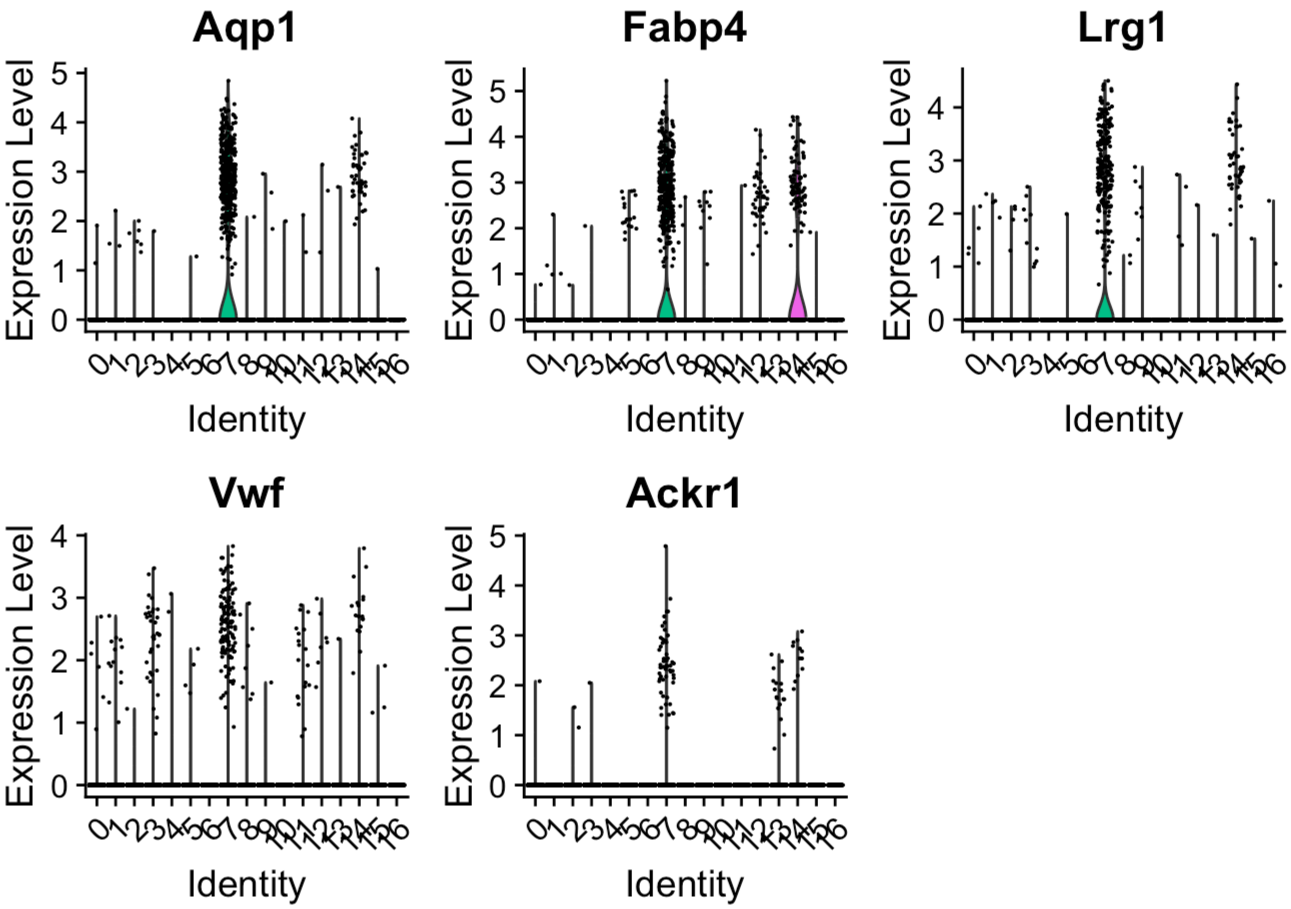


```
# Lrg1: glandular cells
#Aqp1
```

Cluster 8 is Monocyte/APC

```
# Vln plot - cluster 8
VlnPlot(object = seurat_integrated,
        features = c("Cd209a", "Mcemp1", "Flt3", "Gpr141"))
```

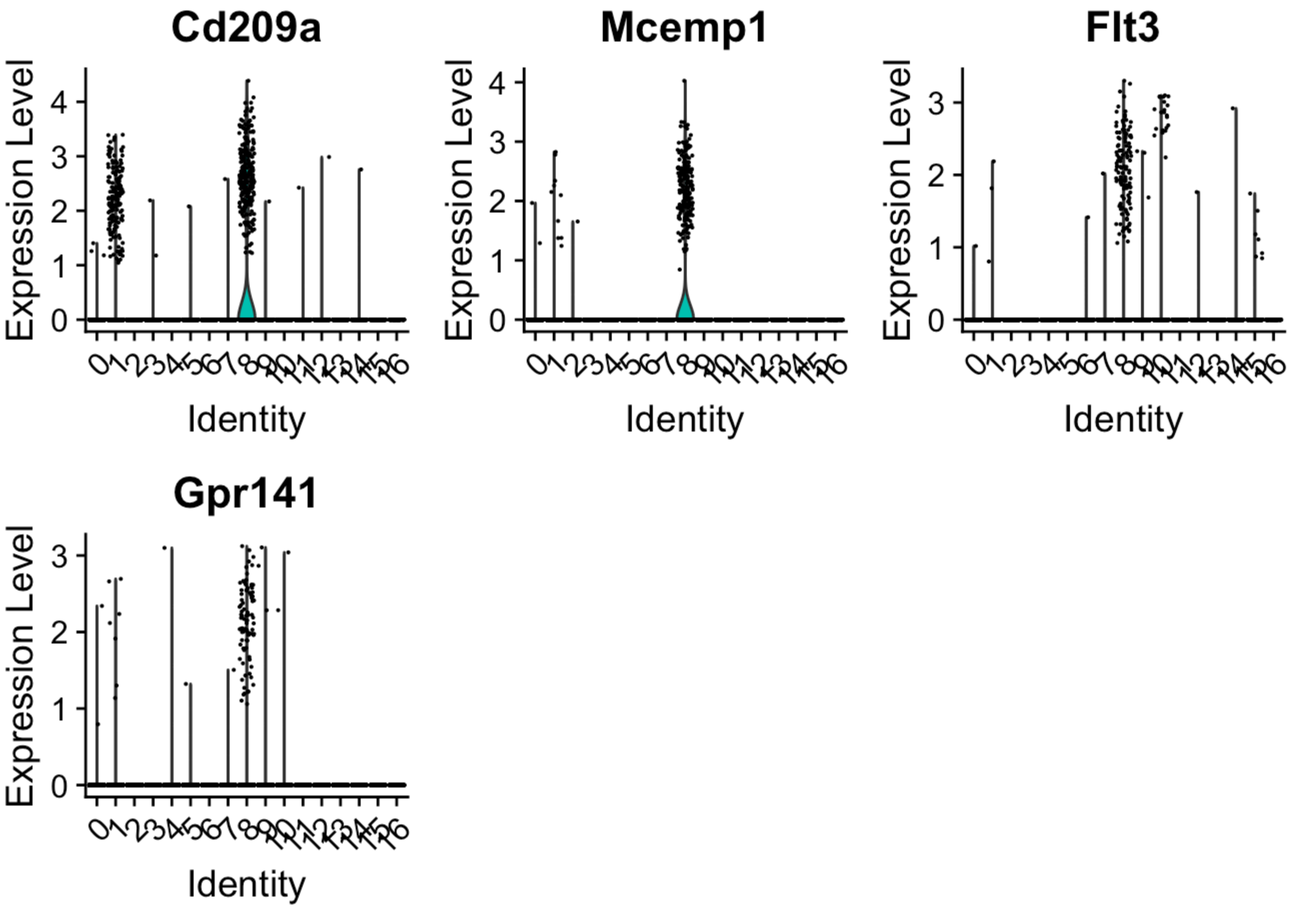


```
# Flt3: plasmacytoid DC
# Ccl2: Langerhans cell
# Mcemp1: Monocytes & Neutrophils
# Gpr141: Also present in memory CD8 T-cell, naive CD8 T-cell
#Macrophage lineage
```

#Cluster 9 is a smooth muscle

```
# Vln plot - cluster 9
VlnPlot(object = seurat_integrated,
        features = c("Cdh19", "Plp1", "Kcna1", "Acta2"))
```

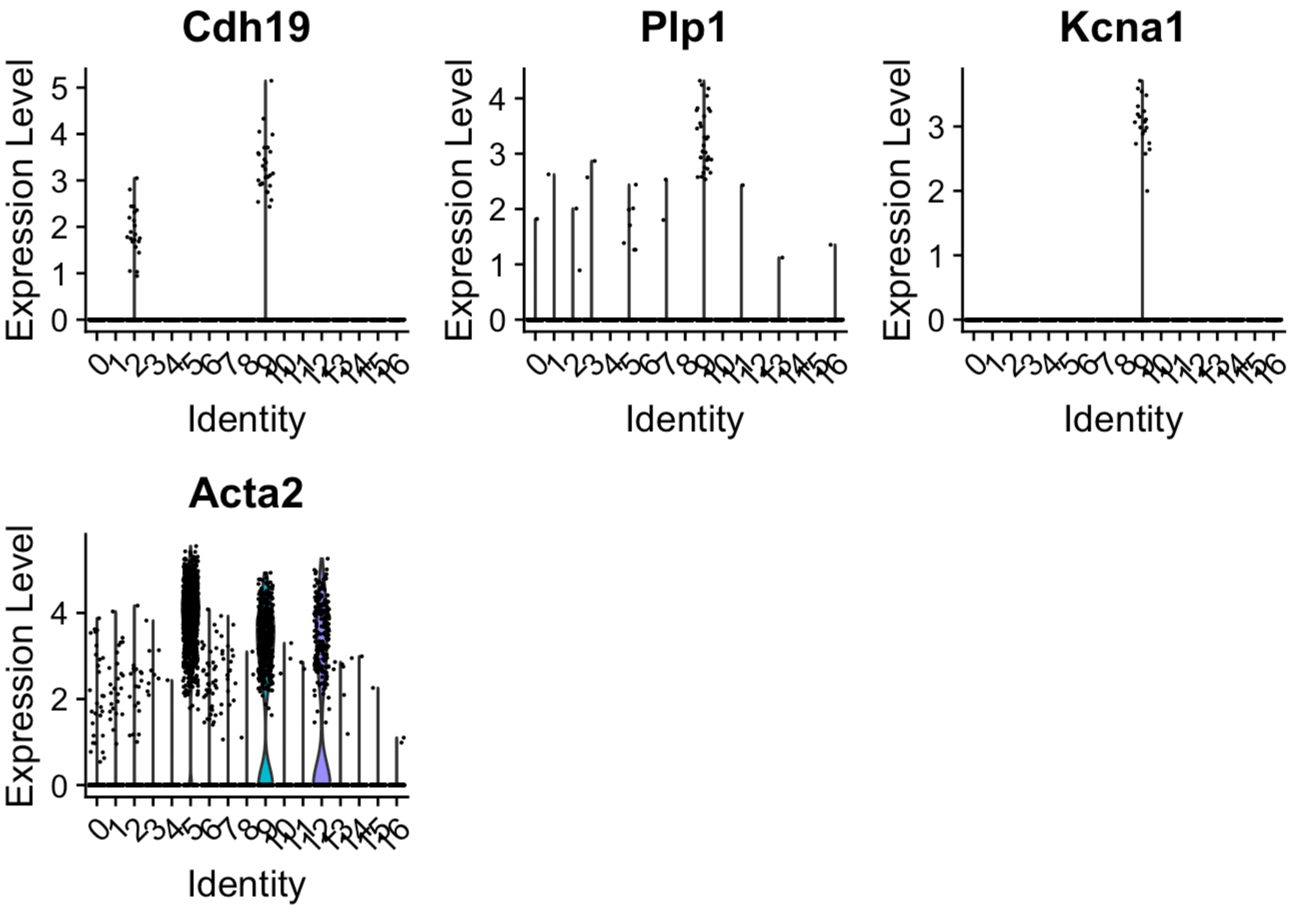

Cluster 9 is Myoepithelial cell

```
# Vln plot - cluster 9
VlnPlot(object = seurat_integrated,
        features = c("Cdh19", "Slc35f1", "Plp1", "S100b", "Cadm2"))
```

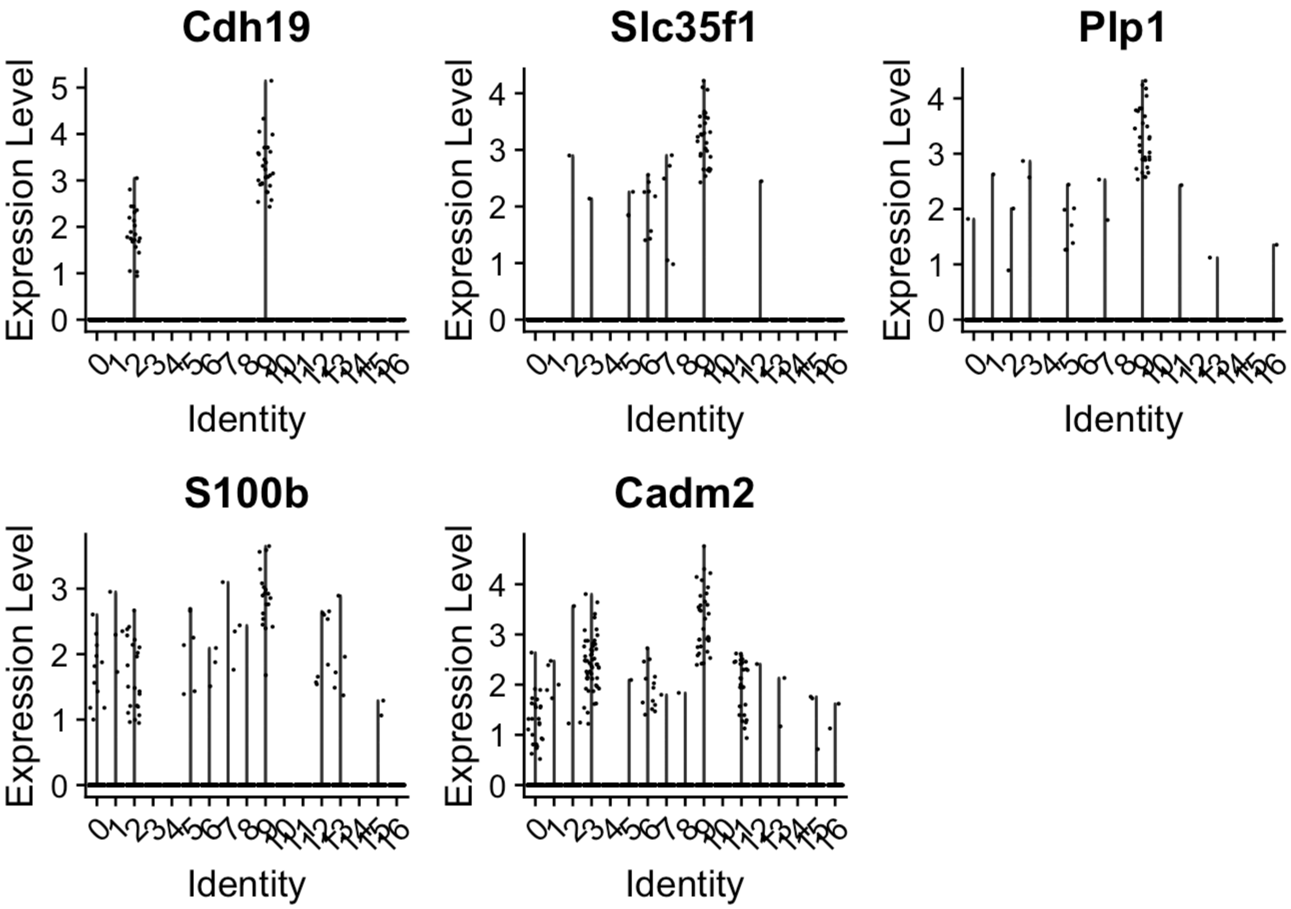

Cluster 10 is naive B-cell (Memory B cells)

H2-Ob (ortholog of HLA-DOB) Cell type enriched (Minor Salivary Gland - Plasma cells, Spleen - B-cells (Spleen))

```
# Vln plot - cluster 10
VlnPlot(object = seurat_integrated,
        features = c("Cd79a", "Bank1", "Ms4a1", "Tnfrsf13c", "H2-Ob", "Blk", "Ighd", "Fc
mr"))
```

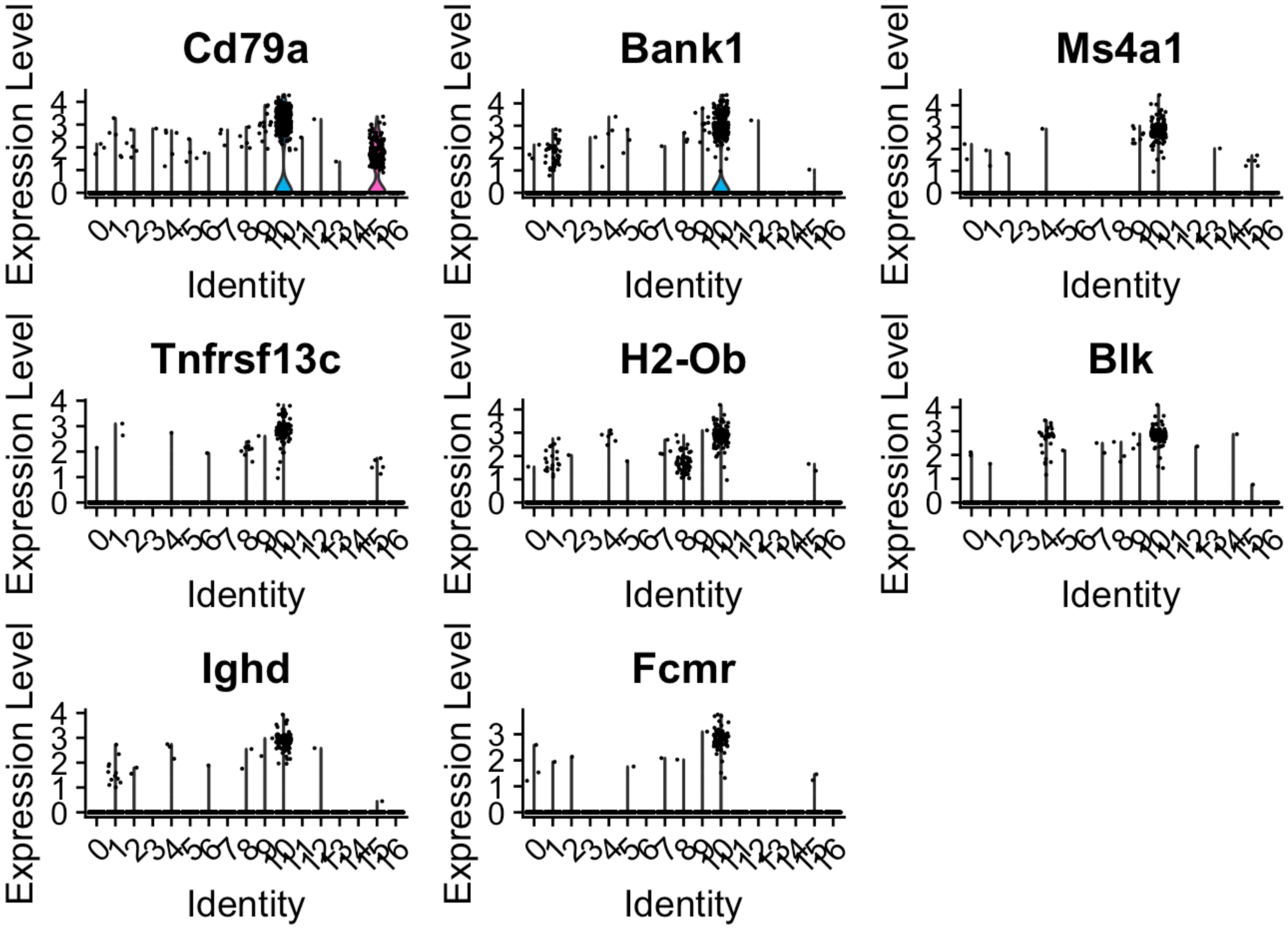


```
#15 might be plasma cell
```

Cluster 11 is a type of glandular cell (Acinar III)

```
# Vln plot - cluster 11
VlnPlot(object = seurat_integrated,
        features = c("Gm46218", "Gm46573", "Scd3"),
        split.by = "sample")
```

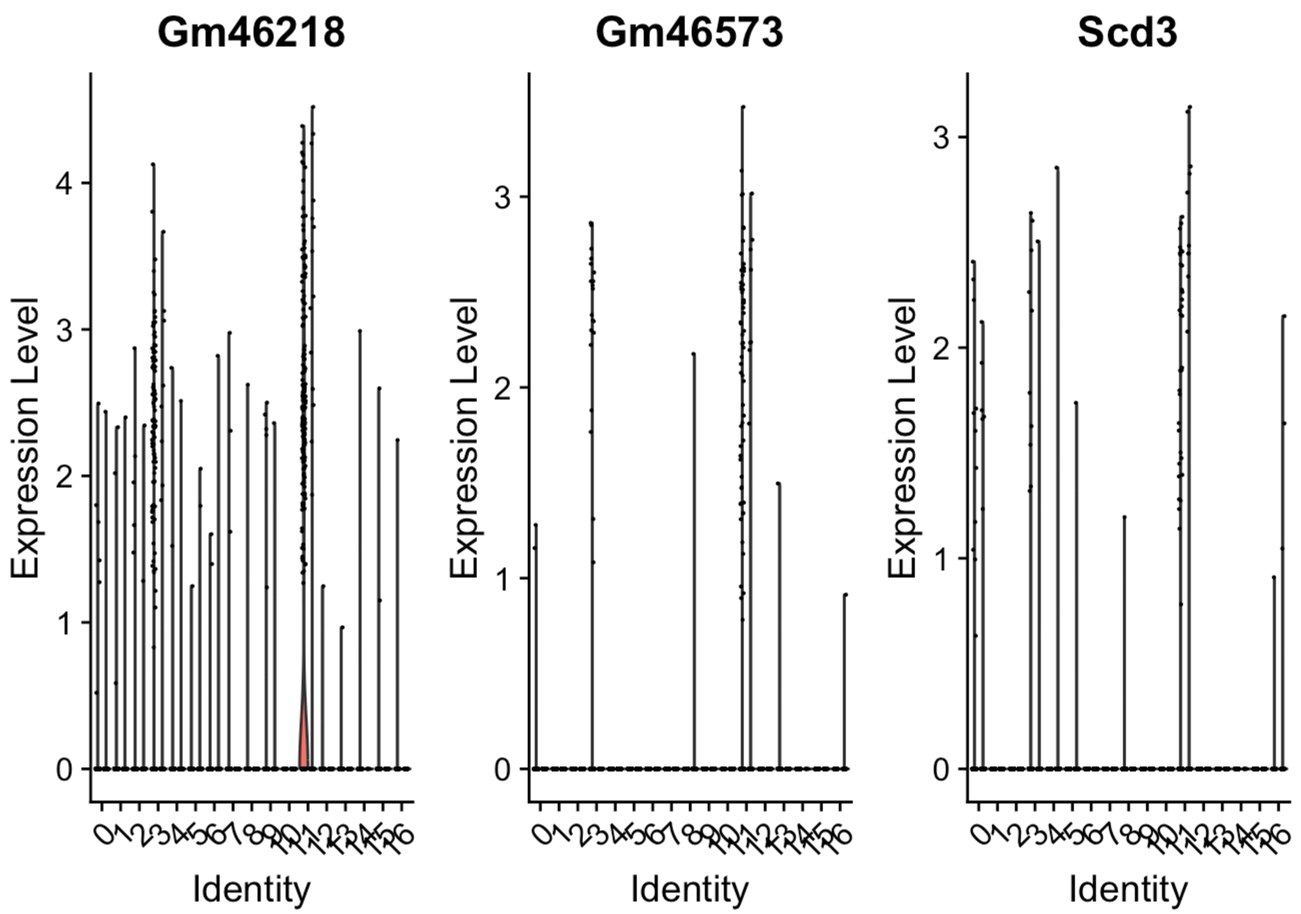

Cluster 12 is Perivascular Cell Myoepithelial Cell

```
# Vln plot - cluster 12
VlnPlot(object = seurat_integrated,
        features = c("Colec11" , "Map3k7cl", "Rgs4", "Myocd","Acta2", "Pdgfrb"))
```

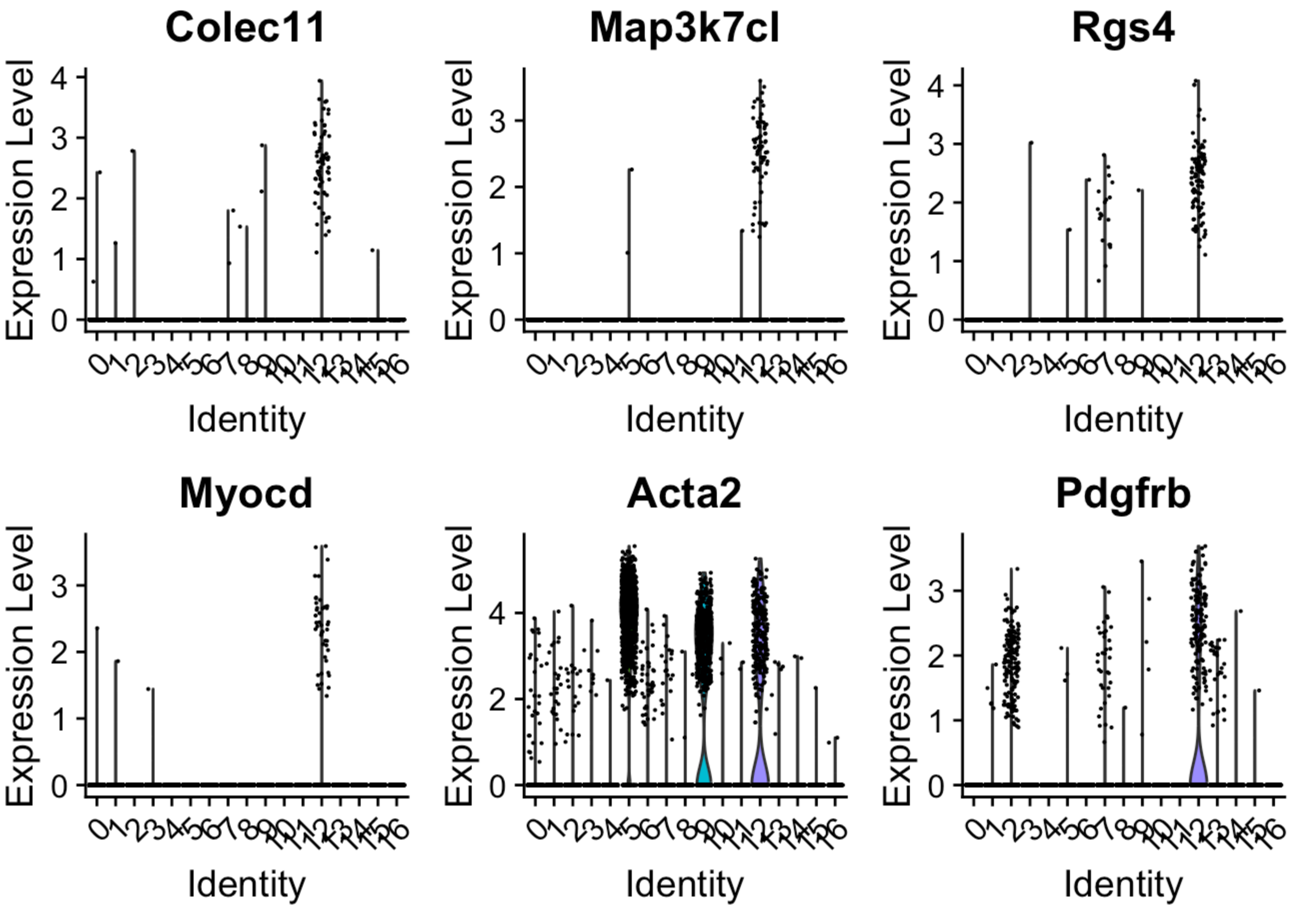


```
#Rgs4 Smooth Muscle Cell, Fibroblast
#Myocd Smooth Muscle Cell
```

Cluster 13 is Squamous Epithelial Cells Opcml is found in salivary fibroblasts Fndc1 also a Fibroblasts

```
# Vln plot - cluster 13
VlnPlot(object = seurat_integrated,
        features = c("Krtdap", "Opcml", "Smpd3", "Fndc1"))
```

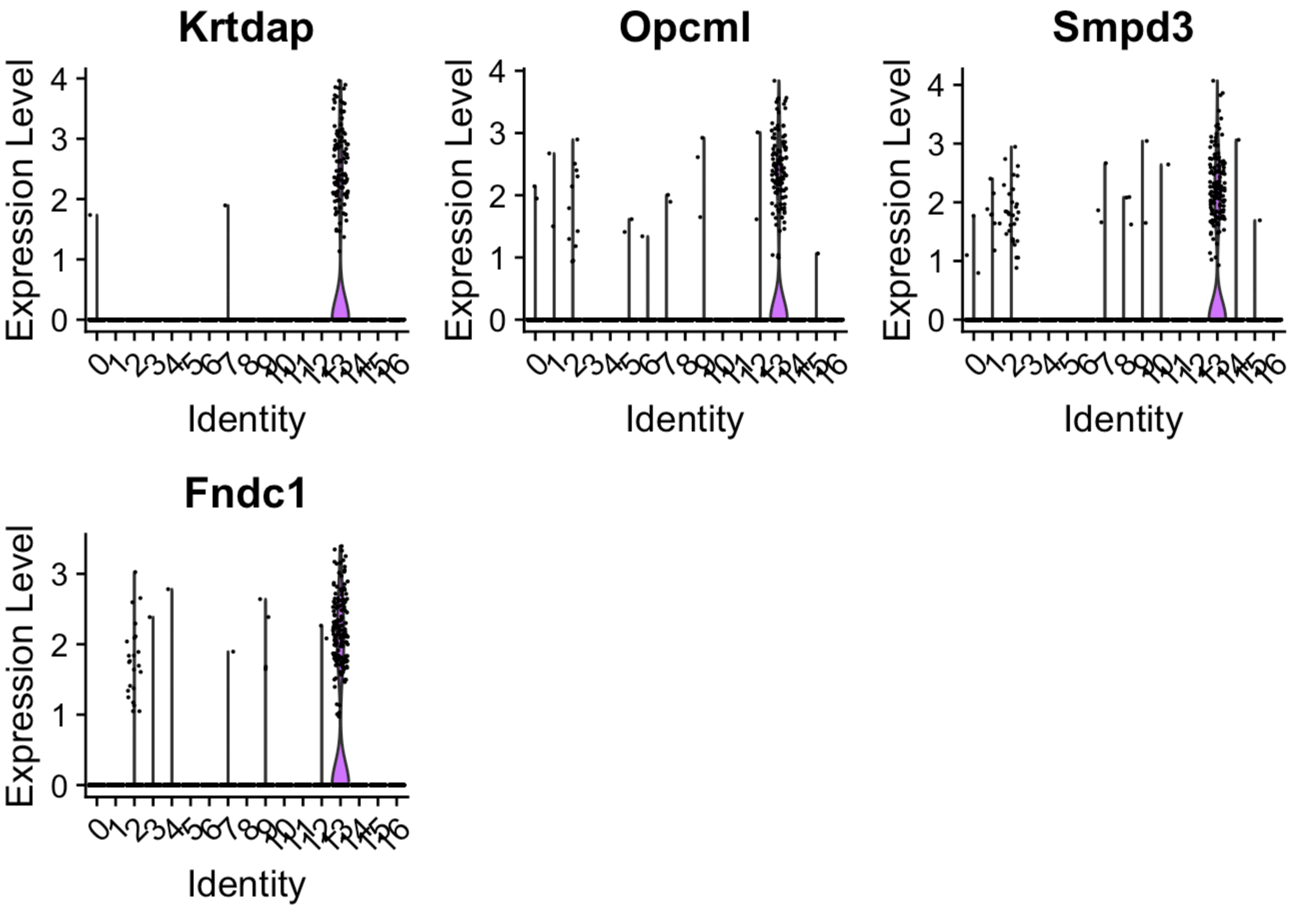

Cluster 14 is Endothelial

```
# Vln plot - cluster 14
VlnPlot(object = seurat_integrated,
        features = c("Exoc3l", "Gm20621", "Car4", "Reep1", "Ushbp1"))
```

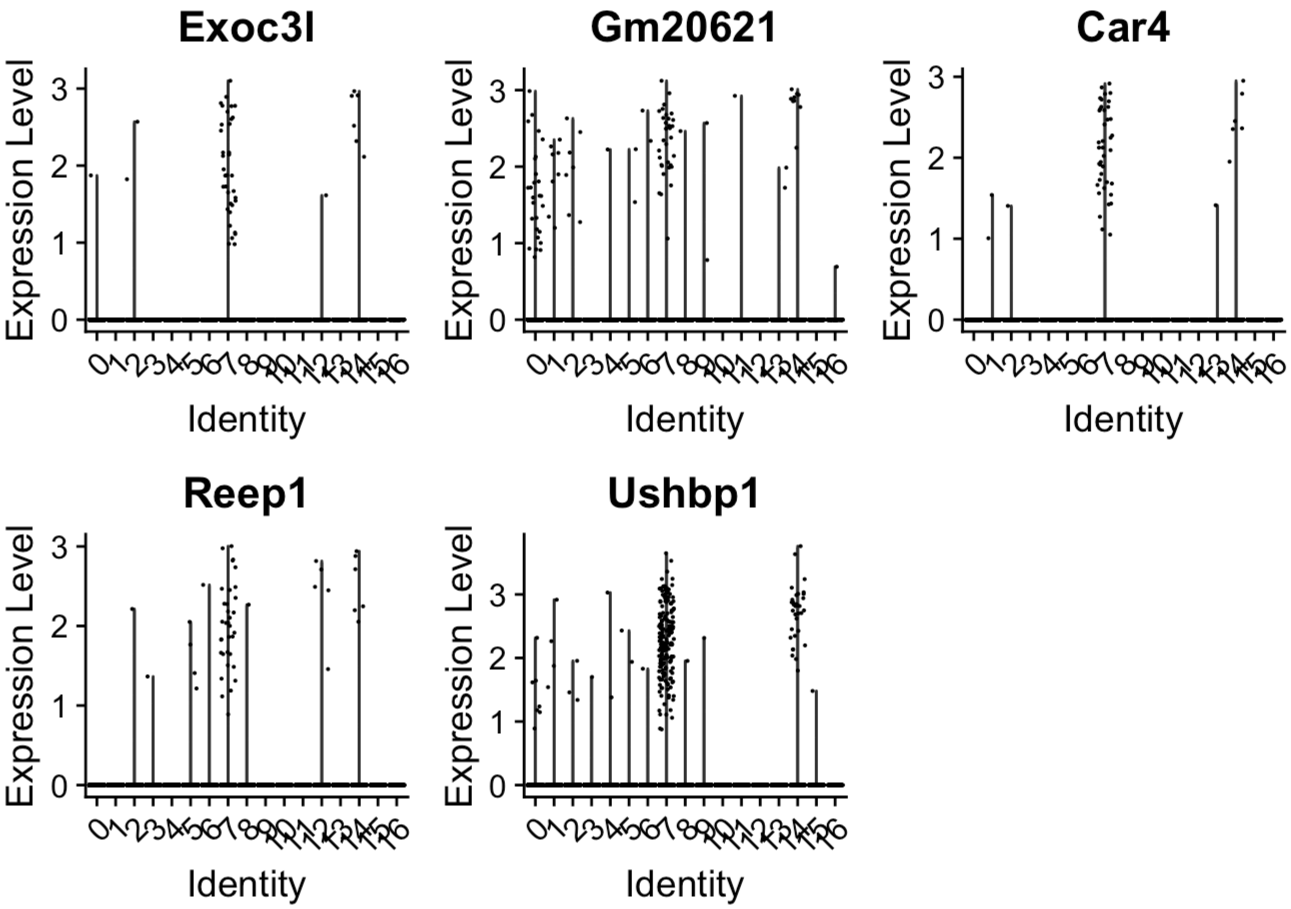


```
#Similar to cluster 7
# Endothelial Cell
```

Cluster 15 is Plasma Cell

```
# Vln plot - cluster 15
VlnPlot(object = seurat_integrated,
        features = c("Iglv1", "Iglc1", "Jchain", "Iglc3", "Igha"))
```

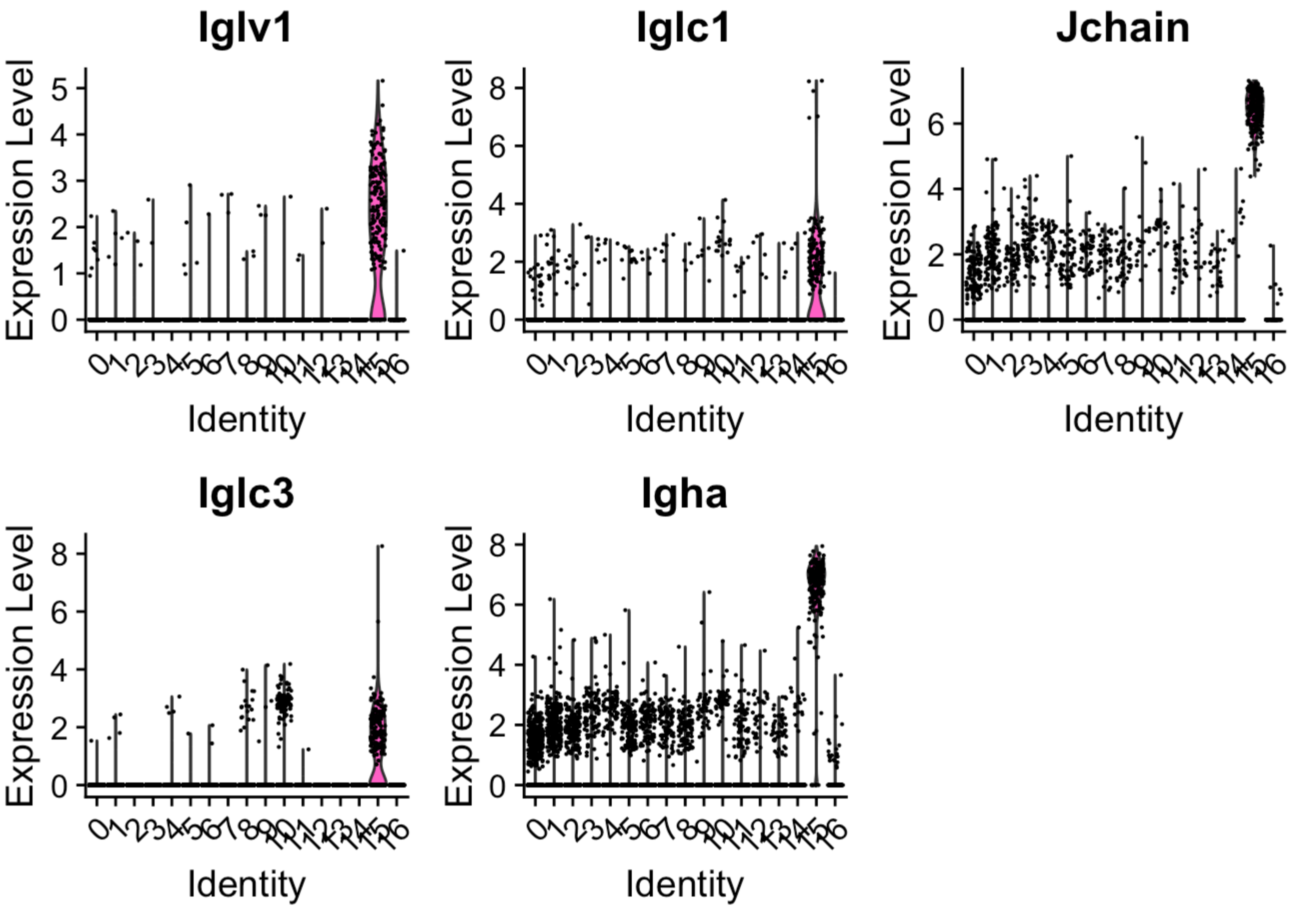


```
VlnPlot(object = seurat_integrated,
        features = c("Igha", "Ighg2b", "Igkc", "Ighm"),
        split.by="sample")
```

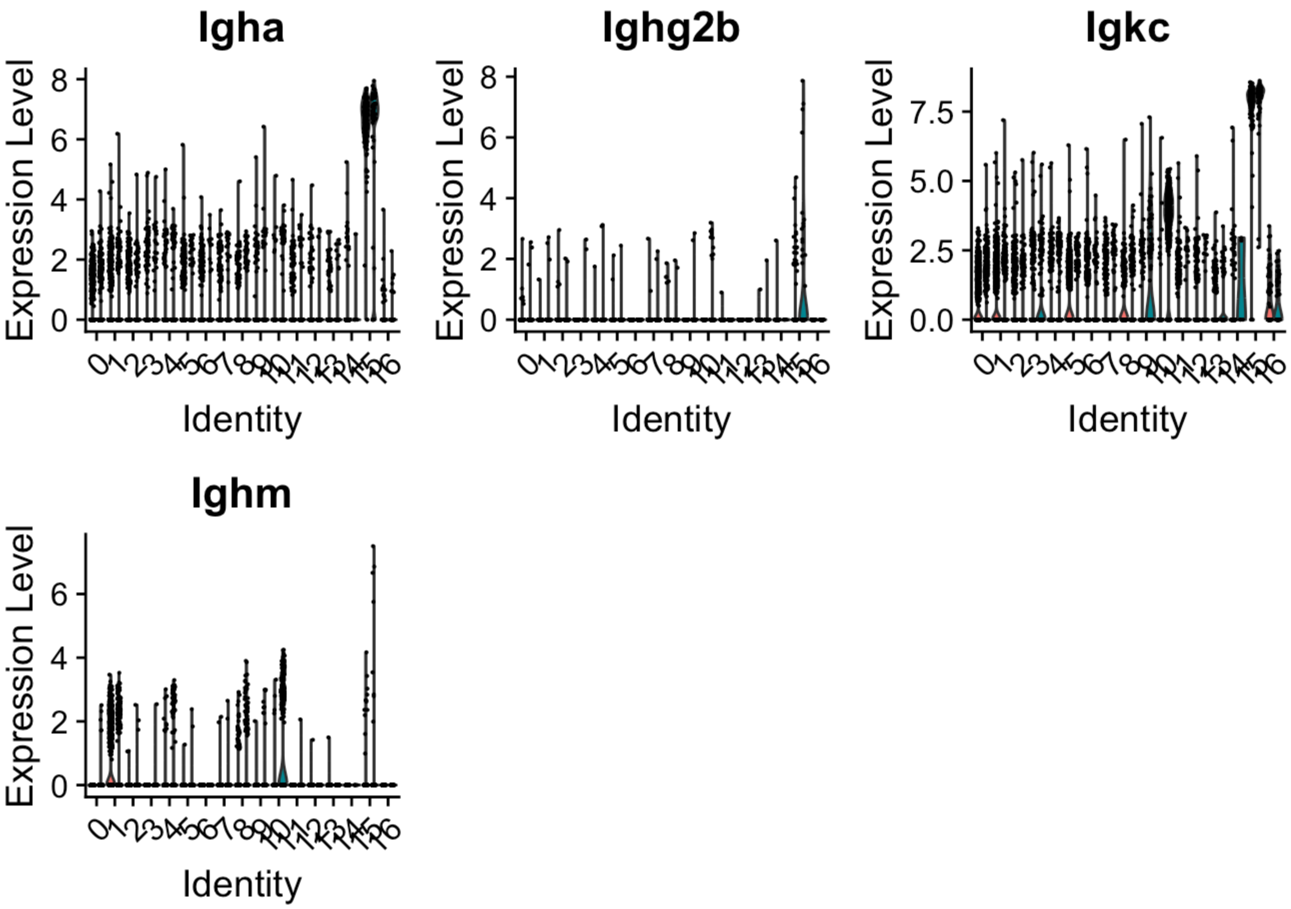

Cluster 16 is Car6+ Acini

```
# Vln plot - cluster 16
VlnPlot(object = seurat_integrated,
        features = c( "Car6", "Mup5", "Mup6", "Dnase1", "Odam"))
```

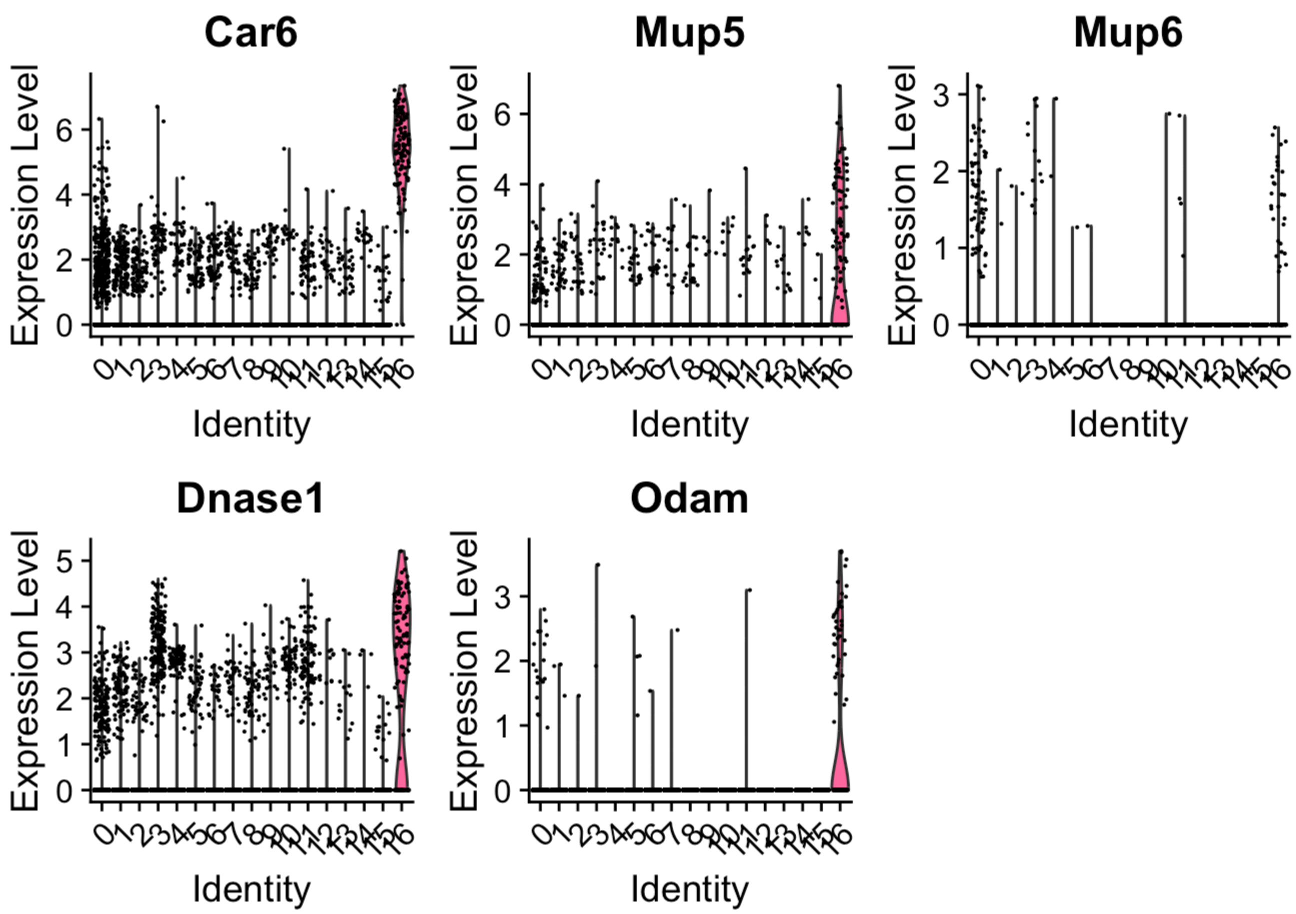


```
#Odam: Serous glandular cells –
```

Rename all Clusters

```
# Rename all identities
#seurat_integrated <- RenameIdents(object = seurat_integrated, cells=0:17)
seurat_integrated <- RenameIdents(object = seurat_integrated,
                               "0" = "Acinar I",
                               "1" = "F13a1+ve Macrophage",
                               "2" = "Fibroblast",
                               "3" = "Acinar II",
                               "4" = "NK & T-cells",
                               "5" = "Myoepithelial I",
                               "6" = "Ductal Cell ",
                               "7" = "Endothelial I",
                               "8" = "Plasmacytoid DC",
                               "9" = "Myoepithelial",
                               "10" = "B-cell/naive B-cell",
                               "11" = "Acinar III",
                               "12" = "Pericyte",
                               "13" = "Fibroblasts",
                               "14" = "Endothelial II",
                               "15" = "Plasma Cells",
                               "16" = "Car6+ Acini"
                               )

# Plot the UMAP
DimPlot(object = seurat_integrated,
        reduction = "umap",
        label = TRUE,
        label.size = 3,
        repel = TRUE)
```

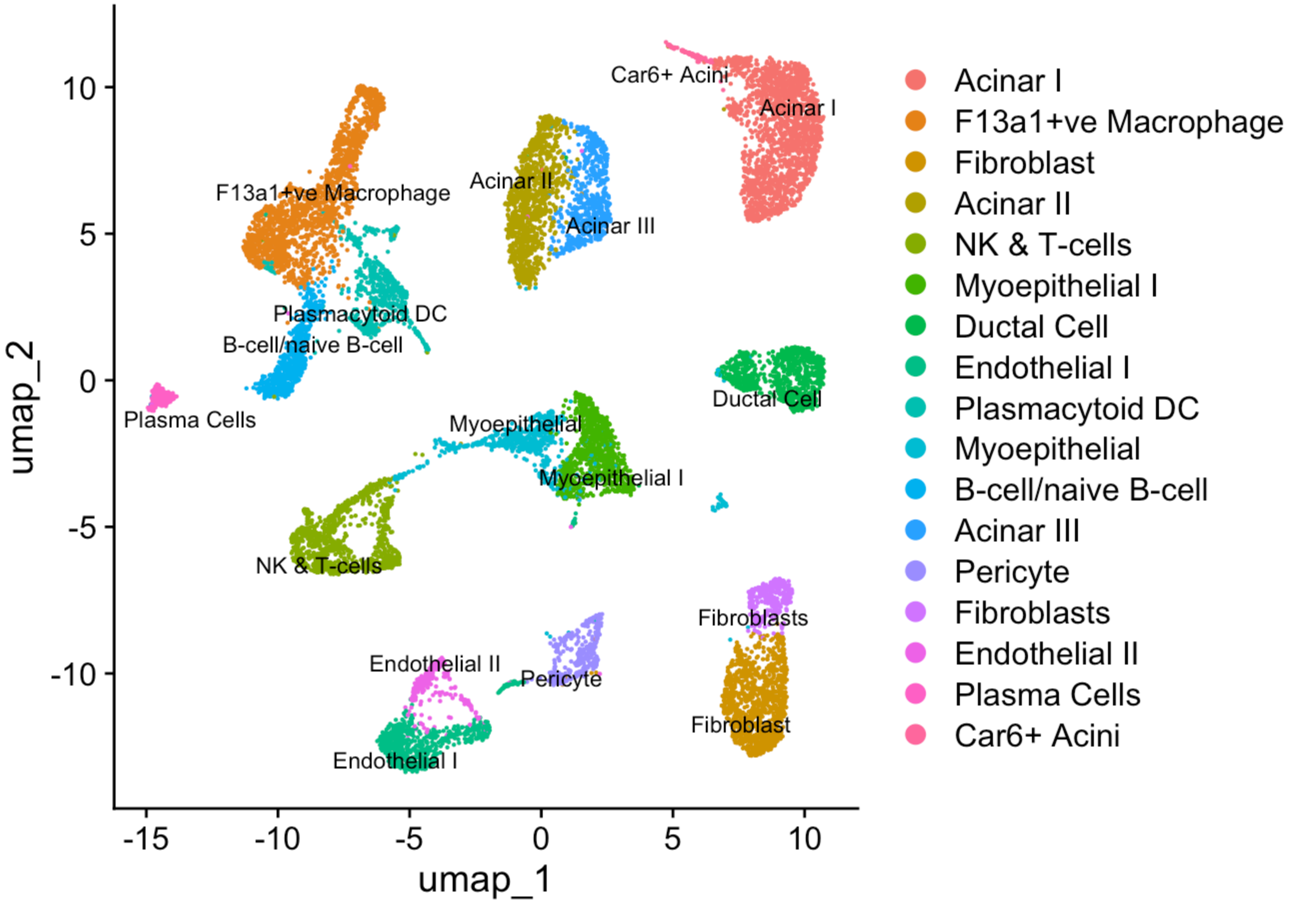


```
setwd("~/Documents/3_Parkinsons_disease/2019/scRNAseq/results/")
#tiff(filename = "UMAP_2.tiff", height=4.5, width=9, res=300, units = "in")
DimPlot(object = seurat_integrated,
        reduction = "umap",
        label = TRUE,
        label.size = 2.5,
        repel = TRUE,
        split.by = "sample")
```

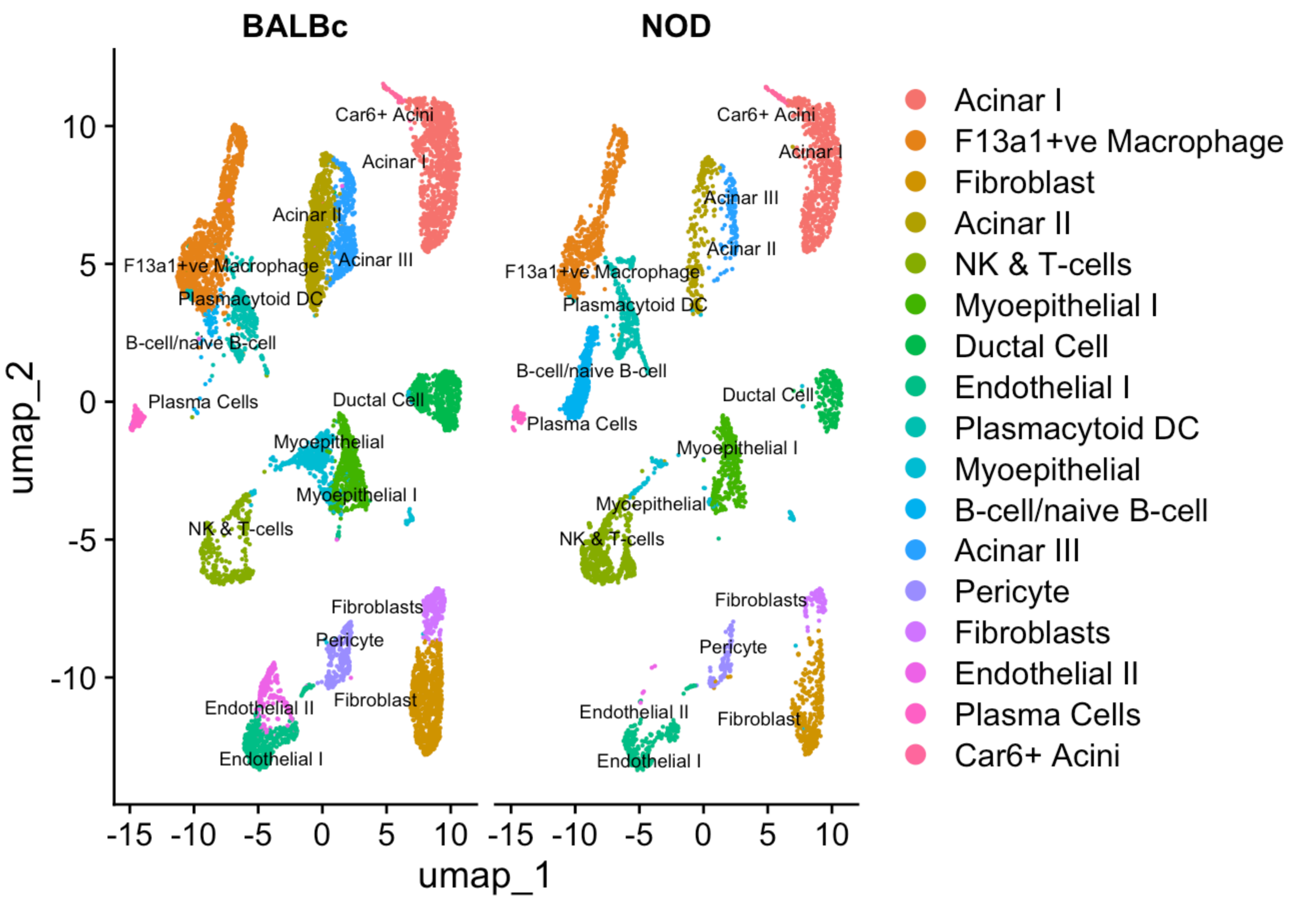


```
#dev.off()
```

IgG Gene UMAP

```
setwd("~/Documents/3_Parkinsons_disease/2019/scRNAseq/results/")
#tiff(filename = "IgG_.tiff", height=4, width=8, res=300, units = "in")
FeaturePlot(seurat_integrated,
            reduction = "umap",
            features = c("Ighg2b"),
            order = TRUE,
            min.cutoff = 'q5',
            label = TRUE,
            label.size = 2.5,
            split.by = "sample",
            repel=TRUE)
```

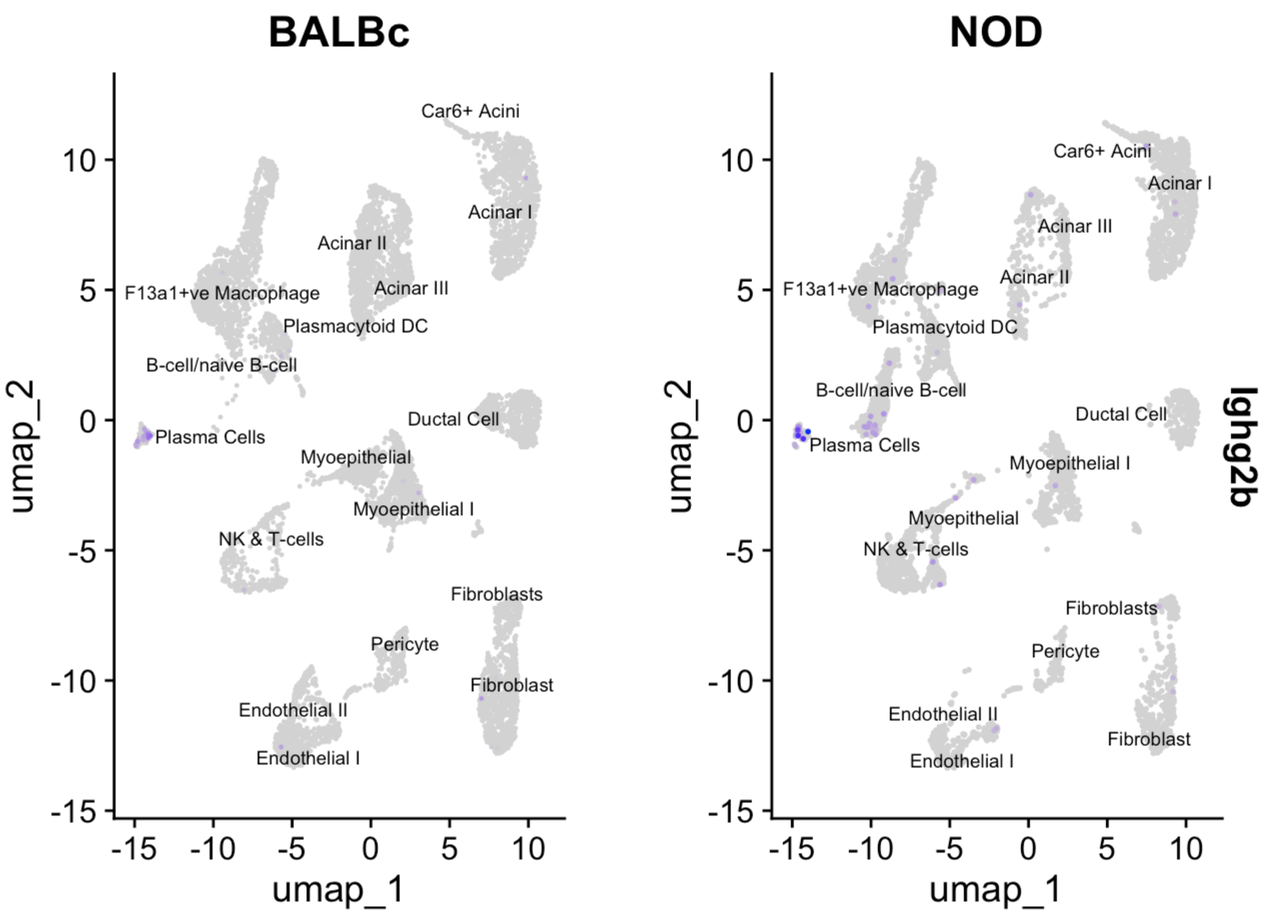


```
#dev.off()

setwd("~/Documents/3_Parkinsons_disease/2019/scRNAseq/results/")
#tiff(filename = "Celltype_2.tiff", height=4, width=8, res=300, units = "in")
DimPlot(object = seurat_integrated,
        reduction = "umap",
        #features = c("Igha", "Ighm", "Ighg2b"),
        label = TRUE,
        label.size = 3,
        repel = TRUE,
        split.by = "sample")
```

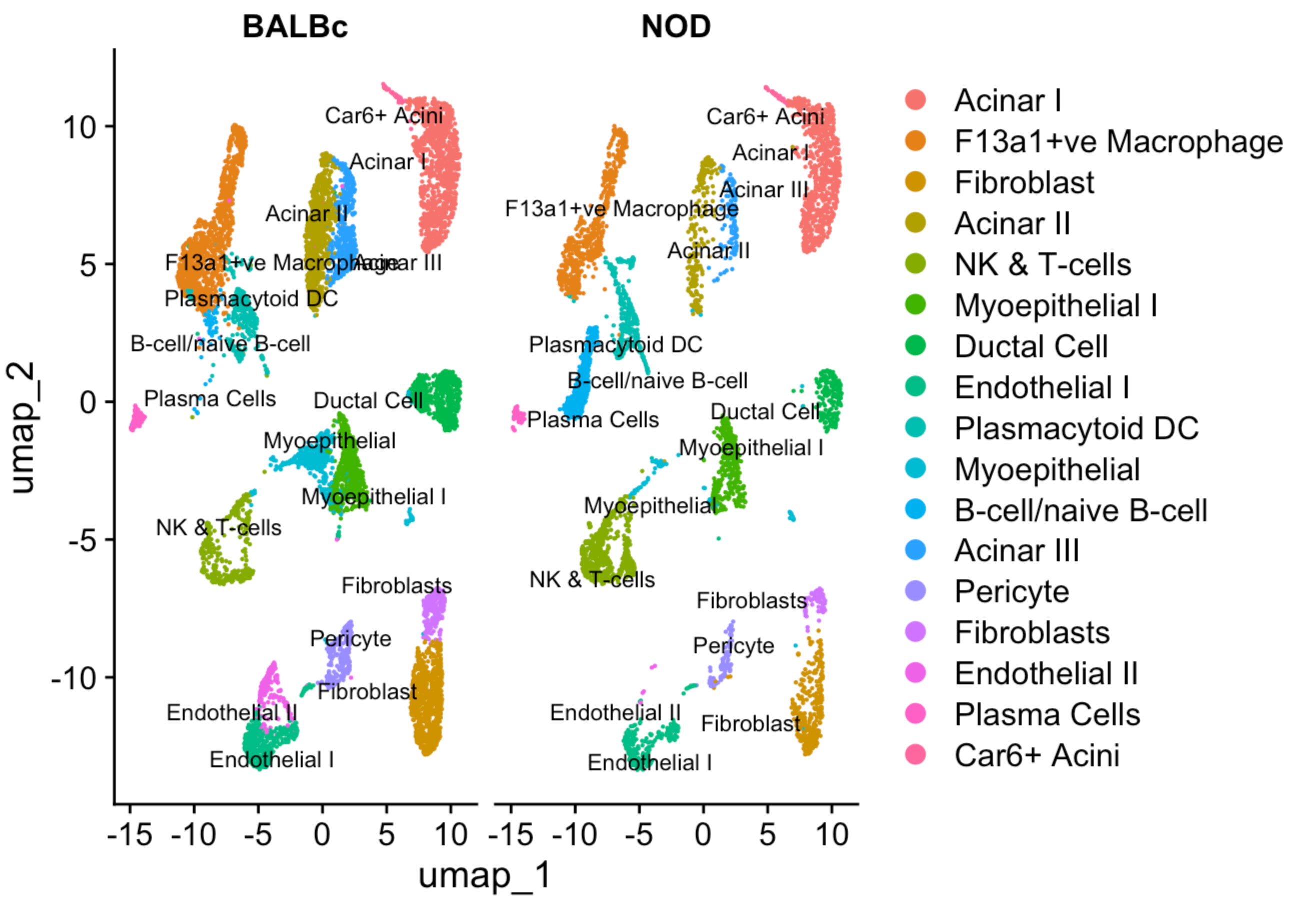


```
#dev.off()
```

Expression of Ig Transporter genes

```
setwd("~/Documents/3_Parkinsons_disease/2019/scRNAseq/results/")
#tiff(filename = "Fcgrt.tiff", height=10, width=7, res=300, units = "in")
FeaturePlot(seurat_integrated,
            reduction = "umap",
            features = c("Fcgr1", "Pigr", "Fcgrt"),
            order = TRUE,
            min.cutoff = 'q5',
            label = TRUE,
            label.size = 2,
            split.by = "sample")
```

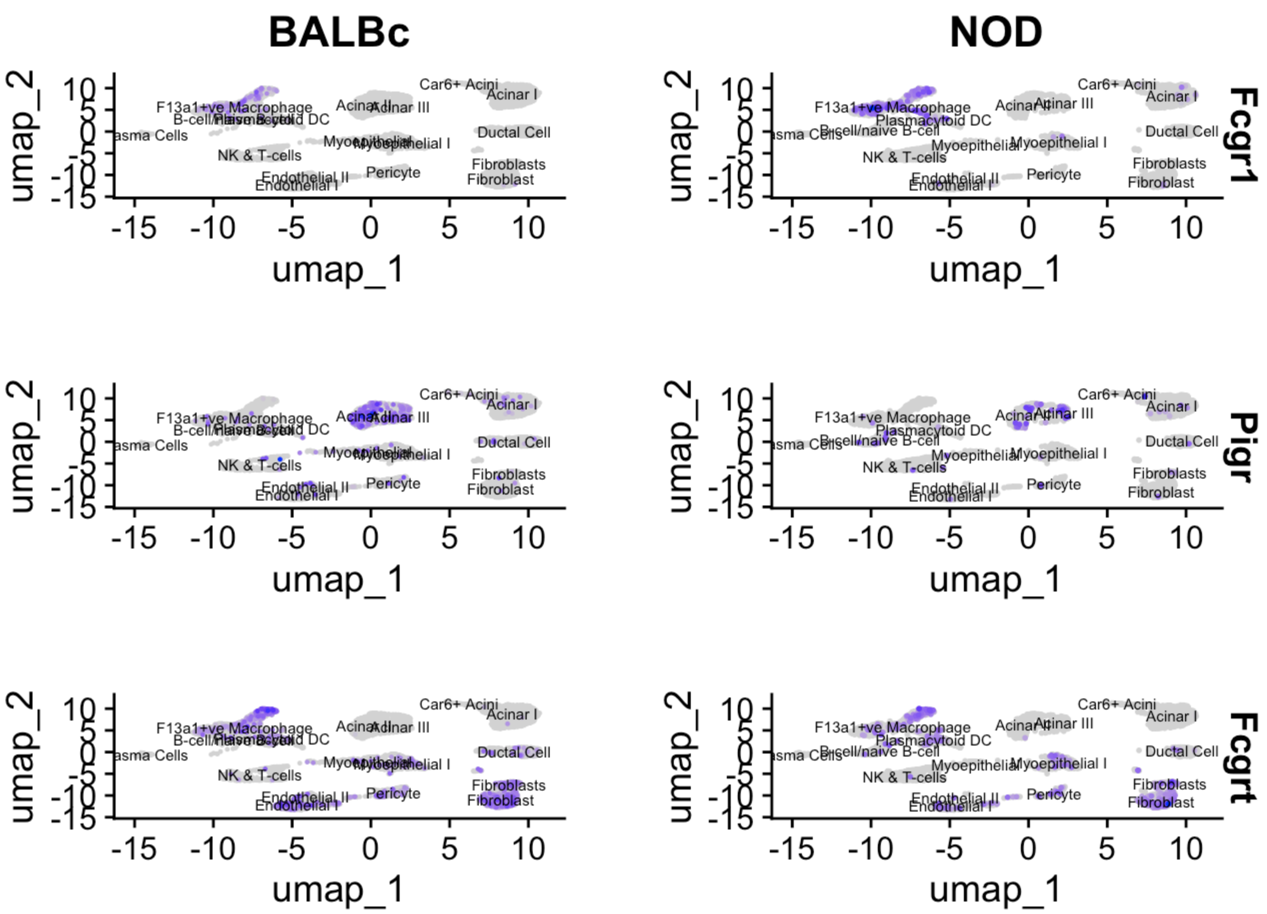


```
#dev.off()

setwd("~/Documents/3_Parkinsons_disease/2019/scRNAseq/results/")
#tiff(filename = "Fcgrt_Vln.tiff", height=10, width=7, res=300, units = "in")
VlnPlot(object = seurat_integrated,
        idents=c("F13a1+ve Macrophage", "Fibroblast", "Fibroblasts", "Plasmacytoid DC",
"Endothelial I"),
        features = c("Fcgrt"),
        split.by = "sample") + ylim(0.025,5) +
   stat_summary(fun = median, fun.min = median, fun.max = median,
                 geom = "crossbar",
                 width = 0.5,
                 position = position_dodge(width = .70))
```

```
## Scale for y is already present.
## Adding another scale for y, which will replace the existing scale.
```

```
## Warning: Removed 3657 rows containing non-finite outside the scale range
## (`stat_ydensity()`).
```

```
## Warning: Removed 3657 rows containing non-finite outside the scale range
## (`stat_summary()`).
```

```
## Warning: Removed 3657 rows containing missing values or values outside the scale rang
e
## (`geom_point()`).
```

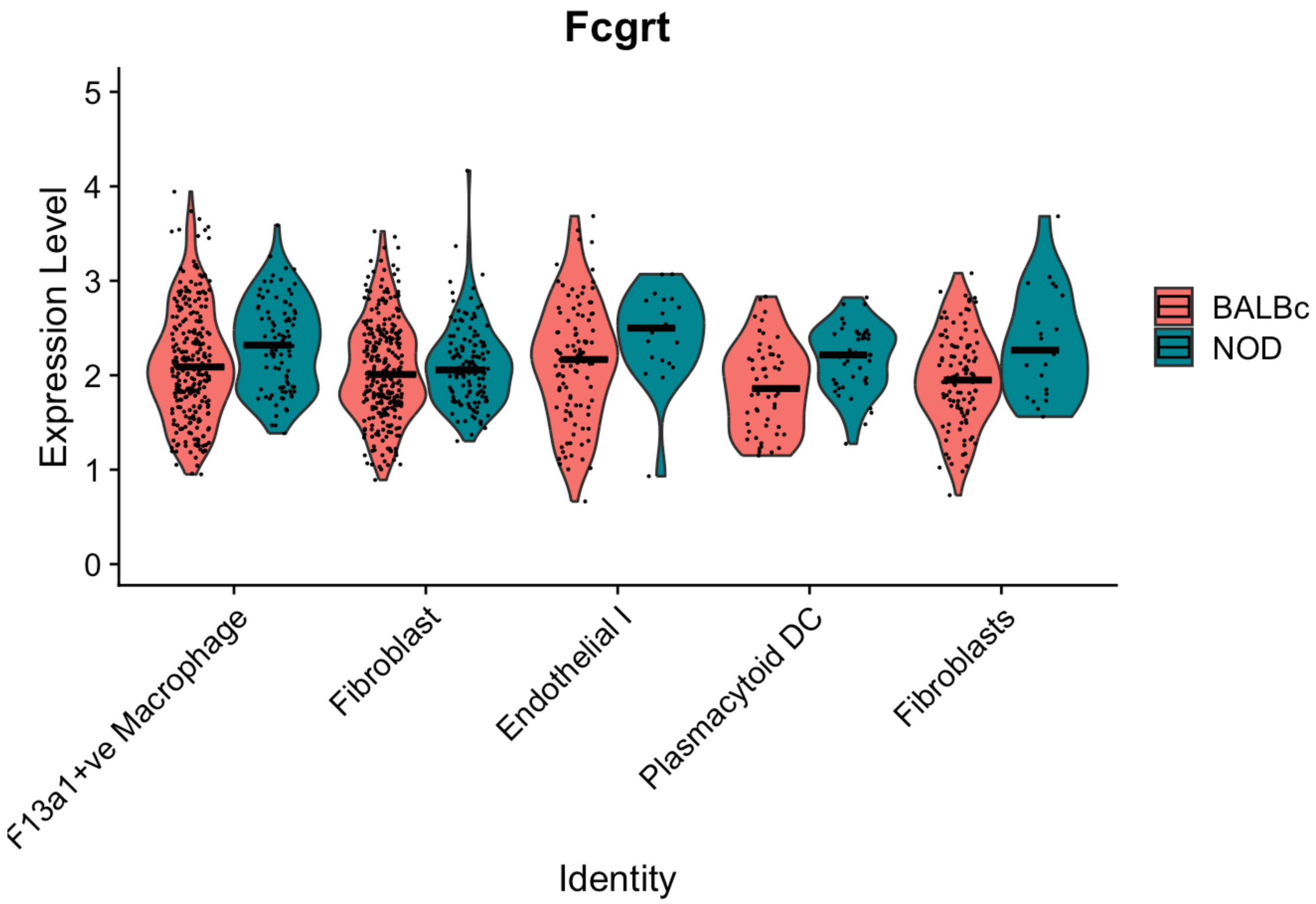


```
#dev.off()
```

#Cathepsin S expression

```
setwd("~/Documents/3_Parkinsons_disease/2019/scRNAseq/results/")
#tiff(filename = "Ctss_UMAP.tiff", height=4.5, width=9, res=300, units = "in")
FeaturePlot(seurat_integrated,
            reduction = "umap",
            features = c("Ctss"),
            order = TRUE,
            min.cutoff = 'q5',
            label = TRUE,
            label.size = 2,
            split.by = "sample")
```

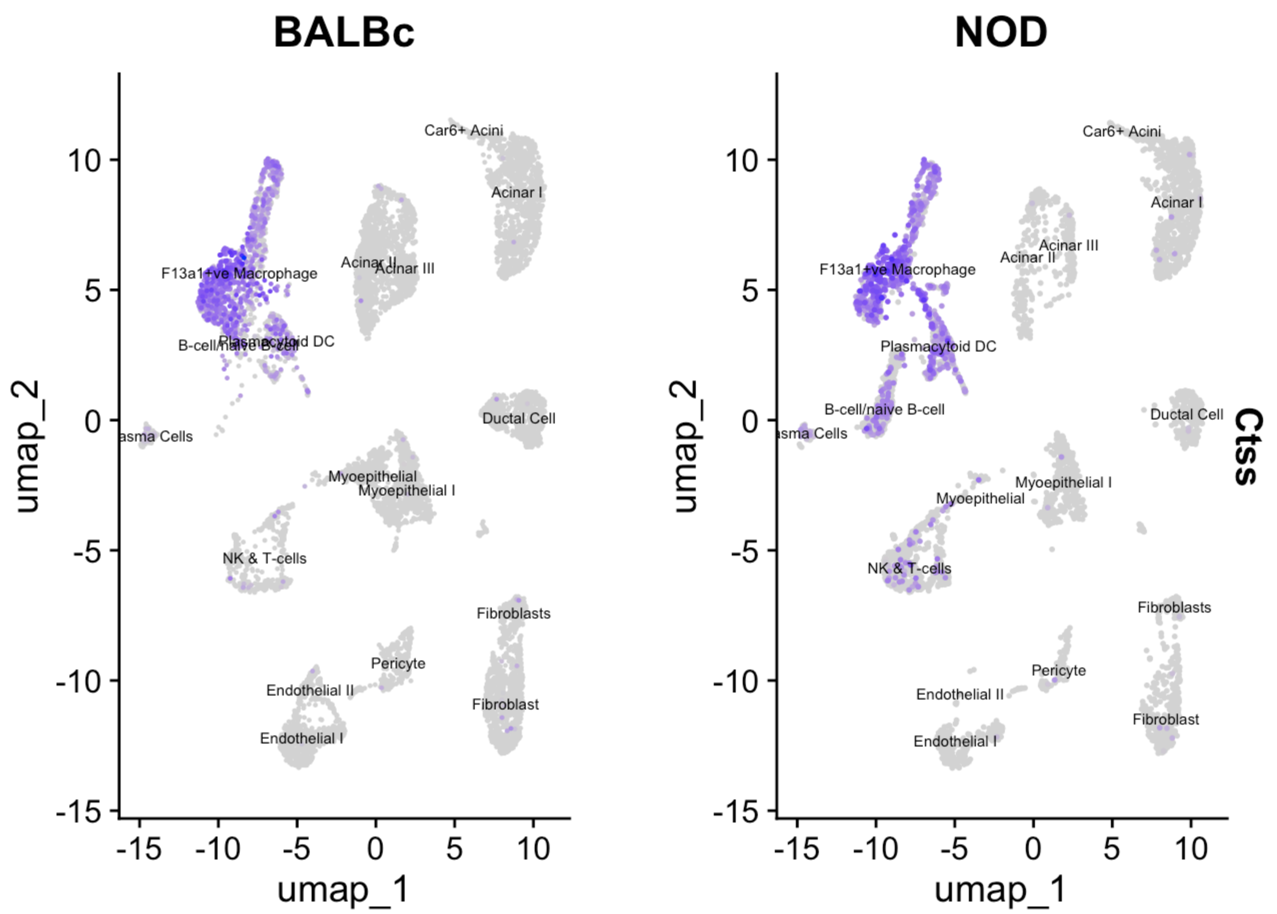


```
#dev.off()
```

```
setwd("~/Documents/3_Parkinsons_disease/2019/scRNAseq/results/")
#tiff(filename = "Ctss_Vln_full.tiff", height=5.5, width=11, res=300, units = "in")
your_font_size <- 5
symnum.args <- list(cutpoints = c(0, 0.00001, 0.0001, 0.001, 0.005, Inf), symbols = c("*
***", "***", "**", "*", "ns"))
a <- VlnPlot(seurat_integrated, features = 'Ctss', split.by = 'sample', pt.size = 0.25,
combine = T, split.plot = F, alpha = 0.3, log = TRUE) + theme_minimal() + theme(legend.t
itle = element_blank())  +
    stat_summary(fun = median, fun.min = median, fun.max = median,
                 geom = "crossbar",
                 width = 0.8,
                 position = position_dodge(width = .70)) +
   ylab("Gene Expression") +
   stat_compare_means( method = "anova", paired = T, symnum.args=symnum.args, label =
"p.signif") +
   #stat_compare_means(size = your_font_size, label = "p.signif") +
   theme(text = element_text(size = 12),
         axis.text.x = element_text(size = 12, angle = 45)) +
    guides(fill = guide_legend(override.aes = list(linetype = 0)),
           color = guide_legend(override.aes = list(linetype = 0))) +
   ylim(0,7)
```

```
## Scale for y is already present.
## Adding another scale for y, which will replace the existing scale.
```

```
print(a)
```

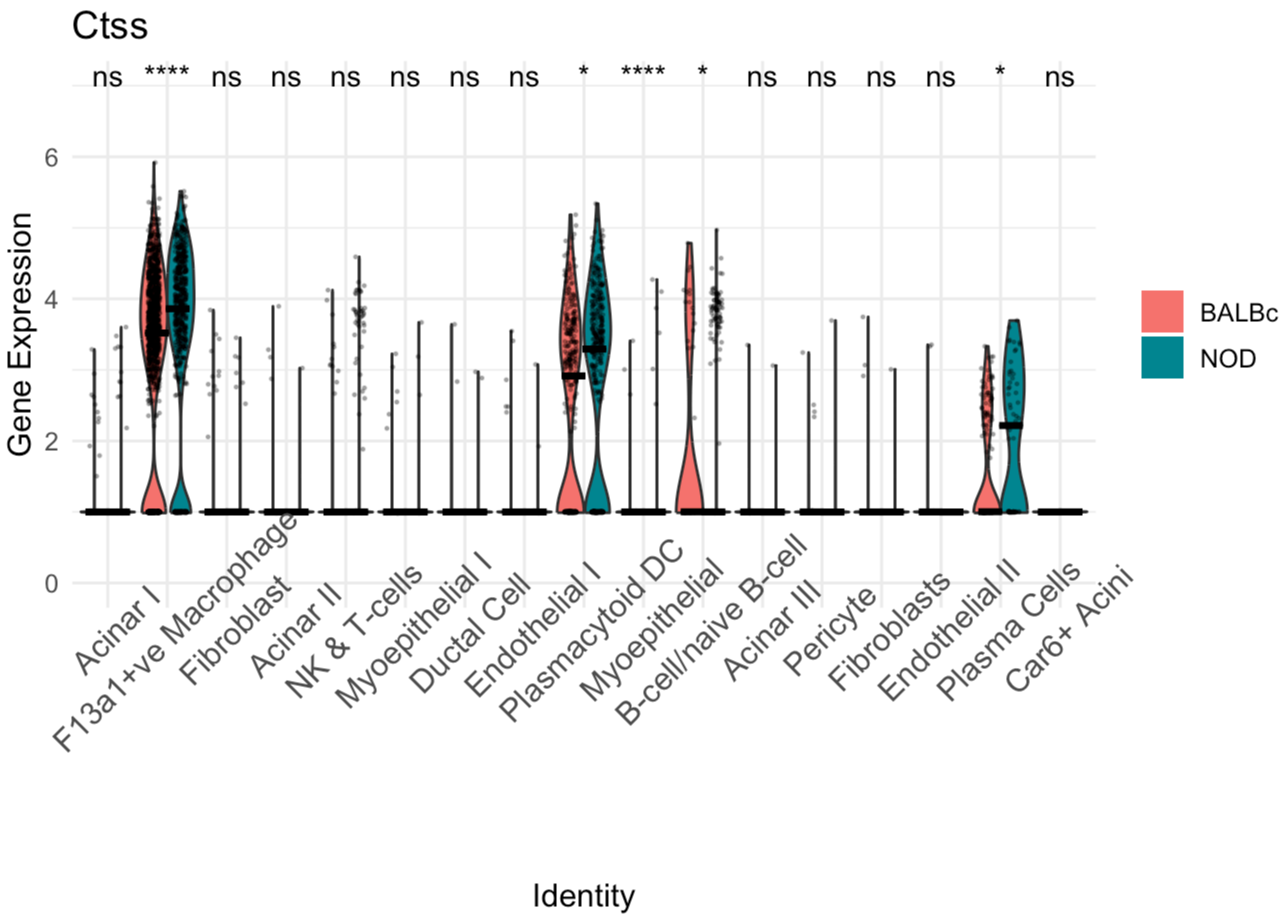


```
#dev.off()
```

Igha Violin Plot

```
setwd("~/Documents/3_Parkinsons_disease/2019/scRNAseq/results/")
#tiff(filename = "Igha_Vln_full.tiff", height=3.75, width=4, res=300, units = "in")
your_font_size <- 7.5
symnum.args <- list(cutpoints = c(0, 0.00001, 0.0001, 0.001, 0.01, Inf), symbols = c("**
**", "***", "**", "*", "ns"))
a <- VlnPlot(seurat_integrated,
             features = 'Igha',
             idents = c("Plasma Cells", "B-cell/naive B-cell"),
             split.by = 'sample',
             layer = "data",
             pt.size = 0.25,
             combine = T,
             split.plot = F,
             alpha = 0.3,
             log = FALSE) +
   theme_minimal() +
   theme(legend.title = element_blank())  +
    stat_summary(fun = median, fun.min = median, fun.max = median,
                 geom = "crossbar",
                 width = 0.4,
                 position = position_dodge(width = .70)) +
   ylab("Gene Expression") +
   stat_compare_means( method = "anova", paired = T, symnum.args=symnum.args, label =
"p.signif") +
   #stat_compare_means(size = your_font_size, label = "p.signif") +
   theme(text = element_text(size = 12),
         axis.text.x = element_text(size = 12, angle = 15)) +
    guides(fill = guide_legend(override.aes = list(linetype = 0)),
           color = guide_legend(override.aes = list(linetype = 0))) +
   ylim(0,10)
```

```
## Scale for y is already present.
## Adding another scale for y, which will replace the existing scale.
```

```
print(a)
```

```
## Warning: Removed 306 rows containing non-finite outside the scale range
## (`stat_ydensity()`).
```

```
## Warning: Removed 306 rows containing non-finite outside the scale range
## (`stat_summary()`).
```

```
## Warning: Removed 306 rows containing non-finite outside the scale range
## (`stat_compare_means()`).
```

```
## Warning: Removed 306 rows containing missing values or values outside the scale range
## (`geom_point()`).
```

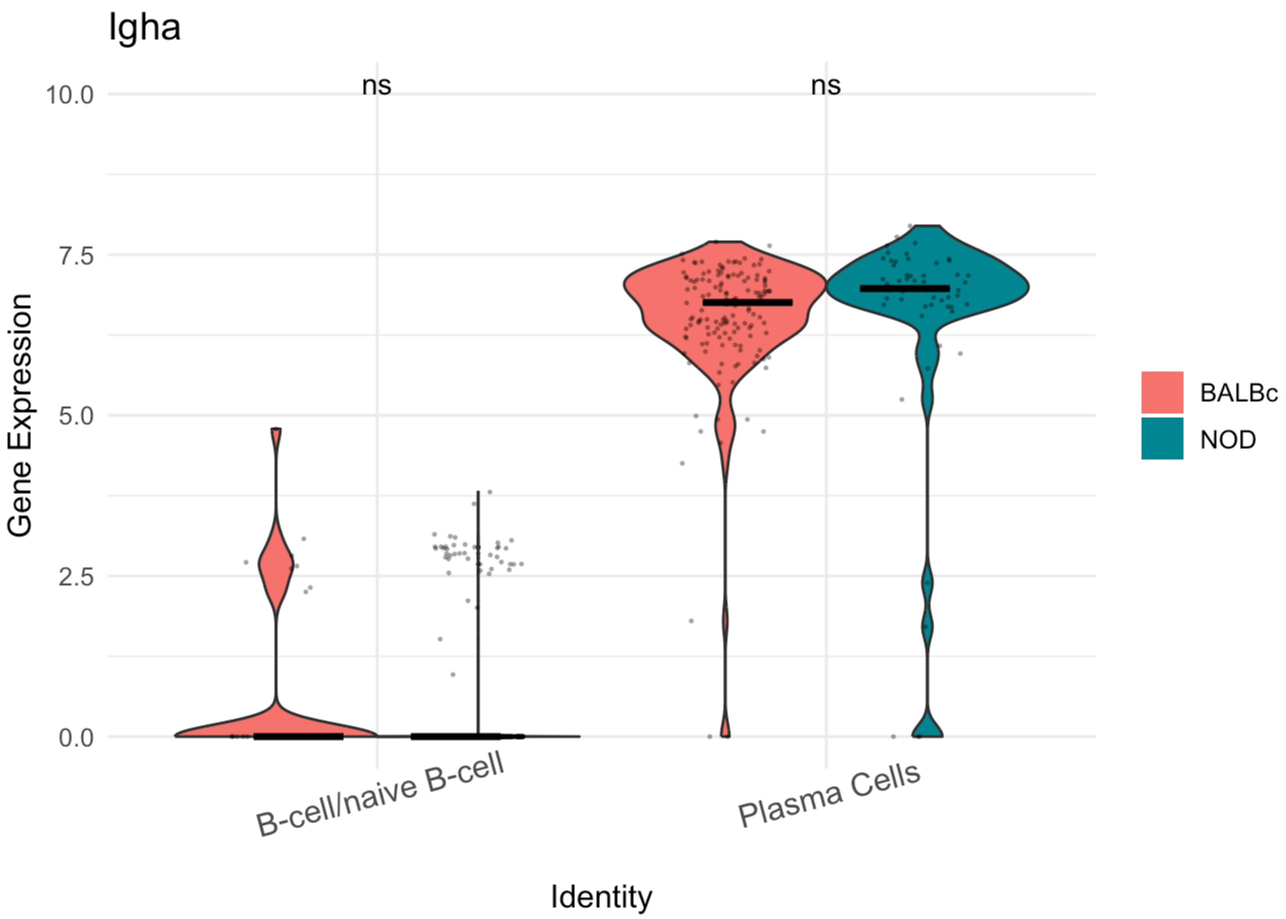
Igha
ns
ns
10.0
7.5
5.0
2.5
0.0
Gene Expression
B-cell/naive B-cell
Plasma Cells
Identity
BALBc
NOD

```
#dev.off()
```

```
setwd("~/Documents/3_Parkinsons_disease/2019/scRNAseq/results/")
#tiff(filename = "Ighg2_Vln_full.tiff", height=3.75, width=4, res=300, units = "in")
your_font_size <- 7.5
symnum.args <- list(cutpoints = c(0, 0.00001, 0.0001, 0.001, 0.01, Inf), symbols = c("**
**", "***", "**", "*", "ns"))
a <- VlnPlot(seurat_integrated,
             features = 'Ighg2b',
             idents = c("Plasma Cells", "B-cell/naive B-cell"),
             split.by = 'sample',
             layer = "data",
             pt.size = 0.25,
             combine = T,
             split.plot = F,
             alpha = 0.3,
             log = FALSE) +
   theme_minimal() +
   theme(legend.title = element_blank())  +
    stat_summary(fun = median, fun.min = median, fun.max = median,
                 geom = "crossbar",
                 width = 0.4,
                 position = position_dodge(width = .70)) +
   ylab("Gene Expression") +
   stat_compare_means( method = "anova", paired = T, symnum.args=symnum.args, label =
"p.signif") +
   #stat_compare_means(size = your_font_size, label = "p.signif") +
   theme(text = element_text(size = 12),
         axis.text.x = element_text(size = 12, angle = 15)) +
    guides(fill = guide_legend(override.aes = list(linetype = 0)),
           color = guide_legend(override.aes = list(linetype = 0))) +
   ylim(0,10)
```

```
## Scale for y is already present.
## Adding another scale for y, which will replace the existing scale.
```

```
print(a)
```

```
## Warning: Removed 421 rows containing non-finite outside the scale range
## (`stat_ydensity()`).
```

```
## Warning: Removed 421 rows containing non-finite outside the scale range
## (`stat_summary()`).
```

```
## Warning: Removed 421 rows containing non-finite outside the scale range
## (`stat_compare_means()`).
```

```
## Warning: Removed 421 rows containing missing values or values outside the scale range
## (`geom_point()`).
```

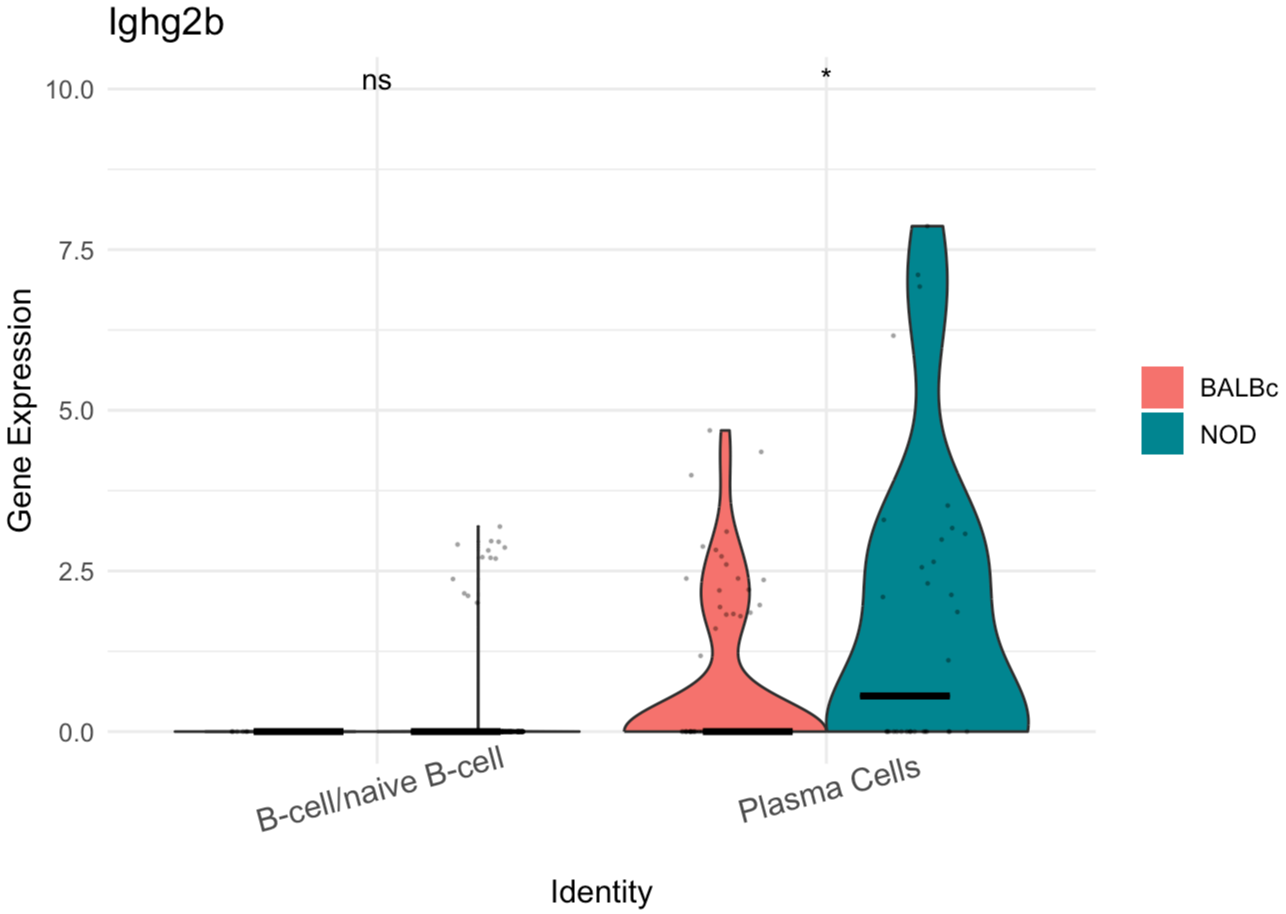
Ighg2b
ns
*
10.0
7.5
5.0
2.5
0.0
Gene Expression
B-cell/naive B-cell
Plasma Cells
Identity
BALBc
NOD

```
#dev.off()
```

```
setwd("~/Documents/3_Parkinsons_disease/2019/scRNAseq/results/")
#tiff(filename = "Ighm_Vln_full.tiff", height=3.75, width=4, res=300, units = "in")
your_font_size <- 7.5
symnum.args <- list(cutpoints = c(0, 0.00001, 0.0001, 0.001, 0.01, Inf), symbols = c("**
**", "***", "**", "*", "ns"))
a <- VlnPlot(seurat_integrated,
             features = c('Ighm'),
             idents = c("Plasma Cells", "B-cell/naive B-cell"),
             split.by = 'sample',
             layer = "data",
             pt.size = 0.25,
             combine = T,
             split.plot = F,
             alpha = 0.3,
             log = FALSE) +
   theme_minimal() +
   theme(legend.title = element_blank())  +
    stat_summary(fun = median, fun.min = median, fun.max = median,
                 geom = "crossbar",
                 width = 0.4,
                 position = position_dodge(width = .70)) +
   ylab("Gene Expression") +
   stat_compare_means( method = "anova", paired = T, symnum.args=symnum.args, label =
"p.signif") +
   #stat_compare_means(size = your_font_size, label = "p.signif") +
   theme(text = element_text(size = 12),
         axis.text.x = element_text(size = 12, angle = 15)) +
    guides(fill = guide_legend(override.aes = list(linetype = 0)),
           color = guide_legend(override.aes = list(linetype = 0))) +
   ylim(0,10)
```

```
## Scale for y is already present.
## Adding another scale for y, which will replace the existing scale.
```

```
print(a)
```

```
## Warning: Removed 333 rows containing non-finite outside the scale range
## (`stat_ydensity()`).
```

```
## Warning: Removed 333 rows containing non-finite outside the scale range
## (`stat_summary()`).
```

```
## Warning: Removed 333 rows containing non-finite outside the scale range
## (`stat_compare_means()`).
```

```
## Warning: Removed 333 rows containing missing values or values outside the scale range
## (`geom_point()`).
```

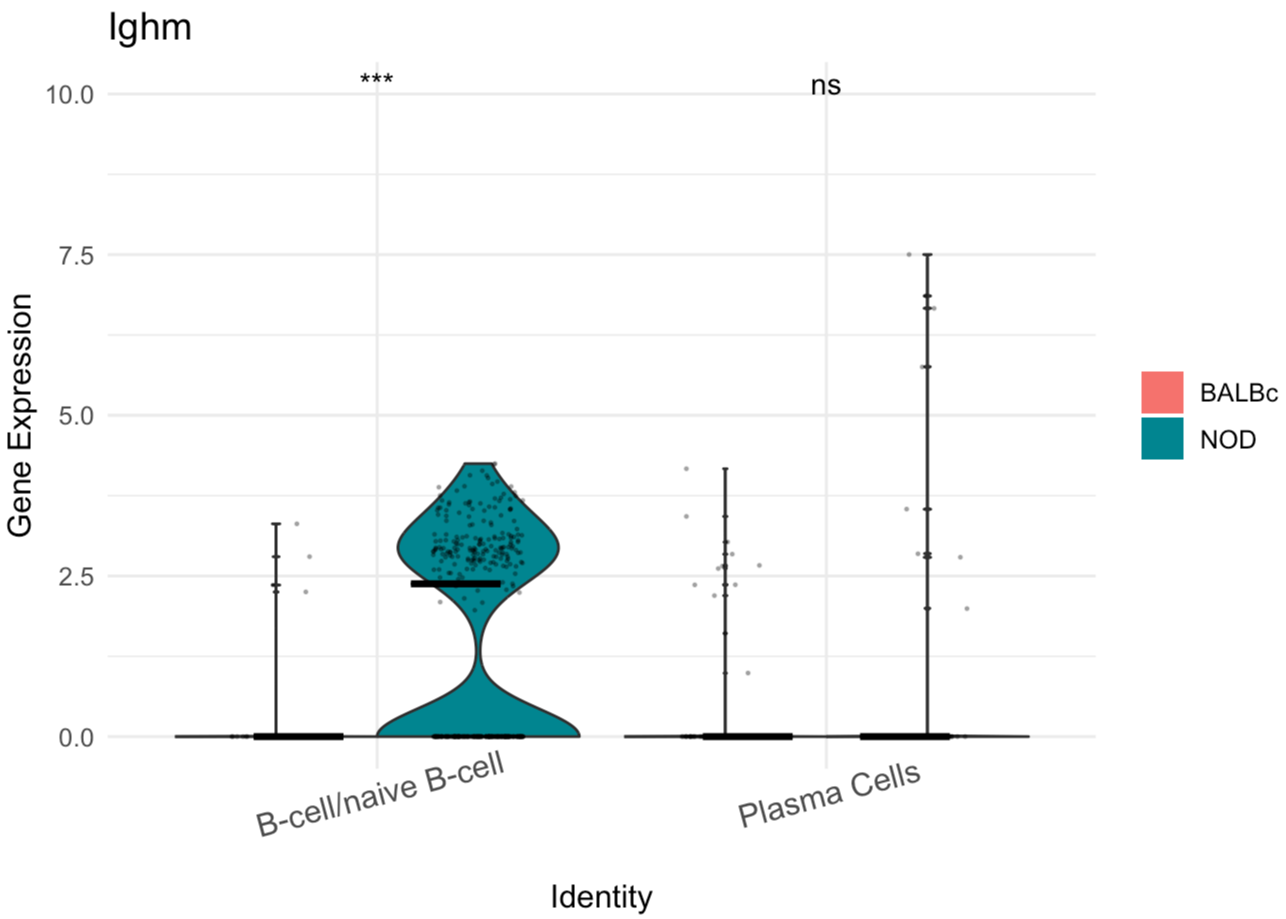


```
#dev.off()
```

```
setwd("~/Documents/3_Parkinsons_disease/2019/scRNAseq/results/")
#tiff(filename = "Germinal_Center1.tiff", height=6, width=7, res=300, units = "in")
FeaturePlot(seurat_integrated,
            reduction = "umap",
            features = c("Ighd", "Cd83"),
            order = TRUE,
            min.cutoff = 'q5',
            label = TRUE,
            label.size = 2,
            split.by = "sample")
```

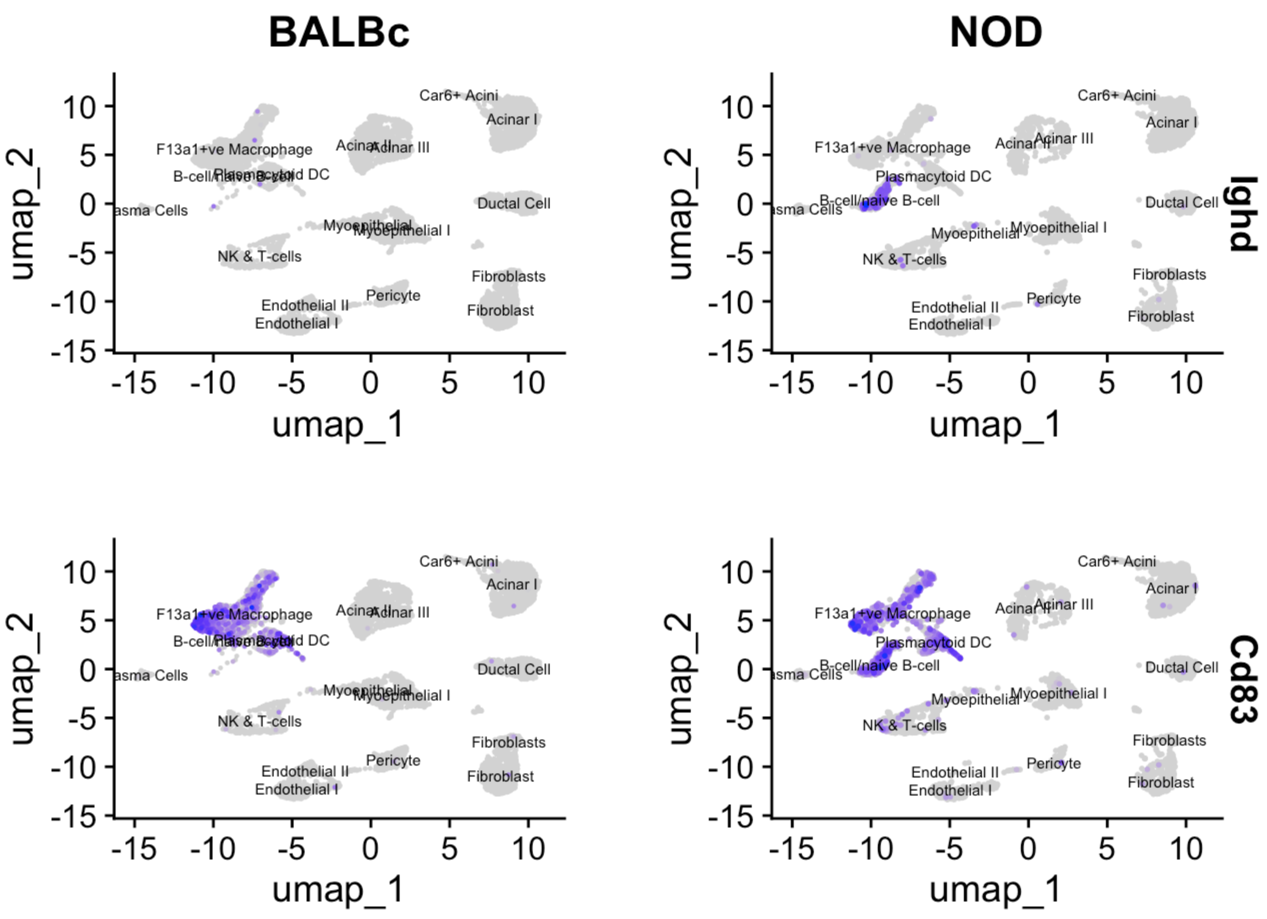


```
#dev.off()

setwd("~/Documents/3_Parkinsons_disease/2019/scRNAseq/results/")
#tiff(filename = "Germinal_Center2.tiff", height=6, width=7, res=300, units = "in")
FeaturePlot(seurat_integrated,
            reduction = "umap",
            features = c("Ighd", "Ntan1"),
            order = TRUE,
            min.cutoff = 'q10',
            label = TRUE,
            label.size = 2,
            split.by = "sample")
```

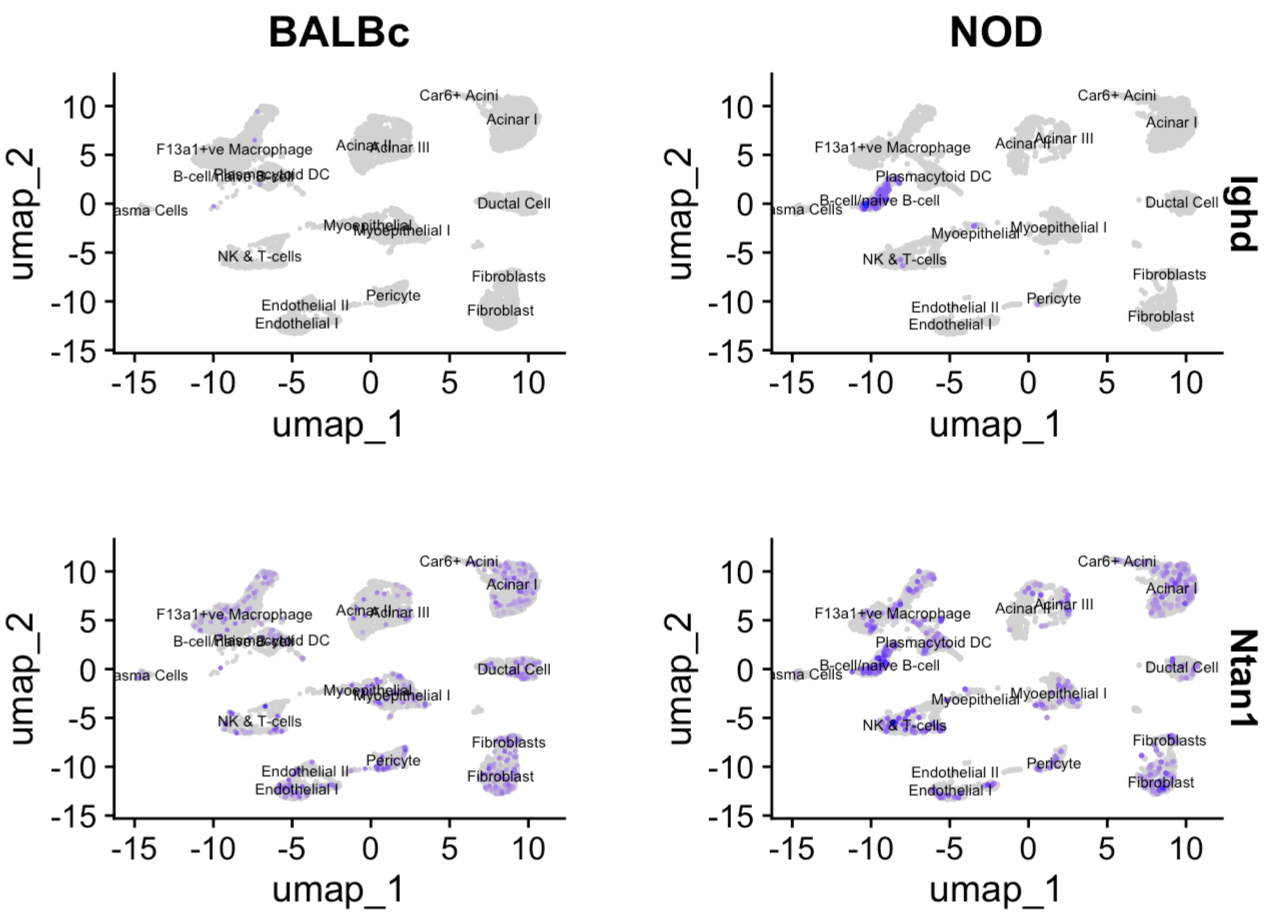


```
#dev.off()

VlnPlot(object = seurat_integrated,
        #idents="B-cell/naive B-cell",
        features = c("Ntan1"),
        slot = "data",
        layer="counts",
        split.by = "sample") + ylim(0,5)
```

```
## Warning: The `slot` argument of `VlnPlot()` is deprecated as of Seurat 5.0.0.
## ℹ Please use the `layer` argument instead.
## This warning is displayed once every 8 hours.
## Call `lifecycle::last_lifecycle_warnings()` to see where this warning was
## generated.
```

```
## Scale for y is already present.
## Adding another scale for y, which will replace the existing scale.
```

```
## Warning: Removed 6218 rows containing non-finite outside the scale range
## (`stat_ydensity()`).
```

```
## Warning: Removed 6218 rows containing missing values or values outside the scale rang
e
## (`geom_point()`).
```

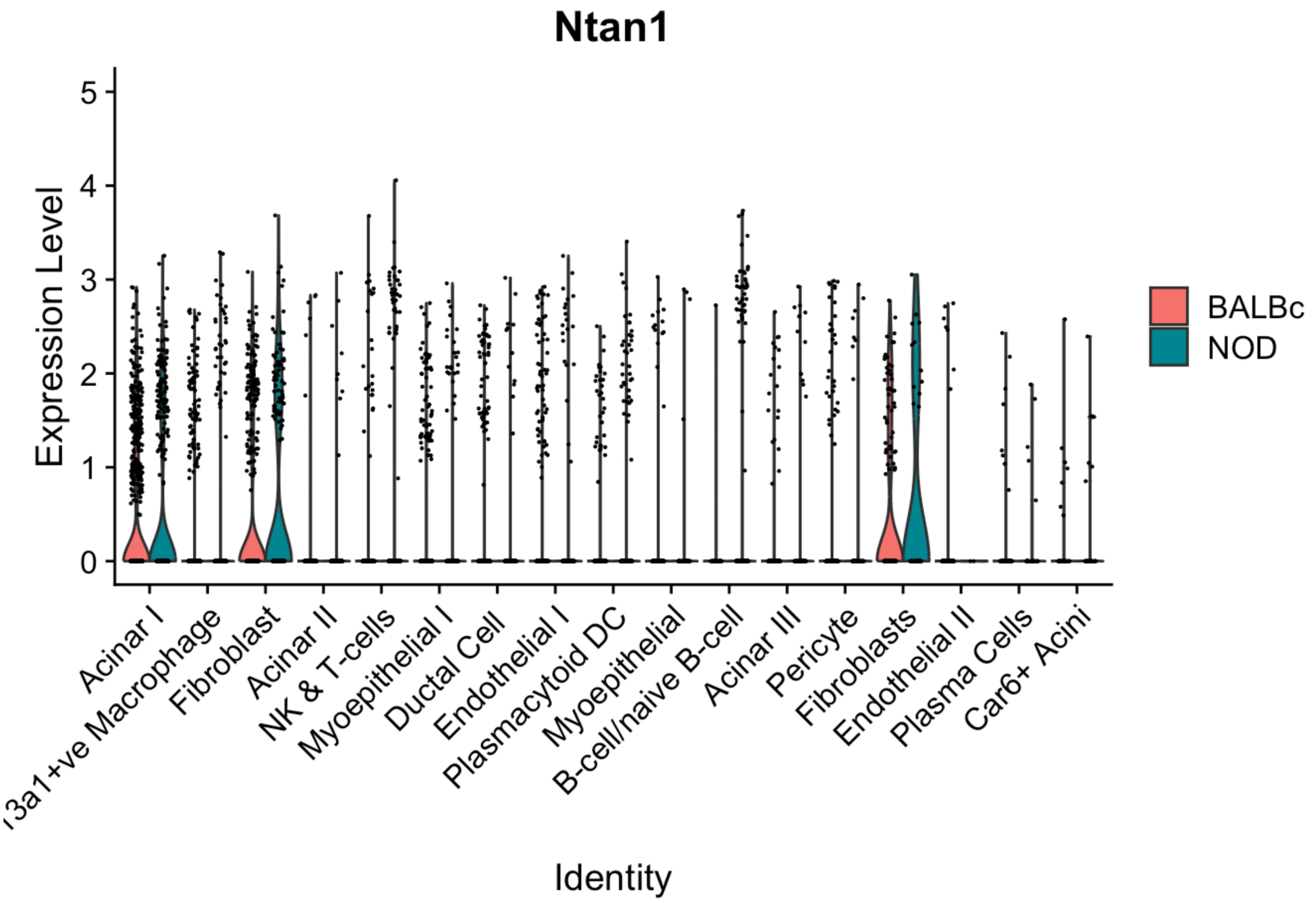


```
VlnPlot(object = seurat_integrated,
        idents="B-cell/naive B-cell",
        features = c("Ighd"),
        slot = "data",
        layer="data",
        split.by = "sample") + ylim(0,5)
```

```
## Scale for y is already present.
## Adding another scale for y, which will replace the existing scale.
```

```
## Warning: Removed 282 rows containing non-finite outside the scale range
## (`stat_ydensity()`).
```

```
## Warning: Removed 282 rows containing missing values or values outside the scale range
## (`geom_point()`).
```

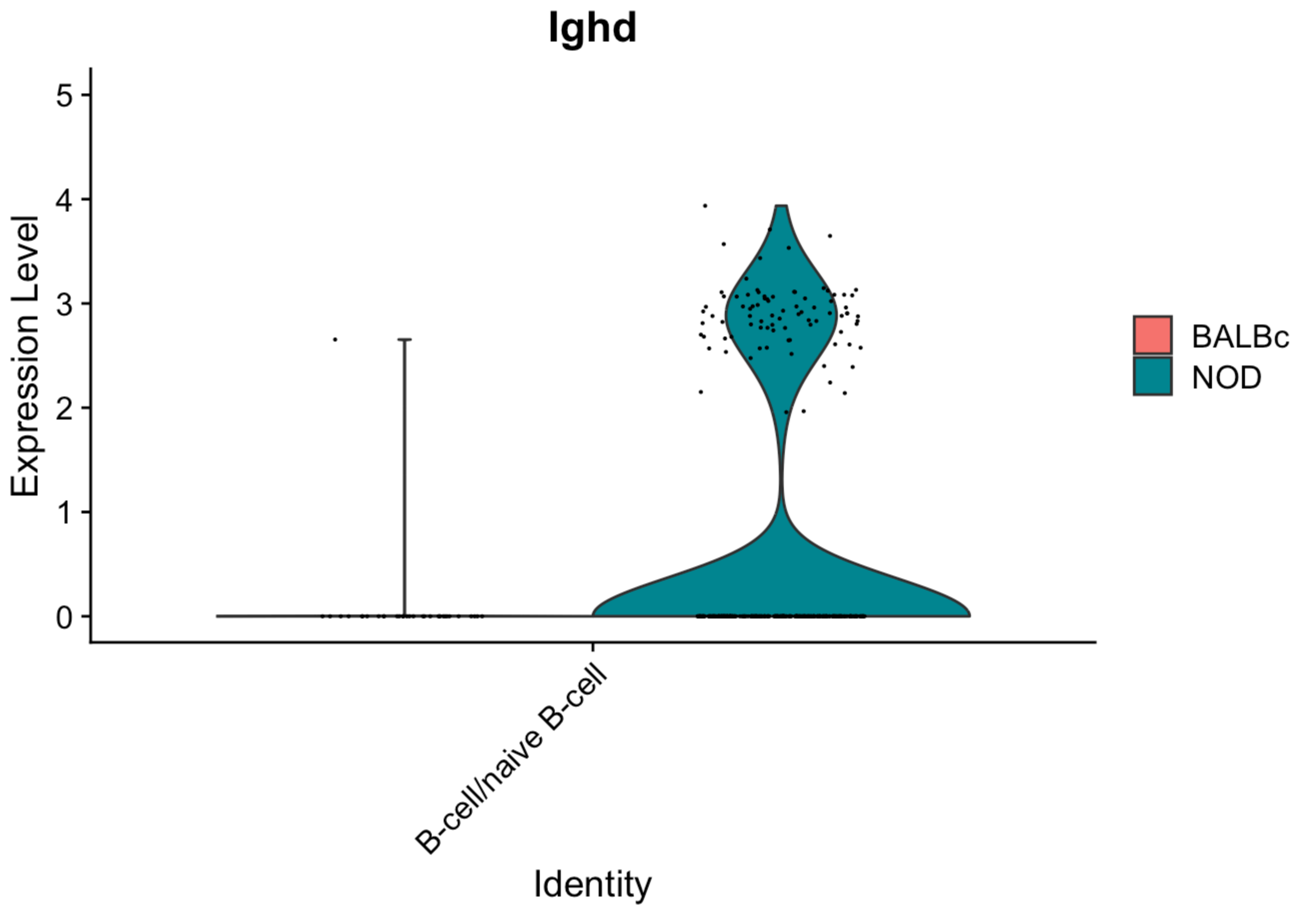


```
setwd("~/Documents/3_Parkinsons_disease/2019/scRNAseq/results/")
#tiff(filename = "Germinal_Center.tiff", height=13, width=7, res=300, units = "in")
FeaturePlot(seurat_integrated,
            reduction = "umap",
            features = c("Cd19", "Cd22", "Cxcr4"),
            order = TRUE,
            min.cutoff = 'q5',
            label = TRUE,
            label.size = 2,
            split.by = "sample")
```

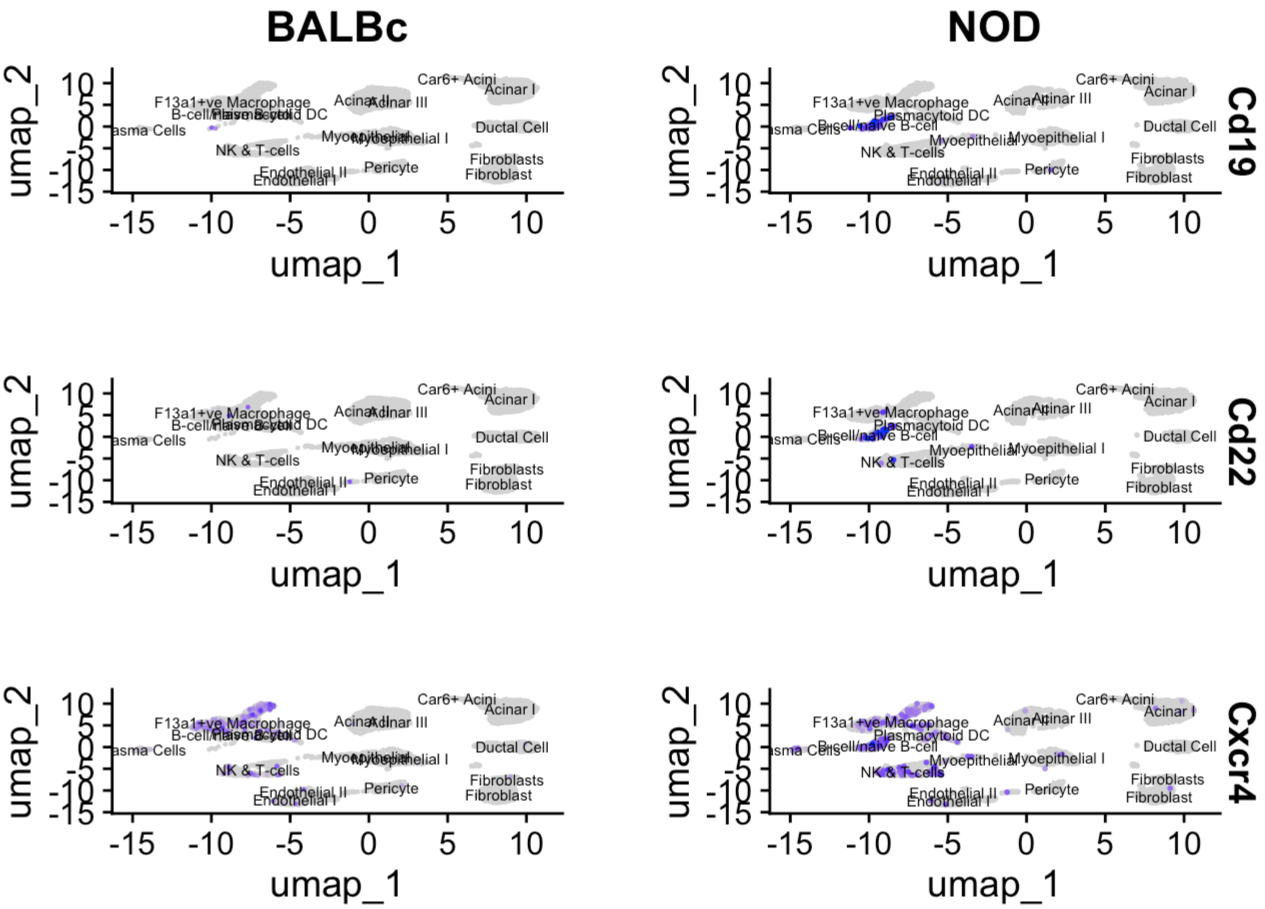


```
#dev.off()
```

```
VlnPlot(object = seurat_integrated,
        #idents="B-cell/naive B-cell",
        features = c("Ighg2b"),
        split.by = "sample") + ylim(0,5)
```

```
## Scale for y is already present.
## Adding another scale for y, which will replace the existing scale.
```

```
## Warning: Removed 6790 rows containing non-finite outside the scale range
## (`stat_ydensity()`).
```

```
## Warning: Removed 6790 rows containing missing values or values outside the scale rang
e
## (`geom_point()`).
```

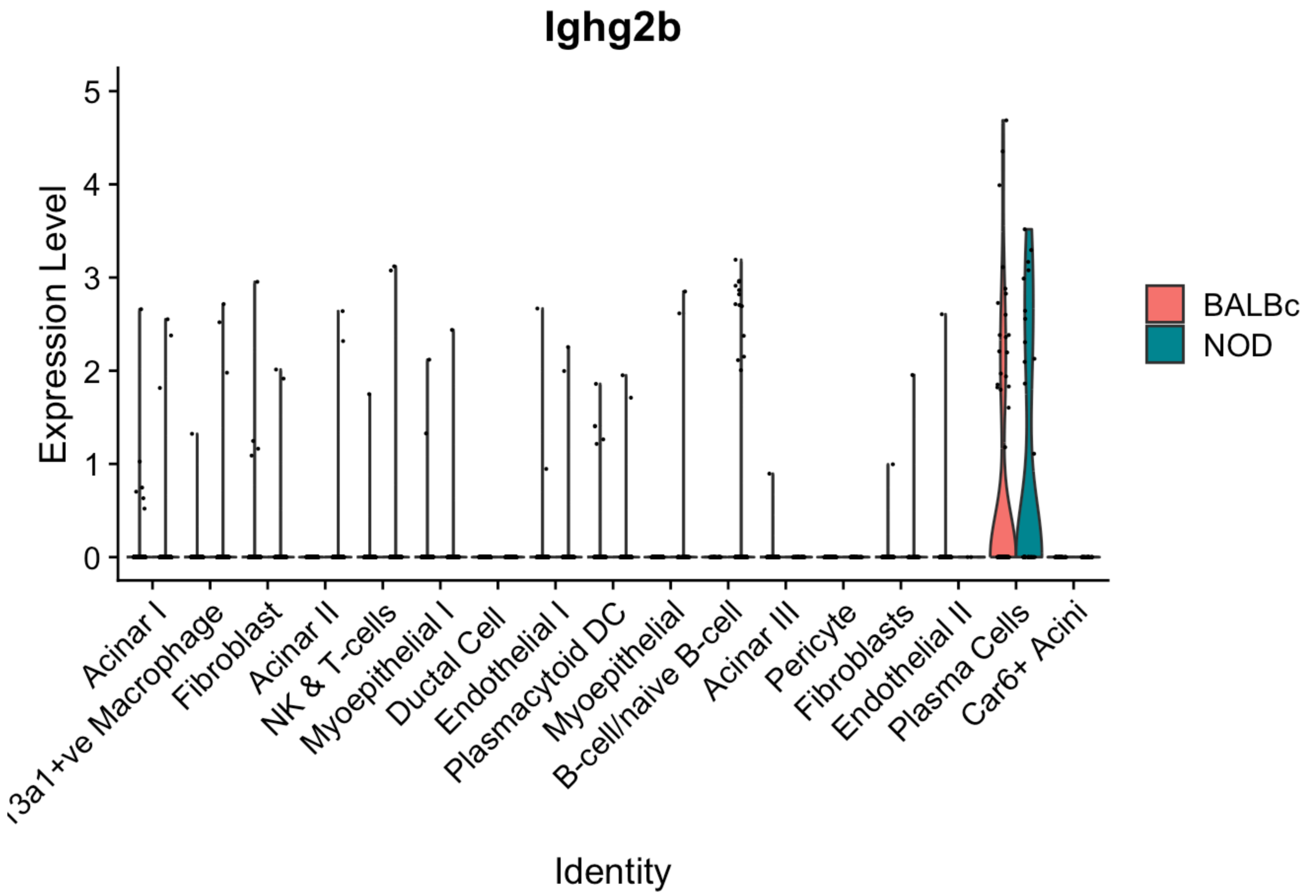


```
#setwd("~/Documents/3_Parkinsons_disease/2019/scRNAseq/results/")
#tiff(filename = "IgG_Production.tiff", height=4, width=8, res=300, units = "in")
VlnPlot(object = seurat_integrated,
        #idents=c("Plasma Cells", "B-cell/naive B-cell"),
        features = c("Igha", "Ighg2b", "Ighm"),
        slot = "data",
        layer="counts",
        split.by = "sample") +
   stat_summary(fun = median, fun.min = median, fun.max = median,
                geom = "crossbar",
                width = 0.6,
                position = position_dodge(width = .70)) +
   xlab("Clusters") +
   ylab("Gene Expression") +
   theme(legend.title = element_blank())
```

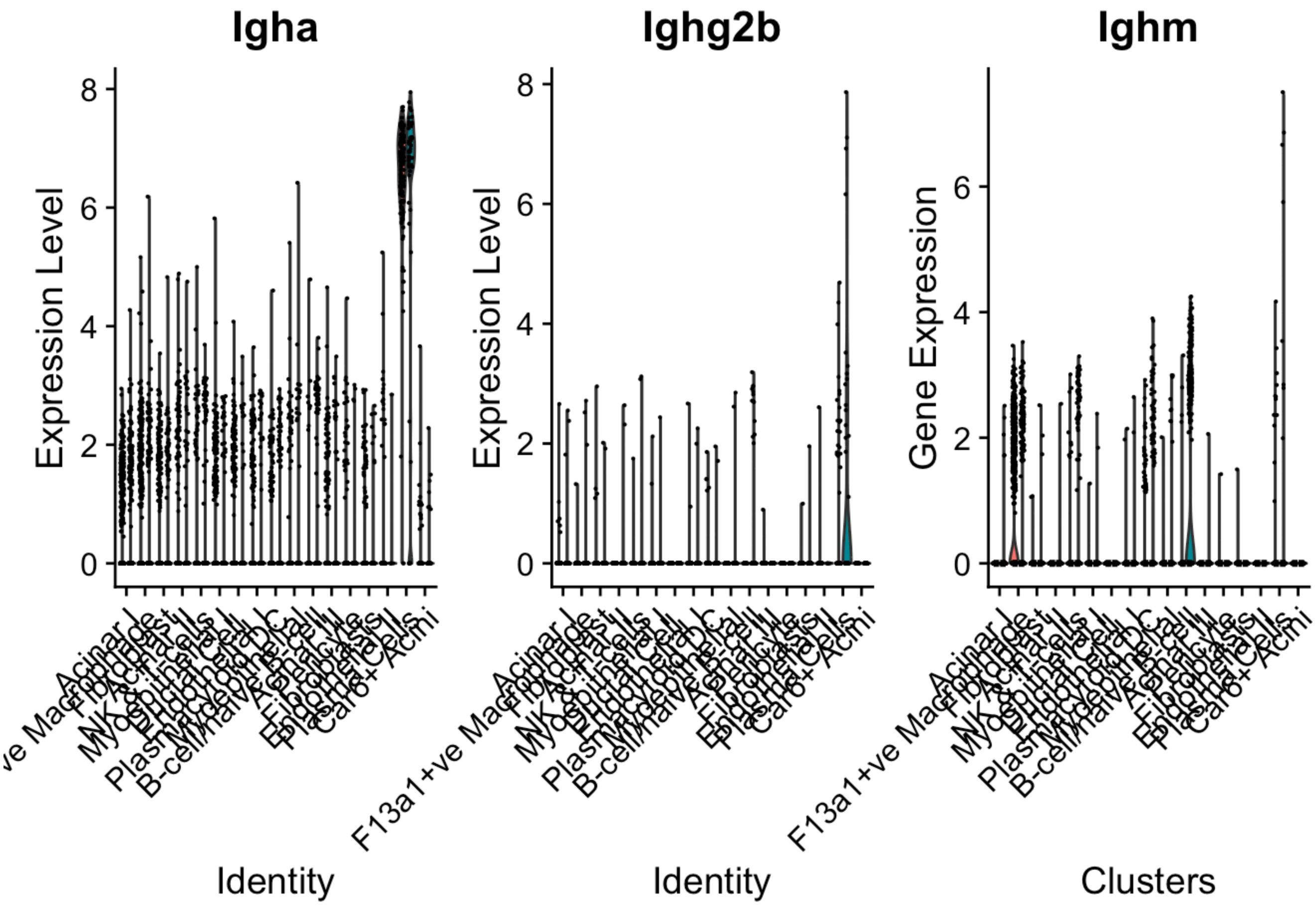


```
#dev.off()
#+ stat_compare_means(x="sample", y="Expression Level", aes(label = paste0("p = ", after
_stat(p.format))), method = "wilcox.test", paired = T, size = 4, label = "p.signif") +
#   theme(text = element_text(size = 20, angle = 45), legend.title = element_blank()) +
#   guides(fill = guide_legend(override.aes = list(linetype = 0)),
#          color = guide_legend(override.aes = list(linetype = 0)))
```
